# Transcriptome analysis of *Arabidopsis* mutants suggests a crosstalk between ABA, ethylene and GSH against combined cold and osmotic stress

**DOI:** 10.1038/srep36867

**Published:** 2016-11-15

**Authors:** Deepak Kumar, Saptarshi Hazra, Riddhi Datta, Sharmila Chattopadhyay

**Affiliations:** 1 Plant Biology Laboratory, Organic & Medicinal Chemistry Division, CSIR- Indian Institute of Chemical Biology, 4, Raja S.C. Mullick Road, Kolkata 700032, India

## Abstract

The involvement of ethylene and abscisic acid in providing stress tolerance and defence response to plants is widely recognized. However, little is known about the cross-talk between glutathione with ethylene and abscisic acid to combat stress *in planta*. Here, transcriptome analysis of combined cold and osmotic stress treated *Arabidopsis* mutants were carried out to elucidate the crosstalk between the abscisic acid, ethylene and glutathione. Microarray experiment revealed the differential regulation of about 2313 and 4131 transcripts in *ein*2 (ethylene insensitive mutant) and *aba*1.6 (abscisic acid mutant) respectively. Functional analysis exposed common down-regulated stress and defence, secondary metabolite biosynthesis viz. phenylpropanoid, lignin and flavonols, redox and transcription factors related genes in *ein*2, *aba*1.6 and *pad2*.1 (glutathione mutant) in response to combined stress treatment. The reduced glutathione content was less in stress treated mutants in comparison to Col-0. Again, selective down-regulated transcripts in stress treated mutants were noted up-regulated after glutathione feeding. Some of the important differentially expressed genes were also validated by comparative proteomics analysis of stress treated mutants. In summary, our results suggested the role of ethylene and abscisic acid in inducing stress-responsive genes and proteins by activating glutathione biosynthesis to combat abiotic stress conditions in plant system.

Plants are consistently exposed to suboptimal climatic and edaphic conditions that adversely affect cellular homeostasis and that ultimately impair their growth and fitness^1^. For survival, plants are bound to respond and adapt efficiently to their ever-changing environmental circumstances. Environmental stresses often take place in combination or in succession, so survival in variable environment can involve the development of multiple mechanisms. Crop productivity will enhance if the yield loss will be minimized in response to abiotic stress. The molecular mechanisms linked with signal transduction, leading to alter the gene expression in order to combat environmental stress, are largely unknown.

The role of abscisic acid in the regulation of key processes relevant to seed germination, plant development, biotic and abiotic stress responses is an established fact^2^. Abiotic stress condition such as drought induces abscisic acid biosynthesis initiating the signaling pathways that lead to a number of molecular and cellular responses, among which the best known are the expression of stress-related genes and proteins. In response to drought stress abscisic acid is a key player that control water status and induces stomatal closure for conserving the water^3^,^4^. Abscisic acid also plays an important role in response to pathogen attack, and the signaling pathways are related significantly between pathogen resistance and abiotic stress tolerance. Role of abscisic acid in stomatal closure also limits pathogen access and also affects pathogen responses by interacting with other hormones associated with plant defence mechanisms^5^. As a phytohormone, ethylene is known for inducing a triple response in seedlings, leaf abscission and other responses to various stresses^6^. The study of various ethylene mutants in *Arabidopsis (Arabidopsis thaliana*), tobacco (*Nicotiana tabacum*), and soybean (*Glycine max*) have revealed that ethylene signaling is required for resistance against some specific pathogens such as *Botrytis cinerea* but not to others^7^,^8^. Ethylene has also played a key role in combating salt, drought, and heat stress by gene-specific regulations by inducing the ethylene response factor1 (*ERF*1) which plays a nodal role in integrating the ethylene and jasmonic acid signaling pathways^9^. Ethylene biosynthesis is also induced by salt stress, which then inhibit its receptors, suppress salt sensitivity conferred by ethylene receptors, and promote ethylene-responsive salt tolerance^10^,^11^. The role of *ethylene insensitive*2 (*EIN*2) gene acts as a functional transducer of ethylene and stress responses in *Arabidopsis*. It also plays a vital role in the ethylene signaling pathway^12^.

Recent investigation also reported the role of abscisic acid in enhancing the tolerance of wheat seedlings against drought and regulating the transcript levels of genes encoding ascorbate-glutathione biosynthesis^13^. The role of glutathione in regulating ACC synthase to induce ethylene in stress condition has already been established in our lab^14^. Ethylene and salicylic acid regulated the glutathione biosynthesis in ozone-exposed *A. thaliana*^15^. Ethylene also has a vital role in providing protection against heat stress-induced oxidative damage along with calcium, abscisic acid, and salicylic acid^16^.

Our previous study revealed the down-regulation of various stresses and defence related genes and proteins in combined stress treated glutathione mutant *pad*2.1^17^. In our present investigation, we have identified various stress and defence, transcription factors, redox related genes and proteins in addition to various secondary metabolite biosynthetic pathway genes which are supposed to be directly or indirectly induced by abscisic acid and ethylene in response to combined cold and osmotic stress treatment. We have also identified most common down-regulated genes and proteins in *pad*2.1, *ein*2 and *aba*1.6 in response to combined abiotic stress, which suggested a crosstalk between glutathione, abscisic acid and ethylene in inducing these genes and proteins to combat stress conditions in plant system.

## Results

### Confirmation of stress response

**Figure 1:**
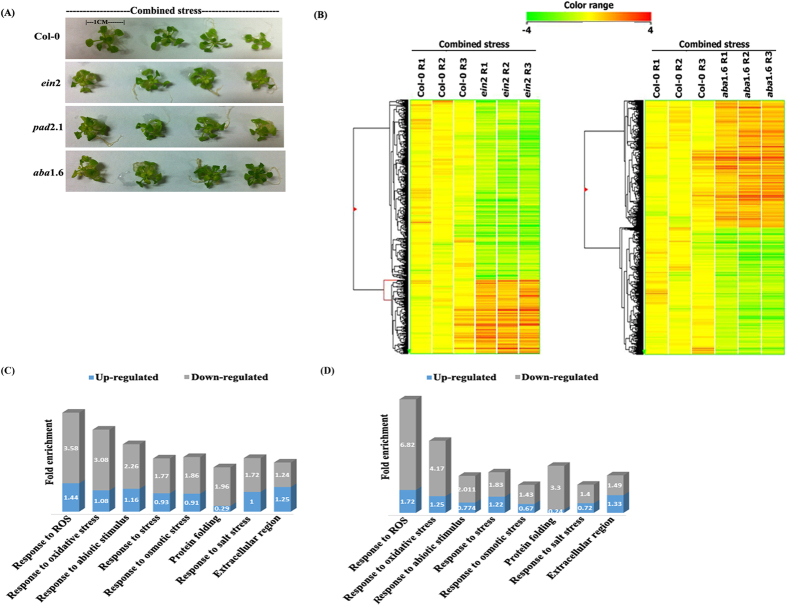
Morphological change of leaves and hierarchical cluster tree with heat map for differentially expressed genes in Col-0, *ein*2 and *aba*1.6 in response to combined stress with Significant enrichment analysis. (**A**) Effect of 6 hr combined stress treatment on the morphology of leaves (**B**) Classification of tree is based on gene expression. Up- (log2 fold change >= 0.60) and down-regulated (log_2_ fold change <= −0.50) genes are shown by red and green colour respectively. By using DAVID^18^, significant enrichment analysis of functional annotated categories for differentially expressed genes in combined stress treated (**C**) *aba*1.6 and (**D**) *ein*2 has been shown.

In response to the combined stress treatment in *ein*2 and *aba*1.6 a distinguished morphological change was noted in comparison to the combined stress treated Col-0. Leaves of stress treated mutants were more wilted in comparison to that of Col-0 (Fig. 1A).

### Microarray experiment

The transcript changes in response to stress treated *ein*2 and *aba*1.6 in comparison to stress treated Col-0 were investigated by microarray experiment. Substantial differences in transcript level responses were observed in stress treated *ein*2 and *aba*1.6 in comparison to stress treated Col-0. Quantification of images and analysis of raw data was observed using the Agilent Feature Extraction Software and Agilent GeneSpring GX Software respectively. The data was normalized in GeneSpring GX using the 75^th^ percentile shift (Percentile shift normalization is a global normalization, where the locations of all the spot intensities in an array are adjusted). In an independent experiment the normalization took each column and the percentile of the expression value for this array across all spots was computed (where n has the range from 0–100 and n = 75 is the median). Subtraction of this value from the expression value of each entity was performed and was normalized to specific control samples. Microarray data revealed the differential regulation of about 2313 genes in *ein*2 amongst which 1156 and 1157 genes were significantly up- (log_2_ fold change >= 0.60) and down-regulated (log_2_ fold change <= −0.50) respectively (Supplementary Table S1). In combined stress treated *aba*1.6 about 4131 genes were found differentially regulated amongst which 2072 and 2059 genes were significantly up-and down-regulated respectively (Supplementary Table S2). Using hierarchical heat map image, the gene expression profile of combined stress treated *ein*2 and *aba*1.6 has been shown (Fig. 1B). Student’s *t*-test was used for measuring the statistical significance of differentially expressed genes.

### DAVID and MAPMAN analysis of differentially expressed genes for categorising them in different functional and pathways groups

Classification of differentially expressed genes based on functional categories and pathways was executed by DAVID biological interpretation tool (http://david.abcc.ncifcrf.gov/)^18^. These differentially regulated transcripts of stress treated *ein*2 and *aba*1.6 were associated with about significant 100 metabolic and biosynthetic pathways (Supplementary Tables S4, S5, S6 and S7) amongst which phenylpropanoid, plant hormone, terpenoid, steroid and flavonoid biosynthetic pathways, methane, glycolipid, ascorbate and alderate metabolism pathways were found down-regulated in both stress treated mutant in comparison to wild type.

Gene ontology analysis and term enrichment for various biological process, molecular function and cellular component were also performed by DAVID. Among functional annotation categories of differentially expressed genes, common significantly enriched categories of down-regulated genes in combined stress treated *ein*2 and *aba*1.6 were response to stress, response to abiotic stimulus, response to reactive oxygen species (ROS), response to oxidative stress etc (Fig. 1C,D).

**Figure 2:**
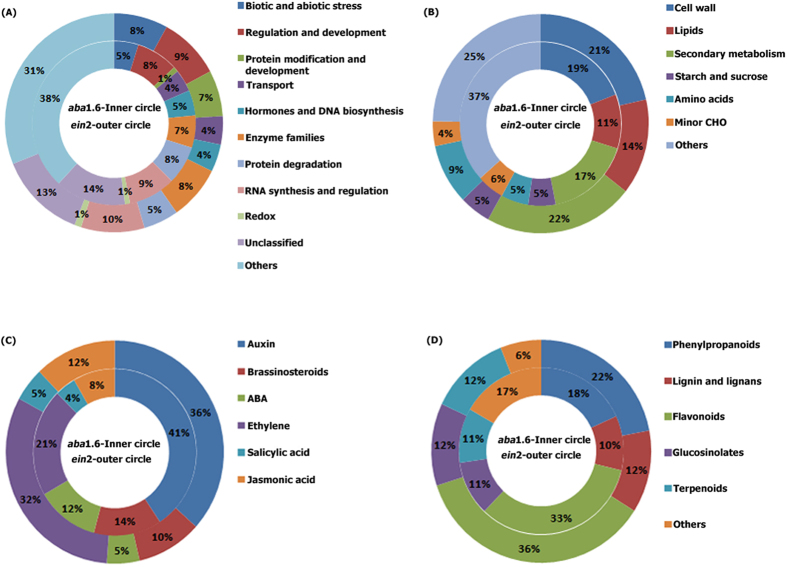
Categorization of differentially expressed genes in different functional groups. By using MAPMAN differentially expressed genes of combined stress treated *ein*2 and *aba*1.6 were categorised in (**A**) Biological function (**B**) cellular metabolic process (**C**) hormones and (**D**) secondary metabolites groups^58^.

Functional annotation of the identified differentially expressed genes in stress treated *ein*2 through MAPMAN revealed 13.00% of unclassified genes, 8.00% were related to abiotic and biotic stress, 9.20% were related to regulation and development, 6.81% were assigned to protein modification and degradation, 4.20% were related to transport, 4.08% were linked to hormones and DNA synthesis, 8.01% were associated to enzyme families, 5.20% were assigned to protein degradation and 9.37% were related to RNA synthesis and regulation. In the same way functional annotation of identified genes in *aba*1.6 revealed 14.00% of unclassified genes, 4.80%, 6.38%, 1.03%, 4.35%, 7.64%, 8.28%, 9.37% and 5.00% of genes were related to biotic and abiotic stress, enzymes, redox, transport, protein modification and degradation, regulation and development, RNA synthesis and regulation, hormones and DNA synthesis respectively (Fig. 2A). Differentially expressed genes associated with cellular metabolism category were categorized in cell wall, lipids, secondary metabolism, starch and sucrose, amino acids and minor CHO metabolism (Fig. 2B). The differentially expressed hormone related genes in *ein*2 and *aba*1.6 were further categorized in auxin, brassinosteroids, ABA, ethylene, salicylic acid and jasmonic acid (Fig. 2C). Genes linked to secondary metabolite category were classified in phenylpropanoids, lignin, flavonoids, glucosinolates and terpenoids (Fig. 2D).

### Common transcription factors affected in stress treated mutants

**Figure 3:**
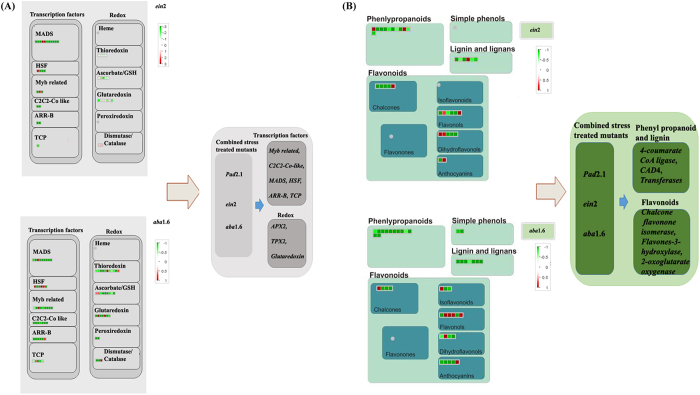
MAPMAN analysis of differentially expressed (**A**) transcription factors and redox related genes (**B**) secondary metabolites biosynthesis and metabolism related genes in combined stress treated *ein*2 and *aba*1.6^58^ (left panel). Common down-regulated genes related to the above mentioned group in combined stress treated *ein*2, *aba*1.6, and *pad*2.1^17^ (right panel).

MAPMAN analysis of differentially expressed genes also revealed about various differentially regulated transcription factors in response to combined stress treatment in *ein*2 and *aba*1.6 amongst which *MYB* related, *C2C2-CO* like, *MADS* (MADS box transcription factor family), *HSF* (Heat shock factors) *ARR-B* and *TCP* were found down-regulated in all the three mutant including *pad*2.1 (Fig. 3A).

### Redox related genes affected in stress treated mutant

Among the cellular redox related pathways thioredoxin, ascorbate/glutathione cycle and glutaredoxin were affected in *pad*2.1, *ein*2 and *aba*1.6 in response to stress treatment. The genes related to ascorbate/glutathione cycle like *APX*2 and *inositol monophosphatase*, *glutaredoxin* and *thioredoxin like TPX*2 and *cis-his rich thioredoxin* were found down-regulated in all the three stress treated mutants (Fig. 3A).

### Common secondary metabolism pathways affected in stress treated mutants

In response to combined stress stimulus phenylpropanoid, lignin and lignan, flavonoids, glucosinolates and terpenoids metabolism and biosynthetic pathways were affected in all the three mutants. The genes concerned with phenylpropanoid and lignin biosynthesis like 4-*coumarate CoA ligase*, *CAD*4 and *transferases* were found down-regulated in stress treated *pad*2.1, *ein*2 and *aba*1.6 (Fig. 3B). In flavonoid biosynthetic pathway *chalcone flavonone isomerase*, *flavones-3-hydroxylase*, *UDP-glycosyl transferase* and *2-oxoglutarate oxygenase* were found down-regulated in all the three stress treated mutants. On the other hand, genes related to glucosinolates biosynthesis and metabolism was found up-regulated in both stress treated *pad*2.1 and *aba*1.6. The majority of genes associated with terpenoid biosynthesis and metabolism were found down-regulated in stress treated *ein*2 and *aba*1.6.

### Affected common stress linked genes in stress treated mutants

**Figure 4:**
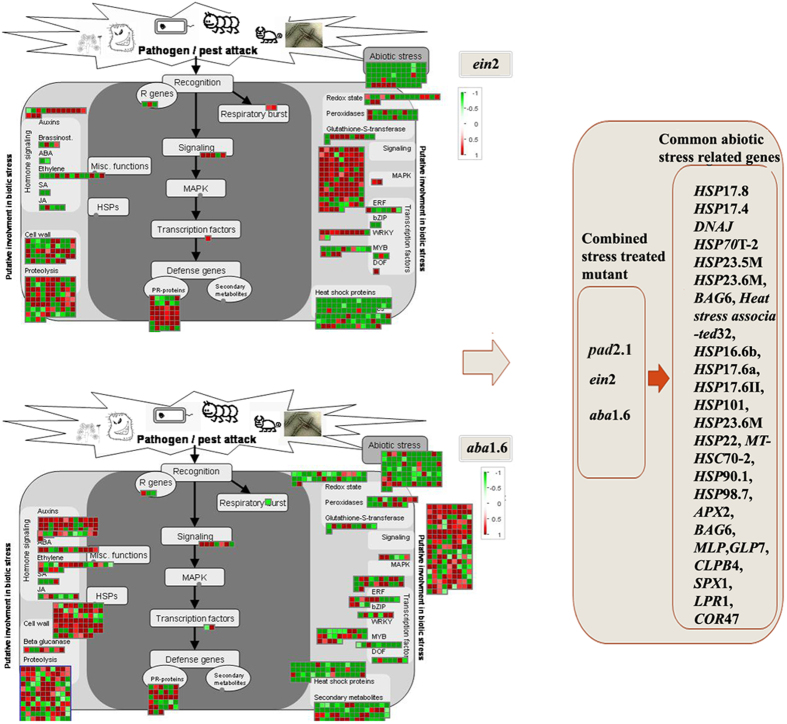
Analysis of differentially expressed stress related genes in combined stress treated *ein*2 and *aba* 1.6 by using MAPMAN^58^ (left panel). Common down-regulated stress related genes in combined stress treated *ein*2, *aba*1.6 and *pad*2.1^17^ (right panel).

The majority of genes related to abiotic stress were found down-regulated in all the three stress treated mutants. Abiotic stress related genes like most the *heat shock* family proteins, *cold regulated proteins (COR*47), *SPX domain containing proteins (SPX*1), low phosphate root proteins (*LPR*1), m*ajor latex proteins (MLP*) and *BCL-2 associated anthogene* were found down-regulated in all the three stress treated mutants (Fig. 4). *Low temperature induced protein (LTI*65) was found up-regulated in stress treated mutant.

Biotic stress related genes like *NBS-LRR class disease resistance proteins, germin like proteins* and *defensin* like proteins were also found down-regulated in stress treated mutants.

### Protein isolation and comparative proteomic analysis

**Figure 5:**
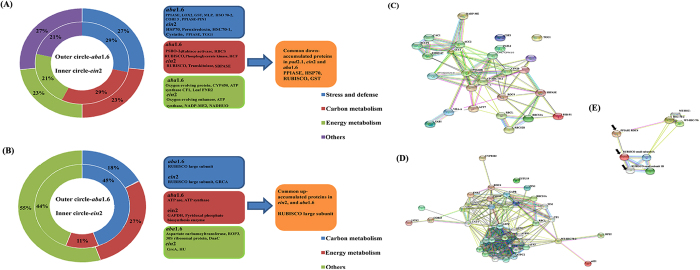
Functional categorization of differentially accumulated proteins and their possible interaction analysis. (**A**) Down-accumulated and (**B**) up-accumulated proteins in combined stress treated *ein*2 and *aba*1.6 (left panel). Common down – and up-accumulated proteins in *ein*2, *aba*1.6 and *pad*2.1^17^. STRING software protein interaction analysis of down-accumulated proteins in combined stress treated (**C**) *ein*2 and (**D**) *aba*1.6^19^. Panel E showed the interaction between common down-accumulated proteins in combined stress treated *ein*2, *aba*1.6 and *pad*2.1^17^.

**Figure 6:**
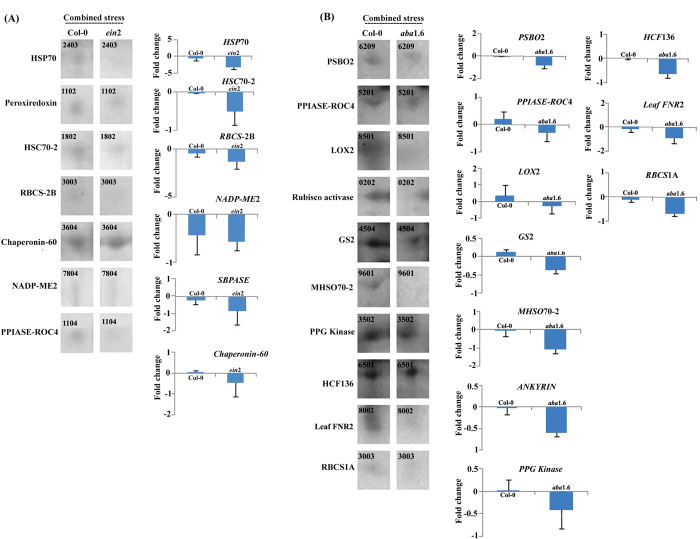
Validation of some differentially expressed genes by using comparative proteomics. (**A**) Combined stress treated Col-0 vs *ein*2 (**B**) Combined stress treated Col-0 vs *aba*1.6. Data are presented as mean ± SE (N = 3). Protein spots were cropped from Supplementary Figure S1 and the second dimension gels were run by using 12% SDS polyacrylamide gels under similar experimental condition at 22 °C. Relative gene expression level between combined stress treated Col-0 vs *ein*2 and combined stress Col-0 vs *aba*1.6 were calculated by Log_2_ fold change. Data are presented as mean ± SE (N = 3).

From the protein isolated from the combined stress treated *ein*2 and *aba*1. 6 comparative proteomics were performed. The identified differentially accumulated protein spots (Supplementary Fig. S1) were further recognized by MALDI TOF MSMS. Approximately 319 and 299 spots were identified in stress treated Col-0 and *ein*2. Between stress treated Col-0 and *ein*2, 120 spots were similar to the overall coefficient of variation being 38.84. Out of 37 differentially accumulated proteins, 9 and 28 of them were found as up- and down-accumulated respectively in stress treated *ein*2 (Supplementary Table S8). Amongst the down-accumulated proteins 29% of them like HSP70, cold shock transcription regulator CspA, peroxiredoxin, peptidyl prolyl cis-trans isomerase ROC4, TGG1 (Thioglucoside glucohydrolase 1) etc. were related to stress and defence category, whereas 29.00% and 21.00% of them were related to carbon and energy metabolism respectively (Fig. 5A).Genes like *HSP*70, and *PPIASE*, *seduheptulose bis-phosphatase (SPBASE*), *chaperonin*-60 and *malic* enzyme were also found down-regulated in the transcriptome study of stress treated *ein*2 (Fig. 6A). Among the up-accumulated proteins majority of the proteins were related to carbon and energy metabolism (Fig. 5B).

About 292 and 280 differentially accumulated protein spots were revealed in stress treated Col-0 and *aba*1.6 respectively. Near about 61 spots showed similarity with overall coefficient of variation being 50.06 between stress treated Col-0 and *aba*1.6. About 42 differentially accumulated proteins were identified by MALDI TOF MSMS. Out of which 11 and 26 proteins were found up- and down-accumulated respectively (Supplementary Table S9). Among down-accumulated proteins in stress treated *aba*1.6, 27.00%, 23.00%, 23.00% and 27.00% were related to stress and defence, energy metabolism, carbon metabolism and others categories respectively (Fig. 5A). Stress and defence related proteins like HSP70 family protein, PPIASE, glutathione transferase 8, lipoxygenase 2 (LOX2), major latex protein (MLP), jasmonic acid responsive protein (CORI3) etc. were found down-accumulated in stress treated *aba*1.6. Genes like *HSP70 family proteins*, *PPIASE, HCF136* etc. were also found down-regulated in stress treated *aba*1.6 (Fig. 6B).

HSPs, PPIASE and RUBISCO small subunits were found down-regulated in both gene and protein expression levels in combined stress treated *pad*2.1, *ein*2 and *aba*1.6 (Figs 5A and 6).

### Analysis differentially accumulated proteins of *ein*2 and *aba*1.6 by STRING 10 software

By using STRING 10 software^19^ interaction between differentially accumulated proteins has been revealed in combined stress treated *ein*2 and *aba*1.6 (Fig. 5C,D). Proteins like HSPs, PPIASE ROC4 and RUBISCO small subunit were found down-accumulated in all the three stress treated mutants at both gene and protein expression level showed more chance to interact with each other (Fig. 5E).

### Validation of transcriptome by quantitative RT-PCR

The result of microarray experiment was further validated by checking the expression of stress and defence related genes like *HSP17.6, HSP90.1, HSP70B, HSP70, CAD4, MYB108, AIG, BCL2* and *MRH6*, *GSH and redox related genes like GSTF8, GSTU14, GSTU23, APX2, TPX2, Monophosphatase*, ABA biosynthesis and responsive genes like *NCED9, CIPK1, LHY, CCA1* and ethylene responsive genes like *LHY, AP2, SHN2* and *ERF* in the stress treated Col-0, *ein*2 and *aba*1.6. All these genes were found down-regulated in stress treated *ein*2 and *aba*1.6 in comparison to stress treated Col-0 (Supplementary Fig. S2).

### Quantification of GSH

**Figure 7:**
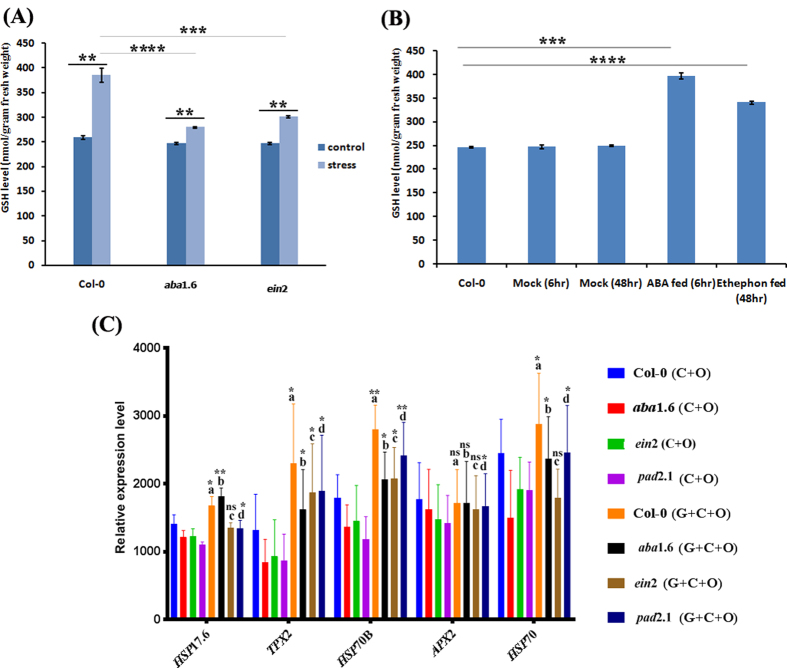
Combined stress treatment and GSH feeding effect. Combined stress treatment effect on (**A**) reduced GSH content in Col-0, *ein*2 and *aba*1.6. (**B**) Effect of ABA and ethephon treatment on reduced GSH accumulation in Col-0. (**C**) Combined stress and GSH feeding effect on the expression of selected genes from microarray experiment in *ein*2, *aba*1.6 and *pad*2.1 by using semi-quantitative RT-PCR. C + O = Cold + osmotic stress, C + O + G = Cold + osmotic stress + GSH. Data are presented as mean ± SE (N = 3). Significant difference between Col-0 (C + O) vs Col-0 (C + O + G), *aba*1.6 (C + O) vs *aba*1.6 (C + O + G), *ein*2 (C + O) vs *ein*2 (C + O + G) and *pad*2.1 (C + O) vs *pad*2.1 (C + O + G) are depicted by a, b, c and d respectively. *p < 0.05, **p < 0.01, ***p < 0.001 and ****p < 0.0001.

GSH was estimated from 3 weeks old Col-0, *ein*2 and *aba*1.6 plants in control (untreated) and combined stress treated condition. In control condition almost similar content of GSH was estimated in Col-0 and mutants but in response to stress condition about 1.30 and 1.40 fold more GSH was estimated in Col-0 than *ein*2 and *aba*1.6 respectively (Fig. 7A). Elevated GSH level was observed in both ABA and ethephon treated Col-0 in comparison to untreated Col-0 (Fig. 7B)

### Gene expression study after GSH feeding in stress treated mutants

After GSH feeding, all the selected stress marker genes like *HSP*70B, *HSP*17.6, *HSP*70, *TPX*2 and *APX*2 showed higher expression in combined stress treated Col-0 in comparison to the mutants (Fig. 7C).

### γ-*ECS* expression study after ABA and ethephon treatment in Col-0

**Figure 8:**
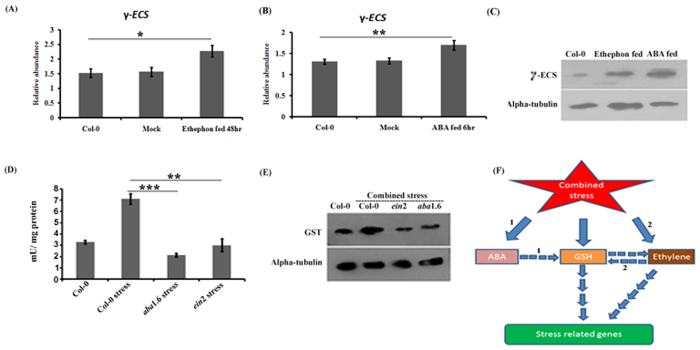
Effect of ABA and ethephon feeding in Col-0 on the expression of *γ-ECS* and accumulation of GSH with the investigation of GST accumulation and activity in combined stress treated Col-0 and mutants. Effect of (**A**) ethephon and (**B**) ABA feeding on the *γ-ECS* transcripts. (**C**) Effect of ABA and ethephon feeding in Col-0 on γ-ECS protein accumulation. (**D**) GST activities of per mg protein isolated from Col-0, combined stress treated Col-0, *ein*2 and *aba*1.6. This result has been taken from Supplementary Figure S4 (**E**) GST protein expression in Col-0, combined stress treated Col-0, *ein*2 and *aba*1.6. Protein bands were cropped from Supplementary Figure S3. Data are presented as mean mean ± SE (N = 3). Significant difference in γ-*ECS* expression and GST activity between Col-0 vs ethaphon and ABA treated Col-0, combined stress treated Col-0 vs combined stress treated mutants depicted by *p < 0.05, **p < 0.01, ***p < 0.001. (**F**) Proposed model for the role of ethylene and ABA in regulating stress related genes in response to combined stress. (1) Combined stress activates ABA biosynthesis which is supposed to induce the expression of various stress related genes via inducing GSH synthesis^13^. (2) In response to combined stress, ethylene induces various stress related genes by cross talking with GSH biosynthetic pathway^15^.

The elevated expression of γ-ECS was noticed in Col-0 at both gene and protein level in response to 6 hr of ABA and 48 hr of ethephon treatment in comparison to untreated Col-0 (Fig. 8A–C).

### GST expression and activity

GST activity was also found maximum in per mg of total protein extracted from combined stress treated Col-0 in comparison to combined stress treated *ein*2 and *aba*1.6 (Fig. 8D). Maximum GST protein expression was found in combined stress treated Col-0 followed by untreated Col-0 and combined stress treated mutants (Fig. 8E).

## Discussion

A large number of genes were found differentially expressed in response to stress treatment in *ein*2 and *aba*1.6. Microarray experiment data also revealed the down-regulation of various stress responsive genes in stress treated *ein*2 and *aba*1.6 which were previously found up-regulated in response to stress in Col-0. Interestingly, majority of stress and defence related genes were commonly found down-regulated in combined stress treated *pad*2.1, *ein*2 and *aba*1.6 in comparison to stress treated Col-0. There can be two possibilities which are supposed to be the reasons of the above mentioned pattern of differential expression of genes. First, a possible reason for differential expression of genes may be the comparatively higher glutathione (GSH), ethylene and abscisic acid (ABA) content can have the direct or indirect role in the induction of these genes and protein expression in response to stress conditions in the plants. Second, this investigation also proposed the role of ethylene and ABA in regulating the expression of various stress and defence related genes via inducing the GSH under stress conditions (Fig. 8F).

### Effect on the expression of transcription factors

Up-regulation of various *MYB* related transcription factors and proteins were reported in abiotic stress treated *A. thaliana*^20^,^21^ Role of ABA in inducing genes encoding *MYB* related proteins has also been reported^22^. Elicited level of GSH also induced the expression of *MYB* related TFs in *Lotus*^23^. Therefore, less content of GSH and ABA in stress treated *pad*2.1 and *aba*1.6 respectively, may be the reason for the down-regulation of *MYB* related TFs. In response to ABA treatment in *Salix sukhowensis HSF* was found up-regulated. Less activation of *HSF*1 in response to depleted GSH has already been reported^24^. So, the less GSH and ABA content in stress treated *pad*2.1 and *aba*1.6 respectively, were supposed to be the main cause of down-regulation of *HSFs*. Ethylene has the role in inducing GSH biosynthesis in response to abiotic stress condition in *A. thaliana*^15^. *MADs bo*x TFs have the role in the floral organ specification, plant growth and development. These TFs were also found up-regulated against cold and dehydration stress^25^. *MADs box* genes were also found up-regulated in response to ABA treatment in barley^26^. These TFs were also found down-regulated in combined stress treated *pad*2.1 which indicated the role of GSH in their induction^17^.

Ethylene has the role in inducing GSH biosynthesis in response to abiotic stress condition in A. *thaliana*^15^. Inhibited ethylene signaling in *ein*2 may be the possible reason for less induction of GSH, which might lead to the down-regulation of *MYB related* TFs, *HSFs* and *MADs box TFs* in *ein*2 in response to stress treatment. ABA also increased the content of GSH in the leaves of maize seedlings^27^. Therefore, the other reason for the down-regulation of transcription factors in stress treated *ein*2 and *aba*1.6 may the less content of GSH in stress treated mutants in comparison to Col-0 (Fig. 7A). Down-regulation of *C2C2-Co* like and *ARR-B* transcription factors in all the three stress treated mutant suggests the importance of GSH, ethylene and ABA for their activation in response to stress condition.

### Effect on the stress responsive genes and proteins

In the present study most of the abiotic stress responsive genes like majority of HSPs family genes like *HSP17.4, HSP17.6, HSP17.8, HSP23.5, HSP26.5, HSP70T-2, HSP90.1, HSP98.7, HSP101* etc, *COR47, SPX1, LPR1, MLP* and *BCL-2* associated anthogene (BAG) were found down-regulated in *ein*2 and *aba*1.6. The comparative proteomics experiment also revealed the down-accumulation of HSPs proteins in all the three stress treated mutants. In the previous study, members of HSPs were found strongly induced in response to low temperature and drought stress^28^,^29^. The role of GSH oxidation on the induction of *HSPs* has been reported earlier^30^. The same members of HSPs were also found down-regulated in combined stress treated GSH mutant *pad*2.1 and up-regulated in GSH fed Col-0^17^,^31^. Estimated less content of GSH in stress treated *ein*2 and *aba*1.6 in comparison to stress treated Col-0 (Fig. 7A) validated the previous studies about the role of ABA and ethylene in inducing GSH and also indicated the role of GSH in inducing these HSPs genes in *ein*2 and *aba*1.6.

Abiotic stress related gene like *COR*47 was found up-accumulated in cold and drought stress treated *A. thaliana*^32^*. COR47* was also found significantly up-regulated in GSH treated *A. thaliana*^33^. ABA depleted mutant *aba*1.6 showed reduced expression of COR in comparison to the wild type in response to mannitol^34^. The role of *ERF1* in inducing *COR47* is an established fact^35^. Since *ein*2 is an ethylene insensitive mutant. Therefore *COR47* was found down-regulated in stress treated *ein*2.

Recent investigations showed that several proteins carrying *SPX* domain were essential for phosphate homeostasis in plants^36^. Phosphate deficiency caused oxidative stress condition in plants which ultimately induced GSH^37^. Therefore GSH might have some role in inducing *SPX*1 as it was found down-regulated in stress treated *pad*2.1^17^. As it has been already mentioned above that both ethylene and ABA have a role in inducing GSH. Corroborating the evidence *SPX*1 was also found down-regulated in stress treated *ein*2 and *aba*1.6.

The role of MLP in providing drought stress tolerance in *Arabidopsis* has been recently established^38^. MLPs were negatively regulated in response to ABA and ethylene treatment in plants^39^,^40^. Positive regulation of *MLP* in response to stress treatment in *pad*2.1, *ein*2 and *aba*1.6 also indicated the role of GSH in inducing *MLP* in response to stress conditions.

*The BCL2 associated antigen (BAG*) proteins can interact with different proteins to regulate the apoptosis like processes ranging from abiotic stresses to pathogen attacks^41^. Down-regulation of *BAG* in all the three stress treated mutant also suggested the importance of GSH, ethylene and ABA on its expression in stress condition.

Germin like protein (GLP) generally has the oxalate oxidase activity which break downs the oxalate in CO_2_ and H_2_O_2_^42^. These proteins also found up-accumulated in salt stress treated barley^43^ and its expression was also modulated by ABA^44^. *GLP*7 was also induced by ethylene^45^. Down-regulation of *GLP*7 of stress treated mutants revealed the importance of both ABA and ethylene on the induction of *GLP* gene against stress condition. Defensin like proteins plays a major role in combating biotic stress condition^46^. Previous report suggested that ethylene response pathways were essential for the induction of plant defensin gene^47^. Less expression of *defensin like gene* in *ein*2 strengthened the view about the role of ethylene in inducing this gene. Role of GSH in inducing this defensin gene has already been suggested^17^. Since ABA has the role in inducing GSH. Therefore, less induction of GSH in stress treated *aba*1.6 supposed to be the main reason for the down-regulation of defensin gene.

### Effect on cellular redox related genes and proteins

Redox related genes like *glutaredoxin, GSTs*, *TPX*2, *APX*2 and *PPIase ROC*4 were found down-regulated in combined stress treated *ein*2 and *aba*1.6. Glutaredoxins have peroxidase activity and its contribution in providing resistance against oxidative stress is an established fact^47^,^48^. Glutaredoxin like proteins were found up-regulated in GSH fed Col-0^31^. In combined stress treated *pad*2.1 glutaredoxin like proteins were also found down-regulated in comparison to stress treated Col-0^17^. As mentioned earlier about the role of both ABA and ethylene in inducing GSH in plants, down-regulation of glutaredoxin may be the consequence of the less content of GSH in stress treated *ein*2 and *aba*1.6 in comparison to stress treated Col-0. In previous studies, GST transcripts have been found up-regulated in response to exogenous application of GSH, ABA and ethylene in plant tissue^31^,^49^,^50^ and their results indicated towards the important role of GSH, ABA and ethylene in inducing *GST*s. The total *APX* activity was highly induced in response to *in vitro* applied H_2_O_2_ or ethylene and ABA^51^,^52^. Less induction of GSH, ABA and ethylene signaling pathways were supposed to be the possible reason for the down-regulation of *GST* and *APX*2 in all the three stress treated mutants. Some *GST* homologs like *GSTU*10*, GSTF*5*, GSTZ*2 and *GSTF*11 were found up-regulated in combined stress treated mutants viz. *ein*2. *aba*1.6 and *pad*2.1 suggested that these work independent of ethylene, ABA and GSH crosstalk but their induction is stress dependent. In previous reports *TPX* was reported sensitive to hyperoxidation under oxidative stress and inactivated TPX activity was important for reducing thioredoxin to other substrate in response to the acute oxidative stress^53^. *TPX*2 was also down-regulated in stress treated *pad*2.1^17^. In stress treated *ein*2 and *aba*1.6 due to less GSH content there were probability of more ROS production. This excessive ROS production might be the reason of the down-regulation of *TPX*2 in both mutants in response to the stress condition. Previous study also suggested about the role of thiol-disulfide exchange in the regulation of *PPIase ROC4 (CYP*20-3) and its contribution in protein folding and assembly in response to light and redox changes^54^. *PPIASE ROC*4 was found down-regulated in stress treated *pad2.1* in both gene and protein level^17^. So, down-regulated *PPIase ROC4* at both gene and protein level in stress treated *ein*2 and *aba*1.6 suggested about the role of GSH and ABA in inducing this gene and protein in response to stress conditions.

### Effect on secondary metabolism pathways

Sinapyl alcohol, coniferyl alcohol and coumeryl alcohol are examples of phenylpropanoids which act as important precursors of lignin biosynthesis. Lignin also played an important role in defence response in plants to stress^55^. Since Lignin and flavonoid biosynthesis were induced in response to GSH treatment in bean cell culture and *A. thaliana* and also negatively regulated in response to abiotic stress treatment in *pad*2.1^17^,^31^,^56^. Therefore, measured less content of GSH in both stress treated *ein*2 and *aba*1.6 in comparison to stress treated Col-0 might be the reason for negative regulation of phenylpropanoid, lignin and flavonoid biosynthetic pathway in these mutants. Role of drought stress in inducing the accumulation of terpenoids in Scots pine seedlings has already been studied. Down-regulation of genes related to terpenoid biosynthesis in stress treated *ein*2 and *aba*1.6 suggested the direct or indirect role of ethylene and ABA in inducing this pathway in response to stress^57^.

### Protein interaction analysis by STRING 10 software

Interactions between common down-accumulated proteins in combined stress treated mutants suggested the important role of GSH, ethylene and ABA in inducing these proteins against stress condition which might help in combating stress condition by their interaction (Fig. 5E).

In conclusion, our study suggests a crosstalk between ABA, ethylene and GSH under stress condition as determined by performing a comprehensive transcriptomic and proteomic analysis of *A. thaliana* mutant *ein*2 and *aba*1.6 in response to combined cold and osmotic stress. The down-regulation of common genes in all the three stress treated mutants suggested the role of ABA and ethylene in inducing stress related genes by activating GSH pathway.

## Methods

### *Arabidopsis* seed germination and combined stress treatment

The seeds of *Arabidopsis* were procured from Nottingham *Arabidopsis* Stock Centre [NASC – Col-0 (N1092), *ein*2 (N8844) and *aba*1.6 (N3772)]. The surface sterilized seeds were grown in Murashige and Skoog (MS) medium and maintained in a growth chamber at 22 °C under a 16 h light/8 h dark cycles. Three weeks old seedlings were further placed in filter paper for 5 min at 4 °C for early dehydration with cold stress and then inoculated in liquid half strength MS medium with 30% PEG at 4 °C for an additional 6 hours for combined cold and osmotic stress treatment. The leaves of stress treated plants were collected for RNA and protein isolation. Morphological changes in response to combined stress treatment in Col-0, *ein*2 and *aba*1.6 were also observed.

### RNA extraction and Microarray analysis

300 mg of leaf tissue was utilized for total RNA extraction using the RNeasy Plant Minikit (Qiagen’s RNeasy Minikit) and eluted in RNAse-free water for each microarray experiment. Each experiment consisted of combined stress treated Col-0, *ein*2 and *aba*1–6 samples and was conducted in replicates of three. For quantitative RT-PCR total RNA was extracted from leaf samples by TriZol method. RNA quality and quantity were ensured with a NanoDrop ND-1000 spectrophotometer and an Agilent 2100 Bioanalyzer. According to the protocols outlined in the GeneChip Expression Analysis Technical Manual all the samples were prepared. Hybridizations to the Agilent custom *Arabidopsis* 8 × 60 k microarray were designed by Genotypic technology private limited (AMADID: 48015), Bangalore, altogether the transcriptome of the combined stress treated Col-0, *ein*2 and *aba*1.6 were compared. Microarray data analysis and normalization have been performed using GeneSpring GX version 12.0 and Microsoft Excel. The transcriptomics data were deposited at Gene Expression Omnibus (GEO) at the National Centre for Biotechnology Information (NCBI, http://www.ncbi.nlm.nih.gov/geo/) with GSE77490 accession number.

### Functional enrichment and annotation

By using Database for Annotation, Visualization and Integrated Discovery (DAVID) v6 differentially expressed genes were annotated in different functional categories and then their functional enrichment analysed. *P* value of 0.05 was used for enriched pathways and gene ontology function.

### Assignment of the differentially regulated genes to functional pathways by MAPMAN and DAVID

Differentially expressed genes recognized by the microarray experiment were also analysed to categorize in different functional pathways by MAPMAN software (http://gabi.rzpd.de/projects/MAPMAN, version 3.5.1)^58^.

### Quantitative RT-PCR analysis

Quantitative RT-PCR was performed from the total RNA of both control and combined stress treated Col-0, *ein*2 and *aba*1.6. By using RevertAid first strand cDNA synthesis kit (Fermentas) using oligo (dT) as primer 1 μg of total RNA of each sample was reverse-transcribed. The quantitative RT-PCR was executed by using the Roche LightCycler 96 System (Roche) with FastStart Essential DNA Green Master (Roche). Selected genes for which quantitative RT-PCR was performed presented in Supplementary Table S10. Amplification was carried out for 40 cycles at 94 °C, 30 sec and 60 °C, 2:30 min with a preceding initial denaturation of 30 sec at 95 °C. *Actin* was used as the reference gene.

### Protein isolation and 2-DE

About 2 gm of leaf tissue of combined stress treated Col-0, *ein*2 and *aba*1.6 was used for total protein extraction by phenol extraction method. The tissue was then finely ground in liquid nitrogen and suspended in extraction buffer (700 mM Sucrose, 500 mM Tris–HCl, pH7.5, 50 mM EDTA, 100 mM KCl, 2% (w/v) β-mercaptoethanol, 1 mM phenylmethylsulfonyl fluoride) and protein extraction was done following standard protocol. The isolated protein was further resuspended in IEF buffer consisting of 7 M urea, 2 M thiourea, 4% 3-[(3-cholamido propyl)-dimethylammonio]- 1-propane sulfonate (CHAPS), 20 mM DTT and 1% (w/v) Bio-Lyte (3/10) ampholyte (BioRad Laboratories, Hercules, CA, USA) as standardized before. Bradford’s method was used for the measurement of protein concentration. 800 μg of protein was passively re-hydrated in immobilized pH gradient strip (11 cm; pH4–7; BioRad) for 12 h. IEF was performed as follows: 250 V for 30 min, 8000 V for 2 h, 8000 V for 26000 V-h, 750 V for 1 h on BioRad PROTEAN IEF Cell system (BioRad). Focused strips were further equilibrated in equilibration buffers I & II (BioRad) for 15 min each. Second dimension gels were run by using 12% SDS polyacrylamide gels and stained with colloidal Coomasie Brilliant Blue (CBB) G-250.

### Image analysis

The gel images were taken and analyzed by using a Versa doc image system (BioRad) and PD Quest software version 8.0.1 (BioRad) respectively. Spot detection was performed by matching the gels automatically and manual verification. Densities of spots were normalized against whole gel densities. The spots detected in at least two replicate gels were selected for annotation. Significant protein fold changes between the samples were statistically evaluated using the *t*-test function implemented in the software.

### In-gel digestion and mass spectrometric analysis

Differentially accumulated spots were excised manually from 2D gels and digested with trypsin (in-gel trypsin digestion kit, Pierce) following the manufacturer’s protocol. The desaltation and analysis of the samples were performed by using Zip-Tip μ-C18 (Millipore) and 4800 MALDI TOF/TOF analyzer (Applied Biosystems) respectively. The 0.5 μl dissolved sample in a solvent consisting of 0.1% trifluoroacetate and 50% acetonitrile (ACN) in MilliQ was mixed with 0.5 μl of matrix solution (1 mg/ml α-cyano-4-hydroxycinnamic acid dissolved in the aforementioned solvent), applied to a 384-MALDI sample target plate, and dried in air. After that peptides were evaporated with a ND:YAG laser at 355 nm. The acceleration of peptides was conducted with 25 kV injection pulse for time of flight analysis. Each spectrum was the cumulative average of 1000 laser shots. The MS/MS spectrum was collected in MS/MS 1 kV positive reflectron mode with fragments generated by post source decay (PSD). The mass tolerance of MS/MS was set to ±20 ppm. Following processing, 10 MS/MS precursors were selected (Minimum signal to noise ratio-50). The instrument was calibrated with the Applied Biosystems 4700 Proteomics Analyzer Calibration Mixture before each analysis. Data interpretation and automated database search were executed by using GPS Explorer Software (Applied Biosystems) and MASCOT program (Matrix Science Ltd) respectively. The interaction between identified differentially accumulated proteins in response combined stress were identified by STRING 10 software.

### Estimation of GSH

Extraction of GSH from leaves of Col-0 (control) and stress treated Col-0, *aba*1.6, *ein*2, ABA and ethephon fed Col-0 as well quantified by using Tsakraklides *et al.* method^59^. The experiments were conducted in three biological replicates.

### Feeding of GSH, ABA and ethephon and semi-quantitative RT-PCR

Both control and stress treated Col-0 and mutants were fed with GSH according to Sinha *et al.*^31^. Col-0 seedlings were also fed with 50 μM of ABA and ethephon for different time periods and after checking the expression of ABA and ethylene marker genes, 6 hr of ABA feeding and 48 hr of ethephon feeding were found to be optimum (data not given). For semi-quantitative RT-PCR total RNAs from both stress treated Col-0 and mutants, GSH fed plus combined stress treated Col-0 and mutants, ABA and ethephon treated leaf tissue were isolated using TriZol reagent (Invitrogen). About 1 μg of total RNA of each sample was reverse-transcribed by RevertAid first strand cDNA synthesis kit (Fermentas) using oligo (dT) as primer. The PCR was carried out using the following thermal cycling profile: 95 °C for 3 min, followed by required number of cycles (95 °C for 30 sec, 58 °C for 30 sec, and 72 °C for 45 sec). The sequences of the primer pairs listed in Supplementary Table S10. PCR products and their sizes were examined by using 1% agarose gel electrophoresis. *Actin* gene was amplified as an endogenous loading control for testing the validity of template preparation. Semi-quantitative RT-PCR products were quantified according to the relative abundance of the band by Quantity One software (BioRad).

### Immunoblotting and GST activity assay

Total protein was isolated from Col-0, ABA, ethephon and combined stress treated Col-0 and mutants according to the above mentioned method. The protein bands of γ-ECS and GST were identified by using a rabbit polyclonal anti γ-ECS and GST antibody as the primary antibodies and horseradish conjugated anti-rabbit IgG was used as secondary antibody (Agrisera). By using Pico chemiluminescent substrate (Pierce) immunoreactive proteins were visualized.

GST activity was measured both from Col-0 and mutants (viz. *ein*2, *aba*1.6) after ABA, ethephon, GSH treatment, and combined stress using the total protein through spectrophotometric assay with 1-chloro-2, 4-dinitrobenzene (CDNB) as a substrate^60^.

### Statistical analysis

All the experiments were conducted at least three biological replicates and the data presented as the mean ± standard error (SE) to compare the GSH content and relative gene expression profiles of Col-0, *ein*2 and *aba*1. 6 under control and stress conditions. One-way analysis of variance (ANOVA) followed by Student-Newman-Keuls multiple comparison test were used for statistical analysis (GraphPad InStat software, ver. 3.1). *P* < 0.05 or 0.01 or 0.001 was considered to be statistically significant.

## Additional Information

**How to cite this article**: Kumar, D. *et al.* Transcriptome analysis of *Arabidopsis* mutants suggests a crosstalk between ABA, ethylene and GSH against combined cold and osmotic stress. *Sci. Rep.* **6**, 36867; doi: 10.1038/srep36867 (2016).

**Publisher’s note:** Springer Nature remains neutral with regard to jurisdictional claims in published maps and institutional affiliations.

## Supplementary Material

Supplementary Information

Supplementary Dataset 1

Supplementary Dataset 2
