# Supplementary material for: Transcriptome analysis of Arabidopsis mutants suggests a crosstalk between ABA, ethylene and GSH against combined cold and osmotic stress
Source: Sci Rep. 2016 Nov 15;6:36867. doi: 10.1038/srep36867 (PMC5109278; doi:10.1038/srep36867)
Supplement: Supplementary Information [file srep36867-s1.pdf]

Transcriptome analysis of *Arabidopsis* mutants suggests a crosstalk between ABA, ethylene and GSH against combined cold and osmotic stress

Deepak Kumar, Saptarshi Hazra, Riddhi Datta and Sharmila Chattopadhyay*

Plant Biology Laboratory, Organic and Medicinal Chemistry Division, CSIR- Indian Institute of Chemical Biology, 4, Raja S.C. Mullick Road, Kolkata 700032, India

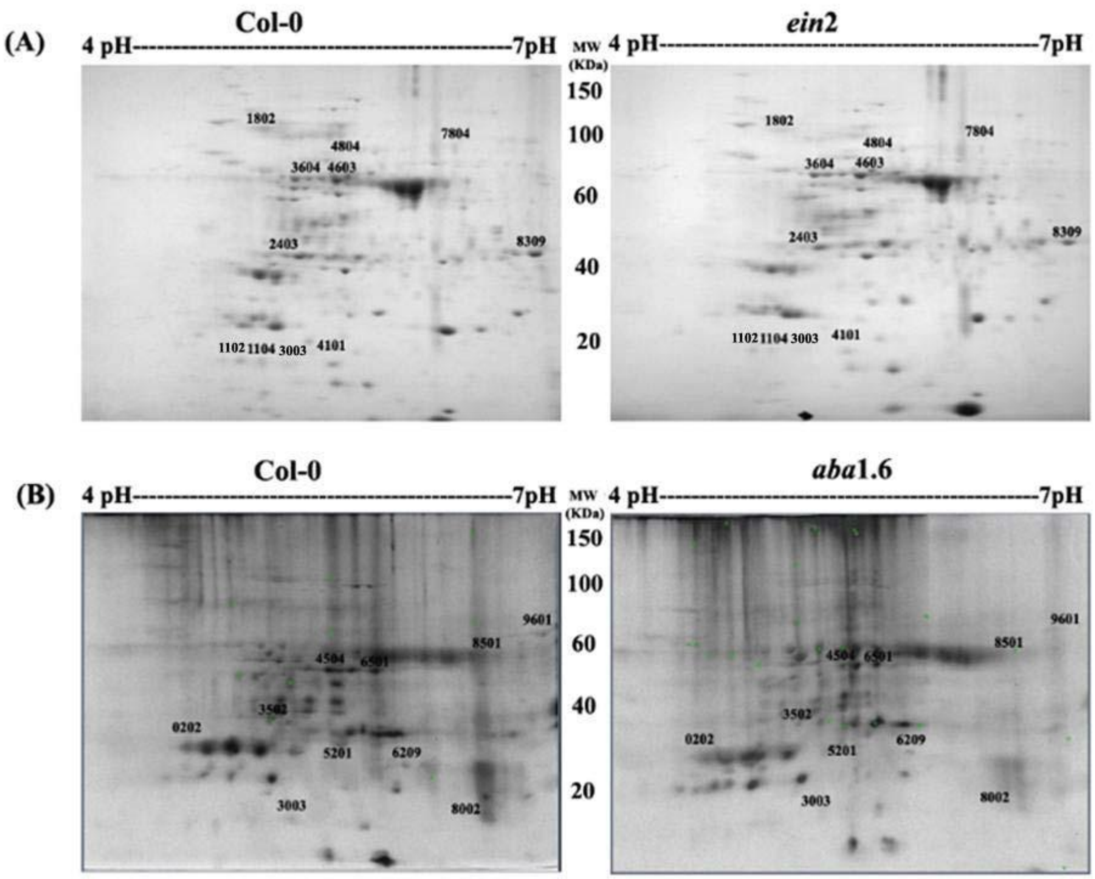

Supplementary Figure S1. 2D-PAGE gel picture with differentially accumulated protein spots in stress treated (A) *ein*2 and (B) *aba*1.6. All the second dimension gels were run by using 12% SDS polyacrylamide gels under similar control climate condition at 22^0C.

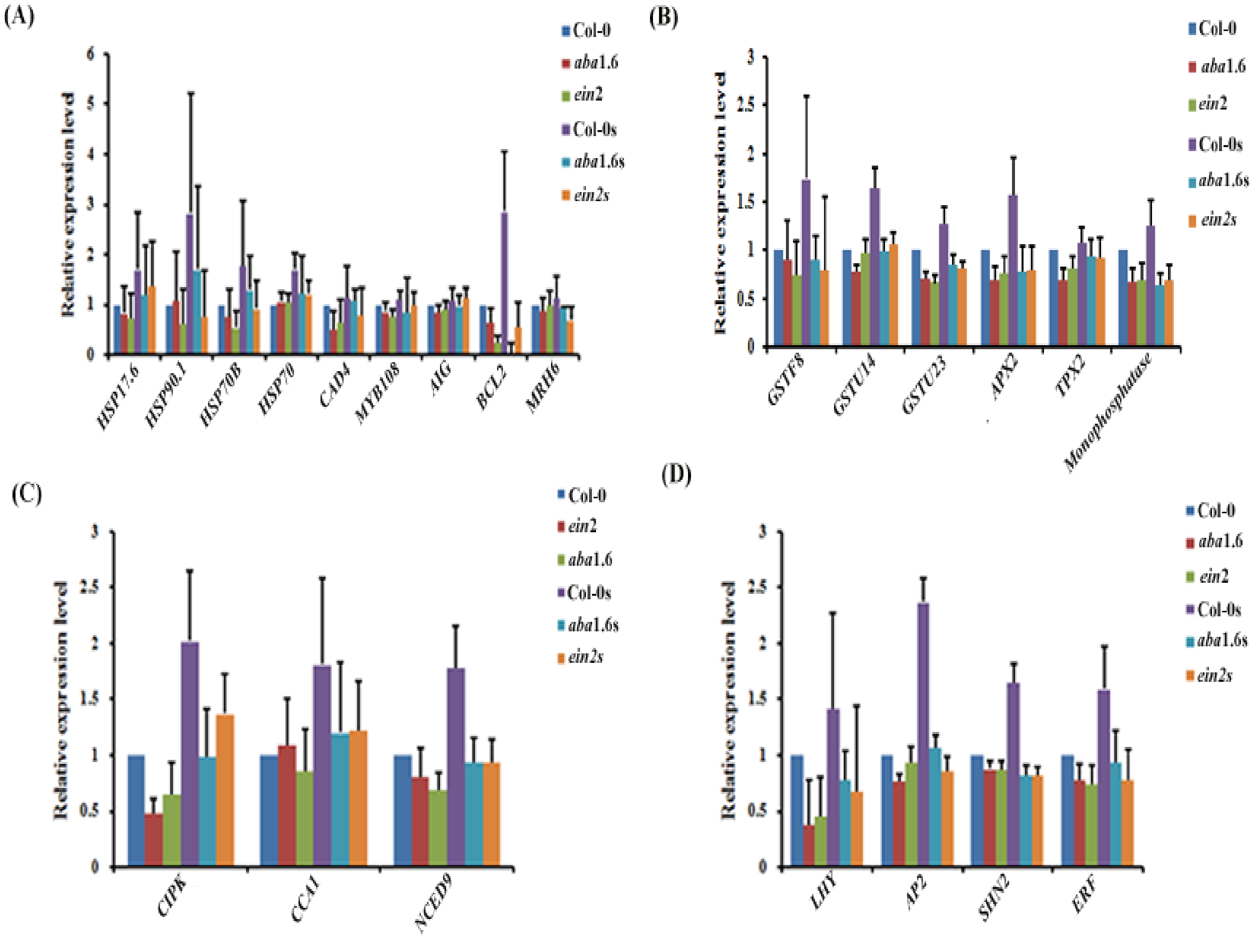

Supplementary Figure S2. Validation of differentially expressed genes in microarray experiment by using quantitative RT-PCR. (A) stress and defence related genes (B) GSH and redox related genes (C) ABA biosynthesis and responsive genes (D) ethylene responsive genes .Data are presented as mean±SE (N=3).Col-0s=Col-0+combined stress, *ein*2s=*ein*2+combined stress and *aba*1.6s=*aba*1.6+stress.

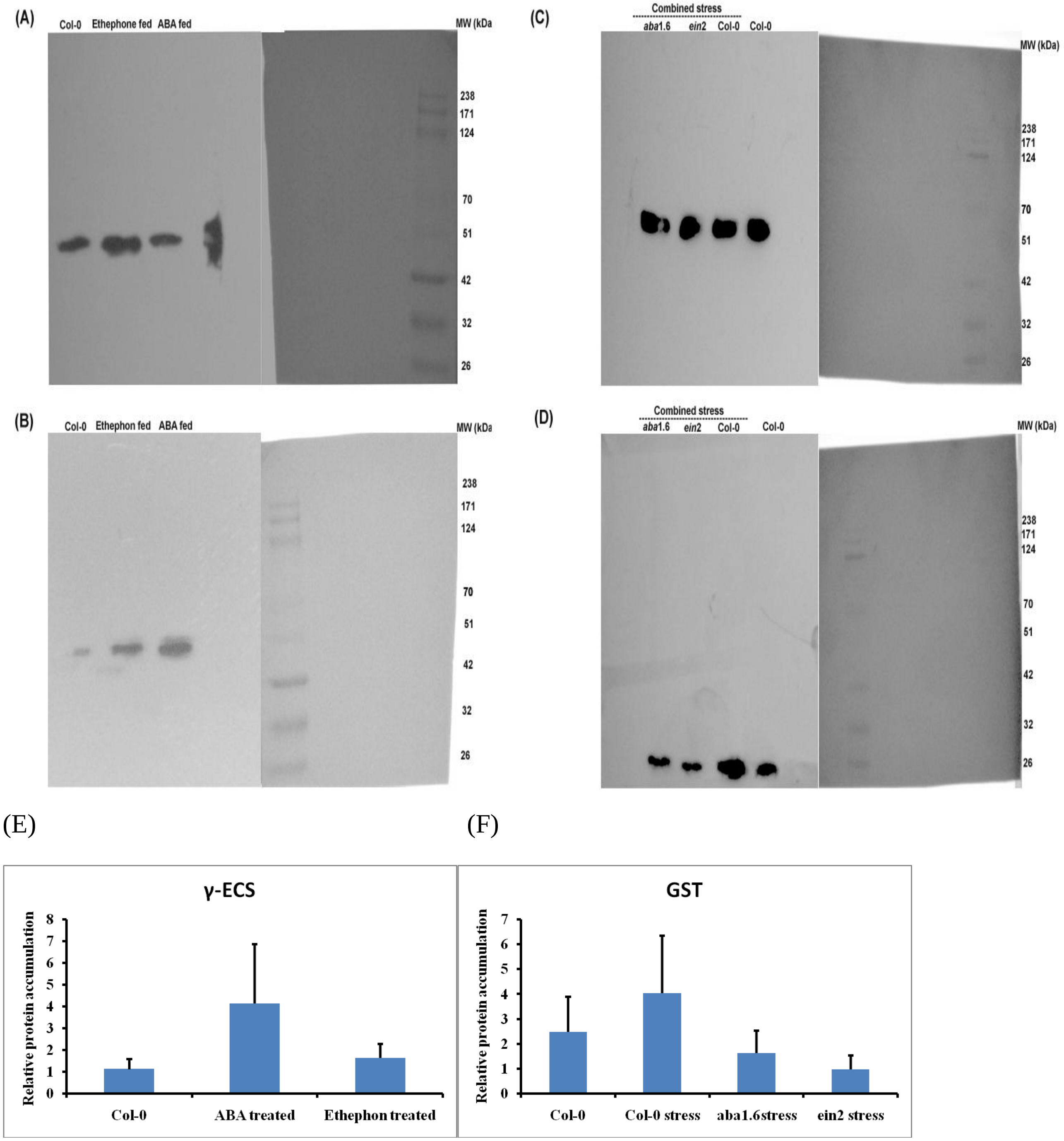

Supplementary Figure S3. Representative Immunoblot for (A) alpha-tubulin protein expression in Col-0, ethephon and ABA fed Col-0 (C) Col-0, combined stress treated Col-0, *aba*1.6 and *ein*2 (B) and (E) γ-ECS protein expression in Col-0, ethephon and ABA fed Col-0 (D) and (F)

GST protein expression in Col-0, combined stress treated Col-0, *aba*1.6 and *ein*2. Data are presented as mean±SE (N=3).

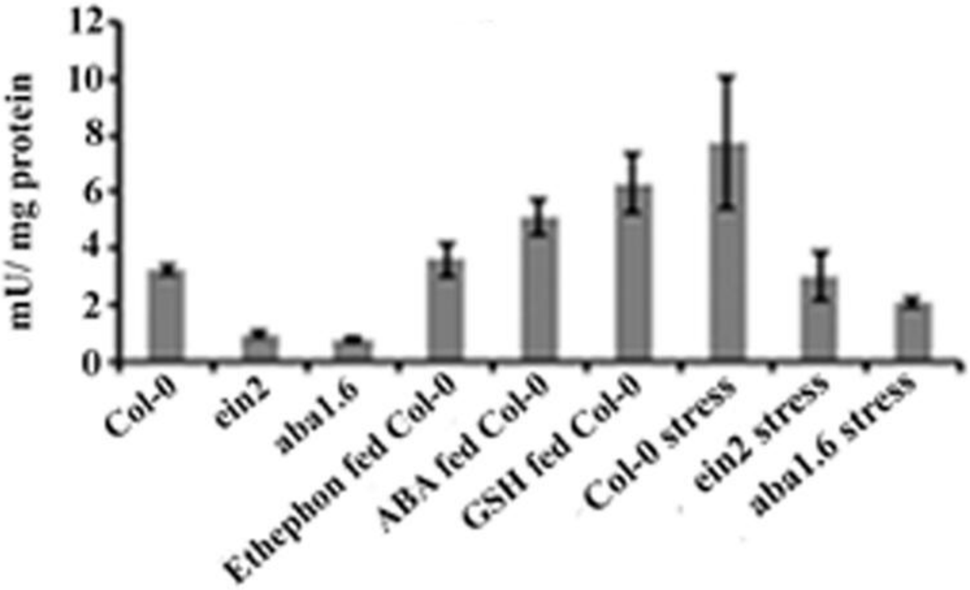

Supplementary Figure S4. GST activities of per mg protein isolated from Col-0, *ein*2, *aba*1.6, ethephon, ABA and GSH fed Col-0, combined stress treated Col-0, *ein*2 and *aba*1.6. Data are presented as mean±SE (N=3).

Supplementary Table S1. Differentially expressed genes in *ein* 2 in response to combined stress. Up-regulated genes in *ein* 2

ProbeName	GeneName	Stress treated *ein* 2 vs Col-0 (Fold change) GeoMean (3 replicate Log2 fold change)	P_Value
GT_A_84_P851276	GAMMA_CA3	1.21	0.0003
GT_At_Specific_00221437	AT4G22530.1	0.74	0.0005
GT_A_84_P830771	AT3G26770	0.84	0.0011
GT_A_84_P808847	WRKY15	0.80	0.0014
GT_A_84_P13955	ATGLR2.5	2.04	0.0021
GT_A_84_P854773	BE038580	1.28	0.0035
GT_A_84_P596791	TA46190_3702	0.81	0.0038
GT_A_84_P870191	AT1G21326	0.94	0.0039
GT_A_84_P595150	AT1G34070	2.28	0.0042
GT_At_Specific_00129617	AGP16	0.94	0.0045
GT_A_84_P788437	AT1G35210	1.39	0.0046
GT_A_84_P15781	CYP81D8	1.23	0.0054
GT_A_84_P834764	AT1G01440	0.68	0.0068
GT_A_84_P853383	AT1G70740	1.15	0.0079
GT_A_84_P866983	EDA39	0.95	0.0086
GT_A_84_P13481	WRKY15	0.64	0.0089
GT_A_84_P23148	PUB13	0.63	0.0090
GT_A_84_P274650	ATARFD1B	2.29	0.0090
GT_A_84_P562392	AT2G34355	1.15	0.0094
GT_A_84_P761741	AT3G46658	0.73	0.0094
GT_A_84_P841532	TA35514_3702	0.81	0.0094
GT_A_84_P861444	KNATM	2.54	0.0095
GT_A_84_P856901	AT5G16030	0.85	0.0105
GT_A_84_P833545	TA50784_3702	1.39	0.0107
GT_A_84_P15239	AT1G63740	0.80	0.0108
GT_A_84_P858974	PUB13	0.65	0.0109
GT_A_84_P533199	AT2G41835	0.65	0.0115
GT_A_84_P758138	AT2G33006	1.19	0.0121
GT_A_84_P551357	AT5G49390	0.76	0.0121
GT_A_84_P850199	TC392633	0.66	0.0122
GT_A_84_P171073	AT1G35210	1.16	0.0123
GT_A_84_P830800	AT5G58390	1.70	0.0125
GT_A_84_P60380	EDA39	0.92	0.0127
GT_A_84_P24086	AT3G44610	0.75	0.0132
GT_A_84_P808846	TA27747_3702	0.71	0.0136
GT_A_84_P777695	LSH4	1.12	0.0139
GT_A_84_P856245	AT1G16690	0.74	0.0140
GT_A_84_P856377	AT1G72900	0.99	0.0141
GT_A_84_P596516	AT4G14450	1.41	0.0147
GT_A_84_P19706	AT5G45000	2.55	0.0150
GT_A_84_P808837	WRKY15	0.74	0.0155
GT_A_84_P860034		0.77	0.0157

GT_A_84_P843385	LOB	1.05	0.0161
GT_A_84_P11976	AT4G30640	0.93	0.0163
GT_A_84_P851136	AT1G61420	0.74	0.0164
GT_A_84_P863416	AT1G13000	0.80	0.0167
GT_A_84_P870394	STY1	1.28	0.0171
GT_A_84_P757111	AT2G31425	2.99	0.0180
GT_A_84_P14045	AT5G48430	1.83	0.0180
GT_A_84_P263680	AT2G47700	0.61	0.0180
GT_A_84_P95119	AT3G14840	0.89	0.0185
GT_A_84_P540376	LSH4	0.90	0.0185
GT_A_84_P15293	CYP78A5	1.27	0.0194
GT_A_84_P585555	AT2G30660	2.35	0.0195
GT_A_84_P12234	AT5G16030	0.71	0.0197
GT_A_84_P818943	AFB2	0.87	0.0206
GT_A_84_P10151	KCS19	1.59	0.0211
GT_A_84_P802613	DR301096	0.92	0.0215
GT_A_84_P737478	DR379000	0.92	0.0219
GT_A_84_P766473	AT5G25260	2.78	0.0228
GT_A_84_P23837	AT1G33640	1.39	0.0231
GT_A_84_P55910	WRKY41	1.87	0.0235
GT_A_84_P605694	AT5G64450	0.68	0.0235
GT_A_84_P13706	AT3G50140	1.31	0.0238
GT_A_84_P793695	AT1G07940	0.82	0.0246
GT_At_Specific_00037743	AT1G34245.1	0.88	0.0247
GT_A_84_P17191	AT1G07160	1.37	0.0249
GT_A_84_P79265	AT3G51330	1.65	0.0258
GT_A_84_P757039	CAX1	0.77	0.0261
GT_At_Specific_00200393	AT4G04490.1	3.51	0.0261
GT_A_84_P604617	AT5G02690	0.73	0.0265
GT_At_Specific_00015083	CYP78A5	0.97	0.0265
GT_A_84_P21338	AT4G01740	1.03	0.0275
GT_A_84_P556238	NP456028	1.06	0.0284
GT_A_84_P23796	GT_A_84_P23796_	2.13	0.0286
GT_A_84_P23287	AT4G22530	1.09	0.0287
GT_A_84_P24092	MYB111	1.08	0.0289
GT_A_84_P789360	ATHB4	1.15	0.0299
GT_A_84_P839339	BT015103	1.22	0.0304
GT_A_84_P22784	KNATM	2.00	0.0306
GT_A_84_P205978	CYP82C3	3.92	0.0307
GT_A_84_P13537	WRKY17	0.87	0.0308
GT_A_84_P802278	BX838959	1.40	0.0309
GT_At_Specific_00159109	RTFL13	3.03	0.0316
GT_A_84_P233599	AT1G61420	1.11	0.0321
GT_A_84_P596836	WNK3	2.76	0.0323
GT_A_84_P11645	CAX1	0.88	0.0324
GT_A_84_P854572	AT3G23310	1.00	0.0325
GT_A_84_P851716	AT4G16430	1.82	0.0327

GT_A_84_P516438	AT3G21570	1.76	0.0328
GT_A_84_P17326	WRKY33	0.65	0.0329
GT_A_84_P525902	AT1G60460	0.91	0.0329
GT_A_84_P751150	ANAC004	1.91	0.0330
GT_A_84_P542165	AT3G29720	1.01	0.0337
GT_A_84_P17640	TC383892	0.82	0.0339
GT_A_84_P19698	AT5G42830	1.80	0.0342
GT_At_Specific_00170991	AT3G43521.1	1.65	0.0342
GT_A_84_P857806	CRK	0.71	0.0344
GT_A_84_P22972	AT2G42980	0.91	0.0346
GT_A_84_P18368	AT3G16850	0.61	0.0357
GT_A_84_P14705	AT3G62780	0.89	0.0360
GT_A_84_P808262	AT5G55960	0.71	0.0366
GT_A_84_P848028	AT4G23470	0.65	0.0369
GT_A_84_P18510	AT4G04490	3.50	0.0371
GT_A_84_P855337	AtTLP2	1.12	0.0375
GT_A_84_P184814	AT5G02680	1.02	0.0376
GT_A_84_P830318	AT3G61670	0.92	0.0380
GT_A_84_P732884	AT2G13547	1.67	0.0387
GT_A_84_P831686	AT1G11060	1.44	0.0396
GT_A_84_P809044	TA27856_3702	2.25	0.0399
GT_A_84_P507017	AT5G44990	2.15	0.0399
GT_A_84_P16994	AT2G20835	0.98	0.0401
GT_A_84_P196584	AK118176	1.62	0.0411
GT_A_84_P512750	AT2G33780	2.72	0.0413
GT_A_84_P858250	AT2G38970	1.56	0.0414
GT_A_84_P22722	CSD1	1.30	0.0415
GT_A_84_P21554	CYCA2;1	1.39	0.0417
GT_A_84_P22847	AT1G14550	3.53	0.0419
GT_A_84_P23501	CCR4	1.59	0.0424
GT_A_84_P19829	AT5G58390	1.80	0.0430
GT_A_84_P10102	AT4G34800	2.33	0.0438
GT_A_84_P19235	ATHB4	1.16	0.0439
GT_A_84_P857505	ATBCB	0.98	0.0439
GT_A_84_P11151	AT5G24940	1.75	0.0440
GT_A_84_P145049	AT1G14880	4.59	0.0441
GT_A_84_P21001	AT1G44130	1.67	0.0442
GT_A_84_P815273	WRKY53	1.73	0.0444
GT_A_84_P12630	AT3G01490	0.78	0.0447
GT_A_84_P826500	TA42420_3702	1.68	0.0448
GT_A_84_P754312	AT1G61475	0.61	0.0459
GT_A_84_P17504	AT3G53730	0.74	0.0459
GT_A_84_P858214	BP835218	0.69	0.0461
GT_A_84_P845944	TC370161	1.51	0.0461
GT_A_84_P502174	AT3G09040	0.70	0.0471
GT_A_84_P217088	PLDGAMMA3	0.92	0.0472
GT_A_84_P510558	AT5G43490	0.96	0.0499

GT_A_84_P851199	AT1G63500	0.76	0.0501
GT_A_84_P827790	TA44076_3702	0.86	0.0505
GT_A_84_P506024	AT5G07770	0.80	0.0505
GT_A_84_P819987	AT3G57800	0.84	0.0509
GT_A_84_P575658	NP453103	1.63	0.0513
GT_A_84_P818871	TA34369_3702	0.99	0.0514
GT_A_84_P606349	AT4G28460	2.37	0.0520
GT_A_84_P555983	AT1G30190	1.62	0.0528
GT_A_84_P15354	AT2G20780	0.80	0.0530
GT_A_84_P13705	AT3G49950	2.08	0.0538
GT_A_84_P811297	AT3G10300	0.70	0.0541
GT_A_84_P277650	AT1G31885	1.75	0.0542
GT_A_84_P17607	ARK3	1.37	0.0543
GT_A_84_P850392	AT3G60960	0.88	0.0547
GT_A_84_P827393	AK118176	1.69	0.0551
GT_A_84_P21916	FMO1	4.45	0.0557
GT_A_84_P68474	AT2G28830	0.80	0.0558
GT_A_84_P862920	BP845828	1.78	0.0560
GT_A_84_P12224	AT5G66320	0.62	0.0562
GT_A_84_P815580	TA31703_3702	0.80	0.0563
GT_A_84_P20402	AT4G04510	2.82	0.0564
GT_At_Specific_00052578	AT1G55175.1	0.92	0.0576
GT_A_84_P567920	AT3G57950	1.70	0.0586
GT_A_84_P22344	AT4G23280	2.06	0.0589
GT_A_84_P24144	AT3G57640	0.82	0.0593
GT_A_84_P768403	IDL3	1.71	0.0594
GT_A_84_P21079	AT2G05320	0.68	0.0595
GT_A_84_P824452	AT3G09410	1.02	0.0608
GT_A_84_P555923	AT1G11120	0.68	0.0608
GT_A_84_P21007	AT2G43570	1.98	0.0609
GT_A_84_P71354	NP185775	1.35	0.0612
GT_A_84_P858035		1.29	0.0615
GT_A_84_P836690	AT1G61460	0.86	0.0629
GT_A_84_P790886	AT1G69340	1.07	0.0632
GT_A_84_P11999	CPK18	1.05	0.0633
GT_A_84_P603904	LOB	1.14	0.0634
GT_A_84_P613352	AT3G61280	2.23	0.0635
GT_A_84_P302360	AT5G37490	1.78	0.0640
GT_A_84_P268160	AT3G13950	1.68	0.0641
GT_A_84_P12784	ATEXO70H1	1.91	0.0648
GT_A_84_P23434	IPT5	0.99	0.0658
GT_A_84_P13406	ATTIM17-1	1.18	0.0659
GT_A_84_P13631	AT3G25190	1.33	0.0667
GT_A_84_P515570	AT3G28850	0.85	0.0669
GT_A_84_P860874	AT3G14415	0.82	0.0671
GT_A_84_P506839	AT4G22980	0.77	0.0675
GT_A_84_P819274	TAF6	1.05	0.0677

GT_A_84_P834307	**AT3G50590**	**1.17**	**0.0678**
GT_A_84_P558934	**AT3G12910**	**1.65**	**0.0682**
GT_A_84_P763290	**WRKY53**	**1.48**	**0.0693**
GT_A_84_P862714	**BX828000**	**0.63**	**0.0695**
GT_A_84_P305590	**AtPP2-A7**	**2.70**	**0.0700**
GT_A_84_P752390	**CNS0ADIY**	**2.77**	**0.0701**
GT_A_84_P530879	**RING1**	**1.60**	**0.0701**
GT_A_84_P21593	**AT1G49830**	**1.22**	**0.0712**
GT_A_84_P723098	**AT3G61198**	**2.55**	**0.0714**
GT_A_84_P15360	**MLO2**	**0.93**	**0.0721**
GT_A_84_P279580	**AT3G50800**	**1.03**	**0.0722**
GT_A_84_P206058	**AT4G37290**	**3.55**	**0.0729**
GT_A_84_P752286	**AT1G60330;AT1G6**	**1.71**	**0.0734**
GT_A_84_P818595	**ATSYTF**	**0.83**	**0.0736**
GT_A_84_P12851	**AT4G10500**	**2.23**	**0.0753**
GT_A_84_P15905	**AT5G40230**	**0.62**	**0.0755**
GT_A_84_P109962	**WAK3**	**4.40**	**0.0755**
GT_A_84_P18998	**AT1G72900**	**0.88**	**0.0757**
GT_A_84_P181424	**AT5G24640**	**1.15**	**0.0761**
GT_A_84_P18111	**CCoAMT**	**1.60**	**0.0762**
GT_A_84_P23077	**AT1G26390**	**2.60**	**0.0772**
GT_A_84_P543552	**TA32442_3702**	**0.90**	**0.0774**
GT_A_84_P242433	**AtRLP42**	**2.91**	**0.0775**
GT_A_84_P10141	**LECRKA4.2**	**1.79**	**0.0775**
GT_A_84_P548849	**AT1G13480**	**1.06**	**0.0776**
GT_A_84_P605805	**AT1G21326**	**0.75**	**0.0776**
GT_A_84_P756996	**AT2G46980**	**0.66**	**0.0778**
GT_A_84_P804827	**AIR12**	**0.75**	**0.0780**
GT_A_84_P763252	**AT4G21366**	**1.31**	**0.0783**
GT_A_84_P809908	**FAD2**	**0.84**	**0.0787**
GT_A_84_P10493	**RDR1**	**0.76**	**0.0796**
GT_A_84_P185874	**AT4G11170**	**2.83**	**0.0797**
GT_A_84_P13831	**AT4G23150**	**3.90**	**0.0806**
GT_A_84_P797768	**BP858161**	**0.92**	**0.0807**
GT_A_84_P12429	**AT1G63820**	**1.48**	**0.0809**
GT_A_84_P816751	**AT1G55840**	**0.63**	**0.0819**
GT_A_84_P23968	**ALD1**	**3.67**	**0.0821**
GT_A_84_P580710	**AT5G60290**	**1.20**	**0.0830**
GT_A_84_P818130	**AT1G62610**	**0.79**	**0.0832**
GT_A_84_P563122	**AT1G61160**	**2.15**	**0.0834**
GT_A_84_P815740	**AT5G21170**	**1.56**	**0.0839**
GT_A_84_P829761	**PRA1.G2**	**0.87**	**0.0848**
GT_A_84_P16097	**AT1G24140**	**0.95**	**0.0849**
GT_At_Specific_00234961	**AT4G33925.1**	**0.76**	**0.0851**
GT_A_84_P848224	**BX839205**	**1.46**	**0.0856**
GT_A_84_P556409	**AT3G60650**	**2.57**	**0.0860**
GT_A_84_P18423	**AT3G47480**	**2.38**	**0.0866**

GT_A_84_P19456	AT4G04500	3.89	0.0867
GT_A_84_P276740	AT5G47580	0.75	0.0869
GT_A_84_P854861	ATMTK	1.16	0.0869
GT_A_84_P834134	AT3G61010	0.63	0.0875
GT_A_84_P23566	AT5G64790	1.42	0.0875
GT_A_84_P235203	AT2G46980	0.65	0.0886
GT_A_84_P19766	COBL5	0.94	0.0893
GT_A_84_P832376	AT1G11330	0.71	0.0894
GT_A_84_P827321	AT4G11890	0.97	0.0904
GT_At_Specific_00172732	AT3G44765.1	0.83	0.0905
GT_A_84_P827497	AT5G22730	0.79	0.0907
GT_A_84_P557876	AT1G77660	1.13	0.0910
GT_A_84_P17193	ATCCS	1.14	0.0911
GT_A_84_P19294	AT1G26380	2.55	0.0918
GT_A_84_P849594	AT2G40270	0.76	0.0921
GT_A_84_P13799	AT4G11230	0.83	0.0921
GT_A_84_P576883	AT4G11000	1.39	0.0925
GT_A_84_P12905	AT4G27250	1.21	0.0930
GT_A_84_P14305	AT1G66780	1.45	0.0930
GT_A_84_P750360	AT1G47860	1.04	0.0931
GT_A_84_P15996	AT5G64110	3.35	0.0934
GT_A_84_P716286	AT5G48530	1.02	0.0944
GT_A_84_P92099	AT2G32880	0.70	0.0959
GT_A_84_P53570	AT2G41810	1.48	0.0961
GT_A_84_P833516	AT4G17660	1.67	0.0965
GT_A_84_P121762	AT2G23270	3.74	0.0967
GT_A_84_P526469	AT5G22150	2.17	0.0971
GT_A_84_P231759	AT3G57800	0.98	0.0974
GT_A_84_P869990	AT5G11140	1.81	0.0978
GT_A_84_P11048	AT4G34810	1.97	0.0979
GT_A_84_P798916	AT4G22520	2.72	0.0981
GT_A_84_P816444	AT2G34560	0.62	0.0984
GT_A_84_P523919	PRA1.G2	0.99	0.0990
GT_A_84_P241903	WR3	0.77	0.0993
GT_A_84_P12579	CYP96A5	1.74	0.0995
GT_A_84_P18230	SCPL51	0.91	0.0995
GT_A_84_P859937	AT1G71180	0.78	0.0999
GT_A_84_P836784	AT4G16807	1.88	0.1004
GT_At_Specific_00244143	AT5G02680.1	0.78	0.1004
GT_A_84_P18657	JKD	1.63	0.1007
GT_A_84_P563433	AT3G43583	1.23	0.1010
GT_A_84_P789377	AT4G39795	1.80	0.1020
GT_A_84_P613251	AT3G18250	2.20	0.1021
GT_A_84_P233559	AT1G10340	1.06	0.1024
GT_A_84_P848817	AT5G10760	2.88	0.1027
GT_A_84_P820600	AT5G50180	0.81	0.1028
GT_A_84_P15959	SUMO3	1.06	0.1034

GT_A_84_P820592	AT5G50180	0.95	0.1036
GT_A_84_P842536	BX815895	2.10	0.1037
GT_A_84_P15381	ATEXPA4	1.40	0.1038
GT_At_Specific_00028430	AT1G26200.1	0.70	0.1039
GT_A_84_P869713	AT5G52760	1.31	0.1040
GT_A_84_P11674	AT2G28940	1.24	0.1049
GT_A_84_P841548	NP227387	1.22	0.1049
GT_A_84_P754063	AT1G07901	0.77	0.1055
GT_A_84_P305950	NP453353	1.14	0.1055
GT_A_84_P830613	AT3G04485	0.97	0.1056
GT_A_84_P826648	IAA14	1.08	0.1056
GT_A_84_P752481	AT1G62590	1.14	0.1056
GT_A_84_P829753	PRA1.G2	0.99	0.1063
GT_A_84_P767554	AT5G59330	1.05	0.1066
GT_A_84_P841120	NP226396	1.81	0.1069
GT_A_84_P19374	NP208622	0.92	0.1079
GT_A_84_P735589	AK220995	1.18	0.1081
GT_A_84_P14710	SIB1	1.28	0.1083
GT_A_84_P111502	AT4G22090	1.36	0.1084
GT_A_84_P849777	AT5G57035	0.61	0.1089
GT_A_84_P833075	AT4G23230	1.17	0.1090
GT_A_84_P271800	EP1	1.22	0.1090
GT_A_84_P14793	AT1G21890	1.69	0.1091
GT_A_84_P229729	AT2G20142	0.90	0.1092
GT_A_84_P20594	PUCHI	1.15	0.1092
GT_A_84_P788891	AT2G41390	2.32	0.1093
GT_A_84_P20961	AT1G33440	1.20	0.1094
GT_A_84_P23363	IAA14	1.14	0.1095
GT_A_84_P613020	AT1G70400	0.69	0.1100
GT_A_84_P10549	AT1G72540	3.07	0.1102
GT_A_84_P15701	EDA36	2.37	0.1103
GT_A_84_P551808	AT3G01345	0.72	0.1103
GT_A_84_P22615	AT1G55200	1.17	0.1104
GT_A_84_P798026	GT_A_84_P798026	1.19	0.1110
GT_A_84_P177574	AtRLP39	3.71	0.1115
GT_A_84_P546754	AT4G23030	1.16	0.1117
GT_A_84_P22467	AT5G11140	1.75	0.1120
GT_A_84_P795094	AT5G28850	1.12	0.1122
GT_A_84_P501421	AT3G60670	1.11	0.1127
GT_A_84_P757444	SCRL17	1.18	0.1140
GT_A_84_P515865	AT5G26690	2.75	0.1141
GT_A_84_P831400	AT1G49320	0.98	0.1144
GT_A_84_P797722	EG464037	0.67	0.1146
GT_A_84_P182854	AT5G35413	2.09	0.1149
GT_A_84_P817317	ADR1-L1	1.55	0.1161
GT_A_84_P14898	AT5G10760	2.76	0.1165
GT_A_84_P598538	AT5G52710	2.21	0.1168

GT_A_84_P828531	**AT5G46340**	**0.60**	**0.1178**
GT_A_84_P597466	**AT4G32580**	**0.63**	**0.1186**
GT_A_84_P762419	**AT3G15518**	**1.22**	**0.1196**
GT_A_84_P15361	**AT2G45220**	**1.82**	**0.1198**
GT_A_84_P23220	**AT3G63380**	**1.03**	**0.1206**
GT_A_84_P564070	**AT1G78860**	**0.87**	**0.1208**
GT_A_84_P99776	**AtRLP41**	**4.12**	**0.1208**
GT_A_84_P17396	**CES101**	**1.04**	**0.1210**
GT_A_84_P17305	**WRKY55**	**1.64**	**0.1210**
GT_A_84_P811949	**AT1G17140**	**0.99**	**0.1214**
GT_A_84_P20058	**ETC2**	**0.82**	**0.1221**
GT_A_84_P245725	**CPuORF37**	**1.31**	**0.1221**
GT_A_84_P128706	**AT3G48080**	**2.16**	**0.1222**
GT_A_84_P767924	**YUC4**	**1.17**	**0.1222**
GT_A_84_P23314	**HAE**	**0.77**	**0.1230**
GT_A_84_P798312	**AT1G10120**	**1.37**	**0.1232**
GT_A_84_P17141	**AT1G50590**	**0.94**	**0.1234**
GT_A_84_P93039	**AT2G47690**	**1.53**	**0.1235**
GT_A_84_P12970	**AT4G17660**	**2.08**	**0.1239**
GT_A_84_P16831	**AT5G27420**	**1.09**	**0.1242**
GT_A_84_P15913	**AT5G42440**	**0.95**	**0.1245**
GT_A_84_P845497	**AT2G20780**	**0.89**	**0.1249**
GT_A_84_P837204	**ATCNGC9**	**1.75**	**0.1255**
GT_A_84_P223559	**LURP1**	**3.42**	**0.1256**
GT_A_84_P730499	**EG479048**	**4.01**	**0.1259**
GT_A_84_P76044	**AT2G34580**	**1.77**	**0.1262**
GT_A_84_P22600	**TOE2**	**1.04**	**0.1264**
GT_A_84_P836365	**AT5G39500**	**0.75**	**0.1268**
GT_A_84_P816349	**CRT3**	**1.45**	**0.1268**
GT_A_84_P11156	**WRKY50**	**3.22**	**0.1271**
GT_A_84_P856262	**AI994784**	**0.96**	**0.1273**
GT_A_84_P562186	**AT1G32570**	**1.04**	**0.1276**
GT_A_84_P765839	**AT4G38825**	**1.17**	**0.1277**
GT_A_84_P21751	**AT1G64260**	**0.90**	**0.1279**
GT_A_84_P766068	**AT5G36226**	**1.45**	**0.1279**
GT_A_84_P22923	**AtRLP24**	**1.34**	**0.1280**
GT_A_84_P750266	**AT1G42090**	**0.90**	**0.1284**
GT_A_84_P750761	**VIM2**	**1.09**	**0.1287**
GT_A_84_P20638	**GLIP1**	**4.85**	**0.1293**
GT_A_84_P544532	**AT1G58225**	**3.26**	**0.1296**
GT_A_84_P10606	**NP457196**	**1.33**	**0.1296**
GT_A_84_P172771	**AT5G06930**	**1.55**	**0.1296**
GT_A_84_P23919	**YLS9**	**1.60**	**0.1296**
GT_A_84_P18875	**GH3.17**	**0.82**	**0.1307**
GT_A_84_P829310	**AT3G04480**	**0.63**	**0.1311**
GT_A_84_P751743	**AT1G78260**	**0.71**	**0.1312**
GT_A_84_P577829	**AT4G28170**	**0.97**	**0.1314**

GT_A_84_P581848	JAG	1.14	0.1315
GT_A_84_P11863	NP281378	0.95	0.1321
GT_A_84_P767462	AT5G59990	1.21	0.1324
GT_A_84_P16446	AT3G16700	1.14	0.1329
GT_A_84_P12432	GSTU10	1.79	0.1330
GT_A_84_P787266	WRKY50	3.10	0.1331
GT_At_Specific_00198817	AT4G03130.1	0.98	0.1339
GT_At_Specific_00073457	ATGSTU10	2.07	0.1344
GT_A_84_P541664	PROPEP3	2.19	0.1345
GT_A_84_P12674	AT1G26400	2.43	0.1350
GT_A_84_P829148	TA45941_3702	1.29	0.1351
GT_A_84_P862999	AT5G25460	1.06	0.1352
GT_A_84_P250315	EXLB3	3.81	0.1364
GT_A_84_P833504	UPS2	1.28	0.1365
GT_A_84_P816388	PLP4	1.34	0.1366
GT_At_Specific_00234264	AT4G33355.1	2.35	0.1367
GT_A_84_P764950	AT4G22517	2.64	0.1369
GT_At_Specific_00268995	AT5G25920.1	2.61	0.1373
GT_A_84_P785407	AT1G76960	1.72	0.1373
GT_A_84_P831912	GTG2	0.64	0.1375
GT_A_84_P12956	AT4G39830	1.41	0.1376
GT_A_84_P855785	AT5G25460	1.01	0.1383
GT_A_84_P532052	AT1G30757	1.25	0.1389
GT_A_84_P23967	ATGLR2.9	1.19	0.1394
GT_A_84_P840321	NP214748	2.40	0.1395
GT_A_84_P853160	SYP121	1.15	0.1397
GT_A_84_P839084	AT1G61440	1.16	0.1406
GT_A_84_P758937	AT3G43622	1.05	0.1407
GT_A_84_P833567	AT5G04860	1.21	0.1411
GT_A_84_P596994	AT1G62610	0.77	0.1420
GT_A_84_P161863	AT3G04480	0.68	0.1421
GT_At_Specific_00092135	AT2G15042.1	1.12	0.1422
GT_A_84_P859101	TC363849	1.40	0.1424
GT_A_84_P835146	AT4G34480	1.05	0.1426
GT_A_84_P15602	ATGLR3.6	0.63	0.1428
GT_A_84_P22341	AT4G22520	2.61	0.1429
GT_At_Specific_00191593	AT3G61020.1	1.01	0.1432
GT_A_84_P13408	AT1G25460	1.05	0.1434
GT_A_84_P19719	AT5G48290	1.73	0.1436
GT_A_84_P13279	AT1G09550	1.19	0.1437
GT_A_84_P788452	AT2G20835	1.70	0.1439
GT_A_84_P19791	AT5G66910	1.00	0.1444
GT_A_84_P815292	LTA2	1.11	0.1446
GT_A_84_P12547	CYP71A12	2.77	0.1446
GT_A_84_P599263	AT4G33960	0.76	0.1448
GT_A_84_P69024	AT3G13030	1.09	0.1448
GT_A_84_P171963	AT2G39410	1.60	0.1450

GT_A_84_P588958	AT1G63630	1.24	0.1464
GT_A_84_P12207	AT5G62040	1.44	0.1465
GT_A_84_P842591	AT1G62910	0.77	0.1465
GT_A_84_P786027	DMR6	1.55	0.1477
GT_A_84_P213788	NP043480	1.91	0.1488
GT_A_84_P16705	AT4G31230	1.41	0.1490
GT_A_84_P513574	LURP1	3.62	0.1496
GT_A_84_P13202	anac036	2.06	0.1501
GT_A_84_P281930	AT3G47820	1.54	0.1502
GT_A_84_P16401	AtRLP20	1.10	0.1508
GT_At_Specific_00289637	AT5G47500.1	1.34	0.1509
GT_A_84_P18770	AT5G47500	1.35	0.1512
GT_At_Specific_00156221	AT3G21350.2	1.82	0.1512
GT_A_84_P562126	AT1G13470	3.67	0.1513
GT_A_84_P753500	AT1G51645	1.01	0.1519
GT_A_84_P590512	AtGRF7	1.03	0.1531
GT_A_84_P15883	CBP60G	1.50	0.1544
GT_A_84_P79105	AT1G66860	1.00	0.1545
GT_A_84_P16982	PDF1.5	1.39	0.1547
GT_A_84_P580951	AT1G68390	1.48	0.1549
GT_A_84_P511702	AT1G53625	1.12	0.1549
GT_A_84_P249495	AT5G52760	1.40	0.1553
GT_A_84_P762469	AT3G26742	0.70	0.1556
GT_A_84_P811245	AT4G40030	0.99	0.1558
GT_A_84_P181884	AT1G17140	0.94	0.1562
GT_A_84_P20326	AT3G50210	0.72	0.1563
GT_A_84_P562094	AT1G13200	1.12	0.1564
GT_A_84_P20114	AtRLP23	4.12	0.1580
GT_A_84_P576707	AT3G09020	1.37	0.1585
GT_At_Specific_00076071	AT1G76960.1	2.64	0.1586
GT_A_84_P836191	TA52349_3702	2.37	0.1587
GT_A_84_P753457	AT1G30282	1.27	0.1587
GT_A_84_P604856	AT5G59990	1.20	0.1589
GT_A_84_P731515	ATCNGC12	1.22	0.1591
GT_A_84_P23800	ATMKK4	0.83	0.1598
GT_A_84_P15115	AT3G24982	3.57	0.1600
GT_A_84_P761881	AT3G04640	1.40	0.1607
GT_A_84_P68884	AT4G21840	3.61	0.1608
GT_A_84_P14396	CPuORF34	0.89	0.1608
GT_A_84_P220458	AT4G23610	2.16	0.1609
GT_A_84_P758187	AT2G26267	1.49	0.1609
GT_A_84_P20996	AT1G51860	1.33	0.1613
GT_A_84_P826986	CRK6	4.02	0.1613
GT_A_84_P788748	AT1G13470	3.46	0.1614
GT_A_84_P792147	AT2G05250	0.83	0.1617
GT_A_84_P14808	AT4G30140	3.22	0.1620
GT_A_84_P13699	AT3G48550	1.06	0.1621

GT_A_84_P835729	AT1G22870	0.73	0.1622
GT_A_84_P836563	AT1G56145	0.87	0.1624
GT_A_84_P834874	AT2G21840	0.77	0.1626
GT_A_84_P844205	AT4G23160	1.99	0.1627
GT_At_Specific_00167632	AT3G30737.1	1.63	0.1641
GT_A_84_P21261	AT3G47540	1.68	0.1643
GT_A_84_P854812	AA394469	2.42	0.1649
GT_At_Specific_00087124	AT2G07320.1	1.48	0.1649
GT_A_84_P240335	AT3G20610	1.19	0.1675
GT_A_84_P847963	BX837267	1.10	0.1676
GT_A_84_P212598	AT1G63400	1.37	0.1679
GT_A_84_P759616	AT3G30405	0.77	0.1679
GT_A_84_P838477	NP032020	1.45	0.1683
GT_A_84_P804395	AtPP2-B10	0.70	0.1697
GT_A_84_P23208	AT3G60420	2.26	0.1708
GT_A_84_P820122	TA35380_3702	1.65	0.1717
GT_A_84_P764933	AT4G22513	2.90	0.1718
GT_A_84_P511918	AT3G01175	1.29	0.1724
GT_A_84_P855638	AT4G30350	0.90	0.1725
GT_A_84_P19115	AT2G43590	1.30	0.1730
GT_A_84_P11690	AT3G03410	1.33	0.1737
GT_A_84_P10800	ATSYTF	0.75	0.1742
GT_A_84_P152818	AT2G31990	0.97	0.1746
GT_A_84_P515035	AT5G54850	0.66	0.1748
GT_A_84_P15234	scpl31	1.31	0.1749
GT_A_84_P14410	AtRLP28	3.00	0.1750
GT_A_84_P14293	AT1G80310	1.30	0.1751
GT_A_84_P24055	AtRLP43	4.11	0.1753
GT_A_84_P820116	AT5G45490	0.95	0.1754
GT_A_84_P20706	AT5G59680	1.12	0.1755
GT_At_Specific_00235724	AT4G34480.1	1.07	0.1762
GT_A_84_P20320	AtMYB45	2.42	0.1770
GT_A_84_P507386	RALFL10	0.99	0.1771
GT_A_84_P17434	NDR1	1.42	0.1772
GT_A_84_P834735	TA51636_3702	1.71	0.1774
GT_A_84_P16550	scpl39	1.46	0.1775
GT_A_84_P23604	AT1G54890	1.43	0.1776
GT_A_84_P547579	AT4G05030	2.26	0.1785
GT_A_84_P858318	AT1G15910	1.06	0.1787
GT_A_84_P837183	AtRLP46	0.93	0.1791
GT_A_84_P844437	AT4G11450	0.65	0.1795
GT_A_84_P18663	AT5G05340	1.86	0.1801
GT_A_84_P275310	AT1G21245	2.03	0.1802
GT_A_84_P83519	AT1G62225	4.23	0.1806
GT_A_84_P18620	AT1G48210	0.96	0.1806
GT_A_84_P838519	AT1G11300	2.26	0.1807
GT_A_84_P11670	NDA2	1.30	0.1809

GT_A_84_P767658	AT5G55420	3.20	0.1810
GT_A_84_P760222	AT3G18145	1.42	0.1812
GT_A_84_P827583	TA43778_3702	0.97	0.1821
GT_A_84_P14560	PAD3	3.75	0.1821
GT_A_84_P856743	AT1G80420	1.01	0.1824
GT_A_84_P12310	AT1G76960	2.92	0.1831
GT_A_84_P20544	AT1G62670	1.52	0.1838
GT_A_84_P17210	AT1G51890	1.31	0.1839
GT_At_Specific_00276875	AT5G37017.1	1.69	0.1839
GT_A_84_P11803	AT3G46930	0.62	0.1849
GT_A_84_P137649	WAK1	2.44	0.1850
GT_A_84_P509353	AT3G28857	2.04	0.1866
GT_A_84_P826417	WRKY30	1.55	0.1867
GT_A_84_P813213	CYP706A1	1.24	0.1875
GT_A_84_P220478	CRT3	1.16	0.1883
GT_A_84_P15036	RLK1	2.54	0.1893
GT_A_84_P18669	AT5G07040	0.70	0.1898
GT_A_84_P761298	AT3G05727	2.86	0.1901
GT_A_84_P829110	AT4G30350	0.82	0.1902
GT_A_84_P836062	AT1G10600	1.41	0.1903
GT_A_84_P15376	anac042	2.94	0.1903
GT_A_84_P797066	EG438208	1.26	0.1910
GT_At_Specific_00031411	AT1G28620.1	0.89	0.1926
GT_A_84_P13764	AT3G44830	3.79	0.1928
GT_A_84_P22733	AT1G04560	1.32	0.1928
GT_A_84_P833492	AT4G34420	1.95	0.1938
GT_A_84_P845506	AT4G16850	1.10	0.1938
GT_A_84_P762494	AT3G49832	0.78	0.1940
GT_A_84_P569671	AT3G48640	3.21	0.1942
GT_A_84_P601105	OFP8	1.59	0.1945
GT_A_84_P856004	AFB2	0.83	0.1947
GT_A_84_P188254	CRK6	3.97	0.1950
GT_A_84_P831091	AT2G46980	1.07	0.1954
GT_A_84_P11516	AT1G20380	0.85	0.1956
GT_A_84_P16574	BGL2	3.50	0.1960
GT_A_84_P13654	LOH2	0.66	0.1965
GT_A_84_P553370	AtPP2-B13	2.54	0.1970
GT_A_84_P825764	AtPP2-B13	2.14	0.1973
GT_A_84_P536931	AT3G63450	0.75	0.1977
GT_At_Specific_00142126	AT3G09960.1	1.12	0.1988
GT_A_84_P12360	AT1G53980	1.13	0.1991
GT_A_84_P101246	AT5G22530	2.51	0.1993
GT_A_84_P521926	AT4G22510	2.26	0.1998
GT_A_84_P573074	AT2G31850	0.86	0.1999
GT_A_84_P835182	TA51914_3702	1.29	0.2001
GT_A_84_P19582	CYP705A3	1.24	0.2002
GT_A_84_P287700	AT5G38940	2.84	0.2002

GT_A_84_P15502	WAG2	2.16	0.2003
GT_A_84_P763170	AT4G35170	1.57	0.2003
GT_A_84_P833145	AT2G41820	1.99	0.2005
GT_A_84_P235303	AT1G66450	2.95	0.2008
GT_A_84_P509620	AT5G19950	1.51	0.2018
GT_A_84_P83469	PROPEP2	3.27	0.2019
GT_A_84_P281450	WAK2	2.11	0.2023
GT_A_84_P510027	AT2G04495	4.45	0.2023
GT_A_84_P163603	AT4G05095	1.29	0.2024
GT_A_84_P151538	ATSUC7	3.09	0.2029
GT_A_84_P16436	FRD3	1.40	0.2033
GT_A_84_P804912	TA25736_3702	1.14	0.2033
GT_A_84_P17268	PR1	4.96	0.2036
GT_A_84_P813414	TA30173_3702	0.62	0.2036
GT_A_84_P19588	ATEXLB1	2.29	0.2036
GT_A_84_P15821	HB51	1.57	0.2036
GT_A_84_P18826	AtZIP12	0.74	0.2037
GT_A_84_P559351	AT5G55460	3.33	0.2039
GT_At_Specific_00279174	AT5G38940.1	2.04	0.2040
GT_A_84_P853126	ATEXO70B2	1.24	0.2041
GT_A_84_P845284		1.13	0.2048
GT_A_84_P769387	AT5G66558	0.66	0.2049
GT_A_84_P818477	HDA6	0.95	0.2054
GT_A_84_P10391	AT3G31406	1.49	0.2064
GT_At_Specific_00097116	CKX2	1.62	0.2069
GT_A_84_P13567	AT2G47130	1.91	0.2072
GT_A_84_P510160	AT3G03240	0.93	0.2073
GT_A_84_P560214	AT5G45095	1.74	0.2076
GT_A_84_P608225	AT5G25010	0.76	0.2078
GT_A_84_P767599	AT5G50560	1.04	0.2079
GT_A_84_P11475	PAP3	1.11	0.2079
GT_A_84_P312233	AT4G23230	0.99	0.2079
GT_A_84_P76184	WRKY30	1.53	0.2080
GT_At_Specific_00139244	AT3G07030.1	1.19	0.2081
GT_At_Specific_00118818	AT2G37430.1	1.27	0.2084
GT_At_Specific_00048375	ATMKK4	0.83	0.2089
GT_A_84_P11669	UPS2	1.13	0.2095
GT_A_84_P838561	NP033841	1.04	0.2103
GT_A_84_P795628		1.40	0.2103
GT_A_84_P819781	MCA1	0.83	0.2107
GT_A_84_P612458	AT3G57440	0.76	0.2108
GT_A_84_P784626	CNBT1	1.16	0.2113
GT_A_84_P17047	ZFP1	2.00	0.2132
GT_A_84_P16510	AT1G35710	3.10	0.2134
GT_A_84_P21131	AT2G25520	0.68	0.2139
GT_A_84_P10014	AT4G10160	1.54	0.2146
GT_A_84_P196604	AT2G40530	0.77	0.2149

GT_A_84_P509231	AT2G41400	1.17	0.2150
GT_A_84_P754415	AT1G68238	1.21	0.2152
GT_A_84_P18901	EG463478	1.43	0.2154
GT_A_84_P18749	AT5G41920	1.13	0.2157
GT_A_84_P286230	CLE27	2.08	0.2160
GT_A_84_P21658	AT5G60980	0.95	0.2170
GT_A_84_P15571	AT3G44240	1.03	0.2177
GT_A_84_P12862	AT4G13190	1.49	0.2178
GT_A_84_P610349	IDA	1.94	0.2179
GT_A_84_P595614	AT4G14390	1.48	0.2191
GT_A_84_P571865	AT3G23420	0.71	0.2192
GT_A_84_P562069	AT1G05370	1.23	0.2195
GT_A_84_P208548	PROPEP1	0.87	0.2196
GT_At_Specific_00020209	AT1G18191.1	1.00	0.2201
GT_A_84_P16980	AT5G28300	0.63	0.2203
GT_A_84_P21864	AT1G73805	2.08	0.2206
GT_A_84_P150248	RRA2	0.75	0.2220
GT_A_84_P91079	AT5G22520	2.04	0.2224
GT_A_84_P538818	TC388624	1.87	0.2225
GT_A_84_P20322	AT3G49340	1.59	0.2227
GT_A_84_P14933	AT5G25460	0.83	0.2235
GT_A_84_P123602	AT3G59570	1.18	0.2237
GT_A_84_P10997	AT4G23310	2.83	0.2245
GT_A_84_P819884	AT4G38550	1.26	0.2251
GT_A_84_P17200	WAKL6	0.91	0.2254
GT_At_Specific_00164987	AT3G28440.1	1.80	0.2256
GT_A_84_P596942	AT1G34245	0.62	0.2258
GT_A_84_P853770	BP813084	1.10	0.2259
GT_At_Specific_00259496	AT5G16940.1	1.15	0.2268
GT_A_84_P797216	AT3G15536	3.44	0.2279
GT_A_84_P19999	AT1G52770	1.49	0.2282
GT_A_84_P787864	AT3G48640	2.41	0.2284
GT_A_84_P807972	NIA2	0.72	0.2295
GT_At_Specific_00270070	CBP60G	1.37	0.2295
GT_A_84_P530824	AT4G33925	0.68	0.2297
GT_A_84_P10209	AT5G25930	1.48	0.2298
GT_A_84_P10846	anac061	3.38	0.2301
GT_A_84_P834994	AT2G15530	1.02	0.2303
GT_A_84_P13433	ATSYP24	1.40	0.2306
GT_A_84_P811778	AT2G28840	1.51	0.2310
GT_A_84_P18273	AT2G42290	0.92	0.2312
GT_A_84_P764946	AT4G22505	4.18	0.2315
GT_A_84_P834773	LNG1	0.82	0.2320
GT_A_84_P13330	AT1G80140	1.15	0.2324
GT_A_84_P819564	TA34953_3702	1.44	0.2331
GT_A_84_P19160	ATPRB1	4.19	0.2335
GT_A_84_P538944	AT5G41450	1.48	0.2339

GT_A_84_P813113	TA29997_3702	1.25	0.2357
GT_A_84_P20180	AT2G32030	0.87	0.2360
GT_A_84_P23915	ATGRP2B	0.63	0.2365
GT_At_Specific_00309899	PROPEP1	0.91	0.2368
GT_A_84_P596819	AT1G04490	0.74	0.2370
GT_A_84_P825792	ATEXLB1	1.80	0.2372
GT_A_84_P17706	AT5G01870	2.15	0.2376
GT_A_84_P216298	AT5G38930	2.62	0.2379
GT_A_84_P603820	AT5G38320	1.47	0.2379
GT_A_84_P17003	AT1G05930	1.75	0.2384
GT_A_84_P839881	AT3G59660	0.61	0.2393
GT_A_84_P297484	AT1G09483	1.39	0.2398
GT_A_84_P528434	AT5G56960	3.01	0.2415
GT_A_84_P791939	AT5G60810	1.54	0.2417
GT_A_84_P836527	AT5G20870	1.31	0.2428
GT_A_84_P22564	AT5G50180	0.75	0.2431
GT_A_84_P194774	RGXT2	1.26	0.2434
GT_A_84_P799229	AT2G47130	2.62	0.2435
GT_A_84_P819731	AT4G32290	1.13	0.2438
GT_A_84_P120252	AT1G27110	0.62	0.2440
GT_At_Specific_00252658	AT5G10504.1	1.63	0.2440
GT_A_84_P18025	BGLU4	1.62	0.2442
GT_A_84_P790858	AT5G52790	0.69	0.2449
GT_A_84_P596217	AT2G23830	2.44	0.2449
GT_AtMg00720_885	orf107d	1.19	0.2463
GT_A_84_P21551	BGLU31	3.14	0.2465
GT_A_84_P588546	R30196	0.92	0.2467
GT_A_84_P21799	AT1G03230	1.23	0.2469
GT_A_84_P22552	AT5G47130	0.98	0.2477
GT_A_84_P833880	AT1G54890	2.03	0.2480
GT_A_84_P20089	AT2G17220	0.81	0.2483
GT_A_84_P18337	AOX1B	0.73	0.2484
GT_A_84_P21578	AT5G39670	1.72	0.2485
GT_A_84_P93839	AT1G78460	0.78	0.2493
GT_A_84_P823391	TA38773_3702	0.97	0.2495
GT_A_84_P830975	AT1G30755	0.75	0.2497
GT_A_84_P161393	AT3G18050	0.61	0.2498
GT_A_84_P14292	AT1G70530	0.78	0.2504
GT_A_84_P563999	AT1G54930	1.31	0.2504
GT_A_84_P125601	AT3G22240	2.45	0.2506
GT_A_84_P530104	AT5G52740	2.67	0.2507
GT_A_84_P521606	AT2G25260	1.31	0.2513
GT_A_84_P19725	CLC-C	0.66	0.2515
GT_At_Specific_00075118	MIR394B	1.25	0.2517
GT_A_84_P23315	XIH	0.61	0.2522
GT_A_84_P598130	PSF2	0.82	0.2526
GT_A_84_P23213	AT3G61810	0.95	0.2528

GT_A_84_P785070	DML1	0.82	0.2530
GT_A_84_P811444	SEC10	0.73	0.2534
GT_A_84_P764941	AT4G22485	3.79	0.2534
GT_A_84_P14856	INT4	0.68	0.2541
GT_A_84_P10568	ATEXO70B2	0.87	0.2552
GT_A_84_P21915	ATEXPA11	1.21	0.2565
GT_A_84_P837037	AT4G34480	1.13	0.2565
GT_A_84_P540992	AT1G63320	1.80	0.2571
GT_A_84_P835374	anac044	1.47	0.2573
GT_A_84_P825403	NST-K1	0.75	0.2585
GT_A_84_P830388	AT1G04500	1.56	0.2585
GT_A_84_P840794	NP221851	0.68	0.2589
GT_A_84_P12725	AT3G28510	3.72	0.2590
GT_At_Specific_00252961	AT5G10760.1	3.32	0.2600
GT_A_84_P760741	AtRLP34	3.30	0.2602
GT_At_Specific_00252235	FLC	2.03	0.2602
GT_A_84_P718723	AT3G57700	0.90	0.2611
GT_A_84_P845218	TC394882	0.77	0.2611
GT_A_84_P150678	AT5G06610	1.08	0.2612
GT_A_84_P581506	AT5G22555	1.60	0.2617
GT_A_84_P17627	MEK1	1.18	0.2625
GT_A_84_P15628	BG3	2.28	0.2626
GT_At_Specific_00175975	AT3G47540.1	1.92	0.2632
GT_At_Specific_00019644	AT1G17640.1	1.05	0.2636
GT_A_84_P783990	NP033471	1.69	0.2641
GT_A_84_P529333	AT1G11420	0.82	0.2643
GT_A_84_P831143	AT1G04010	0.68	0.2645
GT_A_84_P838326	AT1G63670	0.60	0.2645
GT_A_84_P801782	EG433575	1.53	0.2648
GT_A_84_P785972	CB185526	2.84	0.2648
GT_At_Specific_00017282	PDR12	3.04	0.2648
GT_A_84_P820429	GAE4	1.06	0.2652
GT_A_84_P787882	AT1G65920	0.68	0.2653
GT_A_84_P16146	AT1G64710	1.10	0.2660
GT_A_84_P17672	AT4G36150	1.27	0.2667
GT_A_84_P813225	CYP706A1	1.07	0.2669
GT_A_84_P13813	AT4G18650	2.54	0.2675
GT_A_84_P15804	AT4G16960	1.02	0.2675
GT_A_84_P761356	AT3G61898	1.35	0.2680
GT_A_84_P16938	AT5G63190	1.19	0.2683
GT_A_84_P19797	AT5G15730	0.63	0.2687
GT_At_Specific_00268583	AT5G25460.1	0.69	0.2692
GT_A_84_P10469	NF-YA5	0.93	0.2695
GT_A_84_P18500	AT4G01700	0.77	0.2700
GT_A_84_P565031	AT2G30550	1.06	0.2702
GT_A_84_P10502	AT1G18670	0.87	0.2704
GT_A_84_P18587	ATNRT2:1	2.43	0.2705

GT_A_84_P509286	AT3G10290	2.06	0.2720
GT_A_84_P511053	LSH2	0.88	0.2721
GT_A_84_P18943	AT1G70030	0.80	0.2722
GT_A_84_P17116	WAKL4	0.97	0.2723
GT_A_84_P753520	AT1G01448	1.75	0.2728
GT_A_84_P754635	AT1G70581	0.94	0.2730
GT_A_84_P723377	NP226912	1.06	0.2733
GT_A_84_P90919	AT3G61950	0.94	0.2739
GT_A_84_P855383	MSL10	0.75	0.2744
GT_A_84_P231379	AT5G46230	1.02	0.2744
GT_A_84_P10486	AtRLP2	0.63	0.2745
GT_A_84_P18477	BGLU27	4.77	0.2753
GT_A_84_P610036	AT5G38310	1.32	0.2759
GT_A_84_P15972	AT5G58840	1.99	0.2759
GT_A_84_P595029	AtPP2-B14	1.85	0.2762
GT_A_84_P824802	AT2G44370	1.52	0.2763
GT_A_84_P836180	AtRLP23	4.79	0.2769
GT_A_84_P24173	GT72B1	0.92	0.2778
GT_A_84_P850889		0.84	0.2781
GT_A_84_P51550	WRKY54	1.76	0.2792
GT_A_84_P13493	AT2G30670	3.41	0.2793
GT_A_84_P800034	AT4G16580	1.05	0.2794
GT_A_84_P21430	AT4G30250	0.76	0.2801
GT_A_84_P108542	AT2G41180	1.45	0.2806
GT_A_84_P20045	AT1G57630	2.23	0.2815
GT_A_84_P12882	TC371919	1.83	0.2817
GT_A_84_P853277	ANL2	1.03	0.2817
GT_A_84_P850662	AT5G13660	1.25	0.2818
GT_A_84_P186924	ARK1	2.01	0.2821
GT_A_84_P21889	AT1G74360	1.18	0.2823
GT_A_84_P788106	AT2G37390	1.41	0.2827
GT_A_84_P818026	RPS2	1.29	0.2837
GT_A_84_P860899	BX837858	1.68	0.2842
GT_A_84_P16839	NHL25	4.08	0.2856
GT_A_84_P819394	BAK1	0.69	0.2860
GT_A_84_P831728	ATGSTF12	1.39	0.2869
GT_A_84_P62200	ZPR1	1.17	0.2870
GT_A_84_P847681	AT3G08680	1.02	0.2872
GT_A_84_P113182	AT4G39670	0.95	0.2883
GT_A_84_P862059	BX831997	1.16	0.2889
GT_A_84_P191364	AT5G18870	0.91	0.2892
GT_A_84_P15868	anac090	3.87	0.2899
GT_A_84_P16393	NP454719	1.72	0.2914
GT_A_84_P842660	ACS3	1.91	0.2916
GT_A_84_P591116	ZPR2	2.99	0.2922
GT_A_84_P14067	AT5G54860	0.68	0.2922
GT_A_84_P580314	AT3G22235	2.61	0.2931

GT_A_84_P14338	ZFP4	0.77	0.2933
GT_At_Specific_00118768	AT2G37390.1	1.24	0.2941
GT_A_84_P242975	AT1G78410	1.99	0.2948
GT_A_84_P11446	PDR12	2.98	0.2948
GT_A_84_P789339	AY090982	1.13	0.2949
GT_A_84_P857326	AI992567	0.60	0.2957
GT_A_84_P20465	RPS2	1.35	0.2962
GT_A_84_P857966	PRXR1	1.14	0.2964
GT_A_84_P18717	IQD18	0.70	0.2967
GT_A_84_P15485	SYP121	1.19	0.2980
GT_A_84_P10231	AT1G73560	1.32	0.2980
GT_At_Specific_00299358	MIR156H	1.23	0.2981
GT_A_84_P838825	AT1G61660	0.96	0.2998
GT_A_84_P151328	AT3G14710	0.77	0.2999
GT_A_84_P758192	AT2G04864	1.32	0.3000
GT_At_Specific_00027730	AT1G24825.2	1.39	0.3002
GT_A_84_P757925	BX821511	1.57	0.3015
GT_A_84_P793627	AT4G00150	1.25	0.3024
GT_A_84_P55550	IAA29	1.48	0.3042
GT_A_84_P15382	CYP71A13	4.18	0.3042
GT_A_84_P11521	ho4	1.31	0.3043
GT_A_84_P758720	AT2G22520	0.98	0.3047
GT_A_84_P101346	AT2G44300	1.14	0.3047
GT_A_84_P13936	ATL43	1.10	0.3048
GT_A_84_P861191	TC401039	0.67	0.3051
GT_A_84_P611298	AT2G04515	3.08	0.3051
GT_A_84_P15524	AtRLP38	3.08	0.3057
GT_A_84_P170963	AT5G01990	0.89	0.3062
GT_A_84_P837580	AT1G70530	0.87	0.3080
GT_A_84_P772807	AT1G68240	1.59	0.3082
GT_A_84_P808136	TA27368_3702	3.06	0.3085
GT_A_84_P22681	AtRLP11	2.96	0.3086
GT_A_84_P11590	AT2G23200	1.35	0.3089
GT_At_Specific_00256424	AT5G14230.1	1.44	0.3090
GT_A_84_P22825	ATFRUCT5	1.01	0.3091
GT_At_Specific_00103305	AT2G24580.1	1.20	0.3107
GT_A_84_P751516	ATMRP12	1.55	0.3108
GT_A_84_P803664	ATCCS	1.21	0.3113
GT_A_84_P836210	TA52358_3702	0.74	0.3118
GT_A_84_P803729		1.46	0.3137
GT_A_84_P145439	AT2G31865	0.97	0.3145
GT_A_84_P754095	AT1G12411	1.12	0.3179
GT_A_84_P752720	SCRL7	1.11	0.3196
GT_A_84_P833128	CYP709B3	0.63	0.3207
GT_A_84_P133045	AT1G57790	0.75	0.3210
GT_A_84_P11053	TC376054	2.16	0.3212
GT_A_84_P834353	AT1G05310	1.46	0.3220

GT_A_84_P580199	DML1	1.05	0.3230
GT_A_84_P12336	AT1G10040	1.48	0.3240
GT_A_84_P84679	AT1G69520	0.84	0.3240
GT_A_84_P17616	AT4G23670	1.13	0.3247
GT_A_84_P753563	AT1G58590	0.91	0.3270
GT_A_84_P536372	DAR3	2.02	0.3276
GT_A_84_P582920	BAP2	1.34	0.3276
GT_A_84_P23274	AT1G29290	1.07	0.3282
GT_A_84_P547455	ILR2	2.05	0.3283
GT_At_Specific_00280807	AT5G40370.2	0.88	0.3290
GT_A_84_P535181	CLE44	1.41	0.3292
GT_A_84_P757683	MIR396A	1.12	0.3293
GT_A_84_P516780	AT5G43480	0.89	0.3294
GT_A_84_P761782	AT3G44765	0.79	0.3294
GT_A_84_P759889	AT3G44420	0.90	0.3295
GT_A_84_P824477	TA39987_3702	1.21	0.3296
GT_A_84_P838412	TA53499_3702	1.41	0.3301
GT_A_84_P832631	AT1G11330	0.91	0.3306
GT_A_84_P607185	CBL6	0.99	0.3308
GT_A_84_P576971	AT4G35810	1.75	0.3316
GT_A_84_P848876	AT3G44326	2.06	0.3321
GT_A_84_P14299	PR5	1.83	0.3328
GT_A_84_P19804	AT5G19880	1.96	0.3331
GT_A_84_P84999	AT3G28580	1.97	0.3337
GT_A_84_P721528	W43487	0.90	0.3338
GT_A_84_P855287	AT2G35840	0.74	0.3339
GT_A_84_P812910	AT3G22235	3.11	0.3345
GT_A_84_P13101	AT5G48710	1.05	0.3347
GT_At_Specific_00110620	CYP71A13	4.07	0.3349
GT_A_84_P815049	AT1G76010	1.34	0.3351
GT_A_84_P824914	AT4G18205	1.23	0.3352
GT_A_84_P804807	AIR12	0.95	0.3355
GT_A_84_P22854	AT1G51790	1.01	0.3360
GT_At_Specific_00165144	AT3G28580.1	1.94	0.3365
GT_A_84_P857638	CYP71A13	3.95	0.3368
GT_A_84_P759045	AT3G59880	0.66	0.3373
GT_At_Specific_00245713	AT5G04020.1	0.98	0.3378
GT_A_84_P592168	AT5G05300	1.29	0.3379
GT_A_84_P14569	ADOF2	1.10	0.3382
GT_A_84_P10668	AT2G16580	1.76	0.3389
GT_A_84_P829270	AT1G66620	0.82	0.3393
GT_A_84_P23871	AT2G21220	2.27	0.3396
GT_A_84_P12986	AT5G04020	0.75	0.3396
GT_A_84_P19994	ACA7	1.93	0.3396
GT_A_84_P14685	AT3G57730	1.15	0.3415
GT_A_84_P710653	TC401718	0.88	0.3416
GT_A_84_P834362	EIN4	1.03	0.3436

GT_A_84_P210278	**PXMT1**	1.90	**0.3439**
GT_A_84_P808719	**PR5**	1.63	**0.3439**
GT_A_84_P115542	**PAP5**	2.50	**0.3440**
GT_A_84_P22134	**CYP72A9**	0.76	**0.3444**
GT_A_84_P11777	**AT3G25610**	0.78	**0.3444**
GT_A_84_P826004	**ATEXO70B2**	0.88	**0.3445**
GT_A_84_P20824	**AT1G23070**	1.18	**0.3449**
GT_At_Specific_00141510	**AT3G09405.1**	1.26	**0.3450**
GT_A_84_P813238	**ANL2**	0.86	**0.3459**
GT_At_Specific_00127560	**AT2G44600.1**	0.89	**0.3476**
GT_A_84_P77129	**AT2G44370**	1.66	**0.3476**
GT_A_84_P565558	**AT5G56610**	1.20	**0.3477**
GT_A_84_P553387	**AT1G64405**	1.80	**0.3477**
GT_A_84_P15262	**GGT3**	1.41	**0.3482**
GT_A_84_P579146	**AT1G58602**	0.90	**0.3482**
GT_A_84_P837289	**AT5G45480**	1.19	**0.3483**
GT_A_84_P10002	**AT4G04810**	0.85	**0.3484**
GT_A_84_P795368	**BP578074**	0.68	**0.3485**
GT_A_84_P14076	**ZFP2**	1.52	**0.3490**
GT_A_84_P800174	**GGT3**	0.87	**0.3493**
GT_A_84_P798395	**AT1G59865**	1.88	**0.3520**
GT_A_84_P565178	**NIMIN-2**	1.86	**0.3528**
GT_A_84_P808198	**MLP28**	0.96	**0.3533**
GT_A_84_P546356	**AT1G76610**	2.21	**0.3534**
GT_A_84_P13998	**XGD1**	1.05	**0.3535**
GT_A_84_P15864	**AT5G18470**	1.45	**0.3542**
GT_A_84_P737765	**BX833772**	0.81	**0.3543**
GT_A_84_P824281	**PXMT1**	2.18	**0.3546**
GT_A_84_P813284	**ANL2**	1.12	**0.3557**
GT_A_84_P711052	**AtGH9C2**	0.81	**0.3567**
GT_A_84_P763555	**BX826473**	0.89	**0.3569**
GT_A_84_P807739	**TA27151_3702**	1.13	**0.3573**
GT_A_84_P854889	**CYP76C2**	1.71	**0.3574**
GT_A_84_P790814	**ATL43**	1.60	**0.3582**
GT_A_84_P816470	**TA32347_3702**	0.67	**0.3585**
GT_A_84_P19959	**AT1G32970**	1.57	**0.3597**
GT_A_84_P848646	**AT5G36930**	0.97	**0.3597**
GT_A_84_P814689	**AT1G21130**	0.85	**0.3598**
GT_A_84_P563250	**ATARD3**	3.19	**0.3599**
GT_A_84_P502380	**AT4G19970**	2.21	**0.3601**
GT_A_84_P798748	**EG507918**	0.82	**0.3602**
GT_A_84_P113812	**AT3G28155**	0.86	**0.3612**
GT_A_84_P763586	**TC369853**	0.69	**0.3620**
GT_A_84_P564195	**AT2G42900**	0.88	**0.3626**
GT_A_84_P808216	**TA27415_3702**	1.27	**0.3630**
GT_A_84_P155305	**AGL50**	1.16	**0.3633**
GT_A_84_P832725	**AT5G65600**	1.33	**0.3643**

GT_A_84_P10897	**CCR3**	**0.99**	**0.3655**
GT_A_84_P12295	**AT1G64450**	**0.88**	**0.3677**
GT_A_84_P11607	**CYP76C2**	**2.15**	**0.3688**
GT_A_84_P184644	**ALA1**	**1.08**	**0.3689**
GT_A_84_P10768	**AT3G04220**	**1.09**	**0.3689**
GT_A_84_P96816	**AtGDU2**	**2.64**	**0.3698**
GT_A_84_P600633	**AT2G04050**	**0.86**	**0.3706**
GT_A_84_P862797	**AT4G23670**	**1.21**	**0.3719**
GT_A_84_P11224	**AT5G52390**	**1.07**	**0.3743**
GT_A_84_P808407	**TA27519_3702**	**1.11**	**0.3749**
GT_A_84_P838375	**AT1G66980**	**1.12**	**0.3759**
GT_A_84_P825767	**AtPP2-B13**	**1.99**	**0.3769**
GT_A_84_P790935	**AT5G56610**	**1.12**	**0.3778**
GT_A_84_P759661	**AT3G27500**	**1.78**	**0.3798**
GT_A_84_P281230	**AT5G15160**	**0.77**	**0.3803**
GT_A_84_P11679	**AT2G23680**	**0.88**	**0.3808**
GT_At_Specific_00109086	**AT2G29452.1**	**1.40**	**0.3809**
GT_A_84_P18359	**AGC2-1**	**1.40**	**0.3816**
GT_A_84_P11210	**AT5G48380**	**0.97**	**0.3818**
GT_A_84_P218478	**AT1G74300**	**1.05**	**0.3819**
GT_A_84_P837240	**TA52911_3702**	**1.19**	**0.3822**
GT_A_84_P21145	**CHAT**	**2.22**	**0.3845**
GT_A_84_P826544	**GAE3**	**0.64**	**0.3860**
GT_A_84_P814568	**AT2G27340**	**0.89**	**0.3870**
GT_A_84_P305850	**GAD4**	**2.11**	**0.3882**
GT_A_84_P752604	**AT1G09500**	**2.67**	**0.3886**
GT_A_84_P102716	**AT4G18630**	**1.18**	**0.3893**
GT_A_84_P807753	**AT4G23670**	**1.16**	**0.3901**
GT_A_84_P825958	**EDS1**	**1.41**	**0.3904**
GT_A_84_P757088	**AT2G48075**	**0.91**	**0.3911**
GT_A_84_P18955	**AT1G30850**	**2.00**	**0.3913**
GT_A_84_P853555	**AT1G18020**	**1.29**	**0.3924**
GT_A_84_P844447	**AT4G18250**	**1.70**	**0.3956**
GT_A_84_P159935	**AT3G43436**	**0.97**	**0.3963**
GT_A_84_P56360	**AT5G57010**	**1.33**	**0.3965**
GT_A_84_P17847	**AT-HSFA9**	**1.15**	**0.3973**
GT_AntiSense_AtCg01100_503	**ndhA**	**0.90**	**0.3977**
GT_A_84_P23285	**PRXR1**	**0.85**	**0.3992**
GT_A_84_P578215	**AT1G44542**	**0.78**	**0.3993**
GT_A_84_P853578	**PRXR1**	**0.94**	**0.4009**
GT_A_84_P835528	**TA52085_3702**	**0.78**	**0.4015**
GT_A_84_P574406	**AT5G39240**	**0.82**	**0.4018**
GT_A_84_P757642	**AT2G20605**	**1.12**	**0.4034**
GT_A_84_P20780	**AT1G03495**	**3.38**	**0.4043**
GT_A_84_P22736	**AT1G09500**	**2.45**	**0.4049**
GT_A_84_P213308	**TC367969**	**2.14**	**0.4050**
GT_A_84_P784401	**FLC**	**1.70**	**0.4051**

GT_A_84_P838635	AT3G44730	1.20	0.4056
GT_A_84_P158785	TGG3	0.61	0.4060
GT_A_84_P768397	IDL2	1.33	0.4071
GT_A_84_P14446	FRK1	1.85	0.4096
GT_A_84_P20248	AT3G23230	2.57	0.4113
GT_A_84_P591383	AT5G45660	0.69	0.4113
GT_A_84_P835967	AT1G03560	0.68	0.4134
GT_A_84_P740197	AT1G69572	0.91	0.4155
GT_A_84_P528108	AT4G15150	0.71	0.4178
GT_A_84_P21825	ACA.l	0.97	0.4192
GT_A_84_P11414	AT1G05640	0.64	0.4192
GT_At_Specific_00157655	AT3G22436.1	0.68	0.4193
GT_A_84_P23655	MLO6	1.13	0.4201
GT_A_84_P844756	TC377347	1.25	0.4209
GT_A_84_P844839	EDS5	1.35	0.4210
GT_A_84_P15062	ATGA2OX4	1.18	0.4211
GT_A_84_P793020	RGL2	2.00	0.4212
GT_A_84_P11358	AT1G03820	1.19	0.4218
GT_At_Specific_00217982	AT4G19515.1	0.79	0.4225
GT_A_84_P808187	MLP34	0.82	0.4226
GT_A_84_P11580	AT2G47870	0.84	0.4231
GT_A_84_P240885	AT3G44175	1.23	0.4240
GT_A_84_P543246	AT4G37295	1.08	0.4247
GT_A_84_P166063	AT4G15620	0.93	0.4250
GT_A_84_P16384	AT2G25240	0.96	0.4282
GT_A_84_P145709	AT1G13130	1.42	0.4287
GT_A_84_P787412	AT4G28330	1.11	0.4294
GT_At_Specific_00211337	AT4G14365.1	1.39	0.4303
GT_A_84_P16270	AT1G33610	2.30	0.4303
GT_A_84_P796648	AT1G76610	1.34	0.4310
GT_A_84_P23270	AT4G18250	1.69	0.4314
GT_A_84_P18198	AT2G16660	0.79	0.4316
GT_A_84_P19046	ATGSTU12	1.80	0.4332
GT_A_84_P816533	TA32390_3702	1.08	0.4348
GT_A_84_P786557	AT2G25510	1.40	0.4348
GT_A_84_P556161	AT2G22620	0.83	0.4360
GT_A_84_P23678	ATRBOHB	0.73	0.4364
GT_A_84_P14579	AtRLP37	2.23	0.4366
GT_A_84_P816055	TA32017_3702	0.84	0.4371
GT_A_84_P814684	AT1G21130	1.02	0.4380
GT_A_84_P804462	GONST1	0.65	0.4386
GT_A_84_P753559	AT1G58590	0.70	0.4387
GT_A_84_P840641	NP221653	1.86	0.4429
GT_A_84_P831720	EMB1290	0.96	0.4434
GT_A_84_P862588	ATHPTC32	0.89	0.4435
GT_A_84_P589324	AT3G61840	0.85	0.4444
GT_A_84_P824668	MYB106	0.95	0.4457

GT_A_84_P764715	SCRL11	0.73	0.4463
GT_A_84_P753793	MLP28	0.73	0.4470
GT_At_Specific_00252033	AT5G09976.1	0.72	0.4511
GT_A_84_P11973	HMA2	0.65	0.4514
GT_A_84_P759141	AT3G44716	0.60	0.4515
GT_A_84_P834243	AT5G17040	0.73	0.4520
GT_A_84_P861523	TA28292_3702	1.07	0.4522
GT_A_84_P18336	AT1G26590	1.00	0.4533
GT_A_84_P721909	IQD20	2.17	0.4535
GT_A_84_P575998	AT4G14365	1.38	0.4543
GT_A_84_P755699	AT2G34580	1.26	0.4576
GT_A_84_P200454	PDLP6	0.96	0.4592
GT_A_84_P795261	ATHB5	0.77	0.4624
GT_A_84_P834278	AT1G62930	0.72	0.4627
GT_A_84_P12605	AT2G18010	2.29	0.4658
GT_A_84_P835631	AT3G56410	1.18	0.4671
GT_AtCg00640_307	rpl33	0.92	0.4680
GT_A_84_P60560	AT2G25510	1.38	0.4698
GT_A_84_P23719	MYB58	1.10	0.4701
GT_A_84_P845837	AT1G58602	0.84	0.4705
GT_A_84_P769485	AT5G60142	0.81	0.4706
GT_A_84_P582597	AT1G09932	0.77	0.4718
GT_A_84_P14558	AT3G22820	0.99	0.4728
GT_A_84_P821660	RGXT2	0.72	0.4735
GT_A_84_P20522	CYP79B2	1.46	0.4736
GT_A_84_P222519	AT1G21130	0.94	0.4739
GT_A_84_P817983	SVL2	0.93	0.4744
GT_A_84_P10330	AT5G65600	1.12	0.4771
GT_At_Specific_00297969	ANK	2.79	0.4778
GT_A_84_P13964	AT5G14230	1.04	0.4784
GT_A_84_P12477	AT1G79680	0.99	0.4806
GT_At_Specific_00003309	AT1G03820.1	0.96	0.4806
GT_A_84_P838876	NP039098	1.04	0.4823
GT_A_84_P13713	AT3G51660	0.85	0.4824
GT_A_84_P19949	GSTF5	2.37	0.4825
GT_A_84_P839412	AY735672	0.84	0.4831
GT_A_84_P20862	AT1G02030	1.21	0.4833
GT_A_84_P752095	AT1G35270	0.84	0.4840
GT_A_84_P16903	ANK	2.81	0.4845
GT_A_84_P16285	CTF2B	1.47	0.4848
GT_At_Specific_00222232	AT4G23215.1	0.89	0.4861
GT_A_84_P845509	BX839532	1.02	0.4867
GT_A_84_P14803	AT4G29020	1.51	0.4873
GT_A_84_P565337	AT4G21865	0.93	0.4897
GT_A_84_P13682	NP208377	1.24	0.4902
GT_A_84_P856641	BP847842	1.29	0.4907
GT_A_84_P18078	AT1G72000	1.95	0.4914

GT_A_84_P842922	**AT3G13980**	**1.23**	**0.4939**
GT_A_84_P12139	**AT5G44400**	**0.78**	**0.4947**
GT_A_84_P74244	**NP453663**	**1.10**	**0.4954**
GT_A_84_P756613	**AT2G10080**	**1.57**	**0.4958**
GT_A_84_P20823	**SCPL32**	**1.29**	**0.4961**
GT_A_84_P22250	**ACA11**	**0.95**	**0.4973**
GT_A_84_P21470	**RHF1A**	**0.89**	**0.5002**
GT_A_84_P509388	**AT3G52520**	**0.87**	**0.5006**
GT_A_84_P12315	**ATMGL**	**1.18**	**0.5039**
GT_A_84_P20191	**MYB106**	**0.96**	**0.5047**
GT_A_84_P18118	**NAS4**	**1.69**	**0.5051**
GT_A_84_P13577	**AT1G36940**	**0.91**	**0.5054**
GT_A_84_P701035	**IBS1**	**0.75**	**0.5056**
GT_A_84_P20210	**ATGSTF11**	**1.21**	**0.5067**
GT_A_84_P17317	**ATCNGC3**	**1.27**	**0.5086**
GT_A_84_P820887	**AT3G53190**	**1.39**	**0.5123**
GT_A_84_P834838	**PSF2**	**0.76**	**0.5161**
GT_A_84_P506400	**AT1G63130**	**0.85**	**0.5169**
GT_A_84_P19044	**AT1G21440**	**0.76**	**0.5179**
GT_A_84_P522028	**AT5G12930**	**0.85**	**0.5185**
GT_A_84_P23533	**PSD2**	**0.75**	**0.5191**
GT_A_84_P798815	**CYP79B2**	**1.38**	**0.5219**
GT_At_Specific_00029125	**AT1G26771.1**	**1.72**	**0.5225**
GT_A_84_P54620	**AT5G64780**	**1.14**	**0.5271**
GT_A_84_P18819	**ATNRT2.3**	**2.41**	**0.5308**
GT_A_84_P765683	**EG511673**	**1.11**	**0.5356**
GT_A_84_P14443	**AT2G29310**	**1.10**	**0.5356**
GT_A_84_P849694	**ARF10**	**1.12**	**0.5360**
GT_A_84_P15484	**CPuORF63**	**1.19**	**0.5411**
GT_A_84_P216568	**AT1G78030**	**0.67**	**0.5435**
GT_A_84_P22554	**NF-YB2**	**0.71**	**0.5481**
GT_A_84_P10905	**AT3G57630**	**1.11**	**0.5511**
GT_A_84_P196504	**AT5G18010**	**1.33**	**0.5535**
GT_A_84_P786204	**ATEXT3**	**1.18**	**0.5580**
GT_A_84_P805772	**TA26158_3702**	**0.95**	**0.5612**
GT_A_84_P829672	**AT1G09932**	**0.81**	**0.5656**
GT_A_84_P786630	**AT4G30060**	**0.81**	**0.5691**
GT_At_Specific_00282720	**MIR166E**	**1.08**	**0.5692**
GT_A_84_P769532	**AT5G08391**	**0.73**	**0.5697**
GT_A_84_P195614	**AT5G23840**	**0.80**	**0.5718**
GT_A_84_P23394	**ASA1**	**0.92**	**0.5718**
GT_A_84_P284670	**AT2G33850**	**1.10**	**0.5739**
GT_A_84_P12277	**AT1G08105**	**1.16**	**0.5779**
GT_A_84_P20499	**AT4G33810**	**0.66**	**0.5783**
GT_A_84_P503244	**AT4G08593**	**1.34**	**0.5810**
GT_A_84_P825966	**EDS1**	**1.07**	**0.5823**
GT_A_84_P23247	**AT4G08570**	**1.64**	**0.5837**

GT_A_84_P842635	**TC404116**	**1.48**	**0.5841**
GT_A_84_P813910	**AT4G18570**	**0.74**	**0.5846**
GT_A_84_P212918	**EDS5**	**1.26**	**0.5886**
GT_A_84_P243315	**AT3G50900**	**0.64**	**0.5905**
GT_A_84_P22046	**TTG2**	**1.16**	**0.5958**
GT_A_84_P53580	**AtMYB82**	**0.69**	**0.6001**
GT_A_84_P141269	**AT5G18050**	**1.82**	**0.6027**
GT_A_84_P758488	**AT2G18969**	**2.31**	**0.6038**
GT_A_84_P797911	**GONST1**	**0.64**	**0.6060**
GT_A_84_P51570	**UBQ12**	**0.83**	**0.6065**
GT_At_Specific_00241898	**AKN2**	**1.16**	**0.6077**
GT_A_84_P767304	**TC391578**	**0.91**	**0.6162**
GT_A_84_P512146	**AT4G20300**	**0.63**	**0.6162**
GT_A_84_P548023	**ATPP2-A9**	**0.69**	**0.6189**
GT_A_84_P808037	**AT1G28400**	**0.70**	**0.6224**
GT_A_84_P554482	**AT3G03850**	**2.18**	**0.6232**
GT_A_84_P765944	**AT4G21903**	**0.92**	**0.6295**
GT_A_84_P19576	**AKN2**	**0.92**	**0.6357**
GT_A_84_P20172	**CYP710A2**	**0.77**	**0.6370**
GT_A_84_P830547	**AT2G30720**	**0.65**	**0.6469**
GT_A_84_P839281	**ATOPT2**	**0.93**	**0.6501**
GT_A_84_P18553	**B120**	**0.80**	**0.6509**
GT_A_84_P18265	**AT2G18480**	**0.83**	**0.6786**
GT_A_84_P759689	**AT3G44262**	**0.97**	**0.6826**
GT_A_84_P591083	**AT3G51410**	**1.92**	**0.6907**
GT_At_Specific_00150650	**AT3G16800.3**	**1.62**	**0.6923**
GT_A_84_P841875	**NP230522**	**0.92**	**0.6993**
GT_A_84_P18550	**AT4G20780**	**0.96**	**0.7105**
GT_A_84_P111252	**AT5G47730**	**0.70**	**0.7115**
GT_A_84_P846594	**AT5G59050**	**0.95**	**0.7204**
GT_A_84_P828851	**AT1G25370**	**0.75**	**0.7420**
GT_A_84_P825275	**AT1G61260**	**0.69**	**0.7506**
GT_A_84_P21842	**FMO_GS-OX1**	**0.75**	**0.7701**

Down-regulated genes in *ein* 2

		**Stress treated *ein* 2 vs Col-0 Fold change**	
**ProbeName**	**GeneName**	**GeoMean (3 replicate Log2 Fold change)**	**P_Value**
GT_A_84_P18938	**AT1G08800**	**-1.01**	**0.0001**
GT_AtMg01260_1123	**orf205**	**-0.85**	**0.0001**
GT_A_84_P19167	**PAB7**	**-0.92**	**0.0002**
GT_A_84_P858159	**AT1G20730**	**-3.27**	**0.0003**
GT_A_84_P807532	**GAMMA-TIP**	**-1.50**	**0.0006**
GT_A_84_P859300	**ATHSP90.1**	**-2.24**	**0.0008**
GT_A_84_P201578	**AT3G24020**	**-3.35**	**0.0009**
GT_A_84_P22453	**AT1G06540**	**-1.54**	**0.0009**
GT_A_84_P12947	**AtbZIP7**	**-1.01**	**0.0010**

GT_At_Specific_00118094	GAMMA-TIP	-1.65	0.0012
GT_A_84_P199944	AT2G36100	-1.98	0.0013
GT_A_84_P22572	ATHSP90.1	-2.82	0.0013
GT_A_84_P10251	AtPP2-A6	-1.54	0.0013
GT_A_84_P833665	EGY3	-2.26	0.0013
GT_A_84_P20285	AT3G13660	-2.67	0.0015
GT_AntiSense_AtMg00680_870	orf122c	-1.28	0.0016
GT_A_84_P561025	AT5G18340	-1.45	0.0019
GT_A_84_P16459	ATGSL04	-0.70	0.0020
GT_A_84_P311003	AT5G14150	-1.82	0.0020
GT_A_84_P713101	AT4G28850	-1.44	0.0020
GT_A_84_P17353	AT3G01190	-2.80	0.0021
GT_A_84_P238905	scpl1	-1.76	0.0021
GT_A_84_P755656	NP10426972	-0.81	0.0023
GT_A_84_P770341	NP1098887	-0.80	0.0023
GT_A_84_P10802	AT3G20380	-1.20	0.0029
GT_AntiSense_AtCg00480_246	atpB	-0.63	0.0032
GT_A_84_P805209	AT1G62740	-0.95	0.0033
GT_A_84_P23984	AGD11	-1.27	0.0034
GT_A_84_P21375	AT4G13580	-2.22	0.0034
GT_A_84_P754326	AT1G22065	-0.98	0.0034
GT_A_84_P23899	AT2G29500	-3.29	0.0034
GT_A_84_P162633	EGY3	-2.10	0.0035
GT_AntiSense_AtMg01210_110	orf101b	-1.15	0.0037
GT_A_84_P844993	TC364679	-1.07	0.0037
GT_A_84_P283680	AT3G07090	-1.20	0.0041
GT_A_84_P18258	TPS10	-3.03	0.0043
GT_A_84_P22747	AT1G04150	-1.47	0.0044
GT_A_84_P834403	AT4G09150	-1.24	0.0046
GT_A_84_P594615	AT3G28550	-3.64	0.0052
GT_A_84_P20916	VND7	-0.67	0.0053
GT_A_84_P809682	AT5G54940	-0.82	0.0054
GT_A_84_P556555	AT5G02490	-0.70	0.0055
GT_AtMg00710_881	orf120	-1.28	0.0055
GT_A_84_P549559	AT5G38100	-2.92	0.0058
GT_A_84_P753030	RTFL15	-1.29	0.0059
GT_A_84_P23490	AtPP2-A8	-1.48	0.0060
GT_A_84_P567667	AT2G20080	-3.22	0.0061
GT_A_84_P848857	AT3G07090	-1.25	0.0065
GT_A_84_P10453	AT1G04130	-0.85	0.0065
GT_A_84_P65364	AT5G04250	-0.66	0.0065
GT_A_84_P791215	EG467942	-1.11	0.0066
GT_A_84_P17646	AT4G30170	-1.64	0.0071
GT_A_84_P13526	GAMMA-TIP	-1.29	0.0072
GT_A_84_P17242	RD28	-1.85	0.0073
GT_A_84_P803035	CD534138	-0.62	0.0080
GT_A_84_P15341	VHA-E3	-1.31	0.0081

GT_A_84_P19874	AT1G77030	-0.89	0.0081
GT_At_Specific_00022274	AT1G20015.1	-0.90	0.0082
GT_A_84_P19403	AT3G55230	-2.49	0.0082
GT_A_84_P826259	TA42145_3702	-0.79	0.0084
GT_A_84_P770100	ATMG00710	-0.76	0.0086
GT_A_84_P806805	AT3G09440	-1.91	0.0087
GT_A_84_P17027	AT1G30870	-3.62	0.0087
GT_A_84_P16179	AT1G10980	-0.87	0.0088
GT_A_84_P839982	AT1G63410	-3.52	0.0088
GT_A_84_P295894	AT2G20940	-0.91	0.0089
GT_A_84_P186914	BSMT1	-5.68	0.0089
GT_A_84_P545880	AT4G29760	-1.34	0.0093
GT_A_84_P20057	AT1G63580	-1.85	0.0094
GT_A_84_P756881	AT2G07771	-0.76	0.0095
GT_A_84_P815000	BTI2	-0.84	0.0096
GT_A_84_P15912	AT5G42180	-3.23	0.0097
GT_A_84_P582862	AT2G29790	-4.58	0.0099
GT_A_84_P755464	AT2G16190	-3.11	0.0102
GT_A_84_P14349	MYB52	-1.19	0.0103
GT_A_84_P14417	AT2G20560	-2.98	0.0104
GT_A_84_P76404	UNE11	-2.45	0.0108
GT_A_84_P246785	AT2G27370	-1.24	0.0108
GT_A_84_P808886	TA27788_3702	-0.67	0.0109
GT_A_84_P102076	AT5G64690	-0.62	0.0112
GT_A_84_P15946	AT5G51440	-2.47	0.0112
GT_A_84_P544270	AT5G44780	-0.66	0.0113
GT_A_84_P12352	AT1G02190	-0.93	0.0113
GT_A_84_P869042	HSP81-3	-1.18	0.0114
GT_At_Specific_00119739	AT2G38240.1	-1.11	0.0118
GT_A_84_P154335	NIC3	-2.80	0.0120
GT_A_84_P211128	AT1G80240	-1.07	0.0120
GT_A_84_P23488	CYP706A3	-0.82	0.0122
GT_A_84_P835829	TA52200_3702	-1.00	0.0123
GT_A_84_P832698	AT5G56000	-1.25	0.0124
GT_A_84_P826237	AT5G64680	-0.65	0.0124
GT_A_84_P205218	AT3G48340	-1.89	0.0125
GT_AntiSense_AtMg01100_104	orf105a	-0.73	0.0127
GT_A_84_P168163	HSP81-2	-1.47	0.0127
GT_A_84_P603542	AT3G48770	-0.97	0.0128
GT_A_84_P19363	ATHSP17.4	-4.35	0.0128
GT_A_84_P11833	AT3G53830	-2.23	0.0129
GT_A_84_P863474	BP581534	-1.50	0.0129
GT_A_84_P758621	AT2G01008	-1.04	0.0129
GT_A_84_P770393	TC402042	-0.65	0.0130
GT_A_84_P19603	AT1G62740	-0.69	0.0131
GT_A_84_P756324	AT2G07694	-0.88	0.0131
GT_A_84_P22953	CLPB4	-1.70	0.0135

GT_A_84_P819821	**AT2G20560**	**-2.82**	**0.0136**
GT_A_84_P810368	**HSP91**	**-0.64**	**0.0137**
GT_A_84_P267260	**WDL1**	**-0.64**	**0.0138**
GT_A_84_P809477	**PIP1;4**	**-1.51**	**0.0139**
GT_A_84_P788143	**AT3G24020**	**-2.31**	**0.0140**
GT_A_84_P21103	**AT2G41480**	**-1.06**	**0.0140**
GT_A_84_P10688	**AT1G30070**	**-1.83**	**0.0140**
GT_A_84_P20591	**AT5G17820**	**-2.61**	**0.0140**
GT_A_84_P807528	**GAMMA-TIP**	**-1.82**	**0.0141**
GT_A_84_P23446	**SHN2**	**-1.72**	**0.0142**
GT_A_84_P20419	**LBD2**	**-4.45**	**0.0146**
GT_A_84_P13741	**AT3G57600**	**-0.70**	**0.0149**
GT_At_Specific_00217858	**AT4G19430.1**	**-3.06**	**0.0151**
GT_A_84_P21922	**CYP87A2**	**-2.92**	**0.0152**
GT_A_84_P16049	**AT2G07713**	**-1.38**	**0.0154**
GT_A_84_P133635	**AT3G12050**	**-0.85**	**0.0155**
GT_A_84_P831858	**AT1G53860**	**-0.60**	**0.0159**
GT_A_84_P255680	**AT5G05960**	**-0.98**	**0.0159**
GT_A_84_P20897	**AT1G22440**	**-1.11**	**0.0160**
GT_AntiSense_AtMg00450_729	**orf106b**	**-0.90**	**0.0160**
GT_A_84_P93449	**AT4G28150**	**-1.02**	**0.0166**
GT_A_84_P822968	**KNAT7**	**-0.84**	**0.0168**
GT_A_84_P10761	**AT3G09560**	**-0.61**	**0.0168**
GT_A_84_P21609	**NF-YC2**	**-1.68**	**0.0170**
GT_A_84_P22211	**AT3G48720**	**-1.00**	**0.0177**
GT_A_84_P596218	**AT2G25130**	**-1.24**	**0.0178**
GT_A_84_P752754	**AT1G64195**	**-2.05**	**0.0178**
GT_A_84_P23846	**AtGolS1**	**-1.74**	**0.0179**
GT_AntiSense_AtMg00260_662	**orf101a**	**-0.69**	**0.0179**
GT_A_84_P11209	**AT5G48110**	**-1.88**	**0.0179**
GT_A_84_P850377	**emb2444**	**-0.74**	**0.0181**
GT_A_84_P809147	**PIP2A**	**-0.82**	**0.0182**
GT_A_84_P12305	**AT1G52050**	**-2.00**	**0.0183**
GT_A_84_P787157	**AT5G48110**	**-1.84**	**0.0184**
GT_A_84_P298084	**APUM19**	**-1.17**	**0.0186**
GT_A_84_P800605	**UGT88A1**	**-0.89**	**0.0187**
GT_A_84_P20212	**AT3G04710**	**-1.43**	**0.0188**
GT_A_84_P739326	**AT2G43540**	**-0.78**	**0.0188**
GT_A_84_P826776	**AT4G12400**	**-3.16**	**0.0189**
GT_A_84_P819099	**TA34521_3702**	**-2.70**	**0.0191**
GT_A_84_P551649	**AT1G74055**	**-1.05**	**0.0191**
GT_AtMg01370_1181	**orf111d**	**-0.72**	**0.0191**
GT_A_84_P290124	**ATPSK2**	**-0.89**	**0.0193**
GT_A_84_P14320	**ATHSP101**	**-3.39**	**0.0196**
GT_A_84_P560703	**AT3G07150**	**-0.79**	**0.0197**
GT_A_84_P15926	**ARAC2**	**-0.78**	**0.0202**
GT_A_84_P835772	**TA52178_3702**	**-1.20**	**0.0202**

GT_A_84_P287150	**HSP81-3**	**-1.21**	**0.0202**
GT_A_84_P22108	**LON4**	**-1.00**	**0.0203**
GT_A_84_P11914	**AT4G12400**	**-3.08**	**0.0204**
GT_A_84_P19141	**MYB12**	**-0.93**	**0.0204**
GT_A_84_P757106	**AT2G07795**	**-0.83**	**0.0205**
GT_A_84_P13535	**AT2G38240**	**-1.15**	**0.0207**
GT_A_84_P283170	**AT2G21045**	**-1.58**	**0.0207**
GT_A_84_P849648	**SAD2**	**-0.97**	**0.0208**
GT_A_84_P17413	**ROF1**	**-1.50**	**0.0209**
GT_A_84_P12484	**AT1G58170**	**-0.92**	**0.0212**
GT_At_Specific_00290333	**AT5G48110.1**	**-1.31**	**0.0214**
GT_A_84_P14457	**TC399888**	**-3.75**	**0.0216**
GT_AntiSense_AtMg00630_839	**orf110b**	**-0.91**	**0.0216**
GT_A_84_P13988	**AT5G25450**	**-1.92**	**0.0219**
GT_A_84_P15440	**AT1G07400**	**-3.52**	**0.0220**
GT_A_84_P770206	**ATMG01230;AT2G**	**-0.84**	**0.0220**
GT_A_84_P19592	**AT4G36770**	**-1.38**	**0.0221**
GT_A_84_P836184	**TA52348_3702**	**-1.46**	**0.0223**
GT_A_84_P845241		**-1.28**	**0.0227**
GT_A_84_P163223	**AT3G13760**	**-1.57**	**0.0227**
GT_A_84_P517834	**AT1G16290**	**-1.27**	**0.0227**
GT_A_84_P15043	**AT5G62340**	**-3.83**	**0.0230**
GT_A_84_P143109	**AT1G79920**	**-1.14**	**0.0234**
GT_A_84_P578332	**MRH6**	**-2.97**	**0.0236**
GT_A_84_P793361	**NRPB1**	**-0.64**	**0.0236**
GT_A_84_P562425	**AT2G43390**	**-1.27**	**0.0237**
GT_A_84_P166573	**AT1G03700**	**-1.52**	**0.0237**
GT_A_84_P21270	**ATARLA1B**	**-1.66**	**0.0239**
GT_A_84_P310283	**AT5G01320**	**-1.21**	**0.0242**
GT_A_84_P768444	**AT5G36350**	**-1.02**	**0.0242**
GT_A_84_P799584	**AT1G62975**	**-1.72**	**0.0243**
GT_A_84_P529136	**AT5G22970**	**-2.27**	**0.0244**
GT_A_84_P509902	**AT1G36280**	**-0.68**	**0.0247**
GT_A_84_P10627	**AT2G20880**	**-1.11**	**0.0247**
GT_A_84_P785012	**AtGolS1**	**-1.94**	**0.0247**
GT_AntiSense_AtMg01230_111	**orf145b**	**-0.80**	**0.0249**
GT_A_84_P18818	**AT5G60530**	**-1.16**	**0.0249**
GT_A_84_P809138	**TA27909_3702**	**-0.91**	**0.0249**
GT_A_84_P809081	**RANBP1**	**-0.84**	**0.0257**
GT_A_84_P831256	**TA48759_3702**	**-3.46**	**0.0257**
GT_A_84_P813630	**TUB8**	**-0.77**	**0.0259**
GT_A_84_P11456	**AT1G69710**	**-0.81**	**0.0263**
GT_At_Specific_00080749	**AT2G01023.1**	**-1.29**	**0.0264**
GT_A_84_P767112	**MTHSC70-2**	**-1.40**	**0.0267**
GT_A_84_P232289	**AT5G09570**	**-1.44**	**0.0267**
GT_A_84_P530528	**AT2G42720**	**-1.16**	**0.0270**
GT_A_84_P23956	**AT1G07350**	**-1.36**	**0.0271**

GT_A_84_P792159	**AT5G53710**	**-1.07**	**0.0273**
GT_A_84_P17498	**AT3G52620**	**-0.75**	**0.0273**
GT_A_84_P575158	**AT4G30150**	**-0.85**	**0.0274**
GT_A_84_P805904	**EXL4**	**-1.04**	**0.0274**
GT_A_84_P725162	**AT1G47271**	**-1.07**	**0.0276**
GT_A_84_P757840	**AT2G05995**	**-1.63**	**0.0277**
GT_A_84_P790159	**AT5G38000**	**-2.76**	**0.0277**
GT_A_84_P89269	**CLE3**	**-1.28**	**0.0279**
GT_A_84_P169313	**ProT3**	**-2.35**	**0.0282**
GT_A_84_P786781	**AT2G35760**	**-0.72**	**0.0283**
GT_A_84_P22865	**NP306485**	**-2.50**	**0.0284**
GT_A_84_P519441	**AT5G49340**	**-2.66**	**0.0285**
GT_A_84_P19352	**MEE38**	**-1.13**	**0.0288**
GT_A_84_P769714	**TC379145**	**-1.72**	**0.0290**
GT_A_84_P268980	**AT4G30670**	**-1.98**	**0.0291**
GT_A_84_P832823	**AT3G57980**	**-0.63**	**0.0291**
GT_A_84_P22178	**AT3G27150**	**-1.11**	**0.0294**
GT_AntiSense_AtMg00850_918	**orf107e**	**-0.71**	**0.0297**
GT_A_84_P701336	**TA42053_3702**	**-0.71**	**0.0298**
GT_A_84_P858221	**AT5G51950**	**-3.75**	**0.0298**
GT_At_Specific_00087484	**AT2G07684.1**	**-0.73**	**0.0300**
GT_A_84_P18342	**A37**	**-1.85**	**0.0302**
GT_A_84_P574538	**AT5G09780**	**-2.57**	**0.0303**
GT_A_84_P811289	**HSP81-2**	**-1.33**	**0.0303**
GT_A_84_P869688	**AGP30**	**-3.15**	**0.0305**
GT_A_84_P19223	**ATRABA2B**	**-1.42**	**0.0306**
GT_A_84_P755081	**CSY3**	**-1.19**	**0.0306**
GT_A_84_P803542	**AT4G32208**	**-1.35**	**0.0309**
GT_A_84_P786570	**ATPSK2**	**-0.87**	**0.0310**
GT_A_84_P13138	**AT5G59240**	**-1.49**	**0.0311**
GT_A_84_P16547	**KNAT7**	**-0.76**	**0.0321**
GT_A_84_P842322	**AT5G50100**	**-0.67**	**0.0323**
GT_A_84_P18545	**WRKY65**	**-0.98**	**0.0324**
GT_AntiSense_AtMg00840_913	**orf121b**	**-1.44**	**0.0326**
GT_A_84_P16418	**AT3G09440**	**-1.67**	**0.0328**
GT_A_84_P15450	**HSP70T-2**	**-2.75**	**0.0329**
GT_A_84_P18464	**AT3G56620**	**-1.06**	**0.0332**
GT_A_84_P77119	**EXL1**	**-1.60**	**0.0338**
GT_A_84_P800386	**AT2G07806**	**-0.64**	**0.0338**
GT_A_84_P525034	**AT1G62975**	**-1.35**	**0.0340**
GT_A_84_P23288	**AT4G22810**	**-1.85**	**0.0344**
GT_A_84_P10441	**RCI3**	**-1.47**	**0.0344**
GT_A_84_P13435	**SUS6**	**-1.08**	**0.0344**
GT_A_84_P806774	**AT3G09440**	**-1.67**	**0.0346**
GT_A_84_P759774	**AT3G09915**	**-1.36**	**0.0350**
GT_A_84_P735307	**TA44870_3702**	**-0.60**	**0.0350**
GT_A_84_P98636	**AT2G25670**	**-0.73**	**0.0354**

GT_A_84_P17734	**LBD4**	**-0.65**	**0.0355**
GT_A_84_P855542	**AT5G38100**	**-2.02**	**0.0361**
GT_At_Specific_00205533	**AT4G09731.1**	**-0.91**	**0.0367**
GT_AtMg00890_938	**orf106d**	**-0.76**	**0.0372**
GT_A_84_P22781	**AT1G49390**	**-1.13**	**0.0376**
GT_A_84_P835360	**AT1G15290**	**-0.93**	**0.0377**
GT_A_84_P808919	**PIP1B**	**-1.12**	**0.0382**
GT_A_84_P815994	**AT1G54020**	**-1.21**	**0.0383**
GT_A_84_P852782	**TC379107**	**-1.26**	**0.0385**
GT_A_84_P15773	**CPN10**	**-0.76**	**0.0386**
GT_AtMg00850_918	**orf107e**	**-1.22**	**0.0389**
GT_A_84_P532079	**5PTASE11**	**-1.55**	**0.0389**
GT_A_84_P581530	**AT5G36662**	**-1.56**	**0.0389**
GT_A_84_P71544	**AT1G55380**	**-0.97**	**0.0389**
GT_A_84_P13220	**AT1G15420**	**-0.75**	**0.0391**
GT_At_Specific_00314741	**ORF120**	**-1.39**	**0.0394**
GT_A_84_P767553	**AT5G37940**	**-3.41**	**0.0394**
GT_A_84_P868622	**AT3G56600**	**-1.65**	**0.0395**
GT_A_84_P761249	**LCR44**	**-0.83**	**0.0395**
GT_A_84_P550623	**AT1G15190**	**-1.23**	**0.0395**
GT_A_84_P803761	**AT2G07684**	**-0.80**	**0.0395**
GT_A_84_P17436	**ATBZIP4**	**-2.94**	**0.0396**
GT_A_84_P847144		**-1.73**	**0.0396**
GT_A_84_P866949	**HSP81-3**	**-1.02**	**0.0397**
GT_A_84_P204138	**AT1G66080**	**-2.14**	**0.0403**
GT_A_84_P563885	**AT1G08300**	**-0.98**	**0.0404**
GT_A_84_P728641	**UXS1**	**-0.71**	**0.0405**
GT_A_84_P18904	**AT1G18960**	**-2.20**	**0.0406**
GT_A_84_P755951	**TC391294**	**-0.94**	**0.0407**
GT_A_84_P106762	**AT3G25930**	**-2.56**	**0.0411**
GT_A_84_P22203	**TC370048**	**-1.45**	**0.0412**
GT_A_84_P826815	**SHN2**	**-1.44**	**0.0415**
GT_A_84_P860740	**TC369417**	**-1.48**	**0.0416**
GT_A_84_P515793	**AT5G03890**	**-1.34**	**0.0419**
GT_A_84_P789549	**AT4G31110**	**-1.66**	**0.0419**
GT_A_84_P19151	**AT2G39820**	**-0.98**	**0.0419**
GT_A_84_P788534	**AT2G16210**	**-2.22**	**0.0421**
GT_A_84_P807555	**GAMMA-TIP**	**-1.42**	**0.0425**
GT_A_84_P836732	**MSL5**	**-1.06**	**0.0429**
GT_At_Specific_00084627	**AT2G04395.1**	**-2.23**	**0.0430**
GT_A_84_P799545	**AT2G30540**	**-1.26**	**0.0430**
GT_A_84_P754657	**AT1G16022**	**-1.01**	**0.0430**
GT_A_84_P18401	**HSP70**	**-3.23**	**0.0432**
GT_A_84_P831180	**AT1G77680**	**-1.02**	**0.0433**
GT_A_84_P761578	**AT3G06995**	**-0.92**	**0.0437**
GT_A_84_P22325	**AT4G18450**	**-0.89**	**0.0438**
GT_A_84_P554019	**anac097**	**-0.80**	**0.0439**

GT_A_84_P17452	AT3G14780	-1.71	0.0443
GT_A_84_P23591	AT5G20550	-1.04	0.0443
GT_A_84_P526945	HB-3	-2.45	0.0445
GT_A_84_P23712	AT1G59860	-3.57	0.0447
GT_A_84_P23843	AT2G03200	-1.15	0.0448
GT_A_84_P19224	PAP7	-1.66	0.0451
GT_A_84_P17828	AT5G48570	-3.15	0.0451
GT_A_84_P54670	AT2G35760	-0.81	0.0451
GT_A_84_P12094	AT5G24410	-1.27	0.0451
GT_A_84_P104666	AT5G53390	-0.86	0.0453
GT_A_84_P199584	AT3G14200	-1.40	0.0453
GT_A_84_P14071	NAS2	-3.25	0.0454
GT_At_Specific_00063584	AT1G66080.1	-2.09	0.0455
GT_A_84_P768248	AT5G23035	-1.38	0.0459
GT_A_84_P23356	ATGSTU14	-1.93	0.0461
GT_A_84_P846878	AT3G59020	-0.69	0.0463
GT_A_84_P20080	PIP2B	-1.10	0.0466
GT_A_84_P569083	AT5G46440	-0.91	0.0466
GT_A_84_P857058	TC397723	-0.74	0.0467
GT_A_84_P740606	TA26389_3702	-0.80	0.0469
GT_A_84_P813677	AT5G66390	-2.52	0.0471
GT_A_84_P10171	MES5	-1.00	0.0473
GT_A_84_P531892	RANBP1	-0.67	0.0475
GT_A_84_P173301	AT5G49350	-0.80	0.0476
GT_A_84_P839463	AT5G37478	-1.53	0.0476
GT_A_84_P119212	AT1G14060	-0.90	0.0477
GT_A_84_P15059	ROPGEF11	-0.95	0.0480
GT_A_84_P836769	AT1G15290	-0.95	0.0486
GT_A_84_P531386	AT2G34910	-2.00	0.0487
GT_A_84_P839493	TC372736	-1.03	0.0489
GT_A_84_P257500	ENDO3	-2.66	0.0489
GT_A_84_P211708	AT5G41620	-0.84	0.0490
GT_A_84_P22608	PPAN	-0.65	0.0491
GT_A_84_P22667	COPT3	-2.48	0.0493
GT_A_84_P549255	AT3G28600	-2.49	0.0494
GT_A_84_P724266		-1.18	0.0494
GT_A_84_P803124		-1.16	0.0496
GT_A_84_P14538	AT3G04570	-1.63	0.0499
GT_A_84_P756985	RNEE/G	-1.39	0.0504
GT_A_84_P19732	AT5G51950	-3.81	0.0506
GT_A_84_P853245	PIP2A	-0.74	0.0507
GT_A_84_P22274	AT-HSFA7B	-3.20	0.0507
GT_At_Specific_00088253	AT2G07782.1	-0.79	0.0508
GT_A_84_P19198	AT2G18720	-0.80	0.0510
GT_A_84_P767969	TT16	-1.69	0.0511
GT_A_84_P23707	APS2	-1.29	0.0513
GT_A_84_P801042	PIP1B	-0.83	0.0519

GT_A_84_P11120	APT5	-0.75	0.0520
GT_A_84_P13749	ASK5	-1.86	0.0521
GT_A_84_P856356	ATPHB3	-0.85	0.0523
GT_A_84_P581880	AT1G78120	-2.01	0.0524
GT_A_84_P277520	AT2G37990	-0.76	0.0528
GT_A_84_P21371	AT4G12530	-1.28	0.0533
GT_A_84_P189894	AT5G22660	-1.81	0.0533
GT_A_84_P756371	AT2G07672	-0.94	0.0534
GT_A_84_P11767	NP301364	-0.74	0.0537
GT_A_84_P15993	CYP94B1	-1.95	0.0537
GT_A_84_P248695	AT5G12270	-2.26	0.0544
GT_A_84_P845009	TC391407	-1.06	0.0545
GT_A_84_P13908	AT4G15460	-1.69	0.0547
GT_A_84_P860086	TC399981	-0.79	0.0549
GT_A_84_P791120	AT3G25240	-2.42	0.0550
GT_A_84_P18313	CYP77A6	-0.91	0.0551
GT_A_84_P19214	MAPKKK17	-0.82	0.0551
GT_A_84_P158205	AT1G22470	-1.24	0.0552
GT_A_84_P269630	AT2G27505	-2.34	0.0552
GT_A_84_P801380	HCD1	-0.61	0.0556
GT_A_84_P839984	AT1G63580	-1.27	0.0559
GT_A_84_P835481	XIK	-0.73	0.0559
GT_A_84_P556490	AT4G23493	-2.30	0.0559
GT_At_Specific_00254373	HSP17.6II	-3.69	0.0561
GT_A_84_P17025	ALDH2B7	-1.10	0.0565
GT_A_84_P790752	AT4G03380	-2.28	0.0566
GT_A_84_P811621	TA29115_3702	-1.31	0.0566
GT_A_84_P868217	MBP2	-1.17	0.0567
GT_A_84_P15465	AtMYB57	-1.35	0.0568
GT_A_84_P10555	AT1G53540	-3.94	0.0571
GT_A_84_P18618	ANAC076	-0.75	0.0573
GT_A_84_P17695	AT4G16790	-0.68	0.0575
GT_A_84_P843745	NP457133	-1.55	0.0578
GT_A_84_P804286	EG487081	-1.23	0.0578
GT_A_84_P14290	EDA25	-0.64	0.0582
GT_A_84_P11614	ATASE1	-1.29	0.0582
GT_A_84_P201138	AT2G32470	-2.84	0.0585
GT_A_84_P788013	AT2G42720	-1.35	0.0586
GT_AtMg00260_662	orf101a	-1.02	0.0589
GT_A_84_P271340	AT3G09350	-2.11	0.0591
GT_A_84_P12005	AT4G38410	-1.30	0.0591
GT_A_84_P11227	AT5G53400	-0.66	0.0592
GT_A_84_P13883	AT4G34850	-4.06	0.0594
GT_A_84_P11769	HSP60	-1.03	0.0595
GT_A_84_P62060	AT1G65030	-0.83	0.0598
GT_A_84_P848730	TC396102	-0.87	0.0600
GT_A_84_P20417	AT4G10620	-0.65	0.0602

GT_A_84_P16621	AT4G05170	-1.79	0.0609
GT_A_84_P18289	DAD1	-2.44	0.0609
GT_A_84_P16874	AT5G46530	-0.70	0.0609
GT_A_84_P11789	AT3G43250	-1.70	0.0610
GT_A_84_P12394	AT1G14960	-1.97	0.0612
GT_A_84_P262640	AT5G22430	-4.70	0.0613
GT_A_84_P851645	CESA1	-0.73	0.0614
GT_A_84_P596602	AT4G39300	-0.67	0.0620
GT_A_84_P14033	BGLU21	-2.64	0.0622
GT_A_84_P538729	AT4G10290	-3.37	0.0624
GT_A_84_P837201	ASL9	-0.81	0.0632
GT_A_84_P565014	AT2G25770	-2.42	0.0636
GT_A_84_P263710	AT3G27960	-1.28	0.0637
GT_A_84_P844006	AT2G27505	-3.21	0.0648
GT_A_84_P821119	AT3G09350	-2.09	0.0649
GT_A_84_P553260	AT1G13160	-0.74	0.0650
GT_A_84_P581169	AT3G11000	-1.66	0.0654
GT_A_84_P75644	AT2G05440	-1.49	0.0654
GT_A_84_P97266	CLE2	-1.99	0.0659
GT_A_84_P14397	XIF	-1.05	0.0662
GT_A_84_P809155	TA27913_3702	-1.07	0.0668
GT_A_84_P546443	AT2G28085	-1.36	0.0668
GT_A_84_P240515	LSU2	-0.93	0.0670
GT_A_84_P13014	AT5G12110	-2.70	0.0673
GT_A_84_P13472	AT2G45430	-1.57	0.0676
GT_A_84_P523311	TA26574_3702	-0.98	0.0678
GT_A_84_P585587	AT2G39795	-0.68	0.0681
GT_A_84_P736168	AT2G07715	-0.67	0.0682
GT_A_84_P760723	AY299259	-1.36	0.0682
GT_A_84_P786552	AT2G20870	-2.81	0.0683
GT_A_84_P14936	AT5G26220	-1.58	0.0684
GT_A_84_P11920	AT4G13860	-0.98	0.0684
GT_A_84_P12504	UGT71C1	-1.65	0.0685
GT_A_84_P770152	ATMG01350	-1.03	0.0686
GT_A_84_P757143	AT2G36355	-0.96	0.0688
GT_A_84_P573880	AT1G79150	-1.52	0.0691
GT_A_84_P155845	AT2G47950	-1.67	0.0692
GT_A_84_P833758	AT4G13860	-1.27	0.0694
GT_A_84_P22944	SCPL43	-2.32	0.0702
GT_A_84_P840627	NP221616	-1.08	0.0703
GT_A_84_P16133	AtMC7	-1.38	0.0705
GT_A_84_P20653	AT5G45220	-1.77	0.0706
GT_A_84_P551884	AT3G23180	-1.11	0.0707
GT_A_84_P15644	TC365304	-0.78	0.0709
GT_A_84_P825094	TA40791_3702	-1.25	0.0710
GT_A_84_P258360	AT5G26290	-1.44	0.0712
GT_A_84_P10863	CYP94B3	-1.55	0.0713

GT_A_84_P22038	CPuORF5	-1.05	0.0718
GT_A_84_P850157	TA48759_3702	-1.23	0.0719
GT_At_Specific_00246086	C2H2	-1.10	0.0720
GT_A_84_P23544	HSP18.2	-1.81	0.0720
GT_A_84_P809007	TA27833_3702	-1.74	0.0721
GT_A_84_P12424	CYP703A2	-5.87	0.0728
GT_A_84_P826247	AG	-3.32	0.0731
GT_A_84_P760599	AT3G55290	-0.70	0.0732
GT_A_84_P20918	AT1G73780	-1.80	0.0735
GT_At_Specific_00214329	AT4G16550.1	-1.25	0.0738
GT_A_84_P17319	AT1G67670	-0.83	0.0738
GT_A_84_P198244	LBD16	-1.36	0.0739
GT_A_84_P184234	AT3G23090	-1.08	0.0739
GT_A_84_P12164	AT5G51030	-0.99	0.0740
GT_A_84_P557530	AT5G37478	-1.75	0.0746
GT_A_84_P18129	AtGH9B5	-0.98	0.0751
GT_A_84_P20664	THAS1	-1.47	0.0752
GT_A_84_P597276	AT3G25240	-2.29	0.0752
GT_AtMg00870_925	orf184	-0.94	0.0755
GT_A_84_P547377	AT2G43110	-0.95	0.0755
GT_A_84_P818272	AT2G32240	-0.94	0.0760
GT_AtMg01350_1165	orf145c	-0.83	0.0761
GT_A_84_P180164	AT5G47830	-0.95	0.0761
GT_A_84_P293774	VTC5	-1.22	0.0765
GT_A_84_P815065	TA31344_3702	-1.36	0.0765
GT_A_84_P120342	NP502678	-0.85	0.0769
GT_A_84_P18104	AT1G74500	-2.03	0.0774
GT_A_84_P808926	PIP1B	-0.65	0.0777
GT_A_84_P770402	AT2G07798	-1.07	0.0783
GT_At_Specific_00163546	ATPHB4	-0.79	0.0783
GT_A_84_P17637	AT4G28140	-1.99	0.0783
GT_A_84_P756441	AT2G07769	-1.10	0.0784
GT_A_84_P817973	AtbZIP44	-0.65	0.0785
GT_A_84_P803103	AT2G07691	-0.92	0.0786
GT_A_84_P12086	AT5G19100	-1.72	0.0792
GT_A_84_P784743	AT5G62140	-1.64	0.0792
GT_A_84_P10354	PGP13	-1.74	0.0794
GT_A_84_P10179	MYB46	-0.77	0.0795
GT_A_84_P823308	TA38646_3702	-0.89	0.0796
GT_A_84_P12958	MEE48	-6.61	0.0799
GT_A_84_P580134	AT2G16210	-4.00	0.0799
GT_A_84_P22729	AT1G09880	-1.04	0.0800
GT_A_84_P21617	CPHSC70-2EAT_SH	-1.04	0.0801
GT_A_84_P16680	XTR9	-1.79	0.0801
GT_A_84_P14478	MES3	-0.73	0.0803
GT_A_84_P10374	ATSBT4.12	-1.51	0.0804
GT_A_84_P23094	AT3G12860	-1.04	0.0805

GT_A_84_P284960	**NOP10**	**-0.71**	**0.0806**
GT_A_84_P156185	**ATTPS-CIN**	**-1.97**	**0.0807**
GT_A_84_P524680	**JAZ10**	**-1.37**	**0.0810**
GT_A_84_P22612	**AT5G62730**	**-1.71**	**0.0817**
GT_A_84_P757402	**LCR76**	**-1.21**	**0.0824**
GT_A_84_P15968	**CER3**	**-1.26**	**0.0830**
GT_A_84_P162303	**AT2G15000**	**-1.10**	**0.0833**
GT_A_84_P18380	**AT1G12950**	**-2.54**	**0.0836**
GT_A_84_P522226	**IAA33**	**-2.65**	**0.0837**
GT_A_84_P122882	**AT2G32430**	**-0.96**	**0.0838**
GT_A_84_P834486	**AT4G02660**	**-0.83**	**0.0840**
GT_A_84_P10249	**AT5G44630**	**-2.02**	**0.0842**
GT_A_84_P805353	**TA25953_3702**	**-0.80**	**0.0844**
GT_A_84_P511249	**AT4G14730**	**-1.79**	**0.0844**
GT_A_84_P15447	**AT2G25980**	**-1.68**	**0.0848**
GT_A_84_P531425	**BAG6**	**-2.81**	**0.0850**
GT_A_84_P19611	**AT5G05900**	**-1.68**	**0.0852**
GT_A_84_P765827	**AT4G37409**	**-1.66**	**0.0854**
GT_A_84_P10009	**AT4G08870**	**-1.53**	**0.0861**
GT_A_84_P19321	**AT3G13220**	**-3.86**	**0.0862**
GT_A_84_P12049	**AT5G06720**	**-1.85**	**0.0864**
GT_A_84_P18471	**AT3G58660**	**-0.92**	**0.0869**
GT_A_84_P795693	**AK222207**	**-1.57**	**0.0878**
GT_A_84_P262090	**ATPIP5K1**	**-0.94**	**0.0880**
GT_A_84_P195674	**AT2G20870**	**-0.97**	**0.0881**
GT_A_84_P821420	**TA36674_3702**	**-0.84**	**0.0881**
GT_A_84_P21852	**ATGSTU22**	**-1.50**	**0.0885**
GT_A_84_P594516	**AT2G46740**	**-1.97**	**0.0885**
GT_A_84_P19644	**GAD**	**-0.99**	**0.0886**
GT_A_84_P518042	**AT2G20700**	**-0.89**	**0.0888**
GT_A_84_P18032	**AT1G54050**	**-3.86**	**0.0890**
GT_A_84_P793960	**AT1G08920**	**-1.10**	**0.0893**
GT_A_84_P245905	**AT3G13230**	**-1.06**	**0.0894**
GT_A_84_P20579	**HSP17.6II**	**-3.87**	**0.0896**
GT_A_84_P21636	**QRT1**	**-0.72**	**0.0897**
GT_A_84_P10418	**AT1G51440**	**-0.97**	**0.0898**
GT_A_84_P12457	**ST4B**	**-3.12**	**0.0901**
GT_At_Specific_00147526	**AT3G14200.1**	**-1.38**	**0.0902**
GT_A_84_P17460	**ATA1**	**-5.11**	**0.0902**
GT_A_84_P16397	**AT2G44860**	**-0.73**	**0.0906**
GT_A_84_P12747	**AT3G46700**	**-0.69**	**0.0910**
GT_A_84_P302500	**AT1G12540**	**-1.61**	**0.0910**
GT_A_84_P22829	**ENDO_2**	**-2.56**	**0.0912**
GT_A_84_P768159	**LCR6**	**-0.79**	**0.0914**
GT_AtMg01220_1103	**orf113**	**-1.06**	**0.0914**
GT_At_Specific_00050642	**AT1G53542.1**	**-1.08**	**0.0915**
GT_A_84_P567982	**BAM5**	**-2.91**	**0.0918**

GT_A_84_P19534	PRF4	-1.86	0.0919
GT_A_84_P11951	AT4G25340	-1.20	0.0926
GT_A_84_P21229	AT3G21420	-0.75	0.0929
GT_A_84_P232489	AT4G04690	-0.99	0.0934
GT_A_84_P11731	MBF1C	-2.24	0.0934
GT_A_84_P808952	PIP1B	-0.63	0.0938
GT_A_84_P860230	TIP2;2	-1.60	0.0940
GT_A_84_P565635	AT5G41030	-0.82	0.0942
GT_A_84_P11961	RD26	-0.64	0.0945
GT_A_84_P807598	AT5G22430	-4.14	0.0950
GT_A_84_P830125	BAG6	-3.11	0.0951
GT_A_84_P605975	AT2G04800	-1.15	0.0958
GT_A_84_P14618	AT3G11964	-0.80	0.0959
GT_A_84_P15536	JR1	-0.91	0.0964
GT_A_84_P19176	AGO5	-2.79	0.0965
GT_A_84_P754928	AT2G40850	-1.05	0.0968
GT_A_84_P53140	AT1G02870	-0.70	0.0970
GT_A_84_P22420	AT4G15390	-1.54	0.0971
GT_A_84_P115042	AT4G13330	-0.80	0.0971
GT_A_84_P853336	AT5G27120	-0.89	0.0971
GT_A_84_P10202	AT5G24070	-0.95	0.0977
GT_A_84_P10415	MBP2	-1.13	0.0977
GT_A_84_P117762	AT3G03520	-1.15	0.0977
GT_A_84_P13524	MLO15	-1.17	0.0979
GT_A_84_P821272	TA36487_3702	-2.74	0.0984
GT_A_84_P155935	AT4G27590	-1.59	0.0993
GT_A_84_P17327	AT2G34210	-2.85	0.0999
GT_A_84_P856041	AT1G80620	-0.72	0.1002
GT_A_84_P507007	AT5G41590	-1.45	0.1006
GT_A_84_P12343	ATRRP4	-0.98	0.1007
GT_A_84_P10235	AT5G40510	-0.72	0.1009
GT_A_84_P18699	AtMYB56	-1.13	0.1009
GT_A_84_P14308	ATRPAC42	-0.86	0.1014
GT_A_84_P10273	NBP35	-0.62	0.1016
GT_A_84_P770076	NP1098890	-1.34	0.1020
GT_A_84_P20085	AT2G35270	-2.06	0.1024
GT_A_84_P766361	RPL5B	-0.95	0.1026
GT_A_84_P753716	AT1G35255	-1.26	0.1026
GT_A_84_P815058	NF-YC2	-1.32	0.1028
GT_A_84_P10911	AT3G59510	-1.90	0.1030
GT_A_84_P21473	BAM5	-2.76	0.1035
GT_A_84_P823221	JAZ10	-1.19	0.1037
GT_A_84_P21525	AT-HSP17.6A	-3.79	0.1038
GT_A_84_P259610	AT3G01240	-2.65	0.1039
GT_A_84_P224479	AT5G16650	-0.66	0.1050
GT_A_84_P562243	AT1G62350	-0.81	0.1054
GT_A_84_P562958	AT3G06220	-0.69	0.1055

GT_A_84_P22388	AT4G33070	-1.67	0.1055
GT_A_84_P22327	AG	-3.12	0.1061
GT_A_84_P12615	AT2G32240	-0.95	0.1068
GT_A_84_P544120	AT4G32860	-1.63	0.1073
GT_A_84_P212648	NP225571	-0.70	0.1073
GT_A_84_P13156	AT5G63120	-0.70	0.1078
GT_A_84_P15932	TIP2;3	-2.28	0.1079
GT_A_84_P20779	AT2G07714	-0.76	0.1082
GT_A_84_P12617	ANAC038	-1.93	0.1086
GT_A_84_P819143	CPHSC70-2EAT_SH	-1.04	0.1096
GT_A_84_P271080	AT3G56600	-1.29	0.1096
GT_A_84_P19319	AT3G15080	-0.66	0.1097
GT_A_84_P14475	AT1G30080	-1.48	0.1098
GT_A_84_P18574	AT4G26220	-0.72	0.1103
GT_A_84_P20004	AT1G72660	-1.63	0.1104
GT_A_84_P860053	TA31325_3702	-1.19	0.1104
GT_At_Specific_00132469	AtMYB57	-0.71	0.1108
GT_A_84_P755497	AK227826	-0.99	0.1120
GT_A_84_P10861	ATATH7	-1.11	0.1121
GT_A_84_P525040	AT1G65342	-2.57	0.1122
GT_A_84_P856185	AT3G25930	-1.72	0.1124
GT_A_84_P258170	AT5G53700	-1.95	0.1129
GT_A_84_P15735	AT4G26010	-2.17	0.1130
GT_A_84_P225169	AT3G23620	-0.76	0.1133
GT_A_84_P816904	TIP2;3	-1.65	0.1133
GT_A_84_P574551	LAS	-2.32	0.1136
GT_A_84_P837280	MES7	-1.05	0.1144
GT_A_84_P14465	PHT4;2	-0.72	0.1146
GT_A_84_P500838	PHOT2	-1.00	0.1149
GT_A_84_P765493	AT4G14226	-1.15	0.1156
GT_A_84_P763091	AT4G08870	-1.13	0.1157
GT_A_84_P846960	TC383337	-1.15	0.1159
GT_A_84_P821571	AT1G80270	-0.86	0.1166
GT_A_84_P551820	RPSAb	-0.89	0.1169
GT_A_84_P126091	LACS2	-1.16	0.1170
GT_A_84_P309133	AT5G18065	-1.60	0.1175
GT_A_84_P825746	HSA32	-2.25	0.1176
GT_A_84_P11904	STK	-2.20	0.1177
GT_A_84_P13580	AT3G03400	-4.31	0.1181
GT_A_84_P572089	AT1G78990	-2.28	0.1182
GT_A_84_P92549	AT1G27470	-0.72	0.1183
GT_A_84_P784586	ATTPS-CIN	-2.16	0.1187
GT_A_84_P180414	AT5G55970	-1.10	0.1193
GT_A_84_P19357	XTR8	-1.02	0.1194
GT_A_84_P23649	AT1G31050	-1.56	0.1198
GT_At_Specific_00002987	ATNAF1	-0.91	0.1202
GT_A_84_P802924	EG495729	-1.44	0.1202

GT_A_84_P19231	ATHSFA2	-3.16	0.1204
GT_A_84_P19749	EIR1	-1.45	0.1207
GT_A_84_P562234	ZCW32	-1.07	0.1210
GT_A_84_P792897	MEE49	-0.83	0.1211
GT_A_84_P10902	NAP57	-0.88	0.1222
GT_At_Specific_00137950	AT3G06019.1	-0.65	0.1225
GT_A_84_P20921	AT1G77520	-2.36	0.1225
GT_A_84_P23614	AT2G07738	-0.91	0.1228
GT_A_84_P15739	AR192	-1.16	0.1232
GT_A_84_P769660	AT5G48240	-0.73	0.1233
GT_A_84_P23724	ATGSTU25	-1.46	0.1234
GT_A_84_P15530	APUM24	-0.83	0.1236
GT_A_84_P22411	AT4G39130	-1.07	0.1238
GT_A_84_P828509	SUS4	-1.70	0.1239
GT_A_84_P211908	AT5G05500	-1.78	0.1241
GT_A_84_P784216	VSP1	-1.59	0.1243
GT_A_84_P13893	AGL21	-3.34	0.1244
GT_A_84_P21428	PLP8	-0.90	0.1247
GT_A_84_P784282	AT4G16630	-0.83	0.1248
GT_A_84_P597426	HSA32	-2.15	0.1252
GT_A_84_P20389	AT4G01026	-0.82	0.1255
GT_A_84_P11772	AT3G27970	-1.32	0.1259
GT_At_Specific_00157890	AT3G22660.1	-0.82	0.1259
GT_A_84_P12324	AT1G70130	-2.34	0.1269
GT_A_84_P20786	AGL12	-1.33	0.1273
GT_A_84_P573251	AT3G47200	-1.84	0.1274
GT_A_84_P13187	MGD2	-0.78	0.1274
GT_A_84_P17310	AT2G30830	-2.77	0.1277
GT_At_Specific_00234796	CER4	-2.55	0.1277
GT_A_84_P12513	MIPS2	-1.35	0.1277
GT_A_84_P19513	AT4G24830	-0.84	0.1278
GT_A_84_P576786	AT3G43580	-1.05	0.1280
GT_A_84_P825740	RPSAb	-0.88	0.1280
GT_A_84_P808192	MLP34	-1.95	0.1281
GT_A_84_P813787	LACS2	-0.97	0.1282
GT_A_84_P188424	1-Sep	-3.26	0.1283
GT_A_84_P21647	DDB2	-0.69	0.1284
GT_A_84_P803083		-1.59	0.1285
GT_A_84_P809159	PIP2A	-1.02	0.1287
GT_A_84_P823257	AT4G22235	-1.51	0.1288
GT_AntiSense_AtMg00150_609	orf116	-1.42	0.1301
GT_A_84_P13852	HSP21	-2.30	0.1305
GT_A_84_P20047	AT1G31660	-0.88	0.1310
GT_A_84_P817969	AtbZIP44	-0.76	0.1313
GT_A_84_P225469	AT5G52420	-0.64	0.1314
GT_A_84_P222439	FLA15	-1.59	0.1316
GT_A_84_P830119	BAG6	-2.97	0.1316

GT_A_84_P15816	ATNAP9	-0.91	0.1322
GT_A_84_P17954	AT3G19085	-1.38	0.1322
GT_A_84_P51080	AT1G52930	-0.77	0.1323
GT_A_84_P141219	AT5G35320	-0.80	0.1324
GT_A_84_P588148	AT2G07774	-1.06	0.1325
GT_A_84_P155475	AT4G35060	-0.70	0.1327
GT_A_84_P806316	AtATG18h	-1.79	0.1334
GT_A_84_P22643	SPX1	-1.21	0.1337
GT_A_84_P829837	TA46917_3702	-1.25	0.1341
GT_A_84_P815408	CR88	-1.15	0.1344
GT_A_84_P12160	AT5G49770	-0.70	0.1354
GT_A_84_P803809		-0.91	0.1355
GT_A_84_P591730	ELF4-L3	-1.12	0.1358
GT_A_84_P14991	AT5G48740	-1.11	0.1365
GT_A_84_P512333	AT5G43660	-0.88	0.1371
GT_A_84_P800340	AT3G06435	-0.96	0.1375
GT_A_84_P19553	CER4	-2.94	0.1378
GT_A_84_P13031	AT5G19110	-2.27	0.1379
GT_A_84_P602531	PAP14	-3.89	0.1382
GT_A_84_P13426	CYP96A15	-5.46	0.1394
GT_A_84_P11307	EXL4	-1.17	0.1394
GT_A_84_P24130	AT3G54700	-1.48	0.1397
GT_A_84_P222749	AT4G33800	-1.15	0.1399
GT_A_84_P835531	AT5G64170	-1.35	0.1401
GT_A_84_P768705	AT5G20225	-0.74	0.1401
GT_A_84_P22109	AT3G09720	-0.85	0.1403
GT_A_84_P787196	AT5G46115	-0.98	0.1407
GT_A_84_P251325	AT2G41050	-0.78	0.1413
GT_A_84_P277930	AT2G02680	-1.95	0.1417
GT_A_84_P707595	AT5G52415	-1.98	0.1433
GT_AntiSense_AtCg00640_307	rpl33	-1.07	0.1434
GT_A_84_P722142	AT2G43375	-1.29	0.1434
GT_A_84_P22205	AT1G48460	-0.74	0.1435
GT_A_84_P140739	AT5G51780	-2.41	0.1437
GT_A_84_P24132	TT5	-1.23	0.1439
GT_A_84_P851319	MYB59	-1.31	0.1442
GT_A_84_P159165	AT1G17030	-1.04	0.1446
GT_A_84_P840907	AT5G56880	-0.93	0.1453
GT_A_84_P89559	AT5G66540	-1.01	0.1454
GT_A_84_P223359	AT1G54000	-1.28	0.1457
GT_A_84_P16699	AT4G29930	-1.14	0.1462
GT_A_84_P768088	AT5G43513	-1.01	0.1465
GT_A_84_P177444	MEE49	-0.80	0.1471
GT_A_84_P13039	AT5G24420	-1.96	0.1471
GT_A_84_P10735	AT3G05060	-0.74	0.1475
GT_A_84_P570378	AT2G28690	-0.80	0.1480
GT_A_84_P796552	AK229901	-2.26	0.1481

GT_A_84_P851247	AT5G28237	-2.07	0.1482
GT_A_84_P787940	PAP14	-3.45	0.1488
GT_A_84_P851931	PROT1	-0.90	0.1491
GT_A_84_P16735	AT4G39070	-2.44	0.1493
GT_A_84_P22812	ARR15	-1.96	0.1497
GT_A_84_P12797	AT1G21528	-1.15	0.1498
GT_A_84_P717727	TA35714_3702	-0.81	0.1502
GT_A_84_P507728	SP1L5	-1.29	0.1503
GT_A_84_P19904	ERD9	-1.10	0.1503
GT_A_84_P12103	AT5G27120	-0.70	0.1508
GT_A_84_P757973	AT2G44578	-1.67	0.1509
GT_A_84_P12502	TIP4;1	-1.13	0.1514
GT_At_Specific_00035701	AT1G32385.1	-0.84	0.1515
GT_A_84_P274830	CLE40	-1.88	0.1517
GT_A_84_P13173	MYBR1	-0.84	0.1520
GT_A_84_P14126	AT5G16930	-1.12	0.1530
GT_A_84_P15244	ATLP-3	-1.34	0.1547
GT_AtMg01410_1194	orf204	-0.91	0.1556
GT_A_84_P822993	CYP77A6	-1.02	0.1557
GT_A_84_P511391	AT5G17830	-0.68	0.1562
GT_A_84_P770208	EF182951	-1.00	0.1568
GT_A_84_P567310	AT5G47455	-1.39	0.1573
GT_A_84_P97476	VSP1	-1.80	0.1576
GT_A_84_P601919	AT4G34550	-3.05	0.1580
GT_A_84_P594805	AT4G36820	-0.97	0.1586
GT_A_84_P549263	AT3G32980	-1.00	0.1591
GT_A_84_P18276	AT2G39590	-1.58	0.1597
GT_A_84_P14872	AT1G62720	-1.18	0.1601
GT_A_84_P11439	AT1G52560	-3.35	0.1608
GT_A_84_P19854	AT1G17690	-0.67	0.1608
GT_A_84_P23014	AT2G44581	-1.52	0.1611
GT_A_84_P805621	VSP1	-1.48	0.1616
GT_A_84_P787143	AT5G49560	-1.19	0.1619
GT_A_84_P833596	ALMT1	-1.92	0.1620
GT_A_84_P741115	TA31179_3702	-1.14	0.1621
GT_A_84_P195024	AT4G37090	-0.74	0.1627
GT_A_84_P20298	SUS4	-1.92	0.1629
GT_A_84_P73044	MIZ1	-1.01	0.1630
GT_A_84_P795728	AK221753	-1.28	0.1631
GT_A_84_P16575	AT3G57490	-1.12	0.1634
GT_A_84_P792067	AT3G23450	-1.05	0.1637
GT_A_84_P855960	AT2G35130	-1.06	0.1643
GT_A_84_P153218	AT4G21780	-1.08	0.1643
GT_A_84_P155965	MSL10	-0.87	0.1645
GT_A_84_P760530	AT3G06437	-1.27	0.1652
GT_A_84_P92299	AT1G73010	-2.13	0.1666
GT_A_84_P19489	AT1G29320	-1.13	0.1666

GT_A_84_P59510	AT4G32480	-1.75	0.1670
GT_A_84_P19638	ROXY2	-2.44	0.1678
GT_A_84_P767270	PI	-2.95	0.1688
GT_A_84_P15251	AT1G69500	-3.50	0.1695
GT_A_84_P18393	ATRLI1	-1.18	0.1696
GT_A_84_P541962	AT2G16385	-1.39	0.1697
GT_A_84_P22479	AT5G15180	-1.16	0.1699
GT_A_84_P17365	ATERDJ3A	-2.01	0.1706
GT_A_84_P16027	AT5G21150	-3.16	0.1710
GT_At_Specific_00016967	AT1G15250.1	-0.83	0.1712
GT_A_84_P837979	ASL9	-0.65	0.1713
GT_A_84_P295514	AT3G58550	-1.13	0.1713
GT_A_84_P502808	AT1G48405	-1.11	0.1715
GT_At_Specific_00095692	AT2G18220.1	-1.10	0.1715
GT_A_84_P182564	AT4G21323	-1.90	0.1722
GT_A_84_P605545	AT5G14330	-1.05	0.1726
GT_A_84_P247425	AT4G04990	-1.76	0.1731
GT_A_84_P68154	AT3G22660	-0.90	0.1738
GT_A_84_P178164	MLP34	-1.89	0.1742
GT_A_84_P23778	NRPA2	-1.15	0.1749
GT_A_84_P11982	CYP96A2	-1.05	0.1750
GT_A_84_P833332	AT5G20860	-1.24	0.1755
GT_A_84_P15731	ATHSP23.6-MITO	-2.82	0.1762
GT_A_84_P15686	ATHSP22.0	-4.01	0.1766
GT_At_Specific_00274990	AT5G35320.1	-1.16	0.1766
GT_A_84_P24124	PIP2A	-0.94	0.1771
GT_A_84_P188714	AT3G06390	-0.84	0.1771
GT_A_84_P768827	AT5G11630	-1.04	0.1774
GT_A_84_P22231	AT3G53230	-1.25	0.1782
GT_At_Specific_00312635	EPS1	-0.76	0.1782
GT_A_84_P515906	MSP2	-1.62	0.1782
GT_A_84_P11067	PMEI1	-1.52	0.1783
GT_A_84_P20117	AGL6	-2.82	0.1785
GT_A_84_P831247	AT5G43520	-2.78	0.1790
GT_A_84_P11098	CAT6	-1.60	0.1791
GT_A_84_P751510	TC368609	-1.54	0.1798
GT_A_84_P564273	AT3G17030	-0.85	0.1799
GT_A_84_P757549	RALFL15	-2.33	0.1801
GT_A_84_P293424	AT2G05510	-2.84	0.1802
GT_A_84_P797196	EG440258	-2.85	0.1802
GT_A_84_P260410	AT1G78450	-2.66	0.1809
GT_A_84_P14031	AT5G44920	-0.98	0.1815
GT_A_84_P535673	AT1G61430	-1.67	0.1818
GT_A_84_P15703	AT4G18340	-1.13	0.1822
GT_A_84_P843714	AT2G41670	-0.92	0.1823
GT_At_Specific_00014126	AT1G12870.1	-1.25	0.1825
GT_A_84_P571367	AT3G08490	-1.38	0.1825

GT_A_84_P11430	**AT1G15250**	-0.79	**0.1831**
GT_A_84_P808818	**VSP2**	-1.47	**0.1833**
GT_At_Specific_00109467	**UGT71C2**	-1.56	**0.1859**
GT_A_84_P853962	**AT2G46090**	-0.61	**0.1861**
GT_A_84_P20686	**AT5G54370**	-2.13	**0.1863**
GT_A_84_P529286	**AT2G23160**	-0.85	**0.1866**
GT_A_84_P737484	**ATBAG5**	-1.88	**0.1869**
GT_A_84_P834162	ATGSTF4	-0.68	**0.228**
GT_A_84_P18240	**AT2G19310**	-0.96	**0.1882**
GT_A_84_P822023	**AST91**	-1.04	**0.1883**
GT_A_84_P19137	**WRKY56**	-0.74	**0.1899**
GT_A_84_P860823	**CR88**	-1.08	**0.1901**
GT_A_84_P828241	**TA44692_3702**	-1.06	**0.1910**
GT_A_84_P11140	**AT5G18840**	-1.97	**0.1911**
GT_A_84_P12766	**F3H**	-2.32	**0.1915**
GT_A_84_P12960	**GLP9**	-1.26	**0.1924**
GT_A_84_P12936	**AT4G34440**	-0.98	**0.1928**
GT_A_84_P555590	**AT4G22212**	-2.06	**0.1928**
GT_A_84_P299450	**EMB2777**	-0.96	**0.1933**
GT_A_84_P569216	**AT1G08845**	-0.80	**0.1937**
GT_A_84_P577849	**AT4G33770**	-1.11	**0.1940**
GT_A_84_P803062	**ERD5**	-1.50	**0.1945**
GT_A_84_P176164	**AT2G45300**	-0.62	**0.1945**
GT_A_84_P13106	**AT5G50030**	-1.18	**0.1950**
GT_A_84_P14089	**FLA12**	-0.89	**0.1960**
GT_A_84_P22625	**AT1G52270**	-1.38	**0.1963**
GT_A_84_P807539	**F3H**	-2.22	**0.1963**
GT_A_84_P591926	**AT3G21680**	-1.18	**0.1965**
GT_A_84_P22798	**ATGSTU23**	-1.30	**0.1967**
GT_A_84_P159375	**AT4G38080**	-0.73	**0.1970**
GT_A_84_P14930	**AT5G24820**	-3.49	**0.1975**
GT_A_84_P16751	**TIP2;2**	-1.95	**0.1976**
GT_A_84_P827762	**ZFP6**	-0.85	**0.1976**
GT_A_84_P111682	**AT5G43520**	-3.05	**0.1978**
GT_A_84_P10611	**AT2G43530**	-0.97	**0.1989**
GT_A_84_P550508	**AT5G57570**	-1.17	**0.1992**
GT_A_84_P20750	**AT5G19890**	-2.27	**0.1997**
GT_A_84_P222609	**AT1G24265**	-0.76	**0.2005**
GT_A_84_P10341	**AT5G15550**	-0.67	**0.2009**
GT_A_84_P534840	**AT1G80245**	-0.86	**0.2021**
GT_A_84_P23171	**HSFA7A**	-2.32	**0.2027**
GT_A_84_P52180	**AGL8**	-1.58	**0.2028**
GT_A_84_P835549	**TA52094_3702**	-1.86	**0.2029**
GT_A_84_P18813	**AT5G59490**	-1.25	**0.2029**
GT_A_84_P818265	**AT4G32480**	-1.64	**0.2039**
GT_A_84_P844083	**AT2G07721**	-1.30	**0.2048**
GT_A_84_P830028	**TA47215_3702**	-1.97	**0.2049**
GT_A_84_P724814	**CB254609**	-1.19	**0.2057**

GT_A_84_P502791	AT1G32910	-2.50	0.2063
GT_At_Specific_00281680	anac095	-1.36	0.2079
GT_At_Specific_00294567	AT5G51780.1	-1.92	0.2081
GT_A_84_P21046	AT2G38600	-0.97	0.2089
GT_A_84_P17283	AGL44	-2.78	0.2094
GT_A_84_P17199	AT1G15440	-1.09	0.2102
GT_A_84_P845601	IAA18	-0.79	0.2126
GT_At_Specific_00247911	EIF3G2	-1.22	0.2137
GT_A_84_P171863	AT2G26860	-0.69	0.2153
GT_A_84_P22308	AT4G10390	-1.36	0.2165
GT_At_Specific_00018234	CYP79F2	-1.39	0.2166
GT_A_84_P56650	AT5G41080	-2.71	0.2174
GT_A_84_P16803	AT5G14700	-0.90	0.2188
GT_A_84_P587368	AT2G42140	-1.56	0.2193
GT_A_84_P849448	PIP1;4	-0.86	0.2193
GT_A_84_P94199	AT2G29670	-1.04	0.2193
GT_A_84_P21316	EDA14	-0.77	0.2195
GT_A_84_P17304	AT2G40700	-0.72	0.2196
GT_A_84_P811360	ICL	-0.86	0.2200
GT_A_84_P87139	FUT2	-1.36	0.2208
GT_A_84_P528692	AT2G24030	-0.81	0.2217
GT_At_Specific_00059168	MIR413	-0.76	0.2221
GT_A_84_P849379	TC381400	-1.95	0.2223
GT_A_84_P10253	ERD2	-0.75	0.2228
GT_A_84_P544235	AT5G28237	-2.12	0.2231
GT_A_84_P844790	TC402262	-1.46	0.2252
GT_At_Specific_00291240	ATSDI1	-2.06	0.2256
GT_A_84_P819096	TA34519_3702	-4.12	0.2260
GT_A_84_P20555	AT5G05400	-0.71	0.2268
GT_A_84_P21573	AT5G38120	-3.36	0.2287
GT_A_84_P10932	PIP1;4	-0.73	0.2289
GT_A_84_P172281	ATSDI1	-2.04	0.2291
GT_A_84_P21334	AT4G00620	-0.81	0.2299
GT_A_84_P165553	AT2G18900	-1.26	0.2314
GT_A_84_P821066	AGL8	-1.70	0.2317
GT_A_84_P217278	AT3G22570	-0.73	0.2327
GT_A_84_P10977	ATTRANS11	-1.16	0.2328
GT_A_84_P14250	AT1G03070	-1.24	0.2329
GT_A_84_P762988	MAPKKK21	-2.11	0.2330
GT_A_84_P790164	AT5G41080	-2.74	0.2334
GT_A_84_P23737	Hsp70b	-2.73	0.2338
GT_A_84_P13096	HAT2	-0.69	0.2341
GT_A_84_P23973	AtMS2	-1.58	0.2347
GT_A_84_P597160	AT2G39120	-1.01	0.2364
GT_A_84_P802975	TIP2;2	-2.07	0.2369
GT_A_84_P21250	AT3G45230	-0.91	0.2376
GT_A_84_P540888	AT1G17940	-0.81	0.2376

GT_A_84_P21742	AT1G18990	-2.33	0.2382
GT_A_84_P14072	ATEXPA14	-1.53	0.2382
GT_A_84_P162443	AT5G64510	-2.09	0.2385
GT_A_84_P531612	AT4G04745	-1.21	0.2394
GT_A_84_P753767	AT1G04945	-0.95	0.2400
GT_A_84_P15050	AT5G64100	-2.65	0.2410
GT_A_84_P14205	3-Sep	-2.47	0.2416
GT_A_84_P164843	AT3G27550	-0.85	0.2416
GT_A_84_P219198	MLP329	-1.83	0.2419
GT_A_84_P789498	PPCK1	-1.18	0.2424
GT_A_84_P12303	AT1G03810	-1.17	0.2431
GT_A_84_P14063	AT5G53770	-0.74	0.2431
GT_A_84_P828124	AT1G72360	-0.90	0.2435
GT_A_84_P22521	AT5G38780	-1.39	0.2452
GT_A_84_P590665	AT1G22220	-1.56	0.2457
GT_A_84_P22169	AT3G22530	-0.71	0.2458
GT_A_84_P263290	AT4G25400	-2.96	0.2461
GT_A_84_P20994	IAA18	-0.71	0.2463
GT_A_84_P256030	EDA41	-0.84	0.2469
GT_A_84_P21901	AT1G06830	-1.20	0.2470
GT_A_84_P113602	MEE12	-0.83	0.2472
GT_A_84_P811601	TA29106_3702	-2.56	0.2473
GT_A_84_P270820	ATPUP15	-2.16	0.2473
GT_At_Specific_00300952	AT5G57181.1	-1.12	0.2476
GT_A_84_P17537	AT3G61680	-1.01	0.2487
GT_A_84_P787555	ATMAP70-5	-0.75	0.2497
GT_A_84_P14142	AT5G09480	-1.40	0.2498
GT_A_84_P17379	APX2	-2.50	0.2499
GT_At_Specific_00190113	AT3G59778.1	-0.86	0.2504
GT_A_84_P806273	NPQ4	-1.39	0.2511
GT_A_84_P17787	AT5G37670	-2.21	0.2513
GT_A_84_P827457	TA43605_3702	-2.26	0.2520
GT_A_84_P146119	AT1G78930	-1.22	0.2522
GT_A_84_P768126	LCR3	-0.83	0.2524
GT_A_84_P766364	AT5G31770	-0.77	0.2529
GT_A_84_P287970	PCK2	-1.90	0.2531
GT_A_84_P585407	RXW8	-0.98	0.2544
GT_A_84_P595141	JAZ8	-1.89	0.2552
GT_A_84_P21330	ATCHX4	-0.81	0.2555
GT_A_84_P828333	UGT71C2	-1.65	0.2556
GT_A_84_P22610	AT5G62210	-1.43	0.2602
GT_A_84_P17478	AT3G47660	-1.35	0.2604
GT_A_84_P18331	PPCK2	-2.91	0.2606
GT_A_84_P20199	DGD1	-1.04	0.2607
GT_A_84_P16511	PRMT3	-1.21	0.2613
GT_A_84_P814552	3-Sep	-2.71	0.2615
GT_A_84_P14928	SQP2	-1.13	0.2618

GT_A_84_P815721	**BES1**	**-1.34**	**0.2624**
GT_A_84_P785948	**AGP13**	**-1.18**	**0.2665**
GT_A_84_P848231	**AT1G23740**	**-0.90**	**0.2668**
GT_A_84_P21289	**AT3G53940**	**-1.37**	**0.2673**
GT_A_84_P601889	**AT4G26800**	**-1.42**	**0.2697**
GT_A_84_P12004	**AT4G38150**	**-0.75**	**0.2698**
GT_A_84_P132815	**AT2G47790**	**-0.63**	**0.2699**
GT_A_84_P827866	**AT5G16230**	**-0.80**	**0.2700**
GT_A_84_P758697	**AT2G36355**	**-0.70**	**0.2702**
GT_A_84_P841311	**AT3G21460**	**-2.03**	**0.2712**
GT_At_Specific_00258069	**AT5G15700.1**	**-1.07**	**0.2735**
GT_A_84_P21594	**FLA13**	**-1.57**	**0.2738**
GT_A_84_P191974	**AT5G03210**	**-1.02**	**0.2744**
GT_A_84_P311583	**NP10636245**	**-1.26**	**0.2748**
GT_A_84_P18457	**anac063**	**-1.51**	**0.2748**
GT_A_84_P23916	**AT2G19385**	**-0.93**	**0.2750**
GT_A_84_P558374	**TC365940**	**-1.29**	**0.2753**
GT_A_84_P21203	**AT1G26730**	**-1.32**	**0.2754**
GT_A_84_P550637	**AT1G18830**	**-2.52**	**0.2754**
GT_A_84_P21926	**AT1G78950**	**-1.60**	**0.2766**
GT_AtMg01200_1096	**orf294**	**-1.53**	**0.2774**
GT_A_84_P96326	**AT5G38420**	**-1.29**	**0.2777**
GT_A_84_P754250	**MIR846A**	**-1.91**	**0.2786**
GT_A_84_P834383	**SDG37**	**-0.71**	**0.2791**
GT_A_84_P857762	**VSP2**	**-1.34**	**0.2801**
GT_A_84_P15333	**AT2G19740**	**-0.62**	**0.2801**
GT_A_84_P11721	**ANAC055**	**-1.75**	**0.2813**
GT_A_84_P764169	**AT4G02950**	**-0.93**	**0.2821**
GT_A_84_P24070	**CLC-B**	**-2.12**	**0.2822**
GT_A_84_P124342	**AT5G59080**	**-1.10**	**0.2825**
GT_A_84_P111332	**AT1G56210**	**-0.84**	**0.2830**
GT_A_84_P23343	**AT4G35110**	**-0.76**	**0.2831**
GT_A_84_P797619	**PORA**	**-1.47**	**0.2841**
GT_A_84_P13975	**BUD2**	**-1.04**	**0.2851**
GT_A_84_P12990	**GT_A_84_P12990_**	**-0.97**	**0.2859**
GT_A_84_P799948	**DQ108843**	**-2.00**	**0.2867**
GT_A_84_P537901	**AT4G28410**	**-1.11**	**0.2871**
GT_A_84_P22009	**AGL20**	**-0.96**	**0.2872**
GT_A_84_P837084	**AT1G58280**	**-1.35**	**0.2885**
GT_A_84_P11230	**PORA**	**-1.56**	**0.2886**
GT_A_84_P557599	**AT5G57640**	**-1.59**	**0.2897**
GT_A_84_P814996	**TA31293_3702**	**-0.86**	**0.2899**
GT_A_84_P830088	**AT2G03240**	**-0.91**	**0.2899**
GT_A_84_P857510	**AT3G11930**	**-1.05**	**0.2901**
GT_A_84_P10860	**AT3G47580**	**-0.76**	**0.2901**
GT_A_84_P861938	**TA27368_3702**	**-0.93**	**0.2913**
GT_A_84_P805421	**AT5G38420**	**-1.30**	**0.2913**

GT_A_84_P12090	**CYP86B1**	**-0.93**	**0.2950**
GT_A_84_P10561	**ILL5**	**-0.61**	**0.2963**
GT_At_Specific_00141817	**AT3G09720.1**	**-0.89**	**0.2982**
GT_A_84_P791955	**BX828813**	**-0.66**	**0.2982**
GT_A_84_P12058	**FLS**	**-1.84**	**0.2994**
GT_A_84_P16525	**AT3G46210**	**-0.62**	**0.3026**
GT_A_84_P253585	**AT3G26410**	**-0.63**	**0.3028**
GT_A_84_P839669	**AT1G08300**	**-1.11**	**0.3032**
GT_A_84_P750651	**AT1G59930**	**-1.30**	**0.3035**
GT_A_84_P851101	**AT1G03730**	**-0.71**	**0.3038**
GT_A_84_P830890	**AT4G33770**	**-1.08**	**0.3043**
GT_A_84_P21871	**AT1G66540**	**-1.18**	**0.3057**
GT_A_84_P19845	**ATMYB21**	**-2.57**	**0.3058**
GT_AntiSense_AtMg00990_995	**nad3**	**-1.10**	**0.3066**
GT_A_84_P12724	**AT3G24480**	**-0.76**	**0.3085**
GT_A_84_P809112	**MT1C**	**-0.66**	**0.3090**
GT_A_84_P191914	**AT2G21320**	**-1.79**	**0.3092**
GT_A_84_P18229	**ATC**	**-1.36**	**0.3121**
GT_A_84_P533782	**GT_A_84_P533782**	**-1.41**	**0.3124**
GT_A_84_P14848	**XTR7**	**-1.60**	**0.3159**
GT_A_84_P21746	**AT1G17710**	**-2.21**	**0.3163**
GT_A_84_P12727	**NIP7;1**	**-0.82**	**0.3172**
GT_A_84_P18952	**AT1G09460**	**-1.35**	**0.3179**
GT_A_84_P815126	**TA31395_3702**	**-2.09**	**0.3206**
GT_A_84_P824370	**AT5G59080**	**-1.23**	**0.3214**
GT_A_84_P831446	**AT5G17780**	**-2.57**	**0.3223**
GT_A_84_P19960	**NCED9**	**-1.40**	**0.3227**
GT_A_84_P13475	**MGDC**	**-1.97**	**0.3240**
GT_A_84_P849510	**RHM1**	**-0.91**	**0.3258**
GT_A_84_P21894	**AT1G68740**	**-1.59**	**0.3266**
GT_A_84_P801001	**TIP2;2**	**-2.11**	**0.3303**
GT_A_84_P847726	**EPR1**	**-1.71**	**0.3312**
GT_A_84_P72824	**AT5G38890**	**-0.65**	**0.3316**
GT_A_84_P13963	**TT4**	**-1.48**	**0.3346**
GT_A_84_P22697	**UGT78D1**	**-1.02**	**0.3378**
GT_A_84_P522895	**AT5G10210**	**-1.84**	**0.3388**
GT_A_84_P812870	**TA29821_3702**	**-1.27**	**0.3395**
GT_A_84_P511862	**AT2G32550**	**-1.43**	**0.3419**
GT_A_84_P14603	**COBL2**	**-1.37**	**0.3433**
GT_A_84_P18726	**WNK9**	**-2.23**	**0.3488**
GT_A_84_P16959	**AT5G15700**	**-1.13**	**0.3505**
GT_A_84_P809469	**TA28050_3702**	**-0.79**	**0.3506**
GT_A_84_P740321	**FAR5**	**-1.72**	**0.3551**
GT_A_84_P728206	**DR368472**	**-4.10**	**0.3552**
GT_A_84_P15222	**4CL3**	**-1.99**	**0.3593**
GT_A_84_P18829	**ARR6**	**-1.24**	**0.3605**
GT_A_84_P87399	**AT3G06520**	**-0.93**	**0.3649**

GT_A_84_P19577	SHM4	-1.16	0.3698
GT_A_84_P22153	ERD5	-1.33	0.3708
GT_A_84_P22197	AT1G02620	-1.61	0.3710
GT_A_84_P12056	AT5G08030	-1.30	0.3712
GT_A_84_P15123	AT1G02310	-1.08	0.3721
GT_A_84_P541789	AT1G26250	-1.49	0.3724
GT_A_84_P761927	AT3G33528	-0.83	0.3738
GT_A_84_P189814	AT3G52670	-1.43	0.3741
GT_A_84_P757615	AT2G25482	-1.51	0.3752
GT_A_84_P86169	LSH9	-0.62	0.3753
GT_A_84_P814494	AT4G24830	-0.77	0.3763
GT_A_84_P761404	AT3G21460	-2.38	0.3779
GT_A_84_P18328	AT3G07750	-1.19	0.3784
GT_A_84_P19109	AT1G44160	-1.15	0.3791
GT_A_84_P22596	WOX2	-1.34	0.3824
GT_A_84_P21792	ATBCAT-1	-0.96	0.3833
GT_A_84_P20866	AXR3	-1.58	0.3834
GT_A_84_P12026	UBC17	-1.94	0.3863
GT_A_84_P235233	AT4G36610	-0.92	0.3879
GT_A_84_P23590	AT5G20370	-1.50	0.3891
GT_A_84_P562254	AT1G66060	-1.47	0.3925
GT_A_84_P297414	AT3G62070	-0.60	0.3925
GT_A_84_P23204	AT3G59480	-1.50	0.3949
GT_A_84_P16644	YUC2	-0.97	0.3954
GT_At_Specific_00314270	TRNI.4	-0.88	0.3972
GT_A_84_P278740	AT1G52720	-0.73	0.4052
GT_A_84_P15448	ATPP2-A3	-1.93	0.4059
GT_A_84_P838052	AXR3	-1.68	0.4101
GT_A_84_P832673	AT3G20340	-1.40	0.4111
GT_A_84_P556511	AT4G29110	-1.14	0.4128
GT_A_84_P750646	AT1G59920	-1.12	0.4131
GT_A_84_P16520	BXL2	-0.99	0.4133
GT_A_84_P769609	AT5G53048	-1.68	0.4142
GT_A_84_P809692	DELTA-TIP	-0.95	0.4184
GT_A_84_P22447	AT5G05420	-1.24	0.4202
GT_A_84_P122822	AT1G60080	-0.80	0.4262
GT_A_84_P812709	TA29745_3702	-1.17	0.4285
GT_A_84_P17137	ATNAP5	-0.66	0.4299
GT_A_84_P531516	AT3G25700	-0.95	0.4306
GT_A_84_P121202	AT5G56810	-1.25	0.4320
GT_A_84_P809677	DELTA-TIP	-0.80	0.4320
GT_A_84_P12395	AT1G32900	-2.69	0.4331
GT_A_84_P606261	AT4G01023	-1.38	0.4335
GT_A_84_P286080	AT5G19410	-0.80	0.4352
GT_A_84_P510870	AT1G72645	-0.76	0.4391
GT_A_84_P164953	ABF1	-0.72	0.4424
GT_A_84_P14811	AT4G30680	-0.98	0.4468

GT_A_84_P12420	**AT1G14730**	**-1.05**	**0.4482**
GT_A_84_P850921	**AT2G21330**	**-0.90**	**0.4514**
GT_A_84_P179244	**VFB3**	**-0.65**	**0.4524**
GT_A_84_P17207	**AT1G57590**	**-1.53**	**0.4557**
GT_A_84_P224099	**EPR1**	**-1.69**	**0.4562**
GT_A_84_P156895	**AT3G47210**	**-1.19**	**0.4569**
GT_A_84_P17987	**AT1G23140**	**-1.08**	**0.4585**
GT_A_84_P17091	**ATH7**	**-1.05**	**0.4588**
GT_A_84_P15108	**AT1G23205**	**-1.37**	**0.4590**
GT_A_84_P13934	**AT5G05270**	**-1.69**	**0.4601**
GT_A_84_P529724	**AT3G20340**	**-1.24**	**0.4665**
GT_A_84_P755976	**AT2G32350**	**-1.06**	**0.4713**
GT_A_84_P847991	**CCA1**	**-1.97**	**0.4720**
GT_A_84_P12152	**NF-YB6**	**-1.16**	**0.4756**
GT_A_84_P13826	**BGLU47**	**-0.94**	**0.4819**
GT_A_84_P787920	**FAR5**	**-1.26**	**0.4943**
GT_A_84_P558547	**AT5G65850**	**-0.74**	**0.4971**
GT_A_84_P586198	**AT1G15510**	**-0.85**	**0.5015**
GT_A_84_P858735	**VTC4**	**-1.01**	**0.5067**
GT_A_84_P816899	**AT2G34750**	**-0.83**	**0.5077**
GT_A_84_P14468	**CKX5**	**-0.86**	**0.5188**
GT_A_84_P812380	**ERD5**	**-1.37**	**0.5205**
GT_A_84_P761568	**AT3G52060**	**-1.61**	**0.5213**
GT_A_84_P23721	**AT1G14200**	**-2.00**	**0.5255**
GT_A_84_P18531	**AT4G12050**	**-0.81**	**0.5286**
GT_A_84_P240805	**LHCB2.1**	**-0.90**	**0.5344**
GT_A_84_P204968	**ATOCT2**	**-1.00**	**0.5401**
GT_A_84_P868161	**AT1G14200**	**-1.81**	**0.5537**
GT_A_84_P817892	**AT5G05440**	**-1.21**	**0.5543**
GT_A_84_P10277	**IMPA-8**	**-0.71**	**0.5616**
GT_At_Specific_00052106	**AT1G54750.1**	**-1.42**	**0.5757**
GT_A_84_P592063	**AT4G14746**	**-0.71**	**0.5770**
GT_A_84_P146199	**AT5G05440**	**-1.18**	**0.5776**
GT_At_Specific_00073790	**AT1G74929.1**	**-1.00**	**0.5780**
GT_A_84_P288490	**LHCB2.2**	**-0.87**	**0.5906**
GT_A_84_P23912	**ATPT2**	**-1.45**	**0.5944**
GT_At_Specific_00071252	**AT1G72645.1**	**-0.82**	**0.5962**
GT_A_84_P17470	**AT3G46070**	**-1.43**	**0.5977**
GT_A_84_P753739	**4CL1**	**-0.64**	**0.6073**
GT_A_84_P700286	**TA50872_3702**	**-0.64**	**0.6078**
GT_A_84_P761945	**AT3G61028**	**-0.89**	**0.6101**
GT_A_84_P20714	**ACHT5**	**-1.13**	**0.6111**
GT_A_84_P844879	**AT3G06080**	**-0.69**	**0.6176**
GT_A_84_P21181	**AT3G26760**	**-0.69**	**0.6225**
GT_A_84_P14746	**AT-HSFB2B**	**-1.11**	**0.6306**
GT_A_84_P611961	**AT5G35120**	**-0.78**	**0.6308**
GT_A_84_P788197	**AT2G32550**	**-1.02**	**0.6395**

GT_A_84_P751246	**AT1G54750**	**-1.33**	**0.6408**
GT_A_84_P558814	**AT2G25780**	**-0.92**	**0.6444**
GT_A_84_P13323	**AT1G47480**	**-0.79**	**0.6469**
GT_A_84_P852047	**AT5G05440**	**-1.48**	**0.6478**
GT_A_84_P11987	**AT4G33420**	**-0.93**	**0.6555**
GT_A_84_P558224	**AT4G15990**	**-1.02**	**0.6681**
GT_A_84_P10925	**AT3G62950**	**-1.85**	**0.6783**
GT_A_84_P816246	**CAD4**	**-0.79**	**0.4155**
GT_A_84_P819186	**F2KP**	**-0.67**	**0.6996**
GT_A_84_P553302	**AT1G25500**	**-0.63**	**0.7044**
GT_A_84_P793199	**ATPT2**	**-1.19**	**0.7093**
GT_A_84_P16816	**IMS2**	**-1.22**	**0.7193**
GT_A_84_P22166	**ESM1**	**-0.76**	**0.7199**
GT_A_84_P198204	**AT2G17036**	**-0.61**	**0.7528**
GT_A_84_P71564	**MIOX2**	**-1.44**	**0.7585**
GT_A_84_P169033	**TPX2**	**-0.48**	**0.9353**
GT_At_Specific_00238330	**AT4G36850.1**	**-1.06**	**0.8475**
GT_A_84_P192434	**AT4G36850**	**-0.92**	**0.8814**
GT_A_84_P87509	**ATMRP15**	**-0.77**	**0.9096**
GT_At_Specific_00052096	**AT1G54740.1**	**-0.85**	**0.9518**
GT_A_84_P22210	**AT3G48390**	**-1.31**	**0.9654**
GT_A_84_P21458	**BT5**	**-1.00**	**0.9718**
GT_A_84_P14902	**AT5G11670**	**-0.56**	**0.7731**
GT_A_84_P788231	**ATGSTF14**	**-0.57**	**0.0329**
GT_A_84_P800547	**BP581162**	**-1.00**	**0.260**

Supplementary Table S2. Differentially expressed genes in *aba* 1.6 against combined stress.
Up-regulated genes in *aba* 1.6

		Fold change *aba* 1.6 vs Col-0	
**ProbeName**	**GeneName**	**GeoMean(3 replicate)Log2 Fold Change**	**P_Value**
GT_A_84_P149348	AT2G34170	0.90	0.0000
GT_A_84_P827673	AT4G32560	0.67	0.0001
GT_A_84_P849302	AT4G26795	1.32	0.0001
GT_A_84_P274730	CLE13	1.10	0.0004
GT_A_84_P811814	TA29222_3702	0.71	0.0004
GT_A_84_P599208	APRR2	0.79	0.0005
GT_A_84_P83489	AT3G56580	0.64	0.0005
GT_A_84_P589074	AT2G25050	1.09	0.0006
GT_A_84_P18031	AT1G05660	1.74	0.0007
GT_A_84_P851276	GAMMA_CA3	0.83	0.0007
GT_A_84_P829132	AT1G12620	0.67	0.0008
GT_A_84_P787198	AT5G42680	1.49	0.0008
GT_A_84_P20942	ATHVA22A	0.75	0.0008
GT_A_84_P826824	AT1G14230	0.86	0.0008
GT_A_84_P19103	CIB5	0.82	0.0009
GT_A_84_P832277	TA49635_3702	0.63	0.0009
GT_A_84_P537948	AT5G03010	1.04	0.0009
GT_A_84_P310733	AT1G78730	0.62	0.0009
GT_A_84_P754192	AT1G17255	0.73	0.0010
GT_A_84_P819325	AT1G52190	2.11	0.0010
GT_A_84_P514482	AT2G20440	0.75	0.0011
GT_A_84_P12480	AT1G58160	0.63	0.0014
GT_A_84_P599664	CW7	0.72	0.0014
GT_A_84_P14920	AT5G18940	0.70	0.0014
GT_A_84_P570961	AT1G02290	0.85	0.0015
GT_A_84_P10112	EDA40	0.82	0.0015
GT_At_Specific_00134604	AT3G03370.1	0.90	0.0015
GT_A_84_P20167	AT2G39510	0.99	0.0016
GT_A_84_P806297	AT4G18670	0.98	0.0017
GT_A_84_P838453	ATHB-3	1.72	0.0017
GT_A_84_P757826	AT2G19582	0.92	0.0018
GT_A_84_P15640	AT3G60290	2.81	0.0018
GT_A_84_P854582	AT5G43710	1.01	0.0019
GT_A_84_P785087	PHO2	0.78	0.0021
GT_A_84_P18263	AT2G38090	1.18	0.0021
GT_A_84_P17866	AT1G01680	1.63	0.0021
GT_A_84_P12487	AT1G14390	2.33	0.0022
GT_At_Specific_00178534	AT3G49730.1	0.81	0.0022
GT_A_84_P18945	ATSK41	0.67	0.0022
GT_A_84_P824452	AT3G09410	1.47	0.0024
GT_A_84_P19685	AHP3	0.65	0.0025
GT_A_84_P784765	AT5G57270	0.61	0.0026

GT_A_84_P764999	**AT4G26795**	**1.32**	**0.0026**
GT_A_84_P14365	**AT1G29740**	**0.65**	**0.0026**
GT_A_84_P14524		**0.63**	**0.0027**
GT_A_84_P91899	**TC376551**	**0.71**	**0.0027**
GT_A_84_P804017	**AT5G10336**	**0.62**	**0.0027**
GT_A_84_P845669		**1.23**	**0.0028**
GT_A_84_P848953	**AT1G74680**	**1.05**	**0.0029**
GT_A_84_P95119	**AT3G14840**	**1.28**	**0.0029**
GT_A_84_P853208	**TA52396_3702**	**1.01**	**0.0029**
GT_A_84_P19251	**COBL1**	**1.04**	**0.0031**
GT_A_84_P523888	**AT5G48040**	**0.68**	**0.0032**
GT_A_84_P12630	**AT3G01490**	**1.62**	**0.0033**
GT_A_84_P826553	**AT1G20410**	**0.75**	**0.0034**
GT_A_84_P23631	**AT4G18375**	**0.62**	**0.0034**
GT_A_84_P855865	**EXGT-A1**	**1.12**	**0.0035**
GT_A_84_P20688	**AT5G55050**	**0.92**	**0.0036**
GT_A_84_P849653	**AT2G01680**	**1.09**	**0.0037**
GT_A_84_P22444	**PDK1**	**0.60**	**0.0037**
GT_At_Specific_00120274	**AT2G38690.1**	**1.70**	**0.0038**
GT_A_84_P12545	**SGR5**	**1.28**	**0.0039**
GT_A_84_P17272	**PIN4**	**0.87**	**0.0039**
GT_A_84_P21294	**AT3G55090**	**2.02**	**0.0039**
GT_A_84_P12705	**CYP705A18**	**0.90**	**0.0040**
GT_A_84_P11048	**AT4G34810**	**5.82**	**0.0042**
GT_A_84_P853598	**AT1G78730**	**0.84**	**0.0042**
GT_A_84_P853499	**AT1G08480**	**0.79**	**0.0042**
GT_A_84_P17455	**DRT100**	**1.96**	**0.0044**
GT_A_84_P266250	**AT5G01970**	**0.65**	**0.0045**
GT_A_84_P835812	**AT4G39450**	**0.72**	**0.0046**
GT_A_84_P854517	**AT3G59950**	**0.73**	**0.0047**
GT_A_84_P763367	**AT4G06598**	**0.92**	**0.0050**
GT_A_84_P848192	**AT4G03140**	**1.04**	**0.0050**
GT_A_84_P855337	**AtTLP2**	**0.89**	**0.0050**
GT_A_84_P10165	**AT5G08010**	**0.68**	**0.0052**
GT_A_84_P89159	**AT4G05400**	**0.91**	**0.0052**
GT_A_84_P582757	**AT1G70950**	**1.58**	**0.0052**
GT_A_84_P20587	**ATHB-3**	**1.77**	**0.0054**
GT_A_84_P11645	**CAX1**	**0.76**	**0.0055**
GT_A_84_P827171	**AT5G38590**	**1.01**	**0.0055**
GT_A_84_P203668	**XYLT**	**0.62**	**0.0055**
GT_A_84_P13110	**AT5G51350**	**0.61**	**0.0055**
GT_A_84_P811885	**AT3G16850**	**1.18**	**0.0055**
GT_A_84_P835866	**AT5G44410**	**0.83**	**0.0055**
GT_A_84_P10694	**EXP3**	**1.20**	**0.0057**
GT_A_84_P141449	**TC376275**	**0.73**	**0.0057**
GT_A_84_P14739	**AtHSD5**	**3.19**	**0.0057**
GT_A_84_P756180	**AGP9**	**0.72**	**0.0058**

GT_A_84_P549074	**AT2G22530**	**0.61**	**0.0058**
GT_A_84_P17053	**AT1G09820**	**0.68**	**0.0059**
GT_At_Specific_00233093	**AT4G32360.1**	**0.65**	**0.0059**
GT_A_84_P10667	**BLH8**	**1.15**	**0.0059**
GT_A_84_P861913	**TC403018**	**2.47**	**0.0059**
GT_A_84_P858602	**AT3G12710**	**1.95**	**0.0062**
GT_A_84_P13084	**AT5G44410**	**0.82**	**0.0063**
GT_A_84_P21767	**AT1G60630**	**0.89**	**0.0064**
GT_A_84_P816205	**ERS1**	**0.64**	**0.0064**
GT_A_84_P257840	**NP281379**	**0.68**	**0.0065**
GT_A_84_P785399	**AT3G61010**	**0.68**	**0.0065**
GT_A_84_P12670	**AFB2**	**0.62**	**0.0066**
GT_A_84_P828798	**AT2G39510**	**1.08**	**0.0067**
GT_A_84_P849345	**AT3G24740**	**1.05**	**0.0068**
GT_A_84_P14989	**PAT1**	**0.73**	**0.0068**
GT_A_84_P792401	**AT3G26445**	**1.30**	**0.0069**
GT_A_84_P17011	**AT1G15850**	**0.63**	**0.0070**
GT_A_84_P20700	**ATL63**	**0.81**	**0.0070**
GT_A_84_P20356	**AT3G56640**	**0.60**	**0.0071**
GT_A_84_P842329	**NP236707**	**1.27**	**0.0071**
GT_A_84_P21493	**AT5G02540**	**1.32**	**0.0072**
GT_A_84_P818633	**UGT75B1**	**1.64**	**0.0073**
GT_A_84_P12692	**AT3G12710**	**1.97**	**0.0073**
GT_A_84_P92539	**AT2G21830**	**1.15**	**0.0073**
GT_A_84_P21449	**AT4G34770**	**5.11**	**0.0073**
GT_A_84_P11454	**ATCAX5**	**1.08**	**0.0074**
GT_A_84_P17860	**AT5G57850**	**0.74**	**0.0074**
GT_A_84_P17609	**AT4G21870**	**1.02**	**0.0075**
GT_A_84_P20336	**AtbZIP52**	**1.35**	**0.0075**
GT_A_84_P550988	**AT3G19400**	**1.04**	**0.0076**
GT_A_84_P827800	**AT3G28700**	**0.77**	**0.0077**
GT_A_84_P558998	**AT3G42800**	**3.03**	**0.0078**
GT_A_84_P862999	**AT5G25460**	**2.30**	**0.0080**
GT_A_84_P523896	**AT5G50335**	**2.24**	**0.0080**
GT_A_84_P13335	**AT1G08280**	**1.43**	**0.0081**
GT_A_84_P15051	**NPH3**	**1.23**	**0.0081**
GT_A_84_P532740	**AT5G42680**	**1.41**	**0.0082**
GT_A_84_P15787	**AT4G38650**	**0.68**	**0.0082**
GT_A_84_P837815	**AT5G44410**	**0.99**	**0.0085**
GT_A_84_P529817	**AT3G61010**	**0.78**	**0.0085**
GT_A_84_P172771	**AT5G06930**	**2.62**	**0.0086**
GT_A_84_P855785	**AT5G25460**	**2.20**	**0.0087**
GT_A_84_P586083	**GT_A_84_P586**	**0.67**	**0.0087**
GT_A_84_P207048	**AT3G19400**	**0.81**	**0.0088**
GT_A_84_P20511	**AGP18**	**1.90**	**0.0089**
GT_A_84_P549599	**AT5G49960**	**0.84**	**0.0090**
GT_A_84_P13210	**SP1L3**	**0.61**	**0.0090**

GT_A_84_P18230	**SCPL51**	**1.66**	**0.0091**
GT_A_84_P823234	**TA38586_3702**	**0.69**	**0.0092**
GT_A_84_P16193	**ATRECQ4B**	**0.70**	**0.0092**
GT_A_84_P794487	**SGR5**	**1.89**	**0.0093**
GT_A_84_P866944	**AT3G12950**	**0.61**	**0.0094**
GT_A_84_P13132	**XBAT32**	**0.86**	**0.0095**
GT_A_84_P596836	**WNK3**	**4.42**	**0.0096**
GT_A_84_P53520	**AT3G12950**	**0.79**	**0.0097**
GT_A_84_P292434	**AGP26**	**1.10**	**0.0097**
GT_A_84_P19878	**AT1G64640**	**2.31**	**0.0097**
GT_A_84_P842957	**AT1G22790**	**1.01**	**0.0097**
GT_A_84_P761712	**AT3G51075**	**0.78**	**0.0098**
GT_A_84_P23812	**ST4C**	**3.32**	**0.0098**
GT_A_84_P811125	**AT5G25460**	**1.73**	**0.0098**
GT_A_84_P857383	**BX839653**	**0.79**	**0.0099**
GT_A_84_P197484	**AT2G38310**	**1.18**	**0.0099**
GT_A_84_P761741	**AT3G46658**	**0.77**	**0.0100**
GT_A_84_P784694	**AT3G28430**	**0.74**	**0.0101**
GT_A_84_P843942	**BT012005**	**0.93**	**0.0102**
GT_A_84_P267530	**AT5G01800**	**0.68**	**0.0103**
GT_A_84_P554097	**AT1G76870**	**1.28**	**0.0103**
GT_A_84_P23213	**AT3G61810**	**2.40**	**0.0103**
GT_A_84_P12904	**AT4G26940**	**0.72**	**0.0104**
GT_A_84_P847105	**AT5G53020**	**1.26**	**0.0104**
GT_A_84_P821521	**TA36791_3702**	**0.86**	**0.0105**
GT_A_84_P16241	**AT1G52190**	**2.24**	**0.0105**
GT_A_84_P846758	**AT1G44750**	**0.71**	**0.0105**
GT_A_84_P850353	**AT2G45500**	**0.77**	**0.0106**
GT_A_84_P21591	**AZF3**	**1.47**	**0.0106**
GT_A_84_P861922	**SPL2**	**1.35**	**0.0106**
GT_A_84_P148358	**AT3G62630**	**0.78**	**0.0107**
GT_A_84_P77019	**AT3G26445**	**1.14**	**0.0108**
GT_A_84_P12778	**AT3G53840**	**0.75**	**0.0108**
GT_A_84_P851740	**SPL2**	**1.17**	**0.0108**
GT_A_84_P17238	**AT2G46290**	**0.66**	**0.0108**
GT_A_84_P501273	**AT3G04160**	**0.62**	**0.0109**
GT_A_84_P21770	**AT1G23060**	**2.19**	**0.0109**
GT_A_84_P23844	**AT2G43870**	**2.09**	**0.0109**
GT_A_84_P787811	**SOL1**	**0.79**	**0.0110**
GT_At_Specific_00001265	**AT1G02074.1**	**2.12**	**0.0111**
GT_A_84_P827371	**AT3G27470**	**0.65**	**0.0112**
GT_A_84_P17755	**anac088**	**1.38**	**0.0112**
GT_A_84_P19504	**AT4G22730**	**1.08**	**0.0112**
GT_A_84_P753604	**AT1G74088**	**1.31**	**0.0114**
GT_A_84_P161233	**AT3G03370**	**0.73**	**0.0114**
GT_A_84_P827583	**TA43778_3702**	**1.09**	**0.0115**
GT_A_84_P817119	**AT4G00355**	**0.68**	**0.0115**

GT_A_84_P833545	TA50784_3702	1.43	0.0117
GT_A_84_P821393	AT1G17160	0.67	0.0117
GT_At_Specific_00179597	U1-70K	0.60	0.0119
GT_A_84_P11065	CYP96A9	1.13	0.0119
GT_A_84_P552282	AT5G61190	0.72	0.0120
GT_A_84_P14396	CPuORF34	1.38	0.0122
GT_A_84_P808260	ATHB30	0.74	0.0123
GT_At_Specific_00129617	AGP16	1.19	0.0123
GT_A_84_P852838	ATSK41	0.87	0.0124
GT_A_84_P22004	UGT73C6	1.11	0.0124
GT_A_84_P17940	ULT2	1.69	0.0125
GT_A_84_P544654	AT2G23093	0.61	0.0125
GT_A_84_P19738	AT5G53890	0.77	0.0125
GT_A_84_P13706	AT3G50140	2.19	0.0125
GT_A_84_P304340	AT1G17450	0.61	0.0125
GT_A_84_P829880	AT4G19900	0.61	0.0126
GT_A_84_P196484	AT5G64020	0.78	0.0126
GT_At_Specific_00185243	AT3G55420.1	0.72	0.0126
GT_A_84_P270770	AT2G36500	0.76	0.0127
GT_A_84_P555983	AT1G30190	2.39	0.0127
GT_A_84_P852532	AT3G59950	0.79	0.0127
GT_A_84_P844440		1.17	0.0127
GT_A_84_P17980	AT1G64540	0.73	0.0127
GT_A_84_P790792	AT4G37682	1.37	0.0127
GT_A_84_P834115	AT5G54890	0.83	0.0129
GT_A_84_P15056	HSL2	0.69	0.0129
GT_A_84_P210398	AT2G31290	0.79	0.0130
GT_A_84_P845695	AT1G48430	1.67	0.0130
GT_A_84_P10102	AT4G34800	3.11	0.0132
GT_A_84_P567920	AT3G57950	1.62	0.0133
GT_A_84_P869391	AT3G17940	0.81	0.0134
GT_A_84_P13699	AT3G48550	1.94	0.0134
GT_A_84_P13937	AT1G68710	0.91	0.0135
GT_A_84_P507050	AT5G55430	2.37	0.0135
GT_A_84_P835152	AT1G20550	1.80	0.0136
GT_A_84_P592228	AT5G24280	0.73	0.0136
GT_A_84_P796920	AT1G64065	0.84	0.0137
GT_A_84_P822423	AT1G27210	0.81	0.0137
GT_A_84_P815084	AT3G01690	1.02	0.0139
GT_A_84_P825020	AT1G22790	1.18	0.0141
GT_A_84_P11447	GAI	1.10	0.0141
GT_A_84_P216098	CID8	1.11	0.0141
GT_A_84_P179124	AT5G05830	0.79	0.0142
GT_A_84_P24141	SDP1-LIKE	0.86	0.0143
GT_A_84_P262420	AtRLP26	1.63	0.0144
GT_A_84_P21338	AT4G01740	1.04	0.0145
GT_A_84_P806966	ATEXPA8	1.67	0.0145

GT_A_84_P18771	**FRA1**	**0.64**	**0.0147**
GT_A_84_P858214	**BP835218**	**0.82**	**0.0148**
GT_A_84_P23025	**AT2G46780**	**0.91**	**0.0149**
GT_A_84_P223699	**AT3G16580**	**1.68**	**0.0150**
GT_A_84_P12342	**ELP**	**1.49**	**0.0150**
GT_A_84_P786936	**AT2G42300**	**0.80**	**0.0152**
GT_A_84_P23047	**scpl25**	**0.62**	**0.0153**
GT_A_84_P594936	**CYP707A3**	**3.04**	**0.0153**
GT_A_84_P819987	**AT3G57800**	**1.07**	**0.0154**
GT_A_84_P287250	**AT4G22760**	**0.82**	**0.0155**
GT_A_84_P861620	**AI992798**	**0.71**	**0.0156**
GT_A_84_P787719	**AT4G01960**	**0.87**	**0.0157**
GT_A_84_P811245	**AT4G40030**	**1.15**	**0.0157**
GT_A_84_P822989	**TA38272_3702**	**0.61**	**0.0157**
GT_A_84_P737478	**DR379000**	**0.77**	**0.0158**
GT_A_84_P817475	**TA33153_3702**	**0.66**	**0.0158**
GT_A_84_P264730	**CPuORF40**	**0.79**	**0.0159**
GT_A_84_P71834	**AT5G58930**	**0.68**	**0.0160**
GT_A_84_P825125	**AtbZIP52**	**1.37**	**0.0161**
GT_At_Specific_00223833	**AT4G24530.1**	**0.61**	**0.0163**
GT_A_84_P801329	**FLA2**	**1.09**	**0.0164**
GT_A_84_P10765	**CCR1**	**1.88**	**0.0165**
GT_A_84_P819643	**AT3G17940**	**0.82**	**0.0165**
GT_A_84_P11379	**AT1G80790**	**0.60**	**0.0165**
GT_A_84_P15943	**NP230857**	**1.36**	**0.0165**
GT_A_84_P825877	**AT4G28650**	**1.52**	**0.0166**
GT_A_84_P853770	**BP813084**	**2.38**	**0.0167**
GT_A_84_P818943	**AFB2**	**0.81**	**0.0168**
GT_A_84_P863388	**BP867701**	**1.13**	**0.0168**
GT_A_84_P14782	**AT4G24530**	**0.67**	**0.0169**
GT_A_84_P67534	**PIN7**	**1.82**	**0.0169**
GT_A_84_P819086	**AT4G38520**	**0.90**	**0.0170**
GT_A_84_P20726	**AT1G52420**	**0.71**	**0.0170**
GT_At_Specific_00280620	**AT5G40240.1**	**0.69**	**0.0170**
GT_A_84_P824287	**AT1G70230**	**1.04**	**0.0170**
GT_A_84_P18657	**JKD**	**2.59**	**0.0171**
GT_A_84_P826755	**TA42759_3702**	**1.08**	**0.0172**
GT_A_84_P278840	**AT5G08270**	**0.85**	**0.0173**
GT_A_84_P848224	**BX839205**	**1.49**	**0.0173**
GT_At_Specific_00243913	**AT5G02480.1**	**1.57**	**0.0173**
GT_At_Specific_00129217	**AT2G46000.1**	**0.94**	**0.0173**
GT_A_84_P20800	**PRA1.F1**	**1.47**	**0.0174**
GT_A_84_P15764	**AT4G32460**	**1.41**	**0.0174**
GT_A_84_P606561	**AT5G59845**	**1.19**	**0.0176**
GT_A_84_P22087	**AT3G10190**	**1.30**	**0.0176**
GT_A_84_P826434	**AZF3**	**1.73**	**0.0177**
GT_A_84_P14798	**AT4G27900**	**1.21**	**0.0177**

GT_A_84_P828261	TA44707_3702	1.01	0.0177
GT_A_84_P821462	ATSK41	0.63	0.0179
GT_A_84_P807121	TA26786_3702	1.72	0.0179
GT_A_84_P823794	PDK1	0.70	0.0180
GT_A_84_P828934	AT4G01460	0.64	0.0180
GT_A_84_P22764	AT1G65180	1.13	0.0181
GT_A_84_P187444	AT5G48500	0.95	0.0181
GT_A_84_P10530	TUB1	1.67	0.0183
GT_A_84_P530717	AT4G00905	0.81	0.0183
GT_A_84_P835734	AT3G28130	1.23	0.0185
GT_A_84_P509353	AT3G28857	4.02	0.0185
GT_A_84_P786537	AT3G01720	0.77	0.0186
GT_A_84_P855270	AT5G49945	0.64	0.0186
GT_A_84_P759661	AT3G27500	3.80	0.0187
GT_A_84_P11460	AT1G67510	0.94	0.0187
GT_A_84_P535389	AT5G41850	1.05	0.0188
GT_A_84_P17660	AT4G33490	1.81	0.0189
GT_At_Specific_00028430	AT1G26200.1	1.13	0.0189
GT_A_84_P18041	AT1G32690	1.21	0.0191
GT_A_84_P22283	ARV2	0.77	0.0192
GT_A_84_P857453	CYP705A21	1.61	0.0193
GT_A_84_P51020	AT4G31150	0.61	0.0193
GT_A_84_P787532	AT5G06270	0.96	0.0194
GT_A_84_P524941	AT1G21320	1.89	0.0194
GT_At_Specific_00268583	AT5G25460.1	1.96	0.0194
GT_A_84_P837063	NFD4	0.78	0.0195
GT_A_84_P843537	NP454648	0.68	0.0196
GT_A_84_P16086	AT1G52100	1.29	0.0197
GT_A_84_P22396	scpl50	0.67	0.0197
GT_A_84_P168393	AT4G19045	0.91	0.0198
GT_At_Specific_00233873	AT4G32990.1	0.68	0.0198
GT_A_84_P15785	ATEXPA20	0.76	0.0199
GT_A_84_P750336	AT1G17160	0.80	0.0200
GT_A_84_P854812	AA394469	1.00	0.0200
GT_A_84_P199044	AT1G63150	0.90	0.0201
GT_A_84_P761356	AT3G61898	2.72	0.0203
GT_A_84_P21915	ATEXPA11	3.16	0.0204
GT_A_84_P848702	DBE1	0.81	0.0205
GT_At_Specific_00213942	AT4G16260.1	0.70	0.0205
GT_A_84_P510160	AT3G03240	1.47	0.0206
GT_A_84_P819847	CPuORF40	1.04	0.0207
GT_A_84_P13158	SNRK2.5	1.70	0.0207
GT_A_84_P826328	AT3G53100	0.84	0.0207
GT_A_84_P14933	AT5G25460	2.00	0.0208
GT_A_84_P15891	AHK2	0.86	0.0208
GT_A_84_P792126	AT4G39190	0.64	0.0209
GT_At_Specific_00267084	AT5G24200.1	2.40	0.0210

GT_A_84_P822174	SERK2	0.70	0.0210
GT_A_84_P851116	AT2G46780	0.84	0.0210
GT_A_84_P14483	ATCSLC08	0.97	0.0210
GT_A_84_P855388		2.44	0.0212
GT_A_84_P174801	AT3G09690	0.73	0.0212
GT_A_84_P848008	SCPL51	1.44	0.0214
GT_A_84_P826192	TA42106_3702	0.84	0.0214
GT_A_84_P17334	CYP710A1	1.61	0.0215
GT_A_84_P20398	AT4G03140	0.99	0.0216
GT_A_84_P11815	VDAC5	1.22	0.0219
GT_A_84_P15595	AT3G49970	1.60	0.0220
GT_A_84_P814601	AT4G25360	0.70	0.0221
GT_A_84_P585099	AT5G24200	2.49	0.0223
GT_A_84_P15178	DBE1	1.22	0.0224
GT_A_84_P211478	AT5G42320	0.64	0.0224
GT_A_84_P757000	AT2G40640	0.67	0.0224
GT_A_84_P850199	TC392633	0.60	0.0226
GT_A_84_P753500	AT1G51645	1.07	0.0227
GT_A_84_P836259	TA52379_3702	0.88	0.0227
GT_A_84_P513248	AT5G53020	1.35	0.0227
GT_A_84_P12879	AT1G29120	0.73	0.0228
GT_A_84_P23571	AT5G66080	1.21	0.0228
GT_A_84_P15292	AT1G13460	0.89	0.0228
GT_A_84_P856245	AT1G16690	0.79	0.0233
GT_A_84_P143149	PDLP3	0.94	0.0233
GT_A_84_P11569	ATEXPA8	1.73	0.0233
GT_A_84_P534779	ATRECQ4B	0.84	0.0233
GT_A_84_P17394	AT3G14240	0.71	0.0234
GT_A_84_P767648	AT5G47900	0.75	0.0234
GT_A_84_P730098	AK229047	0.81	0.0235
GT_A_84_P835924	AT3G18770	0.90	0.0235
GT_A_84_P796845	SAR1	1.40	0.0235
GT_A_84_P717103	OEP16-3	0.83	0.0236
GT_A_84_P21659	AT1G55360	0.95	0.0236
GT_At_Specific_00200393	AT4G04490.1	2.82	0.0236
GT_A_84_P523919	PRA1.G2	1.29	0.0237
GT_A_84_P12386	AT1G65920	1.59	0.0239
GT_A_84_P845464	AT4G13100	0.86	0.0239
GT_At_Specific_00147861	AT3G14452.1	1.10	0.0240
GT_A_84_P701725	AT5G24318	0.67	0.0241
GT_A_84_P10290	OPT1	1.36	0.0242
GT_A_84_P785814	AT4G11290	2.75	0.0242
GT_A_84_P14374	AT1G31770	0.72	0.0242
GT_A_84_P11824	AtSAT1	0.97	0.0242
GT_A_84_P587734	AT5G16170	1.22	0.0243
GT_A_84_P821176	ATVPS34	0.81	0.0245
GT_A_84_P739523	AT2G31150	1.11	0.0245

GT_A_84_P765191	**AT4G09840**	**1.06**	**0.0246**
GT_A_84_P566482	**AT5G66005**	**0.69**	**0.0247**
GT_A_84_P762549	**AT3G56408**	**0.82**	**0.0247**
GT_A_84_P174551	**AT5G49170**	**0.97**	**0.0249**
GT_A_84_P13126	**AT5G56040**	**1.10**	**0.0249**
GT_A_84_P754709	**AT1G75891**	**0.81**	**0.0249**
GT_A_84_P19119	**AT2G24800**	**2.61**	**0.0250**
GT_A_84_P792619	**AT4G18570**	**0.92**	**0.0250**
GT_A_84_P19050	**AT1G74510**	**0.62**	**0.0250**
GT_A_84_P20149	**AT2G04570**	**1.45**	**0.0250**
GT_A_84_P16579	**AT1G21560**	**0.82**	**0.0251**
GT_A_84_P21732	**AT1G71695**	**0.75**	**0.0251**
GT_A_84_P11034	**FLA5**	**1.58**	**0.0251**
GT_A_84_P16994	**AT2G20835**	**1.20**	**0.0252**
GT_A_84_P17147	**ARAB-1**	**2.01**	**0.0254**
GT_A_84_P165853	**AT5G05180**	**0.85**	**0.0256**
GT_A_84_P862920	**BP845828**	**2.01**	**0.0256**
GT_A_84_P846024	**AT5G04070**	**0.96**	**0.0257**
GT_A_84_P768403	**IDL3**	**2.28**	**0.0258**
GT_A_84_P10300	**AT5G58790**	**0.68**	**0.0259**
GT_A_84_P758943	**BGLU8**	**0.90**	**0.0260**
GT_A_84_P763032	**AT4G08598**	**1.49**	**0.0261**
GT_A_84_P19958	**UGT75B1**	**1.56**	**0.0262**
GT_A_84_P23754	**AIG1**	**1.76**	**0.0262**
GT_A_84_P752591	**AT1G79970**	**1.11**	**0.0262**
GT_A_84_P788457	**AT2G20650**	**0.81**	**0.0263**
GT_At_Specific_00132674	**AT3G01720.1**	**0.70**	**0.0263**
GT_A_84_P830800	**AT5G58390**	**1.55**	**0.0265**
GT_A_84_P596013	**AT1G22060**	**0.79**	**0.0267**
GT_A_84_P819274	**TAF6**	**1.00**	**0.0267**
GT_A_84_P582417	**AT5G26230**	**0.99**	**0.0267**
GT_A_84_P16764	**AT5G02750**	**1.13**	**0.0268**
GT_A_84_P764131	**XDH2**	**1.39**	**0.0269**
GT_A_84_P23837	**AT1G33640**	**1.29**	**0.0269**
GT_A_84_P824588	**AT3G28690**	**0.72**	**0.0270**
GT_A_84_P862121	**AT3G16850**	**0.81**	**0.0271**
GT_A_84_P712221	**AT1G72855**	**1.33**	**0.0272**
GT_A_84_P828302	**AT3G59680**	**0.89**	**0.0272**
GT_A_84_P834307	**AT3G50590**	**1.33**	**0.0273**
GT_A_84_P21344	**GRH1**	**1.16**	**0.0274**
GT_A_84_P113382	**AT3G23930**	**1.85**	**0.0276**
GT_A_84_P833087	**KAN**	**0.66**	**0.0276**
GT_A_84_P753341	**AT1G29357**	**1.34**	**0.0276**
GT_At_Specific_00071474	**AT1G72855.1**	**1.30**	**0.0276**
GT_A_84_P789711	**BX838014**	**1.09**	**0.0277**
GT_A_84_P22232	**AT1G06760**	**0.91**	**0.0277**
GT_A_84_P786788	**ARI12**	**1.35**	**0.0278**

GT_A_84_P598624	AT4G31650	1.41	0.0278
GT_A_84_P20911	ATHB13	0.83	0.0280
GT_A_84_P22008	SMO2-2	0.79	0.0282
GT_A_84_P19014	AT1G78480	1.13	0.0283
GT_A_84_P831505	AT2G25420	0.67	0.0284
GT_A_84_P22024	OBP2	0.83	0.0284
GT_A_84_P11189	AT1G49790	0.82	0.0287
GT_A_84_P16452	AT3G15740	0.90	0.0287
GT_A_84_P12875	TC373188	0.64	0.0289
GT_A_84_P12172	SDG29	0.81	0.0289
GT_A_84_P22905	SFH3	0.77	0.0289
GT_A_84_P829148	TA45941_3702	1.54	0.0290
GT_A_84_P515865	AT5G26690	2.35	0.0290
GT_A_84_P158795	AT4G13540	1.18	0.0291
GT_A_84_P575680	AT2G17680	4.99	0.0293
GT_A_84_P312493	AT4G16060	0.80	0.0294
GT_A_84_P811955	TA29274_3702	1.23	0.0294
GT_A_84_P801437	AT3G16370	0.79	0.0295
GT_A_84_P829886	AT4G19900	0.91	0.0296
GT_A_84_P17568	ATRFNR1	1.05	0.0296
GT_A_84_P12382	ATPME2	1.22	0.0297
GT_At_Specific_00154682	CYP705A18	0.89	0.0297
GT_A_84_P243945	AT5G22580	1.42	0.0297
GT_A_84_P815336	TA31520_3702	0.70	0.0300
GT_A_84_P764577	AT4G14819	4.16	0.0301
GT_A_84_P13824	ATSBT3.12	0.73	0.0301
GT_A_84_P751207	AT1G22980	0.65	0.0302
GT_A_84_P763808	AT4G08940	0.89	0.0302
GT_A_84_P838344	AT2G23100	0.74	0.0302
GT_A_84_P22003	AT2G27060	0.90	0.0303
GT_A_84_P850227	NP186789	0.89	0.0304
GT_A_84_P788171	AT1G50950	1.11	0.0305
GT_A_84_P825282	AT5G23870	1.25	0.0305
GT_A_84_P597535	AT5G14090	1.65	0.0305
GT_A_84_P800461	UNE2	1.26	0.0305
GT_A_84_P11330	AT1G11545	1.89	0.0306
GT_A_84_P16486	CYP705A21	1.20	0.0306
GT_A_84_P765839	AT4G38825	4.06	0.0306
GT_A_84_P820600	AT5G50180	1.11	0.0307
GT_A_84_P548427	AT3G59270	0.62	0.0307
GT_At_Specific_00216849	AT4G18570.1	0.82	0.0307
GT_A_84_P186864	IQD12	1.69	0.0308
GT_A_84_P525902	AT1G60460	1.04	0.0308
GT_A_84_P788511	AT3G08840	0.64	0.0309
GT_A_84_P14722	SULTR3;2	1.82	0.0309
GT_A_84_P17300	AT2G45120	0.93	0.0310
GT_A_84_P16633	AT4G10550	0.93	0.0310

GT_A_84_P784572	AT3G28130	1.12	0.0311
GT_A_84_P18528	AT4G11290	3.49	0.0312
GT_A_84_P815956	TA31977_3702	0.72	0.0313
GT_A_84_P591388	AT5G46850	0.92	0.0313
GT_A_84_P22537	SPL2	1.21	0.0315
GT_At_Specific_00234264	AT4G33355.1	2.66	0.0317
GT_A_84_P19829	AT5G58390	2.02	0.0319
GT_A_84_P842613	NP281268	0.75	0.0320
GT_A_84_P849161	NP222417	1.88	0.0321
GT_A_84_P67694	WAVE1	1.78	0.0323
GT_A_84_P17433	CYP705A15	1.08	0.0324
GT_A_84_P754202	AT1G76878	0.75	0.0325
GT_A_84_P843471	AT2G25050	1.12	0.0325
GT_A_84_P839471	AT5G22608	0.80	0.0327
GT_A_84_P18423	AT3G47480	1.87	0.0327
GT_A_84_P18585	AT4G28650	1.27	0.0328
GT_A_84_P16608	MYB55	1.32	0.0328
GT_A_84_P821925	EXP3	0.85	0.0329
GT_A_84_P817573	TA33253_3702	0.64	0.0329
GT_A_84_P842862	AT5G19840	1.19	0.0329
GT_A_84_P829642	AT1G30160	1.22	0.0330
GT_A_84_P755476	AT2G13480	2.79	0.0330
GT_A_84_P788468	AT2G20440	0.83	0.0331
GT_A_84_P18510	AT4G04490	2.88	0.0331
GT_A_84_P830908	AT5G64020	0.67	0.0334
GT_A_84_P271670	AT5G03830	1.16	0.0336
GT_A_84_P557876	AT1G77660	1.20	0.0336
GT_A_84_P77389	IQD30	1.07	0.0337
GT_A_84_P836362	AT2G17550	1.54	0.0339
GT_A_84_P828767	AT1G32260	0.73	0.0339
GT_A_84_P229619	AT2G42320	0.70	0.0340
GT_A_84_P10151	KCS19	2.80	0.0342
GT_A_84_P842591	AT1G62910	1.36	0.0342
GT_A_84_P189964	AT2G19660	4.24	0.0344
GT_A_84_P74014	AT3G11340	1.09	0.0344
GT_At_Specific_00159469	AT3G23930.1	1.71	0.0346
GT_A_84_P791325	AT1G48780	0.96	0.0347
GT_A_84_P19347	TRFL9	1.24	0.0347
GT_A_84_P72314	VOZ1	1.08	0.0348
GT_A_84_P560912	AT4G24275	2.31	0.0349
GT_A_84_P729473	TAS1B	0.81	0.0349
GT_A_84_P760084	AT3G12915	0.75	0.0350
GT_A_84_P150248	RRA2	1.16	0.0354
GT_A_84_P10747	WAG1	1.40	0.0355
GT_A_84_P14008	AT5G38590	1.20	0.0356
GT_A_84_P279580	AT3G50800	0.86	0.0357
GT_A_84_P15756	AT4G30520	0.67	0.0357

GT_A_84_P848531	TTL	0.61	0.0358
GT_A_84_P787342	XDH2	1.34	0.0359
GT_A_84_P589500	AT5G13920	0.65	0.0360
GT_A_84_P846661	AT2G25590	1.19	0.0360
GT_A_84_P829753	PRA1.G2	1.17	0.0361
GT_A_84_P855942	MAP3KA	1.14	0.0362
GT_A_84_P19668	AT5G27220	1.54	0.0362
GT_A_84_P507058	AT5G57760	3.04	0.0362
GT_At_Specific_00300416	AT5G56747.1	0.61	0.0362
GT_A_84_P827953	AT1G72270	0.61	0.0363
GT_A_84_P848781	FUT13	0.64	0.0363
GT_A_84_P754189	AT1G77138	1.08	0.0365
GT_A_84_P16544	KRP2	1.19	0.0366
GT_A_84_P16980	AT5G28300	1.06	0.0367
GT_A_84_P856554	AT4G06701	1.61	0.0368
GT_A_84_P804260	CPuORF43	0.78	0.0368
GT_At_Specific_00229481	AT4G29310.1	1.91	0.0370
GT_A_84_P827070	AT2G45720	0.69	0.0370
GT_A_84_P18645	AT1G27210	0.61	0.0371
GT_A_84_P828660	AT2G20100	0.84	0.0372
GT_A_84_P13950	TGA4	1.13	0.0373
GT_A_84_P16320	AT2G26360	0.77	0.0374
GT_A_84_P233409	AT5G24210	0.89	0.0374
GT_A_84_P14194	AT1G04040	1.48	0.0377
GT_A_84_P861057	NRT1.1	1.10	0.0380
GT_A_84_P544206	AT5G19060	1.44	0.0382
GT_A_84_P10483	AT1G15260	1.42	0.0383
GT_A_84_P760534	AT3G06440	0.65	0.0385
GT_A_84_P856844	AHBP-1B	1.04	0.0385
GT_A_84_P514022	AT5G10745	0.96	0.0386
GT_A_84_P861017	AT2G36670	1.12	0.0387
GT_A_84_P15272	UNE2	1.09	0.0388
GT_A_84_P807065	AT1G24793	0.96	0.0390
GT_A_84_P20745	PGLCT	0.92	0.0390
GT_A_84_P811949	AT1G17140	1.41	0.0390
GT_A_84_P828142	AT3G20620	0.93	0.0391
GT_A_84_P311843	AT4G30500	1.28	0.0392
GT_A_84_P855778	AT5G58200	0.68	0.0393
GT_A_84_P227839	AT1G67050	1.07	0.0394
GT_A_84_P829463	AT5G17340	1.65	0.0394
GT_A_84_P154215	LNG2	0.72	0.0395
GT_A_84_P263610	AT4G14380	1.08	0.0397
GT_A_84_P818389	AT1G50440	0.77	0.0399
GT_A_84_P567083	AT4G13070	0.70	0.0399
GT_A_84_P816480	TA32349_3702	0.80	0.0400
GT_A_84_P554500	AT3G08670	1.09	0.0401
GT_A_84_P786909	PAP3	1.74	0.0401

GT_A_84_P67134	**CPuORF22**	**0.74**	**0.0402**
GT_A_84_P206348	**AT1G60060**	**1.32**	**0.0402**
GT_A_84_P820592	**AT5G50180**	**1.11**	**0.0402**
GT_A_84_P851051	**AGP4**	**1.60**	**0.0404**
GT_A_84_P848619	**AT1G02145**	**0.62**	**0.0405**
GT_A_84_P23176	**AT3G53100**	**0.77**	**0.0405**
GT_A_84_P24010	**AT3G06740**	**0.89**	**0.0407**
GT_A_84_P826593	**TA42527_3702**	**2.09**	**0.0408**
GT_A_84_P19012	**UGT75B2**	**0.93**	**0.0408**
GT_A_84_P750950	**AT1G23465**	**0.74**	**0.0408**
GT_A_84_P12625	**SYP131**	**1.77**	**0.0409**
GT_A_84_P243195	**AT4G29310**	**1.85**	**0.0409**
GT_A_84_P792613	**DR232279**	**1.75**	**0.0411**
GT_A_84_P23536	**AT5G57970**	**1.50**	**0.0411**
GT_A_84_P22847	**AT1G14550**	**3.74**	**0.0413**
GT_A_84_P23932	**AT2G01070**	**0.73**	**0.0413**
GT_A_84_P576539	**AT1G80540**	**0.82**	**0.0413**
GT_A_84_P233769	**AT5G02480**	**1.41**	**0.0413**
GT_A_84_P16991	**SIP1**	**0.76**	**0.0414**
GT_A_84_P16022	**ATSPS1F**	**0.81**	**0.0415**
GT_A_84_P823977	**AT5G06180**	**0.63**	**0.0416**
GT_A_84_P10109	**AT4G36240**	**1.28**	**0.0417**
GT_A_84_P812986	**AT5G41920**	**1.67**	**0.0417**
GT_A_84_P511366	**AT5G10320**	**0.66**	**0.0418**
GT_A_84_P832647	**TA49826_3702**	**0.74**	**0.0419**
GT_A_84_P23287	**AT4G22530**	**0.85**	**0.0419**
GT_A_84_P750979	**AT1G44940**	**0.65**	**0.0420**
GT_A_84_P845749	**AT3G06770**	**1.62**	**0.0420**
GT_A_84_P850543	**AT3G06770**	**1.40**	**0.0422**
GT_A_84_P21390	**GA2OX8**	**3.08**	**0.0426**
GT_A_84_P850685	**GUT15**	**0.71**	**0.0426**
GT_At_Specific_00201060	**AT4G05030.1**	**1.67**	**0.0430**
GT_A_84_P519209	**AT4G11450**	**1.06**	**0.0430**
GT_A_84_P16446	**AT3G16700**	**1.53**	**0.0430**
GT_A_84_P859502	**FLA2**	**0.92**	**0.0431**
GT_A_84_P279800	**AT5G12170**	**1.00**	**0.0432**
GT_At_Specific_00235814	**AT4G34560.1**	**1.07**	**0.0434**
GT_A_84_P15532	**CID9**	**2.32**	**0.0435**
GT_A_84_P824941	**AT1G29240**	**1.43**	**0.0436**
GT_A_84_P159455	**AT1G12620**	**0.74**	**0.0436**
GT_A_84_P23485	**YUC5**	**3.28**	**0.0436**
GT_A_84_P11008	**AT4G25750**	**1.09**	**0.0437**
GT_A_84_P857924	**AT3G27470**	**1.22**	**0.0437**
GT_A_84_P767872	**AT5G42730**	**1.62**	**0.0438**
GT_A_84_P820213	**AAE1**	**1.31**	**0.0439**
GT_A_84_P752481	**AT1G62590**	**1.49**	**0.0440**
GT_A_84_P853293	**AT1G63010**	**1.14**	**0.0444**

GT_A_84_P845539	TC377407	2.23	0.0444
GT_A_84_P844437	AT4G11450	1.14	0.0444
GT_A_84_P796368	EF183146	0.67	0.0445
GT_A_84_P804662	ACBP2	0.72	0.0445
GT_A_84_P81479	PUB25	0.98	0.0446
GT_A_84_P788513	AT2G16770	0.81	0.0446
GT_A_84_P14545	SCPL27	1.01	0.0447
GT_A_84_P804193	ATIPT1	1.63	0.0447
GT_A_84_P15071	AT5G16940	0.77	0.0448
GT_A_84_P790886	AT1G69340	1.54	0.0450
GT_A_84_P231759	AT3G57800	1.19	0.0450
GT_A_84_P854454	AT2G10940	1.01	0.0452
GT_A_84_P141109	AT4G30410	0.70	0.0452
GT_A_84_P15653	scpl40	1.69	0.0454
GT_A_84_P17338	CYCP4;1	1.48	0.0454
GT_A_84_P572671	AT5G48440	0.78	0.0456
GT_A_84_P18581	OPT6	1.05	0.0457
GT_A_84_P18501	NRT1.1	1.09	0.0457
GT_A_84_P797512	AT3G26100	0.88	0.0459
GT_A_84_P852182	CAT3	1.47	0.0460
GT_A_84_P851423	AT4G32440	0.70	0.0462
GT_A_84_P828057	AT5G57780	1.12	0.0464
GT_A_84_P11535	AT1G58150	1.59	0.0465
GT_A_84_P273780	AT1G49470	1.10	0.0466
GT_A_84_P21466	BRI1	0.67	0.0467
GT_A_84_P846807	TUB5	1.04	0.0467
GT_A_84_P824302	AT4G29310	1.86	0.0468
GT_A_84_P13361	AGO7	1.15	0.0468
GT_A_84_P19725	CLC-C	1.03	0.0468
GT_A_84_P830586	AT5G22310	1.93	0.0469
GT_At_Specific_00194370	AT3G63330.1	0.98	0.0469
GT_A_84_P16776	AT5G06760	1.82	0.0470
GT_A_84_P811603	RD22	1.05	0.0470
GT_A_84_P760477	BX823963	0.97	0.0470
GT_A_84_P800943	IAGLU	1.53	0.0471
GT_A_84_P846684	GT72B1	0.98	0.0471
GT_A_84_P789543	AT5G57760	3.07	0.0471
GT_A_84_P789634	AK227233	0.91	0.0472
GT_A_84_P851229	AT4G13970	1.27	0.0475
GT_A_84_P20311	AT1G48430	1.02	0.0475
GT_A_84_P588280	AT3G04020	0.61	0.0475
GT_A_84_P518748	AT1G26900	0.79	0.0477
GT_A_84_P836727	AT2G27060	1.11	0.0478
GT_A_84_P10420	CB5LP	0.66	0.0480
GT_A_84_P822774	AT3G50210	1.39	0.0480
GT_A_84_P544696	AT2G36110	0.63	0.0481
GT_A_84_P822840	AT3G11340	1.21	0.0481

GT_A_84_P534015	**AT2G23360**	**0.75**	**0.0481**
GT_A_84_P818130	**AT1G62610**	**1.23**	**0.0482**
GT_A_84_P582659	**AT1G30160**	**1.14**	**0.0482**
GT_A_84_P857966	**PRXR1**	**1.83**	**0.0483**
GT_A_84_P834134	**AT3G61010**	**0.64**	**0.0484**
GT_A_84_P11713	**AT3G02980**	**0.93**	**0.0489**
GT_A_84_P21246	**AT3G43960**	**1.64**	**0.0491**
GT_At_Specific_00003309	**AT1G03820.1**	**2.58**	**0.0491**
GT_A_84_P69024	**AT3G13030**	**1.45**	**0.0491**
GT_A_84_P23648	**AT1G51170**	**0.87**	**0.0492**
GT_A_84_P787372	**AT4G32440**	**0.93**	**0.0492**
GT_A_84_P806687	**CAT3**	**1.42**	**0.0495**
GT_A_84_P16143	**AT1G54450**	**0.88**	**0.0497**
GT_A_84_P19976	**IRT3**	**0.87**	**0.0498**
GT_A_84_P754730	**AT1G16489**	**1.24**	**0.0498**
GT_A_84_P15509	**CPuORF30**	**1.07**	**0.0500**
GT_A_84_P818147	**PIN3**	**0.97**	**0.0503**
GT_A_84_P21429	**AT4G30020**	**1.18**	**0.0504**
GT_A_84_P11019	**TET7**	**0.92**	**0.0504**
GT_A_84_P835099	**ATPUP5**	**0.64**	**0.0505**
GT_A_84_P305320	**AT5G22310**	**2.15**	**0.0506**
GT_A_84_P830871	**AT5G03830**	**0.92**	**0.0506**
GT_A_84_P296064	**NP208858**	**1.99**	**0.0506**
GT_A_84_P768715	**AT5G15265**	**1.10**	**0.0507**
GT_At_Specific_00122896	**AT2G40820.1**	**1.13**	**0.0507**
GT_A_84_P96296	**AT3G24450**	**0.73**	**0.0507**
GT_A_84_P826365	**AT3G50650**	**1.49**	**0.0512**
GT_A_84_P10599	**AT1G14460**	**0.71**	**0.0512**
GT_A_84_P220988	**APUM4**	**0.69**	**0.0513**
GT_A_84_P14541	**APC11**	**0.66**	**0.0513**
GT_At_Specific_00113177	**AT2G32820.1**	**1.02**	**0.0515**
GT_A_84_P811841	**AALP**	**1.20**	**0.0517**
GT_A_84_P829630	**DRB4**	**0.97**	**0.0518**
GT_A_84_P501000	**NFD4**	**0.60**	**0.0518**
GT_A_84_P12398	**AT1G53340**	**1.45**	**0.0518**
GT_A_84_P532282	**AT2G37300**	**0.92**	**0.0521**
GT_A_84_P12687	**AT1G26650**	**0.82**	**0.0521**
GT_A_84_P799971	**AT2G01340**	**1.59**	**0.0524**
GT_A_84_P716292	**AK229755**	**0.90**	**0.0525**
GT_A_84_P851395	**AT5G15780**	**0.73**	**0.0525**
GT_A_84_P20185	**EBF1**	**0.66**	**0.0526**
GT_A_84_P18635	**AT4G15160**	**0.72**	**0.0527**
GT_A_84_P17614	**CRK13**	**1.48**	**0.0527**
GT_A_84_P794114	**AY069871**	**0.82**	**0.0527**
GT_A_84_P769325	**AT5G11412**	**2.01**	**0.0530**
GT_A_84_P854874	**AT3G46450**	**1.08**	**0.0531**
GT_A_84_P178444	**AT2G24290**	**0.92**	**0.0533**

GT_A_84_P128641	BRX	0.91	0.0535
GT_A_84_P870394	STY1	0.71	0.0536
GT_A_84_P16963	AT5G16960	0.88	0.0536
GT_At_Specific_00191593	AT3G61020.1	1.33	0.0537
GT_A_84_P763555	BX826473	1.80	0.0537
GT_A_84_P20594	PUCHI	1.58	0.0537
GT_A_84_P21625	LTI65	2.65	0.0539
GT_A_84_P11903	ORA59	2.02	0.0539
GT_A_84_P20679	AT5G52280	1.31	0.0540
GT_A_84_P818327	AT1G51590	0.66	0.0541
GT_A_84_P826352	AT1G16750	1.32	0.0541
GT_A_84_P11358	AT1G03820	2.82	0.0543
GT_A_84_P835598	AT5G22700	0.70	0.0544
GT_A_84_P758683	AT2G21230	0.65	0.0544
GT_A_84_P829924	EYE	1.00	0.0546
GT_A_84_P546754	AT4G23030	1.44	0.0547
GT_A_84_P206528	AT1G79110	1.09	0.0548
GT_A_84_P11329	AT2G01130	0.69	0.0549
GT_A_84_P829494	TA46381_3702	0.84	0.0549
GT_A_84_P787882	AT1G65920	1.73	0.0553
GT_A_84_P855667	AT2G25760	0.65	0.0554
GT_A_84_P583902	AT3G21465	0.83	0.0554
GT_A_84_P817316	TA33020_3702	0.78	0.0559
GT_A_84_P215098	AT4G30710	1.43	0.0560
GT_A_84_P21328	AT3G63330	1.03	0.0561
GT_A_84_P825671	AT4G08940	0.66	0.0561
GT_A_84_P808035	DWF1	0.61	0.0562
GT_A_84_P14764	AT4G20100	1.38	0.0562
GT_A_84_P13859	AT4G29240	0.77	0.0565
GT_A_84_P13165	AT5G65320	2.88	0.0566
GT_A_84_P112762	AT5G02670	0.84	0.0566
GT_A_84_P10482	AT1G14210	0.88	0.0567
GT_A_84_P532007	AT1G15215	0.75	0.0567
GT_A_84_P767749	AT5G39970	1.46	0.0570
GT_A_84_P765701	AT4G26055	0.72	0.0570
GT_A_84_P765161	AT4G37608	0.86	0.0572
GT_A_84_P577990	AT5G44570	1.26	0.0576
GT_A_84_P185924	AT5G19340	0.64	0.0578
GT_A_84_P16577	AT1G21520	1.80	0.0580
GT_A_84_P839412	AY735672	1.93	0.0581
GT_A_84_P790360	AT3G03702	1.12	0.0584
GT_A_84_P857806	CRK	1.60	0.0585
GT_A_84_P819124	TA34543_3702	1.56	0.0587
GT_A_84_P22564	AT5G50180	1.19	0.0587
GT_A_84_P11546	TCP24	0.66	0.0587
GT_A_84_P848155	AA650689	0.82	0.0587
GT_A_84_P798242	AT3G26890	1.44	0.0589

GT_A_84_P786646	**AT2G43060**	**0.83**	**0.0591**
GT_A_84_P13326	**PIN3**	**0.82**	**0.0591**
GT_A_84_P851045	**AT2G34840**	**0.90**	**0.0591**
GT_A_84_P22058	**CCR2**	**2.18**	**0.0593**
GT_A_84_P588626	**AT5G19670**	**0.78**	**0.0593**
GT_A_84_P244795	**AT1G29240**	**1.35**	**0.0596**
GT_A_84_P24159	**AT3G61820**	**1.23**	**0.0601**
GT_A_84_P19276	**AT1G53040**	**1.08**	**0.0604**
GT_A_84_P795830	**DQ108818**	**1.90**	**0.0606**
GT_A_84_P146228	**AAE1**	**1.10**	**0.0608**
GT_A_84_P11173	**AT5G38550**	**1.77**	**0.0609**
GT_A_84_P792558	**DR354216**	**1.46**	**0.0610**
GT_A_84_P814006	**ATPLDDELTA**	**1.26**	**0.0611**
GT_A_84_P613653	**AtGDU3**	**0.98**	**0.0615**
GT_A_84_P18749	**AT5G41920**	**1.90**	**0.0615**
GT_A_84_P13961	**PBS3**	**0.86**	**0.0616**
GT_A_84_P15502	**WAG2**	**4.05**	**0.0616**
GT_A_84_P858250	**AT2G38970**	**1.42**	**0.0620**
GT_A_84_P761743	**AT3G03702**	**1.65**	**0.0620**
GT_A_84_P19554	**AT1G50440**	**0.68**	**0.0621**
GT_A_84_P530096	**AT5G50330**	**0.60**	**0.0622**
GT_A_84_P808432	**PRXR1**	**1.83**	**0.0623**
GT_At_Specific_00159109	**RTFL13**	**2.67**	**0.0623**
GT_A_84_P238143	**AT2G34340**	**1.45**	**0.0625**
GT_A_84_P12653	**AT3G08660**	**1.05**	**0.0626**
GT_At_Specific_00232170	**ATCSLC05**	**0.89**	**0.0626**
GT_A_84_P14657	**AT3G51470**	**1.27**	**0.0627**
GT_A_84_P831322	**AT3G18770**	**1.82**	**0.0627**
GT_A_84_P12584	**ATNUDT6**	**2.46**	**0.0631**
GT_A_84_P19184	**TC403570**	**1.05**	**0.0632**
GT_A_84_P829761	**PRA1.G2**	**0.98**	**0.0634**
GT_A_84_P174151	**AT2G40330**	**1.49**	**0.0634**
GT_A_84_P808422	**PRXR1**	**1.69**	**0.0635**
GT_A_84_P12186	**AT5G57480**	**1.23**	**0.0636**
GT_A_84_P22810	**BEE3**	**2.53**	**0.0636**
GT_A_84_P869674	**AT3G50650**	**1.77**	**0.0637**
GT_A_84_P830613	**AT3G04485**	**1.39**	**0.0640**
GT_A_84_P761775	**AT3G04485**	**0.86**	**0.0642**
GT_At_Specific_00095933	**GUT15**	**0.68**	**0.0642**
GT_A_84_P14793	**AT1G21890**	**3.04**	**0.0645**
GT_A_84_P808775	**AT3G22440**	**0.65**	**0.0646**
GT_At_Specific_00179557	**AT3G50650.1**	**1.28**	**0.0648**
GT_A_84_P751820		**1.07**	**0.0649**
GT_A_84_P119672	**AT5G53830**	**1.41**	**0.0651**
GT_A_84_P795094	**AT5G28850**	**1.22**	**0.0651**
GT_A_84_P19414	**AT3G57680**	**2.20**	**0.0651**
GT_A_84_P14338	**ZFP4**	**1.27**	**0.0652**

GT_A_84_P549710	AT1G05950	0.78	0.0658
GT_A_84_P273250	AT5G56220	1.33	0.0659
GT_A_84_P11475	PAP3	1.79	0.0660
GT_A_84_P62200	ZPR1	1.95	0.0661
GT_A_84_P806696	CAT3	1.39	0.0662
GT_At_Specific_00239073	AT4G37480.1	1.07	0.0662
GT_A_84_P135745	AT5G49215	0.99	0.0663
GT_A_84_P817875	GT_A_84_P817	1.10	0.0664
GT_A_84_P750680	AT1G63330	1.09	0.0670
GT_A_84_P181884	AT1G17140	1.30	0.0670
GT_A_84_P13169	AT5G66330	1.04	0.0671
GT_A_84_P297904	AT2G41230	3.43	0.0672
GT_A_84_P63574	AT2G22960	1.92	0.0672
GT_A_84_P828987	AT1G53040	0.88	0.0673
GT_A_84_P597466	AT4G32580	0.70	0.0676
GT_A_84_P19499	AT4G21400	1.44	0.0678
GT_A_84_P751743	AT1G78260	1.76	0.0681
GT_A_84_P16376	AT2G32630	0.61	0.0682
GT_A_84_P15342	AT2G41820	1.85	0.0683
GT_A_84_P18308	AtRABA5b	0.70	0.0684
GT_A_84_P826388	BR6OX2	2.13	0.0685
GT_A_84_P10269	AT1G29640	1.36	0.0690
GT_A_84_P829727	PBS3	1.01	0.0694
GT_A_84_P22673	AT2G40435	1.80	0.0698
GT_A_84_P15411	CYP71B9	1.71	0.0699
GT_A_84_P21511	AT5G07720	1.29	0.0699
GT_A_84_P100756	TA26043_3702	0.63	0.0700
GT_A_84_P10700	ARR11	0.75	0.0701
GT_A_84_P848029	AT5G53420	1.98	0.0703
GT_A_84_P90919	AT3G61950	2.08	0.0704
GT_At_Specific_00119568	AT2G38090.1	1.03	0.0705
GT_A_84_P98986	AT1G72720	2.11	0.0706
GT_A_84_P854478	NF-YA5	2.19	0.0706
GT_A_84_P524502	ARGOS	2.46	0.0707
GT_A_84_P795657	AT1G65320	0.71	0.0707
GT_A_84_P814112	POP2	1.19	0.0712
GT_A_84_P814302	AT5G11420	1.08	0.0712
GT_A_84_P570004	CPuORF50	1.13	0.0715
GT_A_84_P15647	AT3G62110	0.87	0.0716
GT_A_84_P18587	ATNRT2:1	3.52	0.0717
GT_A_84_P804126	BP799962	1.26	0.0718
GT_A_84_P825992	WAVE1	1.39	0.0720
GT_A_84_P217088	PLDGAMMA3	0.60	0.0722
GT_A_84_P857601		1.63	0.0722
GT_A_84_P861616	CO048667	1.05	0.0725
GT_A_84_P851667	AT3G23840	0.79	0.0729
GT_A_84_P816751	AT1G55840	0.64	0.0730

GT_A_84_P15354	AT2G20780	0.69	0.0732
GT_A_84_P848977	AT1G68600	1.59	0.0732
GT_A_84_P83759	AK228652	1.12	0.0732
GT_A_84_P12295	AT1G64450	1.47	0.0733
GT_A_84_P12867	KAT2	1.48	0.0734
GT_A_84_P855906	BE039541	1.15	0.0736
GT_A_84_P16626	AT4G08770	1.74	0.0737
GT_A_84_P137919	AT5G53220	1.42	0.0741
GT_A_84_P833145	AT2G41820	2.40	0.0741
GT_A_84_P18482	BRH1	1.42	0.0742
GT_A_84_P832866	BPC5	1.02	0.0743
GT_A_84_P842320	NP236646	4.02	0.0743
GT_A_84_P16886	AtPNG1	0.64	0.0744
GT_A_84_P15221	AT1G22540	0.94	0.0745
GT_A_84_P848738	AT3G51950	0.86	0.0746
GT_A_84_P515035	AT5G54850	0.84	0.0748
GT_A_84_P11414	AT1G05640	1.44	0.0752
GT_A_84_P753464	AT1G18415	0.64	0.0755
GT_A_84_P789092	AT4G31150	0.86	0.0760
GT_A_84_P840186	AT3G48490	0.90	0.0761
GT_A_84_P19115	AT2G43590	1.58	0.0762
GT_A_84_P10570	AT1G19840	0.69	0.0762
GT_A_84_P822056	AT1G26650	0.68	0.0763
GT_A_84_P813316	AT3G07460	0.78	0.0768
GT_A_84_P243525	AT5G65040	1.82	0.0769
GT_A_84_P828773	AT3G14360	2.15	0.0769
GT_A_84_P22975	scpl26	0.67	0.0770
GT_A_84_P22825	ATFRUCT5	1.66	0.0774
GT_A_84_P297064	XTH17	1.45	0.0776
GT_A_84_P136105	AT3G14360	2.11	0.0776
GT_A_84_P528200	FHL	1.44	0.0780
GT_At_Specific_00293942	OLEO3	0.67	0.0781
GT_A_84_P845506	AT4G16850	1.44	0.0781
GT_A_84_P590032	AT2G42370	1.47	0.0781
GT_A_84_P845497	AT2G20780	0.85	0.0786
GT_A_84_P814311	AT4G16260	0.73	0.0786
GT_A_84_P790048	AT3G28956	1.35	0.0786
GT_A_84_P17352	RBK2	1.30	0.0787
GT_A_84_P827558	TA43735_3702	0.62	0.0789
GT_A_84_P15363	AT1G11270	0.78	0.0790
GT_A_84_P22569	AT5G51770	0.99	0.0791
GT_A_84_P584003	ULP1B	0.68	0.0792
GT_A_84_P753838	AT1G33615	0.88	0.0792
GT_A_84_P20638	GLIP1	4.51	0.0792
GT_A_84_P18056	MUB5	1.60	0.0793
GT_A_84_P605779	AT1G12845	1.05	0.0794
GT_At_Specific_00306369	AT5G61890.1	2.39	0.0794

GT_A_84_P59580	AT4G34560	1.18	0.0796
GT_A_84_P756996	AT2G46980	0.67	0.0797
GT_A_84_P832242	TA49615_3702	1.26	0.0799
GT_A_84_P821429	AT1G05720	0.64	0.0799
GT_A_84_P311113	AT-HSFA6A	4.02	0.0799
GT_A_84_P809730	AT5G14920	1.28	0.0800
GT_A_84_P766678	AT5G53420	2.08	0.0801
GT_A_84_P13936	ATL43	1.82	0.0806
GT_At_Specific_00157790	AT3G22550.1	1.86	0.0808
GT_A_84_P12415	AT1G27660	0.80	0.0808
GT_A_84_P10469	NF-YA5	2.13	0.0808
GT_A_84_P102416	LSH10	1.43	0.0809
GT_A_84_P12059	TC371211	0.87	0.0810
GT_A_84_P787303	SEC14	1.08	0.0810
GT_A_84_P20326	AT3G50210	0.86	0.0810
GT_A_84_P13931	AT5G04050	0.63	0.0811
GT_A_84_P11343	AT1G02260	0.71	0.0812
GT_A_84_P23664	BDG1	0.67	0.0813
GT_A_84_P240415	AT5G11550	0.66	0.0814
GT_A_84_P23503	AT5G48370	0.85	0.0817
GT_A_84_P23314	HAE	0.88	0.0817
GT_A_84_P822269	AT3G14150	0.80	0.0817
GT_A_84_P11540	AT1G26100	0.62	0.0818
GT_A_84_P817142	AT1G69510	0.84	0.0818
GT_A_84_P808037	AT1G28400	1.50	0.0819
GT_A_84_P843330	NP335312	1.22	0.0821
GT_A_84_P23055	AT1G53110	0.81	0.0823
GT_A_84_P12862	AT4G13190	1.99	0.0825
GT_A_84_P828329	AT3G59150	0.91	0.0826
GT_A_84_P137719	SCPL19	2.66	0.0826
GT_A_84_P751278	BX815895	1.24	0.0826
GT_A_84_P14339	MLP165	1.83	0.0827
GT_A_84_P11049	ADF9	0.68	0.0828
GT_A_84_P20465	RPS2	1.68	0.0828
GT_A_84_P16340	AT2G34840	0.75	0.0830
GT_A_84_P732920	AT1G12300	0.68	0.0831
GT_A_84_P790810	FHL	1.69	0.0833
GT_A_84_P856743	AT1G80420	0.63	0.0833
GT_A_84_P23515	GA20OX2	2.12	0.0834
GT_A_84_P824855	CYP711A1	0.93	0.0835
GT_A_84_P721249	AT5G41612	1.13	0.0836
GT_A_84_P860580	AT1G53040	0.94	0.0842
GT_A_84_P563122	AT1G61160	1.98	0.0842
GT_A_84_P822593	AtGDU3	0.69	0.0842
GT_A_84_P63150	AT5G53420	2.00	0.0845
GT_A_84_P823239	AT5G57700	0.74	0.0848
GT_A_84_P854498	AT5G44020	0.73	0.0848

GT_A_84_P12036	AT5G02410	0.89	0.0850
GT_A_84_P811071	TA28834_3702	0.87	0.0850
GT_A_84_P10042	AT4G21020	2.99	0.0851
GT_A_84_P790814	ATL43	2.54	0.0852
GT_A_84_P17610	SRF8	1.01	0.0853
GT_A_84_P582333	AT4G39190	0.73	0.0854
GT_A_84_P592126	CCD8	1.67	0.0855
GT_A_84_P822067	AT3G10915	0.77	0.0855
GT_A_84_P10266	AT5G48900	1.17	0.0859
GT_A_84_P850710	ZFP4	1.29	0.0863
GT_A_84_P10995	AT4G22840	1.06	0.0863
GT_A_84_P573423	AT4G36600	3.32	0.0866
GT_A_84_P610364	AT1G72490	1.19	0.0866
GT_A_84_P13493	AT2G30670	4.29	0.0867
GT_A_84_P69494	GCP2	1.54	0.0868
GT_A_84_P176634	ATPLDDELTA	1.18	0.0868
GT_A_84_P754404	AT1G22403	0.78	0.0868
GT_A_84_P839623	ATPIP5K1	0.61	0.0870
GT_A_84_P186514	AT5G45100	0.73	0.0870
GT_A_84_P857632	RAP2.2	0.70	0.0873
GT_A_84_P122602	FLA18	0.68	0.0878
GT_A_84_P11937	sks4	1.17	0.0880
GT_A_84_P18886	CAT3	0.98	0.0881
GT_A_84_P19456	AT4G04500	2.64	0.0881
GT_A_84_P66124	CB255404	1.06	0.0882
GT_A_84_P840606	NP221601	0.76	0.0882
GT_At_Specific_00201807	AT4G05590.2	1.71	0.0884
GT_At_Specific_00234961	AT4G33925.1	0.65	0.0885
GT_A_84_P17490	AT3G50650	1.23	0.0886
GT_A_84_P826039	AT2G29525	0.65	0.0886
GT_A_84_P12054	AT5G07580	0.95	0.0887
GT_A_84_P869119	FAD3	0.81	0.0888
GT_A_84_P833206	AT4G36600	2.94	0.0889
GT_A_84_P846110	AT3G49310	0.71	0.0889
GT_A_84_P827399	ATNEK1	0.89	0.0892
GT_A_84_P753520	AT1G01448	2.05	0.0895
GT_A_84_P202258	AT3G07470	0.73	0.0895
GT_A_84_P12451	AT1G72100	1.36	0.0902
GT_A_84_P12113	AT1G68600	1.90	0.0904
GT_A_84_P19244	AT3G05160	1.55	0.0904
GT_A_84_P550340	AT4G37380	0.68	0.0905
GT_A_84_P512655	AT1G77640	2.00	0.0906
GT_A_84_P10745	GLR1.4	0.89	0.0907
GT_A_84_P11503	AT1G75880	0.75	0.0908
GT_A_84_P799343	CPuORF43	1.06	0.0909
GT_A_84_P71224	TA50557_3702	0.96	0.0910
GT_A_84_P592665	AtGDU4	0.61	0.0915

GT_A_84_P726109	ABCB4	1.45	0.0916
GT_A_84_P23871	AT2G21220	3.48	0.0916
GT_A_84_P528560	AT1G54540	0.93	0.0917
GT_A_84_P11260	AT5G61610	0.89	0.0919
GT_A_84_P19222	NP453344	1.47	0.0922
GT_A_84_P835248	AT1G27660	0.98	0.0922
GT_A_84_P15199	AT1G59950	2.06	0.0922
GT_A_84_P757801	AT2G42365	1.10	0.0924
GT_At_Specific_00219528	AT4G21020.1	3.16	0.0926
GT_A_84_P15120	AT1G33840	1.43	0.0927
GT_A_84_P18290	AT2G24350	0.69	0.0928
GT_A_84_P850392	AT3G60960	0.72	0.0929
GT_At_Specific_00174375	AT3G46150.1	0.72	0.0931
GT_A_84_P808523	AT5G63190	1.48	0.0931
GT_A_84_P845621	DET2	0.64	0.0932
GT_A_84_P547579	AT4G05030	2.29	0.0934
GT_A_84_P548594	AT5G05840	2.66	0.0935
GT_A_84_P750345	AT1G49370	1.29	0.0936
GT_A_84_P11727	AT3G15670	2.83	0.0942
GT_A_84_P146938	NIMIN1	1.87	0.0945
GT_A_84_P10579	LUP1	1.34	0.0947
GT_A_84_P13101	AT5G48710	1.66	0.0948
GT_A_84_P821249	AT5G07580	0.88	0.0948
GT_A_84_P117612	RALFL33	0.61	0.0952
GT_A_84_P512914	AT3G46890	1.05	0.0952
GT_A_84_P822552	TA37791_3702	0.90	0.0953
GT_A_84_P867510	ADF4	1.18	0.0953
GT_A_84_P764356	TC380718	2.08	0.0954
GT_A_84_P18875	GH3.17	0.90	0.0954
GT_A_84_P84679	AT1G69520	1.29	0.0959
GT_A_84_P17301	SCPL11	1.61	0.0959
GT_A_84_P766772	AT5G38565	1.01	0.0960
GT_A_84_P124921	AT5G46680	1.11	0.0960
GT_A_84_P817913	TA33540_3702	1.08	0.0961
GT_A_84_P835688	AT5G02670	0.74	0.0962
GT_A_84_P740729	AK228652	1.06	0.0962
GT_A_84_P22600	TOE2	0.89	0.0966
GT_A_84_P24173	GT72B1	1.28	0.0967
GT_A_84_P755309	SCPL11	1.72	0.0967
GT_A_84_P502546	AT5G35525	2.51	0.0967
GT_A_84_P590334	AT4G33625	0.60	0.0968
GT_A_84_P801314	AT5G26600	0.83	0.0970
GT_A_84_P861492	BP820775	0.88	0.0973
GT_A_84_P21044	SCL21	0.67	0.0973
GT_A_84_P817317	ADR1-L1	1.63	0.0974
GT_A_84_P708402	AA041063	1.18	0.0975
GT_A_84_P103616	ARL2	0.96	0.0975

GT_A_84_P853304	AT5G01300	4.26	0.0977
GT_A_84_P792647	AT5G59662	2.08	0.0979
GT_A_84_P502211	AT3G18970	0.87	0.0982
GT_At_Specific_00009407	AT1G09010.1	0.71	0.0983
GT_A_84_P15747	ATM1	0.75	0.0983
GT_A_84_P17710	AT5G02760	3.19	0.0985
GT_A_84_P827374	CLV1	0.67	0.0989
GT_A_84_P12729	ATE2	0.92	0.0994
GT_A_84_P22617	SAL2	1.42	0.0994
GT_At_Specific_00156221	AT3G21350.2	2.02	0.0995
GT_A_84_P19582	CYP705A3	1.59	0.0998
GT_A_84_P11580	AT2G47870	1.65	0.0999
GT_A_84_P121502	AT1G57980	1.20	0.0999
GT_A_84_P223559	LURP1	2.85	0.1001
GT_A_84_P14721	AT1G14600	1.20	0.1002
GT_A_84_P817353	AAC2	0.80	0.1004
GT_A_84_P790839	CNS09Z5D	0.92	0.1005
GT_A_84_P805202	TA25867_3702	0.90	0.1005
GT_A_84_P24145	PDCB5	0.78	0.1006
GT_A_84_P150708	AT1G28400	1.15	0.1008
GT_A_84_P13002	scpl35	0.94	0.1009
GT_A_84_P187174	AT3G17520	4.29	0.1009
GT_A_84_P269270	AT5G50420	0.82	0.1011
GT_A_84_P803345	AT1G18010	1.68	0.1011
GT_A_84_P811915	RAB18	3.19	0.1013
GT_A_84_P594944	AT5G47635	0.93	0.1015
GT_A_84_P234563	AT2G25250	0.73	0.1019
GT_A_84_P14381	AT1G14490	0.82	0.1019
GT_A_84_P109232	AT2G16630	1.65	0.1021
GT_A_84_P102366	AT3G15280	1.37	0.1022
GT_A_84_P828489	AT4G04880	0.61	0.1022
GT_A_84_P22112	AT1G53070	2.24	0.1023
GT_A_84_P830191	AT1G73930	0.69	0.1024
GT_A_84_P808028	ACR3	0.80	0.1025
GT_A_84_P23719	MYB58	1.88	0.1027
GT_A_84_P605032	AT1G68935	0.79	0.1032
GT_A_84_P852180	AT2G36360	0.69	0.1032
GT_A_84_P14045	AT5G48430	0.85	0.1039
GT_A_84_P838362	AT2G43140	2.21	0.1042
GT_A_84_P606215	HR3	0.90	0.1044
GT_A_84_P811833	AALP	1.16	0.1045
GT_At_Specific_00037783	AT1G34280.1	1.72	0.1046
GT_A_84_P14373	AT1G31830	0.69	0.1049
GT_A_84_P10906	BZIP61	1.82	0.1050
GT_A_84_P820429	GAE4	1.56	0.1054
GT_A_84_P55550	IAA29	2.38	0.1059
GT_A_84_P20061	AT2G43580	1.87	0.1060

GT_At_Specific_00238421	SPT	0.91	0.1061
GT_A_84_P845483	TC371882	1.26	0.1063
GT_A_84_P846194	LNG1	1.28	0.1063
GT_A_84_P858035		1.24	0.1064
GT_A_84_P106472	AT1G22790	1.01	0.1065
GT_At_Specific_00031411	AT1G28620.1	1.18	0.1065
GT_A_84_P753419	AT1G60505	1.04	0.1066
GT_A_84_P813219	BME3	0.74	0.1069
GT_A_84_P21256	AT3G46490	3.31	0.1069
GT_A_84_P20206	AT3G10630	0.67	0.1074
GT_A_84_P20320	AtMYB45	3.17	0.1076
GT_A_84_P829523	AT1G21200	0.72	0.1079
GT_A_84_P21549	AT5G24010	1.22	0.1079
GT_A_84_P11935	NDB3	1.16	0.1080
GT_A_84_P563250	ATARD3	3.95	0.1081
GT_A_84_P16605	AT4G00870	1.02	0.1081
GT_A_84_P565339	AT4G23000	0.84	0.1084
GT_A_84_P281110	AT2G35730	2.73	0.1086
GT_A_84_P18061	AT1G10790	1.02	0.1086
GT_At_Specific_00055525	AT1G58460.1	0.93	0.1094
GT_A_84_P12031	AT5G01300	4.61	0.1095
GT_A_84_P750079	AT1G75150	0.91	0.1099
GT_A_84_P836406	AT1G15125	1.44	0.1101
GT_A_84_P766044	BX830275	0.76	0.1103
GT_A_84_P826160	TA42069_3702	0.89	0.1104
GT_A_84_P21751	AT1G64260	0.92	0.1104
GT_A_84_P841068	NP226287	0.93	0.1107
GT_A_84_P242623	TA44866_3702	0.74	0.1107
GT_A_84_P21070	AT2G28840	0.73	0.1109
GT_A_84_P230039	AT5G43065	1.57	0.1111
GT_A_84_P767924	YUC4	1.03	0.1112
GT_A_84_P16441	AT3G07010	1.76	0.1113
GT_A_84_P847963	BX837267	1.06	0.1116
GT_A_84_P19744	ATNHX3	0.86	0.1120
GT_A_84_P869955	AT3G07000	1.64	0.1123
GT_A_84_P812434	AT1G22882	0.91	0.1124
GT_A_84_P834773	LNG1	1.47	0.1126
GT_A_84_P851240	N65547	1.39	0.1127
GT_A_84_P789883	AT1G25570	1.19	0.1129
GT_A_84_P281230	AT5G15160	1.29	0.1131
GT_A_84_P14711	EM1	2.67	0.1132
GT_A_84_P14238	ATPANK1	0.77	0.1134
GT_A_84_P595425	AT3G05240	0.82	0.1135
GT_A_84_P869990	AT5G11140	1.43	0.1136
GT_A_84_P853135		0.74	0.1145
GT_A_84_P846752	BX835450	1.42	0.1147
GT_A_84_P837016	ERS2	1.93	0.1150

GT_A_84_P811890	**EXO**	**2.20**	**0.1151**
GT_A_84_P150068	**AT2G43140**	**2.05**	**0.1151**
GT_A_84_P837204	**ATCNGC9**	**1.82**	**0.1154**
GT_A_84_P820374	**AT5G66420**	**1.01**	**0.1155**
GT_A_84_P128051	**AT3G54780**	**0.94**	**0.1156**
GT_At_Specific_00176873	**WNK3**	**2.15**	**0.1160**
GT_A_84_P787309	**ATEXPA20**	**1.11**	**0.1163**
GT_At_Specific_00066600	**AT1G68600.1**	**1.88**	**0.1164**
GT_A_84_P510106	**AT2G35850**	**0.64**	**0.1170**
GT_A_84_P10083	**XTH19**	**1.68**	**0.1170**
GT_A_84_P78669	**AT5G03120**	**1.83**	**0.1175**
GT_A_84_P536931	**AT3G63450**	**1.07**	**0.1175**
GT_A_84_P847435	**AT3G08600**	**0.85**	**0.1177**
GT_A_84_P837267	**AT5G64320**	**1.50**	**0.1179**
GT_A_84_P166243	**AT5G66420**	**0.74**	**0.1180**
GT_A_84_P817721	**AKIN10**	**1.14**	**0.1182**
GT_A_84_P209848	**AT1G21460**	**1.46**	**0.1183**
GT_A_84_P184814	**AT5G02680**	**0.73**	**0.1188**
GT_A_84_P21909	**AOC4**	**0.63**	**0.1189**
GT_A_84_P13705	**AT3G49950**	**1.65**	**0.1191**
GT_A_84_P785300	**AT3G27640**	**1.00**	**0.1194**
GT_A_84_P12882	**TC371919**	**2.35**	**0.1195**
GT_A_84_P863449	**AT2G47770**	**2.76**	**0.1195**
GT_A_84_P536145	**AT4G31660**	**1.07**	**0.1196**
GT_A_84_P17465	**AT3G44760**	**1.47**	**0.1201**
GT_A_84_P17125	**GT_A_84_P171**	**1.05**	**0.1203**
GT_A_84_P22199	**AT3G45940**	**1.56**	**0.1204**
GT_A_84_P23970	**NGA1**	**0.81**	**0.1210**
GT_A_84_P230859	**AT3G22550**	**1.73**	**0.1212**
GT_A_84_P818026	**RPS2**	**1.47**	**0.1221**
GT_A_84_P169823	**AT1G73820**	**0.84**	**0.1223**
GT_A_84_P811265	**AT1G53780**	**0.78**	**0.1225**
GT_A_84_P269670	**IQD19**	**1.41**	**0.1229**
GT_A_84_P788604	**AY227633**	**0.84**	**0.1235**
GT_A_84_P11670	**NDA2**	**1.33**	**0.1235**
GT_A_84_P18901	**EG463478**	**1.60**	**0.1237**
GT_A_84_P23123	**AT3G13672**	**4.08**	**0.1238**
GT_A_84_P11863	**NP281378**	**0.89**	**0.1240**
GT_A_84_P15381	**ATEXPA4**	**1.46**	**0.1240**
GT_A_84_P800917	**EXL5**	**2.05**	**0.1243**
GT_A_84_P23328	**AT4G31600**	**0.92**	**0.1245**
GT_A_84_P18335	**AT3G02480**	**3.85**	**0.1246**
GT_A_84_P14271	**RGL1**	**1.32**	**0.1248**
GT_A_84_P788501	**AT3G10190**	**1.42**	**0.1249**
GT_A_84_P21658	**AT5G60980**	**1.10**	**0.1250**
GT_A_84_P816106	**FYD**	**0.62**	**0.1251**
GT_A_84_P73664	**AT1G77855**	**0.78**	**0.1252**

GT_A_84_P17666	XDH1	0.97	0.1253
GT_A_84_P540992	AT1G63320	2.24	0.1257
GT_A_84_P821929	AT4G33625	0.90	0.1258
GT_A_84_P153815	AT5G62170	0.98	0.1258
GT_A_84_P12239	AT5G20940	0.93	0.1259
GT_A_84_P14806	AT4G29680	0.69	0.1259
GT_A_84_P819785	TA35149_3702	0.66	0.1262
GT_A_84_P849566	TC391625	1.13	0.1263
GT_A_84_P11294	AT5G20935	0.81	0.1264
GT_A_84_P17216	INT2	2.21	0.1264
GT_A_84_P802051	AT3G16740	0.67	0.1264
GT_A_84_P531636	AT4G14840	0.66	0.1269
GT_A_84_P22898	AT2G43700	1.17	0.1269
GT_A_84_P827923	CCD8	1.62	0.1271
GT_A_84_P209378	CBL10	0.69	0.1283
GT_A_84_P94669	NP454260	1.02	0.1284
GT_A_84_P522586	SUVR4	1.39	0.1290
GT_A_84_P597110	AT2G25220	1.54	0.1299
GT_A_84_P806732	CAT3	1.55	0.1299
GT_A_84_P106016	AT1G15125	1.41	0.1306
GT_A_84_P13577	AT1G36940	1.74	0.1311
GT_A_84_P532635	AT5G03110	0.73	0.1311
GT_A_84_P22733	AT1G04560	2.76	0.1311
GT_A_84_P823272	AT5G41330	1.79	0.1312
GT_A_84_P834735	TA51636_3702	2.55	0.1313
GT_A_84_P257530	AT2G19340	0.69	0.1315
GT_A_84_P121762	AT2G23270	2.78	0.1315
GT_A_84_P788919	ATEXPA8	1.86	0.1315
GT_A_84_P806777	ELP	0.94	0.1318
GT_A_84_P842536	BX815895	1.96	0.1318
GT_A_84_P14489	AT2G13610	0.67	0.1318
GT_A_84_P754886	AK228652	0.94	0.1323
GT_A_84_P196504	AT5G18010	2.78	0.1338
GT_A_84_P608225	AT5G25010	0.90	0.1339
GT_A_84_P565934	AT2G34010	1.51	0.1347
GT_A_84_P196604	AT2G40530	0.90	0.1349
GT_A_84_P606349	AT4G28460	1.53	0.1349
GT_A_84_P18273	AT2G42290	1.24	0.1350
GT_A_84_P20544	AT1G62670	1.47	0.1351
GT_A_84_P12905	AT4G27250	0.95	0.1351
GT_A_84_P847389	GC2	0.61	0.1356
GT_A_84_P826615	AS2	1.21	0.1357
GT_A_84_P22467	AT5G11140	1.23	0.1359
GT_A_84_P18721	SYP41	0.92	0.1359
GT_A_84_P829954	AT1G22330	1.03	0.1362
GT_A_84_P828854	AT4G33130	1.34	0.1364
GT_A_84_P14558	AT3G22820	1.58	0.1365

GT_A_84_P94979	AT5G18060	2.34	0.1369
GT_A_84_P10014	AT4G10160	1.74	0.1372
GT_A_84_P23147	AT3G46280	2.21	0.1376
GT_A_84_P15602	ATGLR3.6	0.75	0.1376
GT_A_84_P12683	CPuORF11	0.75	0.1381
GT_A_84_P521700	AT3G04300	1.71	0.1385
GT_A_84_P11674	AT2G28940	0.85	0.1386
GT_A_84_P854224	CID10	0.84	0.1389
GT_A_84_P16879	AT5G47800	1.59	0.1390
GT_A_84_P611389	AT2G39280	0.76	0.1391
GT_A_84_P15560	BR6OX2	2.00	0.1391
GT_A_84_P811894	EXO	2.07	0.1391
GT_A_84_P859632	SGB1	1.46	0.1392
GT_A_84_P76839	AT4G15730	0.76	0.1395
GT_A_84_P598194	AT3G43850	1.14	0.1397
GT_A_84_P14112	AT1G52290	1.59	0.1397
GT_A_84_P15849	AT5G11930	1.86	0.1402
GT_A_84_P787949	AT2G46150	0.99	0.1403
GT_A_84_P206058	AT4G37290	2.38	0.1404
GT_A_84_P847349	AT4G26790	1.32	0.1404
GT_A_84_P190634	AT4G33130	0.69	0.1406
GT_A_84_P814568	AT2G27340	1.31	0.1407
GT_A_84_P10549	AT1G72540	3.06	0.1408
GT_A_84_P828710	MBD7	0.63	0.1408
GT_A_84_P23247	AT4G08570	5.25	0.1409
GT_A_84_P23285	PRXR1	1.29	0.1411
GT_A_84_P753360	AT1G06265	0.77	0.1414
GT_A_84_P19589	HAT1	0.84	0.1415
GT_A_84_P18532	AT4G12290	1.30	0.1415
GT_A_84_P788127	AtSec20	0.75	0.1416
GT_A_84_P796333	AT5G40980	2.33	0.1419
GT_A_84_P13257	AT1G60530	1.61	0.1421
GT_A_84_P827592	DAR2	0.81	0.1425
GT_A_84_P856004	AFB2	0.92	0.1430
GT_A_84_P15503	AT-HSFA6B	0.86	0.1432
GT_A_84_P11566	NIR1	0.80	0.1433
GT_A_84_P141279	AT1G18000	1.32	0.1442
GT_A_84_P11370	AT1G24240	0.80	0.1446
GT_A_84_P831143	AT1G04010	0.85	0.1450
GT_A_84_P18321	MSL4	1.13	0.1453
GT_A_84_P838477	NP032020	1.49	0.1458
GT_A_84_P735929	BX838743	1.17	0.1460
GT_A_84_P14305	AT1G66780	1.47	0.1461
GT_A_84_P19822	GLR2	0.90	0.1462
GT_A_84_P815273	WRKY53	1.21	0.1464
GT_A_84_P759045	AT3G59880	0.97	0.1470
GT_A_84_P521515	AT1G71140	0.88	0.1474

GT_A_84_P818467	AT5G64430	0.96	0.1477
GT_A_84_P784921	MYB58	1.29	0.1480
GT_A_84_P83309	AT3G07000	1.44	0.1481
GT_A_84_P604842	AT3G56470	1.14	0.1481
GT_At_Specific_00259496	AT5G16940.1	1.35	0.1486
GT_A_84_P16935	AT1G55260	0.69	0.1490
GT_A_84_P22740	AT1G77200	1.65	0.1493
GT_A_84_P788452	AT2G20835	1.71	0.1496
GT_A_84_P784166	CYP707A3	1.31	0.1496
GT_A_84_P69094	AT3G11040	0.71	0.1498
GT_A_84_P761316	MIR157C	1.44	0.1498
GT_A_84_P785722	AT4G36050	0.94	0.1499
GT_A_84_P18364	ZIP1	0.76	0.1502
GT_A_84_P831472	AT5G22700	0.64	0.1503
GT_A_84_P150678	AT5G06610	1.32	0.1506
GT_A_84_P604838	AT3G17280	0.91	0.1507
GT_A_84_P12554	AT2G29150	1.29	0.1508
GT_A_84_P855048	AT1G07440	0.83	0.1510
GT_A_84_P19233	FAD3	0.71	0.1512
GT_A_84_P816425	UPM1	0.96	0.1522
GT_A_84_P787457	AT4G25870	0.78	0.1523
GT_A_84_P764604	AT4G18501	0.79	0.1526
GT_A_84_P18592	AHA2	0.72	0.1530
GT_A_84_P757683	MIR396A	1.73	0.1532
GT_A_84_P64734	ALC	0.91	0.1539
GT_A_84_P109472	AT2G47770	2.62	0.1540
GT_A_84_P13790	EXO	2.19	0.1541
GT_A_84_P836191	TA52349_3702	2.32	0.1543
GT_A_84_P753369	AT1G60525	0.61	0.1544
GT_A_84_P820937	TTL3	0.60	0.1545
GT_A_84_P10502	AT1G18670	1.21	0.1546
GT_A_84_P596994	AT1G62610	0.83	0.1550
GT_A_84_P831555	AT4G11521	0.76	0.1553
GT_A_84_P19766	COBL5	0.66	0.1554
GT_A_84_P17688	AT4G14695	0.94	0.1559
GT_A_84_P14326	AtOCT1	1.37	0.1563
GT_A_84_P833637	AT3G51940	1.39	0.1566
GT_At_Specific_00306027	AT5G61570.1	1.17	0.1568
GT_A_84_P758187	AT2G26267	1.39	0.1570
GT_A_84_P824840	hemf2	1.01	0.1572
GT_A_84_P21626	MEF1	0.85	0.1576
GT_A_84_P16172	NP214745	1.30	0.1582
GT_A_84_P572395	SYP73	1.57	0.1585
GT_A_84_P546356	AT1G76610	2.77	0.1585
GT_A_84_P22382	ATCSLC5	0.78	0.1591
GT_A_84_P598538	AT5G52710	1.93	0.1592
GT_A_84_P798595	AT5G06755	1.39	0.1592

GT_A_84_P11156	WRKY50	2.53	0.1592
GT_A_84_P305230	AT5G18780	1.04	0.1594
GT_A_84_P22133	BGLU43	0.82	0.1594
GT_A_84_P286230	CLE27	2.26	0.1595
GT_A_84_P806759	CAT3	1.50	0.1597
GT_A_84_P820316	AT4G26790	1.09	0.1599
GT_A_84_P12143	CYP707A3	1.29	0.1600
GT_A_84_P15388	AT2G29300	2.15	0.1601
GT_A_84_P606916	AT2G30890	1.31	0.1604
GT_A_84_P819307	AT2G43590	1.76	0.1604
GT_A_84_P141269	AT5G18050	3.38	0.1606
GT_A_84_P178184	LNG1	1.04	0.1608
GT_A_84_P800012	AT1G05550	2.32	0.1610
GT_A_84_P796604	AK228267	0.74	0.1612
GT_A_84_P11224	AT5G52390	1.83	0.1617
GT_A_84_P825403	NST-K1	0.94	0.1618
GT_A_84_P831845	AT1G61100	0.74	0.1622
GT_A_84_P21917	AT1G22330	1.81	0.1626
GT_A_84_P557103	AT2G39530	1.84	0.1626
GT_A_84_P845944	TC370161	1.15	0.1628
GT_A_84_P294124	AT3G52360	0.99	0.1629
GT_A_84_P539856	AT5G50645	1.30	0.1630
GT_A_84_P857652		0.61	0.1635
GT_A_84_P823829	AT3G55420	0.66	0.1638
GT_At_Specific_00085555	AT2G05294.1	2.19	0.1638
GT_A_84_P588958	AT1G63630	1.21	0.1643
GT_A_84_P99776	AtRLP41	3.22	0.1646
GT_A_84_P769363	AT5G06278	0.74	0.1653
GT_A_84_P208548	PROPEP1	0.99	0.1654
GT_At_Specific_00176647	AT3G48131.1	1.25	0.1656
GT_A_84_P12165	AT5G51310	2.78	0.1658
GT_A_84_P800404	EG529050	1.28	0.1659
GT_A_84_P161393	AT3G18050	0.78	0.1661
GT_A_84_P24123	AT3G53250	3.32	0.1663
GT_A_84_P235303	AT1G66450	3.08	0.1667
GT_A_84_P768803	AT5G64572	0.83	0.1667
GT_A_84_P817510	PMR6	0.93	0.1676
GT_A_84_P10941	ATNHX8	0.60	0.1681
GT_A_84_P19588	ATEXLB1	3.42	0.1688
GT_A_84_P855014	OBE2	0.81	0.1694
GT_A_84_P212598	AT1G63400	1.37	0.1696
GT_A_84_P757998	AT2G20562	1.00	0.1698
GT_A_84_P13687	PHOT1	0.63	0.1703
GT_A_84_P785070	DML1	0.91	0.1706
GT_A_84_P759889	AT3G44420	1.17	0.1707
GT_A_84_P859050	TC399099	1.52	0.1708
GT_A_84_P69394	AT4G18670	1.24	0.1710

GT_A_84_P11090	PCS1	0.97	0.1713
GT_A_84_P841211	CPuORF11	0.84	0.1713
GT_A_84_P825792	ATEXLB1	2.95	0.1714
GT_A_84_P20125	CYP711A1	0.82	0.1719
GT_A_84_P842922	AT3G13980	1.70	0.1720
GT_A_84_P16571	WRKY70	0.98	0.1725
GT_A_84_P813977	FAD3	0.85	0.1733
GT_A_84_P17970	AT1G23670	1.26	0.1734
GT_A_84_P828562	ATMPK7	1.07	0.1735
GT_A_84_P20748	RCP1	0.94	0.1739
GT_At_Specific_00109643	AT2G29910.2	1.91	0.1740
GT_A_84_P723377	NP226912	1.11	0.1740
GT_A_84_P271180	AT1G53490	0.68	0.1741
GT_A_84_P542948	AT2G46150	1.08	0.1742
GT_A_84_P505192	AT5G21970	0.73	0.1747
GT_A_84_P859555	AtHB23	1.09	0.1748
GT_A_84_P809908	FAD2	0.62	0.1749
GT_A_84_P15996	AT5G64110	2.59	0.1750
GT_At_Specific_00307432	MIR420	2.29	0.1750
GT_A_84_P564195	AT2G42900	1.12	0.1751
GT_A_84_P96816	AtGDU2	3.04	0.1756
GT_A_84_P812218	VHA-A	0.90	0.1759
GT_At_Specific_00023374	AT1G20967.1	2.30	0.1762
GT_A_84_P11746	AT3G27400	1.12	0.1766
GT_A_84_P579880	AT2G40260	0.65	0.1769
GT_A_84_P101346	AT2G44300	1.56	0.1772
GT_A_84_P768357	AT5G55410	2.67	0.1773
GT_A_84_P823836	AT3G55420	0.70	0.1776
GT_A_84_P800349	EG508469	0.99	0.1776
GT_A_84_P21757	AT1G31040	1.39	0.1778
GT_A_84_P825150	AtHB23	1.26	0.1778
GT_A_84_P856374		1.21	0.1778
GT_A_84_P23363	IAA14	1.26	0.1778
GT_At_Specific_00276875	AT5G37017.1	1.66	0.1790
GT_A_84_P229799	AT5G11970	0.79	0.1797
GT_A_84_P836365	AT5G39500	0.77	0.1799
GT_A_84_P302910	AT2G18910	0.62	0.1802
GT_A_84_P13011	AT5G11420	0.98	0.1805
GT_A_84_P13948	AT5G09520	1.11	0.1811
GT_A_84_P834353	AT1G05310	1.79	0.1811
GT_A_84_P21042	ACA4	0.66	0.1812
GT_At_Specific_00162551	AT3G26580.1	1.01	0.1812
GT_A_84_P21013	APL4	1.17	0.1815
GT_A_84_P813910	AT4G18570	1.39	0.1818
GT_A_84_P20116	MTPB1	0.66	0.1819
GT_A_84_P23633	AT1G43910	1.49	0.1822
GT_A_84_P17132	FUT13	0.74	0.1823

GT_A_84_P10606	NP457196	1.00	0.1828
GT_A_84_P764074	FRS1	0.72	0.1831
GT_A_84_P831091	AT2G46980	1.03	0.1831
GT_A_84_P23977	AT3G09010	1.02	0.1834
GT_A_84_P502380	AT4G19970	2.95	0.1836
GT_A_84_P786357	AT5G18860	0.98	0.1837
GT_A_84_P20961	AT1G33440	0.90	0.1840
GT_A_84_P18949	AT1G04610	1.14	0.1841
GT_A_84_P817529	AT2G45600	0.97	0.1855
GT_A_84_P750360	AT1G47860	0.84	0.1867
GT_A_84_P22906	AT2G42560	4.57	0.1870
GT_A_84_P21916	FMO1	1.91	0.1870
GT_A_84_P763290	WRKY53	1.07	0.1871
GT_A_84_P15113	AT1G71520	1.51	0.1871
GT_A_84_P857389		1.28	0.1873
GT_A_84_P13609	AT1G26580	0.68	0.1875
GT_A_84_P868861	FAD3	0.69	0.1881
GT_A_84_P18265	AT2G18480	1.91	0.1894
GT_A_84_P20644	AT5G42840	1.09	0.1895
GT_A_84_P850105	AT5G66450	1.00	0.1900
GT_A_84_P785407	AT1G76960	1.23	0.1900
GT_A_84_P20256	AtMYB10	1.44	0.1903
GT_A_84_P12429	AT1G63820	0.89	0.1904
GT_A_84_P814858	AT3G60340	0.65	0.1906
GT_A_84_P22191	AT3G43670	1.03	0.1906
GT_A_84_P79045	AT1G06980	0.85	0.1908
GT_A_84_P819521	IGPD	0.62	0.1909
GT_A_84_P806341	AGP9	1.00	0.1914
GT_A_84_P836979	AT5G45380	1.01	0.1914
GT_A_84_P13900	CYP96A12	1.54	0.1917
GT_A_84_P751926	AT1G04010	0.75	0.1917
GT_A_84_P192994	NST-K1	0.71	0.1924
GT_A_84_P554482	AT3G03850	3.74	0.1928
GT_A_84_P751997	AT1G15630	3.02	0.1933
GT_A_84_P832713	TA49873_3702	0.72	0.1936
GT_A_84_P23604	AT1G54890	1.03	0.1937
GT_A_84_P841021	AT5G59410	0.66	0.1947
GT_A_84_P793408	AT1G74670	0.75	0.1948
GT_A_84_P12193	AT5G59230	1.16	0.1948
GT_A_84_P815625	TA31749_3702	1.69	0.1948
GT_A_84_P819781	MCA1	0.89	0.1962
GT_A_84_P13124	AT5G55340	2.07	0.1964
GT_A_84_P591196	AT4G22560	1.15	0.1965
GT_A_84_P806743	CAT3	1.39	0.1966
GT_A_84_P102716	AT4G18630	1.60	0.1966
GT_A_84_P810533	CRA1	1.03	0.1968
GT_A_84_P856143	AT5G19920	0.76	0.1971

GT_A_84_P15766	ATH1	1.20	0.1973
GT_A_84_P847999	AtGUS3	1.02	0.1977
GT_A_84_P297924	AT3G06210	0.90	0.1978
GT_A_84_P191874	ORP4B	0.91	0.1992
GT_A_84_P753820	AT1G80745	0.97	0.1994
GT_A_84_P798380	EG495126	1.13	0.1999
GT_A_84_P16938	AT5G63190	1.32	0.2000
GT_A_84_P21399	AT1G27040	1.33	0.2005
GT_A_84_P708146	AT1G01453	1.84	0.2005
GT_At_Specific_00097116	CKX2	1.40	0.2010
GT_A_84_P14634	AT3G46190	1.38	0.2011
GT_At_Specific_00292766	ATFRO8	0.83	0.2012
GT_A_84_P562069	AT1G05370	1.16	0.2013
GT_At_Specific_00147556	AT3G14220.1	1.17	0.2015
GT_A_84_P181704	PC-MYB1	0.83	0.2015
GT_A_84_P242433	AtRLP42	1.79	0.2015
GT_A_84_P840864	NP221927	2.51	0.2021
GT_A_84_P850662	AT5G13660	1.27	0.2032
GT_A_84_P754686	AT1G71528	0.82	0.2036
GT_A_84_P18560	AT4G23240	1.04	0.2037
GT_A_84_P735589	AK220995	0.90	0.2042
GT_A_84_P822511	AT1G75140	0.67	0.2051
GT_A_84_P853578	PRXR1	1.32	0.2057
GT_A_84_P798312	AT1G10120	0.92	0.2057
GT_A_84_P836062	AT1G10600	1.22	0.2059
GT_A_84_P11516	AT1G20380	0.82	0.2066
GT_A_84_P541664	PROPEP3	1.36	0.2070
GT_A_84_P16676	AMY1	1.63	0.2076
GT_A_84_P504549	AT1G23465	1.11	0.2077
GT_At_Specific_00123313	AT2G41178.1	0.87	0.2081
GT_A_84_P787266	WRKY50	2.27	0.2085
GT_A_84_P177574	AtRLP39	2.64	0.2087
GT_A_84_P506400	AT1G63130	1.29	0.2092
GT_A_84_P572348	AT3G47100	1.94	0.2096
GT_A_84_P804445	AT3G44760	2.40	0.2099
GT_A_84_P20799	GPAT2	1.01	0.2104
GT_A_84_P768073	AT5G51845	1.35	0.2107
GT_A_84_P12769	AT3G51990	0.65	0.2123
GT_A_84_P532052	AT1G30757	1.01	0.2125
GT_A_84_P11248	AT5G59220	1.57	0.2125
GT_A_84_P753563	AT1G58590	0.94	0.2129
GT_A_84_P13698	NP033929	1.07	0.2129
GT_A_84_P846712	ATEXO70F1	1.05	0.2134
GT_A_84_P205588	AT4G35880	0.99	0.2134
GT_A_84_P713061	AT5G01542	0.64	0.2135
GT_A_84_P10023	AT1G17430	0.82	0.2136
GT_At_Specific_00309899	PROPEP1	0.96	0.2136

GT_A_84_P14274	AT1G52690	2.14	0.2137
GT_A_84_P784528	SPT	0.97	0.2145
GT_A_84_P756101	AT2G04260	2.14	0.2146
GT_A_84_P826500	TA42420_3702	1.05	0.2151
GT_A_84_P13344	MLO4	1.08	0.2152
GT_A_84_P19128	WRKY43	0.97	0.2153
GT_A_84_P588546	R30196	0.93	0.2167
GT_A_84_P19794	ATMYB66	1.09	0.2169
GT_A_84_P571059	AT1G22680	1.39	0.2181
GT_A_84_P18091	DR750066	1.98	0.2186
GT_A_84_P849282	BX831997	0.85	0.2191
GT_A_84_P570365	AT2G23970	1.37	0.2199
GT_A_84_P15201	ATPAO4	0.67	0.2203
GT_A_84_P858318	AT1G15910	0.88	0.2207
GT_A_84_P834310	TA51263_3702	1.06	0.2215
GT_A_84_P613591	BIM3	0.97	0.2215
GT_A_84_P272980	AT5G18080	2.60	0.2227
GT_At_Specific_00287576	AT5G45790.1	2.45	0.2228
GT_A_84_P539632	AT4G13990	0.67	0.2228
GT_A_84_P838326	AT1G63670	0.69	0.2230
GT_A_84_P860467	AT1G22180	0.60	0.2260
GT_A_84_P53580	AtMYB82	1.18	0.2260
GT_A_84_P846903	ASP4	0.89	0.2262
GT_A_84_P12725	AT3G28510	3.57	0.2264
GT_A_84_P19769	AT5G61570	1.01	0.2264
GT_A_84_P22692	ATB_BETA	0.69	0.2265
GT_A_84_P550058	AT3G02240	1.19	0.2268
GT_A_84_P835314	FRS1	0.80	0.2268
GT_A_84_P15881	HDA2	0.80	0.2275
GT_A_84_P186444	TET10	1.37	0.2276
GT_A_84_P19729	OLEO3	0.91	0.2285
GT_A_84_P18774	FKBP15-2	0.66	0.2285
GT_A_84_P750177	AY090969	1.00	0.2294
GT_A_84_P23964	AT2G31980	1.68	0.2301
GT_A_84_P757088	AT2G48075	1.36	0.2308
GT_A_84_P14569	ADOF2	1.53	0.2314
GT_A_84_P803356	AtUGT85A4	1.32	0.2318
GT_A_84_P794087	NSF	0.90	0.2322
GT_A_84_P808407	TA27519_3702	1.49	0.2329
GT_A_84_P17657	PC-MYB1	1.28	0.2339
GT_A_84_P18822	MYB28	0.85	0.2342
GT_A_84_P842660	ACS3	1.78	0.2344
GT_A_84_P505887	hemf2	0.84	0.2348
GT_A_84_P18336	AT1G26590	1.37	0.2351
GT_A_84_P83469	PROPEP2	2.35	0.2351
GT_A_84_P13653	AT3G14220	1.18	0.2353
GT_A_84_P856904	N38085	1.01	0.2357

GT_A_84_P18275	AT1G07440	0.87	0.2359
GT_A_84_P21393	AT1G27030	0.90	0.2373
GT_A_84_P834278	AT1G62930	1.00	0.2381
GT_A_84_P796648	AT1G76610	1.73	0.2383
GT_A_84_P15864	AT5G18470	1.45	0.2386
GT_A_84_P548023	ATPP2-A9	1.17	0.2386
GT_A_84_P829940	TA47055_3702	0.60	0.2388
GT_A_84_P792412	DQ108771	1.20	0.2393
GT_A_84_P811782	AT2G31980	1.45	0.2396
GT_A_84_P21241	scpl16	1.49	0.2403
GT_A_84_P589324	AT3G61840	1.07	0.2407
GT_A_84_P121222	AT3G21330	1.71	0.2422
GT_A_84_P846733	TC385853	2.12	0.2429
GT_A_84_P757886	AT2G36792	0.82	0.2435
GT_At_Specific_00256424	AT5G14230.1	1.61	0.2444
GT_A_84_P22136	AHP4	2.63	0.2445
GT_A_84_P145159	AT5G50360	1.47	0.2451
GT_A_84_P769532	AT5G08391	1.08	0.2454
GT_A_84_P563858	AT4G38340	1.13	0.2462
GT_A_84_P12353	AtHB23	1.26	0.2472
GT_A_84_P753559	AT1G58590	0.88	0.2473
GT_A_84_P816154	PAP26	0.85	0.2474
GT_A_84_P10391	AT3G31406	1.39	0.2477
GT_A_84_P833567	AT5G04860	0.78	0.2483
GT_A_84_P798395	AT1G59865	2.19	0.2486
GT_A_84_P21131	AT2G25520	0.64	0.2497
GT_A_84_P20172	CYP710A2	1.31	0.2500
GT_A_84_P19160	ATPRB1	3.63	0.2505
GT_A_84_P14808	AT4G30140	2.75	0.2508
GT_A_84_P186714	NP454247	0.96	0.2511
GT_A_84_P14563	ATMYB26	0.82	0.2513
GT_A_84_P837395	DAR7	0.89	0.2522
GT_A_84_P831588	TA49044_3702	0.98	0.2524
GT_A_84_P11349	AT1G59550	1.30	0.2541
GT_A_84_P22056	VIT1	0.99	0.2547
GT_At_Specific_00001969	AT1G02670.1	0.76	0.2550
GT_A_84_P761298	AT3G05727	2.38	0.2582
GT_A_84_P22784	KNATM	1.14	0.2590
GT_A_84_P784432	CYP707A1	1.23	0.2604
GT_A_84_P560726	AT3G13980	1.46	0.2606
GT_A_84_P12672	AT3G15680	0.66	0.2607
GT_A_84_P607185	CBL6	1.10	0.2613
GT_A_84_P23910	ATGSTZ2	1.19	0.2615
GT_A_84_P13998	XGD1	1.12	0.2618
GT_A_84_P309733	AT5G16350	1.13	0.2620
GT_A_84_P123602	AT3G59570	1.10	0.2625
GT_A_84_P833504	UPS2	1.02	0.2625

GT_A_84_P22720	2A6	0.71	0.2627
GT_A_84_P21478	AT4G16640	1.11	0.2635
GT_A_84_P862059	BX831997	1.15	0.2644
GT_A_84_P816349	CRT3	0.99	0.2650
GT_A_84_P20622	AT5G36260	0.62	0.2651
GT_A_84_P576059	AT4G31260	1.07	0.2654
GT_A_84_P788789	RTFL3	0.95	0.2661
GT_A_84_P833516	AT4G17660	1.19	0.2664
GT_A_84_P835146	AT4G34480	0.84	0.2667
GT_A_84_P513574	LURP1	2.24	0.2671
GT_A_84_P14974	AT5G44460	0.63	0.2677
GT_A_84_P610036	AT5G38310	1.27	0.2685
GT_At_Specific_00136716	AT3G05110.1	1.01	0.2698
GT_At_Specific_00076071	AT1G76960.1	1.91	0.2700
GT_A_84_P816533	TA32390_3702	1.28	0.2707
GT_A_84_P837037	AT4G34480	1.06	0.2720
GT_A_84_P790935	AT5G56610	1.25	0.2753
GT_A_84_P20997	ATCSLD6	2.02	0.2756
GT_A_84_P815258	AT4G25690	0.85	0.2765
GT_A_84_P819731	AT4G32290	0.92	0.2776
GT_A_84_P758007	AT2G27160	0.75	0.2778
GT_A_84_P826986	CRK6	3.16	0.2783
GT_A_84_P14685	AT3G57730	1.25	0.2785
GT_A_84_P799071	EG521973	1.22	0.2786
GT_A_84_P857969	CYP91A2	1.59	0.2788
GT_At_Specific_00235724	AT4G34480.1	0.88	0.2792
GT_A_84_P17003	AT1G05930	1.55	0.2792
GT_A_84_P834838	PSF2	0.98	0.2793
GT_A_84_P831079	TA48622_3702	1.55	0.2800
GT_A_84_P21157	AT3G07120	0.84	0.2809
GT_A_84_P21741	AT1G15900	0.62	0.2817
GT_A_84_P835967	AT1G03560	0.77	0.2824
GT_A_84_P22964	AtPP2-B10	0.66	0.2825
GT_A_84_P836351	AT3G19230	0.78	0.2829
GT_A_84_P206748	TA37232_3702	4.38	0.2847
GT_A_84_P23229	anac067	0.94	0.2870
GT_A_84_P829110	AT4G30350	0.66	0.2880
GT_A_84_P201968	AT2G18100	0.73	0.2882
GT_A_84_P21605	AT5G46830	1.65	0.2882
GT_A_84_P760665	AT3G55310	1.04	0.2882
GT_A_84_P14410	AtRLP28	1.91	0.2885
GT_A_84_P20470	AT1G21910	1.17	0.2889
GT_A_84_P11141	AT5G19090	0.76	0.2903
GT_A_84_P818622	AT3G22600	1.24	0.2914
GT_A_84_P62640	CYP71A24	0.91	0.2917
GT_A_84_P833240	ATEXO70D1	2.11	0.2922
GT_A_84_P824517	TA40021_3702	2.22	0.2924

GT_A_84_P14621	**SULTR4;2**	**0.65**	**0.2928**
GT_A_84_P14060	**AT5G52860**	**1.30**	**0.2934**
GT_A_84_P844518	**AT2G20950**	**0.95**	**0.2943**
GT_A_84_P788106	**AT2G37390**	**1.35**	**0.2945**
GT_A_84_P16729	**ACS8**	**0.84**	**0.2948**
GT_A_84_P11917	**AT4G13180**	**0.63**	**0.2951**
GT_A_84_P858209	**AT2G03480**	**0.67**	**0.2951**
GT_A_84_P259850	**AT2G41550**	**0.69**	**0.2952**
GT_A_84_P815049	**AT1G76010**	**1.46**	**0.2953**
GT_A_84_P800343	**EG508437**	**0.88**	**0.2955**
GT_A_84_P17467	**AT3G45420**	**1.06**	**0.2957**
GT_A_84_P803776	**AtRLP44**	**0.73**	**0.2960**
GT_A_84_P22958	**SLP3**	**0.64**	**0.2962**
GT_A_84_P565558	**AT5G56610**	**1.26**	**0.2963**
GT_A_84_P51740	**AT2G15760**	**1.25**	**0.2974**
GT_A_84_P12605	**AT2G18010**	**2.79**	**0.2979**
GT_A_84_P715622		**0.93**	**0.2980**
GT_A_84_P13287	**AT1G75580**	**0.91**	**0.2986**
GT_A_84_P793206	**AAP5**	**0.82**	**0.2989**
GT_A_84_P784866	**GA3OX1**	**1.54**	**0.2997**
GT_A_84_P798916	**AT4G22520**	**1.74**	**0.3003**
GT_A_84_P14281	**GA3OX1**	**1.64**	**0.3007**
GT_A_84_P587403	**AT3G05110**	**1.52**	**0.3011**
GT_A_84_P13964	**AT5G14230**	**1.27**	**0.3012**
GT_A_84_P13755	**AT3G61610**	**0.89**	**0.3014**
GT_A_84_P764950	**AT4G22517**	**1.90**	**0.3023**
GT_A_84_P823153	**AT2G37050**	**0.96**	**0.3023**
GT_A_84_P12091	**AT5G23400**	**0.93**	**0.3025**
GT_A_84_P12421	**ASP4**	**0.86**	**0.3033**
GT_A_84_P14437	**XERICO**	**1.04**	**0.3035**
GT_A_84_P855383	**MSL10**	**0.66**	**0.3045**
GT_At_Specific_00172615	**AT3G44716.1**	**0.80**	**0.3046**
GT_A_84_P807739	**TA27151_3702**	**1.35**	**0.3049**
GT_A_84_P510821	**AT1G54680**	**0.77**	**0.3052**
GT_A_84_P18955	**AT1G30850**	**2.19**	**0.3055**
GT_A_84_P831305	**AT5G66420**	**0.66**	**0.3056**
GT_A_84_P16087	**AT1G61500**	**0.78**	**0.3057**
GT_A_84_P188254	**CRK6**	**3.10**	**0.3061**
GT_A_84_P511531	**AT5G65570**	**0.69**	**0.3062**
GT_A_84_P521926	**AT4G22510**	**1.84**	**0.3073**
GT_A_84_P108542	**AT2G41180**	**1.41**	**0.3078**
GT_A_84_P830388	**AT1G04500**	**1.16**	**0.3086**
GT_At_Specific_00027730	**AT1G24825.2**	**1.24**	**0.3090**
GT_A_84_P21104	**AT2G19550**	**1.94**	**0.3098**
GT_A_84_P539380	**AT2G35075**	**0.69**	**0.3105**
GT_A_84_P200454	**PDLP6**	**1.10**	**0.3107**
GT_A_84_P860899	**BX837858**	**1.46**	**0.3109**

GT_A_84_P759141	AT3G44716	0.71	0.3124
GT_A_84_P799914	AT3G05640	0.85	0.3124
GT_A_84_P12310	AT1G76960	2.09	0.3136
GT_A_84_P786411	AF418241	1.84	0.3143
GT_A_84_P822418	AT1G27370	1.15	0.3143
GT_At_Specific_00019644	AT1G17640.1	0.77	0.3160
GT_A_84_P10678	AT2G28050	1.29	0.3162
GT_A_84_P20361	SPL15	0.75	0.3166
GT_A_84_P574554	AT1G06475	1.22	0.3170
GT_A_84_P131106	AT3G02910	0.81	0.3171
GT_A_84_P797957	AT5G40380	1.12	0.3175
GT_A_84_P16574	BGL2	2.19	0.3194
GT_A_84_P835401	AT3G54440	0.92	0.3204
GT_A_84_P23480	AT5G42280	0.80	0.3210
GT_A_84_P862797	AT4G23670	1.46	0.3217
GT_A_84_P191364	AT5G18870	0.88	0.3229
GT_A_84_P14560	PAD3	2.84	0.3229
GT_A_84_P843360	NP396398	1.38	0.3232
GT_A_84_P730499	EG479048	2.68	0.3236
GT_At_Specific_00118768	AT2G37390.1	1.11	0.3239
GT_A_84_P21805	XIE	1.17	0.3260
GT_A_84_P797145	EG439403	1.03	0.3265
GT_A_84_P524277	AT2G27775	1.58	0.3273
GT_A_84_P837219	AT5G19090	1.31	0.3276
GT_At_Specific_00252235	FLC	1.53	0.3280
GT_A_84_P20114	AtRLP23	3.06	0.3286
GT_A_84_P868134	AT4G25690	0.75	0.3294
GT_A_84_P264760	AT2G29920	2.49	0.3297
GT_A_84_P18661	CYP77A4	1.89	0.3299
GT_A_84_P587896	OFP12	2.24	0.3320
GT_A_84_P13778	AF418241	1.98	0.3326
GT_A_84_P553387	AT1G64405	1.76	0.3326
GT_A_84_P13615	AT3G26820	0.94	0.3335
GT_A_84_P847681	AT3G08680	1.05	0.3336
GT_A_84_P13764	AT3G44830	2.46	0.3353
GT_A_84_P580199	DML1	1.07	0.3360
GT_A_84_P805318	TA25943_3702	1.75	0.3365
GT_A_84_P255130	AT1G04800	1.00	0.3370
GT_A_84_P125601	AT3G22240	1.76	0.3371
GT_A_84_P580314	AT3G22235	1.80	0.3376
GT_A_84_P137779	AT4G30180	1.80	0.3398
GT_A_84_P521606	AT2G25260	1.15	0.3445
GT_A_84_P150658	DAR7	0.85	0.3446
GT_A_84_P11642	AT2G24170	0.64	0.3449
GT_A_84_P718723	AT3G57700	0.67	0.3450
GT_A_84_P547455	ILR2	1.96	0.3450
GT_A_84_P508506	AT3G55820	0.74	0.3462

GT_A_84_P19994	ACA7	1.93	0.3462
GT_A_84_P816631	AT2G17500	1.42	0.3464
GT_A_84_P844205	AT4G23160	1.08	0.3465
GT_A_84_P787487	AT4G23790	0.75	0.3465
GT_A_84_P710653	TC401718	0.84	0.3474
GT_A_84_P14803	AT4G29020	1.79	0.3477
GT_A_84_P534291	AT4G09300	1.02	0.3478
GT_A_84_P281450	WAK2	1.63	0.3483
GT_A_84_P10170	FLC	1.19	0.3488
GT_A_84_P857742	BX839026	1.31	0.3504
GT_A_84_P266040	AT4G02420	1.28	0.3511
GT_A_84_P544532	AT1G58225	2.00	0.3519
GT_A_84_P817089	TA32832_3702	1.10	0.3530
GT_A_84_P10163	GRP20	2.51	0.3530
GT_A_84_P535181	CLE44	1.48	0.3531
GT_A_84_P598130	PSF2	0.71	0.3533
GT_A_84_P789302	KDR	1.15	0.3544
GT_A_84_P274450	AT5G26930	1.15	0.3554
GT_A_84_P12326	COBL6	0.65	0.3561
GT_A_84_P220478	CRT3	0.80	0.3565
GT_A_84_P826544	GAE3	0.67	0.3571
GT_A_84_P135335	AT3G22104	0.64	0.3573
GT_A_84_P10025	IQD7	0.73	0.3583
GT_At_Specific_00073457	ATGSTU10	0.97	0.3595
GT_A_84_P24057	AT3G19620	1.06	0.3595
GT_At_Specific_00226505	AT4G26795.2	0.91	0.3602
GT_A_84_P267240	ATEXO70H2	0.91	0.3603
GT_A_84_P761372	IDL4	0.90	0.3604
GT_A_84_P509286	AT3G10290	1.50	0.3605
GT_A_84_P13496	AT2G25150	1.71	0.3607
GT_A_84_P194774	RGXT2	1.05	0.3619
GT_A_84_P21261	AT3G47540	1.00	0.3619
GT_A_84_P834362	EIN4	1.01	0.3619
GT_A_84_P12863	HAK5	3.20	0.3639
GT_At_Specific_00068346	CLE26	1.20	0.3647
GT_At_Specific_00228561	AT4G28550.1	0.73	0.3651
GT_A_84_P822194	BCDH_BETA1	1.00	0.3653
GT_A_84_P12843	AT4G08370	1.46	0.3654
GT_A_84_P13831	AT4G23150	2.23	0.3656
GT_A_84_P784210	FLC	1.20	0.3664
GT_A_84_P12432	GSTU10	0.70	0.3668
GT_A_84_P502971	AT2G25290	0.66	0.3674
GT_A_84_P152798	AT5G18030	1.87	0.3684
GT_A_84_P788445	AT2G20950	0.84	0.3688
GT_A_84_P15382	CYP71A13	3.76	0.3693
GT_A_84_P851659	CYP71B37	1.72	0.3707
GT_A_84_P17047	ZFP1	1.59	0.3714

GT_A_84_P230389	AT3G21080	1.37	0.3737
GT_A_84_P171963	AT2G39410	0.93	0.3747
GT_A_84_P11973	HMA2	0.73	0.3749
GT_A_84_P839084	AT1G61440	0.76	0.3749
GT_A_84_P789698	AT1G72416	0.75	0.3750
GT_A_84_P797437	AT1G24270	0.85	0.3756
GT_A_84_P19102	AT1G51940	0.76	0.3766
GT_A_84_P853277	ANL2	0.74	0.3774
GT_A_84_P19721	AT5G48800	0.68	0.3782
GT_A_84_P571568	AT4G19370	0.71	0.3794
GT_A_84_P550387	AT5G13250	1.56	0.3807
GT_A_84_P18477	BGLU27	3.86	0.3811
GT_A_84_P811778	AT2G28840	1.32	0.3819
GT_A_84_P136365	AT5G39450	0.83	0.3829
GT_A_84_P857638	CYP71A13	3.64	0.3844
GT_A_84_P22552	AT5G47130	0.75	0.3848
GT_A_84_P18118	NAS4	1.96	0.3862
GT_A_84_P767789	AT5G53750	0.76	0.3863
GT_A_84_P541510	AT5G10890	0.70	0.3866
GT_A_84_P829552	TA46474_3702	0.76	0.3885
GT_A_84_P22341	AT4G22520	1.62	0.3886
GT_A_84_P759689	AT3G44262	1.35	0.3905
GT_A_84_P824914	AT4G18205	1.09	0.3907
GT_A_84_P21578	AT5G39670	1.11	0.3910
GT_A_84_P852753	CPuORF46	0.93	0.3911
GT_A_84_P564018	AT1G63080	0.66	0.3921
GT_A_84_P14999	AT1G64370	0.64	0.3921
GT_A_84_P834809	scpl16	0.65	0.3926
GT_A_84_P593798	AT3G61660	1.09	0.3937
GT_At_Specific_00110620	CYP71A13	3.73	0.3947
GT_A_84_P785972	CB185526	2.05	0.3962
GT_A_84_P510027	AT2G04495	3.29	0.3968
GT_A_84_P863309	BP667362	1.34	0.3976
GT_A_84_P147028	AT5G18020	1.70	0.3977
GT_A_84_P754785	CLE26	1.21	0.3992
GT_A_84_P853269	FUC95A	0.99	0.3995
GT_A_84_P818385	AT3G16270	0.62	0.3995
GT_A_84_P18175	PE11	0.68	0.4000
GT_A_84_P220358	AT5G50610	1.04	0.4002
GT_A_84_P803918	APUM2	0.87	0.4034
GT_A_84_P843773	NP502379	0.75	0.4039
GT_A_84_P784401	FLC	1.31	0.4048
GT_A_84_P20339	AT3G53210	1.02	0.4052
GT_A_84_P795916	MIF3	0.99	0.4057
GT_A_84_P608670	AT2G28580	0.81	0.4063
GT_A_84_P218478	AT1G74300	0.94	0.4077
GT_A_84_P10161	AT5G07260	0.82	0.4082

GT_A_84_P89649	CYP86C2	1.23	0.4083
GT_A_84_P768397	IDL2	1.33	0.4087
GT_A_84_P843146	ATMRP12	0.71	0.4091
GT_A_84_P13119	AT5G54020	0.76	0.4092
GT_A_84_P512146	AT4G20300	0.78	0.4096
GT_A_84_P764946	AT4G22505	3.19	0.4096
GT_A_84_P11669	UPS2	0.80	0.4105
GT_A_84_P784626	CNBT1	0.73	0.4130
GT_A_84_P195614	AT5G23840	0.97	0.4148
GT_A_84_P595309	AT2G18970	1.39	0.4148
GT_A_84_P820052	EXPA5	1.27	0.4165
GT_A_84_P764548	PUP7	0.94	0.4198
GT_At_Specific_00251546	AT5G09560.1	1.72	0.4208
GT_A_84_P788113	CLE41	0.69	0.4216
GT_A_84_P764941	AT4G22485	2.73	0.4235
GT_A_84_P842676	AT5G24790	0.95	0.4237
GT_A_84_P23768	NCED5	2.03	0.4249
GT_A_84_P20780	AT1G03495	3.27	0.4254
GT_A_84_P812910	AT3G22235	2.43	0.4257
GT_A_84_P760741	AtRLP34	2.40	0.4260
GT_At_Specific_00288320	AT5G46417.1	0.72	0.4265
GT_A_84_P751876	AT1G11803	1.57	0.4266
GT_At_Specific_00127560	AT2G44600.1	0.82	0.4274
GT_A_84_P592920	AT4G01390	2.77	0.4275
GT_A_84_P21135	AT3G03820	1.88	0.4281
GT_A_84_P559351	AT5G55460	2.03	0.4282
GT_A_84_P10135	ROT3	0.72	0.4285
GT_A_84_P16649	NF-YA8	0.79	0.4292
GT_A_84_P22510	AT5G28230	1.05	0.4303
GT_A_84_P711052	AtGH9C2	0.73	0.4353
GT_A_84_P740096	TC398054	1.02	0.4356
GT_A_84_P10444	G6PD4	0.89	0.4360
GT_At_Specific_00012708	MIR171B	0.93	0.4365
GT_A_84_P552861	LBD31	0.66	0.4380
GT_A_84_P536372	DAR3	1.90	0.4380
GT_A_84_P22899	AT2G43850	0.60	0.4398
GT_A_84_P10668	AT2G16580	1.59	0.4404
GT_A_84_P76044	AT2G34580	1.03	0.4405
GT_A_84_P16540	AtRLP44	0.63	0.4406
GT_A_84_P513074	AT4G32970	0.73	0.4412
GT_A_84_P542760	TC397717	0.76	0.4437
GT_A_84_P827741	AtMYB10	0.84	0.4447
GT_A_84_P281930	AT3G47820	1.05	0.4461
GT_A_84_P790950	ATUPS4	1.33	0.4467
GT_A_84_P824477	TA39987_3702	1.03	0.4495
GT_A_84_P10043	AT4G21230	0.83	0.4495
GT_A_84_P530104	AT5G52740	1.44	0.4500

GT_A_84_P795901	DQ108699	0.68	0.4511
GT_At_Specific_00222201	AT4G23200.1	1.51	0.4514
GT_A_84_P22681	AtRLP11	2.14	0.4528
GT_A_84_P11795	MLO3	0.68	0.4532
GT_A_84_P12139	AT5G44400	0.82	0.4554
GT_At_Specific_00109086	AT2G29452.1	1.22	0.4556
GT_A_84_P764634	EF182887	1.30	0.4570
GT_A_84_P12757	AT3G49220	1.01	0.4575
GT_A_84_P836180	AtRLP23	3.55	0.4580
GT_A_84_P610349	IDA	1.19	0.4584
GT_A_84_P813284	ANL2	0.82	0.4601
GT_A_84_P10030	anac071	0.95	0.4608
GT_A_84_P250315	EXLB3	2.07	0.4618
GT_A_84_P23208	AT3G60420	1.15	0.4619
GT_A_84_P21760	AT1G03940	2.60	0.4625
GT_A_84_P274500	AT1G77880	1.04	0.4626
GT_A_84_P529896	AT4G23790	0.92	0.4639
GT_A_84_P834243	AT5G17040	0.70	0.4640
GT_A_84_P750331	AT1G70340	0.74	0.4644
GT_A_84_P13861	AT4G29650	0.64	0.4658
GT_A_84_P758488	AT2G18969	2.63	0.4686
GT_A_84_P137649	WAK1	1.58	0.4688
GT_A_84_P845837	AT1G58602	0.91	0.4692
GT_A_84_P16322	AT2G14620	0.75	0.4712
GT_A_84_P795819	DQ108857	0.89	0.4723
GT_A_84_P853555	AT1G18020	1.08	0.4724
GT_A_84_P540652	AT5G15900	1.96	0.4736
GT_A_84_P856227	BE039478	1.65	0.4757
GT_A_84_P123822	SNZ	1.22	0.4757
GT_A_84_P757642	AT2G20605	0.95	0.4760
GT_A_84_P15376	anac042	1.81	0.4768
GT_A_84_P852904	SCR	1.38	0.4798
GT_A_84_P16270	AT1G33610	1.93	0.4801
GT_A_84_P18535	MHK	0.73	0.4814
GT_A_84_P15524	AtRLP38	2.15	0.4816
GT_A_84_P579146	AT1G58602	0.75	0.4817
GT_A_84_P17706	AT5G01870	1.42	0.4825
GT_A_84_P752095	AT1G35270	0.77	0.4843
GT_A_84_P822777	AT5G44670	0.83	0.4849
GT_A_84_P750195	AT1G73550	0.61	0.4850
GT_A_84_P10905	AT3G57630	1.15	0.4853
GT_A_84_P109962	WAK3	2.36	0.4866
GT_A_84_P758398	AT2G09992	0.81	0.4874
GT_A_84_P16510	AT1G35710	1.98	0.4879
GT_A_84_P827553	SNZ	1.33	0.4883
GT_A_84_P17627	MEK1	0.71	0.4890
GT_At_Specific_00086614	AT2G06562.1	0.69	0.4891

GT_A_84_P819992	AT4G32340	0.67	0.4893
GT_A_84_P824668	MYB106	0.91	0.4895
GT_A_84_P240505	AT2G27770	0.68	0.4895
GT_A_84_P18719	AT1G68630	1.58	0.4901
GT_A_84_P68884	AT4G21840	2.29	0.4902
GT_A_84_P83519	AT1G62225	2.83	0.4937
GT_A_84_P23274	AT1G29290	0.71	0.4951
GT_A_84_P803729		1.03	0.4966
GT_A_84_P813238	ANL2	0.65	0.4987
GT_A_84_P239215	PDF1.3	1.41	0.5003
GT_A_84_P750319	AT1G19630	1.42	0.5011
GT_A_84_P16668	AT4G23200	1.40	0.5013
GT_A_84_P582082	AT3G16370	0.78	0.5023
GT_A_84_P127531	ATEXT3	0.77	0.5053
GT_A_84_P74244	NP453663	1.09	0.5067
GT_A_84_P786204	ATEXT3	1.33	0.5067
GT_A_84_P305850	GAD4	1.74	0.5099
GT_A_84_P196694	PDF1.2c	1.41	0.5113
GT_A_84_P16678	GA20OX1	1.02	0.5137
GT_A_84_P19713	AT1G29450	1.46	0.5146
GT_A_84_P845705	CYP722A1	1.59	0.5151
GT_A_84_P804807	AIR12	0.80	0.5154
GT_A_84_P170243	AT2G44600	0.72	0.5170
GT_A_84_P13279	AT1G09550	0.60	0.5176
GT_A_84_P287700	AT5G38940	1.59	0.5180
GT_A_84_P509388	AT3G52520	0.89	0.5233
GT_A_84_P18244	AT2G35910	0.69	0.5233
GT_A_84_P80109	AT5G40630	1.60	0.5295
GT_A_84_P23646	BCA3	2.21	0.5299
GT_A_84_P16436	FRD3	0.80	0.5325
GT_A_84_P803664	ATCCS	0.95	0.5331
GT_A_84_P12144	AT5G45720	0.65	0.5331
GT_A_84_P10178	AT5G12050	1.00	0.5346
GT_A_84_P599862	AT2G46640	1.11	0.5348
GT_A_84_P162133	AT4G32290	0.89	0.5351
GT_A_84_P843355	GRP20	2.16	0.5362
GT_A_84_P297344	AT5G23240	1.47	0.5371
GT_A_84_P20823	SCPL32	1.25	0.5402
GT_A_84_P11590	AT2G23200	0.78	0.5405
GT_A_84_P21489	AT5G01720	0.73	0.5416
GT_A_84_P601105	OFP8	0.95	0.5428
GT_A_84_P820887	AT3G53190	1.34	0.5428
GT_At_Specific_00024963	AT1G22280.2	0.77	0.5480
GT_A_84_P137009	PDF1.2	1.85	0.5485
GT_A_84_P14443	AT2G29310	1.06	0.5487
GT_A_84_P19804	AT5G19880	1.33	0.5492
GT_A_84_P14014	AT5G40210	1.27	0.5492

GT_A_84_P516993	AT1G30515	0.63	0.5498
GT_A_84_P220458	AT4G23610	1.19	0.5505
GT_A_84_P565178	NIMIN-2	1.27	0.5513
GT_At_Specific_00108922	AT2G29310.1	0.97	0.5528
GT_A_84_P813638	AT1G75300	0.98	0.5535
GT_At_Specific_00017282	PDR12	1.74	0.5543
GT_A_84_P561111	AT5G50890	0.88	0.5556
GT_A_84_P91079	AT5G22520	0.92	0.5558
GT_A_84_P24121	AT3G52900	0.74	0.5582
GT_A_84_P12680	EXPA5	1.37	0.5584
GT_A_84_P63594	AT4G18205	1.00	0.5631
GT_A_84_P507242	TLL1	1.13	0.5647
GT_A_84_P751516	ATMRP12	1.05	0.5656
GT_A_84_P544972	AT4G21740	0.91	0.5660
GT_A_84_P542466	AT5G36710	1.51	0.5680
GT_A_84_P838519	AT1G11300	1.33	0.5684
GT_A_84_P22739	AT1G30800	1.69	0.5686
GT_A_84_P160473	AT1G65250	0.76	0.5691
GT_A_84_P790382	YSL5	0.73	0.5701
GT_A_84_P830275	AT5G15010	0.93	0.5702
GT_A_84_P799571	AT1G35710	0.98	0.5706
GT_A_84_P310613	PDF1.2b	1.19	0.5721
GT_A_84_P11446	PDR12	1.76	0.5728
GT_A_84_P255150	AT2G28410	0.84	0.5734
GT_At_Specific_00155620	AT3G20898.1	0.64	0.5761
GT_At_Specific_00175975	AT3G47540.1	1.05	0.5772
GT_A_84_P20045	AT1G57630	1.40	0.5781
GT_A_84_P832028	AT5G62220	0.92	0.5843
GT_A_84_P547587	TC372824	0.62	0.5867
GT_A_84_P787935	PIL1	2.65	0.5870
GT_A_84_P19046	ATGSTU12	1.22	0.5899
GT_A_84_P721757	AT5G61412	1.54	0.5929
GT_A_84_P765529	AT4G15242	0.85	0.5945
GT_A_84_P611298	AT2G04515	1.85	0.5955
GT_A_84_P210278	PXMT1	1.04	0.5955
GT_A_84_P765604	AT4G05018	1.51	0.5966
GT_A_84_P786630	AT4G30060	0.78	0.5985
GT_A_84_P19325	AT3G12870	0.97	0.5991
GT_At_Specific_00282720	MIR166E	1.05	0.6005
GT_A_84_P562126	AT1G13470	1.96	0.6012
GT_A_84_P825275	AT1G61260	0.80	0.6033
GT_A_84_P834386	VCS	1.33	0.6071
GT_A_84_P795628		0.79	0.6084
GT_A_84_P820051	TA35339_3702	1.07	0.6161
GT_A_84_P17847	AT-HSFA9	0.86	0.6166
GT_A_84_P721909	IQD20	1.72	0.6176
GT_A_84_P17179	AT1G75300	1.12	0.6182

GT_A_84_P515014	TED7	0.86	0.6192
GT_A_84_P111252	AT5G47730	0.78	0.6205
GT_A_84_P824281	PXMT1	1.24	0.6232
GT_A_84_P169473	AT5G30500	0.89	0.6261
GT_A_84_P21136	AT3G05190	0.91	0.6277
GT_A_84_P839113	AT1G24145	1.06	0.6301
GT_A_84_P288904	LSU3	0.80	0.6302
GT_A_84_P15036	RLK1	1.26	0.6320
GT_A_84_P817628	JAL23	0.67	0.6333
GT_A_84_P13010	SHN3	1.25	0.6370
GT_A_84_P788748	AT1G13470	1.84	0.6372
GT_At_Specific_00297969	ANK	1.97	0.6384
GT_A_84_P54620	AT5G64780	0.93	0.6419
GT_A_84_P20191	MYB106	0.80	0.6448
GT_A_84_P555074	AT1G24145	0.96	0.6451
GT_A_84_P303670	AT4G34420	0.88	0.6485
GT_A_84_P290004	AT4G21850	1.42	0.6532
GT_A_84_P16903	ANK	2.01	0.6550
GT_A_84_P769485	AT5G60142	0.62	0.6620
GT_A_84_P812152	AT4G21850	1.46	0.6638
GT_A_84_P13202	anac036	0.85	0.6669
GT_A_84_P761666	AT3G59765	0.83	0.6732
GT_A_84_P187524	AT2G47010	0.65	0.6745
GT_A_84_P808136	TA27368_3702	1.83	0.6756
GT_A_84_P10897	CCR3	0.67	0.6876
GT_A_84_P842635	TC404116	1.25	0.6896
GT_A_84_P14954	CPuORF32	0.94	0.6897
GT_A_84_P796323	AT4G15820	0.97	0.6952
GT_A_84_P91669	AT5G62220	0.68	0.6980
GT_A_84_P18312	AK3	1.17	0.7060
GT_A_84_P17028	FMO_GS-OX3	1.10	0.7071
GT_A_84_P21462	AT4G38560	0.64	0.7074
GT_A_84_P828727	TOM20-2	0.68	0.7074
GT_A_84_P24047	AT3G21950	0.93	0.7106
GT_A_84_P10359	AT5G19800	0.66	0.7116
GT_A_84_P808216	TA27415_3702	0.75	0.7125
GT_A_84_P14029	AT5G44440	1.23	0.7207
GT_A_84_P846594	AT5G59050	0.94	0.7316
GT_A_84_P803724	AAP2	0.97	0.7362
GT_A_84_P763927	AT4G01650	0.64	0.7464
GT_A_84_P13407	SOT17	0.80	0.7529
GT_A_84_P820541	AT3G02640	1.16	0.7546
GT_A_84_P275810	AT4G16610	0.72	0.7627
GT_A_84_P14579	AtRLP37	1.25	0.7653
GT_At_Specific_00165144	AT3G28580.1	1.18	0.7658
GT_A_84_P15262	GGT3	0.72	0.7725
GT_A_84_P755699	AT2G34580	0.84	0.7796

GT_A_84_P751365	**TLL1**	**0.82**	**0.7822**
GT_A_84_P765944	**AT4G21903**	**0.73**	**0.7947**
GT_A_84_P835631	**AT3G56410**	**0.72**	**0.8177**
GT_At_Specific_00042921	**ATTLL1**	**0.72**	**0.8216**
GT_A_84_P84999	**AT3G28580**	**1.09**	**0.8389**
GT_A_84_P756849	**AT2G24730**	**0.88**	**0.8408**
GT_A_84_P107372	**AT3G02120**	**0.86**	**0.8634**
GT_A_84_P13779	**CYCD6;1**	**0.61**	**0.8852**
GT_A_84_P20862	**AT1G02030**	**0.61**	**0.9902**

Down-regulated genes in *aba* 1.6

**ProbeName**	**GeneName**	**Geo mean (3 replicate) Log2 Fold Change**	**P_Value**
GT_A_84_P803124		**-1.00**	**0.0001**
GT_A_84_P17960	**FRO2**	**-1.11**	**0.0001**
GT_A_84_P834403	**AT4G09150**	**-1.30**	**0.0001**
GT_A_84_P12402	**CM3**	**-0.59**	**0.0001**
GT_AtCg01250_21	**ndhB.2**	**-0.65**	**0.0002**
GT_A_84_P17372	**PTF1**	**-0.59**	**0.0003**
GT_A_84_P828950	**AT2G46250**	**-0.69**	**0.0003**
GT_A_84_P808919	**PIP1B**	**-0.79**	**0.0004**
GT_A_84_P805209	**AT1G62740**	**-0.80**	**0.0004**
GT_A_84_P700224	**AT2G23672**	**-0.47**	**0.0005**
GT_AntiSense_AtMg01230_1110	**orf145b**	**-0.90**	**0.0006**
GT_A_84_P787157	**AT5G48110**	**-2.21**	**0.0006**
GT_A_84_P96976	**SAG20**	**-0.86**	**0.0006**
GT_A_84_P90179	**ATHVA22E**	**-1.17**	**0.0010**
GT_A_84_P850377	**emb2444**	**-0.71**	**0.0010**
GT_A_84_P814607	**CYP81F4**	**-1.48**	**0.0013**
GT_A_84_P13957	**AT5G11650**	**-0.96**	**0.0013**
GT_A_84_P13897	**AT4G38810**	**-0.57**	**0.0014**
GT_A_84_P15958	**AT5G54940**	**-0.90**	**0.0016**
GT_AntiSense_AtMg00850_918	**orf107e**	**-0.90**	**0.0016**
GT_A_84_P758621	**AT2G01008**	**-1.21**	**0.0017**
GT_A_84_P16756	**AT1G19540**	**-1.05**	**0.0017**
GT_A_84_P785629	**SCA3**	**-0.61**	**0.0018**
GT_AtMg01260_1123	**orf205**	**-0.86**	**0.0018**
GT_A_84_P807670	**TA27079_3702**	**-0.76**	**0.0019**
GT_A_84_P10133	**AT1G19390**	**-0.57**	**0.0019**
GT_A_84_P10395	**ARR7**	**-1.46**	**0.0020**
GT_A_84_P785967	**AT5G54940**	**-0.95**	**0.0020**
GT_A_84_P93449	**AT4G28150**	**-1.33**	**0.0020**
GT_A_84_P292734	**AT2G37520**	**-0.52**	**0.0020**
GT_A_84_P12086	**AT5G19100**	**-1.90**	**0.0021**
GT_A_84_P841048	**NP225641**	**-1.12**	**0.0021**
GT_A_84_P797225		**-0.94**	**0.0021**

GT_A_84_P844993	**TC364679**	**-0.79**	**0.0021**
GT_A_84_P832767	**TOC159**	**-0.51**	**0.0022**
GT_A_84_P799657	**EG496052**	**-1.46**	**0.0022**
GT_A_84_P18619	**CYP81F4**	**-1.64**	**0.0023**
GT_A_84_P809682	**AT5G54940**	**-1.20**	**0.0024**
GT_A_84_P290124	**ATPSK2**	**-1.49**	**0.0025**
GT_A_84_P525160	**AT2G30350**	**-0.63**	**0.0026**
GT_A_84_P767469	**AT5G37270**	**-1.42**	**0.0027**
GT_A_84_P869288	**ATSERAT1;1**	**-0.75**	**0.0027**
GT_A_84_P13014	**AT5G12110**	**-3.14**	**0.0027**
GT_A_84_P11209	**AT5G48110**	**-1.84**	**0.0028**
GT_A_84_P767439	**AT5G37230**	**-1.79**	**0.0028**
GT_A_84_P168793	**AT5G63490**	**-0.59**	**0.0029**
GT_A_84_P10251	**AtPP2-A6**	**-2.17**	**0.0029**
GT_A_84_P810724	**GGT1**	**-0.58**	**0.0030**
GT_A_84_P68904	**ATBETAFRUCT4**	**-0.96**	**0.0030**
GT_A_84_P816133	**TA32113_3702**	**-0.74**	**0.0031**
GT_A_84_P19352	**MEE38**	**-1.34**	**0.0031**
GT_At_Specific_00122443	**LBD15**	**-0.67**	**0.0031**
GT_A_84_P21190	**AT3G16350**	**-0.57**	**0.0031**
GT_A_84_P859300	**ATHSP90.1**	**-2.77**	**0.0032**
GT_A_84_P19328	**ATPREP1**	**-0.55**	**0.0032**
GT_AntiSense_AtMg00450_729	**orf106b**	**-1.29**	**0.0032**
GT_A_84_P22453	**AT1G06540**	**-1.35**	**0.0032**
GT_A_84_P756830	**AT2G07678**	**-0.81**	**0.0033**
GT_A_84_P785840	**AT4G24200**	**-0.92**	**0.0033**
GT_A_84_P823526	**AT5G04250**	**-0.75**	**0.0033**
GT_A_84_P536652	**AT2G13960**	**-0.72**	**0.0035**
GT_At_Specific_00118094	**GAMMA-TIP**	**-0.99**	**0.0036**
GT_A_84_P21063	**ATGPX1**	**-0.79**	**0.0036**
GT_A_84_P76889	**AT5G13720**	**-0.74**	**0.0037**
GT_A_84_P18258	**TPS10**	**-3.76**	**0.0037**
GT_A_84_P582862	**AT2G29790**	**-4.96**	**0.0037**
GT_A_84_P75644	**AT2G05440**	**-3.36**	**0.0038**
GT_A_84_P814616	**CYP81F4**	**-1.72**	**0.0038**
GT_AntiSense_AtCg00890_426	**ndhB.1**	**-0.56**	**0.0039**
GT_A_84_P17562	**GGT1**	**-0.60**	**0.0039**
GT_AntiSense_AtMg00690_877	**orf240a**	**-0.76**	**0.0040**
GT_A_84_P133635	**AT3G12050**	**-1.04**	**0.0040**
GT_A_84_P16049	**AT2G07713**	**-1.28**	**0.0040**
GT_AtMg00300_687	**orf145a**	**-1.06**	**0.0040**
GT_A_84_P12622	**NTL8**	**-1.14**	**0.0041**
GT_A_84_P10453	**AT1G04130**	**-0.66**	**0.0041**
GT_A_84_P831778	**TA49244_3702**	**-0.69**	**0.0041**
GT_A_84_P264710	**AT2G44820**	**-0.66**	**0.0041**
GT_A_84_P12926	**ATGPX7**	**-2.72**	**0.0042**
GT_A_84_P812231	**AT1G03230**	**-0.55**	**0.0044**

GT_A_84_P784302	ATAF1	-0.65	0.0044
GT_A_84_P814174	ATAF1	-0.55	0.0044
GT_A_84_P862741	TC405377	-1.31	0.0044
GT_A_84_P76784	SHV3	-0.50	0.0045
GT_A_84_P807532	GAMMA-TIP	-0.91	0.0047
GT_AntiSense_AtCg01090_493	ndhI	-0.74	0.0047
GT_A_84_P853908	ATPREP1	-0.81	0.0048
GT_A_84_P15946	AT5G51440	-3.44	0.0048
GT_A_84_P591730	ELF4-L3	-1.56	0.0048
GT_A_84_P24127	AT3G53990	-0.61	0.0048
GT_A_84_P15075	ATNUDX19	-0.49	0.0048
GT_A_84_P580100	AT1G77960	-1.65	0.0049
GT_A_84_P839051	NUA	-0.53	0.0050
GT_A_84_P795314	AT4G00810	-0.58	0.0051
GT_A_84_P762878	BRC1	-2.00	0.0051
GT_A_84_P109412	AT4G27350	-1.18	0.0052
GT_A_84_P19986	ATAF1	-0.68	0.0052
GT_A_84_P13031	AT5G19110	-1.89	0.0052
GT_At_Specific_00080749	AT2G01023.1	-1.69	0.0055
GT_A_84_P21609	NF-YC2	-1.63	0.0055
GT_A_84_P789539	RAP2.9	-1.53	0.0055
GT_A_84_P17242	RD28	-1.32	0.0055
GT_A_84_P10688	AT1G30070	-1.78	0.0056
GT_A_84_P20108	CPuORF17	-0.92	0.0057
GT_A_84_P800165	EG440981	-0.61	0.0057
GT_A_84_P266910	AT1G47655	-0.85	0.0058
GT_A_84_P10697	AT2G37360	-1.06	0.0059
GT_A_84_P17637	AT4G28140	-1.75	0.0060
GT_A_84_P12049	AT5G06720	-1.08	0.0060
GT_A_84_P837201	ASL9	-2.06	0.0060
GT_A_84_P82289	AT5G62910	-0.66	0.0061
GT_A_84_P298024	EMB1687	-0.68	0.0061
GT_A_84_P297784	AT5G64850	-1.04	0.0061
GT_A_84_P193844	AT5G42965	-0.61	0.0064
GT_A_84_P759252	AT3G62010	-0.73	0.0064
GT_A_84_P757769	AT2G24545	-1.21	0.0067
GT_A_84_P23070	HMT3	-1.93	0.0067
GT_A_84_P799565	AT1G21100	-0.78	0.0068
GT_At_Specific_00087781	AT2G07721.1	-0.95	0.0068
GT_AntiSense_AtMg01100_1048	orf105a	-0.81	0.0069
GT_A_84_P123932	AT1G79510	-0.63	0.0069
GT_A_84_P12726	DREB2B	-1.44	0.0070
GT_AntiSense_AtMg01180_1076	orf111b	-0.84	0.0070
GT_At_Specific_00087998	AT2G07749.1	-0.96	0.0070
GT_A_84_P12747	AT3G46700	-1.51	0.0071
GT_A_84_P530732	RAP2.9	-1.59	0.0071
GT_A_84_P224429	UBQ14	-0.87	0.0072

GT_A_84_P844115	**AT2G07827**	**-0.75**	**0.0074**
GT_At_Specific_00232494	**ATGPX7**	**-3.37**	**0.0074**
GT_A_84_P750593	**AT1G56470**	**-0.66**	**0.0074**
GT_A_84_P22614	**AT5G63320**	**-1.06**	**0.0075**
GT_AtMg01040_1019	**orf107f**	**-0.60**	**0.0075**
GT_A_84_P21397	**AT4G23040**	**-0.83**	**0.0076**
GT_A_84_P803085	**AT2G07827**	**-0.80**	**0.0076**
GT_A_84_P12418	**GGPS6**	**-0.59**	**0.0077**
GT_A_84_P604144	**AT1G72500**	**-0.57**	**0.0077**
GT_A_84_P11342	**LEA14**	**-0.70**	**0.0078**
GT_A_84_P123322	**MSL3**	**-0.85**	**0.0079**
GT_A_84_P19603	**AT1G62740**	**-0.73**	**0.0079**
GT_A_84_P844167	**RAP2.1**	**-1.56**	**0.0080**
GT_A_84_P21910	**AtPP2-A12**	**-0.89**	**0.0080**
GT_A_84_P548684	**AT5G41830**	**-1.67**	**0.0080**
GT_A_84_P815188	**ATGPX1**	**-0.89**	**0.0080**
GT_A_84_P598951	**AT2G40520**	**-1.06**	**0.0081**
GT_A_84_P727923	**TA28535_3702**	**-0.89**	**0.0082**
GT_A_84_P10274	**AT1G64355**	**-0.50**	**0.0082**
GT_A_84_P268440	**AT2G15270**	**-0.56**	**0.0083**
GT_A_84_P786948	**AT2G01300**	**-0.98**	**0.0084**
GT_A_84_P16271	**ATDR4**	**-1.21**	**0.0084**
GT_At_Specific_00118743	**AT2G37360.1**	**-1.25**	**0.0087**
GT_A_84_P750801	**AT1G42490**	**-0.79**	**0.0088**
GT_A_84_P17013	**AT1G19140**	**-0.81**	**0.0088**
GT_At_Specific_00084627	**AT2G04395.1**	**-3.68**	**0.0091**
GT_A_84_P836732	**MSL5**	**-1.25**	**0.0091**
GT_A_84_P18818	**AT5G60530**	**-1.30**	**0.0092**
GT_A_84_P754529	**AT1G43171**	**-0.76**	**0.0092**
GT_A_84_P65364	**AT5G04250**	**-0.74**	**0.0094**
GT_A_84_P13814	**AT4G18810**	**-1.01**	**0.0095**
GT_A_84_P15043	**AT5G62340**	**-4.41**	**0.0095**
GT_A_84_P834162	**ATGSTF4**	**-0.58**	**0.0644**
GT_A_84_P284310	**MLP328**	**-2.06**	**0.0096**
GT_A_84_P856001	**AT5G17460**	**-1.55**	**0.0098**
GT_A_84_P251245	**AT5G48655**	**-0.52**	**0.0100**
GT_A_84_P215638	**LEJ1**	**-0.53**	**0.0101**
GT_At_Specific_00290333	**AT5G48110.1**	**-1.97**	**0.0101**
GT_A_84_P70284	**AT2G43920**	**-0.79**	**0.0103**
GT_A_84_P837399	**EDA10**	**-0.50**	**0.0104**
GT_A_84_P722801	**AT2G07825**	**-0.65**	**0.0104**
GT_A_84_P752730	**MIR163**	**-0.78**	**0.0105**
GT_A_84_P23846	**AtGolS1**	**-3.01**	**0.0105**
GT_A_84_P23302	**AlaAT1**	**-0.54**	**0.0109**
GT_A_84_P791104	**SRS7**	**-0.56**	**0.0110**
GT_A_84_P21028	**ACHT3**	**-1.31**	**0.0110**
GT_AntiSense_AtMg00260_662	**orf101a**	**-0.81**	**0.0111**
GT_A_84_P19363	**ATHSP17.4**	**-4.58**	**0.0111**

GT_A_84_P15341	**VHA-E3**	**-1.06**	**0.0111**
GT_A_84_P856876	**ATDR4**	**-1.14**	**0.0112**
GT_A_84_P230259	**AT3G60980**	**-0.65**	**0.0113**
GT_AntiSense_AtMg00680_870	**orf122c**	**-1.27**	**0.0114**
GT_A_84_P169283	**AT5G06570**	**-2.00**	**0.0114**
GT_A_84_P751028	**NP228609**	**-0.95**	**0.0114**
GT_A_84_P18255	**AT1G75360**	**-0.61**	**0.0114**
GT_A_84_P21640	**ATSERAT1;1**	**-0.52**	**0.0115**
GT_A_84_P869280	**CPuORF49**	**-0.68**	**0.0116**
GT_A_84_P826776	**AT4G12400**	**-4.14**	**0.0116**
GT_A_84_P529136	**AT5G22970**	**-2.83**	**0.0116**
GT_A_84_P19611	**AT5G05900**	**-0.87**	**0.0117**
GT_A_84_P856356	**ATPHB3**	**-0.81**	**0.0117**
GT_A_84_P138679	**AT1G75180**	**-0.95**	**0.0118**
GT_A_84_P23899	**AT2G29500**	**-3.95**	**0.0118**
GT_A_84_P10760	**AT3G05820**	**-0.90**	**0.0118**
GT_A_84_P292314	**AT1G06690**	**-0.64**	**0.0119**
GT_A_84_P22953	**CLPB4**	**-1.57**	**0.0119**
GT_A_84_P23535	**CIPK21**	**-1.19**	**0.0120**
GT_A_84_P11865	**AT3G61580**	**-0.95**	**0.0121**
GT_A_84_P11833	**AT3G53830**	**-2.04**	**0.0122**
GT_A_84_P857058	**TC397723**	**-1.40**	**0.0123**
GT_A_84_P515118	**AT5G56200**	**-1.09**	**0.0124**
GT_A_84_P11914	**AT4G12400**	**-4.02**	**0.0124**
GT_A_84_P12590	**ATPDX1.1**	**-1.34**	**0.0124**
GT_A_84_P515793	**AT5G03890**	**-1.18**	**0.0125**
GT_A_84_P810869	**AT3G14420**	**-0.90**	**0.0127**
GT_A_84_P14235	**BGLU46**	**-1.97**	**0.0129**
GT_A_84_P16609	**AT4G01870**	**-1.02**	**0.0129**
GT_A_84_P19116	**WRKY23**	**-0.80**	**0.0129**
GT_A_84_P58590	**AT3G52740**	**-1.04**	**0.0131**
GT_A_84_P804286	**EG487081**	**-1.42**	**0.0134**
GT_A_84_P16821	**APRR5**	**-2.36**	**0.0134**
GT_A_84_P14863	**CPuORF49**	**-0.75**	**0.0134**
GT_At_Specific_00022274	**AT1G20015.1**	**-0.84**	**0.0134**
GT_A_84_P505592	**AT2G04795**	**-0.72**	**0.0135**
GT_A_84_P755951	**TC391294**	**-1.19**	**0.0137**
GT_A_84_P770341	**NP1098887**	**-0.61**	**0.0138**
GT_A_84_P844951	**TC371970**	**-1.11**	**0.0139**
GT_A_84_P858159	**AT1G20730**	**-2.96**	**0.0141**
GT_A_84_P293484	**AT3G19800**	**-0.68**	**0.0141**
GT_A_84_P800386	**AT2G07806**	**-0.69**	**0.0142**
GT_A_84_P752539	**AT1G08920**	**-0.72**	**0.0142**
GT_A_84_P20918	**AT1G73780**	**-2.21**	**0.0142**
GT_A_84_P534067	**AT2G38640**	**-1.30**	**0.0142**
GT_A_84_P861877	**AT5G64850**	**-1.35**	**0.0143**
GT_A_84_P812371	**RSR4**	**-1.13**	**0.0147**

GT_A_84_P572262	**AT3G10815**	**-1.05**	**0.0148**
GT_A_84_P18469	**AT3G57880**	**-1.07**	**0.0149**
GT_A_84_P17828	**AT5G48570**	**-4.33**	**0.0151**
GT_A_84_P816521	**TA32369_3702**	**-0.63**	**0.0151**
GT_A_84_P20128	**ST**	**-0.90**	**0.0151**
GT_A_84_P267260	**WDL1**	**-0.70**	**0.0151**
GT_A_84_P809081	**RANBP1**	**-0.94**	**0.0153**
GT_A_84_P807528	**GAMMA-TIP**	**-1.01**	**0.0153**
GT_AntiSense_AtMg01210_1100	**orf101b**	**-0.81**	**0.0155**
GT_A_84_P568966	**AT5G04790**	**-0.56**	**0.0158**
GT_A_84_P851955	**TC388770**	**-1.10**	**0.0159**
GT_A_84_P22572	**ATHSP90.1**	**-3.52**	**0.0159**
GT_A_84_P816014	**AT4G01000**	**-0.73**	**0.0161**
GT_A_84_P810829	**LHCA1**	**-0.89**	**0.0162**
GT_A_84_P861734	**LHCA1**	**-0.79**	**0.0163**
GT_A_84_P20788	**ATCEL3**	**-0.80**	**0.0165**
GT_A_84_P567667	**AT2G20080**	**-1.55**	**0.0165**
GT_A_84_P10918	**AT3G61260**	**-0.61**	**0.0165**
GT_A_84_P98486	**AT2G23120**	**-0.64**	**0.0167**
GT_A_84_P15874	**SQP1**	**-1.40**	**0.0167**
GT_A_84_P844289	**TC359193**	**-1.49**	**0.0169**
GT_A_84_P19172	**CYP705A13**	**-1.17**	**0.0169**
GT_A_84_P159915	**SLAH2**	**-1.87**	**0.0169**
GT_A_84_P14413	**ADF5**	**-0.89**	**0.0170**
GT_AntiSense_AtMg00630_839	**orf110b**	**-0.85**	**0.0171**
GT_A_84_P292994	**AT4G12980**	**-1.33**	**0.0171**
GT_AtMg00850_918	**orf107e**	**-1.08**	**0.0172**
GT_A_84_P295894	**AT2G20940**	**-0.76**	**0.0172**
GT_A_84_P815065	**TA31344_3702**	**-1.42**	**0.0172**
GT_At_Specific_00120210	**AT2G38640.1**	**-1.34**	**0.0172**
GT_AtMg00450_729	**orf106b**	**-0.78**	**0.0173**
GT_A_84_P789714	**AT2G32240**	**-1.46**	**0.0174**
GT_At_Specific_00219647	**AT4G21105.2**	**-0.59**	**0.0174**
GT_At_Specific_00212911	**UGT84A3**	**-0.69**	**0.0177**
GT_A_84_P167863	**AT3G14420**	**-0.85**	**0.0178**
GT_A_84_P589734	**AT1G13610**	**-2.09**	**0.0178**
GT_A_84_P829687	**AT4G28150**	**-0.99**	**0.0179**
GT_A_84_P18289	**DAD1**	**-2.01**	**0.0179**
GT_At_Specific_00217818	**MIR168A**	**-0.66**	**0.0180**
GT_A_84_P140999	**AT1G19400**	**-0.89**	**0.0180**
GT_A_84_P594570	**AT3G15240**	**-1.04**	**0.0181**
GT_A_84_P825300	**TA41052_3702**	**-1.04**	**0.0181**
GT_At_Specific_00254373	**HSP17.6II**	**-4.76**	**0.0181**
GT_A_84_P815323	**TA31513_3702**	**-1.56**	**0.0182**
GT_A_84_P558846	**AT2G35130**	**-0.98**	**0.0184**
GT_AntiSense_AtMg00400_703	**orf157**	**-0.88**	**0.0184**
GT_AntiSense_AtMg00310_688	**orf154**	**-0.72**	**0.0185**

GT_A_84_P20240	**AT3G21660**	**-1.74**	**0.0186**
GT_A_84_P21270	**ATARLA1B**	**-1.56**	**0.0186**
GT_A_84_P552884	**AT4G09150**	**-1.18**	**0.0187**
GT_A_84_P246255	**AT1G32220**	**-1.13**	**0.0188**
GT_A_84_P17021	**FTSH1**	**-0.72**	**0.0189**
GT_A_84_P603542	**AT3G48770**	**-0.82**	**0.0189**
GT_A_84_P756443	**TC396102**	**-1.11**	**0.0191**
GT_A_84_P844492		**-0.69**	**0.0193**
GT_A_84_P850443	**TC370874**	**-1.15**	**0.0194**
GT_A_84_P17757	**BMY3**	**-1.19**	**0.0195**
GT_A_84_P609769	**AT3G54730**	**-0.79**	**0.0195**
GT_At_Specific_00314220	**TRNA.1**	**-0.60**	**0.0195**
GT_A_84_P23950	**AT1G07450**	**-2.39**	**0.0196**
GT_A_84_P310243	**AT2G41160**	**-0.50**	**0.0196**
GT_A_84_P16704	**AT1G43860**	**-0.86**	**0.0197**
GT_A_84_P14320	**ATHSP101**	**-3.95**	**0.0199**
GT_A_84_P11772	**AT3G27970**	**-1.63**	**0.0199**
GT_A_84_P769320	**AT5G07322**	**-0.75**	**0.0199**
GT_A_84_P106136	**AT3G12130**	**-0.61**	**0.0199**
GT_A_84_P506289	**ASL9**	**-1.97**	**0.0199**
GT_A_84_P827161	**DRIP1**	**-1.04**	**0.0199**
GT_A_84_P189894	**AT5G22660**	**-2.53**	**0.0201**
GT_A_84_P799584	**AT1G62975**	**-1.01**	**0.0201**
GT_A_84_P807519	**TA26986_3702**	**-0.76**	**0.0202**
GT_A_84_P23356	**ATGSTU14**	**-2.16**	**0.0202**
GT_A_84_P20902	**CDSP32**	**-0.75**	**0.0202**
GT_A_84_P15151	**AT1G24130**	**-0.74**	**0.0203**
GT_A_84_P10874	**LTI30**	**-1.39**	**0.0203**
GT_A_84_P12053	**GDH2**	**-0.80**	**0.0204**
GT_A_84_P770436	**NP1098888**	**-0.86**	**0.0205**
GT_A_84_P222529	**AT1G18490**	**-1.17**	**0.0206**
GT_A_84_P799760	**BX838074**	**-1.14**	**0.0206**
GT_At_Specific_00182353	**AT3G52930.1**	**-0.57**	**0.0207**
GT_A_84_P22292	**FIB**	**-0.75**	**0.0208**
GT_At_Specific_00148282	**MSL5**	**-0.62**	**0.0208**
GT_A_84_P75444	**AT2G24100**	**-0.79**	**0.0208**
GT_AntiSense_AtMg00840_913	**orf121b**	**-1.37**	**0.0208**
GT_A_84_P836671	**TA52555_3702**	**-1.51**	**0.0208**
GT_A_84_P850040	**AGO1**	**-0.69**	**0.0208**
GT_A_84_P19301	**AT3G21770**	**-0.61**	**0.0210**
GT_A_84_P12018	**AT4G15420**	**-0.83**	**0.0211**
GT_A_84_P854259	**CR88**	**-1.08**	**0.0211**
GT_A_84_P822968	**KNAT7**	**-0.76**	**0.0212**
GT_A_84_P803761	**AT2G07684**	**-1.04**	**0.0213**
GT_A_84_P810875	**AT3G14420**	**-0.94**	**0.0214**
GT_At_Specific_00087484	**AT2G07684.1**	**-0.84**	**0.0215**
GT_A_84_P828691	**TA45275_3702**	**-0.69**	**0.0215**

GT_A_84_P770393	**TC402042**	-0.56	**0.0217**
GT_A_84_P789549	**AT4G31110**	-1.34	**0.0217**
GT_A_84_P12164	**AT5G51030**	-1.14	**0.0217**
GT_At_Specific_00188752	**AT3G58540.1**	-0.74	**0.0220**
GT_A_84_P15440	**AT1G07400**	-4.50	**0.0221**
GT_A_84_P12305	**AT1G52050**	-1.00	**0.0221**
GT_A_84_P91299	**AT4G33980**	-2.47	**0.0222**
GT_A_84_P214638	**PGR5-LIKE_A**	-1.40	**0.0224**
GT_A_84_P579842		-0.65	**0.0225**
GT_A_84_P17413	**ROF1**	-1.50	**0.0225**
GT_A_84_P811003	**TA28805_3702**	-0.72	**0.0226**
GT_A_84_P23843	**AT2G03200**	-1.00	**0.0226**
GT_A_84_P858027	**ST**	-1.15	**0.0228**
GT_A_84_P786987	**RAP2.1**	-1.02	**0.0228**
GT_A_84_P280930	**AT1G21670**	-1.03	**0.0229**
GT_At_Specific_00007529	**AT1G07450.1**	-2.12	**0.0229**
GT_A_84_P531386	**AT2G34910**	-2.19	**0.0229**
GT_A_84_P12947	**AtbZIP7**	-0.86	**0.0230**
GT_A_84_P826237	**AT5G64680**	-0.65	**0.0230**
GT_A_84_P753030	**RTFL15**	-1.04	**0.0231**
GT_A_84_P125311	**NADK3**	-0.64	**0.0232**
GT_A_84_P14457	**TC399888**	-3.83	**0.0232**
GT_A_84_P839984	**AT1G63580**	-2.13	**0.0232**
GT_A_84_P810368	**HSP91**	-0.64	**0.0233**
GT_A_84_P10273	**NBP35**	-0.79	**0.0233**
GT_A_84_P15284	**MLO9**	-3.93	**0.0233**
GT_A_84_P801252	**AT1G03220**	-0.89	**0.0233**
GT_A_84_P23407	**PMDH2**	-1.03	**0.0234**
GT_A_84_P20831	**AT1G61340**	-1.87	**0.0234**
GT_A_84_P22530	**NRPB12**	-0.56	**0.0235**
GT_A_84_P757457	**AT2G04800**	-1.64	**0.0236**
GT_A_84_P14417	**AT2G20560**	-2.86	**0.0236**
GT_At_Specific_00315147	**RRN5**	-0.94	**0.0237**
GT_At_Specific_00238494	**HSF4**	-0.74	**0.0237**
GT_A_84_P759774	**AT3G09915**	-1.03	**0.0238**
GT_A_84_P23020	**AtbZIP6**	-0.98	**0.0240**
GT_A_84_P241663	**AT3G58540**	-1.08	**0.0241**
GT_A_84_P22865	**NP306485**	-4.05	**0.0241**
GT_A_84_P17436	**ATBZIP4**	-3.87	**0.0242**
GT_A_84_P19306	**GAPA**	-0.90	**0.0243**
GT_A_84_P15450	**HSP70T-2**	-2.90	**0.0243**
GT_A_84_P18054	**AT1G70810**	-0.99	**0.0244**
GT_A_84_P22388	**AT4G33070**	-1.69	**0.0245**
GT_A_84_P708561	**AT2G22821**	-0.99	**0.0247**
GT_A_84_P263710	**AT3G27960**	-0.64	**0.0249**
GT_A_84_P808747	**GAPA**	-0.68	**0.0249**
GT_A_84_P259030	**AT4G36530**	-1.05	**0.0250**

GT_A_84_P104016	AT1G05805	-0.88	0.0250
GT_A_84_P15663	SAM-2	-0.43	0.0250
GT_A_84_P15783	SQE3	-0.95	0.0251
GT_A_84_P558681	AT1G53903	-1.48	0.0253
GT_A_84_P786570	ATPSK2	-1.61	0.0253
GT_A_84_P859437	AV813724	-1.00	0.0254
GT_A_84_P11751	AtIDD11	-1.15	0.0254
GT_A_84_P756338	AT2G07692	-1.09	0.0254
GT_At_Specific_00063584	AT1G66080.1	-2.24	0.0254
GT_A_84_P10761	AT3G09560	-0.60	0.0255
GT_A_84_P20738	AT5G67190	-2.20	0.0255
GT_A_84_P845241		-1.34	0.0256
GT_A_84_P17668	AT4G35250	-1.12	0.0257
GT_A_84_P196304	AT1G29350	-0.83	0.0258
GT_A_84_P812373	RSR4	-1.05	0.0258
GT_At_Specific_00122922	AT2G40850.1	-1.58	0.0260
GT_A_84_P795693	AK222207	-1.27	0.0260
GT_A_84_P594753	AT4G22230	-1.69	0.0261
GT_At_Specific_00311309	MIR170	-2.07	0.0261
GT_A_84_P13580	AT3G03400	-5.34	0.0263
GT_A_84_P10136	AT4G36670	-1.25	0.0263
GT_A_84_P13039	AT5G24420	-2.99	0.0264
GT_A_84_P804390	rpl5	-0.62	0.0267
GT_A_84_P140919	AT2G16365	-1.41	0.0267
GT_A_84_P534040	AT2G31810	-0.72	0.0269
GT_A_84_P837979	ASL9	-2.23	0.0269
GT_A_84_P10998	SGT1A	-1.97	0.0269
GT_A_84_P805904	EXL4	-1.13	0.0273
GT_A_84_P174791	TRB1	-0.87	0.0273
GT_A_84_P825094	TA40791_3702	-1.30	0.0273
GT_AntiSense_AtMg01220_1103	orf113	-0.79	0.0273
GT_AntiSense_AtMg00890_938	orf106d	-0.93	0.0274
GT_A_84_P769159	AT5G15853	-1.55	0.0274
GT_A_84_P708257	AT4G35080	-1.38	0.0276
GT_A_84_P785245	AT4G38550	-1.06	0.0277
GT_A_84_P785012	AtGolS1	-3.25	0.0278
GT_A_84_P590035	AT2G43535	-0.88	0.0278
GT_A_84_P715503		-0.51	0.0278
GT_A_84_P812366	RSR4	-1.10	0.0278
GT_A_84_P813172	AT1G75180	-0.90	0.0279
GT_A_84_P184384	AT1G68440	-0.97	0.0280
GT_A_84_P180414	AT5G55970	-1.52	0.0280
GT_A_84_P21617	CPHSC70-2EAT	-1.37	0.0281
GT_A_84_P770350	TC402456	-1.06	0.0283
GT_A_84_P204138	AT1G66080	-2.22	0.0284
GT_A_84_P578332	MRH6	-1.73	0.0284
GT_A_84_P808064	PSBO2	-0.95	0.0285

GT_A_84_P795254	AT5G65220	-0.60	0.0285
GT_A_84_P14071	NAS2	-3.84	0.0286
GT_A_84_P613238	MSL5	-0.74	0.0286
GT_A_84_P241325	AT1G52870	-0.83	0.0288
GT_A_84_P763707	AT4G06521	-0.82	0.0288
GT_A_84_P22274	AT-HSFA7B	-3.92	0.0289
GT_A_84_P11289	AT5G16010	-0.94	0.0289
GT_A_84_P158205	AT1G22470	-1.13	0.0289
GT_A_84_P831803	AT2G40520	-1.07	0.0289
GT_A_84_P164433	AT4G02920	-1.19	0.0290
GT_A_84_P17404	RNR2A	-0.73	0.0290
GT_A_84_P812767	TA29778_3702	-0.91	0.0291
GT_A_84_P22203	TC370048	-0.83	0.0291
GT_A_84_P767195	AT5G41710	-0.82	0.0294
GT_A_84_P13560	AT2G32150	-0.98	0.0295
GT_A_84_P812233	TA29435_3702	-0.85	0.0295
GT_A_84_P21049	PLA2A	-1.35	0.0296
GT_A_84_P13435	SUS6	-0.82	0.0297
GT_A_84_P835772	TA52178_3702	-1.65	0.0297
GT_At_Specific_00137950	AT3G06019.1	-1.28	0.0298
GT_A_84_P519526	AT3G22100	-1.19	0.0299
GT_A_84_P848730	TC396102	-0.67	0.0300
GT_A_84_P97076	AT1G80380	-0.87	0.0300
GT_A_84_P18737	AT5G38710	-2.20	0.0300
GT_A_84_P13741	AT3G57600	-1.03	0.0301
GT_A_84_P143109	AT1G79920	-1.02	0.0301
GT_A_84_P22619	AT5G64460	-0.54	0.0301
GT_A_84_P21922	CYP87A2	-2.16	0.0302
GT_A_84_P17319	AT1G67670	-1.51	0.0302
GT_A_84_P827680	TA43931_3702	-0.93	0.0303
GT_A_84_P20085	AT2G35270	-2.60	0.0305
GT_A_84_P13135	PLC1	-1.25	0.0305
GT_A_84_P827473	AT4G33980	-2.67	0.0307
GT_A_84_P788285	AT3G19230	-1.37	0.0310
GT_A_84_P18342	A37	-1.73	0.0310
GT_At_Specific_00314230	TRNI.2	-0.57	0.0311
GT_A_84_P507007	AT5G41590	-3.45	0.0312
GT_AtMg00010_1220	orf153a	-0.86	0.0313
GT_A_84_P13988	AT5G25450	-1.82	0.0316
GT_A_84_P20921	AT1G77520	-2.38	0.0318
GT_A_84_P521210	AT5G44005	-0.91	0.0318
GT_AntiSense_AtMg00610_837	orf161	-0.89	0.0319
GT_A_84_P860053	TA31325_3702	-2.38	0.0320
GT_A_84_P15799	UGT84A3	-0.60	0.0320
GT_A_84_P569083	AT5G46440	-0.94	0.0320
GT_At_Specific_00065953	ADO3	-1.96	0.0320
GT_AtMg01230_1110	orf145b	-1.08	0.0321

GT_A_84_P806316	**AtATG18h**	**-3.34**	**0.0322**
GT_A_84_P19979	**AT1G66580**	**-0.64**	**0.0324**
GT_A_84_P67084	**AT1G25400**	**-1.55**	**0.0324**
GT_A_84_P23814	**AT1G20030**	**-1.42**	**0.0324**
GT_A_84_P16133	**AtMC7**	**-1.28**	**0.0325**
GT_A_84_P19290	**FAD5**	**-0.97**	**0.0325**
GT_A_84_P832753	**AT4G18740**	**-1.05**	**0.0326**
GT_A_84_P832698	**AT5G56000**	**-1.18**	**0.0328**
GT_A_84_P756371	**AT2G07672**	**-0.84**	**0.0329**
GT_A_84_P504952	**AT3G50300**	**-1.24**	**0.0329**
GT_A_84_P11493	**FKF1**	**-0.89**	**0.0331**
GT_At_Specific_00226768	**AT4G27030.1**	**-1.79**	**0.0333**
GT_A_84_P850157	**TA48759_3702**	**-0.98**	**0.0333**
GT_A_84_P609850	**AT4G16550**	**-1.77**	**0.0333**
GT_At_Specific_00221269	**AT4G22380.1**	**-0.56**	**0.0338**
GT_A_84_P17513	**AT3G55580**	**-3.70**	**0.0340**
GT_A_84_P10555	**AT1G53540**	**-4.93**	**0.0340**
GT_A_84_P545700	**AT3G23190**	**-0.62**	**0.0340**
GT_A_84_P21208	**AT3G29670**	**-1.34**	**0.0341**
GT_A_84_P755464	**AT2G16190**	**-2.04**	**0.0342**
GT_A_84_P10321	**AT5G63380**	**-0.78**	**0.0342**
GT_A_84_P807650	**TA27069_3702**	**-0.92**	**0.0344**
GT_A_84_P605975	**AT2G04800**	**-1.51**	**0.0344**
GT_AntiSense_AtMg01400_1186	**orf105b**	**-1.11**	**0.0345**
GT_A_84_P500185	**AT1G63590**	**-1.27**	**0.0345**
GT_A_84_P756909	**AT2G07741**	**-0.61**	**0.0345**
GT_At_Specific_00314741	**ORF120**	**-1.43**	**0.0347**
GT_A_84_P20080	**PIP2B**	**-0.71**	**0.0348**
GT_A_84_P511862	**AT2G32550**	**-3.31**	**0.0351**
GT_A_84_P798445	**AT5G64640**	**-0.65**	**0.0351**
GT_At_Specific_00024262	**AT1G21722.1**	**-2.27**	**0.0352**
GT_A_84_P10354	**PGP13**	**-1.22**	**0.0352**
GT_A_84_P763462	**RMA1**	**-0.99**	**0.0353**
GT_A_84_P204118	**AT5G50450**	**-1.92**	**0.0355**
GT_A_84_P20285	**AT3G13660**	**-2.41**	**0.0356**
GT_A_84_P20709	**AT5G60310**	**-1.25**	**0.0356**
GT_A_84_P815195	**ATGPX1**	**-0.82**	**0.0356**
GT_A_84_P576340	**AT1G06750**	**-1.50**	**0.0357**
GT_A_84_P164953	**ABF1**	**-1.94**	**0.0361**
GT_A_84_P224479	**AT5G16650**	**-0.91**	**0.0362**
GT_A_84_P228049	**BT1**	**-1.28**	**0.0362**
GT_At_Specific_00005621	**AT1G05805.1**	**-0.83**	**0.0364**
GT_A_84_P856045	**ATGPX1**	**-0.82**	**0.0367**
GT_A_84_P723394	**AT2G46192**	**-0.64**	**0.0368**
GT_A_84_P846056		**-0.64**	**0.0368**
GT_A_84_P809155	**TA27913_3702**	**-1.09**	**0.0369**
GT_A_84_P819099	**TA34521_3702**	**-2.61**	**0.0370**

GT_A_84_P189144	AT2G35170	-0.64	0.0370
GT_A_84_P821420	TA36674_3702	-1.05	0.0373
GT_A_84_P770045	ATNADH	-0.50	0.0373
GT_A_84_P219878	AT3G16450	-0.76	0.0375
GT_A_84_P20426	AT4G12790	-0.61	0.0376
GT_A_84_P853774	AT5G24430	-0.57	0.0378
GT_A_84_P18930	AT1G60750	-1.24	0.0378
GT_At_Specific_00088253	AT2G07782.1	-0.60	0.0379
GT_A_84_P816178	CDSP32	-0.56	0.0379
GT_A_84_P756324	AT2G07694	-0.92	0.0380
GT_A_84_P93089	AT1G13650	-1.63	0.0380
GT_A_84_P816095	AT4G01026	-1.01	0.0381
GT_At_Specific_00214329	AT4G16550.1	-1.10	0.0381
GT_A_84_P199724	TZP	-1.31	0.0382
GT_A_84_P15225	AT1G08170	-2.02	0.0382
GT_A_84_P15993	CYP94B1	-1.85	0.0383
GT_A_84_P76494	AT3G03990	-0.70	0.0383
GT_A_84_P182154	ATFRO6	-1.24	0.0383
GT_A_84_P833332	AT5G20860	-1.90	0.0385
GT_A_84_P12141	MYB20	-0.99	0.0386
GT_A_84_P23591	AT5G20550	-0.83	0.0386
GT_A_84_P22903	AT1G63450	-1.54	0.0386
GT_A_84_P20212	AT3G04710	-1.33	0.0387
GT_A_84_P815621	ATBETAFRUCT4	-0.85	0.0387
GT_A_84_P13472	AT2G45430	-1.11	0.0387
GT_A_84_P503102	AT3G15440	-3.45	0.0387
GT_A_84_P593014	AT4G31110	-1.35	0.0388
GT_A_84_P567338	AT5G55570	-3.22	0.0388
GT_A_84_P18011	AT1G75690	-1.25	0.0389
GT_A_84_P17937	CINV1	-0.69	0.0391
GT_A_84_P15912	AT5G42180	-2.27	0.0391
GT_A_84_P17113	AT1G22490	-1.71	0.0391
GT_A_84_P94199	AT2G29670	-1.59	0.0393
GT_A_84_P10466	GLP7	-1.04	0.0393
GT_A_84_P806805	AT3G09440	-2.02	0.0394
GT_A_84_P824293	TA39766_3702	-1.33	0.0394
GT_A_84_P824560	TA40086_3702	-0.67	0.0395
GT_A_84_P23707	APS2	-1.45	0.0396
GT_A_84_P785959	AT4G05070	-1.46	0.0397
GT_A_84_P598557	VIN3	-3.24	0.0399
GT_A_84_P769994	TC380043	-0.64	0.0400
GT_A_84_P10009	AT4G08870	-2.16	0.0400
GT_A_84_P18650	RSR4	-1.15	0.0401
GT_A_84_P252515	NTRC	-1.07	0.0404
GT_A_84_P784525	AT4G17098	-1.56	0.0404
GT_A_84_P765827	AT4G37409	-2.42	0.0404
GT_A_84_P17009	AT1G20823	-1.09	0.0404

GT_A_84_P23245	**AT1G06020**	**-0.89**	**0.0405**
GT_A_84_P141649	**AT3G55760**	**-1.17**	**0.0405**
GT_A_84_P819821	**AT2G20560**	**-2.69**	**0.0406**
GT_A_84_P846980		**-2.04**	**0.0406**
GT_A_84_P159165	**AT1G17030**	**-1.49**	**0.0407**
GT_A_84_P10863	**CYP94B3**	**-1.81**	**0.0411**
GT_A_84_P793960	**AT1G08920**	**-1.00**	**0.0412**
GT_A_84_P133525	**ATPSK4**	**-0.75**	**0.0412**
GT_A_84_P21159	**AT3G04010**	**-2.38**	**0.0413**
GT_A_84_P810125	**LTI78**	**-0.66**	**0.0414**
GT_A_84_P842524	**NP237995**	**-0.59**	**0.0414**
GT_A_84_P845111	**AT4G02920**	**-0.96**	**0.0415**
GT_A_84_P269630	**AT2G27505**	**-2.57**	**0.0415**
GT_A_84_P12603	**CTF2A**	**-1.34**	**0.0417**
GT_A_84_P822458	**AT1G70810**	**-0.82**	**0.0417**
GT_A_84_P11068	**AT4G14060**	**-1.39**	**0.0417**
GT_A_84_P10886	**AtPPa4**	**-1.27**	**0.0419**
GT_A_84_P17111	**AT1G02820**	**-3.05**	**0.0420**
GT_A_84_P21186	**AT3G21670**	**-1.16**	**0.0420**
GT_A_84_P861478	**BP640374**	**-0.69**	**0.0421**
GT_A_84_P22781	**AT1G49390**	**-1.09**	**0.0422**
GT_A_84_P20157	**AT2G18470**	**-1.88**	**0.0423**
GT_A_84_P760294	**NF-YB10**	**-0.72**	**0.0423**
GT_A_84_P849455	**TA52032_3702**	**-1.56**	**0.0423**
GT_A_84_P581530	**AT5G36662**	**-2.54**	**0.0424**
GT_A_84_P12417	**GA2OX7**	**-1.65**	**0.0424**
GT_A_84_P721670	**BU917423**	**-3.03**	**0.0424**
GT_A_84_P859545	**PDX1L4**	**-1.26**	**0.0424**
GT_A_84_P563658	**AT5G11290**	**-1.06**	**0.0424**
GT_A_84_P24124	**PIP2A**	**-1.11**	**0.0425**
GT_A_84_P115172	**RIC2**	**-0.79**	**0.0425**
GT_A_84_P16977	**AT5G28750**	**-0.68**	**0.0427**
GT_A_84_P17935	**AT5G37440**	**-1.45**	**0.0427**
GT_A_84_P20389	**AT4G01026**	**-1.16**	**0.0428**
GT_A_84_P812100	**PGK**	**-0.59**	**0.0428**
GT_A_84_P807230	**DRT112**	**-0.50**	**0.0428**
GT_A_84_P828684	**TA45274_3702**	**-0.59**	**0.0431**
GT_A_84_P306860	**AT5G10695**	**-1.48**	**0.0432**
GT_A_84_P18763	**CIPK20**	**-1.97**	**0.0433**
GT_A_84_P12490	**MYB54**	**-0.68**	**0.0434**
GT_A_84_P549559	**AT5G38100**	**-1.72**	**0.0438**
GT_A_84_P14864	**MYB73**	**-0.74**	**0.0438**
GT_A_84_P10736	**AT3G01070**	**-1.01**	**0.0439**
GT_A_84_P816924	**TA32732_3702**	**-0.73**	**0.0439**
GT_A_84_P581880	**AT1G78120**	**-1.86**	**0.0440**
GT_A_84_P840007	**AT1G18460**	**-0.75**	**0.0440**
GT_A_84_P15740	**AT4G27030**	**-1.36**	**0.0440**

GT_A_84_P829339	**AT3G01510**	**-0.76**	**0.0441**
GT_A_84_P10179	**MYB46**	**-0.70**	**0.0441**
GT_A_84_P852782	**TC379107**	**-1.18**	**0.0441**
GT_A_84_P16202	**AT1G07870**	**-0.50**	**0.0441**
GT_A_84_P577620	**AT3G13435**	**-1.37**	**0.0441**
GT_A_84_P10171	**MES5**	**-0.81**	**0.0442**
GT_A_84_P12702	**ATMLP-300B**	**-1.23**	**0.0443**
GT_A_84_P18618	**ANAC076**	**-0.48**	**0.0443**
GT_A_84_P239005	**APUM18**	**-0.61**	**0.0443**
GT_A_84_P23649	**AT1G31050**	**-1.97**	**0.0444**
GT_A_84_P850750	**AT5G50300**	**-0.87**	**0.0444**
GT_A_84_P247395	**AT3G26470**	**-0.75**	**0.0446**
GT_A_84_P830237	**AT5G60030**	**-0.74**	**0.0447**
GT_A_84_P769933	**TA51185_3702**	**-0.53**	**0.0448**
GT_A_84_P799996	**AT5G53490**	**-0.78**	**0.0448**
GT_AtMg00890_938	**orf106d**	**-0.95**	**0.0448**
GT_A_84_P63864	**PGR5**	**-0.82**	**0.0449**
GT_A_84_P850851	**BP563965**	**-0.94**	**0.0449**
GT_A_84_P300030	**ATAF2**	**-0.87**	**0.0449**
GT_A_84_P855960	**AT2G35130**	**-1.09**	**0.0450**
GT_A_84_P20057	**AT1G63580**	**-2.09**	**0.0452**
GT_A_84_P10519	**AT1G74820**	**-1.96**	**0.0453**
GT_A_84_P16310	**CYP98A3**	**-0.88**	**0.0454**
GT_A_84_P13214	**AT2G07691**	**-0.72**	**0.0454**
GT_A_84_P849870	**AT4G33120**	**-0.58**	**0.0454**
GT_A_84_P18334	**MYB108**	**-1.36**	**0.0455**
GT_A_84_P23103	**ATPPC3**	**-0.75**	**0.0455**
GT_A_84_P10324	**PSAN**	**-0.77**	**0.0456**
GT_A_84_P23712	**AT1G59860**	**-4.35**	**0.0457**
GT_A_84_P803093	**AT2G07772**	**-1.33**	**0.0458**
GT_A_84_P23544	**HSP18.2**	**-2.22**	**0.0459**
GT_A_84_P15059	**ROPGEF11**	**-1.40**	**0.0460**
GT_A_84_P763581	**MEE58**	**-0.87**	**0.0460**
GT_AtMg00610_837	**orf161**	**-0.92**	**0.0461**
GT_A_84_P310283	**AT5G01320**	**-1.21**	**0.0462**
GT_A_84_P11568	**AT2G40460**	**-1.10**	**0.0462**
GT_A_84_P753835	**AT1G19115**	**-1.31**	**0.0462**
GT_A_84_P21451	**CAT2**	**-1.43**	**0.0463**
GT_A_84_P601857	**AT4G17550**	**-1.18**	**0.0463**
GT_A_84_P167943	**ERF4**	**-0.82**	**0.0464**
GT_A_84_P526341	**PGR5-LIKE_A**	**-1.28**	**0.0465**
GT_A_84_P16547	**KNAT7**	**-0.60**	**0.0465**
GT_A_84_P815576	**TA31702_3702**	**-1.78**	**0.0468**
GT_A_84_P179004	**LBD15**	**-0.76**	**0.0471**
GT_A_84_P17923	**BZO2H3**	**-1.34**	**0.0472**
GT_A_84_P18796	**PDC2**	**-1.36**	**0.0472**
GT_A_84_P15802	**AT4G16500**	**-0.65**	**0.0472**

GT_A_84_P815058	**NF-YC2**	**-1.51**	**0.0473**
GT_A_84_P15063	**ATNRAMP4**	**-0.59**	**0.0474**
GT_A_84_P155845	**AT2G47950**	**-1.16**	**0.0475**
GT_A_84_P857052	**AVA-P1**	**-0.48**	**0.0475**
GT_A_84_P525034	**AT1G62975**	**-0.80**	**0.0476**
GT_A_84_P14725	**TC377111**	**-0.99**	**0.0477**
GT_A_84_P787140	**ATFRO6**	**-0.98**	**0.0477**
GT_A_84_P809159	**PIP2A**	**-1.07**	**0.0478**
GT_A_84_P751343	**AY057670**	**-1.57**	**0.0481**
GT_A_84_P19141	**MYB12**	**-0.72**	**0.0481**
GT_A_84_P827762	**ZFP6**	**-1.12**	**0.0482**
GT_A_84_P816484	**TA32350_3702**	**-1.40**	**0.0482**
GT_A_84_P18380	**AT1G12950**	**-1.84**	**0.0483**
GT_A_84_P844006	**AT2G27505**	**-3.36**	**0.0484**
GT_A_84_P16960	**COL1**	**-0.88**	**0.0484**
GT_A_84_P293824	**AT5G39650**	**-1.26**	**0.0485**
GT_A_84_P18172	**AT2G19970**	**-1.06**	**0.0487**
GT_A_84_P842001	**AT5G55570**	**-3.04**	**0.0488**
GT_A_84_P154615	**AT1G76590**	**-1.10**	**0.0490**
GT_A_84_P769724	**TC404086**	**-0.58**	**0.0490**
GT_A_84_P12094	**AT5G24410**	**-1.21**	**0.0491**
GT_A_84_P277930	**AT2G02680**	**-1.92**	**0.0491**
GT_A_84_P805340	**TA25950_3702**	**-0.68**	**0.0492**
GT_A_84_P11307	**EXL4**	**-1.52**	**0.0496**
GT_A_84_P824264	**TA39749_3702**	**-0.76**	**0.0497**
GT_A_84_P786823	**ATMRP8**	**-0.80**	**0.0498**
GT_A_84_P522542	**AT2G39240**	**-0.59**	**0.0498**
GT_A_84_P538729	**AT4G10290**	**-3.55**	**0.0499**
GT_AntiSense_AtMg01410_1194	**orf204**	**-1.15**	**0.0502**
GT_A_84_P537588	**AT2G33705**	**-1.40**	**0.0502**
GT_A_84_P21687	**GAPB**	**-1.04**	**0.0503**
GT_A_84_P238905	**scpl1**	**-1.44**	**0.0504**
GT_A_84_P163153	**AT1G74830**	**-2.33**	**0.0505**
GT_A_84_P212158	**AT1G09690**	**-0.48**	**0.0505**
GT_A_84_P23956	**AT1G07350**	**-1.27**	**0.0506**
GT_A_84_P13535	**AT2G38240**	**-0.97**	**0.0509**
GT_A_84_P13690	**AT3G46370**	**-3.00**	**0.0512**
GT_A_84_P581112	**AT2G42150**	**-2.25**	**0.0512**
GT_A_84_P21867	**AT1G77530**	**-4.47**	**0.0513**
GT_A_84_P763873	**TA47282_3702**	**-2.25**	**0.0516**
GT_At_Specific_00246086	**C2H2**	**-1.15**	**0.0516**
GT_A_84_P853675	**BGLU46**	**-1.75**	**0.0516**
GT_A_84_P841491	**NP227289**	**-1.64**	**0.0516**
GT_A_84_P20579	**HSP17.6II**	**-4.82**	**0.0517**
GT_A_84_P822924	**BZO2H3**	**-1.38**	**0.0517**
GT_A_84_P108742	**RAP2.1**	**-1.35**	**0.0517**
GT_A_84_P10860	**AT3G47580**	**-1.28**	**0.0518**

GT_A_84_P811621	**TA29115_3702**	**-0.97**	**0.0519**
GT_A_84_P803155		**-0.60**	**0.0520**
GT_A_84_P18912	**PSBY**	**-0.78**	**0.0520**
GT_A_84_P847056	**TC359579**	**-0.94**	**0.0521**
GT_A_84_P800885	**ATBETAFRUCT4**	**-1.22**	**0.0524**
GT_A_84_P765100	**AT4G17098**	**-1.52**	**0.0524**
GT_A_84_P826969	**AT1G65030**	**-0.58**	**0.0525**
GT_A_84_P22812	**ARR15**	**-2.88**	**0.0525**
GT_A_84_P577314	**AT1G32950**	**-1.42**	**0.0526**
GT_A_84_P784958	**AT1G74840**	**-1.40**	**0.0528**
GT_A_84_P785932	**AT1G25400**	**-1.52**	**0.0529**
GT_A_84_P10884	**PAL2**	**-2.09**	**0.0530**
GT_A_84_P820040	**PLA2A**	**-1.47**	**0.0530**
GT_A_84_P601919	**AT4G34550**	**-3.81**	**0.0531**
GT_A_84_P789561		**-1.60**	**0.0532**
GT_A_84_P849292	**AT1G74840**	**-1.34**	**0.0533**
GT_A_84_P11893	**AT4G04840**	**-1.83**	**0.0533**
GT_A_84_P180164	**AT5G47830**	**-1.00**	**0.0534**
GT_A_84_P275050	**AT3G27210**	**-0.76**	**0.0537**
GT_A_84_P762751	**AT3G10986**	**-1.13**	**0.0538**
GT_A_84_P838689	**ERD9**	**-0.89**	**0.0539**
GT_A_84_P855355	**ATFRO7**	**-0.90**	**0.0540**
GT_A_84_P16057	**ATTRX5**	**-0.90**	**0.0540**
GT_A_84_P16804	**ATMYB16**	**-0.69**	**0.0541**
GT_A_84_P789400	**AT5G21950**	**-1.38**	**0.0542**
GT_A_84_P234169	**AT2G21840**	**-0.86**	**0.0543**
GT_A_84_P807778	**TA27161_3702**	**-1.18**	**0.0544**
GT_A_84_P770298	**ATMG00890**	**-0.97**	**0.0546**
GT_A_84_P200134	**SFH12**	**-2.23**	**0.0546**
GT_A_84_P233989	**AT2G07747**	**-0.85**	**0.0548**
GT_A_84_P21213	**ATMRP8**	**-1.00**	**0.0549**
GT_A_84_P836184	**TA52348_3702**	**-1.02**	**0.0551**
GT_A_84_P11789	**AT3G43250**	**-1.84**	**0.0554**
GT_A_84_P839463	**AT5G37478**	**-1.61**	**0.0555**
GT_A_84_P602770	**AT4G21215**	**-0.89**	**0.0559**
GT_A_84_P211908	**AT5G05500**	**-1.99**	**0.0561**
GT_A_84_P755497	**AK227826**	**-1.12**	**0.0561**
GT_A_84_P803007	**AV563501**	**-0.59**	**0.0562**
GT_A_84_P77119	**EXL1**	**-1.78**	**0.0567**
GT_A_84_P851624	**AT4G11320**	**-1.33**	**0.0567**
GT_A_84_P764560	**AT4G03505**	**-1.66**	**0.0568**
GT_A_84_P22499	**CYP71B11**	**-0.72**	**0.0570**
GT_A_84_P760818	**AT3G24005**	**-0.62**	**0.0571**
GT_A_84_P862493	**AtPP2-A11**	**-0.57**	**0.0572**
GT_At_Specific_00268310	**AT5G25250.1**	**-0.99**	**0.0572**
GT_A_84_P214908	**AT4G15510**	**-0.83**	**0.0572**
GT_A_84_P18836	**AT5G64700**	**-1.03**	**0.0573**

GT_A_84_P21573	**AT5G38120**	**-5.50**	**0.0574**
GT_A_84_P514608	**AT3G09032**	**-1.11**	**0.0574**
GT_At_Specific_00135026	**SUVR3**	**-1.20**	**0.0575**
GT_A_84_P21273	**OBP1**	**-1.21**	**0.0575**
GT_A_84_P519326	**AT5G06520**	**-1.63**	**0.0575**
GT_A_84_P155935	**AT4G27590**	**-1.79**	**0.0575**
GT_A_84_P18032	**AT1G54050**	**-4.48**	**0.0576**
GT_A_84_P784743	**AT5G62140**	**-1.45**	**0.0576**
GT_A_84_P574551	**LAS**	**-2.01**	**0.0576**
GT_A_84_P806273	**NPQ4**	**-1.68**	**0.0579**
GT_A_84_P523311	**TA26574_3702**	**-1.03**	**0.0579**
GT_A_84_P23880	**AT2G03820**	**-0.62**	**0.0580**
GT_A_84_P545146	**AT5G40180**	**-1.33**	**0.0580**
GT_A_84_P21754	**AT1G23710**	**-0.94**	**0.0581**
GT_A_84_P216248	**AT1G69890**	**-1.16**	**0.0582**
GT_A_84_P560513	**AT1G70640**	**-1.93**	**0.0583**
GT_A_84_P19256	**LAC7**	**-0.98**	**0.0584**
GT_A_84_P519178	**BB**	**-0.87**	**0.0584**
GT_A_84_P87139	**FUT2**	**-2.34**	**0.0587**
GT_A_84_P563378	**AT3G15357**	**-0.81**	**0.0588**
GT_A_84_P271340	**AT3G09350**	**-2.69**	**0.0588**
GT_A_84_P752754	**AT1G64195**	**-2.49**	**0.0590**
GT_A_84_P186914	**BSMT1**	**-3.54**	**0.0590**
GT_A_84_P274380	**AT2G25530**	**-0.82**	**0.0591**
GT_A_84_P16341	**ADS2**	**-1.90**	**0.0591**
GT_A_84_P219118	**AT4G38970**	**-1.05**	**0.0591**
GT_A_84_P571144	**AT1G61860**	**-0.93**	**0.0592**
GT_A_84_P18807	**TIL**	**-1.00**	**0.0592**
GT_A_84_P18401	**HSP70**	**-3.00**	**0.0597**
GT_A_84_P789823	**DR375991**	**-1.82**	**0.0598**
GT_A_84_P10505	**AT1G78290**	**-1.61**	**0.0598**
GT_A_84_P802955	**H76628**	**-0.80**	**0.0599**
GT_A_84_P18196	**ATGSTF8**	**-1.18**	**0.0600**
GT_A_84_P22918	**AT2G28600**	**-0.63**	**0.0600**
GT_A_84_P855789	**ATGSTF8**	**-1.12**	**0.0600**
GT_A_84_P821119	**AT3G09350**	**-2.64**	**0.0602**
GT_A_84_P852521	**AT1G78290**	**-1.69**	**0.0603**
GT_A_84_P821127	**TA36299_3702**	**-1.13**	**0.0606**
GT_A_84_P301340	**AT1G80120**	**-1.57**	**0.0610**
GT_A_84_P570186	**AT1G32860**	**-2.47**	**0.0612**
GT_A_84_P14174	**LHCB6**	**-0.70**	**0.0614**
GT_A_84_P789465	**ABF1**	**-1.72**	**0.0614**
GT_A_84_P821906	**TA37110_3702**	**-1.41**	**0.0616**
GT_A_84_P102136	**AT4G18740**	**-1.18**	**0.0618**
GT_A_84_P805353	**TA25953_3702**	**-0.60**	**0.0619**
GT_A_84_P62840	**ELIP2**	**-2.82**	**0.0623**
GT_A_84_P18059	**AT1G22480**	**-3.25**	**0.0624**

GT_A_84_P17164	**AtbZIP**	**-1.12**	**0.0624**
GT_A_84_P14268	**AT1G47710**	**-1.53**	**0.0624**
GT_A_84_P176164	**AT2G45300**	**-0.86**	**0.0624**
GT_A_84_P845642	**AT4G27590**	**-1.41**	**0.0624**
GT_A_84_P19559	**AT1G15010**	**-1.02**	**0.0626**
GT_A_84_P16754	**ATHB40**	**-2.17**	**0.0629**
GT_A_84_P17498	**AT3G52620**	**-0.48**	**0.0630**
GT_A_84_P754651	**AT1G65484**	**-1.73**	**0.0631**
GT_A_84_P750244	**AT1G69140**	**-3.56**	**0.0632**
GT_A_84_P164883	**AT5G42900**	**-2.73**	**0.0634**
GT_A_84_P725162	**AT1G47271**	**-0.98**	**0.0638**
GT_A_84_P808818	**VSP2**	**-1.89**	**0.0639**
GT_A_84_P817501	**TA33162_3702**	**-1.45**	**0.0639**
GT_A_84_P155475	**AT4G35060**	**-0.95**	**0.0640**
GT_A_84_P23758	**AT1G74840**	**-1.69**	**0.0640**
GT_A_84_P819880	**TA35224_3702**	**-0.71**	**0.0641**
GT_A_84_P20924	**AT1G18460**	**-0.67**	**0.0641**
GT_A_84_P752083	**AT1G67855**	**-1.08**	**0.0642**
GT_A_84_P21595	**AT5G44360**	**-1.76**	**0.0643**
GT_A_84_P524680	**JAZ10**	**-1.96**	**0.0643**
GT_A_84_P12466	**HEMA1**	**-1.21**	**0.0643**
GT_A_84_P575402	**AT1G03055**	**-2.14**	**0.0645**
GT_A_84_P21525	**AT-HSP17.6A**	**-4.72**	**0.0648**
GT_A_84_P92989	**AT4G32110**	**-1.05**	**0.0649**
GT_A_84_P861424	**BP640902**	**-0.77**	**0.0654**
GT_A_84_P20887	**SPS2**	**-1.28**	**0.0655**
GT_A_84_P805621	**VSP1**	**-1.93**	**0.0656**
GT_A_84_P853245	**PIP2A**	**-0.82**	**0.0656**
GT_A_84_P599932	**AT3G18670**	**-0.44**	**0.0657**
GT_A_84_P169313	**ProT3**	**-0.86**	**0.0658**
GT_A_84_P192974	**AT5G65660**	**-1.09**	**0.0662**
GT_A_84_P828241	**TA44692_3702**	**-1.56**	**0.0662**
GT_A_84_P849757	**BGLU46**	**-1.99**	**0.0663**
GT_A_84_P795046	**AT2G18690**	**-0.82**	**0.0664**
GT_A_84_P507728	**SP1L5**	**-1.47**	**0.0664**
GT_A_84_P74804	**AT1G79660**	**-1.42**	**0.0665**
GT_A_84_P20968	**WOX14**	**-1.09**	**0.0666**
GT_A_84_P123192	**AT5G51720**	**-1.88**	**0.0666**
GT_A_84_P814816	**RPS15AD**	**-0.50**	**0.0667**
GT_A_84_P850860		**-1.80**	**0.0668**
GT_A_84_P770152	**ATMG01350**	**-0.68**	**0.0669**
GT_A_84_P22685	**AT4G11175**	**-0.66**	**0.0670**
GT_A_84_P846878	**AT3G59020**	**-0.64**	**0.0670**
GT_A_84_P279650	**AT1G19490**	**-0.61**	**0.0674**
GT_A_84_P11284	**AT5G67520**	**-0.93**	**0.0676**
GT_A_84_P784937	**AT1G21670**	**-0.78**	**0.0676**
GT_A_84_P828102	**AT5G62140**	**-0.99**	**0.0679**

GT_A_84_P517367	**LAS1**	**-4.52**	**0.0679**
GT_A_84_P16924	**AT5G60030**	**-0.67**	**0.0680**
GT_A_84_P770208	**EF182951**	**-1.21**	**0.0681**
GT_A_84_P768705	**AT5G20225**	**-1.92**	**0.0682**
GT_A_84_P144289	**AT4G29770**	**-0.72**	**0.0682**
GT_A_84_P816061	**TA32027_3702**	**-0.74**	**0.0683**
GT_A_84_P16032	**AT1G28040**	**-1.78**	**0.0684**
GT_A_84_P100746	**AT3G28220**	**-1.31**	**0.0686**
GT_A_84_P562254	**AT1G66060**	**-3.50**	**0.0688**
GT_A_84_P758548	**Z17783**	**-0.69**	**0.0689**
GT_A_84_P830406	**AT3G53230**	**-0.90**	**0.0690**
GT_A_84_P594805	**AT4G36820**	**-2.50**	**0.0691**
GT_A_84_P575158	**AT4G30150**	**-0.58**	**0.0693**
GT_A_84_P568910	**BGLU9**	**-1.43**	**0.0693**
GT_A_84_P769791	**TC358178**	**-0.70**	**0.0694**
GT_A_84_P757143	**AT2G36355**	**-0.81**	**0.0696**
GT_A_84_P851834	**AT5G23610**	**-0.56**	**0.0698**
GT_A_84_P217328	**AT4G05070**	**-1.69**	**0.0701**
GT_A_84_P22009	**AGL20**	**-1.58**	**0.0702**
GT_A_84_P21297	**AT3G55640**	**-0.83**	**0.0703**
GT_A_84_P10891	**AMP1**	**-0.64**	**0.0705**
GT_A_84_P16617	**ZFHD1**	**-1.29**	**0.0706**
GT_A_84_P12324	**AT1G70130**	**-1.57**	**0.0706**
GT_A_84_P23142	**AT3G45260**	**-1.18**	**0.0707**
GT_A_84_P558649	**AT1G35183**	**-0.73**	**0.0708**
GT_A_84_P132075	**SOUL-1**	**-0.67**	**0.0709**
GT_A_84_P13048	**AGL39**	**-1.38**	**0.0710**
GT_A_84_P832524	**TA49756_3702**	**-0.52**	**0.0711**
GT_A_84_P19181	**AK220677**	**-0.71**	**0.0713**
GT_A_84_P17695	**AT4G16790**	**-0.61**	**0.0716**
GT_A_84_P15621	**AT3G55550**	**-1.80**	**0.0717**
GT_A_84_P16344	**ACR5**	**-0.60**	**0.0722**
GT_A_84_P818774	**PGR5**	**-0.80**	**0.0722**
GT_A_84_P57860	**AT1G65490**	**-2.60**	**0.0723**
GT_A_84_P812437	**AT2G25450**	**-0.57**	**0.0725**
GT_A_84_P844790	**TC402262**	**-1.75**	**0.0725**
GT_A_84_P764204	**AT4G27810**	**-0.77**	**0.0725**
GT_At_Specific_00199496	**AT4G03610.1**	**-0.97**	**0.0726**
GT_A_84_P593234	**AT5G66070**	**-0.59**	**0.0727**
GT_A_84_P288954	**AT2G03020**	**-1.77**	**0.0729**
GT_A_84_P814789	**TA31166_3702**	**-1.16**	**0.0729**
GT_A_84_P822966	**TA38248_3702**	**-1.29**	**0.0730**
GT_A_84_P293424	**AT2G05510**	**-3.68**	**0.0732**
GT_A_84_P796216	**CPuORF54**	**-0.50**	**0.0732**
GT_A_84_P11230	**PORA**	**-1.68**	**0.0733**
GT_A_84_P799001	**AT2G07732**	**-0.94**	**0.0735**
GT_A_84_P21543	**APL1**	**-0.93**	**0.0740**

GT_A_84_P15536	**JR1**	**-0.68**	**0.0743**
GT_A_84_P767112	**MTHSC70-2**	**-1.07**	**0.0746**
GT_A_84_P15582	**AT3G46730**	**-2.46**	**0.0746**
GT_A_84_P22521	**AT5G38780**	**-2.25**	**0.0746**
GT_A_84_P810980	**TA28792_3702**	**-0.55**	**0.0750**
GT_A_84_P11323	**AT2G07701**	**-0.96**	**0.0753**
GT_A_84_P16699	**AT4G29930**	**-1.10**	**0.0753**
GT_A_84_P856574	**CDF2**	**-1.12**	**0.0754**
GT_A_84_P68014	**AT4G34150**	**-1.22**	**0.0757**
GT_A_84_P763091	**AT4G08870**	**-1.70**	**0.0758**
GT_A_84_P14677	**SBPASE**	**-1.29**	**0.0758**
GT_At_Specific_00132428	**AT3G01510.1**	**-0.70**	**0.0758**
GT_A_84_P788405	**AT1G36280**	**-0.86**	**0.0758**
GT_A_84_P795110	**AtGolS3**	**-2.65**	**0.0758**
GT_A_84_P108502	**PDF2.1**	**-1.40**	**0.0759**
GT_A_84_P265920	**AT3G18980**	**-0.72**	**0.0760**
GT_A_84_P560560	**AT2G05270**	**-0.92**	**0.0761**
GT_A_84_P809147	**PIP2A**	**-0.85**	**0.0762**
GT_A_84_P10378	**AT2G07691**	**-1.12**	**0.0762**
GT_A_84_P804048	**EDA30**	**-0.74**	**0.0763**
GT_A_84_P17530	**AT3G59820**	**-0.85**	**0.0763**
GT_A_84_P15250	**AT1G66760**	**-0.95**	**0.0765**
GT_A_84_P22031	**SNG1**	**-0.81**	**0.0769**
GT_A_84_P835829	**TA52200_3702**	**-0.64**	**0.0772**
GT_A_84_P270930	**RCA**	**-0.96**	**0.0773**
GT_A_84_P141439	**VSP2**	**-1.89**	**0.0774**
GT_A_84_P827406	**AT3G13227**	**-0.95**	**0.0774**
GT_A_84_P16177	**ATGA2OX1**	**-2.12**	**0.0780**
GT_A_84_P18071	**ATFD1**	**-1.03**	**0.0782**
GT_A_84_P787572	**AT4G15690**	**-1.47**	**0.0782**
GT_A_84_P849364	**DR225970**	**-0.87**	**0.0783**
GT_A_84_P784216	**VSP1**	**-2.61**	**0.0783**
GT_A_84_P15303	**AT1G55480**	**-1.32**	**0.0785**
GT_A_84_P22802	**AT1G72350**	**-1.75**	**0.0788**
GT_A_84_P23164	**CEJ1**	**-1.96**	**0.0789**
GT_A_84_P13526	**GAMMA-TIP**	**-0.75**	**0.0791**
GT_A_84_P12741	**TET3**	**-0.78**	**0.0794**
GT_A_84_P256160	**YCF37**	**-1.11**	**0.0794**
GT_A_84_P555957	**AT1G20740**	**-2.46**	**0.0795**
GT_A_84_P17194	**AT1G70880**	**-0.99**	**0.0796**
GT_At_Specific_00034137	**AT1G31060.1**	**-0.75**	**0.0797**
GT_A_84_P10849	**SYP72**	**-0.49**	**0.0798**
GT_A_84_P819826	**TA35177_3702**	**-1.03**	**0.0800**
GT_AntiSense_AtCg00340_184	**psaB**	**-0.71**	**0.0800**
GT_A_84_P188714	**AT3G06390**	**-1.50**	**0.0801**
GT_A_84_P19904	**ERD9**	**-0.83**	**0.0803**
GT_A_84_P19078	**AT1G19190**	**-0.85**	**0.0808**

GT_AntiSense_AtMg00150_609	orf116	-1.38	0.0809
GT_A_84_P596656	CAC1	-0.53	0.0810
GT_A_84_P853638	DR348682	-0.87	0.0811
GT_A_84_P13908	AT4G15460	-1.83	0.0812
GT_A_84_P21509	ATRBOHA	-0.78	0.0814
GT_A_84_P808762	FED_A	-0.69	0.0816
GT_A_84_P130476	AT5G62140	-0.73	0.0817
GT_AtMg01350_1165	orf145c	-0.71	0.0818
GT_A_84_P831180	AT1G77680	-0.90	0.0820
GT_At_Specific_00283677	AT5G42655.1	-1.10	0.0820
GT_A_84_P842278	HCF173	-0.94	0.0822
GT_A_84_P291574	ATMAMI	-0.72	0.0822
GT_A_84_P53450	AT1G79770	-1.21	0.0823
GT_A_84_P271510	AT1G76450	-0.95	0.0823
GT_A_84_P233279	AT1G64530	-0.58	0.0824
GT_A_84_P14786	anac011	-1.63	0.0824
GT_A_84_P129496	AT4G27700	-1.07	0.0828
GT_A_84_P809145	PIP2A	-0.76	0.0829
GT_A_84_P835360	AT1G15290	-0.72	0.0830
GT_A_84_P21719	AT5G38350	-2.39	0.0831
GT_A_84_P842034	NP231458	-2.05	0.0831
GT_A_84_P98066	AT1G77680	-0.77	0.0832
GT_A_84_P307130	NPQ4	-1.04	0.0833
GT_A_84_P17953	ACR8	-0.82	0.0833
GT_A_84_P821571	AT1G80270	-0.62	0.0835
GT_A_84_P811589	PSAN	-0.80	0.0839
GT_A_84_P557530	AT5G37478	-1.48	0.0840
GT_A_84_P15816	ATNAP9	-0.79	0.0844
GT_A_84_P803083		-1.70	0.0845
GT_A_84_P16449	ELIP1	-1.66	0.0845
GT_A_84_P768303	AT5G18407	-1.54	0.0848
GT_A_84_P852243	AT5G47430	-0.64	0.0853
GT_A_84_P12617	ANAC038	-1.98	0.0855
GT_A_84_P20029	MAPKKK13	-0.80	0.0859
GT_A_84_P807280	RCA	-1.62	0.0863
GT_A_84_P761415	AT3G42658	-1.76	0.0865
GT_A_84_P803572	AY299241	-0.69	0.0869
GT_A_84_P11955	CGA1	-1.18	0.0870
GT_A_84_P19734	BETA-OHASE_2	-0.81	0.0872
GT_A_84_P51860	AT3G19550	-1.37	0.0873
GT_A_84_P806205	RBCS1A	-0.55	0.0874
GT_A_84_P22745	AT1G03220	-1.01	0.0877
GT_A_84_P754928	AT2G40850	-1.37	0.0878
GT_A_84_P809138	TA27909_3702	-0.94	0.0879
GT_A_84_P817208	TA32958_3702	-0.71	0.0882
GT_A_84_P830995	LAS1	-2.54	0.0883
GT_A_84_P769565	AT5G44585	-1.04	0.0884

GT_A_84_P784916	ERD9	-0.83	0.0884
GT_A_84_P10924	BGLU7	-0.80	0.0884
GT_A_84_P19230	AT2G44800	-2.48	0.0884
GT_A_84_P595498	SDH2-1	-0.80	0.0886
GT_A_84_P848231	AT1G23740	-1.03	0.0889
GT_A_84_P12838	KEA3	-0.73	0.0894
GT_A_84_P161503	AT5G17300	-1.44	0.0894
GT_A_84_P270170	AT1G21100	-1.97	0.0895
GT_A_84_P20653	AT5G45220	-1.71	0.0895
GT_A_84_P16418	AT3G09440	-1.61	0.0896
GT_A_84_P792801	AT3G18530	-0.88	0.0897
GT_A_84_P255210	AT1G76110	-0.94	0.0898
GT_A_84_P721943		-1.08	0.0899
GT_A_84_P789231	AT5G60260	-1.26	0.0900
GT_A_84_P572473	AT4G21920	-1.57	0.0900
GT_A_84_P788197	AT2G32550	-3.11	0.0904
GT_A_84_P793157	AT1G16080	-0.65	0.0907
GT_A_84_P136665	AT4G15430	-1.93	0.0909
GT_A_84_P807037	LHB1B1	-0.78	0.0910
GT_A_84_P707595	AT5G52415	-3.43	0.0910
GT_A_84_P18659	TFL1	-1.90	0.0912
GT_A_84_P512672	MEE66	-0.98	0.0913
GT_A_84_P824779	AT4G01883	-1.25	0.0913
GT_A_84_P195044	AT1G09575	-1.00	0.0916
GT_A_84_P551331	AT5G42655	-1.19	0.0916
GT_A_84_P806774	AT3G09440	-1.61	0.0917
GT_A_84_P184234	AT3G23090	-0.65	0.0918
GT_A_84_P15827	UGT76C2	-1.08	0.0923
GT_A_84_P20043	AT1G54070	-1.62	0.0926
GT_A_84_P803809		-1.09	0.0927
GT_A_84_P90159	NF-YB8	-1.49	0.0927
GT_A_84_P15627	AT3G57020	-0.74	0.0929
GT_A_84_P11127	GUN5	-1.16	0.0929
GT_A_84_P140379	AT3G27350	-0.92	0.0929
GT_A_84_P21735	PHR1	-0.75	0.0931
GT_A_84_P807274	RCA	-1.74	0.0931
GT_At_Specific_00073730	AT1G74875.1	-0.80	0.0932
GT_A_84_P97266	CLE2	-1.82	0.0934
GT_AntiSense_AtMg00010_1220	orf153a	-0.83	0.0934
GT_A_84_P796526	AK230421	-1.17	0.0935
GT_A_84_P52180	AGL8	-1.89	0.0936
GT_A_84_P310503	TSD2	-1.07	0.0938
GT_A_84_P856604	TC397407	-1.05	0.0939
GT_A_84_P133465	AT2G40400	-1.10	0.0941
GT_A_84_P17689	CCT2	-1.87	0.0941
GT_A_84_P525040	AT1G65342	-2.18	0.0941
GT_A_84_P821845	ATNAC2	-0.86	0.0943

GT_A_84_P547884	AT5G64820	-1.56	0.0945
GT_A_84_P20858	AT1G60360	-0.99	0.0946
GT_A_84_P13276	AT1G04530	-1.14	0.0946
GT_A_84_P808972	LHCB6	-0.62	0.0948
GT_At_Specific_00227018	AT4G27290.1	-1.52	0.0952
GT_A_84_P270130	AT4G18440	-1.43	0.0957
GT_A_84_P552063	AT4G29750	-0.58	0.0958
GT_A_84_P23688	AT1G10410	-1.44	0.0958
GT_A_84_P14351	DRT112	-0.56	0.0958
GT_A_84_P791955	BX828813	-1.25	0.0959
GT_A_84_P11287	SP1L4	-1.71	0.0961
GT_A_84_P23310	AT4G27580	-0.78	0.0962
GT_A_84_P166573	AT1G03700	-0.85	0.0963
GT_A_84_P843872	AT1G53430	-1.81	0.0966
GT_A_84_P814898	TA31229_3702	-1.07	0.0967
GT_A_84_P816512	AT2G18690	-1.17	0.0968
GT_A_84_P16765	IMPA-9	-0.63	0.0968
GT_A_84_P807555	GAMMA-TIP	-0.91	0.0969
GT_A_84_P809013	LHCB6	-1.20	0.0970
GT_A_84_P784586	ATTPS-CIN	-1.77	0.0973
GT_A_84_P16114	AtGolS3	-5.01	0.0975
GT_A_84_P13173	MYBR1	-0.81	0.0975
GT_A_84_P803846	AT1G75190	-0.84	0.0976
GT_A_84_P76404	UNE11	-1.36	0.0976
GT_A_84_P255410	AT2G43340	-0.84	0.0976
GT_A_84_P862018	DR229968	-1.53	0.0981
GT_At_Specific_00058459	AT1G61750.1	-0.96	0.0981
GT_A_84_P803055	AK230207	-0.53	0.0987
GT_A_84_P823257	AT4G22235	-1.37	0.0988
GT_A_84_P13872	AT4G32190	-1.82	0.0988
GT_A_84_P251325	AT2G41050	-0.91	0.0990
GT_A_84_P54910	TC378941	-0.69	0.0999
GT_A_84_P262920	AT4G39610	-1.14	0.1000
GT_A_84_P555663	AT5G03545	-1.20	0.1001
GT_A_84_P13791	AT4G09110	-1.08	0.1002
GT_A_84_P847363	RCA	-1.58	0.1002
GT_A_84_P856041	AT1G80620	-0.81	0.1005
GT_At_Specific_00314270	TRNI.4	-1.57	0.1005
GT_A_84_P23194	CRD1	-0.99	0.1009
GT_A_84_P813537	SBPASE	-1.36	0.1012
GT_A_84_P17992	AT1G08790	-0.81	0.1013
GT_A_84_P16314	AT2G22590	-1.00	0.1016
GT_A_84_P22231	AT3G53230	-1.21	0.1024
GT_A_84_P770026	TA49214_3702	-0.70	0.1025
GT_AntiSense_AtMg00180_635	ccb452	-0.49	0.1028
GT_A_84_P860821	AT4G16155	-1.11	0.1028
GT_A_84_P272560	AT3G12970	-1.06	0.1029

GT_A_84_P10196	**NIT4**	**-0.87**	**0.1032**
GT_A_84_P18621	**HAT22**	**-0.95**	**0.1033**
GT_A_84_P199214	**AT1G22630**	**-1.35**	**0.1035**
GT_A_84_P573880	**AT1G79150**	**-1.07**	**0.1036**
GT_A_84_P66364	**AT1G49310**	**-1.43**	**0.1037**
GT_A_84_P18568	**ACP4**	**-0.93**	**0.1037**
GT_A_84_P852984	**AT5G51040**	**-0.71**	**0.1042**
GT_A_84_P74934	**AT4G17800**	**-0.97**	**0.1043**
GT_A_84_P79769	**AT4G38470**	**-1.39**	**0.1043**
GT_A_84_P18577	**AT4G26860**	**-1.01**	**0.1046**
GT_A_84_P21645	**AT5G58110**	**-0.55**	**0.1048**
GT_A_84_P869736	**AGL42**	**-1.57**	**0.1048**
GT_A_84_P701162	**TC364870**	**-1.73**	**0.1051**
GT_A_84_P153218	**AT4G21780**	**-1.29**	**0.1053**
GT_A_84_P833267	**AT4G02850**	**-1.81**	**0.1053**
GT_A_84_P287970	**PCK2**	**-2.98**	**0.1054**
GT_A_84_P500838	**PHOT2**	**-1.08**	**0.1055**
GT_A_84_P13607	**AT3G06830**	**-2.93**	**0.1055**
GT_A_84_P97476	**VSP1**	**-2.74**	**0.1055**
GT_A_84_P293774	**VTC5**	**-1.13**	**0.1056**
GT_A_84_P18190	**AT2G16960**	**-1.36**	**0.1057**
GT_A_84_P14538	**AT3G04570**	**-1.11**	**0.1058**
GT_A_84_P755424	**AT2G15990**	**-2.37**	**0.1066**
GT_A_84_P20835	**CCR2**	**-1.17**	**0.1066**
GT_A_84_P21606	**ATBI1**	**-0.57**	**0.1066**
GT_A_84_P573251	**AT3G47200**	**-2.31**	**0.1071**
GT_A_84_P19223	**ATRABA2B**	**-0.85**	**0.1075**
GT_A_84_P86169	**LSH9**	**-1.03**	**0.1075**
GT_A_84_P822626	**TA37874_3702**	**-1.32**	**0.1076**
GT_A_84_P726299	**TA34141_3702**	**-1.18**	**0.1078**
GT_AtCg00190_145	**rpoB**	**-0.91**	**0.1079**
GT_A_84_P570378	**AT2G28690**	**-1.10**	**0.1079**
GT_A_84_P823227	**AT5G17300**	**-1.34**	**0.1080**
GT_A_84_P528692	**AT2G24030**	**-1.06**	**0.1082**
GT_A_84_P247425	**AT4G04990**	**-2.09**	**0.1084**
GT_A_84_P857762	**VSP2**	**-1.86**	**0.1084**
GT_At_Specific_00152776	**AT3G18530.1**	**-1.03**	**0.1085**
GT_A_84_P22296	**AT4G05010**	**-0.74**	**0.1086**
GT_A_84_P201258	**AT3G16280**	**-1.09**	**0.1086**
GT_A_84_P840627	**NP221616**	**-0.88**	**0.1087**
GT_A_84_P756441	**AT2G07769**	**-0.99**	**0.1087**
GT_A_84_P591506	**AT1G05870**	**-0.96**	**0.1092**
GT_A_84_P701249	**AT2G16586**	**-0.57**	**0.1093**
GT_A_84_P10977	**ATTRANS11**	**-1.26**	**0.1093**
GT_A_84_P787555	**ATMAP70-5**	**-0.97**	**0.1098**
GT_A_84_P131606	**HCF173**	**-1.04**	**0.1098**
GT_A_84_P13218	**AT2G07681**	**-1.15**	**0.1100**

GT_A_84_P22476	AT1G68670	-0.96	0.1101
GT_A_84_P842560	AT1G07650	-0.99	0.1101
GT_A_84_P302880	AT3G12345	-0.72	0.1104
GT_A_84_P724266		-1.12	0.1111
GT_A_84_P19231	ATHSFA2	-3.86	0.1112
GT_A_84_P17442	CYP71B36	-1.11	0.1112
GT_A_84_P18976	AT1G30730	-1.38	0.1114
GT_A_84_P567310	AT5G47455	-1.49	0.1116
GT_A_84_P812187	PDH-E1_BETA	-0.54	0.1119
GT_A_84_P20591	AT5G17820	-0.87	0.1125
GT_A_84_P14822	MYB69	-1.18	0.1126
GT_A_84_P10138	AT4G37190	-0.60	0.1128
GT_A_84_P57900	BHLH039	-3.02	0.1128
GT_A_84_P21336	ATSTE24	-0.66	0.1132
GT_A_84_P808192	MLP34	-1.61	0.1135
GT_A_84_P560548	AT2G01310	-0.65	0.1135
GT_A_84_P767969	TT16	-0.89	0.1136
GT_AntiSense_AtMg00300_687	orf145a	-1.16	0.1136
GT_A_84_P23614	AT2G07738	-1.02	0.1137
GT_AntiSense_AtCg00760_372	rpl36	-0.61	0.1139
GT_A_84_P17108	AT1G71000	-4.12	0.1140
GT_A_84_P729644	AY334555	-1.51	0.1143
GT_A_84_P840629	NP221627	-0.86	0.1144
GT_A_84_P11412	ARR4	-1.55	0.1145
GT_A_84_P826127	TA42031_3702	-1.52	0.1145
GT_A_84_P840927	ATMAMI	-0.68	0.1146
GT_A_84_P108002	AT1G18060	-1.21	0.1146
GT_A_84_P11721	ANAC055	-1.91	0.1147
GT_A_84_P820471	TA35675_3702	-1.05	0.1151
GT_A_84_P845875	AT4G27700	-0.85	0.1151
GT_A_84_P14424	AGL17	-1.60	0.1152
GT_A_84_P11223	AT5G52020	-1.19	0.1152
GT_A_84_P21689	AT5G15750	-0.59	0.1153
GT_A_84_P17027	AT1G30870	-1.43	0.1154
GT_A_84_P766364	AT5G31770	-1.10	0.1154
GT_A_84_P13156	AT5G63120	-0.68	0.1155
GT_A_84_P259610	AT3G01240	-2.69	0.1158
GT_A_84_P20736	HB53	-2.13	0.1160
GT_A_84_P20632	CDF2	-1.12	0.1161
GT_A_84_P736168	AT2G07715	-0.48	0.1165
GT_A_84_P860972	AT2G25450	-1.19	0.1170
GT_A_84_P59510	AT4G32480	-1.86	0.1171
GT_A_84_P151158	OXS3	-1.55	0.1172
GT_A_84_P271080	AT3G56600	-1.19	0.1175
GT_A_84_P799245	AT1G26220	-0.71	0.1175
GT_A_84_P819203	AT5G64460	-0.57	0.1176
GT_A_84_P120342	NP502678	-0.75	0.1178

GT_A_84_P85639	AT3G24190	-1.07	0.1179
GT_A_84_P818353	TA33911_3702	-1.15	0.1179
GT_A_84_P720290	AT1G53633	-1.19	0.1180
GT_A_84_P807303	RCA	-1.70	0.1181
GT_A_84_P858176	PIP2A	-1.14	0.1181
GT_A_84_P18416	AT3G46080	-1.66	0.1182
GT_At_Specific_00286222	MAP18	-1.38	0.1185
GT_A_84_P270090	AT5G11890	-0.56	0.1187
GT_A_84_P855265	YCF37	-0.97	0.1187
GT_A_84_P851931	PROT1	-0.75	0.1188
GT_A_84_P23859	WRKY25	-0.74	0.1188
GT_A_84_P21366	ATMPK5	-0.62	0.1189
GT_A_84_P10144	AT5G02170	-0.76	0.1190
GT_A_84_P10157	AT5G06330	-0.83	0.1192
GT_A_84_P172941	AT2G18690	-1.21	0.1193
GT_A_84_P762555	AT3G05858	-0.81	0.1194
GT_A_84_P586938	ATFRO7	-1.05	0.1197
GT_A_84_P752675	AT1G09157	-1.82	0.1198
GT_A_84_P787539	ATTRANS11	-1.27	0.1200
GT_A_84_P179234	AT5G46770	-0.74	0.1200
GT_A_84_P17109	AT1G74770	-1.56	0.1203
GT_A_84_P789523	AT5G03545	-1.11	0.1206
GT_A_84_P555310	AT2G36650	-1.58	0.1206
GT_A_84_P12766	F3H	-2.13	0.1207
GT_A_84_P768577	AT5G36350	-1.25	0.1212
GT_A_84_P20376	NGA2	-0.54	0.1218
GT_A_84_P602497	COPT4	-0.60	0.1218
GT_A_84_P763057	AT4G14740	-0.85	0.1219
GT_A_84_P845206	TC369211	-1.83	0.1220
GT_A_84_P846989	AT1G22630	-1.17	0.1221
GT_A_84_P544120	AT4G32860	-1.56	0.1224
GT_A_84_P22798	ATGSTU23	-1.21	0.1227
GT_A_84_P19362	ZAT7	-1.38	0.1228
GT_A_84_P69564	AT3G21710	-0.84	0.1229
GT_At_Specific_00168552	AT3G32050.1	-1.35	0.1229
GT_A_84_P730916	TA27785_3702	-1.08	0.1230
GT_A_84_P855665	AT2G18200	-0.92	0.1231
GT_A_84_P168633	AT3G21920	-1.36	0.1234
GT_At_Specific_00088126	AT2G07767.1	-0.83	0.1238
GT_A_84_P17347	AT2G25450	-0.82	0.1243
GT_A_84_P11867	AT3G62040	-0.61	0.1250
GT_A_84_P795100	EIF4E	-0.74	0.1252
GT_A_84_P305730	AT5G24155	-0.79	0.1260
GT_A_84_P821849	AT3G27350	-1.03	0.1260
GT_A_84_P838790	TC395900	-1.11	0.1261
GT_A_84_P178164	MLP34	-1.64	0.1261
GT_A_84_P788408	AT2G22760	-2.73	0.1262

GT_A_84_P23290	**AT4G23290**	**-1.53**	**0.1263**
GT_A_84_P126091	**LACS2**	**-0.72**	**0.1264**
GT_A_84_P14572	**ASK10**	**-1.64**	**0.1265**
GT_A_84_P15977	**ADF11**	**-2.46**	**0.1267**
GT_A_84_P859685	**AT1G68670**	**-0.90**	**0.1271**
GT_A_84_P840181	**AT3G48450**	**-0.70**	**0.1273**
GT_A_84_P765821	**AT4G26701**	**-2.22**	**0.1277**
GT_A_84_P59020	**MAP18**	**-1.47**	**0.1283**
GT_A_84_P19702	**POP1**	**-1.49**	**0.1285**
GT_A_84_P13826	**BGLU47**	**-2.16**	**0.1286**
GT_A_84_P793950	**CPN60A**	**-0.88**	**0.1287**
GT_A_84_P17821	**BOU**	**-1.09**	**0.1288**
GT_A_84_P851348	**BE037627**	**-1.23**	**0.1291**
GT_A_84_P839982	**AT1G63410**	**-2.64**	**0.1295**
GT_A_84_P588148	**AT2G07774**	**-0.80**	**0.1297**
GT_A_84_P10611	**AT2G43530**	**-0.96**	**0.1305**
GT_A_84_P21706	**AT5G19760**	**-0.45**	**0.1305**
GT_A_84_P869688	**AGP30**	**-1.77**	**0.1313**
GT_A_84_P603549	**AT3G51325**	**-0.90**	**0.1315**
GT_A_84_P182114	**AT5G02950**	**-1.03**	**0.1319**
GT_A_84_P786051	**AT4G18440**	**-1.42**	**0.1323**
GT_A_84_P15467	**AT3G01290**	**-1.02**	**0.1326**
GT_A_84_P19845	**ATMYB21**	**-3.48**	**0.1330**
GT_A_84_P20876	**ATAMT1;2**	**-2.33**	**0.1332**
GT_A_84_P521598	**AT2G22760**	**-2.83**	**0.1333**
GT_A_84_P535673	**AT1G61430**	**-2.32**	**0.1334**
GT_A_84_P20137	**AT2G35800**	**-0.87**	**0.1335**
GT_A_84_P761565	**AT3G10185**	**-1.85**	**0.1336**
GT_At_Specific_00294567	**AT5G51780.1**	**-1.72**	**0.1338**
GT_A_84_P868030	**AT3G27415**	**-1.47**	**0.1339**
GT_A_84_P807004	**TA26724_3702**	**-1.33**	**0.1346**
GT_AntiSense_AtCg00640_307	**rpl33**	**-0.92**	**0.1346**
GT_A_84_P788781	**BIP3**	**-1.44**	**0.1347**
GT_A_84_P868622	**AT3G56600**	**-0.96**	**0.1349**
GT_A_84_P87399	**AT3G06520**	**-1.28**	**0.1350**
GT_A_84_P517322	**AT3G19200**	**-1.10**	**0.1354**
GT_A_84_P799545	**AT2G30540**	**-0.80**	**0.1355**
GT_A_84_P17752	**AT5G17310**	**-0.76**	**0.1356**
GT_A_84_P195994	**TC383177**	**-2.72**	**0.1358**
GT_AtCg01120_516	**rps15**	**-0.50**	**0.1358**
GT_A_84_P531425	**BAG6**	**-2.65**	**0.1359**
GT_A_84_P549263	**AT3G32980**	**-1.06**	**0.1363**
GT_A_84_P750651	**AT1G59930**	**-1.79**	**0.1364**
GT_A_84_P16212	**AT1G74470**	**-0.68**	**0.1365**
GT_A_84_P18829	**ARR6**	**-1.73**	**0.1367**
GT_A_84_P101316	**RPS15AD**	**-0.48**	**0.1371**
GT_A_84_P171243	**AT5G42070**	**-1.06**	**0.1377**

GT_A_84_P806740	**CAT1**	**-1.03**	**0.1377**
GT_A_84_P812725	**ATPHB3**	**-0.67**	**0.1378**
GT_A_84_P799867	**AT4G27700**	**-1.25**	**0.1378**
GT_A_84_P12848	**ATPD**	**-0.80**	**0.1380**
GT_A_84_P555590	**AT4G22212**	**-1.94**	**0.1381**
GT_A_84_P869924	**AT4G26580**	**-0.57**	**0.1383**
GT_A_84_P23101	**AT3G21890**	**-1.25**	**0.1385**
GT_A_84_P846226		**-1.83**	**0.1386**
GT_A_84_P22015	**ATGA2OX2**	**-0.85**	**0.1388**
GT_A_84_P16820	**SQE6**	**-0.56**	**0.1388**
GT_A_84_P98716	**AT4G00390**	**-0.75**	**0.1388**
GT_A_84_P809007	**TA27833_3702**	**-1.32**	**0.1389**
GT_A_84_P713101	**AT4G28850**	**-0.97**	**0.1389**
GT_A_84_P799556	**AT1G26850**	**-1.03**	**0.1393**
GT_A_84_P13455	**SIP4**	**-0.86**	**0.1395**
GT_A_84_P822530	**SIP4**	**-1.01**	**0.1397**
GT_A_84_P23620	**AT2G07675**	**-1.02**	**0.1399**
GT_A_84_P19216	**AT2G21330**	**-1.54**	**0.1401**
GT_A_84_P20351	**emb1624**	**-0.71**	**0.1402**
GT_A_84_P768597	**AT5G35698**	**-0.89**	**0.1409**
GT_A_84_P21293	**CXIP1**	**-0.71**	**0.1413**
GT_A_84_P242573	**AT5G54470**	**-1.66**	**0.1414**
GT_A_84_P844083	**AT2G07721**	**-1.04**	**0.1417**
GT_A_84_P809928	**GDH2**	**-1.06**	**0.1426**
GT_A_84_P12169	**CYP715A1**	**-1.82**	**0.1436**
GT_A_84_P797883	**EMB2454**	**-0.63**	**0.1438**
GT_A_84_P258360	**AT5G26290**	**-1.78**	**0.1442**
GT_A_84_P808808	**ALDH11A3**	**-1.17**	**0.1445**
GT_A_84_P851319	**MYB59**	**-0.94**	**0.1446**
GT_A_84_P10672	**BGLU15**	**-0.76**	**0.1447**
GT_A_84_P19259	**UNE9**	**-0.89**	**0.1454**
GT_A_84_P10407	**AT1G23740**	**-0.96**	**0.1456**
GT_A_84_P830125	**BAG6**	**-2.91**	**0.1457**
GT_A_84_P803103	**AT2G07691**	**-1.03**	**0.1458**
GT_A_84_P556490	**AT4G23493**	**-1.81**	**0.1460**
GT_A_84_P853962	**AT2G46090**	**-0.72**	**0.1460**
GT_A_84_P800991	**COR47**	**-0.93**	**0.1465**
GT_At_Specific_00270015	**AGL26**	**-1.27**	**0.1468**
GT_A_84_P20447	**CPK15**	**-1.02**	**0.1471**
GT_A_84_P18125	**AT1G75280**	**-0.91**	**0.1471**
GT_A_84_P812160	**TA29402_3702**	**-1.16**	**0.1474**
GT_A_84_P58750	**AT5G21950**	**-1.38**	**0.1481**
GT_A_84_P12334	**AT1G09360**	**-3.61**	**0.1485**
GT_A_84_P17137	**ATNAP5**	**-0.95**	**0.1490**
GT_A_84_P813108	**AT2G04039**	**-1.42**	**0.1494**
GT_A_84_P547965	**AT1G12030**	**-0.88**	**0.1499**
GT_A_84_P20004	**AT1G72660**	**-1.62**	**0.1504**

GT_A_84_P843362	**NP396445**	**-1.67**	**0.1506**
GT_A_84_P13499	**AT2G14160**	**-1.92**	**0.1507**
GT_A_84_P13023	**ATF2**	**-0.56**	**0.1507**
GT_A_84_P11731	**MBF1C**	**-1.90**	**0.1508**
GT_A_84_P24132	**TT5**	**-1.09**	**0.1511**
GT_A_84_P24110	**AT3G50270**	**-1.17**	**0.1513**
GT_A_84_P12196	**AT5G59750**	**-0.64**	**0.1517**
GT_A_84_P863513	**BP663997**	**-0.69**	**0.1517**
GT_A_84_P850629	**TC393095**	**-1.28**	**0.1519**
GT_A_84_P141219	**AT5G35320**	**-0.74**	**0.1522**
GT_A_84_P805977	**AT1G77940**	**-0.63**	**0.1522**
GT_A_84_P802924	**EG495729**	**-1.17**	**0.1523**
GT_A_84_P522932	**AT5G21222**	**-0.75**	**0.1527**
GT_A_84_P270820	**ATPUP15**	**-2.84**	**0.1530**
GT_A_84_P785478	**TSD2**	**-1.06**	**0.1535**
GT_A_84_P21361	**ATSPS4F**	**-1.30**	**0.1536**
GT_A_84_P868217	**MBP2**	**-0.98**	**0.1539**
GT_A_84_P15731	**ATHSP23.6-MIT**	**-3.03**	**0.1552**
GT_A_84_P58520	**AT2G04039**	**-1.48**	**0.1554**
GT_A_84_P833758	**AT4G13860**	**-0.83**	**0.1556**
GT_A_84_P23931	**ORP1A**	**-1.29**	**0.1557**
GT_A_84_P18813	**AT5G59490**	**-1.22**	**0.1562**
GT_A_84_P807322	**RCA**	**-1.65**	**0.1570**
GT_A_84_P835788	**AT1G61860**	**-0.86**	**0.1572**
GT_A_84_P203838	**AT2G07676**	**-1.24**	**0.1573**
GT_A_84_P819063	**PCL1**	**-0.82**	**0.1575**
GT_A_84_P117052	**AT3G25640**	**-1.29**	**0.1575**
GT_A_84_P787143	**AT5G49560**	**-1.07**	**0.1577**
GT_A_84_P12404	**BRL1**	**-0.57**	**0.1578**
GT_A_84_P603601	**AT4G02850**	**-1.62**	**0.1580**
GT_A_84_P856185	**AT3G25930**	**-1.50**	**0.1580**
GT_A_84_P21742	**AT1G18990**	**-2.70**	**0.1583**
GT_A_84_P768248	**AT5G23035**	**-0.76**	**0.1585**
GT_A_84_P15617	**PIP2;5**	**-0.76**	**0.1586**
GT_A_84_P823687	**AT5G47430**	**-0.67**	**0.1587**
GT_A_84_P844671	**AT4G38470**	**-1.30**	**0.1591**
GT_A_84_P818265	**AT4G32480**	**-1.62**	**0.1594**
GT_A_84_P11098	**CAT6**	**-1.72**	**0.1595**
GT_A_84_P176194	**AT5G02090**	**-0.87**	**0.1597**
GT_A_84_P182224	**AT1G29700**	**-0.83**	**0.1597**
GT_A_84_P829388	**MAPKKK13**	**-0.76**	**0.1597**
GT_A_84_P840449		**-0.60**	**0.1602**
GT_A_84_P24130	**AT3G54700**	**-1.28**	**0.1602**
GT_A_84_P10011	**AT4G09350**	**-1.06**	**0.1603**
GT_At_Specific_00314190	**TRNR.2**	**-0.86**	**0.1604**
GT_A_84_P813841	**TA30478_3702**	**-1.35**	**0.1612**
GT_A_84_P760530	**AT3G06437**	**-1.15**	**0.1615**

GT_AntiSense_AtCg00650_312	**rps18**	**-0.61**	**0.1615**
GT_A_84_P594516	**AT2G46740**	**-1.36**	**0.1617**
GT_A_84_P795798	**SDC**	**-1.45**	**0.1623**
GT_AtMg00400_703	**orf157**	**-0.67**	**0.1626**
GT_A_84_P16494	**CYCP2;1**	**-1.25**	**0.1629**
GT_A_84_P587368	**AT2G42140**	**-2.58**	**0.1630**
GT_A_84_P17461	**AT3G43430**	**-0.85**	**0.1634**
GT_A_84_P162443	**AT5G64510**	**-2.36**	**0.1639**
GT_A_84_P19025	**SHM7**	**-0.79**	**0.1639**
GT_A_84_P23413	**TET16**	**-1.16**	**0.1639**
GT_A_84_P797619	**PORA**	**-1.64**	**0.1641**
GT_At_Specific_00300952	**AT5G57181.1**	**-1.11**	**0.1641**
GT_A_84_P20994	**IAA18**	**-0.83**	**0.1643**
GT_At_Specific_00314021	**TRNV.3**	**-1.15**	**0.1644**
GT_A_84_P829837	**TA46917_3702**	**-1.05**	**0.1656**
GT_A_84_P94599	**AT3G56290**	**-1.71**	**0.1656**
GT_A_84_P10327	**ATGCN5**	**-0.86**	**0.1657**
GT_A_84_P121232	**AT2G03240**	**-0.92**	**0.1661**
GT_A_84_P15366	**AT1G11210**	**-0.71**	**0.1665**
GT_A_84_P13389	**LPR2**	**-0.78**	**0.1667**
GT_A_84_P541962	**AT2G16385**	**-1.32**	**0.1670**
GT_A_84_P20747	**UGT78D2**	**-1.44**	**0.1672**
GT_A_84_P803062	**ERD5**	**-1.65**	**0.1673**
GT_A_84_P830000	**NF-YB10**	**-0.68**	**0.1674**
GT_A_84_P22169	**AT3G22530**	**-0.80**	**0.1674**
GT_A_84_P281300	**PCL1**	**-0.94**	**0.1677**
GT_A_84_P524838	**AT5G66740**	**-2.45**	**0.1677**
GT_A_84_P124471	**AT2G41120**	**-0.84**	**0.1682**
GT_At_Specific_00226582	**AT4G26860.1**	**-1.23**	**0.1682**
GT_A_84_P141359	**AT2G24550**	**-0.87**	**0.1684**
GT_A_84_P11712	**AT3G02810**	**-1.76**	**0.1684**
GT_A_84_P794466	**AT1G33600**	**-0.56**	**0.1686**
GT_A_84_P819138	**AT1G66330**	**-0.60**	**0.1689**
GT_A_84_P845601	**IAA18**	**-0.92**	**0.1690**
GT_A_84_P750559	**AT1G53870**	**-1.12**	**0.1691**
GT_A_84_P814296	**ATCTH**	**-1.45**	**0.1699**
GT_A_84_P10384	**COR47**	**-0.81**	**0.1701**
GT_A_84_P20014	**AT1G75220**	**-0.65**	**0.1709**
GT_A_84_P21046	**AT2G38600**	**-1.09**	**0.1712**
GT_A_84_P807539	**F3H**	**-2.10**	**0.1717**
GT_A_84_P542646	**AT1G15040**	**-3.04**	**0.1717**
GT_A_84_P178774	**BHLH100**	**-3.35**	**0.1726**
GT_A_84_P765764	**AT4G21926**	**-0.88**	**0.1726**
GT_A_84_P532318	**AT2G47370**	**-1.43**	**0.1732**
GT_A_84_P12503	**ATCTH**	**-1.36**	**0.1733**
GT_A_84_P559372	**AT5G61260**	**-1.53**	**0.1734**
GT_A_84_P812870	**TA29821_3702**	**-1.17**	**0.1735**

GT_A_84_P111332	AT1G56210	-1.11	0.1738
GT_A_84_P529724	AT3G20340	-2.08	0.1747
GT_A_84_P19577	SHM4	-1.11	0.1750
GT_A_84_P17129	AT1G70500	-0.91	0.1754
GT_A_84_P832673	AT3G20340	-2.13	0.1757
GT_A_84_P136929	AT1G44920	-0.64	0.1758
GT_A_84_P12482	SR45	-0.81	0.1758
GT_A_84_P818172	AT1G15040	-3.15	0.1759
GT_A_84_P765341	AT4G24973	-2.65	0.1760
GT_A_84_P761328	AT3G12977	-1.15	0.1760
GT_A_84_P786590	AT4G15430	-1.56	0.1761
GT_A_84_P841014	AT5G60260	-1.80	0.1768
GT_A_84_P22197	AT1G02620	-2.18	0.1770
GT_A_84_P850921	AT2G21330	-1.39	0.1775
GT_A_84_P569216	AT1G08845	-0.80	0.1782
GT_A_84_P837766	AT2G44800	-1.82	0.1791
GT_A_84_P859455	IAA18	-0.79	0.1792
GT_A_84_P561436	AT2G07749	-1.25	0.1794
GT_A_84_P818893	AT1G23740	-1.02	0.1808
GT_A_84_P750646	AT1G59920	-1.68	0.1822
GT_At_Specific_00023859	emb2170	-0.63	0.1825
GT_A_84_P97856	AT2G06040	-1.18	0.1826
GT_A_84_P19854	AT1G17690	-0.70	0.1827
GT_At_Specific_00314000	TRNL.4	-1.06	0.1836
GT_A_84_P15415	XTH21	-1.27	0.1838
GT_A_84_P829094	TA45827_3702	-1.00	0.1841
GT_A_84_P855440	AT3G15450	-1.70	0.1848
GT_A_84_P299940	AT1G01355	-1.20	0.1852
GT_A_84_P814403	TA30874_3702	-0.55	0.1854
GT_A_84_P849379	TC381400	-2.24	0.1854
GT_A_84_P762988	MAPKKK21	-2.12	0.1855
GT_A_84_P792453	AT4G08874	-1.42	0.1861
GT_A_84_P799948	DQ108843	-2.48	0.1861
GT_A_84_P10660	ATGSTU1	-0.79	0.1865
GT_A_84_P848657	TC395057	-1.37	0.1867
GT_A_84_P770257	AT2G07674	-0.72	0.1870
GT_A_84_P81409	AT3G15630	-1.63	0.1871
GT_A_84_P12343	ATRRP4	-0.77	0.1878
GT_At_Specific_00052106	AT1G54750.1	-2.49	0.1880
GT_A_84_P807334	RCA	-1.20	0.1883
GT_A_84_P557587	AT5G54095	-1.09	0.1887
GT_A_84_P830119	BAG6	-2.79	0.1888
GT_A_84_P22792	AT1G14890	-1.22	0.1892
GT_A_84_P827457	TA43605_3702	-2.65	0.1895
GT_A_84_P168683	AT2G40960	-0.49	0.1901
GT_A_84_P12987	AT5G04600	-0.61	0.1904
GT_A_84_P17864	CYP86A1	-1.18	0.1925

GT_At_Specific_00291240	**ATSDI1**	**-0.95**	**0.1943**
GT_At_Specific_00123052	**AT2G40960.1**	**-0.51**	**0.1947**
GT_A_84_P23973	**AtMS2**	**-1.53**	**0.1948**
GT_AtMg01110_1058	**orf251**	**-1.43**	**0.1950**
GT_A_84_P12457	**ST4B**	**-2.00**	**0.1950**
GT_A_84_P831247	**AT5G43520**	**-2.42**	**0.1952**
GT_A_84_P15703	**AT4G18340**	**-1.10**	**0.1958**
GT_A_84_P256030	**EDA41**	**-1.30**	**0.1960**
GT_A_84_P10810	**ATMYB0**	**-1.13**	**0.1968**
GT_A_84_P754390	**AT1G67856**	**-1.16**	**0.1968**
GT_A_84_P255500	**AT5G54130**	**-1.98**	**0.1973**
GT_A_84_P11597	**AGO6**	**-1.26**	**0.1974**
GT_A_84_P503428	**AGP41**	**-0.50**	**0.1975**
GT_A_84_P756195	**AT2G04070**	**-1.71**	**0.1978**
GT_A_84_P797196	**EG440258**	**-2.46**	**0.1982**
GT_A_84_P851677	**LHCB3**	**-1.26**	**0.1988**
GT_A_84_P862124	**AT4G01000**	**-0.51**	**0.1988**
GT_A_84_P821066	**AGL8**	**-1.91**	**0.1990**
GT_A_84_P806997	**LHCB3**	**-1.10**	**0.1991**
GT_A_84_P284920	**AT3G01060**	**-1.75**	**0.1993**
GT_A_84_P602531	**PAP14**	**-3.86**	**0.1993**
GT_A_84_P863621	**AGL17**	**-1.21**	**0.2005**
GT_A_84_P572089	**AT1G78990**	**-1.75**	**0.2008**
GT_A_84_P812864	**SHM4**	**-1.17**	**0.2009**
GT_A_84_P287770	**AtPP2-A13**	**-0.58**	**0.2018**
GT_A_84_P861938	**TA27368_3702**	**-1.04**	**0.2019**
GT_A_84_P796552	**AK229901**	**-1.88**	**0.2020**
GT_A_84_P19943	**AT1G49450**	**-0.79**	**0.2025**
GT_A_84_P855831	**AT4G21215**	**-0.95**	**0.2033**
GT_A_84_P812448	**VTC2**	**-1.08**	**0.2040**
GT_A_84_P223359	**AT1G54000**	**-1.44**	**0.2041**
GT_At_Specific_00028480	**AT1G26220.1**	**-0.51**	**0.2044**
GT_A_84_P561767	**AT4G14240**	**-0.55**	**0.2045**
GT_A_84_P867212	**SHM7**	**-0.78**	**0.2048**
GT_A_84_P825746	**HSA32**	**-1.85**	**0.2052**
GT_A_84_P791663	**AT4G01535**	**-0.61**	**0.2056**
GT_A_84_P793549	**AT5G65480**	**-0.61**	**0.2059**
GT_A_84_P11982	**CYP96A2**	**-0.92**	**0.2074**
GT_A_84_P20690	**AT5G55560**	**-0.59**	**0.2080**
GT_A_84_P106762	**AT3G25930**	**-1.51**	**0.2080**
GT_A_84_P21961	**PHT4;1**	**-0.65**	**0.2083**
GT_A_84_P15686	**ATHSP22.0**	**-3.84**	**0.2105**
GT_A_84_P805073	**TA25819_3702**	**-2.11**	**0.2106**
GT_A_84_P801042	**PIP1B**	**-0.49**	**0.2107**
GT_A_84_P12793	**AT3G57190**	**-0.99**	**0.2109**
GT_A_84_P851410	**BE039144**	**-1.88**	**0.2113**
GT_A_84_P752326	**AT1G62420**	**-1.33**	**0.2116**

GT_A_84_P17365	**ATERDJ3A**	**-1.94**	**0.2126**
GT_A_84_P10862	**ARR5**	**-1.04**	**0.2129**
GT_A_84_P819199	**AT5G64460**	**-0.59**	**0.2133**
GT_A_84_P822358	**ATMAMI**	**-0.51**	**0.2140**
GT_A_84_P15345	**LAC3**	**-0.92**	**0.2142**
GT_A_84_P580907	**AT1G53035**	**-1.03**	**0.2143**
GT_A_84_P17954	**AT3G19085**	**-0.98**	**0.2147**
GT_A_84_P868534	**AT3G01060**	**-1.58**	**0.2149**
GT_AntiSense_AtMg00140_604	**orf167**	**-1.03**	**0.2152**
GT_A_84_P828428	**AT3G06660**	**-0.79**	**0.2154**
GT_A_84_P812444	**AT2G25450**	**-1.21**	**0.2157**
GT_A_84_P20023	**NDA1**	**-1.73**	**0.2157**
GT_A_84_P12666	**ATNAC2**	**-0.80**	**0.2164**
GT_A_84_P834298	**PORA**	**-1.84**	**0.2168**
GT_A_84_P18106	**AOAT2**	**-0.62**	**0.2169**
GT_A_84_P751510	**TC368609**	**-1.29**	**0.2171**
GT_A_84_P297914	**AtRLP55**	**-0.55**	**0.2171**
GT_A_84_P816246	**ATCAD4**	**-1.17**	**0.2172**
GT_A_84_P309133	**AT5G18065**	**-1.26**	**0.2173**
GT_A_84_P786169	**CaS**	**-1.16**	**0.2179**
GT_A_84_P21203	**AT1G26730**	**-1.27**	**0.2189**
GT_A_84_P297414	**AT3G62070**	**-1.22**	**0.2191**
GT_A_84_P18332	**GAPC1**	**-0.70**	**0.2194**
GT_A_84_P825453	**TA41237_3702**	**-1.25**	**0.2196**
GT_A_84_P22675	**AT1G30760**	**-1.50**	**0.2200**
GT_A_84_P148568	**AT5G02180**	**-0.74**	**0.2202**
GT_A_84_P68154	**AT3G22660**	**-0.83**	**0.2203**
GT_A_84_P836976	**TA52715_3702**	**-3.02**	**0.2208**
GT_A_84_P761408	**AT3G44042**	**-1.03**	**0.2211**
GT_A_84_P850060	**AT1G74770**	**-1.94**	**0.2215**
GT_A_84_P111682	**AT5G43520**	**-2.68**	**0.2216**
GT_A_84_P167173	**DREB1A**	**-1.25**	**0.2219**
GT_AtMg00770_899	**orf100b**	**-0.61**	**0.2219**
GT_A_84_P14323	**AT1G70680**	**-1.19**	**0.2221**
GT_A_84_P10041	**AT4G20840**	**-1.52**	**0.2225**
GT_AtCg00600_303	**petG**	**-0.99**	**0.2228**
GT_A_84_P13620	**AT3G21600**	**-0.89**	**0.2230**
GT_A_84_P787940	**PAP14**	**-3.27**	**0.2231**
GT_A_84_P21360	**AT1G69570**	**-1.83**	**0.2232**
GT_A_84_P585411	**AT1G60740**	**-1.29**	**0.2247**
GT_A_84_P809643	**TA28144_3702**	**-1.26**	**0.2250**
GT_A_84_P18353	**ATCAD4**	**-1.16**	**0.2252**
GT_A_84_P12152	**NF-YB6**	**-1.77**	**0.2253**
GT_A_84_P606261	**AT4G01023**	**-1.88**	**0.2255**
GT_A_84_P545008	**AT4G31875**	**-1.27**	**0.2258**
GT_A_84_P12303	**AT1G03810**	**-1.12**	**0.2274**
GT_A_84_P860230	**TIP2;2**	**-1.11**	**0.2276**

GT_A_84_P708752	TC368915	-0.81	0.2276
GT_A_84_P595141	JAZ8	-1.89	0.2290
GT_A_84_P572778	AT1G02040	-1.90	0.2291
GT_A_84_P21207	PHT2;1	-0.51	0.2295
GT_A_84_P751246	AT1G54750	-2.31	0.2300
GT_A_84_P724814	CB254609	-1.10	0.2301
GT_A_84_P769714	TC379145	-1.21	0.2303
GT_A_84_P19573	AT1G48100	-1.13	0.2312
GT_A_84_P113562	AT2G34460	-0.92	0.2312
GT_A_84_P807008	TA26725_3702	-1.08	0.2319
GT_A_84_P15006	TRY	-1.02	0.2320
GT_A_84_P15108	AT1G23205	-1.69	0.2324
GT_A_84_P140529	SPA3	-0.49	0.2324
GT_A_84_P537376	AT1G29420	-1.60	0.2327
GT_A_84_P147008	AT1G72070	-0.62	0.2332
GT_A_84_P790781	AT4G33310	-1.15	0.2335
GT_At_Specific_00247911	EIF3G2	-1.06	0.2336
GT_A_84_P753716	AT1G35255	-0.74	0.2338
GT_A_84_P126261	AT4G21445	-0.80	0.2342
GT_A_84_P822956	AT1G18060	-0.92	0.2342
GT_A_84_P300490	VTC2	-1.07	0.2345
GT_A_84_P132815	AT2G47790	-0.66	0.2347
GT_A_84_P12560	STH	-1.54	0.2354
GT_A_84_P599886	AT3G06145	-0.94	0.2358
GT_A_84_P204968	ATOCT2	-1.48	0.2365
GT_A_84_P13426	CYP96A15	-4.45	0.2370
GT_A_84_P20545	OHP	-1.04	0.2381
GT_A_84_P219958	AT5G42760	-2.79	0.2382
GT_A_84_P14472	pal1	-1.02	0.2383
GT_A_84_P192434	AT4G36850	-2.05	0.2387
GT_A_84_P10220	AT5G35970	-1.05	0.2390
GT_A_84_P11960	AT4G27220	-1.72	0.2392
GT_A_84_P805118	AT3G15450	-2.39	0.2394
GT_A_84_P847726	EPR1	-1.91	0.2396
GT_A_84_P16735	AT4G39070	-1.97	0.2397
GT_A_84_P861172	TC401014	-0.99	0.2407
GT_A_84_P20456	AT4G24180	-0.71	0.2407
GT_A_84_P762651	AT3G43432	-0.81	0.2409
GT_A_84_P847144		-0.80	0.2410
GT_A_84_P17431	AY536853	-1.33	0.2410
GT_A_84_P753215	AT1G45227;AT	-1.67	0.2411
GT_A_84_P292864	AT3G24460	-1.47	0.2411
GT_A_84_P296824	AT3G15450	-1.42	0.2412
GT_At_Specific_00193725	AtATG18a	-0.76	0.2413
GT_A_84_P11606	ATGSTU2	-1.00	0.2414
GT_A_84_P820062	TA35345_3702	-2.05	0.2414
GT_A_84_P850374	TC392079	-1.46	0.2425

GT_A_84_P56650	**AT5G41080**	**-2.09**	**0.2431**
GT_A_84_P263290	**AT4G25400**	**-2.32**	**0.2433**
GT_A_84_P800742	**ATCTH**	**-1.39**	**0.2437**
GT_A_84_P816248	**ATCAD4**	**-0.60**	**0.2437**
GT_A_84_P570699	**AT4G24730**	**-0.64**	**0.2444**
GT_A_84_P172281	**ATSDI1**	**-0.96**	**0.2448**
GT_A_84_P182054	**AT5G37690**	**-1.39**	**0.2450**
GT_A_84_P305070	**AT2G15020**	**-1.87**	**0.2457**
GT_A_84_P862707	**BP815481**	**-0.86**	**0.2459**
GT_A_84_P15448	**ATPP2-A3**	**-2.32**	**0.2464**
GT_A_84_P828509	**SUS4**	**-1.26**	**0.2472**
GT_A_84_P851101	**AT1G03730**	**-0.82**	**0.2477**
GT_A_84_P795728	**AK221753**	**-0.85**	**0.2478**
GT_A_84_P814996	**TA31293_3702**	**-0.87**	**0.2480**
GT_AtMg01210_1100	**orf101b**	**-0.72**	**0.2481**
GT_At_Specific_00238330	**AT4G36850.1**	**-2.18**	**0.2481**
GT_A_84_P22153	**ERD5**	**-1.50**	**0.2482**
GT_A_84_P597426	**HSA32**	**-1.76**	**0.2486**
GT_A_84_P832776	**BT3**	**-0.89**	**0.2499**
GT_A_84_P611961	**AT5G35120**	**-1.35**	**0.2501**
GT_At_Specific_00274990	**AT5G35320.1**	**-1.01**	**0.2506**
GT_A_84_P12665	**AT3G16560**	**-1.16**	**0.2515**
GT_At_Specific_00100325	**AT2G22088.1**	**-0.91**	**0.2523**
GT_At_Specific_00050632	**AT1G53541.1**	**-0.64**	**0.2524**
GT_A_84_P105666	**DA1**	**-0.76**	**0.2524**
GT_A_84_P12504	**UGT71C1**	**-0.90**	**0.2541**
GT_A_84_P752211	**AT1G76965**	**-1.38**	**0.2541**
GT_A_84_P734231	**TA30264_3702**	**-1.18**	**0.2543**
GT_A_84_P802894	**AtGLDP2**	**-1.62**	**0.2547**
GT_A_84_P828905	**BGLU15**	**-0.69**	**0.2550**
GT_A_84_P754151	**AT1G26761**	**-1.31**	**0.2553**
GT_A_84_P107712	**AT2G21100**	**-0.95**	**0.2556**
GT_A_84_P811601	**TA29106_3702**	**-2.33**	**0.2562**
GT_A_84_P814110	**CaS**	**-1.15**	**0.2568**
GT_At_Specific_00045391	**AT1G48890.1**	**-1.22**	**0.2571**
GT_A_84_P857548	**AT2G25970**	**-0.51**	**0.2576**
GT_A_84_P847162	**ATTPS03**	**-1.81**	**0.2580**
GT_A_84_P15050	**AT5G64100**	**-2.46**	**0.2581**
GT_A_84_P10456	**NAS3**	**-0.74**	**0.2585**
GT_A_84_P204928	**AT4G01050**	**-0.59**	**0.2588**
GT_A_84_P855180	**AT4G28300**	**-0.83**	**0.2599**
GT_A_84_P10206	**ZFP3**	**-1.26**	**0.2606**
GT_A_84_P10253	**ERD2**	**-0.62**	**0.2607**
GT_A_84_P17199	**AT1G15440**	**-0.98**	**0.2611**
GT_A_84_P15932	**TIP2;3**	**-1.33**	**0.2617**
GT_A_84_P15956	**LHCB3**	**-0.87**	**0.2621**
GT_A_84_P19901	**AT1G30860**	**-1.07**	**0.2625**

GT_A_84_P831762	**AT1G15290**	**-0.66**	**0.2627**
GT_A_84_P850648	**AT5G58370**	**-1.10**	**0.2628**
GT_A_84_P756272	**NP10426526**	**-3.11**	**0.2629**
GT_A_84_P23764	**AtTLP1**	**-0.59**	**0.2632**
GT_A_84_P22494	**AT5G23730**	**-1.18**	**0.2633**
GT_A_84_P819096	**TA34519_3702**	**-4.11**	**0.2634**
GT_A_84_P87549	**HYH**	**-1.13**	**0.2642**
GT_A_84_P152528	**CaS**	**-1.22**	**0.2643**
GT_A_84_P12696	**AT1G26790**	**-2.86**	**0.2647**
GT_A_84_P522030	**AT5G14110**	**-0.90**	**0.2647**
GT_At_Specific_00044462	**AT1G48100.1**	**-1.10**	**0.2648**
GT_A_84_P828124	**AT1G72360**	**-0.85**	**0.2661**
GT_A_84_P786032	**VTC2**	**-1.11**	**0.2670**
GT_A_84_P845932	**AT1G74470**	**-0.69**	**0.2670**
GT_A_84_P787428	**AT4G27654**	**-1.03**	**0.2677**
GT_A_84_P760282	**U2.3**	**-0.61**	**0.2678**
GT_A_84_P12285	**AT1G44000**	**-0.76**	**0.2679**
GT_AtMg01220_1103	**orf113**	**-0.55**	**0.2682**
GT_A_84_P558374	**TC365940**	**-1.30**	**0.2682**
GT_A_84_P812380	**ERD5**	**-1.59**	**0.2683**
GT_A_84_P789439	**AT3G24460**	**-0.60**	**0.2685**
GT_A_84_P18726	**WNK9**	**-2.30**	**0.2694**
GT_A_84_P803506	**ERD5**	**-1.67**	**0.2698**
GT_A_84_P16751	**TIP2;2**	**-1.51**	**0.2703**
GT_A_84_P805135	**AT3G15450**	**-2.51**	**0.2703**
GT_A_84_P20199	**DGD1**	**-0.98**	**0.2707**
GT_A_84_P218538	**AT4G24800**	**-0.90**	**0.2710**
GT_A_84_P73804	**AGL42**	**-1.33**	**0.2713**
GT_A_84_P15364	**ATCYSA**	**-0.58**	**0.2717**
GT_A_84_P558862	**RTFL8**	**-1.06**	**0.2731**
GT_A_84_P566577	**AT1G16730**	**-1.06**	**0.2739**
GT_A_84_P823929	**AT1G03850**	**-1.11**	**0.2748**
GT_A_84_P10439	**BIP3**	**-1.35**	**0.2749**
GT_A_84_P534840	**AT1G80245**	**-0.66**	**0.2751**
GT_A_84_P17160	**ATTAP1**	**-0.62**	**0.2756**
GT_A_84_P15752	**AT4G29690**	**-1.39**	**0.2763**
GT_A_84_P19248	**PPT2**	**-2.24**	**0.2768**
GT_A_84_P843621	**AT2G31010**	**-0.75**	**0.2773**
GT_A_84_P14468	**CKX5**	**-1.36**	**0.2779**
GT_A_84_P140739	**AT5G51780**	**-1.61**	**0.2779**
GT_A_84_P16137	**AT1G10530**	**-0.96**	**0.2795**
GT_A_84_P818067	**TA33649_3702**	**-1.18**	**0.2800**
GT_A_84_P231249	**AT5G08415**	**-0.53**	**0.2804**
GT_A_84_P834383	**SDG37**	**-0.71**	**0.2822**
GT_A_84_P800627	**AT2G02700**	**-2.31**	**0.2824**
GT_A_84_P13806	**AT4G12890**	**-1.27**	**0.2827**
GT_A_84_P846067	**CaS**	**-1.11**	**0.2844**

GT_A_84_P135105	**AT3G17640**	**-1.25**	**0.2846**
GT_A_84_P220658	**AT2G36930**	**-0.64**	**0.2854**
GT_A_84_P263540	**AT3G06660**	**-0.61**	**0.2860**
GT_A_84_P17595	**NCED2**	**-0.91**	**0.2865**
GT_A_84_P79395	**AT1G71240**	**-0.71**	**0.2880**
GT_A_84_P760545	**AT3G06433**	**-1.00**	**0.2881**
GT_A_84_P563929	**emb2170**	**-0.48**	**0.2883**
GT_AtMg00630_839	**orf110b**	**-0.70**	**0.2891**
GT_A_84_P825811	**ATTPS11**	**-1.17**	**0.2898**
GT_A_84_P11805	**AT3G47420**	**-1.60**	**0.2909**
GT_A_84_P17043	**FH4**	**-0.97**	**0.2920**
GT_A_84_P831886	**AT3G07770**	**-0.60**	**0.2922**
GT_A_84_P103806	**AT5G21940**	**-1.06**	**0.2922**
GT_A_84_P794434	**AT4G27460**	**-1.11**	**0.2923**
GT_A_84_P17400	**RSH2**	**-0.64**	**0.2929**
GT_A_84_P157005	**AT4G25290**	**-1.21**	**0.2940**
GT_A_84_P790164	**AT5G41080**	**-2.10**	**0.2942**
GT_A_84_P17379	**APX2**	**-2.03**	**0.2948**
GT_A_84_P17470	**AT3G46070**	**-2.27**	**0.2948**
GT_A_84_P19365	**AT3G46600**	**-0.76**	**0.2950**
GT_A_84_P110522	**AT3G06080**	**-1.00**	**0.2951**
GT_A_84_P21501	**DREB2A**	**-1.77**	**0.2961**
GT_A_84_P191914	**AT2G21320**	**-1.67**	**0.2980**
GT_A_84_P826876	**FLA15**	**-0.62**	**0.2980**
GT_At_Specific_00309400	**AT5G64501.1**	**-0.53**	**0.2983**
GT_A_84_P715335	**TA31585_3702**	**-1.52**	**0.2995**
GT_A_84_P21458	**BT5**	**-2.56**	**0.3020**
GT_A_84_P20875	**SIGA**	**-0.78**	**0.3023**
GT_A_84_P823870	**HYH**	**-1.32**	**0.3028**
GT_A_84_P797786	**AT4G08874**	**-0.77**	**0.3028**
GT_A_84_P842548	**AT1G07700**	**-0.71**	**0.3038**
GT_A_84_P806987	**LHCB3**	**-0.89**	**0.3052**
GT_A_84_P856326	**TC402207**	**-0.96**	**0.3058**
GT_A_84_P24117	**CAM9**	**-1.01**	**0.3072**
GT_A_84_P14914	**AT5G17230**	**-0.94**	**0.3084**
GT_A_84_P806550	**CAB3**	**-0.63**	**0.3087**
GT_A_84_P833445	**AT1G26790**	**-2.88**	**0.3091**
GT_A_84_P19960	**NCED9**	**-1.53**	**0.3097**
GT_A_84_P20844	**AT1G09480**	**-1.27**	**0.3099**
GT_A_84_P853447	**AILP1**	**-0.86**	**0.3099**
GT_A_84_P20475	**AT4G28200**	**-0.77**	**0.3101**
GT_A_84_P728206	**DR368472**	**-4.33**	**0.3106**
GT_A_84_P814104	**CaS**	**-1.11**	**0.3111**
GT_A_84_P137739	**EDL3**	**-0.99**	**0.3117**
GT_A_84_P23929	**ATTPS11**	**-1.25**	**0.3119**
GT_A_84_P819955	**CTF2A**	**-0.96**	**0.3122**
GT_A_84_P817459	**AT5G12470**	**-0.80**	**0.3126**

GT_A_84_P802975	**TIP2;2**	**-1.63**	**0.3128**
GT_A_84_P818968	**AT3G15630**	**-1.68**	**0.3144**
GT_A_84_P13963	**TT4**	**-1.57**	**0.3154**
GT_A_84_P787920	**FAR5**	**-1.69**	**0.3167**
GT_A_84_P590665	**AT1G22220**	**-1.32**	**0.3168**
GT_A_84_P115992	**CIPK1**	**-0.99**	**0.3170**
GT_A_84_P23737	**Hsp70b**	**-2.30**	**0.3172**
GT_A_84_P501308	**AT3G14260**	**-1.01**	**0.3180**
GT_A_84_P830062	**AT2G21100**	**-0.71**	**0.3183**
GT_At_Specific_00091184	**AT2G14206.1**	**-1.65**	**0.3183**
GT_A_84_P19824	**AT2G17880**	**-1.14**	**0.3184**
GT_A_84_P12642	**AT3G10020**	**-1.55**	**0.3185**
GT_A_84_P20298	**SUS4**	**-1.33**	**0.3197**
GT_A_84_P123362	**AFP2**	**-0.65**	**0.3204**
GT_A_84_P126241	**AT5G41900**	**-0.62**	**0.3212**
GT_A_84_P219198	**MLP329**	**-1.58**	**0.3219**
GT_A_84_P803184	**AT3G10020**	**-1.55**	**0.3222**
GT_A_84_P14072	**ATEXPA14**	**-0.93**	**0.3227**
GT_A_84_P76674	**ESK1**	**-0.52**	**0.3245**
GT_A_84_P15873	**AT5G23900**	**-0.44**	**0.3247**
GT_A_84_P17031	**AT1G03850**	**-0.96**	**0.3249**
GT_A_84_P23072	**AT1G26500**	**-0.58**	**0.3266**
GT_A_84_P10925	**AT3G62950**	**-2.76**	**0.3300**
GT_A_84_P18299	**AtSS2**	**-0.64**	**0.3304**
GT_A_84_P708446	**AK229798**	**-0.62**	**0.3307**
GT_A_84_P839794	**NP173972**	**-1.92**	**0.3309**
GT_A_84_P20121	**AT1G07860**	**-0.94**	**0.3313**
GT_A_84_P15333	**AT2G19740**	**-0.57**	**0.3336**
GT_At_Specific_00138797	**AT3G06660.1**	**-0.81**	**0.3339**
GT_A_84_P24090	**AT3G45710**	**-0.93**	**0.3340**
GT_A_84_P816904	**TIP2;3**	**-1.14**	**0.3351**
GT_A_84_P558224	**AT4G15990**	**-1.49**	**0.3359**
GT_A_84_P607832	**AT2G41150**	**-0.98**	**0.3370**
GT_A_84_P810735	**AOAT2**	**-0.94**	**0.3386**
GT_A_84_P17170	**WRKY66**	**-1.14**	**0.3407**
GT_A_84_P762845	**AT3G13061**	**-1.69**	**0.3409**
GT_A_84_P224099	**EPR1**	**-1.94**	**0.3409**
GT_A_84_P116392	**AT2G15910**	**-1.31**	**0.3418**
GT_A_84_P260410	**AT1G78450**	**-1.76**	**0.3427**
GT_A_84_P858464	**AT3G10020**	**-1.34**	**0.3467**
GT_A_84_P10193	**AT5G18600**	**-0.87**	**0.3486**
GT_A_84_P18932	**AST91**	**-0.54**	**0.3499**
GT_A_84_P703109	**TA29342_3702**	**-0.89**	**0.3499**
GT_A_84_P844879	**AT3G06080**	**-1.07**	**0.3517**
GT_A_84_P753607	**AT1G56165**	**-1.25**	**0.3518**
GT_A_84_P862409	**TA27098_3702**	**-1.38**	**0.3525**
GT_A_84_P740321	**FAR5**	**-1.82**	**0.3530**

GT_A_84_P20750	**AT5G19890**	**-1.67**	**0.3533**
GT_A_84_P550637	**AT1G18830**	**-1.81**	**0.3536**
GT_A_84_P16802	**AT5G14470**	**-2.24**	**0.3540**
GT_A_84_P858508	**AT1G10950**	**-0.46**	**0.3566**
GT_A_84_P23924	**AT2G18150**	**-0.96**	**0.3592**
GT_A_84_P764462	**RMA1**	**-1.05**	**0.3607**
GT_A_84_P23171	**HSFA7A**	**-1.81**	**0.3617**
GT_A_84_P789498	**PPCK1**	**-0.99**	**0.3619**
GT_A_84_P753767	**AT1G04945**	**-0.77**	**0.3620**
GT_A_84_P21759	**AT1G55530**	**-0.64**	**0.3639**
GT_A_84_P11328	**AT1G20470**	**-1.57**	**0.3645**
GT_At_Specific_00062660	**AT1G65346.1**	**-0.95**	**0.3645**
GT_A_84_P15515	**AT3G19390**	**-0.79**	**0.3651**
GT_A_84_P845356	**RMA1**	**-0.96**	**0.3658**
GT_A_84_P737484	**ATBAG5**	**-1.47**	**0.3659**
GT_A_84_P23366	**AT4G15400**	**-1.45**	**0.3670**
GT_A_84_P263200	**AT5G43870**	**-0.97**	**0.3678**
GT_A_84_P801001	**TIP2;2**	**-1.74**	**0.3681**
GT_A_84_P766880	**AT5G13770**	**-0.93**	**0.3682**
GT_A_84_P819186	**F2KP**	**-1.04**	**0.3692**
GT_At_Specific_00235280	**UGT73B1**	**-0.70**	**0.3696**
GT_A_84_P759606	**DGD1**	**-0.84**	**0.3699**
GT_A_84_P279060	**AT3G12320**	**-1.75**	**0.3702**
GT_A_84_P17576	**AT4G09760**	**-0.94**	**0.3730**
GT_AntiSense_AtCg01120_516	**rps15**	**-0.92**	**0.3735**
GT_A_84_P830520	**AT2G44010**	**-0.54**	**0.3769**
GT_At_Specific_00073965	**JAC1**	**-1.78**	**0.3782**
GT_A_84_P18001	**AT1G65500**	**-1.56**	**0.3795**
GT_A_84_P21316	**EDA14**	**-0.63**	**0.3797**
GT_A_84_P148978	**AT2G39560**	**-0.81**	**0.3798**
GT_At_Specific_00021795	**AT1G19540.1**	**-0.87**	**0.3812**
GT_A_84_P813553	**AT5G21940**	**-0.90**	**0.3813**
GT_A_84_P51580	**AT1G72060**	**-0.82**	**0.3825**
GT_A_84_P786422	**EDL3**	**-0.83**	**0.3830**
GT_A_84_P186644	**AT2G37240**	**-1.02**	**0.3839**
GT_A_84_P14951	**AT5G38020**	**-1.23**	**0.3843**
GT_A_84_P23668	**ACHT4**	**-0.78**	**0.3848**
GT_A_84_P17452	**AT3G14780**	**-0.77**	**0.3851**
GT_At_Specific_00151698	**AT3G17640.1**	**-1.17**	**0.3851**
GT_A_84_P785981	**ACLA-3**	**-0.69**	**0.3863**
GT_A_84_P10638	**STP1**	**-1.60**	**0.3907**
GT_A_84_P17310	**AT2G30830**	**-1.15**	**0.3911**
GT_A_84_P843020	**AT1G07010**	**-1.05**	**0.3923**
GT_A_84_P837084	**AT1G58280**	**-1.08**	**0.3936**
GT_A_84_P806475	**CAB1**	**-0.58**	**0.3943**
GT_A_84_P13975	**BUD2**	**-0.79**	**0.3952**
GT_A_84_P13916	**AT4G36430**	**-1.35**	**0.3967**

GT_A_84_P22113	VTC4	-0.84	0.3969
GT_A_84_P768849	PEP7	-1.11	0.3976
GT_A_84_P16943	TSA1	-0.53	0.3987
GT_A_84_P91449	AT5G63060	-0.66	0.3995
GT_A_84_P843906	AT1G72060	-0.84	0.4002
GT_A_84_P17398	UVR3	-0.88	0.4008
GT_A_84_P19241	CCA1	-1.83	0.4017
GT_A_84_P603421	AT3G01960	-0.81	0.4059
GT_A_84_P14674	AT1G23880	-0.48	0.4059
GT_A_84_P21292	GR	-0.55	0.4096
GT_A_84_P16933	AT1G55290	-1.64	0.4102
GT_A_84_P13322	AT1G21680	-0.67	0.4102
GT_A_84_P768857	AT5G44572	-0.64	0.4107
GT_A_84_P19318	AT3G22060	-1.15	0.4149
GT_A_84_P17279	CIPK16	-1.21	0.4157
GT_A_84_P19062	PSAH2	-0.75	0.4166
GT_A_84_P232159	ATCSLB03	-1.01	0.4176
GT_A_84_P20292	COL2	-2.48	0.4188
GT_A_84_P19638	ROXY2	-1.82	0.4199
GT_A_84_P806566	TA26506_3702	-0.52	0.4207
GT_A_84_P13141	AT5G59770	-0.65	0.4231
GT_A_84_P87029	ECT1	-0.53	0.4253
GT_A_84_P166653	AT1G07010	-1.13	0.4260
GT_A_84_P795995	TIP2	-0.92	0.4261
GT_A_84_P15222	4CL3	-1.79	0.4287
GT_A_84_P14603	COBL2	-1.18	0.4288
GT_A_84_P852616	SNG1	-0.96	0.4292
GT_A_84_P16894	AT5G51910	-0.48	0.4296
GT_A_84_P113602	MEE12	-0.64	0.4298
GT_A_84_P21494	PRR7	-0.91	0.4314
GT_A_84_P133915	AT2G42220	-0.53	0.4341
GT_A_84_P577849	AT4G33770	-0.75	0.4344
GT_A_84_P198204	AT2G17036	-0.86	0.4352
GT_A_84_P15092	AT1G35560	-0.89	0.4362
GT_A_84_P15329	HSPRO2	-1.07	0.4384
GT_A_84_P823347	AT4G37180	-0.71	0.4403
GT_A_84_P18146	AT1G71730	-0.47	0.4421
GT_A_84_P797027	AT2G22460	-0.96	0.4478
GT_A_84_P230699	AT5G05530	-0.73	0.4508
GT_A_84_P516750	AT5G26620	-0.89	0.4515
GT_A_84_P808612	TIP2	-1.03	0.4532
GT_At_Specific_00258069	AT5G15700.1	-0.74	0.4548
GT_A_84_P861784	BP594753	-1.03	0.4552
GT_A_84_P157425	AT5G12470	-0.94	0.4566
GT_A_84_P17787	AT5G37670	-1.64	0.4592
GT_A_84_P761568	AT3G52060	-1.74	0.4609
GT_A_84_P21802	AT1G78780	-0.58	0.4612

GT_A_84_P16682	**AT4G26190**	**-0.52**	**0.4614**
GT_A_84_P18387	**AT3G24070**	**-0.62**	**0.4615**
GT_A_84_P541636	**AT5G56850**	**-0.72**	**0.4635**
GT_A_84_P806597	**CAB1**	**-0.64**	**0.4639**
GT_A_84_P586369	**PSF1**	**-0.60**	**0.4646**
GT_A_84_P806562	**TA26504_3702**	**-0.50**	**0.4661**
GT_A_84_P20910	**AT1G72360**	**-0.86**	**0.4681**
GT_At_Specific_00062937	**AT1G65500.1**	**-1.10**	**0.4688**
GT_A_84_P22919	**scpl46**	**-1.26**	**0.4701**
GT_A_84_P307290	**AT1G54740**	**-1.58**	**0.4729**
GT_A_84_P222439	**FLA15**	**-0.74**	**0.4758**
GT_At_Specific_00052096	**AT1G54740.1**	**-1.61**	**0.4765**
GT_A_84_P795530	**AT5G22390**	**-1.59**	**0.4765**
GT_A_84_P856698	**AI993110**	**-0.61**	**0.4785**
GT_A_84_P812709	**TA29745_3702**	**-1.12**	**0.4811**
GT_A_84_P851531	**ACBP3**	**-1.04**	**0.4818**
GT_A_84_P847991	**CCA1**	**-1.81**	**0.4821**
GT_A_84_P837561	**AT5G06790**	**-1.04**	**0.4827**
GT_A_84_P869124	**KING1**	**-0.61**	**0.4832**
GT_A_84_P23294	**ACBP3**	**-1.09**	**0.4833**
GT_A_84_P12395	**AT1G32900**	**-2.34**	**0.4848**
GT_A_84_P132245	**AT5G50940**	**-0.71**	**0.4853**
GT_A_84_P558547	**AT5G65850**	**-0.69**	**0.4855**
GT_At_Specific_00073790	**AT1G74929.1**	**-1.08**	**0.4870**
GT_A_84_P23916	**AT2G19385**	**-0.68**	**0.4923**
GT_AntiSense_AtMg00990_995	**nad3**	**-0.80**	**0.4925**
GT_A_84_P761492	**AT3G30385**	**-0.94**	**0.4938**
GT_A_84_P797979	**EG478422**	**-0.52**	**0.4958**
GT_A_84_P813506	**APT3**	**-0.73**	**0.4973**
GT_A_84_P858735	**VTC4**	**-0.90**	**0.4994**
GT_A_84_P566307	**AT5G06790**	**-0.92**	**0.4996**
GT_A_84_P16389	**AT2G34590**	**-0.81**	**0.5003**
GT_A_84_P23284	**AT4G21680**	**-0.86**	**0.5004**
GT_A_84_P146119	**AT1G78930**	**-0.73**	**0.5051**
GT_A_84_P12884	**APT3**	**-0.91**	**0.5100**
GT_A_84_P10158	**WCRKC1**	**-0.74**	**0.5116**
GT_A_84_P23248	**AT4G08850**	**-1.08**	**0.5128**
GT_A_84_P13893	**AGL21**	**-1.75**	**0.5135**
GT_A_84_P815126	**TA31395_3702**	**-1.57**	**0.5136**
GT_A_84_P18692	**CARAB-AK-LYS**	**-0.67**	**0.5164**
GT_A_84_P289354	**MEE14**	**-0.96**	**0.5174**
GT_A_84_P140929	**AT3G58470**	**-0.60**	**0.5174**
GT_A_84_P816899	**AT2G34750**	**-0.78**	**0.5187**
GT_A_84_P238393	**AT4G22900**	**-0.74**	**0.5191**
GT_A_84_P17510	**ATTPS9**	**-0.85**	**0.5192**
GT_A_84_P11700	**UGT72B3**	**-0.68**	**0.5233**
GT_A_84_P858662	**AV521199**	**-0.69**	**0.5235**

GT_A_84_P10787	**CYP72A14**	**-1.09**	**0.5250**
GT_A_84_P519046	**AT3G09450**	**-0.62**	**0.5285**
GT_A_84_P557599	**AT5G57640**	**-1.22**	**0.5286**
GT_A_84_P155535	**AT5G56100**	**-1.55**	**0.5314**
GT_A_84_P10432	**2-Apr**	**-1.21**	**0.5321**
GT_A_84_P806534	**CAB2**	**-0.96**	**0.5417**
GT_A_84_P807217	**AT3G32980**	**-0.76**	**0.5440**
GT_A_84_P192414	**AT5G51790**	**-1.24**	**0.5444**
GT_A_84_P63350	**AT1G29720**	**-1.12**	**0.5449**
GT_A_84_P521345	**BT3**	**-0.56**	**0.5453**
GT_A_84_P868161	**AT1G14200**	**-1.82**	**0.5457**
GT_A_84_P826185	**TA42103_3702**	**-1.09**	**0.5467**
GT_A_84_P209738	**AT5G55530**	**-0.73**	**0.5509**
GT_A_84_P21137	**MTO1**	**-0.43**	**0.5518**
GT_A_84_P847314	**CCA1**	**-1.66**	**0.5527**
GT_A_84_P23721	**AT1G14200**	**-1.95**	**0.5564**
GT_A_84_P833384	**DOG1**	**-1.35**	**0.5588**
GT_A_84_P830293	**TA47612_3702**	**-1.27**	**0.5605**
GT_A_84_P808697	**LHCB2.1**	**-2.36**	**0.5632**
GT_A_84_P715787	**AT1G32928**	**-0.83**	**0.5633**
GT_A_84_P12911	**anac074**	**-0.86**	**0.5636**
GT_A_84_P824370	**AT5G59080**	**-0.80**	**0.5641**
GT_At_Specific_00026728	**ATTPS9**	**-0.69**	**0.5655**
GT_A_84_P14336	**THI2.1**	**-0.95**	**0.5686**
GT_A_84_P78069	**AT2G35820**	**-0.57**	**0.5688**
GT_A_84_P20655	**DOG1**	**-1.36**	**0.5711**
GT_A_84_P169033	**TPX2**	**-1.42**	**0.5737**
GT_A_84_P16925	**AT5G60280**	**-0.57**	**0.5849**
GT_A_84_P20714	**ACHT5**	**-1.21**	**0.5861**
GT_A_84_P815236	**ACHT4**	**-0.67**	**0.5865**
GT_A_84_P824725	**AT5G55530**	**-0.59**	**0.5880**
GT_A_84_P753739	**4CL1**	**-0.67**	**0.5946**
GT_A_84_P14108	**ATATH13**	**-1.05**	**0.5966**
GT_A_84_P600850	**AT3G28540**	**-0.65**	**0.5971**
GT_A_84_P808620	**TIP2**	**-1.15**	**0.5973**
GT_A_84_P505812	**AT3G44120**	**-1.25**	**0.6025**
GT_A_84_P153955	**AT3G22680**	**-0.72**	**0.6049**
GT_A_84_P108832	**AT3G06070**	**-0.77**	**0.6092**
GT_A_84_P825243	**AT2G38820**	**-0.62**	**0.6093**
GT_A_84_P754199	**AT1G67328**	**-1.28**	**0.6095**
GT_A_84_P839669	**AT1G08300**	**-0.74**	**0.6106**
GT_A_84_P861951	**MTO1**	**-0.90**	**0.6148**
GT_A_84_P814618	**AT5G22920**	**-1.75**	**0.6153**
GT_A_84_P265370	**EMB975**	**-0.71**	**0.6157**
GT_A_84_P16440	**AT3G04140**	**-1.01**	**0.6169**
GT_At_Specific_00231736	**AT4G31248.1**	**-0.78**	**0.6181**
GT_A_84_P221276	**AT5G22390**	**-1.07**	**0.6186**

GT_A_84_P258170	AT5G53700	-0.84	0.6203
GT_A_84_P813469	MEE14	-1.07	0.6271
GT_A_84_P13533	AT1G67790	-0.63	0.6295
GT_A_84_P786772	AK228670	-1.13	0.6297
GT_A_84_P22702	AT1G51380	-0.93	0.6323
GT_A_84_P21744	PIP1C	-0.59	0.6327
GT_A_84_P21530	AT5G14120	-0.74	0.6385
GT_A_84_P21831	AT1G15380	-0.88	0.6405
GT_A_84_P830143	AT4G06744	-0.74	0.6411
GT_A_84_P785035	AT2G34750	-0.63	0.6464
GT_A_84_P822947	scpl46	-0.98	0.6477
GT_A_84_P532027	GLTP2	-1.19	0.6485
GT_A_84_P21943	XTR4	-1.01	0.6514
GT_A_84_P757994	ATRL4	-1.18	0.6520
GT_A_84_P130356	AT5G20790	-2.13	0.6553
GT_A_84_P14746	AT-HSFB2B	-1.07	0.6558
GT_A_84_P10198	AT5G22920	-1.59	0.6561
GT_A_84_P810552	BT2	-1.23	0.6562
GT_A_84_P519104	RTFL16	-0.76	0.6601
GT_A_84_P11987	AT4G33420	-0.91	0.6622
GT_A_84_P592063	AT4G14746	-0.63	0.6653
GT_A_84_P829614	TA46524_3702	-0.51	0.6663
GT_A_84_P838052	AXR3	-1.06	0.6703
GT_A_84_P15219	NLA	-0.67	0.6717
GT_A_84_P304680	AT2G30600	-0.95	0.6747
GT_A_84_P760178	AT3G28540	-0.67	0.6754
GT_A_84_P761945	AT3G61028	-0.81	0.6769
GT_A_84_P855484	TC396686	-1.44	0.6819
GT_A_84_P20866	AXR3	-0.93	0.6829
GT_A_84_P13976	AT5G19120	-1.37	0.6872
GT_A_84_P813465	MEE14	-0.96	0.6905
GT_A_84_P235073	AT1G73870	-2.39	0.6925
GT_A_84_P10711	JAL23	-0.52	0.6951
GT_A_84_P15807	ATMPK14	-1.19	0.6967
GT_A_84_P21264	BT2	-1.36	0.7071
GT_A_84_P816855	AT3G07350	-1.01	0.7103
GT_A_84_P522895	AT5G10210	-0.94	0.7170
GT_A_84_P18225	TRI	-0.71	0.7175
GT_A_84_P805324	PIP1C	-0.61	0.7200
GT_A_84_P18414	AT1G02610	-0.95	0.7215
GT_A_84_P850547	TA29936_3702	-0.74	0.7231
GT_A_84_P13656	DIN4	-0.56	0.7291
GT_A_84_P16882	AT5G48540	-0.85	0.7335
GT_A_84_P851088	AT4G38960	-0.85	0.7369
GT_At_Specific_00071252	AT1G72645.1	-0.69	0.7404
GT_A_84_P10434	AT1G70250	-0.55	0.7421
GT_A_84_P87509	ATMRP15	-0.93	0.7463

GT_A_84_P768671	AT5G42567	-0.67	0.7489
GT_A_84_P811729	AT2G33830	-1.22	0.7515
GT_A_84_P825709	AT4G13030	-0.73	0.7564
GT_A_84_P292244	AT3G53690	-0.48	0.7590
GT_A_84_P812267	UGE1	-0.66	0.7595
GT_A_84_P12008	AT4G38960	-0.90	0.7687
GT_A_84_P24070	CLC-B	-1.02	0.7691
GT_A_84_P55070	AT4G13030	-0.51	0.7709
GT_A_84_P94769	FLA16	-0.54	0.7710
GT_A_84_P849657	AT5G19120	-1.45	0.7747
GT_A_84_P16129	ATTPS10	-0.68	0.7748
GT_A_84_P14811	AT4G30680	-0.66	0.7768
GT_A_84_P856422	UGE1	-0.60	0.7787
GT_A_84_P506004	AT5G01790	-0.67	0.7846
GT_A_84_P812266	UGE1	-0.68	0.7875
GT_A_84_P22210	AT3G48390	-1.60	0.7941
GT_A_84_P857510	AT3G11930	-0.53	0.7960
GT_A_84_P851517	BGAL4	-0.94	0.8006
GT_A_84_P208048	AT5G44580	-0.58	0.8056
GT_A_84_P847356	TC387646	-0.80	0.8105
GT_A_84_P827439	CARAB-AK-LYS	-0.59	0.8242
GT_A_84_P18656	AT5G02780	-1.02	0.8277
GT_A_84_P582090	AT3G18773	-0.89	0.8296
GT_A_84_P12570	AT2G28120	-0.98	0.8321
GT_A_84_P16823	LHY	-1.54	0.8359
GT_A_84_P13934	AT5G05270	-1.01	0.8385
GT_A_84_P22366	AT4G28020	-0.62	0.8396
GT_A_84_P83349	AT1G26800	-0.71	0.8525
GT_A_84_P11063	AT4G38950	-0.85	0.8535
GT_A_84_P14131	DIN10	-1.34	0.8535
GT_A_84_P869207	AT1G23390	-0.77	0.8623
GT_A_84_P20669	CPuORF4	-1.29	0.8629
GT_A_84_P852163	DIN10	-1.05	0.8670
GT_A_84_P768392	NF-YC4	-0.57	0.8696
GT_A_84_P16890	AT5G50800	-0.84	0.8756
GT_At_Specific_00175701	ASN1	-1.84	0.8813
GT_A_84_P761404	AT3G21460	-0.99	0.8815
GT_A_84_P549683	AT3G18320	-1.31	0.8856
GT_A_84_P758588	AT2G30766	-1.43	0.8985
GT_A_84_P609548	AT2G25200	-0.67	0.8989
GT_A_84_P808273	TA27460_3702	-0.48	0.9004
GT_A_84_P786694	AT1G23390	-0.66	0.9009
GT_A_84_P758054	AT2G06002	-1.31	0.9166
GT_A_84_P189814	AT3G52670	-0.59	0.9214
GT_A_84_P10964	AT4G11190	-1.16	0.9235
GT_A_84_P841311	AT3G21460	-0.79	0.9260
GT_A_84_P811969	AT5G57655	-0.78	0.9418

GT_A_84_P19386	**AT3G51540**	**-0.80**	**0.9482**
GT_A_84_P793199	**ATPT2**	**-0.65**	**0.9564**
GT_A_84_P14249	**AT1G36060**	**-0.50**	**0.8467**
GT_A_84_P19543	**AT4G31240**	**-0.65**	**0.9601**
GT_A_84_P857022	**AT1G80440**	**-0.90**	**0.9735**
GT_A_84_P198764<br>GT_A_84_P788231	**AT3G49790**<br>**ATGSTF14**	**-0.71**<br>**-0.57**	**0.9852**<br>**0.0329**
GT_A_84_P800547	**ATGSTF10**	**-1.00**	**0.260**

Supplementary Table S3. Genes commonly found down-regulated in all the three mutants in response to combined stress treatment .

**Genes**	**Gene Name**
AT3G09640	*Ascorbate peroxidase*2
AT3G02870	*Inositol monophosphatase*
AT3G24500	*Multiprotein bridging factor* 1C
AT5G45820	*CBL-interacting protein kinase* 20
AT1G01060	*Late elongated hypocotyl* (*LHY*)
AT2G46830	circadian clock associated 1
AT1G53540	*HSP*20
AT2G32120	*Heat-shock protein* 70T-2
AT3G03400	*EF hand calcium-binding protein family*
AT2G29500	*HSP20-like chaperones superfamily protein*
ATCG00920	*Chloroplast-encoded 16S ribosomal RNA*
AT5G01320	*Pyruvate decarboxylase, putative*
AT4G09150	*T-complex protein 11*
AT5G62920	*Response regulator*6
AT5G12020	*17.6 kDa class II heat shock protein*
AT3G46230	*Heat shock protein* 17.4
AT1G78450	*SOUL heme-binding family protein*
AT3G63350	*Winged-helix DNA-binding transcription factor family protein*
AT2G25140	*Casein lytic proteinase* B4
AT2G47180	*Galactinol synthase* 1
AT1G07350	*RNA-binding (RRM/RBD/RNP motifs) family protein*
AT2G04395	AT2G04395.1
AT2G32550	*Cell differentiation, Rcd1-like protein*
AT1G74890	*Response regulator* 15
AT5G48570	*FKBP-type peptidyl-prolyl cis-trans isomerase family protein*
AT1G63580	*Receptor-like protein kinase-related family protein*
AT2G34910	*Root hair specific* 4
AT2G26820	*Phloem protein* 2-A3
AT3G51910	*Heat shock transcription factor* A7A

AT5G23035	*Putative membrane lipoprotein*
AT4G21320	*Aldolase-type TIM barrel family protein*
AT5G64510	Unknown protein
AT4G33070	*Thiamine pyrophosphate dependent pyruvate decarboxylase family protein*
AT1G18830	*Transducin/WD40 repeat-like superfamily protein*
AT1G30070	*SGS domain-containing protein*
AT5G12110	*Glutathione S-transferase, C-terminal-like;Translation elongation factor EF1B/ribosomal protein S6*
AT2G24210	*Terpene synthase 10*
AT5G59780	*Myb domain protein* 59
AT4G10250	*HSP20-like chaperones superfamily protein*
AT1G57750	*Cytochrome P450, family 96, subfamily A, polypeptide 15*
AT5G41080	*PLC-like phosphodiesterases superfamily protein*
AT3G29810	*COBRA-like protein 2 precursor*
AT4G23493	*Unknown protein*
AT1G54050	*HSP20-like chaperones superfamily protein*
AT3G52620	*Unknown protein*
AT5G19100	*Eukaryotic aspartyl protease family protein*
AT5G09590	Heat shock protein 70 (Hsp 70) family protein
AT1G22220	*F-box family protein*
AT3G53830	*Regulator of chromosome condensation (RCC1) family protein*
AT5G25390	*SHN2, Integrase-type DNA-binding superfamily protein*
AT5G12030	*Heat shock protein 17.6A*
AT2G20560	*DNAJ heat shock family protein*
AT3G43190	*Sucrose synthase 4*
AT2G03720	MRH6 (morphogenesis of root hair 6)
AT1G35255	*Unknown protein*
AT5G52640	*Heat shock protein*90.1
AT5G64100	*Peroxidase superfamily protein*
AT3G25230	*Rotamase FKBP 1*
AT1G61430	*S-locus lectin protein kinase family protein*
AT1G74310	*Heat shock protein 101*
AT4G12400	*Stress-inducible protein*
AT2G46090	*Diacylglycerol kinase family protein*
AT4G25200	*Mitochondrion-localized small heat shock protein 23.6*
AT2G21000	*Transposable element gene*
AT1G07400	*HSP20-like chaperones superfamily protein*
AT5G38120	*4-coumarate--CoA ligase family protein / 4-coumaroyl-CoA synthase family protein*
AT1G59860	*HSP20-like chaperones superfamily protein*
AT2G46240	*BCL-2-associated athanogene 6*
AT5G22970	*Unknown protein*
AT1G55580	*LAS (Lateral Suppressor); transcription factor*
AT3G54700	*Carbohydrate transmembrane transporter/ phosphate transmembrane transporter*
AT5G38780	*S-adenosyl-L-methionine-dependent methyltransferases superfamily protein*
AT1G03810	*Nucleic acid-binding, OB-fold-like protein*

AT5G55970	*RING/U-box superfamily protein*
AT2G21320	*B-box zinc finger family protein*
AT1G36060	*Integrase-type DNA-binding superfamily protein*
AT2G38600	*HAD superfamily, subfamily IIIB acid phosphatase*
AT1G59920	*MADS-box family protein*
AT3G27170	*Chloride channel B*
AT2G16385	*Unknown protein*
AT5G52460	*FBD, F-box and Leucine Rich Repeat domains containing protein*
AT2G07738	*Unknown protein*
AT1G58280	*Phosphoglycerate mutase family protein*
AT2G05100	*Photosystem II light harvesting complex gene 2.1*
AT3G62070	*Unknown protein*
AT1G04250	*AUX/IAA transcriptional regulator family protein*
AT4G34550	*F-box family protein*
AT1G27140	*Glutathione S-transferase tau 14*
AT2G47730	*Glutathione S-transferase phi 8*
AT1G78320	*Glutathione S-transferase TAU* 23
AT3G19450	*ATCAD4*
AT1G32900	*UDP-Glycosyltransferase superfamily protein*
AT3G55120	*Chalcone-flavanone isomerase family protein*
AT3G51240	*Flavanone 3-hydroxylase*
AT1G28030	*2-oxoglutarate (2OG) and Fe(II)-dependent oxygenase superfamily protein*
AT1G78390	*NCED9*
AT1G62740	*Stress inducible proteins*
AT5G38100	*S-adenosyl-l-methionine dependent methyl tranferase*
AT1G68825	*ROTUNDIFOLIA like 15 (shoot development)*
AT1G20015	*sno rna*
AT2G16190	*Hydroxyproline-rich glycoprotein family protein (circadian rhythm)*
AT3G48770	*DNA binding protein*
AT3G12050	*Aha1 domain containing protein*
AT1G56170	*Nuclear factor Y, subunit C2*
AT1G64195	*Defencin like protein*
AT3G04710	ankyrin repeat family protein
AT1G79920	hsp70 family protein
AT5G36490	*Gametogenesis related family protein*
AT5G58590	*Ran binding protein (Protein import to nucleus)*
AT1G25430	*Transposable element*
AT1G07410	*RAB GTPase homolog A2B*
AT2G35150	*EXORDIUM like 1*
AT3G12580	*HSP70*
AT2G03200	*Eukaryotic aspartyl protease*
AT5G56080	*Nicotinamine synthase* (plant cell wall modification)
AT3G09350	*FES A (SALT AND HEAT RESPONSIVE GENE) BETA CATENIN REPEAT BINDING PROTEIN*
AT5G59720	*HSP*18.2

AT5G51030	*NAD(P)-binding Rossmann-fold superfamily protein*
AT5G49910	*CHLOROPLAST HSP70-2*
AT5G53700	*RNA BINDING MOTIF*
AT2G26150	*HSFA*
AT3G52370	*FASCICLIN-like arabinogalactan protein 15 precursor*
AT5G02270	*Non-intrinsic ABC protein* 9
AT2G04030	*Chaperone protein htpG family protein*
AT2G06255	*ELF4-like 3*
AT5G19110	*Eukaryotic aspartyl protease family protein*
AT2G02680	*Cysteine/Histidine-rich C1 domain family protein*
AT4G39070	*B-box zinc finger family protein*
AT5G20860	*Plant invertase/pectin methylesterase inhibitor superfamily*
AT3G53230	*ATPase, AAA-type, CDC48 protein*
AT1G30390	*transposable element gene*
AT4G18340	*Glycosyl hydrolase superfamily protein*
AT1G16030	*HSP70B*
AT1G75470	*PURINE PERMEASE*
AT2G45660	*AGAMOUS-like 20*
AT3G27810	*myb domain protein 21*
AT1G18330	*Homeodomain-like superfamily protein*
AT1G65060	*4-coumarate:CoA ligase 3*
AT2G21330	*Fructose-bisphosphate aldolase 1*
AT5G65850	*F-box and associated interaction domains-containing protein*
AT2G38940	*Phosphate transporter 1;4*
AT5G35120	*MADS-box family protein*
AT3G62950	*Thioredoxin superfamily protein*
AT1G65970	*Thioredoxin peroxidase*2
AT1G10620	*GSTF*4
ATGSTF14	*GSTF14*
ATGSTF10	*GSTF*10

Supplementary Table S4. Differentially expressed genes grouped in different functional and pathways categories in *ein2* .

Up-regulated genes grouped in different cellular functions categories

Category	Term	Gene Count
GOTERM_MF_ALL	GO:0004672~protein kinase activity	88
GOTERM_MF_ALL	GO:0016301~kinase activity	100
GOTERM_BP_ALL	GO:0006468~protein amino acid phosphorylation	84
GOTERM_MF_ALL	GO:0016773~phosphotransferase activity, alcohol group as acceptor	90
GOTERM_MF_ALL	GO:0004674~protein serine/threonine kinase activity	75
GOTERM_BP_ALL	GO:0016310~phosphorylation	85
GOTERM_BP_ALL	GO:0043687~post-translational protein modification	95
GOTERM_MF_ALL	GO:0016772~transferase activities	101
GOTERM_BP_ALL	GO:0006796~phosphate metabolic process	88
GOTERM_BP_ALL	GO:0006793~phosphorus metabolic process	88
GOTERM_MF_ALL	GO:0004872~receptor activity	51
GOTERM_BP_ALL	GO:0006464~protein modification process	98
GOTERM_MF_ALL	GO:0060089~molecular transducer activity	56
GOTERM_MF_ALL	GO:0004871~signal transducer activity	56
GOTERM_BP_ALL	GO:0006952~defense response	69
GOTERM_MF_ALL	GO:0001883~purine nucleoside binding	129
GOTERM_MF_ALL	GO:0030554~adenyl nucleotide binding	129
GOTERM_MF_ALL	GO:0001882~nucleoside binding	129
GOTERM_BP_ALL	GO:0043412~biopolymer modification	100
GOTERM_BP_ALL	GO:0045087~innate immune response	28
GOTERM_BP_ALL	GO:0006955~immune response	29
GOTERM_CC_ALL	GO:0012505~endomembrane system	180
GOTERM_MF_ALL	GO:0005516~calmodulin binding	21
GOTERM_MF_ALL	GO:0032559~adenyl ribonucleotide binding	118
GOTERM_BP_ALL	GO:0002376~immune system process	29
GOTERM_MF_ALL	GO:0030246~carbohydrate binding	23
GOTERM_BP_ALL	GO:0009814~defense response, incompatible interaction	15
GOTERM_MF_ALL	GO:0004713~protein tyrosine kinase activity	28
GOTERM_MF_ALL	GO:0017076~purine nucleotide binding	131
GOTERM_MF_ALL	GO:0005524~ATP binding	114
GOTERM_MF_ALL	GO:0016740~transferase activity	133
GOTERM_MF_ALL	GO:0032555~purine ribonucleotide binding	120
GOTERM_MF_ALL	GO:0032553~ribonucleotide binding	120
GOTERM_BP_ALL	GO:0009617~response to bacterium	22
GOTERM_BP_ALL	GO:0051704~multi-organism process	56
GOTERM_BP_ALL	GO:0009607~response to biotic stimulus	51
GOTERM_MF_ALL	GO:0005509~calcium ion binding	29
GOTERM_BP_ALL	GO:0051707~response to other organism	48
GOTERM_MF_ALL	GO:0003824~catalytic activity	317
GOTERM_BP_ALL	GO:0050896~response to stimulus	165
GOTERM_MF_ALL	GO:0004888~transmembrane receptor activity	18
GOTERM_CC_ALL	GO:0031224~intrinsic to membrane	115
GOTERM_BP_ALL	GO:0009751~response to salicylic acid stimulus	15
GOTERM_BP_ALL	GO:0008037~cell recognition	7

GOTERM_BP_ALL	GO:0048544~recognition of pollen	7
GOTERM_BP_ALL	GO:0010200~response to chitin	13
GOTERM_BP_ALL	GO:0006869~lipid transport	14
GOTERM_BP_ALL	GO:0042742~defense response to bacterium	16
GOTERM_MF_ALL	GO:0000166~nucleotide binding	137
GOTERM_BP_ALL	GO:0010033~response to organic substance	59
GOTERM_BP_ALL	GO:0010120~camalexin biosynthetic process	4
GOTERM_BP_ALL	GO:0052317~camalexin metabolic process	4
GOTERM_BP_ALL	GO:0030001~metal ion transport	19
GOTERM_BP_ALL	GO:0009875~pollen-pistil interaction	7
GOTERM_MF_ALL	GO:0005529~sugar binding	12
GOTERM_BP_ALL	GO:0009700~indole phytoalexin biosynthetic process	4
GOTERM_BP_ALL	GO:0042431~indole metabolic process	4
GOTERM_BP_ALL	GO:0052314~phytoalexin metabolic process	4
GOTERM_BP_ALL	GO:0046217~indole phytoalexin metabolic process	4
GOTERM_BP_ALL	GO:0052315~phytoalexin biosynthetic process	4
GOTERM_BP_ALL	GO:0010876~lipid localization	14
GOTERM_BP_ALL	GO:0009627~systemic acquired resistance	6
GOTERM_BP_ALL	GO:0042221~response to chemical stimulus	87
GOTERM_BP_ALL	GO:0031347~regulation of defense response	8
GOTERM_BP_ALL	GO:0008219~cell death	18
GOTERM_BP_ALL	GO:0016265~death	18
GOTERM_BP_ALL	GO:0030005~cellular di-, tri-valent inorganic cation homeostasis	7
GOTERM_BP_ALL	GO:0048569~post-embryonic organ development	14
GOTERM_MF_ALL	GO:0005216~ion channel activity	9
GOTERM_BP_ALL	GO:0006811~ion transport	28
GOTERM_MF_ALL	GO:0005515~protein binding	143
GOTERM_CC_ALL	GO:0044425~membrane part	124
GOTERM_BP_ALL	GO:0055066~di-, tri-valent inorganic cation homeostasis	7
GOTERM_BP_ALL	GO:0080134~regulation of response to stress	8
GOTERM_MF_ALL	GO:0016798~hydrolase activity, acting on glycosyl bonds	25
GOTERM_MF_ALL	GO:0005488~binding	419
GOTERM_BP_ALL	GO:0010227~floral organ abscission	4
GOTERM_MF_ALL	GO:0004553~hydrolase activity, hydrolyzing O-glycosyl compounds	24
GOTERM_BP_ALL	GO:0048583~regulation of response to stimulus	12
GOTERM_MF_ALL	GO:0009055~electron carrier activity	33
GOTERM_CC_ALL	GO:0016021~integral to membrane	88
GOTERM_BP_ALL	GO:0009743~response to carbohydrate stimulus	14
GOTERM_BP_ALL	GO:0009838~abscission	4
GOTERM_BP_ALL	GO:0012501~programmed cell death	15
GOTERM_BP_ALL	GO:0048878~chemical homeostasis	10
GOTERM_MF_ALL	GO:0020037~heme binding	21
GOTERM_BP_ALL	GO:0055082~cellular chemical homeostasis	8
GOTERM_CC_ALL	GO:0005618~cell wall	30
GOTERM_BP_ALL	GO:0006950~response to stress	97
GOTERM_BP_ALL	GO:0007165~signal transduction	54
GOTERM_CC_ALL	GO:0030312~external encapsulating structure	30

GOTERM_MF_ALL	GO:0030553~cGMP binding	4
GOTERM_MF_ALL	GO:0019002~GMP binding	4
GOTERM_BP_ALL	GO:0048527~lateral root development	6
GOTERM_MF_ALL	GO:0016491~oxidoreductase activity	63
GOTERM_BP_ALL	GO:0030003~cellular cation homeostasis	7
GOTERM_MF_ALL	GO:0030552~cAMP binding	4
GOTERM_CC_ALL	GO:0005576~extracellular region	52
GOTERM_MF_ALL	GO:0004497~monooxygenase activity	18
GOTERM_MF_ALL	GO:0005506~iron ion binding	34
GOTERM_MF_ALL	GO:0015662~ATPase activity, coupled to transmembrane movement of ion	6
GOTERM_MF_ALL	GO:0050662~coenzyme binding	20
GOTERM_BP_ALL	GO:0042435~indole derivative biosynthetic process	5
GOTERM_MF_ALL	GO:0015075~ion transmembrane transporter activity	28
GOTERM_MF_ALL	GO:0048037~cofactor binding	26
GOTERM_MF_ALL	GO:0046906~tetrapyrrole binding	21
GOTERM_BP_ALL	GO:0015674~di-, tri-valent inorganic cation transport	6
GOTERM_BP_ALL	GO:0048528~post-embryonic root development	6
GOTERM_MF_ALL	GO:0019825~oxygen binding	14
GOTERM_BP_ALL	GO:0006855~multidrug transport	7
GOTERM_BP_ALL	GO:0010102~lateral root morphogenesis	4
GOTERM_BP_ALL	GO:0010101~post-embryonic root morphogenesis	4
GOTERM_MF_ALL	GO:0050660~FAD binding	11
GOTERM_MF_ALL	GO:0016209~antioxidant activity	10
GOTERM_BP_ALL	GO:0002237~response to molecule of bacterial origin	3
GOTERM_BP_ALL	GO:0006032~chitin catabolic process	4
GOTERM_BP_ALL	GO:0006816~calcium ion transport	4
GOTERM_BP_ALL	GO:0006026~aminoglycan catabolic process	4
GOTERM_BP_ALL	GO:0006030~chitin metabolic process	4
GOTERM_MF_ALL	GO:0004842~ubiquitin-protein ligase activity	15
GOTERM_MF_ALL	GO:0022838~substrate specific channel activity	9
GOTERM_CC_ALL	GO:0009505~plant-type cell wall	15
GOTERM_MF_ALL	GO:0004568~chitinase activity	4
GOTERM_BP_ALL	GO:0042434~indole derivative metabolic process	5
GOTERM_BP_ALL	GO:0042430~indole and derivative metabolic process	5
GOTERM_BP_ALL	GO:0015893~drug transport	7
GOTERM_BP_ALL	GO:0065007~biological regulation	155
GOTERM_MF_ALL	GO:0015238~drug transporter activity	7
GOTERM_MF_ALL	GO:0015267~channel activity	9
GOTERM_MF_ALL	GO:0022803~passive transmembrane transporter activity	9
GOTERM_BP_ALL	GO:0042493~response to drug	7
GOTERM_BP_ALL	GO:0006873~cellular ion homeostasis	7
GOTERM_BP_ALL	GO:0055080~cation homeostasis	7
GOTERM_BP_ALL	GO:0009817~defense response to fungus, incompatible interaction	4
GOTERM_MF_ALL	GO:0004012~phospholipid-translocating ATPase activity	3
GOTERM_MF_ALL	GO:0015247~aminophospholipid transporter activity	3
GOTERM_MF_ALL	GO:0016881~acid-amino acid ligase activity	16
GOTERM_MF_ALL	GO:0016208~AMP binding	4

GOTERM_MF_ALL	GO:0022836~gated channel activity	6
GOTERM_MF_ALL	GO:0019787~small conjugating protein ligase activity	15
GOTERM_BP_ALL	GO:0009626~plant-type hypersensitive response	5
GOTERM_BP_ALL	GO:0007154~cell communication	11
GOTERM_BP_ALL	GO:0006022~aminoglycan metabolic process	4
GOTERM_BP_ALL	GO:0009407~toxin catabolic process	5
GOTERM_BP_ALL	GO:0034050~host programmed cell death induced by symbiont	5
GOTERM_BP_ALL	GO:0009404~toxin metabolic process	5
GOTERM_BP_ALL	GO:0044272~sulfur compound biosynthetic process	8
GOTERM_BP_ALL	GO:0009863~salicylic acid mediated signaling pathway	4
GOTERM_BP_ALL	GO:0048585~negative regulation of response to stimulus	6
GOTERM_MF_ALL	GO:0004364~glutathione transferase activity	5
GOTERM_MF_ALL	GO:0015112~nitrate transmembrane transporter activity	3
GOTERM_MF_ALL	GO:0005319~lipid transporter activity	4
GOTERM_MF_ALL	GO:0030551~cyclic nucleotide binding	4
GOTERM_BP_ALL	GO:0015914~phospholipid transport	3
GOTERM_BP_ALL	GO:0050794~regulation of cellular process	129
GOTERM_BP_ALL	GO:0044036~cell wall macromolecule metabolic process	5
GOTERM_MF_ALL	GO:0043167~ion binding	130
GOTERM_BP_ALL	GO:0016998~cell wall macromolecule catabolic process	4
GOTERM_BP_ALL	GO:0042445~hormone metabolic process	6
GOTERM_BP_ALL	GO:0006812~cation transport	19
GOTERM_BP_ALL	GO:0050801~ion homeostasis	7
GOTERM_BP_ALL	GO:0048610~reproductive cellular process	8
GOTERM_MF_ALL	GO:0043169~cation binding	129
GOTERM_MF_ALL	GO:0016684~oxidoreductase activity, acting on peroxide as acceptor	8
GOTERM_MF_ALL	GO:0004601~peroxidase activity	8
GOTERM_CC_ALL	GO:0048046~apoplast	18
GOTERM_MF_ALL	GO:0015085~calcium ion transmembrane transporter activity	3
GOTERM_BP_ALL	GO:0010817~regulation of hormone levels	8
GOTERM_BP_ALL	GO:0031348~negative regulation of defense response	3
GOTERM_BP_ALL	GO:0045088~regulation of innate immune response	4
GOTERM_MF_ALL	GO:0042562~hormone binding	3
GOTERM_MF_ALL	GO:0005548~phospholipid transporter activity	3
GOTERM_MF_ALL	GO:0004712~protein serine/threonine/tyrosine kinase activity	5
GOTERM_BP_ALL	GO:0048437~floral organ development	8
GOTERM_MF_ALL	GO:0042625~ATPase activity, coupled to transmembrane movement of ion	6
GOTERM_BP_ALL	GO:0006915~apoptosis	10
GOTERM_MF_ALL	GO:0008509~anion transmembrane transporter activity	7
GOTERM_MF_ALL	GO:0070001~aspartic-type peptidase activity	6
GOTERM_MF_ALL	GO:0004190~aspartic-type endopeptidase activity	6
GOTERM_MF_ALL	GO:0030247~polysaccharide binding	3
GOTERM_MF_ALL	GO:0008066~glutamate receptor activity	3
GOTERM_MF_ALL	GO:0005230~extracellular ligand-gated ion channel activity	3
GOTERM_MF_ALL	GO:0005234~extracellular-glutamate-gated ion channel activity	3
GOTERM_MF_ALL	GO:0001871~pattern binding	3
GOTERM_MF_ALL	GO:0004970~ionotropic glutamate receptor activity	3

GOTERM_BP_ALL	GO:0009850~auxin metabolic process	4
GOTERM_CC_ALL	GO:0044421~extracellular region part	4
GOTERM_MF_ALL	GO:0015082~di-, tri-valent inorganic cation transmembrane transporter ac	5
GOTERM_BP_ALL	GO:0042446~hormone biosynthetic process	4
GOTERM_BP_ALL	GO:0050776~regulation of immune response	4
GOTERM_BP_ALL	GO:0002682~regulation of immune system process	4
GOTERM_MF_ALL	GO:0022857~transmembrane transporter activity	39
GOTERM_BP_ALL	GO:0009723~response to ethylene stimulus	13
GOTERM_MF_ALL	GO:0003700~transcription factor activity	63
GOTERM_BP_ALL	GO:0009856~pollination	8
GOTERM_BP_ALL	GO:0009851~auxin biosynthetic process	3
GOTERM_BP_ALL	GO:0032446~protein modification by small protein conjugation	8
GOTERM_BP_ALL	GO:0006800~oxygen and reactive oxygen species metabolic process	7
GOTERM_MF_ALL	GO:0005217~intracellular ligand-gated ion channel activity	3
GOTERM_BP_ALL	GO:0055114~oxidation reduction	47
GOTERM_BP_ALL	GO:0051173~positive regulation of nitrogen compound metabolic process	5
GOTERM_BP_ALL	GO:0045935~positive regulation of nucleobase, nucleoside, nucleotide and	5
GOTERM_MF_ALL	GO:0016879~ligase activity, forming carbon-nitrogen bonds	16
GOTERM_BP_ALL	GO:0046777~protein amino acid autophosphorylation	3
GOTERM_BP_ALL	GO:0048518~positive regulation of biological process	11
GOTERM_BP_ALL	GO:0006350~transcription	51
GOTERM_BP_ALL	GO:0043094~cellular metabolic compound salvage	5
GOTERM_MF_ALL	GO:0050378~UDP-glucuronate 4-epimerase activity	2
GOTERM_MF_ALL	GO:0004607~phosphatidylcholine-sterol O-acyltransferase activity	2
GOTERM_BP_ALL	GO:0007166~cell surface receptor linked signal transduction	9
GOTERM_MF_ALL	GO:0030528~transcription regulator activity	70
GOTERM_BP_ALL	GO:0050789~regulation of biological process	134
GOTERM_BP_ALL	GO:0019438~aromatic compound biosynthetic process	11
GOTERM_BP_ALL	GO:0009697~salicylic acid biosynthetic process	2
GOTERM_BP_ALL	GO:0000302~response to reactive oxygen species	8
GOTERM_BP_ALL	GO:0048513~organ development	27
GOTERM_BP_ALL	GO:0010193~response to ozone	3
GOTERM_MF_ALL	GO:0035252~UDP-xylosyltransferase activity	2
GOTERM_BP_ALL	GO:0048731~system development	27
GOTERM_BP_ALL	GO:0009725~response to hormone stimulus	36
GOTERM_MF_ALL	GO:0016705~oxidoreductase activity, acting on paired donors, with incorp	10
GOTERM_BP_ALL	GO:0048364~root development	10
GOTERM_BP_ALL	GO:0022622~root system development	10
GOTERM_BP_ALL	GO:0034614~cellular response to reactive oxygen species	6
GOTERM_BP_ALL	GO:0000165~MAPKKK cascade	2
GOTERM_MF_ALL	GO:0022891~substrate-specific transmembrane transporter activity	31
GOTERM_BP_ALL	GO:0006790~sulfur metabolic process	9
GOTERM_BP_ALL	GO:0009620~response to fungus	19
GOTERM_BP_ALL	GO:0055074~calcium ion homeostasis	3
GOTERM_BP_ALL	GO:0009816~defense response to bacterium, incompatible interaction	3
GOTERM_BP_ALL	GO:0006874~cellular calcium ion homeostasis	3
GOTERM_MF_ALL	GO:0008810~cellulase activity	3

GOTERM_BP_ALL	GO:0034599~cellular response to oxidative stress	6
GOTERM_CC_ALL	GO:0031225~anchored to membrane	14
GOTERM_BP_ALL	GO:0042592~homeostatic process	13
GOTERM_BP_ALL	GO:0043067~regulation of programmed cell death	3
GOTERM_MF_ALL	GO:0016765~transferase activity, transferring alkyl or aryl (other than met	7
GOTERM_BP_ALL	GO:0070647~protein modification by small protein conjugation or removal	8
GOTERM_MF_ALL	GO:0016566~specific transcriptional repressor activity	2
GOTERM_MF_ALL	GO:0004714~transmembrane receptor protein tyrosine kinase activity	2
GOTERM_BP_ALL	GO:0000272~polysaccharide catabolic process	5
GOTERM_BP_ALL	GO:0009886~post-embryonic morphogenesis	5
GOTERM_CC_ALL	GO:0000145~exocyst	3
GOTERM_BP_ALL	GO:0006810~transport	76
GOTERM_BP_ALL	GO:0019725~cellular homeostasis	11
GOTERM_BP_ALL	GO:0009938~negative regulation of gibberellic acid mediated signaling	2
GOTERM_BP_ALL	GO:0016567~protein ubiquitination	7
GOTERM_BP_ALL	GO:0009908~flower development	11
GOTERM_BP_ALL	GO:0019748~secondary metabolic process	18
GOTERM_BP_ALL	GO:0065008~regulation of biological quality	26
GOTERM_BP_ALL	GO:0051234~establishment of localization	76
GOTERM_BP_ALL	GO:0002684~positive regulation of immune system process	3
GOTERM_BP_ALL	GO:0045089~positive regulation of innate immune response	3
GOTERM_BP_ALL	GO:0002218~activation of innate immune response	3
GOTERM_BP_ALL	GO:0002253~activation of immune response	3
GOTERM_BP_ALL	GO:0043455~regulation of secondary metabolic process	3
GOTERM_BP_ALL	GO:0050778~positive regulation of immune response	3
GOTERM_MF_ALL	GO:0015276~ligand-gated ion channel activity	3
GOTERM_MF_ALL	GO:0022834~ligand-gated channel activity	3
GOTERM_MF_ALL	GO:0004784~superoxide dismutase activity	2
GOTERM_MF_ALL	GO:0016721~oxidoreductase activity, acting on superoxide radicals as acce	2
GOTERM_MF_ALL	GO:0004185~serine-type carboxypeptidase activity	4
GOTERM_MF_ALL	GO:0070008~serine-type exopeptidase activity	4
GOTERM_BP_ALL	GO:0009664~plant-type cell wall organization	5
GOTERM_BP_ALL	GO:0010941~regulation of cell death	3
GOTERM_MF_ALL	GO:0046872~metal ion binding	117
GOTERM_BP_ALL	GO:0031349~positive regulation of defense response	3
GOTERM_BP_ALL	GO:0034754~cellular hormone metabolic process	3
GOTERM_BP_ALL	GO:0031325~positive regulation of cellular metabolic process	5
GOTERM_BP_ALL	GO:0045941~positive regulation of transcription	4
GOTERM_MF_ALL	GO:0042285~xylosyltransferase activity	2
GOTERM_MF_ALL	GO:0005338~nucleotide-sugar transmembrane transporter activity	2
GOTERM_MF_ALL	GO:0050664~oxidoreductase activity, acting on NADH or NADPH, with oxy	2
GOTERM_MF_ALL	GO:0008061~chitin binding	2
GOTERM_CC_ALL	GO:0042598~vesicular fraction	3
GOTERM_CC_ALL	GO:0005792~microsome	3
GOTERM_MF_ALL	GO:0005215~transporter activity	46
GOTERM_BP_ALL	GO:0009893~positive regulation of metabolic process	5
GOTERM_BP_ALL	GO:0055085~transmembrane transport	10

GOTERM_BP_ALL	GO:0010628~positive regulation of gene expression	4
GOTERM_BP_ALL	GO:0015706~nitrate transport	2
GOTERM_BP_ALL	GO:0019509~methionine salvage	2
GOTERM_BP_ALL	GO:0031540~regulation of anthocyanin biosynthetic process	2
GOTERM_BP_ALL	GO:0045489~pectin biosynthetic process	2
GOTERM_BP_ALL	GO:0043102~amino acid salvage	2
GOTERM_BP_ALL	GO:0009605~response to external stimulus	15
GOTERM_BP_ALL	GO:0051179~localization	77
GOTERM_BP_ALL	GO:0042545~cell wall modification	7
GOTERM_BP_ALL	GO:0048522~positive regulation of cellular process	7
GOTERM_BP_ALL	GO:0006754~ATP biosynthetic process	6
GOTERM_BP_ALL	GO:0046034~ATP metabolic process	6
GOTERM_MF_ALL	GO:0046983~protein dimerization activity	11
GOTERM_BP_ALL	GO:0008643~carbohydrate transport	5
GOTERM_MF_ALL	GO:0004708~MAP kinase kinase activity	2
GOTERM_MF_ALL	GO:0043565~sequence-specific DNA binding	19
GOTERM_MF_ALL	GO:0042803~protein homodimerization activity	4
GOTERM_BP_ALL	GO:0007243~protein kinase cascade	2
GOTERM_BP_ALL	GO:0009696~salicylic acid metabolic process	2
GOTERM_BP_ALL	GO:0010204~defense response signaling pathway, resistance gene-indepe	2
GOTERM_BP_ALL	GO:0007389~pattern specification process	6
GOTERM_BP_ALL	GO:0006576~biogenic amine metabolic process	4
GOTERM_BP_ALL	GO:0070301~cellular response to hydrogen peroxide	5
GOTERM_BP_ALL	GO:0042744~hydrogen peroxide catabolic process	5
GOTERM_MF_ALL	GO:0008081~phosphoric diester hydrolase activity	4
GOTERM_MF_ALL	GO:0015103~inorganic anion transmembrane transporter activity	4
GOTERM_MF_ALL	GO:0016563~transcription activator activity	7
GOTERM_MF_ALL	GO:0004180~carboxypeptidase activity	4
GOTERM_BP_ALL	GO:0009828~plant-type cell wall loosening	3
GOTERM_MF_ALL	GO:0015144~carbohydrate transmembrane transporter activity	6
GOTERM_BP_ALL	GO:0009144~purine nucleoside triphosphate metabolic process	6
GOTERM_BP_ALL	GO:0009201~ribonucleoside triphosphate biosynthetic process	6
GOTERM_BP_ALL	GO:0009145~purine nucleoside triphosphate biosynthetic process	6
GOTERM_BP_ALL	GO:0009205~purine ribonucleoside triphosphate metabolic process	6
GOTERM_BP_ALL	GO:0009206~purine ribonucleoside triphosphate biosynthetic process	6
GOTERM_BP_ALL	GO:0009199~ribonucleoside triphosphate metabolic process	6
GOTERM_BP_ALL	GO:0015780~nucleotide-sugar transport	2
GOTERM_BP_ALL	GO:0009733~response to auxin stimulus	14
GOTERM_BP_ALL	GO:0042743~hydrogen peroxide metabolic process	5
GOTERM_BP_ALL	GO:0009142~nucleoside triphosphate biosynthetic process	6
GOTERM_BP_ALL	GO:0022406~membrane docking	3
GOTERM_BP_ALL	GO:0048278~vesicle docking	3
GOTERM_MF_ALL	GO:0046873~metal ion transmembrane transporter activity	7
GOTERM_CC_ALL	GO:0005626~insoluble fraction	3
GOTERM_CC_ALL	GO:0005624~membrane fraction	3
GOTERM_BP_ALL	GO:0009141~nucleoside triphosphate metabolic process	6
GOTERM_MF_ALL	GO:0008889~glycerophosphodiester phosphodiesterase activity	2

GOTERM_BP_ALL	GO:0009719~response to endogenous stimulus	36
GOTERM_BP_ALL	GO:0010557~positive regulation of macromolecule biosynthetic process	4
GOTERM_BP_ALL	GO:0042542~response to hydrogen peroxide	6
GOTERM_MF_ALL	GO:0022892~substrate-specific transporter activity	33
GOTERM_BP_ALL	GO:0043562~cellular response to nitrogen levels	2
GOTERM_BP_ALL	GO:0043069~negative regulation of programmed cell death	2
GOTERM_BP_ALL	GO:0009937~regulation of gibberellic acid mediated signaling	2
GOTERM_MF_ALL	GO:0015297~antiporter activity	7
GOTERM_BP_ALL	GO:0009308~amine metabolic process	19
GOTERM_BP_ALL	GO:0006979~response to oxidative stress	12
GOTERM_BP_ALL	GO:0003002~regionalization	5
GOTERM_BP_ALL	GO:0009611~response to wounding	7
GOTERM_MF_ALL	GO:0008113~peptide-methionine-(S)-S-oxide reductase activity	2
GOTERM_BP_ALL	GO:0048827~phyllome development	9
GOTERM_BP_ALL	GO:0009615~response to virus	3
GOTERM_BP_ALL	GO:0006887~exocytosis	3
GOTERM_BP_ALL	GO:0005975~carbohydrate metabolic process	35
GOTERM_BP_ALL	GO:0080010~regulation of oxygen and reactive oxygen species metabolic p	2
GOTERM_BP_ALL	GO:0009962~regulation of flavonoid biosynthetic process	2
GOTERM_BP_ALL	GO:0006879~cellular iron ion homeostasis	2
GOTERM_BP_ALL	GO:0045488~pectin metabolic process	2
GOTERM_BP_ALL	GO:0006801~superoxide metabolic process	2
GOTERM_BP_ALL	GO:0009966~regulation of signal transduction	6
GOTERM_BP_ALL	GO:0048646~anatomical structure formation involved in morphogenesis	5
GOTERM_BP_ALL	GO:0006725~cellular aromatic compound metabolic process	14
GOTERM_BP_ALL	GO:0019219~regulation of nucleobase, nucleoside, nucleotide and nucleic	72
GOTERM_MF_ALL	GO:0045551~cinnamyl-alcohol dehydrogenase activity	2
GOTERM_MF_ALL	GO:0016833~oxo-acid-lyase activity	2
GOTERM_MF_ALL	GO:0042626~ATPase activity, coupled to transmembrane movement of su	8
GOTERM_MF_ALL	GO:0043492~ATPase activity, coupled to movement of substances	8
GOTERM_BP_ALL	GO:0045449~regulation of transcription	71
GOTERM_MF_ALL	GO:0015291~secondary active transmembrane transporter activity	12
GOTERM_BP_ALL	GO:0009891~positive regulation of biosynthetic process	4
GOTERM_BP_ALL	GO:0031328~positive regulation of cellular biosynthetic process	4
GOTERM_CC_ALL	GO:0000267~cell fraction	3
GOTERM_BP_ALL	GO:0010646~regulation of cell communication	6
GOTERM_BP_ALL	GO:0031537~regulation of anthocyanin metabolic process	2
GOTERM_BP_ALL	GO:0060548~negative regulation of cell death	2
GOTERM_BP_ALL	GO:0010167~response to nitrate	2
GOTERM_BP_ALL	GO:0010648~negative regulation of cell communication	3
GOTERM_BP_ALL	GO:0009968~negative regulation of signal transduction	3
GOTERM_CC_ALL	GO:0005615~extracellular space	2
GOTERM_BP_ALL	GO:0010604~positive regulation of macromolecule metabolic process	4
GOTERM_MF_ALL	GO:0004806~triacylglycerol lipase activity	3
GOTERM_BP_ALL	GO:0051171~regulation of nitrogen compound metabolic process	72
GOTERM_MF_ALL	GO:0042973~glucan endo-1,3-beta-D-glucosidase activity	2
GOTERM_MF_ALL	GO:0005388~calcium-transporting ATPase activity	2

GOTERM_BP_ALL	GO:0009853~photorespiration	3
GOTERM_BP_ALL	GO:0009561~megagametogenesis	3
GOTERM_CC_ALL	GO:0031226~intrinsic to plasma membrane	5
GOTERM_BP_ALL	GO:0042128~nitrate assimilation	2
GOTERM_BP_ALL	GO:0010252~auxin homeostasis	2
GOTERM_BP_ALL	GO:0009612~response to mechanical stimulus	2
GOTERM_BP_ALL	GO:0032583~regulation of gene-specific transcription	2
GOTERM_BP_ALL	GO:0042126~nitrate metabolic process	2
GOTERM_CC_ALL	GO:0044448~cell cortex part	3
GOTERM_MF_ALL	GO:0016820~hydrolase activity, acting on acid anhydrides, catalyzing trans	8
GOTERM_BP_ALL	GO:0006875~cellular metal ion homeostasis	3
GOTERM_BP_ALL	GO:0055065~metal ion homeostasis	3
GOTERM_MF_ALL	GO:0016829~lyase activity	14
GOTERM_BP_ALL	GO:0009889~regulation of biosynthetic process	73
GOTERM_BP_ALL	GO:0031326~regulation of cellular biosynthetic process	73
GOTERM_BP_ALL	GO:0010218~response to far red light	3
GOTERM_BP_ALL	GO:0007167~enzyme linked receptor protein signaling pathway	6
GOTERM_BP_ALL	GO:0007169~transmembrane receptor protein tyrosine kinase signaling pa	6
GOTERM_BP_ALL	GO:0055072~iron ion homeostasis	2
GOTERM_MF_ALL	GO:0008374~O-acyltransferase activity	3
GOTERM_CC_ALL	GO:0005938~cell cortex	3
GOTERM_CC_ALL	GO:0016020~membrane	177
GOTERM_BP_ALL	GO:0009150~purine ribonucleotide metabolic process	6
GOTERM_BP_ALL	GO:0009152~purine ribonucleotide biosynthetic process	6
GOTERM_BP_ALL	GO:0009827~plant-type cell wall modification	3
GOTERM_BP_ALL	GO:0009809~lignin biosynthetic process	3
GOTERM_MF_ALL	GO:0004089~carbonate dehydratase activity	2
GOTERM_MF_ALL	GO:0015239~multidrug transporter activity	2
GOTERM_MF_ALL	GO:0004022~alcohol dehydrogenase (NAD) activity	2
GOTERM_BP_ALL	GO:0000096~sulfur amino acid metabolic process	4
GOTERM_BP_ALL	GO:0031323~regulation of cellular metabolic process	75
GOTERM_BP_ALL	GO:0010197~polar nucleus fusion	2
GOTERM_CC_ALL	GO:0005886~plasma membrane	73
GOTERM_BP_ALL	GO:0050832~defense response to fungus	15
GOTERM_BP_ALL	GO:0010556~regulation of macromolecule biosynthetic process	71
GOTERM_BP_ALL	GO:0000741~karyogamy	2
GOTERM_BP_ALL	GO:0048284~organelle fusion	2
GOTERM_BP_ALL	GO:0009559~embryo sac central cell differentiation	2
GOTERM_BP_ALL	GO:0010091~trichome branching	2
GOTERM_BP_ALL	GO:0006164~purine nucleotide biosynthetic process	6
GOTERM_MF_ALL	GO:0016706~oxidoreductase activity, acting on paired donors, with incorp	4
GOTERM_BP_ALL	GO:0006555~methionine metabolic process	3
GOTERM_BP_ALL	GO:0009690~cytokinin metabolic process	2
GOTERM_BP_ALL	GO:0009965~leaf morphogenesis	5
GOTERM_MF_ALL	GO:0022804~active transmembrane transporter activity	21
GOTERM_MF_ALL	GO:0008514~organic anion transmembrane transporter activity	2
GOTERM_BP_ALL	GO:0000097~sulfur amino acid biosynthetic process	3

GOTERM_BP_ALL	GO:0080090~regulation of primary metabolic process	74
GOTERM_BP_ALL	GO:0009260~ribonucleotide biosynthetic process	6
GOTERM_BP_ALL	GO:0006163~purine nucleotide metabolic process	6
GOTERM_MF_ALL	GO:0015405~P-P-bond-hydrolysis-driven transmembrane transporter activ	9
GOTERM_MF_ALL	GO:0017171~serine hydrolase activity	7
GOTERM_MF_ALL	GO:0008236~serine-type peptidase activity	7
GOTERM_MF_ALL	GO:0015399~primary active transmembrane transporter activity	9
GOTERM_BP_ALL	GO:0009636~response to toxin	2
GOTERM_BP_ALL	GO:0006071~glycerol metabolic process	2
GOTERM_BP_ALL	GO:0031667~response to nutrient levels	4
GOTERM_BP_ALL	GO:0032940~secretion by cell	3
GOTERM_BP_ALL	GO:0046903~secretion	3
GOTERM_BP_ALL	GO:0009311~oligosaccharide metabolic process	3
GOTERM_BP_ALL	GO:0051716~cellular response to stimulus	34
GOTERM_MF_ALL	GO:0005385~zinc ion transmembrane transporter activity	2
GOTERM_MF_ALL	GO:0016709~oxidoreductase activity, acting on paired donors, with incorp	3
GOTERM_BP_ALL	GO:0009259~ribonucleotide metabolic process	6
GOTERM_MF_ALL	GO:0016830~carbon-carbon lyase activity	5
GOTERM_BP_ALL	GO:0015698~inorganic anion transport	3
GOTERM_CC_ALL	GO:0005788~endoplasmic reticulum lumen	2
GOTERM_BP_ALL	GO:0019400~alditol metabolic process	2
GOTERM_MF_ALL	GO:0008289~lipid binding	9
GOTERM_MF_ALL	GO:0016564~transcription repressor activity	3
GOTERM_BP_ALL	GO:0010468~regulation of gene expression	74
GOTERM_CC_ALL	GO:0005795~Golgi stack	2
GOTERM_MF_ALL	GO:0070279~vitamin B6 binding	5
GOTERM_MF_ALL	GO:0030170~pyridoxal phosphate binding	5
GOTERM_BP_ALL	GO:0001708~cell fate specification	2
GOTERM_BP_ALL	GO:0045892~negative regulation of transcription, DNA-dependent	2
GOTERM_BP_ALL	GO:0051253~negative regulation of RNA metabolic process	2
GOTERM_BP_ALL	GO:0006568~tryptophan metabolic process	2
GOTERM_BP_ALL	GO:0006586~indolalkylamine metabolic process	2
GOTERM_MF_ALL	GO:0004499~flavin-containing monooxygenase activity	2
GOTERM_BP_ALL	GO:0009734~auxin mediated signaling pathway	5
GOTERM_BP_ALL	GO:0048584~positive regulation of response to stimulus	3
GOTERM_MF_ALL	GO:0004175~endopeptidase activity	11
GOTERM_MF_ALL	GO:0016874~ligase activity	17
GOTERM_CC_ALL	GO:0031012~extracellular matrix	2
GOTERM_BP_ALL	GO:0048519~negative regulation of biological process	13
GOTERM_BP_ALL	GO:0033554~cellular response to stress	15
GOTERM_CC_ALL	GO:0044432~endoplasmic reticulum part	4
GOTERM_BP_ALL	GO:0010015~root morphogenesis	4
GOTERM_BP_ALL	GO:0006997~nucleus organization	2
GOTERM_BP_ALL	GO:0007186~G-protein coupled receptor protein signaling pathway	2
GOTERM_BP_ALL	GO:0009086~methionine biosynthetic process	2
GOTERM_MF_ALL	GO:0016837~carbon-oxygen lyase activity, acting on polysaccharides	2
GOTERM_MF_ALL	GO:0030570~pectate lyase activity	2

GOTERM_MF_ALL	GO:0008238~exopeptidase activity	4
GOTERM_BP_ALL	GO:0048366~leaf development	7
GOTERM_MF_ALL	GO:0016835~carbon-oxygen lyase activity	5
GOTERM_MF_ALL	GO:0015294~solute:cation symporter activity	5
GOTERM_BP_ALL	GO:0009225~nucleotide-sugar metabolic process	2
GOTERM_BP_ALL	GO:0009831~plant-type cell wall modification during multidimensional cell	2
GOTERM_BP_ALL	GO:0046483~heterocycle metabolic process	15
GOTERM_BP_ALL	GO:0006575~cellular amino acid derivative metabolic process	10
GOTERM_BP_ALL	GO:0009753~response to jasmonic acid stimulus	6
GOTERM_BP_ALL	GO:0048825~cotyledon development	2
GOTERM_MF_ALL	GO:0016682~oxidoreductase activity, acting on diphenols and related subs	2
GOTERM_BP_ALL	GO:0009991~response to extracellular stimulus	4
GOTERM_BP_ALL	GO:0009653~anatomical structure morphogenesis	16
GOTERM_BP_ALL	GO:0042547~cell wall modification during multidimensional cell growth	2
GOTERM_BP_ALL	GO:0009737~response to abscisic acid stimulus	10
GOTERM_BP_ALL	GO:0048523~negative regulation of cellular process	7
GOTERM_BP_ALL	GO:0006355~regulation of transcription, DNA-dependent	36
GOTERM_CC_ALL	GO:0046658~anchored to plasma membrane	3
GOTERM_MF_ALL	GO:0005402~cation:sugar symporter activity	4
GOTERM_MF_ALL	GO:0015295~solute:hydrogen symporter activity	4
GOTERM_MF_ALL	GO:0005351~sugar:hydrogen symporter activity	4
GOTERM_BP_ALL	GO:0045165~cell fate commitment	2
GOTERM_MF_ALL	GO:0016671~oxidoreductase activity, acting on sulfur group of donors, dis	2
GOTERM_BP_ALL	GO:0051252~regulation of RNA metabolic process	36
GOTERM_MF_ALL	GO:0016746~transferase activity, transferring acyl groups	10
GOTERM_MF_ALL	GO:0016831~carboxy-lyase activity	3
GOTERM_MF_ALL	GO:0045330~aspartyl esterase activity	3
GOTERM_BP_ALL	GO:0032501~multicellular organismal process	56
GOTERM_MF_ALL	GO:0045735~nutrient reservoir activity	3
GOTERM_BP_ALL	GO:0009808~lignin metabolic process	3
GOTERM_MF_ALL	GO:0016298~lipase activity	5
GOTERM_CC_ALL	GO:0005789~endoplasmic reticulum membrane	3
GOTERM_MF_ALL	GO:0010181~FMN binding	2
GOTERM_BP_ALL	GO:0006305~DNA alkylation	2
GOTERM_BP_ALL	GO:0006306~DNA methylation	2
GOTERM_MF_ALL	GO:0042802~identical protein binding	6
GOTERM_MF_ALL	GO:0005507~copper ion binding	6
GOTERM_MF_ALL	GO:0016407~acetyltransferase activity	3
GOTERM_CC_ALL	GO:0005773~vacuole	20
GOTERM_MF_ALL	GO:0008171~O-methyltransferase activity	2
GOTERM_MF_ALL	GO:0016857~racemase and epimerase activity, acting on carbohydrates an	2
GOTERM_BP_ALL	GO:0006944~membrane fusion	2
GOTERM_CC_ALL	GO:0000325~plant-type vacuole	3
GOTERM_BP_ALL	GO:0009887~organ morphogenesis	6
GOTERM_MF_ALL	GO:0016747~transferase activity, transferring acyl groups other than amin	9
GOTERM_MF_ALL	GO:0016667~oxidoreductase activity, acting on sulfur group of donors	4
GOTERM_CC_ALL	GO:0042175~nuclear envelope-endoplasmic reticulum network	3

GOTERM_MF_ALL	GO:0005484~SNAP receptor activity	2
GOTERM_BP_ALL	GO:0006304~DNA modification	2
GOTERM_BP_ALL	GO:0009165~nucleotide biosynthetic process	6
GOTERM_BP_ALL	GO:0031669~cellular response to nutrient levels	3
GOTERM_BP_ALL	GO:0060255~regulation of macromolecule metabolic process	74
GOTERM_BP_ALL	GO:0019222~regulation of metabolic process	77
GOTERM_BP_ALL	GO:0044267~cellular protein metabolic process	113
GOTERM_BP_ALL	GO:0048645~organ formation	2
GOTERM_BP_ALL	GO:0019538~protein metabolic process	129
GOTERM_CC_ALL	GO:0005887~integral to plasma membrane	2
GOTERM_BP_ALL	GO:0032870~cellular response to hormone stimulus	14
GOTERM_BP_ALL	GO:0009755~hormone-mediated signaling	14
GOTERM_MF_ALL	GO:0051119~sugar transmembrane transporter activity	4
GOTERM_BP_ALL	GO:0006470~protein amino acid dephosphorylation	3
GOTERM_BP_ALL	GO:0009066~aspartate family amino acid metabolic process	3
GOTERM_BP_ALL	GO:0048438~floral whorl development	4
GOTERM_BP_ALL	GO:0006904~vesicle docking during exocytosis	2
GOTERM_BP_ALL	GO:0009867~jasmonic acid mediated signaling pathway	2
GOTERM_BP_ALL	GO:0000902~cell morphogenesis	7
GOTERM_MF_ALL	GO:0015293~symporter activity	5
GOTERM_MF_ALL	GO:0004091~carboxylesterase activity	11
GOTERM_BP_ALL	GO:0009738~abscisic acid mediated signaling	3
GOTERM_BP_ALL	GO:0007242~intracellular signaling cascade	25
GOTERM_MF_ALL	GO:0016854~racemase and epimerase activity	2
GOTERM_MF_ALL	GO:0046982~protein heterodimerization activity	2
GOTERM_BP_ALL	GO:0006730~one-carbon metabolic process	4
GOTERM_BP_ALL	GO:0040007~growth	8
GOTERM_BP_ALL	GO:0044271~nitrogen compound biosynthetic process	16
GOTERM_BP_ALL	GO:0048589~developmental growth	6
GOTERM_MF_ALL	GO:0005261~cation channel activity	2
GOTERM_BP_ALL	GO:0009312~oligosaccharide biosynthetic process	2
GOTERM_CC_ALL	GO:0009705~plant-type vacuole membrane	2
GOTERM_BP_ALL	GO:0009553~embryo sac development	3
GOTERM_BP_ALL	GO:0048367~shoot development	9
GOTERM_MF_ALL	GO:0016679~oxidoreductase activity, acting on diphenols and related subs	2
GOTERM_BP_ALL	GO:0009845~seed germination	2
GOTERM_BP_ALL	GO:0019751~polyol metabolic process	2
GOTERM_BP_ALL	GO:0034404~nucleobase, nucleoside and nucleotide biosynthetic process	6
GOTERM_BP_ALL	GO:0034654~nucleobase, nucleoside, nucleotide and nucleic acid biosynth	6
GOTERM_BP_ALL	GO:0022621~shoot system development	9
GOTERM_MF_ALL	GO:0005102~receptor binding	2
GOTERM_BP_ALL	GO:0010016~shoot morphogenesis	5
GOTERM_BP_ALL	GO:0042401~biogenic amine biosynthetic process	2
GOTERM_BP_ALL	GO:0005976~polysaccharide metabolic process	7
GOTERM_BP_ALL	GO:0006820~anion transport	3
GOTERM_BP_ALL	GO:0044248~cellular catabolic process	29
GOTERM_CC_ALL	GO:0031300~intrinsic to organelle membrane	2

GOTERM_MF_ALL	GO:0050661~NADP or NADPH binding	2
GOTERM_BP_ALL	GO:0031668~cellular response to extracellular stimulus	3
GOTERM_BP_ALL	GO:0040029~regulation of gene expression, epigenetic	5
GOTERM_CC_ALL	GO:0005783~endoplasmic reticulum	13
GOTERM_MF_ALL	GO:0042578~phosphoric ester hydrolase activity	11
GOTERM_MF_ALL	GO:0000287~magnesium ion binding	10
GOTERM_BP_ALL	GO:0032787~monocarboxylic acid metabolic process	11
GOTERM_MF_ALL	GO:0022832~voltage-gated channel activity	2
GOTERM_MF_ALL	GO:0005244~voltage-gated ion channel activity	2
GOTERM_MF_ALL	GO:0016846~carbon-sulfur lyase activity	2
GOTERM_CC_ALL	GO:0030964~NADH dehydrogenase complex	2
GOTERM_CC_ALL	GO:0045271~respiratory chain complex I	2
GOTERM_BP_ALL	GO:0048466~androecium development	2
GOTERM_BP_ALL	GO:0009067~aspartate family amino acid biosynthetic process	2
GOTERM_BP_ALL	GO:0048443~stamen development	2
GOTERM_BP_ALL	GO:0009826~unidimensional cell growth	5
GOTERM_BP_ALL	GO:0060560~developmental growth involved in morphogenesis	5
GOTERM_BP_ALL	GO:0016054~organic acid catabolic process	3
GOTERM_BP_ALL	GO:0046395~carboxylic acid catabolic process	3
GOTERM_MF_ALL	GO:0003993~acid phosphatase activity	2
GOTERM_BP_ALL	GO:0016311~dephosphorylation	3
GOTERM_BP_ALL	GO:0009063~cellular amino acid catabolic process	2
GOTERM_MF_ALL	GO:0016651~oxidoreductase activity, acting on NADH or NADPH	3
GOTERM_BP_ALL	GO:0016458~gene silencing	4
GOTERM_BP_ALL	GO:0042398~cellular amino acid derivative biosynthetic process	6
GOTERM_MF_ALL	GO:0016763~transferase activity, transferring pentosyl groups	2
GOTERM_BP_ALL	GO:0010090~trichome morphogenesis	2
GOTERM_MF_ALL	GO:0046915~transition metal ion transmembrane transporter activity	2
GOTERM_BP_ALL	GO:0009056~catabolic process	40
GOTERM_BP_ALL	GO:0032989~cellular component morphogenesis	7
GOTERM_BP_ALL	GO:0009926~auxin polar transport	2
GOTERM_BP_ALL	GO:0009825~multidimensional cell growth	2
GOTERM_BP_ALL	GO:0006753~nucleoside phosphate metabolic process	7
GOTERM_BP_ALL	GO:0009117~nucleotide metabolic process	7
GOTERM_MF_ALL	GO:0019842~vitamin binding	5
GOTERM_BP_ALL	GO:0009310~amine catabolic process	2
GOTERM_MF_ALL	GO:0004721~phosphoprotein phosphatase activity	6
GOTERM_MF_ALL	GO:0019829~cation-transporting ATPase activity	2
GOTERM_BP_ALL	GO:0010035~response to inorganic substance	16
GOTERM_MF_ALL	GO:0016614~oxidoreductase activity, acting on CH-OH group of donors	5
GOTERM_BP_ALL	GO:0046578~regulation of Ras protein signal transduction	2
GOTERM_BP_ALL	GO:0009914~hormone transport	2
GOTERM_BP_ALL	GO:0051056~regulation of small GTPase mediated signal transduction	2
GOTERM_BP_ALL	GO:0040008~regulation of growth	2
GOTERM_BP_ALL	GO:0009699~phenylpropanoid biosynthetic process	4
GOTERM_BP_ALL	GO:0010629~negative regulation of gene expression	5
GOTERM_BP_ALL	GO:0048869~cellular developmental process	13

GOTERM_BP_ALL	GO:0009698~phenylpropanoid metabolic process	5
GOTERM_BP_ALL	GO:0048440~carpel development	2
GOTERM_BP_ALL	GO:0006519~cellular amino acid and derivative metabolic process	18
GOTERM_MF_ALL	GO:0046914~transition metal ion binding	83
GOTERM_BP_ALL	GO:0016052~carbohydrate catabolic process	6
GOTERM_BP_ALL	GO:0044106~cellular amine metabolic process	12
GOTERM_BP_ALL	GO:0032502~developmental process	54
GOTERM_BP_ALL	GO:0007047~cell wall organization	8
GOTERM_BP_ALL	GO:0030154~cell differentiation	9
GOTERM_BP_ALL	GO:0009057~macromolecule catabolic process	20
GOTERM_BP_ALL	GO:0009069~serine family amino acid metabolic process	2
GOTERM_BP_ALL	GO:0009873~ethylene mediated signaling pathway	5
GOTERM_CC_ALL	GO:0070469~respiratory chain	3
GOTERM_MF_ALL	GO:0015035~protein disulfide oxidoreductase activity	2
GOTERM_BP_ALL	GO:0009791~post-embryonic development	25
GOTERM_MF_ALL	GO:0004252~serine-type endopeptidase activity	3
GOTERM_CC_ALL	GO:0044431~Golgi apparatus part	3
GOTERM_MF_ALL	GO:0016616~oxidoreductase activity, acting on the CH-OH group of donor	4
GOTERM_BP_ALL	GO:0048467~gynoecium development	2
GOTERM_BP_ALL	GO:0048509~regulation of meristem development	2
GOTERM_BP_ALL	GO:0048581~negative regulation of post-embryonic development	2
GOTERM_BP_ALL	GO:0016049~cell growth	6
GOTERM_MF_ALL	GO:0003677~DNA binding	72
GOTERM_BP_ALL	GO:0010114~response to red light	2
GOTERM_BP_ALL	GO:0000904~cell morphogenesis involved in differentiation	3
GOTERM_BP_ALL	GO:0016441~posttranscriptional gene silencing	3
GOTERM_MF_ALL	GO:0070011~peptidase activity, acting on L-amino acid peptides	15
GOTERM_BP_ALL	GO:0009812~flavonoid metabolic process	2
GOTERM_BP_ALL	GO:0033036~macromolecule localization	20
GOTERM_BP_ALL	GO:0009416~response to light stimulus	13
GOTERM_MF_ALL	GO:0008415~acyltransferase activity	6
GOTERM_BP_ALL	GO:0016481~negative regulation of transcription	2
GOTERM_BP_ALL	GO:0010605~negative regulation of macromolecule metabolic process	5
GOTERM_BP_ALL	GO:0045229~external encapsulating structure organization	8
GOTERM_MF_ALL	GO:0008324~cation transmembrane transporter activity	12
GOTERM_BP_ALL	GO:0006508~proteolysis	30
GOTERM_BP_ALL	GO:0010026~trichome differentiation	2
GOTERM_BP_ALL	GO:0009624~response to nematode	2
GOTERM_BP_ALL	GO:0009072~aromatic amino acid family metabolic process	2
GOTERM_BP_ALL	GO:0035315~hair cell differentiation	2
GOTERM_MF_ALL	GO:0030599~pectinesterase activity	4
GOTERM_MF_ALL	GO:0008233~peptidase activity	16
GOTERM_CC_ALL	GO:0000786~nucleosome	2
GOTERM_BP_ALL	GO:0007275~multicellular organismal development	48
GOTERM_BP_ALL	GO:0009064~glutamine family amino acid metabolic process	2
GOTERM_MF_ALL	GO:0015036~disulfide oxidoreductase activity	2
GOTERM_BP_ALL	GO:0000003~reproduction	26

GOTERM_MF_ALL	GO:0016702~oxidoreductase activity, acting on single donors with incorpo	2
GOTERM_MF_ALL	GO:0004722~protein serine/threonine phosphatase activity	4
GOTERM_BP_ALL	GO:0008361~regulation of cell size	6
GOTERM_CC_ALL	GO:0008287~protein serine/threonine phosphatase complex	2
GOTERM_BP_ALL	GO:0016138~glycoside biosynthetic process	2
GOTERM_CC_ALL	GO:0000151~ubiquitin ligase complex	6
GOTERM_MF_ALL	GO:0051213~dioxygenase activity	2
GOTERM_BP_ALL	GO:0022414~reproductive process	25
GOTERM_BP_ALL	GO:0009314~response to radiation	13
GOTERM_BP_ALL	GO:0055086~nucleobase, nucleoside and nucleotide metabolic process	7
GOTERM_BP_ALL	GO:0048856~anatomical structure development	38
GOTERM_BP_ALL	GO:0051603~proteolysis involved in cellular protein catabolic process	15
GOTERM_MF_ALL	GO:0016887~ATPase activity	12
GOTERM_BP_ALL	GO:0043086~negative regulation of catalytic activity	2
GOTERM_BP_ALL	GO:0009892~negative regulation of metabolic process	5
GOTERM_BP_ALL	GO:0009913~epidermal cell differentiation	3
GOTERM_CC_ALL	GO:0032993~protein-DNA complex	2
GOTERM_BP_ALL	GO:0006520~cellular amino acid metabolic process	10
GOTERM_MF_ALL	GO:0016769~transferase activity, transferring nitrogenous groups	2
GOTERM_CC_ALL	GO:0005794~Golgi apparatus	9
GOTERM_BP_ALL	GO:0051172~negative regulation of nitrogen compound metabolic process	2
GOTERM_BP_ALL	GO:0045934~negative regulation of nucleobase, nucleoside, nucleotide an	2
GOTERM_BP_ALL	GO:0044257~cellular protein catabolic process	15
GOTERM_MF_ALL	GO:0032561~guanyl ribonucleotide binding	7
GOTERM_BP_ALL	GO:0008544~epidermis development	3
GOTERM_BP_ALL	GO:0007398~ectoderm development	3
GOTERM_BP_ALL	GO:0032535~regulation of cellular component size	6
GOTERM_BP_ALL	GO:0008152~metabolic process	317
GOTERM_BP_ALL	GO:0065009~regulation of molecular function	4
GOTERM_MF_ALL	GO:0016787~hydrolase activity	91
GOTERM_MF_ALL	GO:0019001~guanyl nucleotide binding	7
GOTERM_BP_ALL	GO:0034728~nucleosome organization	2
GOTERM_BP_ALL	GO:0010558~negative regulation of macromolecule biosynthetic process	2
GOTERM_BP_ALL	GO:0006334~nucleosome assembly	2
GOTERM_MF_ALL	GO:0016701~oxidoreductase activity, acting on single donors with incorpo	2
GOTERM_MF_ALL	GO:0003779~actin binding	2
GOTERM_MF_ALL	GO:0004650~polygalacturonase activity	2
GOTERM_BP_ALL	GO:0043414~biopolymer methylation	2
GOTERM_BP_ALL	GO:0015833~peptide transport	2
GOTERM_BP_ALL	GO:0006857~oligopeptide transport	2
GOTERM_BP_ALL	GO:0044092~negative regulation of molecular function	2
GOTERM_BP_ALL	GO:0051239~regulation of multicellular organismal process	5
GOTERM_MF_ALL	GO:0016836~hydro-lyase activity	2
GOTERM_BP_ALL	GO:0031497~chromatin assembly	2
GOTERM_BP_ALL	GO:0009309~amine biosynthetic process	5
GOTERM_BP_ALL	GO:0009411~response to UV	2
GOTERM_BP_ALL	GO:0065004~protein-DNA complex assembly	2

GOTERM_BP_ALL	GO:0016042~lipid catabolic process	5
GOTERM_MF_ALL	GO:0008168~methyltransferase activity	6
GOTERM_BP_ALL	GO:0031327~negative regulation of cellular biosynthetic process	2
GOTERM_BP_ALL	GO:0009890~negative regulation of biosynthetic process	2
GOTERM_BP_ALL	GO:0000041~transition metal ion transport	2
GOTERM_BP_ALL	GO:0007568~aging	2
GOTERM_CC_ALL	GO:0044455~mitochondrial membrane part	2
GOTERM_BP_ALL	GO:0030163~protein catabolic process	15
GOTERM_MF_ALL	GO:0016741~transferase activity, transferring one-carbon groups	6
GOTERM_BP_ALL	GO:0030029~actin filament-based process	2
GOTERM_CC_ALL	GO:0005774~vacuolar membrane	2
GOTERM_BP_ALL	GO:0009639~response to red or far red light	4
GOTERM_BP_ALL	GO:0006323~DNA packaging	2
GOTERM_BP_ALL	GO:0019941~modification-dependent protein catabolic process	14
GOTERM_BP_ALL	GO:0043632~modification-dependent macromolecule catabolic process	14
GOTERM_BP_ALL	GO:0009651~response to salt stress	9
GOTERM_BP_ALL	GO:0032259~methylation	2
GOTERM_BP_ALL	GO:0044265~cellular macromolecule catabolic process	15
GOTERM_BP_ALL	GO:0046394~carboxylic acid biosynthetic process	10
GOTERM_BP_ALL	GO:0016053~organic acid biosynthetic process	10
GOTERM_CC_ALL	GO:0044437~vacuolar part	2
GOTERM_BP_ALL	GO:0019752~carboxylic acid metabolic process	20
GOTERM_BP_ALL	GO:0043436~oxoacid metabolic process	20
GOTERM_BP_ALL	GO:0009555~pollen development	3
GOTERM_BP_ALL	GO:0006082~organic acid metabolic process	20
GOTERM_CC_ALL	GO:0031966~mitochondrial membrane	5
GOTERM_MF_ALL	GO:0005083~small GTPase regulator activity	2
GOTERM_BP_ALL	GO:0000160~two-component signal transduction system (phosphorelay)	5
GOTERM_CC_ALL	GO:0044459~plasma membrane part	6
GOTERM_MF_ALL	GO:0016791~phosphatase activity	7
GOTERM_BP_ALL	GO:0009414~response to water deprivation	4
GOTERM_MF_ALL	GO:0042623~ATPase activity, coupled	8
GOTERM_CC_ALL	GO:0044464~cell part	522
GOTERM_CC_ALL	GO:0005623~cell	522
GOTERM_BP_ALL	GO:0051093~negative regulation of developmental process	2
GOTERM_MF_ALL	GO:0004540~ribonuclease activity	2
GOTERM_BP_ALL	GO:0016137~glycoside metabolic process	2
GOTERM_MF_ALL	GO:0030695~GTPase regulator activity	2
GOTERM_MF_ALL	GO:0004519~endonuclease activity	2
GOTERM_BP_ALL	GO:0042180~cellular ketone metabolic process	20
GOTERM_MF_ALL	GO:0022890~inorganic cation transmembrane transporter activity	5
GOTERM_MF_ALL	GO:0008194~UDP-glycosyltransferase activity	5
GOTERM_CC_ALL	GO:0000785~chromatin	2
GOTERM_BP_ALL	GO:0008652~cellular amino acid biosynthetic process	4
GOTERM_BP_ALL	GO:0031324~negative regulation of cellular metabolic process	2
GOTERM_BP_ALL	GO:0009415~response to water	4
GOTERM_CC_ALL	GO:0005740~mitochondrial envelope	5

GOTERM_MF_ALL	GO:0016788~hydrolase activity, acting on ester bonds	26
GOTERM_BP_ALL	GO:0048229~gametophyte development	4
GOTERM_BP_ALL	GO:0043170~macromolecule metabolic process	189
GOTERM_BP_ALL	GO:0034637~cellular carbohydrate biosynthetic process	4
GOTERM_BP_ALL	GO:0009409~response to cold	5
GOTERM_MF_ALL	GO:0031406~carboxylic acid binding	2
GOTERM_MF_ALL	GO:0060589~nucleoside-triphosphatase regulator activity	2
GOTERM_BP_ALL	GO:0048580~regulation of post-embryonic development	3
GOTERM_BP_ALL	GO:0045454~cell redox homeostasis	3
GOTERM_BP_ALL	GO:0010608~posttranscriptional regulation of gene expression	3
GOTERM_MF_ALL	GO:0003774~motor activity	2
GOTERM_BP_ALL	GO:0006333~chromatin assembly or disassembly	2
GOTERM_BP_ALL	GO:0006970~response to osmotic stress	9
GOTERM_BP_ALL	GO:0009909~regulation of flower development	2
GOTERM_BP_ALL	GO:0050790~regulation of catalytic activity	3
GOTERM_MF_ALL	GO:0017111~nucleoside-triphosphatase activity	19
GOTERM_BP_ALL	GO:0009628~response to abiotic stimulus	31
GOTERM_BP_ALL	GO:0009888~tissue development	5
GOTERM_MF_ALL	GO:0016818~hydrolase activity, acting on acid anhydrides, in phosphorus-c	20
GOTERM_BP_ALL	GO:0016192~vesicle-mediated transport	6
GOTERM_MF_ALL	GO:0030145~manganese ion binding	5
GOTERM_MF_ALL	GO:0016817~hydrolase activity, acting on acid anhydrides	20
GOTERM_BP_ALL	GO:0033692~cellular polysaccharide biosynthetic process	2
GOTERM_BP_ALL	GO:0006633~fatty acid biosynthetic process	3
GOTERM_BP_ALL	GO:0048468~cell development	3
GOTERM_BP_ALL	GO:0016051~carbohydrate biosynthetic process	5
GOTERM_BP_ALL	GO:0044260~cellular macromolecule metabolic process	168
GOTERM_BP_ALL	GO:0006807~nitrogen compound metabolic process	86
GOTERM_BP_ALL	GO:0000271~polysaccharide biosynthetic process	2
GOTERM_BP_ALL	GO:0016044~membrane organization	2
GOTERM_BP_ALL	GO:0050793~regulation of developmental process	5
GOTERM_MF_ALL	GO:0008270~zinc ion binding	42
GOTERM_MF_ALL	GO:0008092~cytoskeletal protein binding	2
GOTERM_BP_ALL	GO:0048608~reproductive structure development	17
GOTERM_BP_ALL	GO:0006631~fatty acid metabolic process	4
GOTERM_MF_ALL	GO:0016462~pyrophosphatase activity	19
GOTERM_CC_ALL	GO:0019898~extrinsic to membrane	4
GOTERM_BP_ALL	GO:0006629~lipid metabolic process	18
GOTERM_MF_ALL	GO:0016853~isomerase activity	4
GOTERM_BP_ALL	GO:0022900~electron transport chain	3
GOTERM_MF_ALL	GO:0004857~enzyme inhibitor activity	3
GOTERM_BP_ALL	GO:0009739~response to gibberellin stimulus	2
GOTERM_CC_ALL	GO:0005743~mitochondrial inner membrane	3
GOTERM_MF_ALL	GO:0008757~S-adenosylmethionine-dependent methyltransferase activity	2
GOTERM_BP_ALL	GO:0006644~phospholipid metabolic process	2
GOTERM_MF_ALL	GO:0004518~nuclease activity	3
GOTERM_BP_ALL	GO:0003006~reproductive developmental process	18

GOTERM_BP_ALL	GO:0009408~response to heat	2
GOTERM_BP_ALL	GO:0044238~primary metabolic process	250
GOTERM_BP_ALL	GO:0019637~organophosphate metabolic process	2
GOTERM_BP_ALL	GO:0006399~tRNA metabolic process	2
GOTERM_BP_ALL	GO:0018130~heterocycle biosynthetic process	2
GOTERM_BP_ALL	GO:0010038~response to metal ion	7
GOTERM_CC_ALL	GO:0044429~mitochondrial part	5
GOTERM_BP_ALL	GO:0031640~killing of cells of another organism	5
GOTERM_BP_ALL	GO:0001906~cell killing	5
GOTERM_BP_ALL	GO:0034641~cellular nitrogen compound metabolic process	79
GOTERM_BP_ALL	GO:0009266~response to temperature stimulus	6
GOTERM_BP_ALL	GO:0006139~nucleobase, nucleoside, nucleotide and nucleic acid metaboli	65
GOTERM_CC_ALL	GO:0044427~chromosomal part	2
GOTERM_BP_ALL	GO:0044237~cellular metabolic process	233
GOTERM_BP_ALL	GO:0044264~cellular polysaccharide metabolic process	2
GOTERM_BP_ALL	GO:0006325~chromatin organization	3
GOTERM_BP_ALL	GO:0046686~response to cadmium ion	5
GOTERM_CC_ALL	GO:0005739~mitochondrion	29
GOTERM_BP_ALL	GO:0006511~ubiquitin-dependent protein catabolic process	4
GOTERM_CC_ALL	GO:0019866~organelle inner membrane	3
GOTERM_BP_ALL	GO:0044255~cellular lipid metabolic process	8
GOTERM_CC_ALL	GO:0042579~microbody	2
GOTERM_CC_ALL	GO:0005777~peroxisome	2
GOTERM_BP_ALL	GO:0006066~alcohol metabolic process	4
GOTERM_MF_ALL	GO:0030234~enzyme regulator activity	5
GOTERM_BP_ALL	GO:0051641~cellular localization	8
GOTERM_BP_ALL	GO:0006281~DNA repair	2
GOTERM_CC_ALL	GO:0044430~cytoskeletal part	2
GOTERM_BP_ALL	GO:0051649~establishment of localization in cell	7
GOTERM_MF_ALL	GO:0005525~GTP binding	3
GOTERM_BP_ALL	GO:0006974~response to DNA damage stimulus	2
GOTERM_BP_ALL	GO:0051276~chromosome organization	3
GOTERM_BP_ALL	GO:0006732~coenzyme metabolic process	2
GOTERM_CC_ALL	GO:0005694~chromosome	2
GOTERM_BP_ALL	GO:0065003~macromolecular complex assembly	3
GOTERM_BP_ALL	GO:0034622~cellular macromolecular complex assembly	2
GOTERM_CC_ALL	GO:0005829~cytosol	11
GOTERM_BP_ALL	GO:0010154~fruit development	6
GOTERM_BP_ALL	GO:0009987~cellular process	298
GOTERM_BP_ALL	GO:0043933~macromolecular complex subunit organization	3
GOTERM_BP_ALL	GO:0006259~DNA metabolic process	4
GOTERM_CC_ALL	GO:0005634~nucleus	79
GOTERM_BP_ALL	GO:0034621~cellular macromolecular complex subunit organization	2
GOTERM_BP_ALL	GO:0044262~cellular carbohydrate metabolic process	7
GOTERM_BP_ALL	GO:0008610~lipid biosynthetic process	5
GOTERM_CC_ALL	GO:0005856~cytoskeleton	2
GOTERM_MF_ALL	GO:0016757~transferase activity, transferring glycosyl groups	6

GOTERM_BP_ALL	GO:0048316~seed development	5
GOTERM_CC_ALL	GO:0005730~nucleolus	3
GOTERM_BP_ALL	GO:0022607~cellular component assembly	3
GOTERM_MF_ALL	GO:0016758~transferase activity, transferring hexosyl groups	4
GOTERM_BP_ALL	GO:0006457~protein folding	2
GOTERM_BP_ALL	GO:0034660~ncRNA metabolic process	2
GOTERM_BP_ALL	GO:0046907~intracellular transport	4
GOTERM_MF_ALL	GO:0005198~structural molecule activity	6
GOTERM_BP_ALL	GO:0051186~cofactor metabolic process	2
GOTERM_BP_ALL	GO:0016043~cellular component organization	22
GOTERM_BP_ALL	GO:0045184~establishment of protein localization	5
GOTERM_BP_ALL	GO:0015031~protein transport	5
GOTERM_BP_ALL	GO:0009793~embryonic development ending in seed dormancy	3
GOTERM_CC_ALL	GO:0031090~organelle membrane	11
GOTERM_BP_ALL	GO:0008104~protein localization	5
GOTERM_BP_ALL	GO:0070727~cellular macromolecule localization	2
GOTERM_CC_ALL	GO:0005840~ribosome	4
GOTERM_MF_ALL	GO:0003735~structural constituent of ribosome	3
GOTERM_CC_ALL	GO:0031981~nuclear lumen	4
GOTERM_CC_ALL	GO:0043234~protein complex	22
GOTERM_CC_ALL	GO:0009570~chloroplast stroma	3
GOTERM_BP_ALL	GO:0006091~generation of precursor metabolites and energy	3
GOTERM_BP_ALL	GO:0009790~embryonic development	3
GOTERM_BP_ALL	GO:0006996~organelle organization	6
GOTERM_CC_ALL	GO:0009532~plastid stroma	3
GOTERM_CC_ALL	GO:0070013~intracellular organelle lumen	6
GOTERM_CC_ALL	GO:0043233~organelle lumen	6
GOTERM_CC_ALL	GO:0031974~membrane-enclosed lumen	6
GOTERM_CC_ALL	GO:0031967~organelle envelope	7
GOTERM_CC_ALL	GO:0031975~envelope	7
GOTERM_CC_ALL	GO:0009579~thylakoid	3
GOTERM_BP_ALL	GO:0034645~cellular macromolecule biosynthetic process	58
GOTERM_BP_ALL	GO:0009059~macromolecule biosynthetic process	58
GOTERM_BP_ALL	GO:0044249~cellular biosynthetic process	91
GOTERM_CC_ALL	GO:0009941~chloroplast envelope	2
GOTERM_BP_ALL	GO:0009058~biosynthetic process	95
GOTERM_CC_ALL	GO:0009526~plastid envelope	2
GOTERM_CC_ALL	GO:0044428~nuclear part	4
GOTERM_CC_ALL	GO:0030529~ribonucleoprotein complex	4
GOTERM_BP_ALL	GO:0016070~RNA metabolic process	4
GOTERM_BP_ALL	GO:0010467~gene expression	56
GOTERM_BP_ALL	GO:0044085~cellular component biogenesis	3
GOTERM_CC_ALL	GO:0043228~non-membrane-bounded organelle	10
GOTERM_CC_ALL	GO:0043232~intracellular non-membrane-bounded organelle	10
GOTERM_MF_ALL	GO:0003676~nucleic acid binding	82
GOTERM_CC_ALL	GO:0032991~macromolecular complex	27
GOTERM_CC_ALL	GO:0044434~chloroplast part	4

GOTERM_CC_ALL	GO:0044435~plastid part	4
GOTERM_CC_ALL	GO:0009507~chloroplast	46
GOTERM_CC_ALL	GO:0009536~plastid	47
GOTERM_CC_ALL	GO:0044444~cytoplasmic part	127
GOTERM_MF_ALL	GO:0003723~RNA binding	7
GOTERM_CC_ALL	GO:0005737~cytoplasm	143
GOTERM_BP_ALL	GO:0006412~translation	4
GOTERM_CC_ALL	GO:0043231~intracellular membrane-bounded organelle	181
GOTERM_CC_ALL	GO:0043227~membrane-bounded organelle	181
GOTERM_BP_ALL	GO:0048464~calyx development	1
GOTERM_BP_ALL	GO:0043068~positive regulation of programmed cell death	1
GOTERM_BP_ALL	GO:0051336~regulation of hydrolase activity	1
GOTERM_BP_ALL	GO:0009823~cytokinin catabolic process	1
GOTERM_BP_ALL	GO:0007010~cytoskeleton organization	1
GOTERM_BP_ALL	GO:0009251~glucan catabolic process	1
GOTERM_BP_ALL	GO:0042402~biogenic amine catabolic process	1
GOTERM_BP_ALL	GO:0000305~response to oxygen radical	1
GOTERM_BP_ALL	GO:0009742~brassinosteroid mediated signaling	1
GOTERM_BP_ALL	GO:0009631~cold acclimation	1
GOTERM_BP_ALL	GO:0010152~pollen maturation	1
GOTERM_BP_ALL	GO:0043039~tRNA aminoacylation	1
GOTERM_BP_ALL	GO:0006916~anti-apoptosis	1
GOTERM_BP_ALL	GO:0051254~positive regulation of RNA metabolic process	1
GOTERM_BP_ALL	GO:0019761~glucosinolate biosynthetic process	1
GOTERM_BP_ALL	GO:0006109~regulation of carbohydrate metabolic process	1
GOTERM_BP_ALL	GO:0033506~glucosinolate biosynthetic process from homomethionine	1
GOTERM_BP_ALL	GO:0010039~response to iron ion	1
GOTERM_BP_ALL	GO:0010439~regulation of glucosinolate biosynthetic process	1
GOTERM_BP_ALL	GO:0048442~sepal development	1
GOTERM_BP_ALL	GO:0048441~petal development	1
GOTERM_BP_ALL	GO:0021700~developmental maturation	1
GOTERM_BP_ALL	GO:0006690~icosanoid metabolic process	1
GOTERM_BP_ALL	GO:0006913~nucleocytoplasmic transport	1
GOTERM_BP_ALL	GO:0010476~gibberellin-mediated signaling	1
GOTERM_BP_ALL	GO:0009861~jasmonic acid and ethylene-dependent systemic resistance	1
GOTERM_BP_ALL	GO:0008654~phospholipid biosynthetic process	1
GOTERM_BP_ALL	GO:0009410~response to xenobiotic stimulus	1
GOTERM_BP_ALL	GO:0042440~pigment metabolic process	1
GOTERM_BP_ALL	GO:0010043~response to zinc ion	1
GOTERM_BP_ALL	GO:0051169~nuclear transport	1
GOTERM_BP_ALL	GO:0030026~cellular manganese ion homeostasis	1
GOTERM_BP_ALL	GO:0007264~small GTPase mediated signal transduction	1
GOTERM_BP_ALL	GO:0008284~positive regulation of cell proliferation	1
GOTERM_BP_ALL	GO:0006351~transcription, DNA-dependent	1
GOTERM_BP_ALL	GO:0048444~floral organ morphogenesis	1
GOTERM_BP_ALL	GO:0009641~shade avoidance	1
GOTERM_BP_ALL	GO:0006352~transcription initiation	1

GOTERM_BP_ALL	GO:0006826~iron ion transport	1
GOTERM_BP_ALL	GO:0051245~negative regulation of cellular defense response	1
GOTERM_BP_ALL	GO:0030245~cellulose catabolic process	1
GOTERM_BP_ALL	GO:0006090~pyruvate metabolic process	1
GOTERM_BP_ALL	GO:0010675~regulation of cellular carbohydrate metabolic process	1
GOTERM_BP_ALL	GO:0032774~RNA biosynthetic process	1
GOTERM_BP_ALL	GO:0015672~monovalent inorganic cation transport	1
GOTERM_BP_ALL	GO:0016143~S-glycoside metabolic process	1
GOTERM_BP_ALL	GO:0042538~hyperosmotic salinity response	1
GOTERM_BP_ALL	GO:0006949~syncytium formation	1
GOTERM_BP_ALL	GO:0048545~response to steroid hormone stimulus	1
GOTERM_BP_ALL	GO:0009684~indoleacetic acid biosynthetic process	1
GOTERM_BP_ALL	GO:0009954~proximal/distal pattern formation	1
GOTERM_BP_ALL	GO:0048449~floral organ formation	1
GOTERM_BP_ALL	GO:0009646~response to absence of light	1
GOTERM_BP_ALL	GO:0051101~regulation of DNA binding	1
GOTERM_BP_ALL	GO:0048507~meristem development	1
GOTERM_BP_ALL	GO:0010075~regulation of meristem growth	1
GOTERM_BP_ALL	GO:0009820~alkaloid metabolic process	1
GOTERM_BP_ALL	GO:0046218~indolalkylamine catabolic process	1
GOTERM_BP_ALL	GO:0051607~defense response to virus	1
GOTERM_BP_ALL	GO:0051188~cofactor biosynthetic process	1
GOTERM_BP_ALL	GO:0000103~sulfate assimilation	1
GOTERM_BP_ALL	GO:0048480~stigma development	1
GOTERM_BP_ALL	GO:0010266~response to vitamin B1	1
GOTERM_BP_ALL	GO:0005986~sucrose biosynthetic process	1
GOTERM_BP_ALL	GO:0006878~cellular copper ion homeostasis	1
GOTERM_BP_ALL	GO:0035295~tube development	1
GOTERM_BP_ALL	GO:0010386~lateral root primordium development	1
GOTERM_BP_ALL	GO:0042219~cellular amino acid derivative catabolic process	1
GOTERM_BP_ALL	GO:0008033~tRNA processing	1
GOTERM_BP_ALL	GO:0009957~epidermal cell fate specification	1
GOTERM_BP_ALL	GO:0006739~NADP metabolic process	1
GOTERM_BP_ALL	GO:0019471~4-hydroxyproline metabolic process	1
GOTERM_BP_ALL	GO:0009992~cellular water homeostasis	1
GOTERM_BP_ALL	GO:0010104~regulation of ethylene mediated signaling pathway	1
GOTERM_BP_ALL	GO:0033273~response to vitamin	1
GOTERM_BP_ALL	GO:0044242~cellular lipid catabolic process	1
GOTERM_BP_ALL	GO:0030258~lipid modification	1
GOTERM_BP_ALL	GO:0031936~negative regulation of chromatin silencing	1
GOTERM_BP_ALL	GO:0034470~ncRNA processing	1
GOTERM_BP_ALL	GO:0043062~extracellular structure organization	1
GOTERM_BP_ALL	GO:0032582~negative regulation of gene-specific transcription	1
GOTERM_BP_ALL	GO:0008300~isoprenoid catabolic process	1
GOTERM_BP_ALL	GO:0033037~polysaccharide localization	1
GOTERM_BP_ALL	GO:0006544~glycine metabolic process	1
GOTERM_BP_ALL	GO:0009582~detection of abiotic stimulus	1

GOTERM_BP_ALL	GO:0009871~jasmonic acid and ethylene-dependent systemic resistance, e	1
GOTERM_BP_ALL	GO:0006829~zinc ion transport	1
GOTERM_BP_ALL	GO:0006882~cellular zinc ion homeostasis	1
GOTERM_BP_ALL	GO:0005985~sucrose metabolic process	1
GOTERM_BP_ALL	GO:0010045~response to nickel ion	1
GOTERM_BP_ALL	GO:0046209~nitric oxide metabolic process	1
GOTERM_BP_ALL	GO:0006541~glutamine metabolic process	1
GOTERM_BP_ALL	GO:0006605~protein targeting	1
GOTERM_BP_ALL	GO:0009581~detection of external stimulus	1
GOTERM_BP_ALL	GO:0032313~regulation of Rab GTPase activity	1
GOTERM_BP_ALL	GO:0019430~removal of superoxide radicals	1
GOTERM_BP_ALL	GO:0043480~pigment accumulation in tissues	1
GOTERM_BP_ALL	GO:0044275~cellular carbohydrate catabolic process	1
GOTERM_BP_ALL	GO:0052544~callose deposition in cell wall during defense response	1
GOTERM_BP_ALL	GO:0046486~glycerolipid metabolic process	1
GOTERM_BP_ALL	GO:0002252~immune effector process	1
GOTERM_BP_ALL	GO:0006006~glucose metabolic process	1
GOTERM_BP_ALL	GO:0010942~positive regulation of cell death	1
GOTERM_BP_ALL	GO:0045815~positive regulation of gene expression, epigenetic	1
GOTERM_BP_ALL	GO:0016570~histone modification	1
GOTERM_BP_ALL	GO:0010224~response to UV-B	1
GOTERM_BP_ALL	GO:0010363~regulation of plant-type hypersensitive response	1
GOTERM_BP_ALL	GO:0005996~monosaccharide metabolic process	1
GOTERM_BP_ALL	GO:0006733~oxidoreduction coenzyme metabolic process	1
GOTERM_BP_ALL	GO:0048829~root cap development	1
GOTERM_BP_ALL	GO:0015784~GDP-mannose transport	1
GOTERM_BP_ALL	GO:0009630~gravitropism	1
GOTERM_BP_ALL	GO:0055069~zinc ion homeostasis	1
GOTERM_BP_ALL	GO:0006809~nitric oxide biosynthetic process	1
GOTERM_BP_ALL	GO:0006972~hyperosmotic response	1
GOTERM_BP_ALL	GO:0042127~regulation of cell proliferation	1
GOTERM_BP_ALL	GO:0034613~cellular protein localization	1
GOTERM_BP_ALL	GO:0043087~regulation of GTPase activity	1
GOTERM_BP_ALL	GO:0007610~behavior	1
GOTERM_BP_ALL	GO:0006414~translational elongation	1
GOTERM_BP_ALL	GO:0050982~detection of mechanical stimulus	1
GOTERM_BP_ALL	GO:0046836~glycolipid transport	1
GOTERM_BP_ALL	GO:0016575~histone deacetylation	1
GOTERM_BP_ALL	GO:0009691~cytokinin biosynthetic process	1
GOTERM_BP_ALL	GO:0046283~anthocyanin metabolic process	1
GOTERM_BP_ALL	GO:0009087~methionine catabolic process	1
GOTERM_BP_ALL	GO:0009862~systemic acquired resistance, salicylic acid mediated signaling	1
GOTERM_BP_ALL	GO:0010434~bract formation	1
GOTERM_BP_ALL	GO:0031047~gene silencing by RNA	1
GOTERM_BP_ALL	GO:0048582~positive regulation of post-embryonic development	1
GOTERM_BP_ALL	GO:0019953~sexual reproduction	1
GOTERM_BP_ALL	GO:0009866~induced systemic resistance, ethylene mediated signaling pat	1

GOTERM_BP_ALL	GO:0052545~callose localization	1
GOTERM_BP_ALL	GO:0051302~regulation of cell division	1
GOTERM_BP_ALL	GO:0010187~negative regulation of seed germination	1
GOTERM_BP_ALL	GO:0007585~respiratory gaseous exchange	1
GOTERM_BP_ALL	GO:0042594~response to starvation	1
GOTERM_BP_ALL	GO:0009629~response to gravity	1
GOTERM_BP_ALL	GO:0010228~vegetative to reproductive phase transition	1
GOTERM_BP_ALL	GO:0010150~leaf senescence	1
GOTERM_BP_ALL	GO:0052546~cell wall pectin metabolic process	1
GOTERM_BP_ALL	GO:0046620~regulation of organ growth	1
GOTERM_BP_ALL	GO:0006563~L-serine metabolic process	1
GOTERM_BP_ALL	GO:0006805~xenobiotic metabolic process	1
GOTERM_BP_ALL	GO:0010260~organ senescence	1
GOTERM_BP_ALL	GO:0010432~bract development	1
GOTERM_BP_ALL	GO:0032776~DNA methylation on cytosine	1
GOTERM_BP_ALL	GO:0030418~nicotianamine biosynthetic process	1
GOTERM_BP_ALL	GO:0009787~regulation of abscisic acid mediated signaling	1
GOTERM_BP_ALL	GO:0010048~vernalization response	1
GOTERM_BP_ALL	GO:0016568~chromatin modification	1
GOTERM_BP_ALL	GO:0007267~cell-cell signaling	1
GOTERM_BP_ALL	GO:0010092~specification of organ identity	1
GOTERM_BP_ALL	GO:0010199~organ boundary specification between lateral organs and the	1
GOTERM_BP_ALL	GO:0006769~nicotinamide metabolic process	1
GOTERM_BP_ALL	GO:0046164~alcohol catabolic process	1
GOTERM_BP_ALL	GO:0070298~negative regulation of two-component signal transduction	1
GOTERM_BP_ALL	GO:0006418~tRNA aminoacylation for protein translation	1
GOTERM_BP_ALL	GO:0034433~steroid esterification	1
GOTERM_BP_ALL	GO:0019760~glucosinolate metabolic process	1
GOTERM_BP_ALL	GO:0030036~actin cytoskeleton organization	1
GOTERM_BP_ALL	GO:0010310~regulation of hydrogen peroxide metabolic process	1
GOTERM_BP_ALL	GO:0005984~disaccharide metabolic process	1
GOTERM_BP_ALL	GO:0060148~positive regulation of posttranscriptional gene silencing	1
GOTERM_BP_ALL	GO:0048465~corolla development	1
GOTERM_BP_ALL	GO:0006891~intra-Golgi vesicle-mediated transport	1
GOTERM_BP_ALL	GO:0048479~style development	1
GOTERM_BP_ALL	GO:0010358~leaf shaping	1
GOTERM_BP_ALL	GO:0006084~acetyl-CoA metabolic process	1
GOTERM_BP_ALL	GO:0010597~green leaf volatile biosynthetic process	1
GOTERM_BP_ALL	GO:0019318~hexose metabolic process	1
GOTERM_BP_ALL	GO:0051241~negative regulation of multicellular organismal process	1
GOTERM_BP_ALL	GO:0009932~cell tip growth	1
GOTERM_BP_ALL	GO:0006086~acetyl-CoA biosynthetic process from pyruvate	1
GOTERM_BP_ALL	GO:0019757~glycosinolate metabolic process	1
GOTERM_BP_ALL	GO:0052542~callose deposition during defense response	1
GOTERM_BP_ALL	GO:0043473~pigmentation	1
GOTERM_BP_ALL	GO:0000304~response to singlet oxygen	1
GOTERM_BP_ALL	GO:0043038~amino acid activation	1

GOTERM_BP_ALL	GO:0007584~response to nutrient	1
GOTERM_BP_ALL	GO:0002239~response to oomycetes	1
GOTERM_BP_ALL	GO:0048563~post-embryonic organ morphogenesis	1
GOTERM_BP_ALL	GO:0000098~sulfur amino acid catabolic process	1
GOTERM_BP_ALL	GO:0009640~photomorphogenesis	1
GOTERM_BP_ALL	GO:0009610~response to symbiotic fungus	1
GOTERM_BP_ALL	GO:0046365~monosaccharide catabolic process	1
GOTERM_BP_ALL	GO:0070297~regulation of two-component signal transduction	1
GOTERM_BP_ALL	GO:0009870~defense response signaling pathway, resistance gene-depend	1
GOTERM_BP_ALL	GO:0030243~cellulose metabolic process	1
GOTERM_BP_ALL	GO:0046219~indolalkylamine biosynthetic process	1
GOTERM_BP_ALL	GO:0009606~tropism	1
GOTERM_BP_ALL	GO:0030198~extracellular matrix organization	1
GOTERM_BP_ALL	GO:0009955~adaxial/abaxial pattern formation	1
GOTERM_BP_ALL	GO:0055071~manganese ion homeostasis	1
GOTERM_BP_ALL	GO:0055070~copper ion homeostasis	1
GOTERM_BP_ALL	GO:0016925~protein sumoylation	1
GOTERM_BP_ALL	GO:0006098~pentose-phosphate shunt	1
GOTERM_BP_ALL	GO:0032483~regulation of Rab protein signal transduction	1
GOTERM_BP_ALL	GO:0045893~positive regulation of transcription, DNA-dependent	1
GOTERM_BP_ALL	GO:0052543~callose deposition in cell wall	1
GOTERM_BP_ALL	GO:0080111~DNA demethylation	1
GOTERM_BP_ALL	GO:0045814~negative regulation of gene expression, epigenetic	1
GOTERM_BP_ALL	GO:0042439~ethanolamine and derivative metabolic process	1
GOTERM_BP_ALL	GO:0010073~meristem maintenance	1
GOTERM_BP_ALL	GO:0030417~nicotianamine metabolic process	1
GOTERM_BP_ALL	GO:0051094~positive regulation of developmental process	1
GOTERM_BP_ALL	GO:0030048~actin filament-based movement	1
GOTERM_BP_ALL	GO:0070482~response to oxygen levels	1
GOTERM_BP_ALL	GO:0044273~sulfur compound catabolic process	1
GOTERM_BP_ALL	GO:0042752~regulation of circadian rhythm	1
GOTERM_BP_ALL	GO:0000162~tryptophan biosynthetic process	1
GOTERM_BP_ALL	GO:0010589~leaf proximal/distal pattern formation	1
GOTERM_BP_ALL	GO:0035195~gene silencing by miRNA	1
GOTERM_BP_ALL	GO:0006284~base-excision repair	1
GOTERM_BP_ALL	GO:0008202~steroid metabolic process	1
GOTERM_BP_ALL	GO:0009073~aromatic amino acid family biosynthetic process	1
GOTERM_BP_ALL	GO:0018208~peptidyl-proline modification	1
GOTERM_BP_ALL	GO:0009451~RNA modification	1
GOTERM_BP_ALL	GO:0009860~pollen tube growth	1
GOTERM_BP_ALL	GO:0006886~intracellular protein transport	1
GOTERM_BP_ALL	GO:0018193~peptidyl-amino acid modification	1
GOTERM_BP_ALL	GO:0009798~axis specification	1
GOTERM_BP_ALL	GO:0010149~senescence	1
GOTERM_BP_ALL	GO:0016103~diterpenoid catabolic process	1
GOTERM_BP_ALL	GO:0048588~developmental cell growth	1
GOTERM_BP_ALL	GO:0010029~regulation of seed germination	1

GOTERM_BP_ALL	GO:0009108~coenzyme biosynthetic process	1
GOTERM_BP_ALL	GO:0010229~inflorescence development	1
GOTERM_BP_ALL	GO:0006461~protein complex assembly	1
GOTERM_BP_ALL	GO:0015692~lead ion transport	1
GOTERM_BP_ALL	GO:0010382~cellular cell wall macromolecule metabolic process	1
GOTERM_BP_ALL	GO:0009740~gibberellic acid mediated signaling	1
GOTERM_BP_ALL	GO:0042981~regulation of apoptosis	1
GOTERM_BP_ALL	GO:0015781~pyrimidine nucleotide-sugar transport	1
GOTERM_BP_ALL	GO:0010051~xylem and phloem pattern formation	1
GOTERM_BP_ALL	GO:0048511~rhythmic process	1
GOTERM_BP_ALL	GO:0010433~bract morphogenesis	1
GOTERM_BP_ALL	GO:0006007~glucose catabolic process	1
GOTERM_BP_ALL	GO:0010383~cell wall polysaccharide metabolic process	1
GOTERM_BP_ALL	GO:0009683~indoleacetic acid metabolic process	1
GOTERM_BP_ALL	GO:0009910~negative regulation of flower development	1
GOTERM_BP_ALL	GO:0009685~gibberellin metabolic process	1
GOTERM_BP_ALL	GO:0048506~regulation of timing of meristematic phase transition	1
GOTERM_BP_ALL	GO:0048510~regulation of timing of transition from vegetative to reprodu	1
GOTERM_BP_ALL	GO:0051606~detection of stimulus	1
GOTERM_BP_ALL	GO:0007638~mechanosensory behavior	1
GOTERM_BP_ALL	GO:0032012~regulation of ARF protein signal transduction	1
GOTERM_BP_ALL	GO:0006396~RNA processing	1
GOTERM_BP_ALL	GO:0016101~diterpenoid metabolic process	1
GOTERM_BP_ALL	GO:0018401~peptidyl-proline hydroxylation to 4-hydroxy-L-proline	1
GOTERM_BP_ALL	GO:0019344~cysteine biosynthetic process	1
GOTERM_BP_ALL	GO:0009608~response to symbiont	1
GOTERM_BP_ALL	GO:0015772~oligosaccharide transport	1
GOTERM_BP_ALL	GO:0043481~anthocyanin accumulation in tissues in response to UV light	1
GOTERM_BP_ALL	GO:0002238~response to molecule of fungal origin	1
GOTERM_BP_ALL	GO:0010393~galacturonan metabolic process	1
GOTERM_BP_ALL	GO:0046496~nicotinamide nucleotide metabolic process	1
GOTERM_BP_ALL	GO:0006612~protein targeting to membrane	1
GOTERM_BP_ALL	GO:0009635~response to herbicide	1
GOTERM_BP_ALL	GO:0006813~potassium ion transport	1
GOTERM_BP_ALL	GO:0019758~glycosinolate biosynthetic process	1
GOTERM_BP_ALL	GO:0000303~response to superoxide	1
GOTERM_BP_ALL	GO:0019458~methionine catabolic process via 2-oxobutanoate	1
GOTERM_BP_ALL	GO:0010306~rhamnogalacturonan II biosynthetic process	1
GOTERM_BP_ALL	GO:0044042~glucan metabolic process	1
GOTERM_BP_ALL	GO:0080135~regulation of cellular response to stress	1
GOTERM_BP_ALL	GO:0043603~cellular amide metabolic process	1
GOTERM_BP_ALL	GO:0033559~unsaturated fatty acid metabolic process	1
GOTERM_BP_ALL	GO:0001666~response to hypoxia	1
GOTERM_BP_ALL	GO:0042436~indole derivative catabolic process	1
GOTERM_BP_ALL	GO:0019320~hexose catabolic process	1
GOTERM_BP_ALL	GO:0015690~aluminum ion transport	1
GOTERM_BP_ALL	GO:0009642~response to light intensity	1

GOTERM_BP_ALL	GO:0009068~aspartate family amino acid catabolic process	1
GOTERM_BP_ALL	GO:0009682~induced systemic resistance	1
GOTERM_BP_ALL	GO:0006884~cell volume homeostasis	1
GOTERM_BP_ALL	GO:0043255~regulation of carbohydrate biosynthetic process	1
GOTERM_BP_ALL	GO:0070271~protein complex biogenesis	1
GOTERM_BP_ALL	GO:0009813~flavonoid biosynthetic process	1
GOTERM_BP_ALL	GO:0035265~organ growth	1
GOTERM_BP_ALL	GO:0052386~cell wall thickening	1
GOTERM_BP_ALL	GO:0010087~phloem or xylem histogenesis	1
GOTERM_BP_ALL	GO:0040034~regulation of development, heterochronic	1
GOTERM_BP_ALL	GO:0010158~abaxial cell fate specification	1
GOTERM_BP_ALL	GO:0019722~calcium-mediated signaling	1
GOTERM_BP_ALL	GO:0046622~positive regulation of organ growth	1
GOTERM_BP_ALL	GO:0006476~protein amino acid deacetylation	1
GOTERM_BP_ALL	GO:0009911~positive regulation of flower development	1
GOTERM_BP_ALL	GO:0048859~formation of anatomical boundary	1
GOTERM_BP_ALL	GO:0006569~tryptophan catabolic process	1
GOTERM_BP_ALL	GO:0016144~S-glycoside biosynthetic process	1
GOTERM_BP_ALL	GO:0046470~phosphatidylcholine metabolic process	1
GOTERM_BP_ALL	GO:0006342~chromatin silencing	1
GOTERM_BP_ALL	GO:0055046~microgametogenesis	1
GOTERM_BP_ALL	GO:0010498~proteasomal protein catabolic process	1
GOTERM_BP_ALL	GO:0052482~cell wall thickening during defense response	1
GOTERM_BP_ALL	GO:0048653~anther development	1
GOTERM_BP_ALL	GO:0009741~response to brassinosteroid stimulus	1
GOTERM_BP_ALL	GO:0051098~regulation of binding	1
GOTERM_BP_ALL	GO:0042762~regulation of sulfur metabolic process	1
GOTERM_BP_ALL	GO:0042447~hormone catabolic process	1
GOTERM_BP_ALL	GO:0043476~pigment accumulation	1
GOTERM_BP_ALL	GO:0031935~regulation of chromatin silencing	1
GOTERM_BP_ALL	GO:0043648~dicarboxylic acid metabolic process	1
GOTERM_BP_ALL	GO:0045927~positive regulation of growth	1
GOTERM_BP_ALL	GO:0045487~gibberellin catabolic process	1
GOTERM_BP_ALL	GO:0051301~cell division	1
GOTERM_BP_ALL	GO:0032318~regulation of Ras GTPase activity	1
GOTERM_BP_ALL	GO:0046688~response to copper ion	1
GOTERM_BP_ALL	GO:0043478~pigment accumulation in response to UV light	1
GOTERM_BP_ALL	GO:0042178~xenobiotic catabolic process	1
GOTERM_BP_ALL	GO:0009267~cellular response to starvation	1
GOTERM_BP_ALL	GO:0048532~anatomical structure arrangement	1
GOTERM_BP_ALL	GO:0010160~formation of organ boundary	1
GOTERM_BP_ALL	GO:0048638~regulation of developmental growth	1
GOTERM_BP_ALL	GO:0010618~aerenchyma formation	1
GOTERM_BP_ALL	GO:0009854~oxidative photosynthetic carbon pathway	1
GOTERM_BP_ALL	GO:0006650~glycerophospholipid metabolic process	1
GOTERM_BP_ALL	GO:0019932~second-messenger-mediated signaling	1
GOTERM_BP_ALL	GO:0019362~pyridine nucleotide metabolic process	1

GOTERM_BP_ALL	GO:0006260~DNA replication	1
GOTERM_BP_ALL	GO:0010249~auxin conjugate metabolic process	1
GOTERM_BP_ALL	GO:0007018~microtubule-based movement	1
GOTERM_BP_ALL	GO:0007017~microtubule-based process	1
GOTERM_BP_ALL	GO:0019439~aromatic compound catabolic process	1
GOTERM_BP_ALL	GO:0009943~adaxial/abaxial axis specification	1
GOTERM_BP_ALL	GO:0006720~isoprenoid metabolic process	1
GOTERM_BP_ALL	GO:0015766~disaccharide transport	1
GOTERM_BP_ALL	GO:0006085~acetyl-CoA biosynthetic process	1
GOTERM_BP_ALL	GO:0043066~negative regulation of apoptosis	1
GOTERM_BP_ALL	GO:0048193~Golgi vesicle transport	1
GOTERM_BP_ALL	GO:0043401~steroid hormone mediated signaling	1
GOTERM_BP_ALL	GO:0051726~regulation of cell cycle	1
GOTERM_BP_ALL	GO:0016115~terpenoid catabolic process	1
GOTERM_BP_ALL	GO:0030705~cytoskeleton-dependent intracellular transport	1
GOTERM_BP_ALL	GO:0015770~sucrose transport	1
GOTERM_BP_ALL	GO:0046417~chorismate metabolic process	1
GOTERM_BP_ALL	GO:0043479~pigment accumulation in tissues in response to UV light	1
GOTERM_BP_ALL	GO:0048868~pollen tube development	1
GOTERM_BP_ALL	GO:0051090~regulation of transcription factor activity	1
GOTERM_BP_ALL	GO:0006821~chloride transport	1
GOTERM_BP_ALL	GO:0046351~disaccharide biosynthetic process	1
GOTERM_BP_ALL	GO:0006954~inflammatory response	1
GOTERM_BP_ALL	GO:0035194~posttranscriptional gene silencing by RNA	1
GOTERM_BP_ALL	GO:0034434~sterol esterification	1
GOTERM_BP_ALL	GO:0060147~regulation of posttranscriptional gene silencing	1
GOTERM_BP_ALL	GO:0010398~xylogalacturonan metabolic process	1
GOTERM_BP_ALL	GO:0006534~cysteine metabolic process	1
GOTERM_BP_ALL	GO:0002229~defense response to oomycetes	1
GOTERM_BP_ALL	GO:0000038~very-long-chain fatty acid metabolic process	1
GOTERM_BP_ALL	GO:0009074~aromatic amino acid family catabolic process	1
GOTERM_BP_ALL	GO:0016569~covalent chromatin modification	1
GOTERM_BP_ALL	GO:0010214~seed coat development	1
GOTERM_BP_ALL	GO:0010185~regulation of cellular defense response	1
GOTERM_BP_ALL	GO:0010093~specification of floral organ identity	1
GOTERM_BP_ALL	GO:0006536~glutamate metabolic process	1
GOTERM_BP_ALL	GO:0009070~serine family amino acid biosynthetic process	1
GOTERM_BP_ALL	GO:0010105~negative regulation of ethylene mediated signaling pathway	1
GOTERM_BP_ALL	GO:0006721~terpenoid metabolic process	1
GOTERM_BP_ALL	GO:0046700~heterocycle catabolic process	1
GOTERM_BP_ALL	GO:0030104~water homeostasis	1
GOTERM_CC_ALL	GO:0043229~intracellular organelle	185
GOTERM_CC_ALL	GO:0030054~cell junction	1
GOTERM_CC_ALL	GO:0005768~endosome	1
GOTERM_CC_ALL	GO:0031461~cullin-RING ubiquitin ligase complex	1
GOTERM_CC_ALL	GO:0034702~ion channel complex	1
GOTERM_CC_ALL	GO:0016602~CCAAT-binding factor complex	1

GOTERM_CC_ALL	GO:0022626~cytosolic ribosome	1
GOTERM_CC_ALL	GO:0000139~Golgi membrane	1
GOTERM_CC_ALL	GO:0005654~nucleoplasm	1
GOTERM_CC_ALL	GO:0005874~microtubule	1
GOTERM_CC_ALL	GO:0019005~SCF ubiquitin ligase complex	1
GOTERM_CC_ALL	GO:0031304~intrinsic to mitochondrial inner membrane	1
GOTERM_CC_ALL	GO:0015629~actin cytoskeleton	1
GOTERM_CC_ALL	GO:0009530~primary cell wall	1
GOTERM_CC_ALL	GO:0043226~organelle	185
GOTERM_CC_ALL	GO:0048196~middle lamella-containing extracellular matrix	1
GOTERM_CC_ALL	GO:0044424~intracellular part	210
GOTERM_CC_ALL	GO:0044422~organelle part	24
GOTERM_CC_ALL	GO:0005911~cell-cell junction	1
GOTERM_CC_ALL	GO:0044446~intracellular organelle part	24
GOTERM_CC_ALL	GO:0055044~symplast	1
GOTERM_CC_ALL	GO:0005622~intracellular	223
GOTERM_CC_ALL	GO:0005578~proteinaceous extracellular matrix	1
GOTERM_CC_ALL	GO:0005950~anthranilate synthase complex	1
GOTERM_CC_ALL	GO:0031201~SNARE complex	1
GOTERM_CC_ALL	GO:0044451~nucleoplasm part	1
GOTERM_CC_ALL	GO:0005744~mitochondrial inner membrane presequence translocase com	1
GOTERM_CC_ALL	GO:0015630~microtubule cytoskeleton	1
GOTERM_CC_ALL	GO:0034707~chloride channel complex	1
GOTERM_CC_ALL	GO:0009506~plasmodesma	1
GOTERM_CC_ALL	GO:0005667~transcription factor complex	1
GOTERM_CC_ALL	GO:0031227~intrinsic to endoplasmic reticulum membrane	1
GOTERM_CC_ALL	GO:0000326~protein storage vacuole	1
GOTERM_CC_ALL	GO:0000322~storage vacuole	1
GOTERM_CC_ALL	GO:0016459~myosin complex	1
GOTERM_CC_ALL	GO:0005875~microtubule associated complex	1
GOTERM_CC_ALL	GO:0009986~cell surface	1
GOTERM_MF_ALL	GO:0003746~translation elongation factor activity	1
GOTERM_MF_ALL	GO:0004565~beta-galactosidase activity	1
GOTERM_MF_ALL	GO:0008429~phosphatidylethanolamine binding	1
GOTERM_MF_ALL	GO:0015926~glucosidase activity	1
GOTERM_MF_ALL	GO:0004197~cysteine-type endopeptidase activity	1
GOTERM_MF_ALL	GO:0051540~metal cluster binding	1
GOTERM_MF_ALL	GO:0005254~chloride channel activity	1
GOTERM_MF_ALL	GO:0004649~poly(ADP-ribose) glycohydrolase activity	1
GOTERM_MF_ALL	GO:0010298~dihydrocamalexic acid decarboxylase activity	1
GOTERM_MF_ALL	GO:0030410~nicotianamine synthase activity	1
GOTERM_MF_ALL	GO:0016413~O-acetyltransferase activity	1
GOTERM_MF_ALL	GO:0004527~exonuclease activity	1
GOTERM_MF_ALL	GO:0008234~cysteine-type peptidase activity	1
GOTERM_MF_ALL	GO:0080104~5-methylthiopropyl glucosinolate S-oxygenase activity	1
GOTERM_MF_ALL	GO:0080095~phosphatidylethanolamine-sterol O-acyltransferase activity	1
GOTERM_MF_ALL	GO:0008447~L-ascorbate oxidase activity	1

GOTERM_MF_ALL	GO:0005201~extracellular matrix structural constituent	1
GOTERM_MF_ALL	GO:0003906~DNA-(apurinic or apyrimidinic site) lyase activity	1
GOTERM_MF_ALL	GO:0045543~gibberellin 2-beta-dioxygenase activity	1
GOTERM_MF_ALL	GO:0004683~calmodulin-dependent protein kinase activity	1
GOTERM_MF_ALL	GO:0050505~hydroquinone glucosyltransferase activity	1
GOTERM_MF_ALL	GO:0035251~UDP-glucosyltransferase activity	1
GOTERM_MF_ALL	GO:0008138~protein tyrosine/serine/threonine phosphatase activity	1
GOTERM_MF_ALL	GO:0015450~P-P-bond-hydrolysis-driven protein transmembrane transpor	1
GOTERM_MF_ALL	GO:0070704~sterol desaturase activity	1
GOTERM_MF_ALL	GO:0015300~solute:solute antiporter activity	1
GOTERM_MF_ALL	GO:0015434~cadmium-transporting ATPase activity	1
GOTERM_MF_ALL	GO:0015299~solute:hydrogen antiporter activity	1
GOTERM_MF_ALL	GO:0004407~histone deacetylase activity	1
GOTERM_MF_ALL	GO:0015925~galactosidase activity	1
GOTERM_MF_ALL	GO:0000822~inositol hexakisphosphate binding	1
GOTERM_MF_ALL	GO:0046857~oxidoreductase activity, acting on other nitrogenous compou	1
GOTERM_MF_ALL	GO:0046522~S-methyl-5-thioribose kinase activity	1
GOTERM_MF_ALL	GO:0043495~protein anchor	1
GOTERM_MF_ALL	GO:0008375~acetylglucosaminyltransferase activity	1
GOTERM_MF_ALL	GO:0003777~microtubule motor activity	1
GOTERM_MF_ALL	GO:0030151~molybdenum ion binding	1
GOTERM_MF_ALL	GO:0004049~anthranilate synthase activity	1
GOTERM_MF_ALL	GO:0004965~GABA-B receptor activity	1
GOTERM_MF_ALL	GO:0016720~delta12-fatty acid dehydrogenase activity	1
GOTERM_MF_ALL	GO:0003712~transcription cofactor activity	1
GOTERM_MF_ALL	GO:0043178~alcohol binding	1
GOTERM_MF_ALL	GO:0010011~auxin binding	1
GOTERM_MF_ALL	GO:0008378~galactosyltransferase activity	1
GOTERM_MF_ALL	GO:0005199~structural constituent of cell wall	1
GOTERM_MF_ALL	GO:0004609~phosphatidylserine decarboxylase activity	1
GOTERM_MF_ALL	GO:0005086~ARF guanyl-nucleotide exchange factor activity	1
GOTERM_MF_ALL	GO:0004673~protein histidine kinase activity	1
GOTERM_MF_ALL	GO:0010427~abscisic acid binding	1
GOTERM_MF_ALL	GO:0003954~NADH dehydrogenase activity	1
GOTERM_MF_ALL	GO:0003973~(S)-2-hydroxy-acid oxidase activity	1
GOTERM_MF_ALL	GO:0016463~zinc-exporting ATPase activity	1
GOTERM_MF_ALL	GO:0016531~copper chaperone activity	1
GOTERM_MF_ALL	GO:0000249~C-22 sterol desaturase activity	1
GOTERM_MF_ALL	GO:0016418~S-acetyltransferase activity	1
GOTERM_MF_ALL	GO:0005458~GDP-mannose transmembrane transporter activity	1
GOTERM_MF_ALL	GO:0019104~DNA N-glycosylase activity	1
GOTERM_MF_ALL	GO:0016638~oxidoreductase activity, acting on the CH-NH2 group of dono	1
GOTERM_MF_ALL	GO:0005267~potassium channel activity	1
GOTERM_MF_ALL	GO:0004564~beta-fructofuranosidase activity	1
GOTERM_MF_ALL	GO:0051020~GTPase binding	1
GOTERM_MF_ALL	GO:0034062~RNA polymerase activity	1
GOTERM_MF_ALL	GO:0003924~GTPase activity	1

GOTERM_MF_ALL	GO:0031405~lipoic acid binding	1
GOTERM_MF_ALL	GO:0000175~3'-5'-exoribonuclease activity	1
GOTERM_MF_ALL	GO:0005249~voltage-gated potassium channel activity	1
GOTERM_MF_ALL	GO:0016876~ligase activity, forming aminoacyl-tRNA and related compoun	1
GOTERM_MF_ALL	GO:0004630~phospholipase D activity	1
GOTERM_MF_ALL	GO:0015198~oligopeptide transporter activity	1
GOTERM_MF_ALL	GO:0008308~voltage-gated anion channel activity	1
GOTERM_MF_ALL	GO:0004930~G-protein coupled receptor activity	1
GOTERM_MF_ALL	GO:0005253~anion channel activity	1
GOTERM_MF_ALL	GO:0016899~oxidoreductase activity, acting on the CH-OH group of donor	1
GOTERM_MF_ALL	GO:0005365~myo-inositol transmembrane transporter activity	1
GOTERM_MF_ALL	GO:0015116~sulfate transmembrane transporter activity	1
GOTERM_MF_ALL	GO:0015166~polyol transmembrane transporter activity	1
GOTERM_MF_ALL	GO:0017089~glycolipid transporter activity	1
GOTERM_MF_ALL	GO:0080107~8-methylthiopropyl glucosinolate S-oxygenase activity	1
GOTERM_MF_ALL	GO:0005543~phospholipid binding	1
GOTERM_MF_ALL	GO:0016417~S-acyltransferase activity	1
GOTERM_MF_ALL	GO:0016291~acyl-CoA thioesterase activity	1
GOTERM_MF_ALL	GO:0080096~phosphatidate-sterol O-acyltransferase activity	1
GOTERM_MF_ALL	GO:0000156~two-component response regulator activity	1
GOTERM_MF_ALL	GO:0019139~cytokinin dehydrogenase activity	1
GOTERM_MF_ALL	GO:0015165~pyrimidine nucleotide sugar transmembrane transporter acti	1
GOTERM_MF_ALL	GO:0016811~hydrolase activity, acting on carbon-nitrogen (but not peptid	1
GOTERM_MF_ALL	GO:0004020~adenylylsulfate kinase activity	1
GOTERM_MF_ALL	GO:0080102~3-methylthiopropyl glucosinolate S-oxygenase activity	1
GOTERM_MF_ALL	GO:0008080~N-acetyltransferase activity	1
GOTERM_MF_ALL	GO:0042910~xenobiotic transporter activity	1
GOTERM_MF_ALL	GO:0004372~glycine hydroxymethyltransferase activity	1
GOTERM_MF_ALL	GO:0019899~enzyme binding	1
GOTERM_MF_ALL	GO:0033925~mannosyl-glycoprotein endo-beta-N-acetylglucosaminidase a	1
GOTERM_MF_ALL	GO:0010279~indole-3-acetic acid amido synthetase activity	1
GOTERM_MF_ALL	GO:0004338~glucan 1,3-beta-glucosidase activity	1
GOTERM_MF_ALL	GO:0019213~deacetylase activity	1
GOTERM_MF_ALL	GO:0004532~exoribonuclease activity	1
GOTERM_MF_ALL	GO:0033293~monocarboxylic acid binding	1
GOTERM_MF_ALL	GO:0015298~solute:cation antiporter activity	1
GOTERM_MF_ALL	GO:0019840~isoprenoid binding	1
GOTERM_MF_ALL	GO:0016799~hydrolase activity, hydrolyzing N-glycosyl compounds	1
GOTERM_MF_ALL	GO:0008565~protein transporter activity	1
GOTERM_MF_ALL	GO:0015086~cadmium ion transmembrane transporter activity	1
GOTERM_MF_ALL	GO:0004742~dihydrolipoyllysine-residue acetyltransferase activity	1
GOTERM_MF_ALL	GO:0009916~alternative oxidase activity	1
GOTERM_MF_ALL	GO:0004679~AMP-activated protein kinase activity	1
GOTERM_MF_ALL	GO:0004616~phosphogluconate dehydrogenase (decarboxylating) activity	1
GOTERM_MF_ALL	GO:0030523~dihydrolipoamide S-acyltransferase activity	1
GOTERM_MF_ALL	GO:0008506~sucrose:hydrogen symporter activity	1
GOTERM_MF_ALL	GO:0016810~hydrolase activity, acting on carbon-nitrogen (but not peptid	1

GOTERM_MF_ALL	GO:0008237~metallopeptidase activity	1
GOTERM_MF_ALL	GO:0048038~quinone binding	1
GOTERM_MF_ALL	GO:0016661~oxidoreductase activity, acting on other nitrogenous compou	1
GOTERM_MF_ALL	GO:0008455~alpha-1,6-mannosylglycoprotein 2-beta-N-acetylglucosaminy	1
GOTERM_MF_ALL	GO:0005099~Ras GTPase activator activity	1
GOTERM_MF_ALL	GO:0016755~transferase activity, transferring amino-acyl groups	1
GOTERM_MF_ALL	GO:0016775~phosphotransferase activity, nitrogenous group as acceptor	1
GOTERM_MF_ALL	GO:0008891~glycolate oxidase activity	1
GOTERM_MF_ALL	GO:0005097~Rab GTPase activator activity	1
GOTERM_MF_ALL	GO:0004812~aminoacyl-tRNA ligase activity	1
GOTERM_MF_ALL	GO:0015197~peptide transporter activity	1
GOTERM_MF_ALL	GO:0008135~translation factor activity, nucleic acid binding	1
GOTERM_MF_ALL	GO:0047720~indoleacetaldoxime dehydratase activity	1
GOTERM_MF_ALL	GO:0022833~mechanically gated channel activity	1
GOTERM_MF_ALL	GO:0008199~ferric iron binding	1
GOTERM_MF_ALL	GO:0070290~NAPE-specific phospholipase D activity	1
GOTERM_MF_ALL	GO:0016896~exoribonuclease activity, producing 5'-phosphomonoesters	1
GOTERM_MF_ALL	GO:0045485~omega-6 fatty acid desaturase activity	1
GOTERM_MF_ALL	GO:0046527~glucosyltransferase activity	1
GOTERM_MF_ALL	GO:0008940~nitrate reductase activity	1
GOTERM_MF_ALL	GO:0003840~gamma-glutamyltransferase activity	1
GOTERM_MF_ALL	GO:0016875~ligase activity, forming carbon-oxygen bonds	1
GOTERM_MF_ALL	GO:0016717~oxidoreductase activity, acting on paired donors, with oxidati	1
GOTERM_MF_ALL	GO:0031404~chloride ion binding	1
GOTERM_MF_ALL	GO:0043168~anion binding	1
GOTERM_MF_ALL	GO:0004392~heme oxygenase (decyclizing) activity	1
GOTERM_MF_ALL	GO:0022843~voltage-gated cation channel activity	1
GOTERM_MF_ALL	GO:0080105~6-methylthiopropyl glucosinolate S-oxygenase activity	1
GOTERM_MF_ALL	GO:0004451~isocitrate lyase activity	1
GOTERM_MF_ALL	GO:0005247~voltage-gated chloride channel activity	1
GOTERM_MF_ALL	GO:0051539~4 iron, 4 sulfur cluster binding	1
GOTERM_MF_ALL	GO:0031386~protein tag	1
GOTERM_MF_ALL	GO:0051861~glycolipid binding	1
GOTERM_MF_ALL	GO:0051536~iron-sulfur cluster binding	1
GOTERM_MF_ALL	GO:0015665~alcohol transmembrane transporter activity	1
GOTERM_MF_ALL	GO:0016847~1-aminocyclopropane-1-carboxylate synthase activity	1
GOTERM_MF_ALL	GO:0016641~oxidoreductase activity, acting on the CH-NH2 group of dono	1
GOTERM_MF_ALL	GO:0004675~transmembrane receptor protein serine/threonine kinase act	1
GOTERM_MF_ALL	GO:0015154~disaccharide transmembrane transporter activity	1
GOTERM_MF_ALL	GO:0016410~N-acyltransferase activity	1
GOTERM_MF_ALL	GO:0008559~xenobiotic-transporting ATPase activity	1
GOTERM_MF_ALL	GO:0046870~cadmium ion binding	1
GOTERM_MF_ALL	GO:0004351~glutamate decarboxylase activity	1
GOTERM_MF_ALL	GO:0051669~fructan beta-fructosidase activity	1
GOTERM_MF_ALL	GO:0015369~calcium:hydrogen antiporter activity	1
GOTERM_MF_ALL	GO:0008131~amine oxidase activity	1
GOTERM_MF_ALL	GO:0005085~guanyl-nucleotide exchange factor activity	1

GOTERM_MF_ALL	GO:0008320~protein transmembrane transporter activity	1
GOTERM_MF_ALL	GO:0019137~thioglucosidase activity	1
GOTERM_MF_ALL	GO:0008381~mechanically-gated ion channel activity	1
GOTERM_MF_ALL	GO:0016645~oxidoreductase activity, acting on the CH-NH group of donor	1
GOTERM_MF_ALL	GO:0050307~sucrose-phosphatase activity	1
GOTERM_MF_ALL	GO:0051082~unfolded protein binding	1
GOTERM_MF_ALL	GO:0009672~auxin:hydrogen symporter activity	1
GOTERM_MF_ALL	GO:0004866~endopeptidase inhibitor activity	1
GOTERM_MF_ALL	GO:0008422~beta-glucosidase activity	1
GOTERM_MF_ALL	GO:0009703~nitrate reductase (NADH) activity	1
GOTERM_MF_ALL	GO:0008134~transcription factor binding	1
GOTERM_MF_ALL	GO:0010327~acetyl CoA:(Z)-3-hexen-1-ol acetyltransferase activity	1
GOTERM_MF_ALL	GO:0008408~3'-5' exonuclease activity	1
GOTERM_MF_ALL	GO:0008515~sucrose transmembrane transporter activity	1
GOTERM_MF_ALL	GO:0005366~myo-inositol:hydrogen symporter activity	1
GOTERM_MF_ALL	GO:0080106~7-methylthiopropyl glucosinolate S-oxygenase activity	1
GOTERM_MF_ALL	GO:0016289~CoA hydrolase activity	1
GOTERM_MF_ALL	GO:0030414~peptidase inhibitor activity	1
GOTERM_MF_ALL	GO:0003702~RNA polymerase II transcription factor activity	1
GOTERM_MF_ALL	GO:0010309~acireductone dioxygenase [iron(II)-requiring] activity	1
GOTERM_MF_ALL	GO:0022884~macromolecule transmembrane transporter activity	1
GOTERM_MF_ALL	GO:0004811~tRNA isopentenyltransferase activity	1
GOTERM_MF_ALL	GO:0008047~enzyme activator activity	1
GOTERM_MF_ALL	GO:0015491~cation:cation antiporter activity	1
GOTERM_MF_ALL	GO:0016917~GABA receptor activity	1
GOTERM_MF_ALL	GO:0016790~thiolester hydrolase activity	1
GOTERM_MF_ALL	GO:0004521~endoribonuclease activity	1
GOTERM_MF_ALL	GO:0018826~methionine gamma-lyase activity	1
GOTERM_MF_ALL	GO:0051139~metal ion:hydrogen antiporter activity	1
GOTERM_MF_ALL	GO:0004867~serine-type endopeptidase inhibitor activity	1
GOTERM_MF_ALL	GO:0033558~protein deacetylase activity	1
GOTERM_MF_ALL	GO:0035198~miRNA binding	1
GOTERM_MF_ALL	GO:0004222~metalloendopeptidase activity	1
GOTERM_MF_ALL	GO:0015368~calcium:cation antiporter activity	1
GOTERM_MF_ALL	GO:0010297~heteroglycan binding	1
GOTERM_MF_ALL	GO:0004725~protein tyrosine phosphatase activity	1
GOTERM_MF_ALL	GO:0016779~nucleotidyltransferase activity	1
GOTERM_MF_ALL	GO:0003968~RNA-directed RNA polymerase activity	1
GOTERM_MF_ALL	GO:0016532~superoxide dismutase copper chaperone activity	1
GOTERM_MF_ALL	GO:0031072~heat shock protein binding	1
GOTERM_MF_ALL	GO:0016796~exonuclease activity, active with either ribo- or deoxyribonuc	1
GOTERM_MF_ALL	GO:0051740~ethylene binding	1
GOTERM_MF_ALL	GO:0008551~cadmium-exporting ATPase activity	1
GOTERM_MF_ALL	GO:0080103~4-methylthiopropyl glucosinolate S-oxygenase activity	1
GOTERM_MF_ALL	GO:0042409~caffeoyl-CoA O-methyltransferase activity	1
GOTERM_MF_ALL	GO:0000155~two-component sensor activity	1
GOTERM_MF_ALL	GO:0016174~NAD(P)H oxidase activity	1

GOTERM_MF_ALL	GO:0005096~GTPase activator activity	1
GOTERM_MF_ALL	GO:0008483~transaminase activity	1
GOTERM_MF_ALL	GO:0003713~transcription coactivator activity	1
GOTERM_MF_ALL	GO:0004535~poly(A)-specific ribonuclease activity	1
GOTERM_MF_ALL	GO:0004620~phospholipase activity	1
GOTERM_MF_ALL	GO:0016530~metallochaperone activity	1
GOTERM_MF_ALL	GO:0031219~levanase activity	1

Pathways up-regulated in *ein* 2

Category	Term	Gene Count
KEGG_PATHWAY	ath00680:Methane metabolism	7
KEGG_PATHWAY	ath00360:Phenylalanine metabolism	6
KEGG_PATHWAY	ath00945:Stilbenoid, diarylheptanoid and gingerol biosynthesis	5
KEGG_PATHWAY	ath00903:Limonene and pinene degradation	5
KEGG_PATHWAY	ath00940:Phenylpropanoid biosynthesis	6
KEGG_PATHWAY	ath00980:Metabolism of xenobiotics by cytochrome P450	3
KEGG_PATHWAY	ath00380:Tryptophan metabolism	3
KEGG_PATHWAY	ath00500:Starch and sucrose metabolism	4
KEGG_PATHWAY	ath01061:Biosynthesis of phenylpropanoids	7
KEGG_PATHWAY	ath00960:Tropane, piperidine and pyridine alkaloid biosynthesis	2
KEGG_PATHWAY	ath00908:Zeatin biosynthesis	2
PANTHER_PATHWAY	P00059:p53 pathway	3
KEGG_PATHWAY	ath00910:Nitrogen metabolism	2
KEGG_PATHWAY	ath04130:SNARE interactions in vesicular transport	2
KEGG_PATHWAY	ath00480:Glutathione metabolism	2
KEGG_PATHWAY	ath00190:Oxidative phosphorylation	3
KEGG_PATHWAY	ath00520:Amino sugar and nucleotide sugar metabolism	2
PANTHER_PATHWAY	P00018:EGF receptor signaling pathway	2
PANTHER_PATHWAY	P00021:FGF signaling pathway	2
PANTHER_PATHWAY	P00029:Huntington disease	2
KEGG_PATHWAY	ath01064:Biosynthesis of alkaloids derived from ornithine, lysine and nicot	2
KEGG_PATHWAY	ath01070:Biosynthesis of plant hormones	2
KEGG_PATHWAY	ath03410:Base excision repair	1
KEGG_PATHWAY	ath00260:Glycine, serine and threonine metabolism	1
KEGG_PATHWAY	ath00100:Steroid biosynthesis	1
KEGG_PATHWAY	ath00564:Glycerophospholipid metabolism	1
KEGG_PATHWAY	ath01063:Biosynthesis of alkaloids derived from shikimate pathway	1
KEGG_PATHWAY	ath00400:Phenylalanine, tyrosine and tryptophan biosynthesis	1
KEGG_PATHWAY	ath00740:Riboflavin metabolism	1
KEGG_PATHWAY	ath03022:Basal transcription factors	1
KEGG_PATHWAY	ath00270:Cysteine and methionine metabolism	1
KEGG_PATHWAY	ath00670:One carbon pool by folate	1
KEGG_PATHWAY	ath01040:Biosynthesis of unsaturated fatty acids	1
KEGG_PATHWAY	ath00071:Fatty acid metabolism	1
KEGG_PATHWAY	ath03010:Ribosome	1
KEGG_PATHWAY	ath00450:Selenoamino acid metabolism	1
KEGG_PATHWAY	ath00040:Pentose and glucuronate interconversions	1

KEGG_PATHWAY	ath04144:Endocytosis	1
KEGG_PATHWAY	ath00350:Tyrosine metabolism	1
KEGG_PATHWAY	ath00460:Cyanoamino acid metabolism	1
KEGG_PATHWAY	ath00565:Ether lipid metabolism	1
KEGG_PATHWAY	ath00010:Glycolysis / Gluconeogenesis	1
KEGG_PATHWAY	ath00630:Glyoxylate and dicarboxylate metabolism	1
KEGG_PATHWAY	ath00944:Flavone and flavonol biosynthesis	1
KEGG_PATHWAY	ath00966:Glucosinolate biosynthesis	1
KEGG_PATHWAY	ath01062:Biosynthesis of terpenoids and steroids	1
PANTHER_PATHWAY	P00055:Transcription regulation by bZIP transcription factor	1
PANTHER_PATHWAY	P05914:Nicotine degradation	1
PANTHER_PATHWAY	P05912:Dopamine receptor mediated signaling pathway	1
PANTHER_PATHWAY	P00049:Parkinson disease	1
PANTHER_PATHWAY	P02778:Sulfate assimilation	1
PANTHER_PATHWAY	P00001:Adrenaline and noradrenaline biosynthesis	1
PANTHER_PATHWAY	P02725:Allantoin degradation	1
PANTHER_PATHWAY	P02783:Tryptophan biosynthesis	1
PANTHER_PATHWAY	P00023:General transcription regulation	1
PANTHER_PATHWAY	P02754:Methylcitrate cycle	1
PANTHER_PATHWAY	P00006:Apoptosis signaling pathway	1
PANTHER_PATHWAY	P00044:Nicotinic acetylcholine receptor signaling pathway	1
PANTHER_PATHWAY	P00037:Ionotropic glutamate receptor pathway	1
PANTHER_PATHWAY	P00057:Wnt signaling pathway	1
PANTHER_PATHWAY	P00039:Metabotropic glutamate receptor group III pathway	1
PANTHER_PATHWAY	P04395:Vasopressin synthesis	1
PANTHER_PATHWAY	P02776:Serine glycine biosynthesis	1
PANTHER_PATHWAY	P00034:Integrin signalling pathway	1
PANTHER_PATHWAY	P04393:Ras Pathway	1
PANTHER_PATHWAY	P00032:Insulin/IGF pathway-mitogen activated protein kinase kinase/MAP	1

Supplementary Table S5. Differentially expressed genes grouped in different functional categories.

Down-regulated genes of *ein* 2 grouped in different cellular function categories (DAVID analysis)

Category	Term	Gene Count
GOTERM_BP_ALL	GO:0009408~response to heat	45
GOTERM_BP_ALL	GO:0042542~response to hydrogen peroxide	32
GOTERM_BP_ALL	GO:0000302~response to reactive oxygen species	33
GOTERM_BP_ALL	GO:0009266~response to temperature stimulus	51
GOTERM_BP_ALL	GO:0006979~response to oxidative stress	42
GOTERM_BP_ALL	GO:0006950~response to stress	151
GOTERM_BP_ALL	GO:0009644~response to high light intensity	18
GOTERM_BP_ALL	GO:0010035~response to inorganic substance	58
GOTERM_BP_ALL	GO:0009642~response to light intensity	19
GOTERM_BP_ALL	GO:0050896~response to stimulus	201
GOTERM_BP_ALL	GO:0009628~response to abiotic stimulus	84
GOTERM_BP_ALL	GO:0042221~response to chemical stimulus	113
GOTERM_BP_ALL	GO:0006457~protein folding	29
GOTERM_BP_ALL	GO:0070301~cellular response to hydrogen peroxide	16
GOTERM_BP_ALL	GO:0042744~hydrogen peroxide catabolic process	16
GOTERM_BP_ALL	GO:0042743~hydrogen peroxide metabolic process	16
GOTERM_BP_ALL	GO:0034614~cellular response to reactive oxygen species	16
GOTERM_BP_ALL	GO:0034599~cellular response to oxidative stress	16
GOTERM_BP_ALL	GO:0009699~phenylpropanoid biosynthetic process	18
GOTERM_MF_ALL	GO:0016684~oxidoreductase activity, acting on peroxide a	17
GOTERM_MF_ALL	GO:0004601~peroxidase activity	17
GOTERM_BP_ALL	GO:0009698~phenylpropanoid metabolic process	20
GOTERM_BP_ALL	GO:0019748~secondary metabolic process	35
GOTERM_BP_ALL	GO:0006800~oxygen and reactive oxygen species metabol	16
GOTERM_BP_ALL	GO:0019438~aromatic compound biosynthetic process	23
GOTERM_MF_ALL	GO:0046906~tetrapyrrole binding	32
GOTERM_BP_ALL	GO:0009813~flavonoid biosynthetic process	11
GOTERM_BP_ALL	GO:0042398~cellular amino acid derivative biosynthetic p	21
GOTERM_BP_ALL	GO:0006725~cellular aromatic compound metabolic proce	30
GOTERM_MF_ALL	GO:0016209~antioxidant activity	17
GOTERM_BP_ALL	GO:0009812~flavonoid metabolic process	11
GOTERM_MF_ALL	GO:0020037~heme binding	29
GOTERM_BP_ALL	GO:0006575~cellular amino acid derivative metabolic pro	25
GOTERM_BP_ALL	GO:0055114~oxidation reduction	67
GOTERM_CC_ALL	GO:0005730~nucleolus	26
GOTERM_MF_ALL	GO:0005506~iron ion binding	44
GOTERM_CC_ALL	GO:0070013~intracellular organelle lumen	40
GOTERM_CC_ALL	GO:0043233~organelle lumen	40
GOTERM_BP_ALL	GO:0009416~response to light stimulus	32
GOTERM_CC_ALL	GO:0031974~membrane-enclosed lumen	40
GOTERM_BP_ALL	GO:0009314~response to radiation	32
GOTERM_MF_ALL	GO:0051082~unfolded protein binding	12
GOTERM_BP_ALL	GO:0010584~pollen exine formation	5
GOTERM_MF_ALL	GO:0015250~water channel activity	7

GOTERM_MF_ALL	GO:0005372~water transporter activity	7
GOTERM_BP_ALL	GO:0009605~response to external stimulus	25
GOTERM_CC_ALL	GO:0005576~extracellular region	61
GOTERM_MF_ALL	GO:0016491~oxidoreductase activity	71
GOTERM_BP_ALL	GO:0044262~cellular carbohydrate metabolic process	32
GOTERM_BP_ALL	GO:0010208~pollen wall assembly	5
GOTERM_BP_ALL	GO:0010927~cellular component assembly involved in mo	5
GOTERM_MF_ALL	GO:0080045~quercetin 3'-O-glucosyltransferase activity	3
GOTERM_CC_ALL	GO:0031981~nuclear lumen	28
GOTERM_BP_ALL	GO:0009611~response to wounding	13
GOTERM_MF_ALL	GO:0043565~sequence-specific DNA binding	28
GOTERM_MF_ALL	GO:0009055~electron carrier activity	35
GOTERM_BP_ALL	GO:0006350~transcription	63
GOTERM_BP_ALL	GO:0010038~response to metal ion	24
GOTERM_BP_ALL	GO:0009753~response to jasmonic acid stimulus	13
GOTERM_MF_ALL	GO:0003700~transcription factor activity	74
GOTERM_BP_ALL	GO:0019751~polyol metabolic process	6
GOTERM_MF_ALL	GO:0016758~transferase activity, transferring hexosyl gro	23
GOTERM_BP_ALL	GO:0006519~cellular amino acid and derivative metabolic	33
GOTERM_BP_ALL	GO:0033554~cellular response to stress	25
GOTERM_BP_ALL	GO:0048229~gametophyte development	14
GOTERM_MF_ALL	GO:0016830~carbon-carbon lyase activity	10
GOTERM_MF_ALL	GO:0035250~UDP-galactosyltransferase activity	3
GOTERM_BP_ALL	GO:0048646~anatomical structure formation involved in m	9
GOTERM_BP_ALL	GO:0010025~wax biosynthetic process	4
GOTERM_BP_ALL	GO:0006020~inositol metabolic process	4
GOTERM_BP_ALL	GO:0010166~wax metabolic process	4
GOTERM_CC_ALL	GO:0043228~non-membrane-bounded organelle	52
GOTERM_CC_ALL	GO:0043232~intracellular non-membrane-bounded organe	52
GOTERM_BP_ALL	GO:0046686~response to cadmium ion	20
GOTERM_BP_ALL	GO:0048869~cellular developmental process	25
GOTERM_MF_ALL	GO:0016563~transcription activator activity	11
GOTERM_MF_ALL	GO:0016831~carboxy-lyase activity	7
GOTERM_MF_ALL	GO:0016829~lyase activity	21
GOTERM_MF_ALL	GO:0008194~UDP-glycosyltransferase activity	15
GOTERM_BP_ALL	GO:0042254~ribosome biogenesis	16
GOTERM_BP_ALL	GO:0031667~response to nutrient levels	8
GOTERM_BP_ALL	GO:0006833~water transport	3
GOTERM_BP_ALL	GO:0042044~fluid transport	3
GOTERM_BP_ALL	GO:0019375~galactolipid biosynthetic process	3
GOTERM_BP_ALL	GO:0019374~galactolipid metabolic process	3
GOTERM_BP_ALL	GO:0044085~cellular component biogenesis	32
GOTERM_BP_ALL	GO:0022613~ribonucleoprotein complex biogenesis	16
GOTERM_BP_ALL	GO:0009415~response to water	13
GOTERM_BP_ALL	GO:0042594~response to starvation	7
GOTERM_MF_ALL	GO:0030528~transcription regulator activity	79
GOTERM_CC_ALL	GO:0044428~nuclear part	31

GOTERM_MF_ALL	GO:0016701~oxidoreductase activity, acting on single don	7
GOTERM_MF_ALL	GO:0051087~chaperone binding	3
GOTERM_BP_ALL	GO:0051716~cellular response to stimulus	46
GOTERM_CC_ALL	GO:0031980~mitochondrial lumen	7
GOTERM_CC_ALL	GO:0005759~mitochondrial matrix	7
GOTERM_MF_ALL	GO:0005528~FK506 binding	4
GOTERM_MF_ALL	GO:0005527~macrolide binding	4
GOTERM_CC_ALL	GO:0005618~cell wall	30
GOTERM_BP_ALL	GO:0009555~pollen development	10
GOTERM_MF_ALL	GO:0016705~oxidoreductase activity, acting on paired don	13
GOTERM_MF_ALL	GO:0046527~glucosyltransferase activity	10
GOTERM_BP_ALL	GO:0016036~cellular response to phosphate starvation	5
GOTERM_CC_ALL	GO:0005773~vacuole	31
GOTERM_MF_ALL	GO:0016757~transferase activity, transferring glycosyl gro	25
GOTERM_CC_ALL	GO:0030312~external encapsulating structure	30
GOTERM_BP_ALL	GO:0009991~response to extracellular stimulus	8
GOTERM_MF_ALL	GO:0008144~drug binding	4
GOTERM_MF_ALL	GO:0035251~UDP-glucosyltransferase activity	9
GOTERM_BP_ALL	GO:0032787~monocarboxylic acid metabolic process	20
GOTERM_MF_ALL	GO:0016872~intramolecular lyase activity	3
GOTERM_BP_ALL	GO:0005975~carbohydrate metabolic process	44
GOTERM_BP_ALL	GO:0006355~regulation of transcription, DNA-dependent	49
GOTERM_CC_ALL	GO:0005634~nucleus	128
GOTERM_MF_ALL	GO:0022838~substrate specific channel activity	9
GOTERM_MF_ALL	GO:0016702~oxidoreductase activity, acting on single don	6
GOTERM_BP_ALL	GO:0051252~regulation of RNA metabolic process	49
GOTERM_MF_ALL	GO:0015267~channel activity	9
GOTERM_MF_ALL	GO:0022803~passive transmembrane transporter activity	9
GOTERM_MF_ALL	GO:0003993~acid phosphatase activity	5
GOTERM_BP_ALL	GO:0009267~cellular response to starvation	6
GOTERM_BP_ALL	GO:0009908~flower development	14
GOTERM_MF_ALL	GO:0051213~dioxygenase activity	6
GOTERM_MF_ALL	GO:0045486~naringenin 3-dioxygenase activity	2
GOTERM_MF_ALL	GO:0016207~4-coumarate-CoA ligase activity	3
GOTERM_BP_ALL	GO:0006631~fatty acid metabolic process	13
GOTERM_BP_ALL	GO:0019853~L-ascorbic acid biosynthetic process	3
GOTERM_BP_ALL	GO:0019852~L-ascorbic acid metabolic process	3
GOTERM_BP_ALL	GO:0009404~toxin metabolic process	5
GOTERM_BP_ALL	GO:0009407~toxin catabolic process	5
GOTERM_MF_ALL	GO:0004091~carboxylesterase activity	18
GOTERM_BP_ALL	GO:0010200~response to chitin	9
GOTERM_MF_ALL	GO:0004364~glutathione transferase activity	5
GOTERM_MF_ALL	GO:0016877~ligase activity, forming carbon-sulfur bonds	4
GOTERM_BP_ALL	GO:0006066~alcohol metabolic process	16
GOTERM_CC_ALL	GO:0000325~plant-type vacuole	6
GOTERM_BP_ALL	GO:0009414~response to water deprivation	11
GOTERM_BP_ALL	GO:0045229~external encapsulating structure organization	16

GOTERM_MF_ALL	GO:0004527~exonuclease activity	6
GOTERM_BP_ALL	GO:0031669~cellular response to nutrient levels	6
GOTERM_MF_ALL	GO:0016762~xyloglucan:xyloglucosyl transferase activity	4
GOTERM_MF_ALL	GO:0016713~oxidoreductase activity, acting on paired don	2
GOTERM_MF_ALL	GO:0015193~L-proline transmembrane transporter activity	2
GOTERM_MF_ALL	GO:0046509~1,2-diacylglycerol 3-beta-galactosyltransfera	2
GOTERM_MF_ALL	GO:0016788~hydrolase activity, acting on ester bonds	43
GOTERM_BP_ALL	GO:0080110~sporopollenin biosynthetic process	2
GOTERM_BP_ALL	GO:0015824~proline transport	2
GOTERM_MF_ALL	GO:0015035~protein disulfide oxidoreductase activity	5
GOTERM_MF_ALL	GO:0019842~vitamin binding	10
GOTERM_BP_ALL	GO:0006970~response to osmotic stress	20
GOTERM_CC_ALL	GO:0005829~cytosol	31
GOTERM_CC_ALL	GO:0005840~ribosome	22
GOTERM_BP_ALL	GO:0006817~phosphate transport	3
GOTERM_BP_ALL	GO:0010286~heat acclimation	3
GOTERM_BP_ALL	GO:0015698~inorganic anion transport	5
GOTERM_MF_ALL	GO:0080044~quercetin 7-O-glucosyltransferase activity	3
GOTERM_BP_ALL	GO:0016051~carbohydrate biosynthetic process	14
GOTERM_CC_ALL	GO:0022626~cytosolic ribosome	16
GOTERM_BP_ALL	GO:0009247~glycolipid biosynthetic process	3
GOTERM_BP_ALL	GO:0045449~regulation of transcription	81
GOTERM_MF_ALL	GO:0005509~calcium ion binding	19
GOTERM_BP_ALL	GO:0034637~cellular carbohydrate biosynthetic process	11
GOTERM_MF_ALL	GO:0016595~glutamate binding	2
GOTERM_BP_ALL	GO:0010197~polar nucleus fusion	3
GOTERM_MF_ALL	GO:0008378~galactosyltransferase activity	4
GOTERM_MF_ALL	GO:0016706~oxidoreductase activity, acting on paired don	6
GOTERM_BP_ALL	GO:0030154~cell differentiation	16
GOTERM_BP_ALL	GO:0006629~lipid metabolic process	34
GOTERM_MF_ALL	GO:0046983~protein dimerization activity	13
GOTERM_BP_ALL	GO:0046165~alcohol biosynthetic process	4
GOTERM_CC_ALL	GO:0048046~apoplast	18
GOTERM_BP_ALL	GO:0042180~cellular ketone metabolic process	34
GOTERM_BP_ALL	GO:0000741~karyogamy	3
GOTERM_BP_ALL	GO:0009559~embryo sac central cell differentiation	3
GOTERM_BP_ALL	GO:0048284~organelle fusion	3
GOTERM_BP_ALL	GO:0006073~cellular glucan metabolic process	8
GOTERM_BP_ALL	GO:0019219~regulation of nucleobase, nucleoside, nucleo	81
GOTERM_MF_ALL	GO:0019825~oxygen binding	12
GOTERM_CC_ALL	GO:0009705~plant-type vacuole membrane	4
GOTERM_BP_ALL	GO:0009651~response to salt stress	18
GOTERM_MF_ALL	GO:0015036~disulfide oxidoreductase activity	5
GOTERM_BP_ALL	GO:0031668~cellular response to extracellular stimulus	6
GOTERM_MF_ALL	GO:0005102~receptor binding	4
GOTERM_MF_ALL	GO:0043765~T/G mismatch-specific endonuclease activity	2
GOTERM_MF_ALL	GO:0017057~6-phosphogluconolactonase activity	2

GOTERM_BP_ALL	GO:0044042~glucan metabolic process	9
GOTERM_BP_ALL	GO:0051171~regulation of nitrogen compound metabolic p	81
GOTERM_MF_ALL	GO:0046910~pectinesterase inhibitor activity	5
GOTERM_BP_ALL	GO:0006082~organic acid metabolic process	33
GOTERM_MF_ALL	GO:0008536~Ran GTPase binding	3
GOTERM_MF_ALL	GO:0016405~CoA-ligase activity	3
GOTERM_BP_ALL	GO:0019752~carboxylic acid metabolic process	33
GOTERM_BP_ALL	GO:0043436~oxoacid metabolic process	33
GOTERM_BP_ALL	GO:0048438~floral whorl development	7
GOTERM_BP_ALL	GO:0006664~glycolipid metabolic process	3
GOTERM_BP_ALL	GO:0051254~positive regulation of RNA metabolic proces	3
GOTERM_MF_ALL	GO:0004497~monooxygenase activity	15
GOTERM_BP_ALL	GO:0048569~post-embryonic organ development	10
GOTERM_BP_ALL	GO:0044264~cellular polysaccharide metabolic process	9
GOTERM_BP_ALL	GO:0010556~regulation of macromolecule biosynthetic pr	81
GOTERM_MF_ALL	GO:0004518~nuclease activity	10
GOTERM_BP_ALL	GO:0009889~regulation of biosynthetic process	82
GOTERM_BP_ALL	GO:0031326~regulation of cellular biosynthetic process	82
GOTERM_MF_ALL	GO:0080043~quercetin 3-O-glucosyltransferase activity	3
GOTERM_MF_ALL	GO:0016878~acid-thiol ligase activity	3
GOTERM_MF_ALL	GO:0031418~L-ascorbic acid binding	3
GOTERM_BP_ALL	GO:0009561~megagametogenesis	4
GOTERM_BP_ALL	GO:0046394~carboxylic acid biosynthetic process	19
GOTERM_BP_ALL	GO:0016053~organic acid biosynthetic process	19
GOTERM_MF_ALL	GO:0045430~chalcone isomerase activity	2
GOTERM_MF_ALL	GO:0000014~single-stranded DNA specific endodeoxyribo	2
GOTERM_MF_ALL	GO:0016157~sucrose synthase activity	2
GOTERM_MF_ALL	GO:0004737~pyruvate decarboxylase activity	2
GOTERM_MF_ALL	GO:0017016~Ras GTPase binding	3
GOTERM_MF_ALL	GO:0031267~small GTPase binding	3
GOTERM_BP_ALL	GO:0048443~stamen development	4
GOTERM_BP_ALL	GO:0048466~androecium development	4
GOTERM_BP_ALL	GO:0048522~positive regulation of cellular process	8
GOTERM_BP_ALL	GO:0006021~inositol biosynthetic process	2
GOTERM_BP_ALL	GO:0015804~neutral amino acid transport	2
GOTERM_MF_ALL	GO:0004428~inositol or phosphatidylinositol kinase activi	4
GOTERM_BP_ALL	GO:0008610~lipid biosynthetic process	19
GOTERM_MF_ALL	GO:0003677~DNA binding	89
GOTERM_MF_ALL	GO:0031406~carboxylic acid binding	6
GOTERM_MF_ALL	GO:0016620~oxidoreductase activity, acting on the aldehy	4
GOTERM_MF_ALL	GO:0003824~catalytic activity	293
GOTERM_MF_ALL	GO:0016887~ATPase activity	20
GOTERM_MF_ALL	GO:0060590~ATPase regulator activity	2
GOTERM_BP_ALL	GO:0031323~regulation of cellular metabolic process	84
GOTERM_BP_ALL	GO:0006997~nucleus organization	3
GOTERM_BP_ALL	GO:0046467~membrane lipid biosynthetic process	3
GOTERM_BP_ALL	GO:0046173~polyol biosynthetic process	2

GOTERM_MF_ALL	GO:0016853~isomerase activity	11
GOTERM_BP_ALL	GO:0044255~cellular lipid metabolic process	21
GOTERM_BP_ALL	GO:0005976~polysaccharide metabolic process	11
GOTERM_MF_ALL	GO:0080046~quercetin 4'-O-glucosyltransferase activity	2
GOTERM_MF_ALL	GO:0080030~methyl indole-3-acetate esterase activity	2
GOTERM_MF_ALL	GO:0051020~GTPase binding	3
GOTERM_CC_ALL	GO:0030529~ribonucleoprotein complex	27
GOTERM_BP_ALL	GO:0080090~regulation of primary metabolic process	83
GOTERM_MF_ALL	GO:0019899~enzyme binding	4
GOTERM_CC_ALL	GO:0030076~light-harvesting complex	3
GOTERM_CC_ALL	GO:0009707~chloroplast outer membrane	3
GOTERM_BP_ALL	GO:0009411~response to UV	5
GOTERM_BP_ALL	GO:0006972~hyperosmotic response	4
GOTERM_MF_ALL	GO:0016765~transferase activity, transferring alkyl or aryl	7
GOTERM_BP_ALL	GO:0051555~flavonol biosynthetic process	2
GOTERM_BP_ALL	GO:0051552~flavone metabolic process	2
GOTERM_BP_ALL	GO:0051553~flavone biosynthetic process	2
GOTERM_BP_ALL	GO:0051554~flavonol metabolic process	2
GOTERM_MF_ALL	GO:0003743~translation initiation factor activity	6
GOTERM_MF_ALL	GO:0016168~chlorophyll binding	3
GOTERM_BP_ALL	GO:0042546~cell wall biogenesis	5
GOTERM_BP_ALL	GO:0009719~response to endogenous stimulus	39
GOTERM_BP_ALL	GO:0009743~response to carbohydrate stimulus	10
GOTERM_MF_ALL	GO:0000175~3'-5'-exoribonuclease activity	3
GOTERM_MF_ALL	GO:0015175~neutral amino acid transmembrane transport	2
GOTERM_MF_ALL	GO:0030976~thiamin pyrophosphate binding	2
GOTERM_MF_ALL	GO:0015179~L-amino acid transmembrane transporter act	2
GOTERM_BP_ALL	GO:0010468~regulation of gene expression	83
GOTERM_BP_ALL	GO:0048437~floral organ development	7
GOTERM_CC_ALL	GO:0031225~anchored to membrane	14
GOTERM_CC_ALL	GO:0012505~endomembrane system	144
GOTERM_BP_ALL	GO:0009113~purine base biosynthetic process	2
GOTERM_BP_ALL	GO:0006308~DNA catabolic process	2
GOTERM_CC_ALL	GO:0005774~vacuolar membrane	5
GOTERM_MF_ALL	GO:0030246~carbohydrate binding	10
GOTERM_BP_ALL	GO:0044248~cellular catabolic process	37
GOTERM_MF_ALL	GO:0004520~endodeoxyribonuclease activity	2
GOTERM_MF_ALL	GO:0070001~aspartic-type peptidase activity	5
GOTERM_MF_ALL	GO:0004190~aspartic-type endopeptidase activity	5
GOTERM_MF_ALL	GO:0003755~peptidyl-prolyl cis-trans isomerase activity	4
GOTERM_BP_ALL	GO:0006820~anion transport	5
GOTERM_CC_ALL	GO:0044437~vacuolar part	5
GOTERM_MF_ALL	GO:0016896~exoribonuclease activity, producing 5'-phosp	3
GOTERM_MF_ALL	GO:0004532~exoribonuclease activity	3
GOTERM_BP_ALL	GO:0008284~positive regulation of cell proliferation	2
GOTERM_MF_ALL	GO:0016859~cis-trans isomerase activity	4
GOTERM_MF_ALL	GO:0004540~ribonuclease activity	5

GOTERM_BP_ALL	GO:0009765~photosynthesis, light harvesting	3
GOTERM_MF_ALL	GO:0004536~deoxyribonuclease activity	2
GOTERM_MF_ALL	GO:0016796~exonuclease activity, active with either ribo-	3
GOTERM_CC_ALL	GO:0009527~plastid outer membrane	3
GOTERM_BP_ALL	GO:0048518~positive regulation of biological process	10
GOTERM_BP_ALL	GO:0006633~fatty acid biosynthetic process	8
GOTERM_BP_ALL	GO:0042364~water-soluble vitamin biosynthetic process	4
GOTERM_BP_ALL	GO:0043193~positive regulation of gene-specific transcrip	2
GOTERM_BP_ALL	GO:0010311~lateral root formation	2
GOTERM_BP_ALL	GO:0009696~salicylic acid metabolic process	2
GOTERM_BP_ALL	GO:0043647~inositol phosphate metabolic process	2
GOTERM_BP_ALL	GO:0032870~cellular response to hormone stimulus	18
GOTERM_BP_ALL	GO:0009755~hormone-mediated signaling	18
GOTERM_BP_ALL	GO:0032502~developmental process	66
GOTERM_BP_ALL	GO:0016137~glycoside metabolic process	5
GOTERM_BP_ALL	GO:0006576~biogenic amine metabolic process	4
GOTERM_BP_ALL	GO:0044249~cellular biosynthetic process	145
GOTERM_BP_ALL	GO:0006098~pentose-phosphate shunt	3
GOTERM_MF_ALL	GO:0003899~DNA-directed RNA polymerase activity	4
GOTERM_BP_ALL	GO:0005986~sucrose biosynthetic process	2
GOTERM_BP_ALL	GO:0010089~xylem histogenesis	2
GOTERM_BP_ALL	GO:0006144~purine base metabolic process	2
GOTERM_BP_ALL	GO:0009064~glutamine family amino acid metabolic proc	4
GOTERM_BP_ALL	GO:0006767~water-soluble vitamin metabolic process	4
GOTERM_CC_ALL	GO:0005643~nuclear pore	3
GOTERM_MF_ALL	GO:0015293~symporter activity	7
GOTERM_MF_ALL	GO:0008889~glycerophosphodiester phosphodiesterase ac	2
GOTERM_MF_ALL	GO:0031559~oxidosqualene cyclase activity	2
GOTERM_BP_ALL	GO:0060255~regulation of macromolecule metabolic proc	84
GOTERM_BP_ALL	GO:0006643~membrane lipid metabolic process	3
GOTERM_BP_ALL	GO:0046148~pigment biosynthetic process	5
GOTERM_BP_ALL	GO:0019222~regulation of metabolic process	87
GOTERM_MF_ALL	GO:0042623~ATPase activity, coupled	14
GOTERM_BP_ALL	GO:0016138~glycoside biosynthetic process	4
GOTERM_BP_ALL	GO:0010015~root morphogenesis	5
GOTERM_BP_ALL	GO:0009653~anatomical structure morphogenesis	19
GOTERM_MF_ALL	GO:0008135~translation factor activity, nucleic acid bindi	7
GOTERM_MF_ALL	GO:0005199~structural constituent of cell wall	3
GOTERM_BP_ALL	GO:0006952~defense response	39
GOTERM_BP_ALL	GO:0051173~positive regulation of nitrogen compound me	4
GOTERM_BP_ALL	GO:0045935~positive regulation of nucleobase, nucleoside	4
GOTERM_MF_ALL	GO:0030599~pectinesterase activity	7
GOTERM_BP_ALL	GO:0007275~multicellular organismal development	60
GOTERM_BP_ALL	GO:0010033~response to organic substance	44
GOTERM_CC_ALL	GO:0080008~CUL4 RING ubiquitin ligase complex	6
GOTERM_BP_ALL	GO:0006739~NADP metabolic process	3
GOTERM_BP_ALL	GO:0032501~multicellular organismal process	63

GOTERM_BP_ALL	GO:0009734~auxin mediated signaling pathway	6
GOTERM_CC_ALL	GO:0015629~actin cytoskeleton	3
GOTERM_BP_ALL	GO:0045454~cell redox homeostasis	7
GOTERM_BP_ALL	GO:0009625~response to insect	2
GOTERM_BP_ALL	GO:0007154~cell communication	8
GOTERM_BP_ALL	GO:0048856~anatomical structure development	49
GOTERM_BP_ALL	GO:0044275~cellular carbohydrate catabolic process	7
GOTERM_BP_ALL	GO:0009615~response to virus	3
GOTERM_CC_ALL	GO:0046930~pore complex	3
GOTERM_MF_ALL	GO:0034062~RNA polymerase activity	4
GOTERM_BP_ALL	GO:0009723~response to ethylene stimulus	11
GOTERM_MF_ALL	GO:0015294~solute:cation symporter activity	6
GOTERM_BP_ALL	GO:0009607~response to biotic stimulus	33
GOTERM_BP_ALL	GO:0042401~biogenic amine biosynthetic process	3
GOTERM_CC_ALL	GO:0009505~plant-type cell wall	11
GOTERM_BP_ALL	GO:0000059~protein import into nucleus, docking	2
GOTERM_BP_ALL	GO:0051640~organelle localization	2
GOTERM_BP_ALL	GO:0042981~regulation of apoptosis	2
GOTERM_BP_ALL	GO:0008360~regulation of cell shape	2
GOTERM_BP_ALL	GO:0044247~cellular polysaccharide catabolic process	2
GOTERM_BP_ALL	GO:0046112~nucleobase biosynthetic process	2
GOTERM_BP_ALL	GO:0009880~embryonic pattern specification	2
GOTERM_MF_ALL	GO:0043492~ATPase activity, coupled to movement of su	8
GOTERM_MF_ALL	GO:0042626~ATPase activity, coupled to transmembrane	8
GOTERM_BP_ALL	GO:0006139~nucleobase, nucleoside, nucleotide and nucle	90
GOTERM_MF_ALL	GO:0015295~solute:hydrogen symporter activity	5
GOTERM_MF_ALL	GO:0005402~cation:sugar symporter activity	5
GOTERM_MF_ALL	GO:0005351~sugar:hydrogen symporter activity	5
GOTERM_MF_ALL	GO:0005315~inorganic phosphate transmembrane transpor	2
GOTERM_MF_ALL	GO:0016903~oxidoreductase activity, acting on the aldehy	4
GOTERM_MF_ALL	GO:0003682~chromatin binding	3
GOTERM_MF_ALL	GO:0016597~amino acid binding	3
GOTERM_MF_ALL	GO:0004806~triacylglycerol lipase activity	3
GOTERM_BP_ALL	GO:0032989~cellular component morphogenesis	10
GOTERM_BP_ALL	GO:0009056~catabolic process	48
GOTERM_BP_ALL	GO:0006606~protein import into nucleus	3
GOTERM_BP_ALL	GO:0051169~nuclear transport	4
GOTERM_BP_ALL	GO:0006913~nucleocytoplasmic transport	4
GOTERM_MF_ALL	GO:0005275~amine transmembrane transporter activity	4
GOTERM_BP_ALL	GO:0006364~rRNA processing	6
GOTERM_BP_ALL	GO:0016072~rRNA metabolic process	6
GOTERM_MF_ALL	GO:0043176~amine binding	3
GOTERM_BP_ALL	GO:0042538~hyperosmotic salinity response	3
GOTERM_BP_ALL	GO:0051170~nuclear import	3
GOTERM_BP_ALL	GO:0009807~lignan biosynthetic process	2
GOTERM_BP_ALL	GO:0032583~regulation of gene-specific transcription	2
GOTERM_BP_ALL	GO:0009556~microsporogenesis	2

GOTERM_BP_ALL	GO:0006525~arginine metabolic process	2
GOTERM_BP_ALL	GO:0009806~lignan metabolic process	2
GOTERM_BP_ALL	GO:0010604~positive regulation of macromolecule metabo	4
GOTERM_BP_ALL	GO:0044237~cellular metabolic process	274
GOTERM_CC_ALL	GO:0005622~intracellular	343
GOTERM_MF_ALL	GO:0008134~transcription factor binding	3
GOTERM_MF_ALL	GO:0016787~hydrolase activity	106
GOTERM_MF_ALL	GO:0016820~hydrolase activity, acting on acid anhydrides	8
GOTERM_MF_ALL	GO:0022804~active transmembrane transporter activity	22
GOTERM_BP_ALL	GO:0009058~biosynthetic process	149
GOTERM_BP_ALL	GO:0009110~vitamin biosynthetic process	4
GOTERM_MF_ALL	GO:0008408~3'-5' exonuclease activity	3
GOTERM_BP_ALL	GO:0051762~sesquiterpene biosynthetic process	2
GOTERM_BP_ALL	GO:0030705~cytoskeleton-dependent intracellular transpo	2
GOTERM_BP_ALL	GO:0016106~sesquiterpenoid biosynthetic process	2
GOTERM_BP_ALL	GO:0005985~sucrose metabolic process	2
GOTERM_BP_ALL	GO:0006536~glutamate metabolic process	2
GOTERM_BP_ALL	GO:0030048~actin filament-based movement	2
GOTERM_BP_ALL	GO:0022610~biological adhesion	2
GOTERM_BP_ALL	GO:0007155~cell adhesion	2
GOTERM_MF_ALL	GO:0008168~methyltransferase activity	10
GOTERM_BP_ALL	GO:0034504~protein localization in nucleus	3
GOTERM_BP_ALL	GO:0016143~S-glycoside metabolic process	3
GOTERM_BP_ALL	GO:0019757~glycosinolate metabolic process	3
GOTERM_BP_ALL	GO:0046496~nicotinamide nucleotide metabolic process	3
GOTERM_BP_ALL	GO:0006769~nicotinamide metabolic process	3
GOTERM_BP_ALL	GO:0019760~glucosinolate metabolic process	3
GOTERM_BP_ALL	GO:0003006~reproductive developmental process	30
GOTERM_MF_ALL	GO:0016741~transferase activity, transferring one-carbon	10
GOTERM_BP_ALL	GO:0005996~monosaccharide metabolic process	8
GOTERM_MF_ALL	GO:0016667~oxidoreductase activity, acting on sulfur gro	5
GOTERM_BP_ALL	GO:0009809~lignin biosynthetic process	3
GOTERM_BP_ALL	GO:0042440~pigment metabolic process	5
GOTERM_BP_ALL	GO:0010228~vegetative to reproductive phase transition	4
GOTERM_BP_ALL	GO:0046688~response to copper ion	2
GOTERM_BP_ALL	GO:0019319~hexose biosynthetic process	2
GOTERM_BP_ALL	GO:0045893~positive regulation of transcription, DNA-de	2
GOTERM_BP_ALL	GO:0048236~plant-type spore development	2
GOTERM_BP_ALL	GO:0009269~response to desiccation	2
GOTERM_BP_ALL	GO:0006595~polyamine metabolic process	2
GOTERM_MF_ALL	GO:0046912~transferase activity, transferring acyl groups,	2
GOTERM_MF_ALL	GO:0015114~phosphate transmembrane transporter activit	2
GOTERM_CC_ALL	GO:0016459~myosin complex	2
GOTERM_CC_ALL	GO:0044424~intracellular part	330
GOTERM_BP_ALL	GO:0006766~vitamin metabolic process	4
GOTERM_CC_ALL	GO:0019898~extrinsic to membrane	9
GOTERM_MF_ALL	GO:0051119~sugar transmembrane transporter activity	5

GOTERM_BP_ALL	GO:0048608~reproductive structure development	27
GOTERM_CC_ALL	GO:0009941~chloroplast envelope	16
GOTERM_BP_ALL	GO:0009112~nucleobase metabolic process	2
GOTERM_BP_ALL	GO:0046364~monosaccharide biosynthetic process	2
GOTERM_BP_ALL	GO:0010224~response to UV-B	3
GOTERM_BP_ALL	GO:0000160~two-component signal transduction system (	9
GOTERM_BP_ALL	GO:0009553~embryo sac development	4
GOTERM_MF_ALL	GO:0042802~identical protein binding	7
GOTERM_BP_ALL	GO:0007047~cell wall organization	11
GOTERM_BP_ALL	GO:0019362~pyridine nucleotide metabolic process	3
GOTERM_BP_ALL	GO:0021700~developmental maturation	3
GOTERM_BP_ALL	GO:0009914~hormone transport	3
GOTERM_BP_ALL	GO:0008152~metabolic process	345
GOTERM_MF_ALL	GO:0022857~transmembrane transporter activity	34
GOTERM_BP_ALL	GO:0048513~organ development	24
GOTERM_BP_ALL	GO:0009733~response to auxin stimulus	13
GOTERM_BP_ALL	GO:0048731~system development	24
GOTERM_BP_ALL	GO:0048440~carpel development	3
GOTERM_BP_ALL	GO:0043603~cellular amide metabolic process	3
GOTERM_BP_ALL	GO:0016114~terpenoid biosynthetic process	4
GOTERM_MF_ALL	GO:0008026~ATP-dependent helicase activity	5
GOTERM_MF_ALL	GO:0070035~purine NTP-dependent helicase activity	5
GOTERM_MF_ALL	GO:0046943~carboxylic acid transmembrane transporter a	4
GOTERM_MF_ALL	GO:0005342~organic acid transmembrane transporter activ	4
GOTERM_MF_ALL	GO:0016832~aldehyde-lyase activity	2
GOTERM_BP_ALL	GO:0022414~reproductive process	32
GOTERM_BP_ALL	GO:0050832~defense response to fungus	15
GOTERM_BP_ALL	GO:0006071~glycerol metabolic process	2
GOTERM_BP_ALL	GO:0022604~regulation of cell morphogenesis	2
GOTERM_MF_ALL	GO:0016746~transferase activity, transferring acyl groups	11
GOTERM_MF_ALL	GO:0015405~P-P-bond-hydrolysis-driven transmembrane t	9
GOTERM_MF_ALL	GO:0015399~primary active transmembrane transporter ac	9
GOTERM_BP_ALL	GO:0048364~root development	8
GOTERM_BP_ALL	GO:0022622~root system development	8
GOTERM_MF_ALL	GO:0016866~intramolecular transferase activity	3
GOTERM_MF_ALL	GO:0016709~oxidoreductase activity, acting on paired don	3
GOTERM_BP_ALL	GO:0009735~response to cytokinin stimulus	4
GOTERM_BP_ALL	GO:0031325~positive regulation of cellular metabolic proc	4
GOTERM_BP_ALL	GO:0048527~lateral root development	3
GOTERM_MF_ALL	GO:0022891~substrate-specific transmembrane transporter	27
GOTERM_CC_ALL	GO:0005777~peroxisome	7
GOTERM_CC_ALL	GO:0042579~microbody	7
GOTERM_BP_ALL	GO:0009893~positive regulation of metabolic process	4
GOTERM_CC_ALL	GO:0005788~endoplasmic reticulum lumen	2
GOTERM_BP_ALL	GO:0019400~alditol metabolic process	2
GOTERM_MF_ALL	GO:0003735~structural constituent of ribosome	14
GOTERM_MF_ALL	GO:0015144~carbohydrate transmembrane transporter acti	5

GOTERM_BP_ALL	GO:0022607~cellular component assembly	12
GOTERM_BP_ALL	GO:0034641~cellular nitrogen compound metabolic proce	103
GOTERM_MF_ALL	GO:0016564~transcription repressor activity	3
GOTERM_BP_ALL	GO:0046395~carboxylic acid catabolic process	4
GOTERM_BP_ALL	GO:0016054~organic acid catabolic process	4
GOTERM_BP_ALL	GO:0006714~sesquiterpenoid metabolic process	2
GOTERM_BP_ALL	GO:0010101~post-embryonic root morphogenesis	2
GOTERM_BP_ALL	GO:0010102~lateral root morphogenesis	2
GOTERM_BP_ALL	GO:0051761~sesquiterpene metabolic process	2
GOTERM_BP_ALL	GO:0009620~response to fungus	16
GOTERM_MF_ALL	GO:0017111~nucleoside-triphosphatase activity	27
GOTERM_MF_ALL	GO:0016645~oxidoreductase activity, acting on the CH-N	2
GOTERM_BP_ALL	GO:0016052~carbohydrate catabolic process	8
GOTERM_BP_ALL	GO:0009309~amine biosynthetic process	8
GOTERM_CC_ALL	GO:0009526~plastid envelope	16
GOTERM_BP_ALL	GO:0006825~copper ion transport	2
GOTERM_BP_ALL	GO:0009637~response to blue light	3
GOTERM_BP_ALL	GO:0045941~positive regulation of transcription	3
GOTERM_BP_ALL	GO:0048467~gynoecium development	3
GOTERM_BP_ALL	GO:0009791~post-embryonic development	30
GOTERM_BP_ALL	GO:0000003~reproduction	32
GOTERM_BP_ALL	GO:0006869~lipid transport	6
GOTERM_BP_ALL	GO:0048610~reproductive cellular process	5
GOTERM_BP_ALL	GO:0048528~post-embryonic root development	3
GOTERM_BP_ALL	GO:0006733~oxidoreduction coenzyme metabolic process	3
GOTERM_BP_ALL	GO:0010628~positive regulation of gene expression	3
GOTERM_BP_ALL	GO:0048653~anther development	2
GOTERM_BP_ALL	GO:0009658~chloroplast organization	3
GOTERM_BP_ALL	GO:0006807~nitrogen compound metabolic process	105
GOTERM_MF_ALL	GO:0005215~transporter activity	42
GOTERM_CC_ALL	GO:0010287~plastoglobule	3
GOTERM_BP_ALL	GO:0010817~regulation of hormone levels	5
GOTERM_BP_ALL	GO:0009739~response to gibberellin stimulus	5
GOTERM_BP_ALL	GO:0009225~nucleotide-sugar metabolic process	2
GOTERM_BP_ALL	GO:0009084~glutamine family amino acid biosynthetic pr	2
GOTERM_BP_ALL	GO:0042127~regulation of cell proliferation	2
GOTERM_BP_ALL	GO:0046246~terpene biosynthetic process	2
GOTERM_MF_ALL	GO:0015103~inorganic anion transmembrane transporter a	3
GOTERM_MF_ALL	GO:0031072~heat shock protein binding	5
GOTERM_MF_ALL	GO:0005198~structural molecule activity	18
GOTERM_CC_ALL	GO:0033178~proton-transporting two-sector ATPase comp	2
GOTERM_BP_ALL	GO:0009820~alkaloid metabolic process	3
GOTERM_BP_ALL	GO:0043067~regulation of programmed cell death	2
GOTERM_MF_ALL	GO:0022892~substrate-specific transporter activity	30
GOTERM_CC_ALL	GO:0005783~endoplasmic reticulum	15
GOTERM_CC_ALL	GO:0031461~cullin-RING ubiquitin ligase complex	6
GOTERM_BP_ALL	GO:0043623~cellular protein complex assembly	5

GOTERM_BP_ALL	GO:0051707~response to other organism	28
GOTERM_CC_ALL	GO:0031968~organelle outer membrane	3
GOTERM_MF_ALL	GO:0015171~amino acid transmembrane transporter activi	3
GOTERM_BP_ALL	GO:0016144~S-glycoside biosynthetic process	2
GOTERM_BP_ALL	GO:0019758~glycosinolate biosynthetic process	2
GOTERM_BP_ALL	GO:0045165~cell fate commitment	2
GOTERM_BP_ALL	GO:0019761~glucosinolate biosynthetic process	2
GOTERM_MF_ALL	GO:0005525~GTP binding	9
GOTERM_CC_ALL	GO:0033279~ribosomal subunit	10
GOTERM_MF_ALL	GO:0046961~proton-transporting ATPase activity, rotation	2
GOTERM_BP_ALL	GO:0010557~positive regulation of macromolecule biosyn	3
GOTERM_CC_ALL	GO:0046658~anchored to plasma membrane	3
GOTERM_CC_ALL	GO:0005635~nuclear envelope	3
GOTERM_CC_ALL	GO:0015935~small ribosomal subunit	5
GOTERM_BP_ALL	GO:0046483~heterocycle metabolic process	15
GOTERM_MF_ALL	GO:0016462~pyrophosphatase activity	27
GOTERM_BP_ALL	GO:0019318~hexose metabolic process	6
GOTERM_BP_ALL	GO:0006091~generation of precursor metabolites and ener	14
GOTERM_MF_ALL	GO:0045735~nutrient reservoir activity	3
GOTERM_BP_ALL	GO:0009911~positive regulation of flower development	2
GOTERM_BP_ALL	GO:0010941~regulation of cell death	2
GOTERM_BP_ALL	GO:0065008~regulation of biological quality	22
GOTERM_MF_ALL	GO:0004553~hydrolase activity, hydrolyzing O-glycosyl c	14
GOTERM_MF_ALL	GO:0016298~lipase activity	5
GOTERM_BP_ALL	GO:0009808~lignin metabolic process	3
GOTERM_BP_ALL	GO:0010087~phloem or xylem histogenesis	2
GOTERM_BP_ALL	GO:0006721~terpenoid metabolic process	4
GOTERM_MF_ALL	GO:0008171~O-methyltransferase activity	2
GOTERM_MF_ALL	GO:0004437~inositol or phosphatidylinositol phosphatase	2
GOTERM_CC_ALL	GO:0019867~outer membrane	3
GOTERM_BP_ALL	GO:0006865~amino acid transport	3
GOTERM_BP_ALL	GO:0006732~coenzyme metabolic process	7
GOTERM_BP_ALL	GO:0019321~pentose metabolic process	2
GOTERM_BP_ALL	GO:0007005~mitochondrion organization	2
GOTERM_MF_ALL	GO:0016818~hydrolase activity, acting on acid anhydrides	27
GOTERM_MF_ALL	GO:0008509~anion transmembrane transporter activity	4
GOTERM_BP_ALL	GO:0015837~amine transport	3
GOTERM_MF_ALL	GO:0042578~phosphoric ester hydrolase activity	12
GOTERM_BP_ALL	GO:0010876~lipid localization	6
GOTERM_CC_ALL	GO:0031969~chloroplast membrane	3
GOTERM_BP_ALL	GO:0009062~fatty acid catabolic process	2
GOTERM_BP_ALL	GO:0048481~ovule development	2
GOTERM_BP_ALL	GO:0009891~positive regulation of biosynthetic process	3
GOTERM_BP_ALL	GO:0009932~cell tip growth	3
GOTERM_BP_ALL	GO:0031328~positive regulation of cellular biosynthetic p	3
GOTERM_MF_ALL	GO:0003712~transcription cofactor activity	2
GOTERM_MF_ALL	GO:0016747~transferase activity, transferring acyl groups	9

GOTERM_MF_ALL	GO:0016817~hydrolase activity, acting on acid anhydrides	27
GOTERM_MF_ALL	GO:0003779~actin binding	3
GOTERM_MF_ALL	GO:0016791~phosphatase activity	10
GOTERM_MF_ALL	GO:0017171~serine hydrolase activity	6
GOTERM_MF_ALL	GO:0008236~serine-type peptidase activity	6
GOTERM_MF_ALL	GO:0016627~oxidoreductase activity, acting on the CH-C	3
GOTERM_BP_ALL	GO:0008283~cell proliferation	2
GOTERM_BP_ALL	GO:0046351~disaccharide biosynthetic process	2
GOTERM_BP_ALL	GO:0005982~starch metabolic process	2
GOTERM_BP_ALL	GO:0009081~branched chain family amino acid metabolic	2
GOTERM_CC_ALL	GO:0005856~cytoskeleton	8
GOTERM_MF_ALL	GO:0004175~endopeptidase activity	10
GOTERM_CC_ALL	GO:0005737~cytoplasm	230
GOTERM_MF_ALL	GO:0016779~nucleotidyltransferase activity	6
GOTERM_BP_ALL	GO:0044092~negative regulation of molecular function	3
GOTERM_BP_ALL	GO:0001906~cell killing	10
GOTERM_BP_ALL	GO:0031640~killing of cells of another organism	10
GOTERM_BP_ALL	GO:0009639~response to red or far red light	6
GOTERM_BP_ALL	GO:0009873~ethylene mediated signaling pathway	6
GOTERM_CC_ALL	GO:0044429~mitochondrial part	10
GOTERM_MF_ALL	GO:0004857~enzyme inhibitor activity	6
GOTERM_BP_ALL	GO:0009725~response to hormone stimulus	30
GOTERM_MF_ALL	GO:0008092~cytoskeletal protein binding	4
GOTERM_MF_ALL	GO:0046982~protein heterodimerization activity	2
GOTERM_MF_ALL	GO:0032561~guanyl ribonucleotide binding	9
GOTERM_BP_ALL	GO:0022900~electron transport chain	6
GOTERM_BP_ALL	GO:0000271~polysaccharide biosynthetic process	4
GOTERM_MF_ALL	GO:0016798~hydrolase activity, acting on glycosyl bonds	14
GOTERM_BP_ALL	GO:0048469~cell maturation	2
GOTERM_BP_ALL	GO:0048765~root hair cell differentiation	2
GOTERM_BP_ALL	GO:0048764~trichoblast maturation	2
GOTERM_BP_ALL	GO:0048582~positive regulation of post-embryonic develo	2
GOTERM_BP_ALL	GO:0042214~terpene metabolic process	2
GOTERM_CC_ALL	GO:0045259~proton-transporting ATP synthase complex	2
GOTERM_MF_ALL	GO:0019001~guanyl nucleotide binding	9
GOTERM_BP_ALL	GO:0000272~polysaccharide catabolic process	3
GOTERM_BP_ALL	GO:0030029~actin filament-based process	3
GOTERM_BP_ALL	GO:0006413~translational initiation	3
GOTERM_BP_ALL	GO:0048588~developmental cell growth	3
GOTERM_BP_ALL	GO:0009886~post-embryonic morphogenesis	3
GOTERM_CC_ALL	GO:0022627~cytosolic small ribosomal subunit	4
GOTERM_BP_ALL	GO:0009312~oligosaccharide biosynthetic process	2
GOTERM_BP_ALL	GO:0009250~glucan biosynthetic process	3
GOTERM_BP_ALL	GO:0033365~protein localization in organelle	3
GOTERM_MF_ALL	GO:0019200~carbohydrate kinase activity	2
GOTERM_BP_ALL	GO:0009251~glucan catabolic process	2
GOTERM_CC_ALL	GO:0044421~extracellular region part	2

GOTERM_BP_ALL	GO:0015849~organic acid transport	3
GOTERM_BP_ALL	GO:0046942~carboxylic acid transport	3
GOTERM_MF_ALL	GO:0005488~binding	398
GOTERM_MF_ALL	GO:0000156~two-component response regulator activity	2
GOTERM_MF_ALL	GO:0070279~vitamin B6 binding	4
GOTERM_MF_ALL	GO:0030170~pyridoxal phosphate binding	4
GOTERM_BP_ALL	GO:0010054~trichoblast differentiation	2
GOTERM_BP_ALL	GO:0008299~isoprenoid biosynthetic process	4
GOTERM_BP_ALL	GO:0009664~plant-type cell wall organization	3
GOTERM_CC_ALL	GO:0044430~cytoskeletal part	6
GOTERM_BP_ALL	GO:0008652~cellular amino acid biosynthetic process	6
GOTERM_BP_ALL	GO:0017038~protein import	3
GOTERM_BP_ALL	GO:0006006~glucose metabolic process	4
GOTERM_BP_ALL	GO:0046164~alcohol catabolic process	4
GOTERM_BP_ALL	GO:0009629~response to gravity	2
GOTERM_BP_ALL	GO:0070271~protein complex biogenesis	6
GOTERM_BP_ALL	GO:0006461~protein complex assembly	6
GOTERM_MF_ALL	GO:0005529~sugar binding	4
GOTERM_CC_ALL	GO:0045271~respiratory chain complex I	2
GOTERM_CC_ALL	GO:0030964~NADH dehydrogenase complex	2
GOTERM_BP_ALL	GO:0030384~phosphoinositide metabolic process	2
GOTERM_BP_ALL	GO:0030258~lipid modification	2
GOTERM_BP_ALL	GO:0005984~disaccharide metabolic process	2
GOTERM_BP_ALL	GO:0051094~positive regulation of developmental process	2
GOTERM_BP_ALL	GO:0022603~regulation of anatomical structure morphoge	2
GOTERM_BP_ALL	GO:0034470~ncRNA processing	6
GOTERM_MF_ALL	GO:0008757~S-adenosylmethionine-dependent methyltran	4
GOTERM_MF_ALL	GO:0016763~transferase activity, transferring pentosyl gro	2
GOTERM_MF_ALL	GO:0004519~endonuclease activity	3
GOTERM_BP_ALL	GO:0006694~steroid biosynthetic process	2
GOTERM_BP_ALL	GO:0010218~response to far red light	2
GOTERM_BP_ALL	GO:0009741~response to brassinosteroid stimulus	2
GOTERM_BP_ALL	GO:0009073~aromatic amino acid family biosynthetic pro	2
GOTERM_BP_ALL	GO:0046356~acetyl-CoA catabolic process	2
GOTERM_BP_ALL	GO:0009063~cellular amino acid catabolic process	2
GOTERM_BP_ALL	GO:0006099~tricarboxylic acid cycle	2
GOTERM_BP_ALL	GO:0046417~chorismate metabolic process	2
GOTERM_MF_ALL	GO:0015291~secondary active transmembrane transporter	9
GOTERM_MF_ALL	GO:0050660~FAD binding	5
GOTERM_BP_ALL	GO:0060560~developmental growth involved in morphoge	5
GOTERM_BP_ALL	GO:0009826~unidimensional cell growth	5
GOTERM_BP_ALL	GO:0051704~multi-organism process	31
GOTERM_CC_ALL	GO:0042170~plastid membrane	3
GOTERM_BP_ALL	GO:0019684~photosynthesis, light reaction	3
GOTERM_CC_ALL	GO:0044445~cytosolic part	8
GOTERM_CC_ALL	GO:0043229~intracellular organelle	288
GOTERM_CC_ALL	GO:0043226~organelle	288

GOTERM_BP_ALL	GO:0009926~auxin polar transport	2
GOTERM_BP_ALL	GO:0009640~photomorphogenesis	2
GOTERM_BP_ALL	GO:0010053~root epidermal cell differentiation	2
GOTERM_MF_ALL	GO:0019829~cation-transporting ATPase activity	2
GOTERM_BP_ALL	GO:0009310~amine catabolic process	2
GOTERM_CC_ALL	GO:0043231~intracellular membrane-bounded organelle	275
GOTERM_CC_ALL	GO:0031967~organelle envelope	22
GOTERM_BP_ALL	GO:0019725~cellular homeostasis	7
GOTERM_CC_ALL	GO:0000151~ubiquitin ligase complex	7
GOTERM_CC_ALL	GO:0044446~intracellular organelle part	82
GOTERM_CC_ALL	GO:0044432~endoplasmic reticulum part	3
GOTERM_CC_ALL	GO:0044422~organelle part	82
GOTERM_BP_ALL	GO:0045333~cellular respiration	3
GOTERM_MF_ALL	GO:0003676~nucleic acid binding	131
GOTERM_CC_ALL	GO:0043227~membrane-bounded organelle	275
GOTERM_BP_ALL	GO:0009109~coenzyme catabolic process	2
GOTERM_MF_ALL	GO:0008289~lipid binding	7
GOTERM_MF_ALL	GO:0060589~nucleoside-triphosphatase regulator activity	3
GOTERM_CC_ALL	GO:0031975~envelope	22
GOTERM_BP_ALL	GO:0009736~cytokinin mediated signaling	2
GOTERM_BP_ALL	GO:0009582~detection of abiotic stimulus	2
GOTERM_BP_ALL	GO:0009606~tropism	2
GOTERM_BP_ALL	GO:0048609~reproductive process in a multicellular organ	2
GOTERM_BP_ALL	GO:0009060~aerobic respiration	2
GOTERM_BP_ALL	GO:0009409~response to cold	7
GOTERM_BP_ALL	GO:0006119~oxidative phosphorylation	3
GOTERM_MF_ALL	GO:0008565~protein transporter activity	4
GOTERM_BP_ALL	GO:0055086~nucleobase, nucleoside and nucleotide metab	8
GOTERM_BP_ALL	GO:0015931~nucleobase, nucleoside, nucleotide and nucle	2
GOTERM_MF_ALL	GO:0003774~motor activity	3
GOTERM_CC_ALL	GO:0015934~large ribosomal subunit	5
GOTERM_MF_ALL	GO:0004197~cysteine-type endopeptidase activity	2
GOTERM_MF_ALL	GO:0004386~helicase activity	5
GOTERM_MF_ALL	GO:0005516~calmodulin binding	5
GOTERM_BP_ALL	GO:0007264~small GTPase mediated signal transduction	3
GOTERM_BP_ALL	GO:0009657~plastid organization	3
GOTERM_MF_ALL	GO:0003924~GTPase activity	3
GOTERM_BP_ALL	GO:0006396~RNA processing	13
GOTERM_BP_ALL	GO:0019637~organophosphate metabolic process	4
GOTERM_BP_ALL	GO:0009311~oligosaccharide metabolic process	2
GOTERM_BP_ALL	GO:0009860~pollen tube growth	2
GOTERM_MF_ALL	GO:0008483~transaminase activity	2
GOTERM_BP_ALL	GO:0007242~intracellular signaling cascade	24
GOTERM_CC_ALL	GO:0022625~cytosolic large ribosomal subunit	4
GOTERM_BP_ALL	GO:0032504~multicellular organism reproduction	2
GOTERM_BP_ALL	GO:0018130~heterocycle biosynthetic process	4
GOTERM_MF_ALL	GO:0004185~serine-type carboxypeptidase activity	2

GOTERM_MF_ALL	GO:0070008~serine-type exopeptidase activity	2
GOTERM_BP_ALL	GO:0015980~energy derivation by oxidation of organic co	3
GOTERM_BP_ALL	GO:0016042~lipid catabolic process	6
GOTERM_CC_ALL	GO:0070469~respiratory chain	3
GOTERM_MF_ALL	GO:0004252~serine-type endopeptidase activity	3
GOTERM_BP_ALL	GO:0009581~detection of external stimulus	2
GOTERM_BP_ALL	GO:0009832~plant-type cell wall biogenesis	2
GOTERM_BP_ALL	GO:0006720~isoprenoid metabolic process	4
GOTERM_BP_ALL	GO:0006790~sulfur metabolic process	5
GOTERM_BP_ALL	GO:0034622~cellular macromolecular complex assembly	6
GOTERM_BP_ALL	GO:0042592~homeostatic process	8
GOTERM_BP_ALL	GO:0009617~response to bacterium	7
GOTERM_BP_ALL	GO:0051186~cofactor metabolic process	8
GOTERM_BP_ALL	GO:0006084~acetyl-CoA metabolic process	2
GOTERM_BP_ALL	GO:0044106~cellular amine metabolic process	12
GOTERM_CC_ALL	GO:0031226~intrinsic to plasma membrane	3
GOTERM_BP_ALL	GO:0010114~response to red light	2
GOTERM_BP_ALL	GO:0015979~photosynthesis	5
GOTERM_MF_ALL	GO:0042803~protein homodimerization activity	2
GOTERM_BP_ALL	GO:0000904~cell morphogenesis involved in differentiatio	3
GOTERM_BP_ALL	GO:0044242~cellular lipid catabolic process	2
GOTERM_MF_ALL	GO:0015078~hydrogen ion transmembrane transporter acti	4
GOTERM_MF_ALL	GO:0050662~coenzyme binding	10
GOTERM_BP_ALL	GO:0009308~amine metabolic process	14
GOTERM_BP_ALL	GO:0016049~cell growth	6
GOTERM_BP_ALL	GO:0055085~transmembrane transport	6
GOTERM_MF_ALL	GO:0008081~phosphoric diester hydrolase activity	2
GOTERM_BP_ALL	GO:0009737~response to abscisic acid stimulus	8
GOTERM_BP_ALL	GO:0051187~cofactor catabolic process	2
GOTERM_MF_ALL	GO:0070011~peptidase activity, acting on L-amino acid pe	15
GOTERM_MF_ALL	GO:0004180~carboxypeptidase activity	2
GOTERM_MF_ALL	GO:0016810~hydrolase activity, acting on carbon-nitrogen	3
GOTERM_BP_ALL	GO:0043413~biopolymer glycosylation	2
GOTERM_BP_ALL	GO:0006650~glycerophospholipid metabolic process	2
GOTERM_BP_ALL	GO:0015986~ATP synthesis coupled proton transport	2
GOTERM_BP_ALL	GO:0070085~glycosylation	2
GOTERM_BP_ALL	GO:0015985~energy coupled proton transport, down electr	2
GOTERM_BP_ALL	GO:0006486~protein amino acid glycosylation	2
GOTERM_BP_ALL	GO:0009101~glycoprotein biosynthetic process	2
GOTERM_BP_ALL	GO:0009072~aromatic amino acid family metabolic proces	2
GOTERM_BP_ALL	GO:0050794~regulation of cellular process	110
GOTERM_BP_ALL	GO:0048589~developmental growth	5
GOTERM_BP_ALL	GO:0033692~cellular polysaccharide biosynthetic process	3
GOTERM_MF_ALL	GO:0008324~cation transmembrane transporter activity	12
GOTERM_BP_ALL	GO:0016043~cellular component organization	38
GOTERM_BP_ALL	GO:0034404~nucleobase, nucleoside and nucleotide biosy	5
GOTERM_BP_ALL	GO:0034654~nucleobase, nucleoside, nucleotide and nucle	5

GOTERM_BP_ALL	GO:0006007~glucose catabolic process	3
GOTERM_BP_ALL	GO:0009116~nucleoside metabolic process	2
GOTERM_CC_ALL	GO:0016469~proton-transporting two-sector ATPase comp	2
GOTERM_MF_ALL	GO:0008233~peptidase activity	16
GOTERM_BP_ALL	GO:0019320~hexose catabolic process	3
GOTERM_BP_ALL	GO:0046365~monosaccharide catabolic process	3
GOTERM_BP_ALL	GO:0051321~meiotic cell cycle	2
GOTERM_MF_ALL	GO:0048037~cofactor binding	14
GOTERM_CC_ALL	GO:0005886~plasma membrane	66
GOTERM_BP_ALL	GO:0044272~sulfur compound biosynthetic process	3
GOTERM_MF_ALL	GO:0022836~gated channel activity	2
GOTERM_MF_ALL	GO:0045330~aspartyl esterase activity	2
GOTERM_CC_ALL	GO:0044459~plasma membrane part	7
GOTERM_MF_ALL	GO:0046914~transition metal ion binding	82
GOTERM_CC_ALL	GO:0005834~heterotrimeric G-protein complex	2
GOTERM_BP_ALL	GO:0009100~glycoprotein metabolic process	2
GOTERM_BP_ALL	GO:0007010~cytoskeleton organization	3
GOTERM_BP_ALL	GO:0009751~response to salicylic acid stimulus	4
GOTERM_BP_ALL	GO:0008361~regulation of cell size	6
GOTERM_BP_ALL	GO:0034621~cellular macromolecular complex subunit or	6
GOTERM_CC_ALL	GO:0019897~extrinsic to plasma membrane	2
GOTERM_BP_ALL	GO:0030243~cellulose metabolic process	2
GOTERM_BP_ALL	GO:0051128~regulation of cellular component organizatio	2
GOTERM_BP_ALL	GO:0019953~sexual reproduction	2
GOTERM_BP_ALL	GO:0044271~nitrogen compound biosynthetic process	14
GOTERM_MF_ALL	GO:0016769~transferase activity, transferring nitrogenous	2
GOTERM_MF_ALL	GO:0030234~enzyme regulator activity	9
GOTERM_BP_ALL	GO:0046486~glycerolipid metabolic process	2
GOTERM_BP_ALL	GO:0034220~ion transmembrane transport	2
GOTERM_BP_ALL	GO:0043094~cellular metabolic compound salvage	2
GOTERM_BP_ALL	GO:0043086~negative regulation of catalytic activity	2
GOTERM_CC_ALL	GO:0015630~microtubule cytoskeleton	4
GOTERM_MF_ALL	GO:0015077~monovalent inorganic cation transmembrane	4
GOTERM_BP_ALL	GO:0008202~steroid metabolic process	2
GOTERM_MF_ALL	GO:0015075~ion transmembrane transporter activity	15
GOTERM_CC_ALL	GO:0005739~mitochondrion	38
GOTERM_BP_ALL	GO:0006753~nucleoside phosphate metabolic process	6
GOTERM_BP_ALL	GO:0009117~nucleotide metabolic process	6
GOTERM_BP_ALL	GO:0051606~detection of stimulus	2
GOTERM_BP_ALL	GO:0065003~macromolecular complex assembly	7
GOTERM_MF_ALL	GO:0016836~hydro-lyase activity	2
GOTERM_BP_ALL	GO:0065009~regulation of molecular function	4
GOTERM_BP_ALL	GO:0006886~intracellular protein transport	7
GOTERM_CC_ALL	GO:0044464~cell part	547
GOTERM_CC_ALL	GO:0005623~cell	547
GOTERM_BP_ALL	GO:0048468~cell development	4
GOTERM_BP_ALL	GO:0032535~regulation of cellular component size	6

GOTERM_BP_ALL	GO:0006520~cellular amino acid metabolic process	10
GOTERM_BP_ALL	GO:0051234~establishment of localization	63
GOTERM_BP_ALL	GO:0042445~hormone metabolic process	2
GOTERM_MF_ALL	GO:0019843~rRNA binding	2
GOTERM_MF_ALL	GO:0016614~oxidoreductase activity, acting on CH-OH gr	4
GOTERM_MF_ALL	GO:0016835~carbon-oxygen lyase activity	3
GOTERM_BP_ALL	GO:0010467~gene expression	92
GOTERM_BP_ALL	GO:0000041~transition metal ion transport	2
GOTERM_BP_ALL	GO:0043648~dicarboxylic acid metabolic process	2
GOTERM_BP_ALL	GO:0007568~aging	2
GOTERM_CC_ALL	GO:0044455~mitochondrial membrane part	2
GOTERM_BP_ALL	GO:0007017~microtubule-based process	3
GOTERM_BP_ALL	GO:0009738~abscisic acid mediated signaling	2
GOTERM_BP_ALL	GO:0006811~ion transport	13
GOTERM_BP_ALL	GO:0006644~phospholipid metabolic process	3
GOTERM_MF_ALL	GO:0017076~purine nucleotide binding	81
GOTERM_BP_ALL	GO:0048868~pollen tube development	2
GOTERM_BP_ALL	GO:0035295~tube development	2
GOTERM_MF_ALL	GO:0016740~transferase activity	85
GOTERM_BP_ALL	GO:0040007~growth	6
GOTERM_MF_ALL	GO:0001882~nucleoside binding	73
GOTERM_BP_ALL	GO:0000902~cell morphogenesis	5
GOTERM_MF_ALL	GO:0042625~ATPase activity, coupled to transmembrane	2
GOTERM_BP_ALL	GO:0006810~transport	62
GOTERM_BP_ALL	GO:0034613~cellular protein localization	7
GOTERM_BP_ALL	GO:0009887~organ morphogenesis	4
GOTERM_BP_ALL	GO:0046907~intracellular transport	10
GOTERM_MF_ALL	GO:0015238~drug transporter activity	2
GOTERM_BP_ALL	GO:0043933~macromolecular complex subunit organizatio	7
GOTERM_BP_ALL	GO:0006818~hydrogen transport	2
GOTERM_BP_ALL	GO:0015992~proton transport	2
GOTERM_BP_ALL	GO:0007165~signal transduction	34
GOTERM_MF_ALL	GO:0032555~purine ribonucleotide binding	75
GOTERM_MF_ALL	GO:0032553~ribonucleotide binding	75
GOTERM_MF_ALL	GO:0001883~purine nucleoside binding	72
GOTERM_MF_ALL	GO:0030554~adenyl nucleotide binding	72
GOTERM_BP_ALL	GO:0009152~purine ribonucleotide biosynthetic process	3
GOTERM_BP_ALL	GO:0009150~purine ribonucleotide metabolic process	3
GOTERM_BP_ALL	GO:0006605~protein targeting	3
GOTERM_BP_ALL	GO:0009856~pollination	3
GOTERM_BP_ALL	GO:0008643~carbohydrate transport	2
GOTERM_BP_ALL	GO:0050793~regulation of developmental process	6
GOTERM_MF_ALL	GO:0005524~ATP binding	66
GOTERM_MF_ALL	GO:0005216~ion channel activity	2
GOTERM_CC_ALL	GO:0044451~nucleoplasm part	3
GOTERM_MF_ALL	GO:0022890~inorganic cation transmembrane transporter	5
GOTERM_BP_ALL	GO:0034660~ncRNA metabolic process	6

GOTERM_BP_ALL	GO:0042545~cell wall modification	3
GOTERM_CC_ALL	GO:0031224~intrinsic to membrane	76
GOTERM_CC_ALL	GO:0031977~thylakoid lumen	2
GOTERM_BP_ALL	GO:0051179~localization	63
GOTERM_MF_ALL	GO:0051540~metal cluster binding	2
GOTERM_MF_ALL	GO:0051536~iron-sulfur cluster binding	2
GOTERM_BP_ALL	GO:0006164~purine nucleotide biosynthetic process	3
GOTERM_MF_ALL	GO:0008238~exopeptidase activity	2
GOTERM_BP_ALL	GO:0070727~cellular macromolecule localization	7
GOTERM_BP_ALL	GO:0010154~fruit development	11
GOTERM_BP_ALL	GO:0042742~defense response to bacterium	4
GOTERM_BP_ALL	GO:0034645~cellular macromolecule biosynthetic process	85
GOTERM_BP_ALL	GO:0009260~ribonucleotide biosynthetic process	3
GOTERM_BP_ALL	GO:0006163~purine nucleotide metabolic process	3
GOTERM_BP_ALL	GO:0050789~regulation of biological process	114
GOTERM_BP_ALL	GO:0044238~primary metabolic process	267
GOTERM_BP_ALL	GO:0051649~establishment of localization in cell	11
GOTERM_MF_ALL	GO:0032559~adenyl ribonucleotide binding	66
GOTERM_BP_ALL	GO:0009108~coenzyme biosynthetic process	2
GOTERM_BP_ALL	GO:0009987~cellular process	328
GOTERM_BP_ALL	GO:0009059~macromolecule biosynthetic process	85
GOTERM_BP_ALL	GO:0009259~ribonucleotide metabolic process	3
GOTERM_CC_ALL	GO:0032991~macromolecular complex	58
GOTERM_MF_ALL	GO:0004888~transmembrane receptor activity	4
GOTERM_BP_ALL	GO:0009909~regulation of flower development	2
GOTERM_MF_ALL	GO:0000166~nucleotide binding	95
GOTERM_CC_ALL	GO:0005654~nucleoplasm	3
GOTERM_BP_ALL	GO:0050790~regulation of catalytic activity	3
GOTERM_MF_ALL	GO:0000287~magnesium ion binding	7
GOTERM_BP_ALL	GO:0010016~shoot morphogenesis	3
GOTERM_MF_ALL	GO:0008234~cysteine-type peptidase activity	3
GOTERM_BP_ALL	GO:0048367~shoot development	6
GOTERM_BP_ALL	GO:0022621~shoot system development	6
GOTERM_MF_ALL	GO:0030145~manganese ion binding	5
GOTERM_BP_ALL	GO:0009888~tissue development	5
GOTERM_BP_ALL	GO:0048827~phyllome development	4
GOTERM_CC_ALL	GO:0009507~chloroplast	90
GOTERM_BP_ALL	GO:0051246~regulation of protein metabolic process	2
GOTERM_BP_ALL	GO:0051188~cofactor biosynthetic process	3
GOTERM_BP_ALL	GO:0009793~embryonic development ending in seed dorm	8
GOTERM_BP_ALL	GO:0006955~immune response	6
GOTERM_MF_ALL	GO:0004842~ubiquitin-protein ligase activity	5
GOTERM_CC_ALL	GO:0016021~integral to membrane	59
GOTERM_BP_ALL	GO:0009790~embryonic development	9
GOTERM_MF_ALL	GO:0005507~copper ion binding	3
GOTERM_BP_ALL	GO:0009965~leaf morphogenesis	2
GOTERM_BP_ALL	GO:0046034~ATP metabolic process	2

GOTERM_BP_ALL	GO:0006754~ATP biosynthetic process	2
GOTERM_BP_ALL	GO:0007166~cell surface receptor linked signal transducti	3
GOTERM_BP_ALL	GO:0006730~one-carbon metabolic process	2
GOTERM_BP_ALL	GO:0007389~pattern specification process	2
GOTERM_BP_ALL	GO:0051641~cellular localization	11
GOTERM_MF_ALL	GO:0008415~acyltransferase activity	4
GOTERM_MF_ALL	GO:0019787~small conjugating protein ligase activity	5
GOTERM_CC_ALL	GO:0009536~plastid	91
GOTERM_BP_ALL	GO:0009913~epidermal cell differentiation	2
GOTERM_BP_ALL	GO:0065007~biological regulation	124
GOTERM_MF_ALL	GO:0043167~ion binding	100
GOTERM_CC_ALL	GO:0005874~microtubule	2
GOTERM_BP_ALL	GO:0009199~ribonucleoside triphosphate metabolic proce	2
GOTERM_BP_ALL	GO:0009145~purine nucleoside triphosphate biosynthetic	2
GOTERM_BP_ALL	GO:0009144~purine nucleoside triphosphate metabolic pro	2
GOTERM_BP_ALL	GO:0009206~purine ribonucleoside triphosphate biosynthe	2
GOTERM_BP_ALL	GO:0009201~ribonucleoside triphosphate biosynthetic pro	2
GOTERM_BP_ALL	GO:0009205~purine ribonucleoside triphosphate metaboli	2
GOTERM_BP_ALL	GO:0002376~immune system process	6
GOTERM_BP_ALL	GO:0009165~nucleotide biosynthetic process	3
GOTERM_BP_ALL	GO:0051301~cell division	3
GOTERM_BP_ALL	GO:0008544~epidermis development	2
GOTERM_BP_ALL	GO:0007398~ectoderm development	2
GOTERM_BP_ALL	GO:0009142~nucleoside triphosphate biosynthetic process	2
GOTERM_BP_ALL	GO:0009141~nucleoside triphosphate metabolic process	2
GOTERM_BP_ALL	GO:0016070~RNA metabolic process	15
GOTERM_CC_ALL	GO:0044444~cytoplasmic part	190
GOTERM_BP_ALL	GO:0006915~apoptosis	3
GOTERM_CC_ALL	GO:0009579~thylakoid	10
GOTERM_BP_ALL	GO:0048316~seed development	9
GOTERM_MF_ALL	GO:0043169~cation binding	99
GOTERM_MF_ALL	GO:0016874~ligase activity	10
GOTERM_CC_ALL	GO:0044425~membrane part	88
GOTERM_CC_ALL	GO:0005743~mitochondrial inner membrane	3
GOTERM_BP_ALL	GO:0045087~innate immune response	5
GOTERM_MF_ALL	GO:0016881~acid-amino acid ligase activity	5
GOTERM_BP_ALL	GO:0006511~ubiquitin-dependent protein catabolic proces	5
GOTERM_BP_ALL	GO:0048366~leaf development	3
GOTERM_BP_ALL	GO:0006996~organelle organization	13
GOTERM_BP_ALL	GO:0007169~transmembrane receptor protein tyrosine kin	2
GOTERM_BP_ALL	GO:0007167~enzyme linked receptor protein signaling pat	2
GOTERM_MF_ALL	GO:0005515~protein binding	101
GOTERM_MF_ALL	GO:0016879~ligase activity, forming carbon-nitrogen bon	6
GOTERM_MF_ALL	GO:0046872~metal ion binding	92
GOTERM_BP_ALL	GO:0009451~RNA modification	2
GOTERM_MF_ALL	GO:0016616~oxidoreductase activity, acting on the CH-O	2
GOTERM_CC_ALL	GO:0044436~thylakoid part	6

GOTERM_BP_ALL	GO:0006812~cation transport	7
GOTERM_BP_ALL	GO:0030001~metal ion transport	4
GOTERM_MF_ALL	GO:0015297~antiporter activity	2
GOTERM_BP_ALL	GO:0051239~regulation of multicellular organismal proces	3
GOTERM_BP_ALL	GO:0016265~death	4
GOTERM_BP_ALL	GO:0008219~cell death	4
GOTERM_BP_ALL	GO:0048580~regulation of post-embryonic development	2
GOTERM_MF_ALL	GO:0004722~protein serine/threonine phosphatase activity	2
GOTERM_CC_ALL	GO:0043234~protein complex	32
GOTERM_CC_ALL	GO:0016020~membrane	156
GOTERM_CC_ALL	GO:0009570~chloroplast stroma	7
GOTERM_CC_ALL	GO:0044434~chloroplast part	20
GOTERM_BP_ALL	GO:0016071~mRNA metabolic process	2
GOTERM_CC_ALL	GO:0031966~mitochondrial membrane	3
GOTERM_BP_ALL	GO:0015672~monovalent inorganic cation transport	2
GOTERM_BP_ALL	GO:0033036~macromolecule localization	14
GOTERM_BP_ALL	GO:0012501~programmed cell death	3
GOTERM_CC_ALL	GO:0019866~organelle inner membrane	3
GOTERM_BP_ALL	GO:0010629~negative regulation of gene expression	2
GOTERM_CC_ALL	GO:0009532~plastid stroma	7
GOTERM_CC_ALL	GO:0044435~plastid part	20
GOTERM_CC_ALL	GO:0005740~mitochondrial envelope	3
GOTERM_BP_ALL	GO:0007049~cell cycle	3
GOTERM_BP_ALL	GO:0009057~macromolecule catabolic process	13
GOTERM_CC_ALL	GO:0042651~thylakoid membrane	4
GOTERM_CC_ALL	GO:0005794~Golgi apparatus	5
GOTERM_BP_ALL	GO:0045184~establishment of protein localization	8
GOTERM_BP_ALL	GO:0015031~protein transport	8
GOTERM_BP_ALL	GO:0010605~negative regulation of macromolecule metab	2
GOTERM_BP_ALL	GO:0044265~cellular macromolecule catabolic process	10
GOTERM_BP_ALL	GO:0048523~negative regulation of cellular process	2
GOTERM_MF_ALL	GO:0003723~RNA binding	28
GOTERM_BP_ALL	GO:0008104~protein localization	8
GOTERM_CC_ALL	GO:0034357~photosynthetic membrane	4
GOTERM_BP_ALL	GO:0009892~negative regulation of metabolic process	2
GOTERM_MF_ALL	GO:0004721~phosphoprotein phosphatase activity	2
GOTERM_BP_ALL	GO:0006508~proteolysis	21
GOTERM_CC_ALL	GO:0031090~organelle membrane	15
GOTERM_CC_ALL	GO:0031976~plastid thylakoid	4
GOTERM_CC_ALL	GO:0009534~chloroplast thylakoid	4
GOTERM_CC_ALL	GO:0031984~organelle subcompartment	4
GOTERM_CC_ALL	GO:0009535~chloroplast thylakoid membrane	3
GOTERM_CC_ALL	GO:0055035~plastid thylakoid membrane	3
GOTERM_MF_ALL	GO:0004872~receptor activity	8
GOTERM_BP_ALL	GO:0044260~cellular macromolecule metabolic process	159
GOTERM_BP_ALL	GO:0048519~negative regulation of biological process	4
GOTERM_BP_ALL	GO:0043170~macromolecule metabolic process	176

GOTERM_MF_ALL	GO:0060089~molecular transducer activity	11
GOTERM_MF_ALL	GO:0004871~signal transducer activity	11
GOTERM_BP_ALL	GO:0019941~modification-dependent protein catabolic pro	7
GOTERM_BP_ALL	GO:0043632~modification-dependent macromolecule cata	7
GOTERM_BP_ALL	GO:0051603~proteolysis involved in cellular protein catab	7
GOTERM_BP_ALL	GO:0044257~cellular protein catabolic process	7
GOTERM_BP_ALL	GO:0006259~DNA metabolic process	3
GOTERM_BP_ALL	GO:0030163~protein catabolic process	7
GOTERM_MF_ALL	GO:0008270~zinc ion binding	31
GOTERM_MF_ALL	GO:0016773~phosphotransferase activity, alcohol group a	21
GOTERM_MF_ALL	GO:0004713~protein tyrosine kinase activity	2
GOTERM_MF_ALL	GO:0004674~protein serine/threonine kinase activity	14
GOTERM_MF_ALL	GO:0016772~transferase activity, transferring phosphorus-	28
GOTERM_MF_ALL	GO:0016301~kinase activity	21
GOTERM_BP_ALL	GO:0016310~phosphorylation	17
GOTERM_MF_ALL	GO:0004672~protein kinase activity	14
GOTERM_BP_ALL	GO:0006412~translation	18
GOTERM_BP_ALL	GO:0006468~protein amino acid phosphorylation	14
GOTERM_BP_ALL	GO:0006796~phosphate metabolic process	18
GOTERM_BP_ALL	GO:0006793~phosphorus metabolic process	18
GOTERM_BP_ALL	GO:0019538~protein metabolic process	89
GOTERM_BP_ALL	GO:0044267~cellular protein metabolic process	73
GOTERM_BP_ALL	GO:0043687~post-translational protein modification	16
GOTERM_BP_ALL	GO:0006464~protein modification process	20
GOTERM_BP_ALL	GO:0043412~biopolymer modification	22
GOTERM_BP_ALL	GO:0043289~apocarotenoid biosynthetic process	1
GOTERM_BP_ALL	GO:0032103~positive regulation of response to external st	1
GOTERM_BP_ALL	GO:0009853~photorespiration	1
GOTERM_BP_ALL	GO:0010091~trichome branching	1
GOTERM_BP_ALL	GO:0010243~response to organic nitrogen	1
GOTERM_BP_ALL	GO:0032413~negative regulation of ion transmembrane tra	1
GOTERM_BP_ALL	GO:0018920~glyphosate metabolic process	1
GOTERM_BP_ALL	GO:0009065~glutamine family amino acid catabolic proce	1
GOTERM_BP_ALL	GO:0009156~ribonucleoside monophosphate biosynthetic	1
GOTERM_BP_ALL	GO:0010360~negative regulation of anion channel activity	1
GOTERM_BP_ALL	GO:0006722~triterpenoid metabolic process	1
GOTERM_BP_ALL	GO:0032409~regulation of transporter activity	1
GOTERM_BP_ALL	GO:0032446~protein modification by small protein conjug	1
GOTERM_BP_ALL	GO:0045596~negative regulation of cell differentiation	1
GOTERM_BP_ALL	GO:0016481~negative regulation of transcription	1
GOTERM_BP_ALL	GO:0030203~glycosaminoglycan metabolic process	1
GOTERM_BP_ALL	GO:0042256~mature ribosome assembly	1
GOTERM_BP_ALL	GO:0080134~regulation of response to stress	1
GOTERM_BP_ALL	GO:0009069~serine family amino acid metabolic process	1
GOTERM_BP_ALL	GO:0030417~nicotianamine metabolic process	1
GOTERM_BP_ALL	GO:0015994~chlorophyll metabolic process	1
GOTERM_BP_ALL	GO:0006357~regulation of transcription from RNA polym	1

GOTERM_BP_ALL	GO:0006024~glycosaminoglycan biosynthetic process	1
GOTERM_BP_ALL	GO:0042430~indole and derivative metabolic process	1
GOTERM_BP_ALL	GO:0009827~plant-type cell wall modification	1
GOTERM_BP_ALL	GO:0046856~phosphoinositide dephosphorylation	1
GOTERM_BP_ALL	GO:0009690~cytokinin metabolic process	1
GOTERM_BP_ALL	GO:0042777~plasma membrane ATP synthesis coupled pr	1
GOTERM_BP_ALL	GO:0008380~RNA splicing	1
GOTERM_BP_ALL	GO:0010253~UDP-rhamnose biosynthetic process	1
GOTERM_BP_ALL	GO:0006586~indolalkylamine metabolic process	1
GOTERM_BP_ALL	GO:0007186~G-protein coupled receptor protein signaling	1
GOTERM_BP_ALL	GO:0051645~Golgi localization	1
GOTERM_BP_ALL	GO:0009817~defense response to fungus, incompatible int	1
GOTERM_BP_ALL	GO:0009851~auxin biosynthetic process	1
GOTERM_BP_ALL	GO:0046174~polyol catabolic process	1
GOTERM_BP_ALL	GO:0009697~salicylic acid biosynthetic process	1
GOTERM_BP_ALL	GO:0051258~protein polymerization	1
GOTERM_BP_ALL	GO:0010026~trichome differentiation	1
GOTERM_BP_ALL	GO:0044070~regulation of anion transport	1
GOTERM_BP_ALL	GO:0048235~pollen sperm cell differentiation	1
GOTERM_BP_ALL	GO:0044038~cell wall macromolecule biosynthetic proces	1
GOTERM_BP_ALL	GO:0019310~inositol catabolic process	1
GOTERM_BP_ALL	GO:0051174~regulation of phosphorus metabolic process	1
GOTERM_BP_ALL	GO:0006401~RNA catabolic process	1
GOTERM_BP_ALL	GO:0008295~spermidine biosynthetic process	1
GOTERM_BP_ALL	GO:0009098~leucine biosynthetic process	1
GOTERM_BP_ALL	GO:0007602~phototransduction	1
GOTERM_BP_ALL	GO:0010361~regulation of anion channel activity by blue l	1
GOTERM_BP_ALL	GO:0010118~stomatal movement	1
GOTERM_BP_ALL	GO:0019300~rhamnose biosynthetic process	1
GOTERM_BP_ALL	GO:0048444~floral organ morphogenesis	1
GOTERM_BP_ALL	GO:0032147~activation of protein kinase activity	1
GOTERM_BP_ALL	GO:0051028~mRNA transport	1
GOTERM_BP_ALL	GO:0006537~glutamate biosynthetic process	1
GOTERM_BP_ALL	GO:0034765~regulation of ion transmembrane transport	1
GOTERM_BP_ALL	GO:0009641~shade avoidance	1
GOTERM_BP_ALL	GO:0046685~response to arsenic	1
GOTERM_BP_ALL	GO:0006402~mRNA catabolic process	1
GOTERM_BP_ALL	GO:0009396~folic acid and derivative biosynthetic proces	1
GOTERM_BP_ALL	GO:0070589~cellular component macromolecule biosynth	1
GOTERM_BP_ALL	GO:0017004~cytochrome complex assembly	1
GOTERM_BP_ALL	GO:0031647~regulation of protein stability	1
GOTERM_BP_ALL	GO:0043467~regulation of generation of precursor metabo	1
GOTERM_BP_ALL	GO:0009638~phototropism	1
GOTERM_BP_ALL	GO:0048638~regulation of developmental growth	1
GOTERM_BP_ALL	GO:0007018~microtubule-based movement	1
GOTERM_BP_ALL	GO:0044273~sulfur compound catabolic process	1
GOTERM_BP_ALL	GO:0009127~purine nucleoside monophosphate biosynthe	1

GOTERM_BP_ALL	GO:0043455~regulation of secondary metabolic process	1
GOTERM_BP_ALL	GO:0009086~methionine biosynthetic process	1
GOTERM_BP_ALL	GO:0030245~cellulose catabolic process	1
GOTERM_BP_ALL	GO:0046777~protein amino acid autophosphorylation	1
GOTERM_BP_ALL	GO:0032412~regulation of ion transmembrane transporter	1
GOTERM_BP_ALL	GO:0006821~chloride transport	1
GOTERM_BP_ALL	GO:0006805~xenobiotic metabolic process	1
GOTERM_BP_ALL	GO:0006562~proline catabolic process	1
GOTERM_BP_ALL	GO:0006023~aminoglycan biosynthetic process	1
GOTERM_BP_ALL	GO:0006568~tryptophan metabolic process	1
GOTERM_BP_ALL	GO:0009704~de-etiolation	1
GOTERM_BP_ALL	GO:0050821~protein stabilization	1
GOTERM_BP_ALL	GO:0032957~inositol trisphosphate metabolic process	1
GOTERM_BP_ALL	GO:0048507~meristem development	1
GOTERM_BP_ALL	GO:0006281~DNA repair	1
GOTERM_BP_ALL	GO:0043068~positive regulation of programmed cell death	1
GOTERM_BP_ALL	GO:0009123~nucleoside monophosphate metabolic proces	1
GOTERM_BP_ALL	GO:0009648~photoperiodism	1
GOTERM_BP_ALL	GO:0034620~cellular response to unfolded protein	1
GOTERM_BP_ALL	GO:0002213~defense response to insect	1
GOTERM_BP_ALL	GO:0033013~tetrapyrrole metabolic process	1
GOTERM_BP_ALL	GO:0000226~microtubule cytoskeleton organization	1
GOTERM_BP_ALL	GO:0006000~fructose metabolic process	1
GOTERM_BP_ALL	GO:0046219~indolalkylamine biosynthetic process	1
GOTERM_BP_ALL	GO:0010647~positive regulation of cell communication	1
GOTERM_BP_ALL	GO:0043269~regulation of ion transport	1
GOTERM_BP_ALL	GO:0000097~sulfur amino acid biosynthetic process	1
GOTERM_BP_ALL	GO:0010263~tricyclic triterpenoid biosynthetic process	1
GOTERM_BP_ALL	GO:0006325~chromatin organization	1
GOTERM_BP_ALL	GO:0009962~regulation of flavonoid biosynthetic process	1
GOTERM_BP_ALL	GO:0001522~pseudouridine synthesis	1
GOTERM_BP_ALL	GO:0051131~chaperone-mediated protein complex assemb	1
GOTERM_BP_ALL	GO:0051667~establishment of plastid localization	1
GOTERM_BP_ALL	GO:0019220~regulation of phosphate metabolic process	1
GOTERM_BP_ALL	GO:0042761~very-long-chain fatty acid biosynthetic proce	1
GOTERM_BP_ALL	GO:0019299~rhamnose metabolic process	1
GOTERM_BP_ALL	GO:0046083~adenine metabolic process	1
GOTERM_BP_ALL	GO:0048768~root hair cell tip growth	1
GOTERM_BP_ALL	GO:0032410~negative regulation of transporter activity	1
GOTERM_BP_ALL	GO:0048509~regulation of meristem development	1
GOTERM_BP_ALL	GO:0006986~response to unfolded protein	1
GOTERM_BP_ALL	GO:0009687~abscisic acid metabolic process	1
GOTERM_BP_ALL	GO:0009850~auxin metabolic process	1
GOTERM_BP_ALL	GO:0000270~peptidoglycan metabolic process	1
GOTERM_BP_ALL	GO:0009124~nucleoside monophosphate biosynthetic proc	1
GOTERM_BP_ALL	GO:0009823~cytokinin catabolic process	1
GOTERM_BP_ALL	GO:0051607~defense response to virus	1

GOTERM_BP_ALL	GO:0048878~chemical homeostasis	1
GOTERM_BP_ALL	GO:0006974~response to DNA damage stimulus	1
GOTERM_BP_ALL	GO:0045934~negative regulation of nucleobase, nucleosid	1
GOTERM_BP_ALL	GO:0010646~regulation of cell communication	1
GOTERM_BP_ALL	GO:0007623~circadian rhythm	1
GOTERM_BP_ALL	GO:0000578~embryonic axis specification	1
GOTERM_BP_ALL	GO:0080027~response to herbivore	1
GOTERM_BP_ALL	GO:0050982~detection of mechanical stimulus	1
GOTERM_BP_ALL	GO:0006168~adenine salvage	1
GOTERM_BP_ALL	GO:0032879~regulation of localization	1
GOTERM_BP_ALL	GO:0007276~gamete generation	1
GOTERM_BP_ALL	GO:0046487~glyoxylate metabolic process	1
GOTERM_BP_ALL	GO:0016139~glycoside catabolic process	1
GOTERM_BP_ALL	GO:0006188~IMP biosynthetic process	1
GOTERM_BP_ALL	GO:0048571~long-day photoperiodism	1
GOTERM_BP_ALL	GO:0009082~branched chain family amino acid biosynthet	1
GOTERM_BP_ALL	GO:0009798~axis specification	1
GOTERM_BP_ALL	GO:0006097~glyoxylate cycle	1
GOTERM_BP_ALL	GO:0006560~proline metabolic process	1
GOTERM_BP_ALL	GO:0009410~response to xenobiotic stimulus	1
GOTERM_BP_ALL	GO:0031407~oxylipin metabolic process	1
GOTERM_BP_ALL	GO:0009161~ribonucleoside monophosphate metabolic pr	1
GOTERM_BP_ALL	GO:0006022~aminoglycan metabolic process	1
GOTERM_BP_ALL	GO:0033517~myo-inositol hexakisphosphate metabolic pr	1
GOTERM_BP_ALL	GO:0042325~regulation of phosphorylation	1
GOTERM_BP_ALL	GO:0009423~chorismate biosynthetic process	1
GOTERM_BP_ALL	GO:0010152~pollen maturation	1
GOTERM_BP_ALL	GO:0009168~purine ribonucleoside monophosphate biosy	1
GOTERM_BP_ALL	GO:0002252~immune effector process	1
GOTERM_BP_ALL	GO:0006351~transcription, DNA-dependent	1
GOTERM_BP_ALL	GO:0009901~anther dehiscence	1
GOTERM_BP_ALL	GO:0009067~aspartate family amino acid biosynthetic pro	1
GOTERM_BP_ALL	GO:0042548~regulation of photosynthesis, light reaction	1
GOTERM_BP_ALL	GO:0070647~protein modification by small protein conjug	1
GOTERM_BP_ALL	GO:0033674~positive regulation of kinase activity	1
GOTERM_BP_ALL	GO:0009828~plant-type cell wall loosening	1
GOTERM_BP_ALL	GO:0006551~leucine metabolic process	1
GOTERM_BP_ALL	GO:0048544~recognition of pollen	1
GOTERM_BP_ALL	GO:0051049~regulation of transport	1
GOTERM_BP_ALL	GO:0007584~response to nutrient	1
GOTERM_BP_ALL	GO:0051644~plastid localization	1
GOTERM_BP_ALL	GO:0042773~ATP synthesis coupled electron transport	1
GOTERM_BP_ALL	GO:0016311~dephosphorylation	1
GOTERM_BP_ALL	GO:0032968~positive regulation of RNA elongation from	1
GOTERM_BP_ALL	GO:0015893~drug transport	1
GOTERM_BP_ALL	GO:0010438~cellular response to sulfur starvation	1
GOTERM_BP_ALL	GO:0030036~actin cytoskeleton organization	1

GOTERM_BP_ALL	GO:0040008~regulation of growth	1
GOTERM_BP_ALL	GO:0009695~jasmonic acid biosynthetic process	1
GOTERM_BP_ALL	GO:0000038~very-long-chain fatty acid metabolic process	1
GOTERM_BP_ALL	GO:0006855~multidrug transport	1
GOTERM_BP_ALL	GO:0042493~response to drug	1
GOTERM_BP_ALL	GO:0045490~pectin catabolic process	1
GOTERM_BP_ALL	GO:0048441~petal development	1
GOTERM_BP_ALL	GO:0006596~polyamine biosynthetic process	1
GOTERM_BP_ALL	GO:0032786~positive regulation of RNA elongation	1
GOTERM_BP_ALL	GO:0042775~mitochondrial ATP synthesis coupled electro	1
GOTERM_BP_ALL	GO:0051181~cofactor transport	1
GOTERM_BP_ALL	GO:0016458~gene silencing	1
GOTERM_BP_ALL	GO:0018298~protein-chromophore linkage	1
GOTERM_BP_ALL	GO:0016125~sterol metabolic process	1
GOTERM_BP_ALL	GO:0015886~heme transport	1
GOTERM_BP_ALL	GO:0042446~hormone biosynthetic process	1
GOTERM_BP_ALL	GO:0031540~regulation of anthocyanin biosynthetic proce	1
GOTERM_BP_ALL	GO:0010090~trichome morphogenesis	1
GOTERM_BP_ALL	GO:0010031~circumnutation	1
GOTERM_BP_ALL	GO:0051259~protein oligomerization	1
GOTERM_BP_ALL	GO:0034284~response to monosaccharide stimulus	1
GOTERM_BP_ALL	GO:0009942~longitudinal axis specification	1
GOTERM_BP_ALL	GO:0045860~positive regulation of protein kinase activity	1
GOTERM_BP_ALL	GO:0032107~regulation of response to nutrient levels	1
GOTERM_BP_ALL	GO:0034440~lipid oxidation	1
GOTERM_BP_ALL	GO:0045859~regulation of protein kinase activity	1
GOTERM_BP_ALL	GO:0043288~apocarotenoid metabolic process	1
GOTERM_BP_ALL	GO:0048496~maintenance of organ identity	1
GOTERM_BP_ALL	GO:0008615~pyridoxine biosynthetic process	1
GOTERM_BP_ALL	GO:0010073~meristem maintenance	1
GOTERM_BP_ALL	GO:0051338~regulation of transferase activity	1
GOTERM_BP_ALL	GO:0009627~systemic acquired resistance	1
GOTERM_BP_ALL	GO:0051236~establishment of RNA localization	1
GOTERM_BP_ALL	GO:0007267~cell-cell signaling	1
GOTERM_BP_ALL	GO:0010043~response to zinc ion	1
GOTERM_BP_ALL	GO:0006122~mitochondrial electron transport, ubiquinol t	1
GOTERM_BP_ALL	GO:0009688~abscisic acid biosynthetic process	1
GOTERM_BP_ALL	GO:0042181~ketone biosynthetic process	1
GOTERM_BP_ALL	GO:0008272~sulfate transport	1
GOTERM_BP_ALL	GO:0009875~pollen-pistil interaction	1
GOTERM_BP_ALL	GO:0009066~aspartate family amino acid metabolic proce	1
GOTERM_BP_ALL	GO:0042434~indole derivative metabolic process	1
GOTERM_BP_ALL	GO:0022904~respiratory electron transport chain	1
GOTERM_BP_ALL	GO:0046700~heterocycle catabolic process	1
GOTERM_BP_ALL	GO:0010196~nonphotochemical quenching	1
GOTERM_BP_ALL	GO:0019395~fatty acid oxidation	1
GOTERM_BP_ALL	GO:0009740~gibberellic acid mediated signaling	1

GOTERM_BP_ALL	GO:0006096~glycolysis	1
GOTERM_BP_ALL	GO:0009825~multidimensional cell growth	1
GOTERM_BP_ALL	GO:0010143~cutin biosynthetic process	1
GOTERM_BP_ALL	GO:0046839~phospholipid dephosphorylation	1
GOTERM_BP_ALL	GO:0015995~chlorophyll biosynthetic process	1
GOTERM_BP_ALL	GO:0031537~regulation of anthocyanin metabolic process	1
GOTERM_BP_ALL	GO:0046040~IMP metabolic process	1
GOTERM_BP_ALL	GO:0010149~senescence	1
GOTERM_BP_ALL	GO:0048573~photoperiodism, flowering	1
GOTERM_BP_ALL	GO:0009583~detection of light stimulus	1
GOTERM_BP_ALL	GO:0009647~skotomorphogenesis	1
GOTERM_BP_ALL	GO:0010157~response to chlorate	1
GOTERM_BP_ALL	GO:0051276~chromosome organization	1
GOTERM_BP_ALL	GO:0010017~red or far red light signaling pathway	1
GOTERM_BP_ALL	GO:0010362~negative regulation of anion channel activity	1
GOTERM_BP_ALL	GO:0043549~regulation of kinase activity	1
GOTERM_BP_ALL	GO:0048446~petal morphogenesis	1
GOTERM_BP_ALL	GO:0000162~tryptophan biosynthetic process	1
GOTERM_BP_ALL	GO:0000096~sulfur amino acid metabolic process	1
GOTERM_BP_ALL	GO:0010109~regulation of photosynthesis	1
GOTERM_BP_ALL	GO:0019762~glucosinolate catabolic process	1
GOTERM_BP_ALL	GO:0009867~jasmonic acid mediated signaling pathway	1
GOTERM_BP_ALL	GO:0019685~photosynthesis, dark reaction	1
GOTERM_BP_ALL	GO:0006014~D-ribose metabolic process	1
GOTERM_BP_ALL	GO:0032259~methylation	1
GOTERM_BP_ALL	GO:0009624~response to nematode	1
GOTERM_BP_ALL	GO:0009646~response to absence of light	1
GOTERM_BP_ALL	GO:0009742~brassinosteroid mediated signaling	1
GOTERM_BP_ALL	GO:0080003~thalianol metabolic process	1
GOTERM_BP_ALL	GO:0042732~D-xylose metabolic process	1
GOTERM_BP_ALL	GO:0032106~positive regulation of response to extracellul	1
GOTERM_BP_ALL	GO:0010654~apical cell fate commitment	1
GOTERM_BP_ALL	GO:0016568~chromatin modification	1
GOTERM_BP_ALL	GO:0001708~cell fate specification	1
GOTERM_BP_ALL	GO:0010382~cellular cell wall macromolecule metabolic p	1
GOTERM_BP_ALL	GO:0033478~UDP-rhamnose metabolic process	1
GOTERM_BP_ALL	GO:0032774~RNA biosynthetic process	1
GOTERM_BP_ALL	GO:0050657~nucleic acid transport	1
GOTERM_BP_ALL	GO:0010315~auxin efflux	1
GOTERM_BP_ALL	GO:0008535~respiratory chain complex IV assembly	1
GOTERM_BP_ALL	GO:0006792~regulation of sulfur utilization	1
GOTERM_BP_ALL	GO:0006863~purine transport	1
GOTERM_BP_ALL	GO:0032104~regulation of response to extracellular stimul	1
GOTERM_BP_ALL	GO:0055046~microgametogenesis	1
GOTERM_BP_ALL	GO:0015833~peptide transport	1
GOTERM_BP_ALL	GO:0048583~regulation of response to stimulus	1
GOTERM_BP_ALL	GO:0045488~pectin metabolic process	1

GOTERM_BP_ALL	GO:0009630~gravitropism	1
GOTERM_BP_ALL	GO:0080135~regulation of cellular response to stress	1
GOTERM_BP_ALL	GO:0006778~porphyrin metabolic process	1
GOTERM_BP_ALL	GO:0009566~fertilization	1
GOTERM_BP_ALL	GO:0048574~long-day photoperiodism, flowering	1
GOTERM_BP_ALL	GO:0042447~hormone catabolic process	1
GOTERM_BP_ALL	GO:0030244~cellulose biosynthetic process	1
GOTERM_BP_ALL	GO:0008216~spermidine metabolic process	1
GOTERM_BP_ALL	GO:0009694~jasmonic acid metabolic process	1
GOTERM_BP_ALL	GO:0016567~protein ubiquitination	1
GOTERM_BP_ALL	GO:0019759~glycosinolate catabolic process	1
GOTERM_BP_ALL	GO:0042435~indole derivative biosynthetic process	1
GOTERM_BP_ALL	GO:0022898~regulation of transmembrane transporter acti	1
GOTERM_BP_ALL	GO:0009715~chalcone biosynthetic process	1
GOTERM_BP_ALL	GO:0016098~monoterpenoid metabolic process	1
GOTERM_BP_ALL	GO:0080040~positive regulation of cellular response to ph	1
GOTERM_BP_ALL	GO:0009749~response to glucose stimulus	1
GOTERM_BP_ALL	GO:0006555~methionine metabolic process	1
GOTERM_BP_ALL	GO:0030418~nicotianamine biosynthetic process	1
GOTERM_BP_ALL	GO:0034762~regulation of transmembrane transport	1
GOTERM_BP_ALL	GO:0010558~negative regulation of macromolecule biosyn	1
GOTERM_BP_ALL	GO:0010476~gibberellin-mediated signaling	1
GOTERM_BP_ALL	GO:0042255~ribosome assembly	1
GOTERM_BP_ALL	GO:0006366~transcription from RNA polymerase II prom	1
GOTERM_BP_ALL	GO:0030104~water homeostasis	1
GOTERM_BP_ALL	GO:0009226~nucleotide-sugar biosynthetic process	1
GOTERM_BP_ALL	GO:0005983~starch catabolic process	1
GOTERM_BP_ALL	GO:0051274~beta-glucan biosynthetic process	1
GOTERM_BP_ALL	GO:0009567~double fertilization forming a zygote and end	1
GOTERM_BP_ALL	GO:0051789~response to protein stimulus	1
GOTERM_BP_ALL	GO:0010288~response to lead ion	1
GOTERM_BP_ALL	GO:0048232~male gamete generation	1
GOTERM_BP_ALL	GO:0050879~multicellular organismal movement	1
GOTERM_BP_ALL	GO:0010044~response to aluminum ion	1
GOTERM_BP_ALL	GO:0010608~posttranscriptional regulation of gene expres	1
GOTERM_BP_ALL	GO:0048545~response to steroid hormone stimulus	1
GOTERM_BP_ALL	GO:0000956~nuclear-transcribed mRNA catabolic process	1
GOTERM_BP_ALL	GO:0006857~oligopeptide transport	1
GOTERM_BP_ALL	GO:0043085~positive regulation of catalytic activity	1
GOTERM_BP_ALL	GO:0008614~pyridoxine metabolic process	1
GOTERM_BP_ALL	GO:0006075~1,3-beta-glucan biosynthetic process	1
GOTERM_BP_ALL	GO:0048825~cotyledon development	1
GOTERM_BP_ALL	GO:0032268~regulation of cellular protein metabolic proc	1
GOTERM_BP_ALL	GO:0006839~mitochondrial transport	1
GOTERM_BP_ALL	GO:0042547~cell wall modification during multidimensio	1
GOTERM_BP_ALL	GO:0032101~regulation of response to external stimulus	1
GOTERM_BP_ALL	GO:0034754~cellular hormone metabolic process	1

GOTERM_BP_ALL	GO:0006635~fatty acid beta-oxidation	1
GOTERM_BP_ALL	GO:0043335~protein unfolding	1
GOTERM_BP_ALL	GO:0019253~reductive pentose-phosphate cycle	1
GOTERM_BP_ALL	GO:0042335~cuticle development	1
GOTERM_BP_ALL	GO:0008215~spermine metabolic process	1
GOTERM_BP_ALL	GO:0006949~syncytium formation	1
GOTERM_BP_ALL	GO:0010359~regulation of anion channel activity	1
GOTERM_BP_ALL	GO:0048497~maintenance of floral organ identity	1
GOTERM_BP_ALL	GO:0016145~S-glycoside catabolic process	1
GOTERM_BP_ALL	GO:0006563~L-serine metabolic process	1
GOTERM_BP_ALL	GO:0032958~inositol phosphate biosynthetic process	1
GOTERM_BP_ALL	GO:0051791~medium-chain fatty acid metabolic process	1
GOTERM_BP_ALL	GO:0010942~positive regulation of cell death	1
GOTERM_BP_ALL	GO:0009167~purine ribonucleoside monophosphate metab	1
GOTERM_BP_ALL	GO:0008037~cell recognition	1
GOTERM_BP_ALL	GO:0048465~corolla development	1
GOTERM_BP_ALL	GO:0009273~peptidoglycan-based cell wall biogenesis	1
GOTERM_BP_ALL	GO:0050658~RNA transport	1
GOTERM_BP_ALL	GO:0019295~coenzyme M biosynthetic process	1
GOTERM_BP_ALL	GO:0006597~spermine biosynthetic process	1
GOTERM_BP_ALL	GO:0046084~adenine biosynthetic process	1
GOTERM_BP_ALL	GO:0009252~peptidoglycan biosynthetic process	1
GOTERM_BP_ALL	GO:0010683~tricyclic triterpenoid metabolic process	1
GOTERM_BP_ALL	GO:0043101~purine salvage	1
GOTERM_BP_ALL	GO:0031327~negative regulation of cellular biosynthetic p	1
GOTERM_BP_ALL	GO:0009958~positive gravitropism	1
GOTERM_BP_ALL	GO:0000184~nuclear-transcribed mRNA catabolic process	1
GOTERM_BP_ALL	GO:0006723~cuticle hydrocarbon biosynthetic process	1
GOTERM_BP_ALL	GO:0042816~vitamin B6 metabolic process	1
GOTERM_BP_ALL	GO:0007205~activation of protein kinase C activity by G-p	1
GOTERM_BP_ALL	GO:0009833~primary cell wall biogenesis	1
GOTERM_BP_ALL	GO:0051792~medium-chain fatty acid biosynthetic proces	1
GOTERM_BP_ALL	GO:0022618~ribonucleoprotein complex assembly	1
GOTERM_BP_ALL	GO:0009126~purine nucleoside monophosphate metabolic	1
GOTERM_BP_ALL	GO:0043650~dicarboxylic acid biosynthetic process	1
GOTERM_BP_ALL	GO:0010075~regulation of meristem growth	1
GOTERM_BP_ALL	GO:0031408~oxylipin biosynthetic process	1
GOTERM_BP_ALL	GO:0009612~response to mechanical stimulus	1
GOTERM_BP_ALL	GO:0048511~rhythmic process	1
GOTERM_BP_ALL	GO:0051656~establishment of organelle localization	1
GOTERM_BP_ALL	GO:0009814~defense response, incompatible interaction	1
GOTERM_BP_ALL	GO:0009834~secondary cell wall biogenesis	1
GOTERM_BP_ALL	GO:0006074~1,3-beta-glucan metabolic process	1
GOTERM_BP_ALL	GO:0019742~pentacyclic triterpenoid metabolic process	1
GOTERM_BP_ALL	GO:0048563~post-embryonic organ morphogenesis	1
GOTERM_BP_ALL	GO:0043096~purine base salvage	1
GOTERM_BP_ALL	GO:0019252~starch biosynthetic process	1

GOTERM_BP_ALL	GO:0006090~pyruvate metabolic process	1
GOTERM_BP_ALL	GO:0019296~coenzyme M metabolic process	1
GOTERM_BP_ALL	GO:0031324~negative regulation of cellular metabolic pro	1
GOTERM_BP_ALL	GO:0016126~sterol biosynthetic process	1
GOTERM_BP_ALL	GO:0012502~induction of programmed cell death	1
GOTERM_BP_ALL	GO:0009746~response to hexose stimulus	1
GOTERM_BP_ALL	GO:0006397~mRNA processing	1
GOTERM_BP_ALL	GO:0010193~response to ozone	1
GOTERM_BP_ALL	GO:0032784~regulation of RNA elongation	1
GOTERM_BP_ALL	GO:0043401~steroid hormone mediated signaling	1
GOTERM_BP_ALL	GO:0009890~negative regulation of biosynthetic process	1
GOTERM_BP_ALL	GO:0015851~nucleobase transport	1
GOTERM_BP_ALL	GO:0045595~regulation of cell differentiation	1
GOTERM_BP_ALL	GO:0009831~plant-type cell wall modification during mult	1
GOTERM_BP_ALL	GO:0044036~cell wall macromolecule metabolic process	1
GOTERM_BP_ALL	GO:0048584~positive regulation of response to stimulus	1
GOTERM_BP_ALL	GO:0010223~secondary shoot formation	1
GOTERM_BP_ALL	GO:0006760~folic acid and derivative metabolic process	1
GOTERM_BP_ALL	GO:0006414~translational elongation	1
GOTERM_BP_ALL	GO:0044093~positive regulation of molecular function	1
GOTERM_BP_ALL	GO:0009585~red, far-red light phototransduction	1
GOTERM_BP_ALL	GO:0034605~cellular response to heat	1
GOTERM_BP_ALL	GO:0048767~root hair elongation	1
GOTERM_BP_ALL	GO:0006544~glycine metabolic process	1
GOTERM_BP_ALL	GO:0010346~shoot formation	1
GOTERM_BP_ALL	GO:0051347~positive regulation of transferase activity	1
GOTERM_BP_ALL	GO:0051273~beta-glucan metabolic process	1
GOTERM_BP_ALL	GO:0046488~phosphatidylinositol metabolic process	1
GOTERM_BP_ALL	GO:0032109~positive regulation of response to nutrient le	1
GOTERM_BP_ALL	GO:0051093~negative regulation of developmental proces	1
GOTERM_BP_ALL	GO:0060151~peroxisome localization	1
GOTERM_BP_ALL	GO:0006526~arginine biosynthetic process	1
GOTERM_BP_ALL	GO:0033014~tetrapyrrole biosynthetic process	1
GOTERM_BP_ALL	GO:0015977~carbon utilization by fixation of carbon dioxi	1
GOTERM_BP_ALL	GO:0006779~porphyrin biosynthetic process	1
GOTERM_BP_ALL	GO:0009900~dehiscence	1
GOTERM_BP_ALL	GO:0000060~protein import into nucleus, translocation	1
GOTERM_BP_ALL	GO:0006081~cellular aldehyde metabolic process	1
GOTERM_BP_ALL	GO:0042550~photosystem I stabilization	1
GOTERM_BP_ALL	GO:0042819~vitamin B6 biosynthetic process	1
GOTERM_BP_ALL	GO:0051172~negative regulation of nitrogen compound m	1
GOTERM_BP_ALL	GO:0010345~suberin biosynthetic process	1
GOTERM_BP_ALL	GO:0006403~RNA localization	1
GOTERM_BP_ALL	GO:0051051~negative regulation of transport	1
GOTERM_BP_ALL	GO:0009902~chloroplast relocation	1
GOTERM_BP_ALL	GO:0019745~pentacyclic triterpenoid biosynthetic process	1
GOTERM_BP_ALL	GO:0008654~phospholipid biosynthetic process	1

GOTERM_BP_ALL	GO:0009714~chalcone metabolic process	1
GOTERM_BP_ALL	GO:0006003~fructose 2,6-bisphosphate metabolic process	1
GOTERM_BP_ALL	GO:0006094~gluconeogenesis	1
GOTERM_BP_ALL	GO:0009083~branched chain family amino acid catabolic	1
GOTERM_BP_ALL	GO:0016099~monoterpenoid biosynthetic process	1
GOTERM_BP_ALL	GO:0010264~myo-inositol hexakisphosphate biosynthetic	1
GOTERM_BP_ALL	GO:0034243~regulation of RNA elongation from RNA pol	1
GOTERM_BP_ALL	GO:0051646~mitochondrion localization	1
GOTERM_BP_ALL	GO:0016104~triterpenoid biosynthetic process	1
GOTERM_BP_ALL	GO:0035315~hair cell differentiation	1
GOTERM_CC_ALL	GO:0015030~Cajal body	1
GOTERM_CC_ALL	GO:0034707~chloride channel complex	1
GOTERM_CC_ALL	GO:0031012~extracellular matrix	1
GOTERM_CC_ALL	GO:0016591~DNA-directed RNA polymerase II, holoenzy	1
GOTERM_CC_ALL	GO:0044439~peroxisomal part	1
GOTERM_CC_ALL	GO:0016602~CCAAT-binding factor complex	1
GOTERM_CC_ALL	GO:0005852~eukaryotic translation initiation factor 3 com	1
GOTERM_CC_ALL	GO:0010169~thioglucosidase complex	1
GOTERM_CC_ALL	GO:0031907~microbody lumen	1
GOTERM_CC_ALL	GO:0009925~basal plasma membrane	1
GOTERM_CC_ALL	GO:0000148~1,3-beta-glucan synthase complex	1
GOTERM_CC_ALL	GO:0005746~mitochondrial respiratory chain	1
GOTERM_CC_ALL	GO:0009517~PSII associated light-harvesting complex II	1
GOTERM_CC_ALL	GO:0016461~unconventional myosin complex	1
GOTERM_CC_ALL	GO:0005753~mitochondrial proton-transporting ATP synt	1
GOTERM_CC_ALL	GO:0045178~basal part of cell	1
GOTERM_CC_ALL	GO:0032040~small-subunit processome	1
GOTERM_CC_ALL	GO:0005750~mitochondrial respiratory chain complex III	1
GOTERM_CC_ALL	GO:0009501~amyloplast	1
GOTERM_CC_ALL	GO:0005782~peroxisomal matrix	1
GOTERM_CC_ALL	GO:0005768~endosome	1
GOTERM_CC_ALL	GO:0044438~microbody part	1
GOTERM_CC_ALL	GO:0048492~ribulose bisphosphate carboxylase complex	1
GOTERM_CC_ALL	GO:0005625~soluble fraction	1
GOTERM_CC_ALL	GO:0042406~extrinsic to endoplasmic reticulum membran	1
GOTERM_CC_ALL	GO:0030684~preribosome	1
GOTERM_CC_ALL	GO:0055029~nuclear DNA-directed RNA polymerase com	1
GOTERM_CC_ALL	GO:0042175~nuclear envelope-endoplasmic reticulum net	1
GOTERM_CC_ALL	GO:0000323~lytic vacuole	1
GOTERM_CC_ALL	GO:0045275~respiratory chain complex III	1
GOTERM_CC_ALL	GO:0009524~phragmoplast	1
GOTERM_CC_ALL	GO:0005815~microtubule organizing center	1
GOTERM_CC_ALL	GO:0034702~ion channel complex	1
GOTERM_CC_ALL	GO:0009573~chloroplast ribulose bisphosphate carboxylas	1
GOTERM_CC_ALL	GO:0016604~nuclear body	1
GOTERM_CC_ALL	GO:0030880~RNA polymerase complex	1
GOTERM_CC_ALL	GO:0009341~beta-galactosidase complex	1

GOTERM_CC_ALL	GO:0009503~thylakoid light-harvesting complex	1
GOTERM_CC_ALL	GO:0016323~basolateral plasma membrane	1
GOTERM_CC_ALL	GO:0031312~extrinsic to organelle membrane	1
GOTERM_CC_ALL	GO:0005665~DNA-directed RNA polymerase II, core com	1
GOTERM_CC_ALL	GO:0009504~cell plate	1
GOTERM_CC_ALL	GO:0009521~photosystem	1
GOTERM_CC_ALL	GO:0005875~microtubule associated complex	1
GOTERM_CC_ALL	GO:0005667~transcription factor complex	1
GOTERM_CC_ALL	GO:0009514~glyoxysome	1
GOTERM_CC_ALL	GO:0000428~DNA-directed RNA polymerase complex	1
GOTERM_CC_ALL	GO:0010319~stromule	1
GOTERM_CC_ALL	GO:0009543~chloroplast thylakoid lumen	1
GOTERM_CC_ALL	GO:0045298~tubulin complex	1
GOTERM_CC_ALL	GO:0000267~cell fraction	1
GOTERM_CC_ALL	GO:0009523~photosystem II	1
GOTERM_CC_ALL	GO:0031978~plastid thylakoid lumen	1
GOTERM_CC_ALL	GO:0000922~spindle pole	1
GOTERM_CC_ALL	GO:0005853~eukaryotic translation elongation factor 1 co	1
GOTERM_CC_ALL	GO:0045261~proton-transporting ATP synthase complex,	1
GOTERM_CC_ALL	GO:0005819~spindle	1
GOTERM_CC_ALL	GO:0005789~endoplasmic reticulum membrane	1
GOTERM_CC_ALL	GO:0005615~extracellular space	1
GOTERM_MF_ALL	GO:0003871~5-methyltetrahydropteroyltriglutamate-homo	1
GOTERM_MF_ALL	GO:0045300~acyl-[acyl-carrier-protein] desaturase activity	1
GOTERM_MF_ALL	GO:0046915~transition metal ion transmembrane transport	1
GOTERM_MF_ALL	GO:0016782~transferase activity, transferring sulfur-conta	1
GOTERM_MF_ALL	GO:0004499~flavin-containing monooxygenase activity	1
GOTERM_MF_ALL	GO:0080032~methyl jasmonate esterase activity	1
GOTERM_MF_ALL	GO:0004512~inositol-3-phosphate synthase activity	1
GOTERM_MF_ALL	GO:0008417~fucosyltransferase activity	1
GOTERM_MF_ALL	GO:0008934~inositol-1(or 4)-monophosphatase activity	1
GOTERM_MF_ALL	GO:0015923~mannosidase activity	1
GOTERM_MF_ALL	GO:0005543~phospholipid binding	1
GOTERM_MF_ALL	GO:0008936~nicotinamidase activity	1
GOTERM_MF_ALL	GO:0050551~myrcene synthase activity	1
GOTERM_MF_ALL	GO:0018685~alkane 1-monooxygenase activity	1
GOTERM_MF_ALL	GO:0015450~P-P-bond-hydrolysis-driven protein transme	1
GOTERM_MF_ALL	GO:0010280~UDP-L-rhamnose synthase activity	1
GOTERM_MF_ALL	GO:0010436~carotenoid dioxygenase activity	1
GOTERM_MF_ALL	GO:0003854~3-beta-hydroxy-delta5-steroid dehydrogenas	1
GOTERM_MF_ALL	GO:0004565~beta-galactosidase activity	1
GOTERM_MF_ALL	GO:0008121~ubiquinol-cytochrome-c reductase activity	1
GOTERM_MF_ALL	GO:0015082~di-, tri-valent inorganic cation transmembran	1
GOTERM_MF_ALL	GO:0016307~phosphatidylinositol phosphate kinase activi	1
GOTERM_MF_ALL	GO:0015200~methylammonium transmembrane transporte	1
GOTERM_MF_ALL	GO:0009815~1-aminocyclopropane-1-carboxylate oxidase	1
GOTERM_MF_ALL	GO:0004018~N6-(1,2-dicarboxyethyl)AMP AMP-lyase (fu	1

GOTERM_MF_ALL	GO:0022833~mechanically gated channel activity	1
GOTERM_MF_ALL	GO:0051539~4 iron, 4 sulfur cluster binding	1
GOTERM_MF_ALL	GO:0004657~proline dehydrogenase activity	1
GOTERM_MF_ALL	GO:0080133~midchain alkane hydroxylase activity	1
GOTERM_MF_ALL	GO:0003873~6-phosphofructo-2-kinase activity	1
GOTERM_MF_ALL	GO:0016229~steroid dehydrogenase activity	1
GOTERM_MF_ALL	GO:0042054~histone methyltransferase activity	1
GOTERM_MF_ALL	GO:0016279~protein-lysine N-methyltransferase activity	1
GOTERM_MF_ALL	GO:0016811~hydrolase activity, acting on carbon-nitrogen	1
GOTERM_MF_ALL	GO:0016775~phosphotransferase activity, nitrogenous gro	1
GOTERM_MF_ALL	GO:0042910~xenobiotic transporter activity	1
GOTERM_MF_ALL	GO:0016410~N-acyltransferase activity	1
GOTERM_MF_ALL	GO:0004612~phosphoenolpyruvate carboxykinase (ATP)	1
GOTERM_MF_ALL	GO:0016899~oxidoreductase activity, acting on the CH-O	1
GOTERM_MF_ALL	GO:0003746~translation elongation factor activity	1
GOTERM_MF_ALL	GO:0004467~long-chain-fatty-acid-CoA ligase activity	1
GOTERM_MF_ALL	GO:0008097~5S rRNA binding	1
GOTERM_MF_ALL	GO:0004645~phosphorylase activity	1
GOTERM_MF_ALL	GO:0016760~cellulose synthase (UDP-forming) activity	1
GOTERM_MF_ALL	GO:0070456~galactose-1-phosphate phosphatase activity	1
GOTERM_MF_ALL	GO:0050661~NADP or NADPH binding	1
GOTERM_MF_ALL	GO:0003777~microtubule motor activity	1
GOTERM_MF_ALL	GO:0046030~inositol trisphosphate phosphatase activity	1
GOTERM_MF_ALL	GO:0050377~UDP-glucose 4,6-dehydratase activity	1
GOTERM_MF_ALL	GO:0008443~phosphofructokinase activity	1
GOTERM_MF_ALL	GO:0004055~argininosuccinate synthase activity	1
GOTERM_MF_ALL	GO:0010181~FMN binding	1
GOTERM_MF_ALL	GO:0003885~D-arabinono-1,4-lactone oxidase activity	1
GOTERM_MF_ALL	GO:0005310~dicarboxylic acid transmembrane transporter	1
GOTERM_MF_ALL	GO:0016838~carbon-oxygen lyase activity, acting on phos	1
GOTERM_MF_ALL	GO:0001671~ATPase activator activity	1
GOTERM_MF_ALL	GO:0004143~diacylglycerol kinase activity	1
GOTERM_MF_ALL	GO:0004176~ATP-dependent peptidase activity	1
GOTERM_MF_ALL	GO:0016833~oxo-acid-lyase activity	1
GOTERM_MF_ALL	GO:0015116~sulfate transmembrane transporter activity	1
GOTERM_MF_ALL	GO:0005345~purine transmembrane transporter activity	1
GOTERM_MF_ALL	GO:0008810~cellulase activity	1
GOTERM_MF_ALL	GO:0016790~thiolester hydrolase activity	1
GOTERM_MF_ALL	GO:0004673~protein histidine kinase activity	1
GOTERM_MF_ALL	GO:0016717~oxidoreductase activity, acting on paired don	1
GOTERM_MF_ALL	GO:0003785~actin monomer binding	1
GOTERM_MF_ALL	GO:0016854~racemase and epimerase activity	1
GOTERM_MF_ALL	GO:0051738~xanthophyll binding	1
GOTERM_MF_ALL	GO:0003713~transcription coactivator activity	1
GOTERM_MF_ALL	GO:0042085~5-methyltetrahydropteroyltri-L-glutamate-de	1
GOTERM_MF_ALL	GO:0008200~ion channel inhibitor activity	1
GOTERM_MF_ALL	GO:0004402~histone acetyltransferase activity	1

GOTERM_MF_ALL	GO:0015645~fatty-acid ligase activity	1
GOTERM_MF_ALL	GO:0048040~UDP-glucuronate decarboxylase activity	1
GOTERM_MF_ALL	GO:0051184~cofactor transporter activity	1
GOTERM_MF_ALL	GO:0016628~oxidoreductase activity, acting on the CH-C	1
GOTERM_MF_ALL	GO:0004108~citrate (Si)-synthase activity	1
GOTERM_MF_ALL	GO:0030695~GTPase regulator activity	1
GOTERM_MF_ALL	GO:0005247~voltage-gated chloride channel activity	1
GOTERM_MF_ALL	GO:0019203~carbohydrate phosphatase activity	1
GOTERM_MF_ALL	GO:0000774~adenyl-nucleotide exchange factor activity	1
GOTERM_MF_ALL	GO:0005085~guanyl-nucleotide exchange factor activity	1
GOTERM_MF_ALL	GO:0015491~cation:cation antiporter activity	1
GOTERM_MF_ALL	GO:0033613~transcription activator binding	1
GOTERM_MF_ALL	GO:0031409~pigment binding	1
GOTERM_MF_ALL	GO:0016308~1-phosphatidylinositol-4-phosphate 5-kinase	1
GOTERM_MF_ALL	GO:0015300~solute:solute antiporter activity	1
GOTERM_MF_ALL	GO:0034595~phosphoinositide 5-phosphatase activity	1
GOTERM_MF_ALL	GO:0015298~solute:cation antiporter activity	1
GOTERM_MF_ALL	GO:0033840~NDP-glucose-starch glucosyltransferase acti	1
GOTERM_MF_ALL	GO:0016248~channel inhibitor activity	1
GOTERM_MF_ALL	GO:0004084~branched-chain-amino-acid transaminase act	1
GOTERM_MF_ALL	GO:0004372~glycine hydroxymethyltransferase activity	1
GOTERM_MF_ALL	GO:0005088~Ras guanyl-nucleotide exchange factor activ	1
GOTERM_MF_ALL	GO:0008559~xenobiotic-transporting ATPase activity	1
GOTERM_MF_ALL	GO:0080031~methyl salicylate esterase activity	1
GOTERM_MF_ALL	GO:0008865~fructokinase activity	1
GOTERM_MF_ALL	GO:0015299~solute:hydrogen antiporter activity	1
GOTERM_MF_ALL	GO:0070568~guanylyltransferase activity	1
GOTERM_MF_ALL	GO:0032549~ribonucleoside binding	1
GOTERM_MF_ALL	GO:0030410~nicotianamine synthase activity	1
GOTERM_MF_ALL	GO:0051015~actin filament binding	1
GOTERM_MF_ALL	GO:0016856~racemase and epimerase activity, acting on h	1
GOTERM_MF_ALL	GO:0008963~phospho-N-acetylmuramoyl-pentapeptide-tra	1
GOTERM_MF_ALL	GO:0008271~secondary active sulfate transmembrane tran	1
GOTERM_MF_ALL	GO:0005244~voltage-gated ion channel activity	1
GOTERM_MF_ALL	GO:0004331~fructose-2,6-bisphosphate 2-phosphatase acti	1
GOTERM_MF_ALL	GO:0045549~9-cis-epoxycarotenoid dioxygenase activity	1
GOTERM_MF_ALL	GO:0004611~phosphoenolpyruvate carboxykinase activity	1
GOTERM_MF_ALL	GO:0004396~hexokinase activity	1
GOTERM_MF_ALL	GO:0016759~cellulose synthase activity	1
GOTERM_MF_ALL	GO:0034768~(E)-beta-ocimene synthase activity	1
GOTERM_MF_ALL	GO:0008308~voltage-gated anion channel activity	1
GOTERM_MF_ALL	GO:0043021~ribonucleoprotein binding	1
GOTERM_MF_ALL	GO:0004451~isocitrate lyase activity	1
GOTERM_MF_ALL	GO:0008170~N-methyltransferase activity	1
GOTERM_MF_ALL	GO:0004567~beta-mannosidase activity	1
GOTERM_MF_ALL	GO:0005451~monovalent cation:hydrogen antiporter activ	1
GOTERM_MF_ALL	GO:0051766~inositol trisphosphate kinase activity	1

GOTERM_MF_ALL	GO:0016630~protochlorophyllide reductase activity	1
GOTERM_MF_ALL	GO:0004351~glutamate decarboxylase activity	1
GOTERM_MF_ALL	GO:0004221~ubiquitin thiolesterase activity	1
GOTERM_MF_ALL	GO:0010347~L-galactose-1-phosphate phosphatase activit	1
GOTERM_MF_ALL	GO:0003866~3-phosphoshikimate 1-carboxyvinyltransfera	1
GOTERM_MF_ALL	GO:0009011~starch synthase activity	1
GOTERM_MF_ALL	GO:0034485~phosphatidylinositol-3,4,5-trisphosphate 5-p	1
GOTERM_MF_ALL	GO:0047230~flavonol-3-O-glucoside L-rhamnosyltransfer	1
GOTERM_MF_ALL	GO:0019139~cytokinin dehydrogenase activity	1
GOTERM_MF_ALL	GO:0015662~ATPase activity, coupled to transmembrane	1
GOTERM_MF_ALL	GO:0001727~lipid kinase activity	1
GOTERM_MF_ALL	GO:0047325~inositol tetrakisphosphate 1-kinase activity	1
GOTERM_MF_ALL	GO:0004506~squalene monooxygenase activity	1
GOTERM_MF_ALL	GO:0009881~photoreceptor activity	1
GOTERM_MF_ALL	GO:0015932~nucleobase, nucleoside, nucleotide and nucle	1
GOTERM_MF_ALL	GO:0015326~cationic amino acid transmembrane transpor	1
GOTERM_MF_ALL	GO:0019840~isoprenoid binding	1
GOTERM_MF_ALL	GO:0016985~mannan endo-1,4-beta-mannosidase activity	1
GOTERM_MF_ALL	GO:0004445~inositol-polyphosphate 5-phosphatase activit	1
GOTERM_MF_ALL	GO:0046481~digalactosyldiacylglycerol synthase activity	1
GOTERM_MF_ALL	GO:0010329~auxin efflux transmembrane transporter activ	1
GOTERM_MF_ALL	GO:0034594~phosphatidylinositol trisphosphate phosphata	1
GOTERM_MF_ALL	GO:0003711~transcription elongation regulator activity	1
GOTERM_MF_ALL	GO:0015239~multidrug transporter activity	1
GOTERM_MF_ALL	GO:0005375~copper ion transmembrane transporter activit	1
GOTERM_MF_ALL	GO:0045431~flavonol synthase activity	1
GOTERM_MF_ALL	GO:0004332~fructose-bisphosphate aldolase activity	1
GOTERM_MF_ALL	GO:0016984~ribulose-bisphosphate carboxylase activity	1
GOTERM_MF_ALL	GO:0004834~tryptophan synthase activity	1
GOTERM_MF_ALL	GO:0016278~lysine N-methyltransferase activity	1
GOTERM_MF_ALL	GO:0008252~nucleotidase activity	1
GOTERM_MF_ALL	GO:0008809~carnitine racemase activity	1
GOTERM_MF_ALL	GO:0016842~amidine-lyase activity	1
GOTERM_MF_ALL	GO:0004044~amidophosphoribosyltransferase activity	1
GOTERM_MF_ALL	GO:0016210~naringenin-chalcone synthase activity	1
GOTERM_MF_ALL	GO:0016641~oxidoreductase activity, acting on the CH-N	1
GOTERM_MF_ALL	GO:0008970~phospholipase A1 activity	1
GOTERM_MF_ALL	GO:0016846~carbon-sulfur lyase activity	1
GOTERM_MF_ALL	GO:0008047~enzyme activator activity	1
GOTERM_MF_ALL	GO:0042084~5-methyltetrahydrofolate-dependent methyltr	1
GOTERM_MF_ALL	GO:0008237~metallopeptidase activity	1
GOTERM_MF_ALL	GO:0016860~intramolecular oxidoreductase activity	1
GOTERM_MF_ALL	GO:0009982~pseudouridine synthase activity	1
GOTERM_MF_ALL	GO:0016681~oxidoreductase activity, acting on diphenols	1
GOTERM_MF_ALL	GO:0015556~C4-dicarboxylate transmembrane transporter	1
GOTERM_MF_ALL	GO:0016407~acetyltransferase activity	1
GOTERM_MF_ALL	GO:0043168~anion binding	1

GOTERM_MF_ALL	GO:0016679~oxidoreductase activity, acting on diphenols	1
GOTERM_MF_ALL	GO:0046873~metal ion transmembrane transporter activity	1
GOTERM_MF_ALL	GO:0010177~methylthioalkylmalate synthase activity	1
GOTERM_MF_ALL	GO:0022832~voltage-gated channel activity	1
GOTERM_MF_ALL	GO:0004028~3-chloroallyl aldehyde dehydrogenase activit	1
GOTERM_MF_ALL	GO:0051746~thalianol synthase activity	1
GOTERM_MF_ALL	GO:0016247~channel regulator activity	1
GOTERM_MF_ALL	GO:0009882~blue light photoreceptor activity	1
GOTERM_MF_ALL	GO:0016161~beta-amylase activity	1
GOTERM_MF_ALL	GO:0008381~mechanically-gated ion channel activity	1
GOTERM_MF_ALL	GO:0015232~heme transporter activity	1
GOTERM_MF_ALL	GO:0016840~carbon-nitrogen lyase activity	1
GOTERM_MF_ALL	GO:0016847~1-aminocyclopropane-1-carboxylate synthas	1
GOTERM_MF_ALL	GO:0004014~adenosylmethionine decarboxylase activity	1
GOTERM_MF_ALL	GO:0008429~phosphatidylethanolamine binding	1
GOTERM_MF_ALL	GO:0004747~ribokinase activity	1
GOTERM_MF_ALL	GO:0008783~agmatinase activity	1
GOTERM_MF_ALL	GO:0008441~3'(2'),5'-bisphosphate nucleotidase activity	1
GOTERM_MF_ALL	GO:0003999~adenine phosphoribosyltransferase activity	1
GOTERM_MF_ALL	GO:0009672~auxin:hydrogen symporter activity	1
GOTERM_MF_ALL	GO:0018024~histone-lysine N-methyltransferase activity	1
GOTERM_MF_ALL	GO:0046480~galactolipid galactosyltransferase activity	1
GOTERM_MF_ALL	GO:0043813~phosphatidylinositol-3,5-bisphosphate 5-pho	1
GOTERM_MF_ALL	GO:0004053~arginase activity	1
GOTERM_MF_ALL	GO:0034593~phosphatidylinositol bisphosphate phosphata	1
GOTERM_MF_ALL	GO:0015140~malate transmembrane transporter activity	1
GOTERM_MF_ALL	GO:0003852~2-isopropylmalate synthase activity	1
GOTERM_MF_ALL	GO:0010180~thioglucosidase binding	1
GOTERM_MF_ALL	GO:0017050~D-erythro-sphingosine kinase activity	1
GOTERM_MF_ALL	GO:0003680~AT DNA binding	1
GOTERM_MF_ALL	GO:0004022~alcohol dehydrogenase (NAD) activity	1
GOTERM_MF_ALL	GO:0004468~lysine N-acetyltransferase activity	1
GOTERM_MF_ALL	GO:0005083~small GTPase regulator activity	1
GOTERM_MF_ALL	GO:0016780~phosphotransferase activity, for other substit	1
GOTERM_MF_ALL	GO:0042973~glucan endo-1,3-beta-D-glucosidase activity	1
GOTERM_MF_ALL	GO:0031404~chloride ion binding	1
GOTERM_MF_ALL	GO:0003701~RNA polymerase I transcription factor activi	1
GOTERM_MF_ALL	GO:0080019~fatty acyl-CoA reductase (alcohol-forming) a	1
GOTERM_MF_ALL	GO:0005253~anion channel activity	1
GOTERM_MF_ALL	GO:0046933~hydrogen ion transporting ATP synthase acti	1
GOTERM_MF_ALL	GO:0005200~structural constituent of cytoskeleton	1
GOTERM_MF_ALL	GO:0015174~basic amino acid transmembrane transporter	1
GOTERM_MF_ALL	GO:0022884~macromolecule transmembrane transporter a	1
GOTERM_MF_ALL	GO:0047714~galactolipase activity	1
GOTERM_MF_ALL	GO:0003843~1,3-beta-glucan synthase activity	1
GOTERM_MF_ALL	GO:0015089~high affinity copper ion transmembrane trans	1
GOTERM_MF_ALL	GO:0016813~hydrolase activity, acting on carbon-nitrogen	1

GOTERM_MF_ALL	GO:0042409~caffeoyl-CoA O-methyltransferase activity	1
GOTERM_MF_ALL	GO:0008172~S-methyltransferase activity	1
GOTERM_MF_ALL	GO:0051765~inositol tetrakisphosphate kinase activity	1
GOTERM_MF_ALL	GO:0010178~IAA-amino acid conjugate hydrolase activity	1
GOTERM_MF_ALL	GO:0050113~inositol oxygenase activity	1
GOTERM_MF_ALL	GO:0016638~oxidoreductase activity, acting on the CH-N	1
GOTERM_MF_ALL	GO:0031957~very-long-chain-fatty-acid-CoA ligase activit	1
GOTERM_MF_ALL	GO:0008320~protein transmembrane transporter activity	1
GOTERM_MF_ALL	GO:0008080~N-acetyltransferase activity	1
GOTERM_MF_ALL	GO:0005254~chloride channel activity	1
GOTERM_MF_ALL	GO:0015385~sodium:hydrogen antiporter activity	1
GOTERM_MF_ALL	GO:0008083~growth factor activity	1
GOTERM_MF_ALL	GO:0004439~phosphatidylinositol-4,5-bisphosphate 5-pho	1
GOTERM_MF_ALL	GO:0031177~phosphopantetheine binding	1
GOTERM_MF_ALL	GO:0042300~beta-amyrin synthase activity	1
GOTERM_MF_ALL	GO:0016863~intramolecular oxidoreductase activity, trans	1
GOTERM_MF_ALL	GO:0030955~potassium ion binding	1
GOTERM_MF_ALL	GO:0080118~brassinosteroid sulfotransferase activity	1
GOTERM_MF_ALL	GO:0016160~amylase activity	1
GOTERM_MF_ALL	GO:0008276~protein methyltransferase activity	1
GOTERM_MF_ALL	GO:0008705~methionine synthase activity	1
GOTERM_MF_ALL	GO:0015562~efflux transmembrane transporter activity	1
GOTERM_MF_ALL	GO:0016621~cinnamoyl-CoA reductase activity	1
GOTERM_MF_ALL	GO:0004029~aldehyde dehydrogenase (NAD) activity	1
GOTERM_MF_ALL	GO:0008017~microtubule binding	1
GOTERM_MF_ALL	GO:0035300~inositol-1,3,4-trisphosphate 5/6-kinase activi	1
GOTERM_MF_ALL	GO:0004620~phospholipase activity	1
GOTERM_MF_ALL	GO:0015631~tubulin binding	1
GOTERM_MF_ALL	GO:0010475~galactose-1-phosphate guanylyltransferase (	1
GOTERM_MF_ALL	GO:0031420~alkali metal ion binding	1
GOTERM_MF_ALL	GO:0000155~two-component sensor activity	1
GOTERM_MF_ALL	GO:0004521~endoribonuclease activity	1
GOTERM_MF_ALL	GO:0004165~dodecenoyl-CoA delta-isomerase activity	1
GOTERM_MF_ALL	GO:0015205~nucleobase transmembrane transporter activi	1
GOTERM_MF_ALL	GO:0033764~steroid dehydrogenase activity, acting on the	1
GOTERM_MF_ALL	GO:0008553~hydrogen-exporting ATPase activity, phosph	1
GOTERM_MF_ALL	GO:0031127~alpha(1,2)-fucosyltransferase activity	1
GOTERM_MF_ALL	GO:0008146~sulfotransferase activity	1
GOTERM_MF_ALL	GO:0043022~ribosome binding	1
GOTERM_MF_ALL	GO:0080048~GDP-D-glucose phosphorylase activity	1
GOTERM_MF_ALL	GO:0015925~galactosidase activity	1
GOTERM_MF_ALL	GO:0005089~Rho guanyl-nucleotide exchange factor activ	1
GOTERM_MF_ALL	GO:0016688~L-ascorbate peroxidase activity	1
GOTERM_MF_ALL	GO:0008107~galactoside 2-alpha-L-fucosyltransferase acti	1
GOTERM_MF_ALL	GO:0051739~ammonia transporter activity	1

Down-regulated genes of *ein* 2 grouped into different metabolic pathways (KEGG and DAVID analysis).

Category	Term	Gene Count
KEGG_PATHWAY	ath00680:Methane metabolism	17
KEGG_PATHWAY	ath00940:Phenylpropanoid biosynthesis	18
KEGG_PATHWAY	ath00360:Phenylalanine metabolism	15
KEGG_PATHWAY	ath01061:Biosynthesis of phenylpropanoids	25
PANTHER_PATHWA	P00006:Apoptosis signaling pathway	6
KEGG_PATHWAY	ath00561:Glycerolipid metabolism	4
KEGG_PATHWAY	ath00941:Flavonoid biosynthesis	4
KEGG_PATHWAY	ath03018:RNA degradation	6
KEGG_PATHWAY	ath04144:Endocytosis	7
PANTHER_PATHWA	P00049:Parkinson disease	6
KEGG_PATHWAY	ath00053:Ascorbate and aldarate metabolism	4
PANTHER_PATHWA	P00029:Huntington disease	5
KEGG_PATHWAY	ath00966:Glucosinolate biosynthesis	3
KEGG_PATHWAY	ath00562:Inositol phosphate metabolism	4
KEGG_PATHWAY	ath00250:Alanine, aspartate and glutamate metabolism	4
PANTHER_PATHWA	P00016:Cytoskeletal regulation by Rho GTPase	3
KEGG_PATHWAY	ath03040:Spliceosome	7
KEGG_PATHWAY	ath00010:Glycolysis / Gluconeogenesis	6
KEGG_PATHWAY	ath00901:Indole alkaloid biosynthesis	2
PANTHER_PATHWA	P00044:Nicotinic acetylcholine receptor signaling pathway	2
KEGG_PATHWAY	ath00630:Glyoxylate and dicarboxylate metabolism	3
KEGG_PATHWAY	ath00071:Fatty acid metabolism	3
KEGG_PATHWAY	ath00410:beta-Alanine metabolism	2
KEGG_PATHWAY	ath00196:Photosynthesis	2
KEGG_PATHWAY	ath00051:Fructose and mannose metabolism	3
PANTHER_PATHWA	P00032:Insulin/IGF pathway-mitogen activated protein kin	2
KEGG_PATHWAY	ath00650:Butanoate metabolism	2
KEGG_PATHWAY	ath00330:Arginine and proline metabolism	3
PANTHER_PATHWA	P02738:De novo purine biosynthesis	2
KEGG_PATHWAY	ath00460:Cyanoamino acid metabolism	2
KEGG_PATHWAY	ath00030:Pentose phosphate pathway	3
KEGG_PATHWAY	ath00230:Purine metabolism	5
KEGG_PATHWAY	ath00130:Ubiquinone and other terpenoid-quinone biosynt	2
KEGG_PATHWAY	ath03020:RNA polymerase	2
KEGG_PATHWAY	ath04712:Circadian rhythm	2
KEGG_PATHWAY	ath00620:Pyruvate metabolism	3
KEGG_PATHWAY	ath00380:Tryptophan metabolism	2
KEGG_PATHWAY	ath04070:Phosphatidylinositol signaling system	2
KEGG_PATHWAY	ath00400:Phenylalanine, tyrosine and tryptophan biosynthe	2
KEGG_PATHWAY	ath01065:Biosynthesis of alkaloids derived from histidine	6
KEGG_PATHWAY	ath00710:Carbon fixation in photosynthetic organisms	3
KEGG_PATHWAY	ath00260:Glycine, serine and threonine metabolism	2
KEGG_PATHWAY	ath01063:Biosynthesis of alkaloids derived from shikimate	6
KEGG_PATHWAY	ath03010:Ribosome	8
PANTHER_PATHWA	P00018:EGF receptor signaling pathway	2
PANTHER_PATHWA	P00021:FGF signaling pathway	2

KEGG_PATHWAY	ath00500:Starch and sucrose metabolism	3
KEGG_PATHWAY	ath00020:Citrate cycle (TCA cycle)	2
KEGG_PATHWAY	ath00903:Limonene and pinene degradation	2
KEGG_PATHWAY	ath00240:Pyrimidine metabolism	2
KEGG_PATHWAY	ath00520:Amino sugar and nucleotide sugar metabolism	2
KEGG_PATHWAY	ath00195:Photosynthesis	2
KEGG_PATHWAY	ath01070:Biosynthesis of plant hormones	8
KEGG_PATHWAY	ath00190:Oxidative phosphorylation	3
KEGG_PATHWAY	ath04120:Ubiquitin mediated proteolysis	2
KEGG_PATHWAY	ath01062:Biosynthesis of terpenoids and steroids	4
KEGG_PATHWAY	ath01066:Biosynthesis of alkaloids derived from terpenoid	3
KEGG_PATHWAY	ath01064:Biosynthesis of alkaloids derived from ornithine,	2
KEGG_PATHWAY	ath00270:Cysteine and methionine metabolism	1
KEGG_PATHWAY	ath00350:Tyrosine metabolism	1
KEGG_PATHWAY	ath00670:One carbon pool by folate	1
KEGG_PATHWAY	ath00944:Flavone and flavonol biosynthesis	1
KEGG_PATHWAY	ath00906:Carotenoid biosynthesis	1
KEGG_PATHWAY	ath00908:Zeatin biosynthesis	1
KEGG_PATHWAY	ath00564:Glycerophospholipid metabolism	1
KEGG_PATHWAY	ath00480:Glutathione metabolism	1
KEGG_PATHWAY	ath00902:Monoterpenoid biosynthesis	1
KEGG_PATHWAY	ath00100:Steroid biosynthesis	1
KEGG_PATHWAY	ath00280:Valine, leucine and isoleucine degradation	1
KEGG_PATHWAY	ath00061:Fatty acid biosynthesis	1
KEGG_PATHWAY	ath00523:Polyketide sugar unit biosynthesis	1
KEGG_PATHWAY	ath00640:Propanoate metabolism	1
KEGG_PATHWAY	ath00340:Histidine metabolism	1
KEGG_PATHWAY	ath00980:Metabolism of xenobiotics by cytochrome P450	1
KEGG_PATHWAY	ath03420:Nucleotide excision repair	1
KEGG_PATHWAY	ath00945:Stilbenoid, diarylheptanoid and gingerol biosynth	1
KEGG_PATHWAY	ath00290:Valine, leucine and isoleucine biosynthesis	1
KEGG_PATHWAY	ath00310:Lysine degradation	1
KEGG_PATHWAY	ath00430:Taurine and hypotaurine metabolism	1
KEGG_PATHWAY	ath01040:Biosynthesis of unsaturated fatty acids	1
PANTHER_PATHWA	P00051:TCA cycle	1
PANTHER_PATHWA	P02776:Serine glycine biosynthesis	1
PANTHER_PATHWA	P00007:Axon guidance mediated by semaphorins	1
PANTHER_PATHWA	P00034:Integrin signalling pathway	1
PANTHER_PATHWA	P00025:Hedgehog signaling pathway	1
PANTHER_PATHWA	P00053:T cell activation	1
PANTHER_PATHWA	P02785:Valine biosynthesis	1
PANTHER_PATHWA	P00045:Notch signaling pathway	1
PANTHER_PATHWA	P00008:Axon guidance mediated by Slit/Robo	1
PANTHER_PATHWA	P02783:Tryptophan biosynthesis	1
PANTHER_PATHWA	P02724:Alanine biosynthesis	1
PANTHER_PATHWA	P00031:Inflammation mediated by chemokine and cytokin	1
PANTHER_PATHWA	P04393:Ras Pathway	1

PANTHER_PATHWA	P02772:Pyruvate metabolism	1
PANTHER_PATHWA	P02734:Chorismate biosynthesis	1
PANTHER_PATHWA	P00010:B cell activation	1
PANTHER_PATHWA	P00009:Axon guidance mediated by netrin	1
PANTHER_PATHWA	P00046:Oxidative stress response	1
PANTHER_PATHWA	P00022:General transcription by RNA polymerase I	1
PANTHER_PATHWA	P00047:PDGF signaling pathway	1
PANTHER_PATHWA	P00030:Hypoxia response via HIF activation	1
PANTHER_PATHWA	P05918:p38 MAPK pathway	1
PANTHER_PATHWA	P02721:ATP synthesis	1
PANTHER_PATHWA	P02763:Peptidoglycan biosynthesis	1
PANTHER_PATHWA	P00059:p53 pathway	1
PANTHER_PATHWA	P00001:Adrenaline and noradrenaline biosynthesis	1
PANTHER_PATHWA	P02749:Leucine biosynthesis	1
PANTHER_PATHWA	P00057:Wnt signaling pathway	1
PANTHER_PATHWA	P05912:Dopamine receptor mediated signaling pathway	1
PANTHER_PATHWA	P02728:Arginine biosynthesis	1
PANTHER_PATHWA	P02748:Isoleucine biosynthesis	1

Supplementary Table S6. Differentially expressed genes grouped in different functional and pathways categories.

Up-regulated genes of *aba* 1.6 grouped in different cellular functions categories (DAVID analysis)

Category	Term	Gene Count
GOTERM_BP_ALL	GO:0010033~response to organic substance	112
GOTERM_BP_ALL	GO:0009725~response to hormone stimulus	92
GOTERM_BP_ALL	GO:0009719~response to endogenous stimulus	94
GOTERM_CC_ALL	GO:0012505~endomembrane system	289
GOTERM_BP_ALL	GO:0042221~response to chemical stimulus	147
GOTERM_BP_ALL	GO:0010817~regulation of hormone levels	19
GOTERM_CC_ALL	GO:0030312~external encapsulating structure	58
GOTERM_MF_ALL	GO:0004180~carboxypeptidase activity	13
GOTERM_MF_ALL	GO:0004872~receptor activity	54
GOTERM_MF_ALL	GO:0004185~serine-type carboxypeptidase activity	12
GOTERM_MF_ALL	GO:0070008~serine-type exopeptidase activity	12
GOTERM_MF_ALL	GO:0004674~protein serine/threonine kinase activity	80
GOTERM_CC_ALL	GO:0031224~intrinsic to membrane	187
GOTERM_MF_ALL	GO:0060089~molecular transducer activity	66
GOTERM_MF_ALL	GO:0004871~signal transducer activity	66
GOTERM_CC_ALL	GO:0005618~cell wall	56
GOTERM_MF_ALL	GO:0004672~protein kinase activity	88
GOTERM_BP_ALL	GO:0065008~regulation of biological quality	58
GOTERM_BP_ALL	GO:0006468~protein amino acid phosphorylation	86
GOTERM_MF_ALL	GO:0016301~kinase activity	105
GOTERM_BP_ALL	GO:0009733~response to auxin stimulus	35
GOTERM_CC_ALL	GO:0016021~integral to membrane	155
GOTERM_MF_ALL	GO:0004713~protein tyrosine kinase activity	32
GOTERM_BP_ALL	GO:0048589~developmental growth	22
GOTERM_BP_ALL	GO:0030001~metal ion transport	27
GOTERM_MF_ALL	GO:0016773~phosphotransferase activity, alcohol group a	93
GOTERM_BP_ALL	GO:0050896~response to stimulus	249
GOTERM_BP_ALL	GO:0006793~phosphorus metabolic process	94
GOTERM_BP_ALL	GO:0048878~chemical homeostasis	16
GOTERM_BP_ALL	GO:0006796~phosphate metabolic process	94
GOTERM_BP_ALL	GO:0016310~phosphorylation	88
GOTERM_BP_ALL	GO:0060560~developmental growth involved in morphog	19
GOTERM_BP_ALL	GO:0009826~unidimensional cell growth	19
GOTERM_MF_ALL	GO:0016798~hydrolase activity, acting on glycosyl bonds	40
GOTERM_BP_ALL	GO:0009755~hormone-mediated signaling	39
GOTERM_BP_ALL	GO:0032870~cellular response to hormone stimulus	39
GOTERM_MF_ALL	GO:0008236~serine-type peptidase activity	20
GOTERM_MF_ALL	GO:0017171~serine hydrolase activity	20
GOTERM_BP_ALL	GO:0008361~regulation of cell size	24
GOTERM_BP_ALL	GO:0009926~auxin polar transport	9
GOTERM_BP_ALL	GO:0016049~cell growth	23
GOTERM_CC_ALL	GO:0044425~membrane part	209
GOTERM_CC_ALL	GO:0005773~vacuole	53
GOTERM_BP_ALL	GO:0042547~cell wall modification during multidimensio	7

GOTERM_MF_ALL	GO:0008238~exopeptidase activity	13
GOTERM_CC_ALL	GO:0009505~plant-type cell wall	27
GOTERM_BP_ALL	GO:0010876~lipid localization	19
GOTERM_BP_ALL	GO:0065007~biological regulation	259
GOTERM_BP_ALL	GO:0009914~hormone transport	9
GOTERM_BP_ALL	GO:0040007~growth	25
GOTERM_MF_ALL	GO:0003700~transcription factor activity	115
GOTERM_BP_ALL	GO:0007169~transmembrane receptor protein tyrosine ki	16
GOTERM_BP_ALL	GO:0007167~enzyme linked receptor protein signaling pa	16
GOTERM_MF_ALL	GO:0004553~hydrolase activity, hydrolyzing O-glycosyl	37
GOTERM_BP_ALL	GO:0042446~hormone biosynthetic process	8
GOTERM_MF_ALL	GO:0016772~transferase activity, transferring phosphorus	109
GOTERM_BP_ALL	GO:0042445~hormone metabolic process	11
GOTERM_BP_ALL	GO:0032535~regulation of cellular component size	24
GOTERM_MF_ALL	GO:0020037~heme binding	33
GOTERM_BP_ALL	GO:0051179~localization	143
GOTERM_MF_ALL	GO:0003824~catalytic activity	495
GOTERM_BP_ALL	GO:0009723~response to ethylene stimulus	25
GOTERM_BP_ALL	GO:0010227~floral organ abscission	5
GOTERM_BP_ALL	GO:0009751~response to salicylic acid stimulus	17
GOTERM_MF_ALL	GO:0030528~transcription regulator activity	127
GOTERM_BP_ALL	GO:0000160~two-component signal transduction system (	22
GOTERM_BP_ALL	GO:0007166~cell surface receptor linked signal transduct	18
GOTERM_BP_ALL	GO:0048569~post-embryonic organ development	19
GOTERM_BP_ALL	GO:0006464~protein modification process	110
GOTERM_BP_ALL	GO:0009664~plant-type cell wall organization	11
GOTERM_BP_ALL	GO:0000902~cell morphogenesis	21
GOTERM_BP_ALL	GO:0016132~brassinosteroid biosynthetic process	4
GOTERM_BP_ALL	GO:0016129~phytosteroid biosynthetic process	4
GOTERM_BP_ALL	GO:0009828~plant-type cell wall loosening	7
GOTERM_BP_ALL	GO:0051716~cellular response to stimulus	71
GOTERM_MF_ALL	GO:0005509~calcium ion binding	34
GOTERM_BP_ALL	GO:0009838~abscission	5
GOTERM_BP_ALL	GO:0009831~plant-type cell wall modification during mu	6
GOTERM_BP_ALL	GO:0043687~post-translational protein modification	97
GOTERM_BP_ALL	GO:0006811~ion transport	40
GOTERM_MF_ALL	GO:0004497~monooxygenase activity	28
GOTERM_BP_ALL	GO:0009739~response to gibberellin stimulus	14
GOTERM_MF_ALL	GO:0019825~oxygen binding	22
GOTERM_MF_ALL	GO:0042562~hormone binding	5
GOTERM_BP_ALL	GO:0006869~lipid transport	16
GOTERM_BP_ALL	GO:0002237~response to molecule of bacterial origin	4
GOTERM_BP_ALL	GO:0009814~defense response, incompatible interaction	12
GOTERM_MF_ALL	GO:0047215~indole-3-acetate beta-glucosyltransferase ac	3
GOTERM_BP_ALL	GO:0009606~tropism	8
GOTERM_MF_ALL	GO:0046906~tetrapyrrole binding	33
GOTERM_MF_ALL	GO:0015075~ion transmembrane transporter activity	44

GOTERM_CC_ALL	GO:0031225~anchored to membrane	28
GOTERM_BP_ALL	GO:0007242~intracellular signaling cascade	58
GOTERM_BP_ALL	GO:0030005~cellular di-, tri-valent inorganic cation home	8
GOTERM_BP_ALL	GO:0010105~negative regulation of ethylene mediated sig	4
GOTERM_BP_ALL	GO:0070298~negative regulation of two-component signa	4
GOTERM_MF_ALL	GO:0005216~ion channel activity	11
GOTERM_BP_ALL	GO:0051234~establishment of localization	135
GOTERM_MF_ALL	GO:0022857~transmembrane transporter activity	69
GOTERM_MF_ALL	GO:0016740~transferase activity	181
GOTERM_BP_ALL	GO:0007165~signal transduction	83
GOTERM_MF_ALL	GO:0005515~protein binding	224
GOTERM_BP_ALL	GO:0006810~transport	134
GOTERM_BP_ALL	GO:0009968~negative regulation of signal transduction	7
GOTERM_BP_ALL	GO:0010648~negative regulation of cell communication	7
GOTERM_MF_ALL	GO:0022836~gated channel activity	9
GOTERM_MF_ALL	GO:0015293~symporter activity	15
GOTERM_BP_ALL	GO:0009629~response to gravity	7
GOTERM_CC_ALL	GO:0005576~extracellular region	84
GOTERM_MF_ALL	GO:0051740~ethylene binding	3
GOTERM_BP_ALL	GO:0032989~cellular component morphogenesis	21
GOTERM_BP_ALL	GO:0009605~response to external stimulus	29
GOTERM_BP_ALL	GO:0010104~regulation of ethylene mediated signaling p	4
GOTERM_BP_ALL	GO:0070297~regulation of two-component signal transdu	4
GOTERM_BP_ALL	GO:0009827~plant-type cell wall modification	7
GOTERM_BP_ALL	GO:0055066~di-, tri-valent inorganic cation homeostasis	8
GOTERM_BP_ALL	GO:0006800~oxygen and reactive oxygen species metabo	12
GOTERM_MF_ALL	GO:0009672~auxin:hydrogen symporter activity	4
GOTERM_MF_ALL	GO:0022804~active transmembrane transporter activity	44
GOTERM_BP_ALL	GO:0009653~anatomical structure morphogenesis	36
GOTERM_BP_ALL	GO:0006949~syncytium formation	4
GOTERM_BP_ALL	GO:0051651~maintenance of location in cell	4
GOTERM_BP_ALL	GO:0009825~multidimensional cell growth	7
GOTERM_BP_ALL	GO:0048437~floral organ development	13
GOTERM_MF_ALL	GO:0015294~solute:cation symporter activity	13
GOTERM_BP_ALL	GO:0010268~brassinosteroid homeostasis	3
GOTERM_MF_ALL	GO:0016491~oxidoreductase activity	97
GOTERM_MF_ALL	GO:0015291~secondary active transmembrane transporter	25
GOTERM_MF_ALL	GO:0022891~substrate-specific transmembrane transporte	55
GOTERM_BP_ALL	GO:0045088~regulation of innate immune response	6
GOTERM_BP_ALL	GO:0006721~terpenoid metabolic process	11
GOTERM_MF_ALL	GO:0030246~carbohydrate binding	19
GOTERM_MF_ALL	GO:0004673~protein histidine kinase activity	5
GOTERM_MF_ALL	GO:0000155~two-component sensor activity	5
GOTERM_MF_ALL	GO:0016775~phosphotransferase activity, nitrogenous gr	5
GOTERM_BP_ALL	GO:0009873~ethylene mediated signaling pathway	16
GOTERM_BP_ALL	GO:0016128~phytosteroid metabolic process	4
GOTERM_BP_ALL	GO:0016131~brassinosteroid metabolic process	4

GOTERM_MF_ALL	GO:0022892~substrate-specific transporter activity	62
GOTERM_MF_ALL	GO:0005215~transporter activity	83
GOTERM_BP_ALL	GO:0043412~biopolymer modification	113
GOTERM_MF_ALL	GO:0030570~pectate lyase activity	5
GOTERM_MF_ALL	GO:0016837~carbon-oxygen lyase activity, acting on poly	5
GOTERM_BP_ALL	GO:0007047~cell wall organization	23
GOTERM_MF_ALL	GO:0005516~calmodulin binding	16
GOTERM_BP_ALL	GO:0009908~flower development	20
GOTERM_MF_ALL	GO:0035252~UDP-xylosyltransferase activity	3
GOTERM_BP_ALL	GO:0009416~response to light stimulus	35
GOTERM_CC_ALL	GO:0048196~middle lamella-containing extracellular mat	3
GOTERM_BP_ALL	GO:0009686~gibberellin biosynthetic process	4
GOTERM_BP_ALL	GO:0051235~maintenance of location	4
GOTERM_MF_ALL	GO:0005506~iron ion binding	50
GOTERM_BP_ALL	GO:0009630~gravitropism	6
GOTERM_BP_ALL	GO:0016102~diterpenoid biosynthetic process	4
GOTERM_MF_ALL	GO:0016709~oxidoreductase activity, acting on paired do	7
GOTERM_BP_ALL	GO:0050776~regulation of immune response	6
GOTERM_BP_ALL	GO:0002682~regulation of immune system process	6
GOTERM_BP_ALL	GO:0006350~transcription	85
GOTERM_BP_ALL	GO:0009966~regulation of signal transduction	12
GOTERM_BP_ALL	GO:0009938~negative regulation of gibberellic acid medi	3
GOTERM_BP_ALL	GO:0045449~regulation of transcription	126
GOTERM_BP_ALL	GO:0006812~cation transport	29
GOTERM_BP_ALL	GO:0050801~ion homeostasis	10
GOTERM_BP_ALL	GO:0002218~activation of innate immune response	5
GOTERM_BP_ALL	GO:0002253~activation of immune response	5
GOTERM_BP_ALL	GO:0045089~positive regulation of innate immune respon	5
GOTERM_BP_ALL	GO:0002684~positive regulation of immune system proce	5
GOTERM_BP_ALL	GO:0009863~salicylic acid mediated signaling pathway	5
GOTERM_BP_ALL	GO:0050778~positive regulation of immune response	5
GOTERM_BP_ALL	GO:0008202~steroid metabolic process	8
GOTERM_CC_ALL	GO:0044421~extracellular region part	6
GOTERM_BP_ALL	GO:0009314~response to radiation	35
GOTERM_MF_ALL	GO:0022838~substrate specific channel activity	12
GOTERM_MF_ALL	GO:0009055~electron carrier activity	45
GOTERM_MF_ALL	GO:0016564~transcription repressor activity	7
GOTERM_BP_ALL	GO:0055080~cation homeostasis	9
GOTERM_BP_ALL	GO:0048583~regulation of response to stimulus	14
GOTERM_BP_ALL	GO:0010646~regulation of cell communication	12
GOTERM_MF_ALL	GO:0015267~channel activity	12
GOTERM_MF_ALL	GO:0022803~passive transmembrane transporter activity	12
GOTERM_MF_ALL	GO:0016705~oxidoreductase activity, acting on paired do	17
GOTERM_BP_ALL	GO:0055082~cellular chemical homeostasis	9
GOTERM_BP_ALL	GO:0045229~external encapsulating structure organizatio	23
GOTERM_BP_ALL	GO:0007389~pattern specification process	11
GOTERM_BP_ALL	GO:0030003~cellular cation homeostasis	8

GOTERM_BP_ALL	GO:0009687~abscisic acid metabolic process	4
GOTERM_BP_ALL	GO:0043288~apocarotenoid metabolic process	4
GOTERM_BP_ALL	GO:0009410~response to xenobiotic stimulus	3
GOTERM_BP_ALL	GO:0031349~positive regulation of defense response	5
GOTERM_BP_ALL	GO:0015833~peptide transport	8
GOTERM_BP_ALL	GO:0006857~oligopeptide transport	8
GOTERM_BP_ALL	GO:0048869~cellular developmental process	32
GOTERM_BP_ALL	GO:0019219~regulation of nucleobase, nucleoside, nucle	126
GOTERM_MF_ALL	GO:0030247~polysaccharide binding	4
GOTERM_MF_ALL	GO:0001871~pattern binding	4
GOTERM_BP_ALL	GO:0005976~polysaccharide metabolic process	18
GOTERM_BP_ALL	GO:0050794~regulation of cellular process	204
GOTERM_BP_ALL	GO:0042744~hydrogen peroxide catabolic process	9
GOTERM_BP_ALL	GO:0070301~cellular response to hydrogen peroxide	9
GOTERM_BP_ALL	GO:0006629~lipid metabolic process	52
GOTERM_BP_ALL	GO:0009740~gibberellic acid mediated signaling	5
GOTERM_BP_ALL	GO:0009627~systemic acquired resistance	5
GOTERM_BP_ALL	GO:0010476~gibberellin-mediated signaling	5
GOTERM_MF_ALL	GO:0046873~metal ion transmembrane transporter activit	13
GOTERM_MF_ALL	GO:0016755~transferase activity, transferring amino-acyl	3
GOTERM_MF_ALL	GO:0042285~xylosyltransferase activity	3
GOTERM_BP_ALL	GO:0055114~oxidation reduction	76
GOTERM_MF_ALL	GO:0016762~xyloglucan:xyloglucosyl transferase activity	5
GOTERM_BP_ALL	GO:0048523~negative regulation of cellular process	16
GOTERM_MF_ALL	GO:0008131~amine oxidase activity	4
GOTERM_BP_ALL	GO:0010223~secondary shoot formation	3
GOTERM_BP_ALL	GO:0010346~shoot formation	3
GOTERM_BP_ALL	GO:0006650~glycerophospholipid metabolic process	7
GOTERM_BP_ALL	GO:0006486~protein amino acid glycosylation	7
GOTERM_BP_ALL	GO:0009101~glycoprotein biosynthetic process	7
GOTERM_BP_ALL	GO:0043413~biopolymer glycosylation	7
GOTERM_BP_ALL	GO:0070085~glycosylation	7
GOTERM_MF_ALL	GO:0008289~lipid binding	19
GOTERM_BP_ALL	GO:0051171~regulation of nitrogen compound metabolic	126
GOTERM_BP_ALL	GO:0009685~gibberellin metabolic process	4
GOTERM_MF_ALL	GO:0000249~C-22 sterol desaturase activity	2
GOTERM_MF_ALL	GO:0004854~xanthine dehydrogenase activity	2
GOTERM_BP_ALL	GO:0042542~response to hydrogen peroxide	11
GOTERM_BP_ALL	GO:0009734~auxin mediated signaling pathway	11
GOTERM_BP_ALL	GO:0042743~hydrogen peroxide metabolic process	9
GOTERM_BP_ALL	GO:0009628~response to abiotic stimulus	76
GOTERM_MF_ALL	GO:0005385~zinc ion transmembrane transporter activity	4
GOTERM_BP_ALL	GO:0031347~regulation of defense response	7
GOTERM_BP_ALL	GO:0016101~diterpenoid metabolic process	4
GOTERM_BP_ALL	GO:0048731~system development	44
GOTERM_BP_ALL	GO:0022622~root system development	16
GOTERM_BP_ALL	GO:0048364~root development	16

GOTERM_BP_ALL	GO:0010152~pollen maturation	3
GOTERM_BP_ALL	GO:0010556~regulation of macromolecule biosynthetic p	126
GOTERM_BP_ALL	GO:0042545~cell wall modification	12
GOTERM_BP_ALL	GO:0021700~developmental maturation	6
GOTERM_MF_ALL	GO:0008324~cation transmembrane transporter activity	31
GOTERM_BP_ALL	GO:0042592~homeostatic process	21
GOTERM_BP_ALL	GO:0031326~regulation of cellular biosynthetic process	127
GOTERM_BP_ALL	GO:0009889~regulation of biosynthetic process	127
GOTERM_BP_ALL	GO:0010102~lateral root morphogenesis	4
GOTERM_BP_ALL	GO:0010101~post-embryonic root morphogenesis	4
GOTERM_BP_ALL	GO:0051761~sesquiterpene metabolic process	4
GOTERM_BP_ALL	GO:0006714~sesquiterpenoid metabolic process	4
GOTERM_BP_ALL	GO:0009100~glycoprotein metabolic process	7
GOTERM_BP_ALL	GO:0044264~cellular polysaccharide metabolic process	13
GOTERM_BP_ALL	GO:0044036~cell wall macromolecule metabolic process	6
GOTERM_BP_ALL	GO:0010468~regulation of gene expression	132
GOTERM_BP_ALL	GO:0046470~phosphatidylcholine metabolic process	3
GOTERM_BP_ALL	GO:0009639~response to red or far red light	14
GOTERM_BP_ALL	GO:0042214~terpene metabolic process	5
GOTERM_BP_ALL	GO:0048527~lateral root development	6
GOTERM_BP_ALL	GO:0034614~cellular response to reactive oxygen species	9
GOTERM_BP_ALL	GO:0048513~organ development	43
GOTERM_BP_ALL	GO:0046486~glycerolipid metabolic process	7
GOTERM_BP_ALL	GO:0006873~cellular ion homeostasis	8
GOTERM_BP_ALL	GO:0034599~cellular response to oxidative stress	9
GOTERM_MF_ALL	GO:0003785~actin monomer binding	2
GOTERM_MF_ALL	GO:0016726~oxidoreductase activity, acting on CH or CH	2
GOTERM_MF_ALL	GO:0033925~mannosyl-glycoprotein endo-beta-N-acetylg	2
GOTERM_BP_ALL	GO:0051457~maintenance of protein location in nucleus	2
GOTERM_CC_ALL	GO:0031012~extracellular matrix	4
GOTERM_BP_ALL	GO:0009861~jasmonic acid and ethylene-dependent syste	3
GOTERM_BP_ALL	GO:0009937~regulation of gibberellic acid mediated sign	3
GOTERM_BP_ALL	GO:0032507~maintenance of protein location in cell	3
GOTERM_BP_ALL	GO:0042439~ethanolamine and derivative metabolic proc	3
GOTERM_BP_ALL	GO:0048585~negative regulation of response to stimulus	7
GOTERM_BP_ALL	GO:0009850~auxin metabolic process	5
GOTERM_MF_ALL	GO:0050660~FAD binding	13
GOTERM_BP_ALL	GO:0006879~cellular iron ion homeostasis	3
GOTERM_BP_ALL	GO:0045185~maintenance of protein location	3
GOTERM_MF_ALL	GO:0005102~receptor binding	5
GOTERM_MF_ALL	GO:0015144~carbohydrate transmembrane transporter act	10
GOTERM_BP_ALL	GO:0048825~cotyledon development	4
GOTERM_BP_ALL	GO:0006814~sodium ion transport	4
GOTERM_BP_ALL	GO:0044092~negative regulation of molecular function	7
GOTERM_BP_ALL	GO:0080090~regulation of primary metabolic process	129
GOTERM_BP_ALL	GO:0048528~post-embryonic root development	6
GOTERM_MF_ALL	GO:0001883~purine nucleoside binding	146

GOTERM_MF_ALL	GO:0030554~adenyl nucleotide binding	146
GOTERM_BP_ALL	GO:0042538~hyperosmotic salinity response	5
GOTERM_BP_ALL	GO:0006022~aminoglycan metabolic process	4
GOTERM_BP_ALL	GO:0010029~regulation of seed germination	4
GOTERM_BP_ALL	GO:0006720~isoprenoid metabolic process	11
GOTERM_BP_ALL	GO:0031323~regulation of cellular metabolic process	129
GOTERM_CC_ALL	GO:0055044~symplast	3
GOTERM_CC_ALL	GO:0009506~plasmodesma	3
GOTERM_MF_ALL	GO:0070704~sterol desaturase activity	2
GOTERM_MF_ALL	GO:0010295~(+)-abscisic acid 8'-hydroxylase activity	2
GOTERM_BP_ALL	GO:0050789~regulation of biological process	214
GOTERM_BP_ALL	GO:0010015~root morphogenesis	8
GOTERM_MF_ALL	GO:0042802~identical protein binding	13
GOTERM_MF_ALL	GO:0001882~nucleoside binding	146
GOTERM_MF_ALL	GO:0016684~oxidoreductase activity, acting on peroxide	10
GOTERM_MF_ALL	GO:0004601~peroxidase activity	10
GOTERM_BP_ALL	GO:0009798~axis specification	4
GOTERM_MF_ALL	GO:0005244~voltage-gated ion channel activity	5
GOTERM_MF_ALL	GO:0022832~voltage-gated channel activity	5
GOTERM_MF_ALL	GO:0070011~peptidase activity, acting on L-amino acid p	35
GOTERM_CC_ALL	GO:0000145~exocyst	4
GOTERM_BP_ALL	GO:0080134~regulation of response to stress	7
GOTERM_MF_ALL	GO:0016757~transferase activity, transferring glycosyl gr	32
GOTERM_BP_ALL	GO:0042128~nitrate assimilation	3
GOTERM_BP_ALL	GO:0042126~nitrate metabolic process	3
GOTERM_BP_ALL	GO:0009612~response to mechanical stimulus	3
GOTERM_MF_ALL	GO:0015276~ligand-gated ion channel activity	4
GOTERM_MF_ALL	GO:0022834~ligand-gated channel activity	4
GOTERM_BP_ALL	GO:0006694~steroid biosynthetic process	5
GOTERM_BP_ALL	GO:0009741~response to brassinosteroid stimulus	5
GOTERM_BP_ALL	GO:0006644~phospholipid metabolic process	10
GOTERM_BP_ALL	GO:0016042~lipid catabolic process	15
GOTERM_MF_ALL	GO:0019104~DNA N-glycosylase activity	3
GOTERM_MF_ALL	GO:0016209~antioxidant activity	11
GOTERM_MF_ALL	GO:0008374~O-acyltransferase activity	5
GOTERM_BP_ALL	GO:0000302~response to reactive oxygen species	11
GOTERM_MF_ALL	GO:0016835~carbon-oxygen lyase activity	10
GOTERM_BP_ALL	GO:0055072~iron ion homeostasis	3
GOTERM_BP_ALL	GO:0060255~regulation of macromolecule metabolic pro	133
GOTERM_MF_ALL	GO:0004571~mannosyl-oligosaccharide 1,2-alpha-manno	2
GOTERM_MF_ALL	GO:0045544~gibberellin 20-oxidase activity	2
GOTERM_MF_ALL	GO:0046915~transition metal ion transmembrane transpo	5
GOTERM_BP_ALL	GO:0046247~terpene catabolic process	2
GOTERM_BP_ALL	GO:0009954~proximal/distal pattern formation	2
GOTERM_BP_ALL	GO:0006805~xenobiotic metabolic process	2
GOTERM_CC_ALL	GO:0009360~DNA polymerase III complex	2
GOTERM_BP_ALL	GO:0032502~developmental process	104

GOTERM_MF_ALL	GO:0015082~di-, tri-valent inorganic cation transmembra	6
GOTERM_BP_ALL	GO:0065009~regulation of molecular function	12
GOTERM_BP_ALL	GO:0016114~terpenoid biosynthetic process	7
GOTERM_MF_ALL	GO:0004089~carbonate dehydratase activity	3
GOTERM_MF_ALL	GO:0005548~phospholipid transporter activity	3
GOTERM_BP_ALL	GO:0016998~cell wall macromolecule catabolic process	4
GOTERM_BP_ALL	GO:0048519~negative regulation of biological process	24
GOTERM_BP_ALL	GO:0009617~response to bacterium	17
GOTERM_BP_ALL	GO:0005975~carbohydrate metabolic process	58
GOTERM_BP_ALL	GO:0006487~protein amino acid N-linked glycosylation	3
GOTERM_BP_ALL	GO:0019915~lipid storage	3
GOTERM_MF_ALL	GO:0043167~ion binding	201
GOTERM_CC_ALL	GO:0005911~cell-cell junction	3
GOTERM_MF_ALL	GO:0016857~racemase and epimerase activity, acting on	4
GOTERM_BP_ALL	GO:0009735~response to cytokinin stimulus	7
GOTERM_MF_ALL	GO:0005496~steroid binding	3
GOTERM_MF_ALL	GO:0043169~cation binding	200
GOTERM_BP_ALL	GO:0019222~regulation of metabolic process	137
GOTERM_MF_ALL	GO:0004190~aspartic-type endopeptidase activity	7
GOTERM_MF_ALL	GO:0070001~aspartic-type peptidase activity	7
GOTERM_BP_ALL	GO:0009736~cytokinin mediated signaling	5
GOTERM_BP_ALL	GO:0006972~hyperosmotic response	5
GOTERM_BP_ALL	GO:0009943~adaxial/abaxial axis specification	3
GOTERM_BP_ALL	GO:0019637~organophosphate metabolic process	10
GOTERM_MF_ALL	GO:0016758~transferase activity, transferring hexosyl gro	25
GOTERM_CC_ALL	GO:0016020~membrane	310
GOTERM_BP_ALL	GO:0042493~response to drug	7
GOTERM_CC_ALL	GO:0005811~lipid particle	3
GOTERM_CC_ALL	GO:0012511~monolayer-surrounded lipid storage body	3
GOTERM_MF_ALL	GO:0010294~abscisic acid glucosyltransferase activity	2
GOTERM_MF_ALL	GO:0050378~UDP-glucuronate 4-epimerase activity	2
GOTERM_MF_ALL	GO:0008526~phosphatidylinositol transporter activity	2
GOTERM_MF_ALL	GO:0015924~mannosyl-oligosaccharide mannosidase acti	2
GOTERM_MF_ALL	GO:0000822~inositol hexakisphosphate binding	2
GOTERM_MF_ALL	GO:0004607~phosphatidylcholine-sterol O-acyltransferas	2
GOTERM_CC_ALL	GO:0008287~protein serine/threonine phosphatase compl	6
GOTERM_BP_ALL	GO:0030198~extracellular matrix organization	2
GOTERM_BP_ALL	GO:0007030~Golgi organization	2
GOTERM_BP_ALL	GO:0043062~extracellular structure organization	2
GOTERM_MF_ALL	GO:0015238~drug transporter activity	7
GOTERM_MF_ALL	GO:0043492~ATPase activity, coupled to movement of s	13
GOTERM_MF_ALL	GO:0042626~ATPase activity, coupled to transmembrane	13
GOTERM_BP_ALL	GO:0043086~negative regulation of catalytic activity	6
GOTERM_MF_ALL	GO:0005230~extracellular ligand-gated ion channel activi	3
GOTERM_MF_ALL	GO:0004970~ionotropic glutamate receptor activity	3
GOTERM_MF_ALL	GO:0005234~extracellular-glutamate-gated ion channel a	3
GOTERM_MF_ALL	GO:0008066~glutamate receptor activity	3

GOTERM_BP_ALL	GO:0006904~vesicle docking during exocytosis	4
GOTERM_BP_ALL	GO:0010016~shoot morphogenesis	11
GOTERM_BP_ALL	GO:0046903~secretion	5
GOTERM_BP_ALL	GO:0032940~secretion by cell	5
GOTERM_BP_ALL	GO:0042742~defense response to bacterium	13
GOTERM_MF_ALL	GO:0016641~oxidoreductase activity, acting on the CH-N	4
GOTERM_MF_ALL	GO:0015197~peptide transporter activity	3
GOTERM_MF_ALL	GO:0015198~oligopeptide transporter activity	3
GOTERM_MF_ALL	GO:0016799~hydrolase activity, hydrolyzing N-glycosyl	3
GOTERM_BP_ALL	GO:0048278~vesicle docking	4
GOTERM_BP_ALL	GO:0022406~membrane docking	4
GOTERM_MF_ALL	GO:0032559~adenyl ribonucleotide binding	133
GOTERM_BP_ALL	GO:0009851~auxin biosynthetic process	3
GOTERM_BP_ALL	GO:0006829~zinc ion transport	3
GOTERM_BP_ALL	GO:0009636~response to toxin	3
GOTERM_MF_ALL	GO:0043178~alcohol binding	2
GOTERM_CC_ALL	GO:0005783~endoplasmic reticulum	28
GOTERM_MF_ALL	GO:0046872~metal ion binding	189
GOTERM_BP_ALL	GO:0007584~response to nutrient	2
GOTERM_BP_ALL	GO:0007610~behavior	2
GOTERM_BP_ALL	GO:0050793~regulation of developmental process	17
GOTERM_BP_ALL	GO:0009832~plant-type cell wall biogenesis	5
GOTERM_MF_ALL	GO:0016820~hydrolase activity, acting on acid anhydride	13
GOTERM_MF_ALL	GO:0016787~hydrolase activity	174
GOTERM_MF_ALL	GO:0005217~intracellular ligand-gated ion channel activi	3
GOTERM_MF_ALL	GO:0016854~racemase and epimerase activity	4
GOTERM_BP_ALL	GO:0007154~cell communication	12
GOTERM_BP_ALL	GO:0010073~meristem maintenance	5
GOTERM_MF_ALL	GO:0004650~polygalacturonase activity	6
GOTERM_BP_ALL	GO:0006470~protein amino acid dephosphorylation	6
GOTERM_MF_ALL	GO:0005529~sugar binding	9
GOTERM_MF_ALL	GO:0005199~structural constituent of cell wall	4
GOTERM_MF_ALL	GO:0005261~cation channel activity	4
GOTERM_MF_ALL	GO:0005097~Rab GTPase activator activity	3
GOTERM_BP_ALL	GO:0048584~positive regulation of response to stimulus	5
GOTERM_BP_ALL	GO:0048438~floral whorl development	8
GOTERM_MF_ALL	GO:0005381~iron ion transmembrane transporter activity	2
GOTERM_MF_ALL	GO:0010011~auxin binding	2
GOTERM_MF_ALL	GO:0008725~DNA-3-methyladenine glycosylase I activit	2
GOTERM_MF_ALL	GO:0015081~sodium ion transmembrane transporter activ	2
GOTERM_MF_ALL	GO:0004714~transmembrane receptor protein tyrosine ki	2
GOTERM_MF_ALL	GO:0016725~oxidoreductase activity, acting on CH or CH	2
GOTERM_MF_ALL	GO:0015370~solute:sodium symporter activity	2
GOTERM_MF_ALL	GO:0004556~alpha-amylase activity	2
GOTERM_MF_ALL	GO:0016566~specific transcriptional repressor activity	2
GOTERM_BP_ALL	GO:0006887~exocytosis	4
GOTERM_BP_ALL	GO:0032483~regulation of Rab protein signal transductio	3

GOTERM_BP_ALL	GO:0045892~negative regulation of transcription, DNA-d	3
GOTERM_BP_ALL	GO:0051253~negative regulation of RNA metabolic proc	3
GOTERM_BP_ALL	GO:0032313~regulation of Rab GTPase activity	3
GOTERM_BP_ALL	GO:0052317~camalexin metabolic process	2
GOTERM_BP_ALL	GO:0009956~radial pattern formation	2
GOTERM_BP_ALL	GO:0009682~induced systemic resistance	2
GOTERM_BP_ALL	GO:0010120~camalexin biosynthetic process	2
GOTERM_CC_ALL	GO:0030054~cell junction	3
GOTERM_CC_ALL	GO:0005788~endoplasmic reticulum lumen	3
GOTERM_MF_ALL	GO:0008233~peptidase activity	35
GOTERM_BP_ALL	GO:0006073~cellular glucan metabolic process	9
GOTERM_BP_ALL	GO:0009414~response to water deprivation	12
GOTERM_BP_ALL	GO:0015674~di-, tri-valent inorganic cation transport	5
GOTERM_BP_ALL	GO:0009791~post-embryonic development	50
GOTERM_BP_ALL	GO:0000271~polysaccharide biosynthetic process	8
GOTERM_BP_ALL	GO:0042546~cell wall biogenesis	6
GOTERM_MF_ALL	GO:0016563~transcription activator activity	10
GOTERM_MF_ALL	GO:0051119~sugar transmembrane transporter activity	8
GOTERM_BP_ALL	GO:0048856~anatomical structure development	76
GOTERM_MF_ALL	GO:0004499~flavin-containing monooxygenase activity	3
GOTERM_BP_ALL	GO:0003002~regionalization	7
GOTERM_BP_ALL	GO:0044255~cellular lipid metabolic process	29
GOTERM_CC_ALL	GO:0019898~extrinsic to membrane	15
GOTERM_BP_ALL	GO:0048588~developmental cell growth	6
GOTERM_BP_ALL	GO:0009886~post-embryonic morphogenesis	6
GOTERM_MF_ALL	GO:0005524~ATP binding	130
GOTERM_BP_ALL	GO:0009955~adaxial/abaxial pattern formation	3
GOTERM_BP_ALL	GO:0009737~response to abscisic acid stimulus	18
GOTERM_CC_ALL	GO:0005795~Golgi stack	3
GOTERM_BP_ALL	GO:0055085~transmembrane transport	14
GOTERM_BP_ALL	GO:0009875~pollen-pistil interaction	4
GOTERM_BP_ALL	GO:0006855~multidrug transport	6
GOTERM_MF_ALL	GO:0005345~purine transmembrane transporter activity	3
GOTERM_MF_ALL	GO:0009927~histidine phosphotransfer kinase activity	2
GOTERM_MF_ALL	GO:0003905~alkylbase DNA N-glycosylase activity	2
GOTERM_CC_ALL	GO:0044432~endoplasmic reticulum part	7
GOTERM_MF_ALL	GO:0000156~two-component response regulator activity	4
GOTERM_MF_ALL	GO:0015405~P-P-bond-hydrolysis-driven transmembrane	15
GOTERM_MF_ALL	GO:0017076~purine nucleotide binding	154
GOTERM_BP_ALL	GO:0009991~response to extracellular stimulus	7
GOTERM_BP_ALL	GO:0042431~indole metabolic process	2
GOTERM_BP_ALL	GO:0052314~phytoalexin metabolic process	2
GOTERM_BP_ALL	GO:0052315~phytoalexin biosynthetic process	2
GOTERM_BP_ALL	GO:0046217~indole phytoalexin metabolic process	2
GOTERM_BP_ALL	GO:0010229~inflorescence development	2
GOTERM_BP_ALL	GO:0009700~indole phytoalexin biosynthetic process	2
GOTERM_CC_ALL	GO:0042575~DNA polymerase complex	2

GOTERM_BP_ALL	GO:0006032~chitin catabolic process	3
GOTERM_BP_ALL	GO:0006030~chitin metabolic process	3
GOTERM_BP_ALL	GO:0006026~aminoglycan catabolic process	3
GOTERM_BP_ALL	GO:0006816~calcium ion transport	3
GOTERM_MF_ALL	GO:0015399~primary active transmembrane transporter a	15
GOTERM_BP_ALL	GO:0006576~biogenic amine metabolic process	5
GOTERM_MF_ALL	GO:0042803~protein homodimerization activity	5
GOTERM_BP_ALL	GO:0048646~anatomical structure formation involved in	7
GOTERM_MF_ALL	GO:0050661~NADP or NADPH binding	4
GOTERM_MF_ALL	GO:0004806~triacylglycerol lipase activity	4
GOTERM_MF_ALL	GO:0004568~chitinase activity	3
GOTERM_MF_ALL	GO:0015297~antiporter activity	10
GOTERM_BP_ALL	GO:0048466~androecium development	4
GOTERM_BP_ALL	GO:0030384~phosphoinositide metabolic process	4
GOTERM_BP_ALL	GO:0048443~stamen development	4
GOTERM_BP_ALL	GO:0006874~cellular calcium ion homeostasis	3
GOTERM_BP_ALL	GO:0009742~brassinosteroid mediated signaling	3
GOTERM_BP_ALL	GO:0048767~root hair elongation	3
GOTERM_BP_ALL	GO:0048545~response to steroid hormone stimulus	3
GOTERM_BP_ALL	GO:0006284~base-excision repair	3
GOTERM_BP_ALL	GO:0055074~calcium ion homeostasis	3
GOTERM_BP_ALL	GO:0043401~steroid hormone mediated signaling	3
GOTERM_BP_ALL	GO:0042127~regulation of cell proliferation	3
GOTERM_MF_ALL	GO:0004091~carboxylesterase activity	21
GOTERM_CC_ALL	GO:0000139~Golgi membrane	4
GOTERM_CC_ALL	GO:0031300~intrinsic to organelle membrane	4
GOTERM_MF_ALL	GO:0008061~chitin binding	2
GOTERM_MF_ALL	GO:0015368~calcium:cation antiporter activity	2
GOTERM_BP_ALL	GO:0050790~regulation of catalytic activity	10
GOTERM_BP_ALL	GO:0009415~response to water	12
GOTERM_BP_ALL	GO:0045087~innate immune response	17
GOTERM_BP_ALL	GO:0006355~regulation of transcription, DNA-dependent	62
GOTERM_BP_ALL	GO:0051241~negative regulation of multicellular organis	2
GOTERM_BP_ALL	GO:0008300~isoprenoid catabolic process	2
GOTERM_BP_ALL	GO:0010187~negative regulation of seed germination	2
GOTERM_BP_ALL	GO:0016115~terpenoid catabolic process	2
GOTERM_BP_ALL	GO:0045489~pectin biosynthetic process	2
GOTERM_BP_ALL	GO:0015706~nitrate transport	2
GOTERM_CC_ALL	GO:0000152~nuclear ubiquitin ligase complex	2
GOTERM_BP_ALL	GO:0032501~multicellular organismal process	96
GOTERM_BP_ALL	GO:0008654~phospholipid biosynthetic process	6
GOTERM_BP_ALL	GO:0006820~anion transport	6
GOTERM_BP_ALL	GO:0000003~reproduction	52
GOTERM_MF_ALL	GO:0003993~acid phosphatase activity	4
GOTERM_BP_ALL	GO:0016246~RNA interference	3
GOTERM_MF_ALL	GO:0016702~oxidoreductase activity, acting on single do	5
GOTERM_MF_ALL	GO:0005402~cation:sugar symporter activity	7

GOTERM_MF_ALL	GO:0005351~sugar:hydrogen symporter activity	7
GOTERM_MF_ALL	GO:0015295~solute:hydrogen symporter activity	7
GOTERM_BP_ALL	GO:0051252~regulation of RNA metabolic process	62
GOTERM_BP_ALL	GO:0044042~glucan metabolic process	10
GOTERM_MF_ALL	GO:0016706~oxidoreductase activity, acting on paired do	6
GOTERM_BP_ALL	GO:0015893~drug transport	6
GOTERM_MF_ALL	GO:0016298~lipase activity	9
GOTERM_BP_ALL	GO:0048608~reproductive structure development	42
GOTERM_BP_ALL	GO:0009626~plant-type hypersensitive response	4
GOTERM_MF_ALL	GO:0016208~AMP binding	3
GOTERM_MF_ALL	GO:0005249~voltage-gated potassium channel activity	3
GOTERM_CC_ALL	GO:0044448~cell cortex part	4
GOTERM_BP_ALL	GO:0031668~cellular response to extracellular stimulus	6
GOTERM_BP_ALL	GO:0031667~response to nutrient levels	6
GOTERM_MF_ALL	GO:0046527~glucosyltransferase activity	9
GOTERM_MF_ALL	GO:0004034~aldose 1-epimerase activity	2
GOTERM_BP_ALL	GO:0046474~glycerophospholipid biosynthetic process	3
GOTERM_BP_ALL	GO:0033554~cellular response to stress	25
GOTERM_BP_ALL	GO:0010267~RNA interference, production of ta-siRNAs	2
GOTERM_BP_ALL	GO:0010204~defense response signaling pathway, resista	2
GOTERM_BP_ALL	GO:0034050~host programmed cell death induced by sym	4
GOTERM_MF_ALL	GO:0051213~dioxygenase activity	5
GOTERM_MF_ALL	GO:0016638~oxidoreductase activity, acting on the CH-N	4
GOTERM_MF_ALL	GO:0015205~nucleobase transmembrane transporter activ	3
GOTERM_MF_ALL	GO:0022843~voltage-gated cation channel activity	3
GOTERM_BP_ALL	GO:0043455~regulation of secondary metabolic process	3
GOTERM_MF_ALL	GO:0000166~nucleotide binding	181
GOTERM_BP_ALL	GO:0008643~carbohydrate transport	6
GOTERM_MF_ALL	GO:0008194~UDP-glycosyltransferase activity	14
GOTERM_MF_ALL	GO:0046983~protein dimerization activity	15
GOTERM_BP_ALL	GO:0009753~response to jasmonic acid stimulus	10
GOTERM_BP_ALL	GO:0019725~cellular homeostasis	14
GOTERM_CC_ALL	GO:0005938~cell cortex	4
GOTERM_BP_ALL	GO:0048518~positive regulation of biological process	13
GOTERM_MF_ALL	GO:0004175~endopeptidase activity	18
GOTERM_BP_ALL	GO:0022621~shoot system development	17
GOTERM_MF_ALL	GO:0004630~phospholipase D activity	2
GOTERM_MF_ALL	GO:0031402~sodium ion binding	2
GOTERM_MF_ALL	GO:0070290~NAPE-specific phospholipase D activity	2
GOTERM_BP_ALL	GO:0006979~response to oxidative stress	17
GOTERM_BP_ALL	GO:0016311~dephosphorylation	6
GOTERM_MF_ALL	GO:0045735~nutrient reservoir activity	5
GOTERM_BP_ALL	GO:0006508~proteolysis	58
GOTERM_BP_ALL	GO:0009862~systemic acquired resistance, salicylic acid	2
GOTERM_BP_ALL	GO:0006144~purine base metabolic process	2
GOTERM_BP_ALL	GO:0051056~regulation of small GTPase mediated signal	4
GOTERM_BP_ALL	GO:0046578~regulation of Ras protein signal transductio	4

GOTERM_BP_ALL	GO:0040008~regulation of growth	4
GOTERM_CC_ALL	GO:0044431~Golgi apparatus part	7
GOTERM_BP_ALL	GO:0051258~protein polymerization	3
GOTERM_BP_ALL	GO:0009911~positive regulation of flower development	3
GOTERM_BP_ALL	GO:0048638~regulation of developmental growth	3
GOTERM_CC_ALL	GO:0034702~ion channel complex	2
GOTERM_BP_ALL	GO:0033692~cellular polysaccharide biosynthetic process	7
GOTERM_BP_ALL	GO:0008610~lipid biosynthetic process	24
GOTERM_BP_ALL	GO:0002376~immune system process	18
GOTERM_MF_ALL	GO:0008509~anion transmembrane transporter activity	7
GOTERM_CC_ALL	GO:0031226~intrinsic to plasma membrane	7
GOTERM_MF_ALL	GO:0030551~cyclic nucleotide binding	3
GOTERM_MF_ALL	GO:0005319~lipid transporter activity	3
GOTERM_BP_ALL	GO:0015672~monovalent inorganic cation transport	10
GOTERM_BP_ALL	GO:0048511~rhythmic process	4
GOTERM_MF_ALL	GO:0016769~transferase activity, transferring nitrogenou	5
GOTERM_MF_ALL	GO:0043565~sequence-specific DNA binding	27
GOTERM_BP_ALL	GO:0010382~cellular cell wall macromolecule metabolic	2
GOTERM_MF_ALL	GO:0005267~potassium channel activity	3
GOTERM_MF_ALL	GO:0010181~FMN binding	3
GOTERM_CC_ALL	GO:0016602~CCAAT-binding factor complex	2
GOTERM_BP_ALL	GO:0031669~cellular response to nutrient levels	5
GOTERM_BP_ALL	GO:0045017~glycerolipid biosynthetic process	3
GOTERM_CC_ALL	GO:0005789~endoplasmic reticulum membrane	5
GOTERM_MF_ALL	GO:0050662~coenzyme binding	21
GOTERM_BP_ALL	GO:0006955~immune response	17
GOTERM_MF_ALL	GO:0032555~purine ribonucleotide binding	141
GOTERM_MF_ALL	GO:0032553~ribonucleotide binding	141
GOTERM_MF_ALL	GO:0015112~nitrate transmembrane transporter activity	2
GOTERM_MF_ALL	GO:0008113~peptide-methionine-(S)-S-oxide reductase a	2
GOTERM_MF_ALL	GO:0003677~DNA binding	131
GOTERM_MF_ALL	GO:0048037~cofactor binding	29
GOTERM_MF_ALL	GO:0016746~transferase activity, transferring acyl groups	17
GOTERM_BP_ALL	GO:0010200~response to chitin	8
GOTERM_MF_ALL	GO:0046914~transition metal ion binding	148
GOTERM_BP_ALL	GO:0006801~superoxide metabolic process	2
GOTERM_BP_ALL	GO:0080010~regulation of oxygen and reactive oxygen sp	2
GOTERM_BP_ALL	GO:0009638~phototropism	2
GOTERM_BP_ALL	GO:0009870~defense response signaling pathway, resista	2
GOTERM_BP_ALL	GO:0009625~response to insect	2
GOTERM_BP_ALL	GO:0018106~peptidyl-histidine phosphorylation	2
GOTERM_BP_ALL	GO:0045488~pectin metabolic process	2
GOTERM_BP_ALL	GO:0032506~cytokinetic process	2
GOTERM_BP_ALL	GO:0009900~dehiscence	2
GOTERM_MF_ALL	GO:0003779~actin binding	5
GOTERM_MF_ALL	GO:0016701~oxidoreductase activity, acting on single do	5
GOTERM_BP_ALL	GO:0015698~inorganic anion transport	4

GOTERM_BP_ALL	GO:0051239~regulation of multicellular organismal proce	12
GOTERM_BP_ALL	GO:0022414~reproductive process	49
GOTERM_BP_ALL	GO:0009965~leaf morphogenesis	7
GOTERM_MF_ALL	GO:0004721~phosphoprotein phosphatase activity	12
GOTERM_MF_ALL	GO:0005099~Ras GTPase activator activity	3
GOTERM_MF_ALL	GO:0005484~SNAP receptor activity	3
GOTERM_BP_ALL	GO:0048827~phyllome development	12
GOTERM_BP_ALL	GO:0005982~starch metabolic process	3
GOTERM_CC_ALL	GO:0042175~nuclear envelope-endoplasmic reticulum ne	5
GOTERM_MF_ALL	GO:0015562~efflux transmembrane transporter activity	2
GOTERM_MF_ALL	GO:0008308~voltage-gated anion channel activity	2
GOTERM_MF_ALL	GO:0031420~alkali metal ion binding	5
GOTERM_BP_ALL	GO:0048507~meristem development	6
GOTERM_BP_ALL	GO:0000041~transition metal ion transport	5
GOTERM_BP_ALL	GO:0018202~peptidyl-histidine modification	2
GOTERM_BP_ALL	GO:0010167~response to nitrate	2
GOTERM_BP_ALL	GO:0009593~detection of chemical stimulus	2
GOTERM_BP_ALL	GO:0006813~potassium ion transport	4
GOTERM_MF_ALL	GO:0015662~ATPase activity, coupled to transmembrane	4
GOTERM_BP_ALL	GO:0009867~jasmonic acid mediated signaling pathway	3
GOTERM_BP_ALL	GO:0048544~recognition of pollen	3
GOTERM_BP_ALL	GO:0008037~cell recognition	3
GOTERM_BP_ALL	GO:0048367~shoot development	16
GOTERM_MF_ALL	GO:0005507~copper ion binding	10
GOTERM_BP_ALL	GO:0000272~polysaccharide catabolic process	5
GOTERM_MF_ALL	GO:0004722~protein serine/threonine phosphatase activit	9
GOTERM_BP_ALL	GO:0007275~multicellular organismal development	88
GOTERM_BP_ALL	GO:0048509~regulation of meristem development	4
GOTERM_BP_ALL	GO:0003006~reproductive developmental process	44
GOTERM_BP_ALL	GO:0008299~isoprenoid biosynthetic process	7
GOTERM_MF_ALL	GO:0042910~xenobiotic transporter activity	2
GOTERM_MF_ALL	GO:0008559~xenobiotic-transporting ATPase activity	2
GOTERM_MF_ALL	GO:0042973~glucan endo-1,3-beta-D-glucosidase activity	2
GOTERM_MF_ALL	GO:0005253~anion channel activity	2
GOTERM_MF_ALL	GO:0008601~protein phosphatase type 2A regulator activ	2
GOTERM_MF_ALL	GO:0019239~deaminase activity	2
GOTERM_BP_ALL	GO:0009894~regulation of catabolic process	2
GOTERM_BP_ALL	GO:0006506~GPI anchor biosynthetic process	2
GOTERM_BP_ALL	GO:0010252~auxin homeostasis	2
GOTERM_BP_ALL	GO:0009944~polarity specification of adaxial/abaxial axi	2
GOTERM_BP_ALL	GO:0048465~corolla development	2
GOTERM_BP_ALL	GO:0019216~regulation of lipid metabolic process	2
GOTERM_BP_ALL	GO:0048441~petal development	2
GOTERM_CC_ALL	GO:0000159~protein phosphatase type 2A complex	2
GOTERM_CC_ALL	GO:0005615~extracellular space	2
GOTERM_CC_ALL	GO:0005887~integral to plasma membrane	3
GOTERM_BP_ALL	GO:0010114~response to red light	4

GOTERM_BP_ALL	GO:0033365~protein localization in organelle	5
GOTERM_BP_ALL	GO:0048582~positive regulation of post-embryonic devel	3
GOTERM_BP_ALL	GO:0048469~cell maturation	3
GOTERM_BP_ALL	GO:0048765~root hair cell differentiation	3
GOTERM_BP_ALL	GO:0048764~trichoblast maturation	3
GOTERM_MF_ALL	GO:0004712~protein serine/threonine/tyrosine kinase acti	4
GOTERM_MF_ALL	GO:0015923~mannosidase activity	2
GOTERM_MF_ALL	GO:0016160~amylase activity	2
GOTERM_MF_ALL	GO:0004551~nucleotide diphosphatase activity	2
GOTERM_MF_ALL	GO:0016759~cellulose synthase activity	3
GOTERM_MF_ALL	GO:0008378~galactosyltransferase activity	3
GOTERM_BP_ALL	GO:0019252~starch biosynthetic process	2
GOTERM_BP_ALL	GO:0006536~glutamate metabolic process	2
GOTERM_BP_ALL	GO:0065001~specification of axis polarity	2
GOTERM_BP_ALL	GO:0007155~cell adhesion	2
GOTERM_BP_ALL	GO:0022610~biological adhesion	2
GOTERM_CC_ALL	GO:0048046~apoplast	21
GOTERM_MF_ALL	GO:0016747~transferase activity, transferring acyl groups	15
GOTERM_BP_ALL	GO:0016481~negative regulation of transcription	4
GOTERM_MF_ALL	GO:0015491~cation:cation antiporter activity	4
GOTERM_MF_ALL	GO:0035251~UDP-glucosyltransferase activity	7
GOTERM_BP_ALL	GO:0019748~secondary metabolic process	23
GOTERM_CC_ALL	GO:0005794~Golgi apparatus	20
GOTERM_MF_ALL	GO:0004252~serine-type endopeptidase activity	6
GOTERM_MF_ALL	GO:0016790~thiolester hydrolase activity	6
GOTERM_MF_ALL	GO:0019888~protein phosphatase regulator activity	2
GOTERM_MF_ALL	GO:0019208~phosphatase regulator activity	2
GOTERM_MF_ALL	GO:0015239~multidrug transporter activity	2
GOTERM_BP_ALL	GO:0009651~response to salt stress	20
GOTERM_BP_ALL	GO:0048532~anatomical structure arrangement	3
GOTERM_BP_ALL	GO:0007623~circadian rhythm	3
GOTERM_BP_ALL	GO:0000911~cytokinesis by cell plate formation	2
GOTERM_BP_ALL	GO:0006505~GPI anchor metabolic process	2
GOTERM_BP_ALL	GO:0030422~RNA interference, production of siRNA	2
GOTERM_BP_ALL	GO:0009834~secondary cell wall biogenesis	2
GOTERM_BP_ALL	GO:0009934~regulation of meristem structural organizati	2
GOTERM_MF_ALL	GO:0042625~ATPase activity, coupled to transmembrane	5
GOTERM_CC_ALL	GO:0031227~intrinsic to endoplasmic reticulum membran	2
GOTERM_CC_ALL	GO:0005774~vacuolar membrane	5
GOTERM_MF_ALL	GO:0015300~solute:solute antiporter activity	5
GOTERM_BP_ALL	GO:0010051~xylem and phloem pattern formation	3
GOTERM_BP_ALL	GO:0010054~trichoblast differentiation	3
GOTERM_BP_ALL	GO:0048468~cell development	9
GOTERM_BP_ALL	GO:0051093~negative regulation of developmental proce	5
GOTERM_BP_ALL	GO:0009267~cellular response to starvation	4
GOTERM_BP_ALL	GO:0010074~maintenance of meristem identity	2
GOTERM_BP_ALL	GO:0006808~regulation of nitrogen utilization	2

GOTERM_BP_ALL	GO:0009112~nucleobase metabolic process	2
GOTERM_MF_ALL	GO:0046943~carboxylic acid transmembrane transporter	5
GOTERM_MF_ALL	GO:0005342~organic acid transmembrane transporter acti	5
GOTERM_BP_ALL	GO:0032318~regulation of Ras GTPase activity	3
GOTERM_MF_ALL	GO:0003725~double-stranded RNA binding	2
GOTERM_MF_ALL	GO:0008146~sulfotransferase activity	2
GOTERM_MF_ALL	GO:0030553~cGMP binding	2
GOTERM_MF_ALL	GO:0019002~GMP binding	2
GOTERM_BP_ALL	GO:0009690~cytokinin metabolic process	2
GOTERM_BP_ALL	GO:0009631~cold acclimation	2
GOTERM_BP_ALL	GO:0010118~stomatal movement	2
GOTERM_CC_ALL	GO:0044437~vacuolar part	5
GOTERM_MF_ALL	GO:0015298~solute:cation antiporter activity	4
GOTERM_BP_ALL	GO:0048580~regulation of post-embryonic development	8
GOTERM_BP_ALL	GO:0010629~negative regulation of gene expression	9
GOTERM_BP_ALL	GO:0044262~cellular carbohydrate metabolic process	27
GOTERM_MF_ALL	GO:0008514~organic anion transmembrane transporter ac	2
GOTERM_MF_ALL	GO:0030552~cAMP binding	2
GOTERM_BP_ALL	GO:0051094~positive regulation of developmental proces	3
GOTERM_BP_ALL	GO:0006875~cellular metal ion homeostasis	3
GOTERM_BP_ALL	GO:0055065~metal ion homeostasis	3
GOTERM_CC_ALL	GO:0009898~internal side of plasma membrane	3
GOTERM_MF_ALL	GO:0016846~carbon-sulfur lyase activity	3
GOTERM_BP_ALL	GO:0010075~regulation of meristem growth	2
GOTERM_BP_ALL	GO:0033205~cytokinesis during cell cycle	2
GOTERM_BP_ALL	GO:0043331~response to dsRNA	2
GOTERM_BP_ALL	GO:0031050~dsRNA fragmentation	2
GOTERM_MF_ALL	GO:0045330~aspartyl esterase activity	4
GOTERM_BP_ALL	GO:0019953~sexual reproduction	4
GOTERM_BP_ALL	GO:0009611~response to wounding	8
GOTERM_BP_ALL	GO:0010218~response to far red light	3
GOTERM_MF_ALL	GO:0022890~inorganic cation transmembrane transporter	12
GOTERM_BP_ALL	GO:0009887~organ morphogenesis	9
GOTERM_MF_ALL	GO:0016717~oxidoreductase activity, acting on paired do	2
GOTERM_MF_ALL	GO:0001727~lipid kinase activity	2
GOTERM_MF_ALL	GO:0008134~transcription factor binding	3
GOTERM_MF_ALL	GO:0004221~ubiquitin thiolesterase activity	4
GOTERM_BP_ALL	GO:0019932~second-messenger-mediated signaling	2
GOTERM_BP_ALL	GO:0046489~phosphoinositide biosynthetic process	2
GOTERM_BP_ALL	GO:0046777~protein amino acid autophosphorylation	2
GOTERM_CC_ALL	GO:0046658~anchored to plasma membrane	4
GOTERM_BP_ALL	GO:0043087~regulation of GTPase activity	3
GOTERM_MF_ALL	GO:0016881~acid-amino acid ligase activity	15
GOTERM_BP_ALL	GO:0051172~negative regulation of nitrogen compound m	4
GOTERM_BP_ALL	GO:0045934~negative regulation of nucleobase, nucleosi	4
GOTERM_BP_ALL	GO:0042594~response to starvation	4
GOTERM_MF_ALL	GO:0015932~nucleobase, nucleoside, nucleotide and nucl	3

GOTERM_MF_ALL	GO:0016763~transferase activity, transferring pentosyl gr	3
GOTERM_MF_ALL	GO:0019213~deacetylase activity	2
GOTERM_BP_ALL	GO:0009407~toxin catabolic process	3
GOTERM_BP_ALL	GO:0009640~photomorphogenesis	3
GOTERM_BP_ALL	GO:0009404~toxin metabolic process	3
GOTERM_BP_ALL	GO:0010053~root epidermal cell differentiation	3
GOTERM_BP_ALL	GO:0009932~cell tip growth	4
GOTERM_BP_ALL	GO:0051606~detection of stimulus	4
GOTERM_BP_ALL	GO:0006863~purine transport	2
GOTERM_CC_ALL	GO:0005744~mitochondrial inner membrane presequence	2
GOTERM_BP_ALL	GO:0030154~cell differentiation	16
GOTERM_BP_ALL	GO:0010558~negative regulation of macromolecule biosy	4
GOTERM_BP_ALL	GO:0033036~macromolecule localization	36
GOTERM_MF_ALL	GO:0048038~quinone binding	2
GOTERM_BP_ALL	GO:0051336~regulation of hydrolase activity	3
GOTERM_BP_ALL	GO:0006012~galactose metabolic process	2
GOTERM_BP_ALL	GO:0015851~nucleobase transport	2
GOTERM_BP_ALL	GO:0019827~stem cell maintenance	2
GOTERM_BP_ALL	GO:0035194~posttranscriptional gene silencing by RNA	5
GOTERM_MF_ALL	GO:0004364~glutathione transferase activity	3
GOTERM_MF_ALL	GO:0016847~1-aminocyclopropane-1-carboxylate syntha	2
GOTERM_MF_ALL	GO:0016645~oxidoreductase activity, acting on the CH-N	2
GOTERM_MF_ALL	GO:0005275~amine transmembrane transporter activity	4
GOTERM_BP_ALL	GO:0048864~stem cell development	2
GOTERM_BP_ALL	GO:0042558~pteridine and derivative metabolic process	2
GOTERM_BP_ALL	GO:0010193~response to ozone	2
GOTERM_BP_ALL	GO:0006826~iron ion transport	2
GOTERM_CC_ALL	GO:0000325~plant-type vacuole	4
GOTERM_BP_ALL	GO:0048440~carpel development	3
GOTERM_BP_ALL	GO:0048609~reproductive process in a multicellular orga	3
GOTERM_MF_ALL	GO:0016836~hydro-lyase activity	4
GOTERM_BP_ALL	GO:0032787~monocarboxylic acid metabolic process	18
GOTERM_BP_ALL	GO:0006970~response to osmotic stress	20
GOTERM_MF_ALL	GO:0003887~DNA-directed DNA polymerase activity	2
GOTERM_BP_ALL	GO:0009743~response to carbohydrate stimulus	10
GOTERM_BP_ALL	GO:0015931~nucleobase, nucleoside, nucleotide and nucl	3
GOTERM_MF_ALL	GO:0004842~ubiquitin-protein ligase activity	13
GOTERM_BP_ALL	GO:0009816~defense response to bacterium, incompatibl	2
GOTERM_BP_ALL	GO:0042157~lipoprotein metabolic process	2
GOTERM_BP_ALL	GO:0009225~nucleotide-sugar metabolic process	2
GOTERM_BP_ALL	GO:0042158~lipoprotein biosynthetic process	2
GOTERM_BP_ALL	GO:0048863~stem cell differentiation	2
GOTERM_BP_ALL	GO:0006497~protein amino acid lipidation	2
GOTERM_BP_ALL	GO:0009890~negative regulation of biosynthetic process	4
GOTERM_BP_ALL	GO:0010228~vegetative to reproductive phase transition	4
GOTERM_BP_ALL	GO:0031327~negative regulation of cellular biosynthetic	4
GOTERM_BP_ALL	GO:0007568~aging	4

GOTERM_BP_ALL	GO:0048610~reproductive cellular process	6
GOTERM_MF_ALL	GO:0003924~GTPase activity	5
GOTERM_MF_ALL	GO:0070279~vitamin B6 binding	6
GOTERM_MF_ALL	GO:0030170~pyridoxal phosphate binding	6
GOTERM_BP_ALL	GO:0009860~pollen tube growth	3
GOTERM_BP_ALL	GO:0009311~oligosaccharide metabolic process	3
GOTERM_MF_ALL	GO:0008810~cellulase activity	2
GOTERM_MF_ALL	GO:0003713~transcription coactivator activity	2
GOTERM_MF_ALL	GO:0016791~phosphatase activity	15
GOTERM_MF_ALL	GO:0042578~phosphoric ester hydrolase activity	18
GOTERM_BP_ALL	GO:0040034~regulation of development, heterochronic	2
GOTERM_BP_ALL	GO:0045596~negative regulation of cell differentiation	2
GOTERM_BP_ALL	GO:0010605~negative regulation of macromolecule meta	9
GOTERM_MF_ALL	GO:0016829~lyase activity	18
GOTERM_MF_ALL	GO:0005488~binding	646
GOTERM_BP_ALL	GO:0032504~multicellular organism reproduction	3
GOTERM_MF_ALL	GO:0004197~cysteine-type endopeptidase activity	3
GOTERM_MF_ALL	GO:0005096~GTPase activator activity	3
GOTERM_BP_ALL	GO:0048366~leaf development	9
GOTERM_BP_ALL	GO:0045859~regulation of protein kinase activity	2
GOTERM_BP_ALL	GO:0043549~regulation of kinase activity	2
GOTERM_MF_ALL	GO:0008483~transaminase activity	3
GOTERM_CC_ALL	GO:0005741~mitochondrial outer membrane	2
GOTERM_MF_ALL	GO:0016853~isomerase activity	11
GOTERM_BP_ALL	GO:0015849~organic acid transport	4
GOTERM_BP_ALL	GO:0046942~carboxylic acid transport	4
GOTERM_MF_ALL	GO:0016788~hydrolase activity, acting on ester bonds	51
GOTERM_BP_ALL	GO:0016458~gene silencing	6
GOTERM_BP_ALL	GO:0031047~gene silencing by RNA	5
GOTERM_BP_ALL	GO:0016441~posttranscriptional gene silencing	5
GOTERM_MF_ALL	GO:0015299~solute:hydrogen antiporter activity	3
GOTERM_BP_ALL	GO:0019761~glucosinolate biosynthetic process	2
GOTERM_BP_ALL	GO:0042219~cellular amino acid derivative catabolic pro	2
GOTERM_BP_ALL	GO:0016144~S-glycoside biosynthetic process	2
GOTERM_BP_ALL	GO:0019758~glycosinolate biosynthetic process	2
GOTERM_BP_ALL	GO:0045165~cell fate commitment	2
GOTERM_MF_ALL	GO:0019787~small conjugating protein ligase activity	13
GOTERM_CC_ALL	GO:0044464~cell part	904
GOTERM_CC_ALL	GO:0005623~cell	904
GOTERM_CC_ALL	GO:0005798~Golgi-associated vesicle	2
GOTERM_MF_ALL	GO:0016671~oxidoreductase activity, acting on sulfur gro	2
GOTERM_BP_ALL	GO:0048467~gynoecium development	3
GOTERM_BP_ALL	GO:0048581~negative regulation of post-embryonic deve	3
GOTERM_BP_ALL	GO:0009637~response to blue light	3
GOTERM_BP_ALL	GO:0034637~cellular carbohydrate biosynthetic process	9
GOTERM_MF_ALL	GO:0016782~transferase activity, transferring sulfur-cont	2
GOTERM_CC_ALL	GO:0044459~plasma membrane part	13

GOTERM_CC_ALL	GO:0005667~transcription factor complex	3
GOTERM_BP_ALL	GO:0009409~response to cold	11
GOTERM_MF_ALL	GO:0005083~small GTPase regulator activity	4
GOTERM_BP_ALL	GO:0010087~phloem or xylem histogenesis	2
GOTERM_BP_ALL	GO:0034754~cellular hormone metabolic process	2
GOTERM_BP_ALL	GO:0051338~regulation of transferase activity	2
GOTERM_BP_ALL	GO:0006612~protein targeting to membrane	2
GOTERM_CC_ALL	GO:0019005~SCF ubiquitin ligase complex	2
GOTERM_MF_ALL	GO:0051537~2 iron, 2 sulfur cluster binding	2
GOTERM_BP_ALL	GO:0009056~catabolic process	65
GOTERM_BP_ALL	GO:0016116~carotenoid metabolic process	2
GOTERM_BP_ALL	GO:0006944~membrane fusion	2
GOTERM_BP_ALL	GO:0006760~folic acid and derivative metabolic process	2
GOTERM_BP_ALL	GO:0016108~tetraterpenoid metabolic process	2
GOTERM_MF_ALL	GO:0016810~hydrolase activity, acting on carbon-nitroge	5
GOTERM_BP_ALL	GO:0009624~response to nematode	3
GOTERM_BP_ALL	GO:0009892~negative regulation of metabolic process	9
GOTERM_BP_ALL	GO:0044272~sulfur compound biosynthetic process	5
GOTERM_BP_ALL	GO:0042325~regulation of phosphorylation	2
GOTERM_BP_ALL	GO:0009308~amine metabolic process	23
GOTERM_CC_ALL	GO:0042598~vesicular fraction	2
GOTERM_CC_ALL	GO:0005792~microsome	2
GOTERM_BP_ALL	GO:0006950~response to stress	120
GOTERM_MF_ALL	GO:0008081~phosphoric diester hydrolase activity	3
GOTERM_MF_ALL	GO:0016811~hydrolase activity, acting on carbon-nitroge	3
GOTERM_MF_ALL	GO:0015103~inorganic anion transmembrane transporter	3
GOTERM_BP_ALL	GO:0009150~purine ribonucleotide metabolic process	6
GOTERM_BP_ALL	GO:0009152~purine ribonucleotide biosynthetic process	6
GOTERM_BP_ALL	GO:0006754~ATP biosynthetic process	5
GOTERM_BP_ALL	GO:0046034~ATP metabolic process	5
GOTERM_BP_ALL	GO:0009856~pollination	6
GOTERM_MF_ALL	GO:0030695~GTPase regulator activity	4
GOTERM_MF_ALL	GO:0008270~zinc ion binding	81
GOTERM_MF_ALL	GO:0016278~lysine N-methyltransferase activity	2
GOTERM_MF_ALL	GO:0051539~4 iron, 4 sulfur cluster binding	2
GOTERM_MF_ALL	GO:0003712~transcription cofactor activity	2
GOTERM_MF_ALL	GO:0018024~histone-lysine N-methyltransferase activity	2
GOTERM_MF_ALL	GO:0016279~protein-lysine N-methyltransferase activity	2
GOTERM_MF_ALL	GO:0015385~sodium:hydrogen antiporter activity	2
GOTERM_MF_ALL	GO:0008092~cytoskeletal protein binding	5
GOTERM_BP_ALL	GO:0016125~sterol metabolic process	2
GOTERM_BP_ALL	GO:0009933~meristem structural organization	2
GOTERM_BP_ALL	GO:0031324~negative regulation of cellular metabolic pr	4
GOTERM_BP_ALL	GO:0006952~defense response	50
GOTERM_MF_ALL	GO:0008415~acyltransferase activity	10
GOTERM_MF_ALL	GO:0003954~NADH dehydrogenase activity	2
GOTERM_BP_ALL	GO:0046483~heterocycle metabolic process	20

GOTERM_BP_ALL	GO:0009888~tissue development	11
GOTERM_BP_ALL	GO:0009793~embryonic development ending in seed dor	17
GOTERM_BP_ALL	GO:0019220~regulation of phosphate metabolic process	2
GOTERM_BP_ALL	GO:0009744~response to sucrose stimulus	2
GOTERM_BP_ALL	GO:0051174~regulation of phosphorus metabolic process	2
GOTERM_BP_ALL	GO:0006643~membrane lipid metabolic process	2
GOTERM_BP_ALL	GO:0006281~DNA repair	8
GOTERM_MF_ALL	GO:0015171~amino acid transmembrane transporter activ	3
GOTERM_MF_ALL	GO:0051536~iron-sulfur cluster binding	4
GOTERM_MF_ALL	GO:0051540~metal cluster binding	4
GOTERM_CC_ALL	GO:0031968~organelle outer membrane	3
GOTERM_BP_ALL	GO:0006164~purine nucleotide biosynthetic process	6
GOTERM_BP_ALL	GO:0042435~indole derivative biosynthetic process	2
GOTERM_BP_ALL	GO:0034285~response to disaccharide stimulus	2
GOTERM_BP_ALL	GO:0009199~ribonucleoside triphosphate metabolic proc	5
GOTERM_BP_ALL	GO:0009144~purine nucleoside triphosphate metabolic pr	5
GOTERM_BP_ALL	GO:0009201~ribonucleoside triphosphate biosynthetic pr	5
GOTERM_BP_ALL	GO:0009206~purine ribonucleoside triphosphate biosynth	5
GOTERM_BP_ALL	GO:0009145~purine nucleoside triphosphate biosynthetic	5
GOTERM_BP_ALL	GO:0009205~purine ribonucleoside triphosphate metabol	5
GOTERM_MF_ALL	GO:0016831~carboxy-lyase activity	3
GOTERM_BP_ALL	GO:0009057~macromolecule catabolic process	32
GOTERM_MF_ALL	GO:0046982~protein heterodimerization activity	2
GOTERM_BP_ALL	GO:0009142~nucleoside triphosphate biosynthetic proces	5
GOTERM_BP_ALL	GO:0045595~regulation of cell differentiation	2
GOTERM_BP_ALL	GO:0043094~cellular metabolic compound salvage	3
GOTERM_BP_ALL	GO:0006865~amino acid transport	3
GOTERM_BP_ALL	GO:0009141~nucleoside triphosphate metabolic process	5
GOTERM_CC_ALL	GO:0005624~membrane fraction	2
GOTERM_CC_ALL	GO:0005626~insoluble fraction	2
GOTERM_MF_ALL	GO:0042054~histone methyltransferase activity	2
GOTERM_BP_ALL	GO:0009260~ribonucleotide biosynthetic process	6
GOTERM_BP_ALL	GO:0006163~purine nucleotide metabolic process	6
GOTERM_MF_ALL	GO:0060589~nucleoside-triphosphatase regulator activity	4
GOTERM_BP_ALL	GO:0015837~amine transport	3
GOTERM_BP_ALL	GO:0009251~glucan catabolic process	2
GOTERM_BP_ALL	GO:0009607~response to biotic stimulus	41
GOTERM_BP_ALL	GO:0016051~carbohydrate biosynthetic process	11
GOTERM_BP_ALL	GO:0009891~positive regulation of biosynthetic process	3
GOTERM_BP_ALL	GO:0031328~positive regulation of cellular biosynthetic p	3
GOTERM_BP_ALL	GO:0009259~ribonucleotide metabolic process	6
GOTERM_BP_ALL	GO:0044248~cellular catabolic process	44
GOTERM_BP_ALL	GO:0006974~response to DNA damage stimulus	8
GOTERM_CC_ALL	GO:0009705~plant-type vacuole membrane	2
GOTERM_BP_ALL	GO:0009909~regulation of flower development	4
GOTERM_MF_ALL	GO:0042623~ATPase activity, coupled	15
GOTERM_CC_ALL	GO:0019867~outer membrane	3

GOTERM_MF_ALL	GO:0016879~ligase activity, forming carbon-nitrogen bon	15
GOTERM_BP_ALL	GO:0000910~cytokinesis	2
GOTERM_BP_ALL	GO:0035195~gene silencing by miRNA	3
GOTERM_BP_ALL	GO:0051603~proteolysis involved in cellular protein cata	25
GOTERM_BP_ALL	GO:0030163~protein catabolic process	26
GOTERM_BP_ALL	GO:0010154~fruit development	20
GOTERM_BP_ALL	GO:0009066~aspartate family amino acid metabolic proce	3
GOTERM_BP_ALL	GO:0042430~indole and derivative metabolic process	2
GOTERM_BP_ALL	GO:0042434~indole derivative metabolic process	2
GOTERM_CC_ALL	GO:0012506~vesicle membrane	2
GOTERM_MF_ALL	GO:0016597~amino acid binding	2
GOTERM_MF_ALL	GO:0008757~S-adenosylmethionine-dependent methyltra	5
GOTERM_BP_ALL	GO:0006260~DNA replication	5
GOTERM_BP_ALL	GO:0009411~response to UV	3
GOTERM_BP_ALL	GO:0006790~sulfur metabolic process	7
GOTERM_BP_ALL	GO:0009067~aspartate family amino acid biosynthetic pr	2
GOTERM_BP_ALL	GO:0022603~regulation of anatomical structure morphog	2
GOTERM_BP_ALL	GO:0030258~lipid modification	2
GOTERM_BP_ALL	GO:0005984~disaccharide metabolic process	2
GOTERM_MF_ALL	GO:0004620~phospholipase activity	2
GOTERM_MF_ALL	GO:0034061~DNA polymerase activity	2
GOTERM_MF_ALL	GO:0043176~amine binding	2
GOTERM_CC_ALL	GO:0000267~cell fraction	2
GOTERM_BP_ALL	GO:0044257~cellular protein catabolic process	25
GOTERM_MF_ALL	GO:0016765~transferase activity, transferring alkyl or ary	5
GOTERM_BP_ALL	GO:0000904~cell morphogenesis involved in differentiati	4
GOTERM_CC_ALL	GO:0000151~ubiquitin ligase complex	10
GOTERM_BP_ALL	GO:0016143~S-glycoside metabolic process	2
GOTERM_BP_ALL	GO:0019760~glucosinolate metabolic process	2
GOTERM_BP_ALL	GO:0019757~glycosinolate metabolic process	2
GOTERM_BP_ALL	GO:0034504~protein localization in nucleus	2
GOTERM_MF_ALL	GO:0005451~monovalent cation:hydrogen antiporter acti	2
GOTERM_BP_ALL	GO:0030029~actin filament-based process	3
GOTERM_MF_ALL	GO:0016887~ATPase activity	20
GOTERM_BP_ALL	GO:0042440~pigment metabolic process	4
GOTERM_MF_ALL	GO:0004888~transmembrane receptor activity	8
GOTERM_BP_ALL	GO:0048868~pollen tube development	3
GOTERM_BP_ALL	GO:0035295~tube development	3
GOTERM_BP_ALL	GO:0009250~glucan biosynthetic process	3
GOTERM_MF_ALL	GO:0004428~inositol or phosphatidylinositol kinase activ	2
GOTERM_CC_ALL	GO:0030964~NADH dehydrogenase complex	2
GOTERM_CC_ALL	GO:0045271~respiratory chain complex I	2
GOTERM_BP_ALL	GO:0051707~response to other organism	37
GOTERM_MF_ALL	GO:0008047~enzyme activator activity	3
GOTERM_BP_ALL	GO:0055086~nucleobase, nucleoside and nucleotide meta	11
GOTERM_BP_ALL	GO:0044265~cellular macromolecule catabolic process	26
GOTERM_BP_ALL	GO:0007018~microtubule-based movement	3

GOTERM_BP_ALL	GO:0006807~nitrogen compound metabolic process	151
GOTERM_BP_ALL	GO:0009555~pollen development	5
GOTERM_BP_ALL	GO:0040029~regulation of gene expression, epigenetic	6
GOTERM_CC_ALL	GO:0005777~peroxisome	7
GOTERM_CC_ALL	GO:0042579~microbody	7
GOTERM_BP_ALL	GO:0019941~modification-dependent protein catabolic pr	24
GOTERM_BP_ALL	GO:0043632~modification-dependent macromolecule cat	24
GOTERM_MF_ALL	GO:0016667~oxidoreductase activity, acting on sulfur gro	4
GOTERM_BP_ALL	GO:0018193~peptidyl-amino acid modification	2
GOTERM_BP_ALL	GO:0006779~porphyrin biosynthetic process	2
GOTERM_BP_ALL	GO:0009310~amine catabolic process	2
GOTERM_MF_ALL	GO:0003702~RNA polymerase II transcription factor acti	2
GOTERM_MF_ALL	GO:0008276~protein methyltransferase activity	2
GOTERM_CC_ALL	GO:0044455~mitochondrial membrane part	3
GOTERM_CC_ALL	GO:0005886~plasma membrane	109
GOTERM_CC_ALL	GO:0031988~membrane-bounded vesicle	3
GOTERM_CC_ALL	GO:0009528~plastid inner membrane	2
GOTERM_BP_ALL	GO:0018130~heterocycle biosynthetic process	5
GOTERM_BP_ALL	GO:0016044~membrane organization	4
GOTERM_BP_ALL	GO:0006555~methionine metabolic process	2
GOTERM_BP_ALL	GO:0031325~positive regulation of cellular metabolic pro	3
GOTERM_MF_ALL	GO:0030145~manganese ion binding	10
GOTERM_BP_ALL	GO:0034404~nucleobase, nucleoside and nucleotide bios	7
GOTERM_BP_ALL	GO:0034654~nucleobase, nucleoside, nucleotide and nucl	7
GOTERM_BP_ALL	GO:0006633~fatty acid biosynthetic process	6
GOTERM_BP_ALL	GO:0008219~cell death	10
GOTERM_BP_ALL	GO:0016265~death	10
GOTERM_BP_ALL	GO:0033014~tetrapyrrole biosynthetic process	2
GOTERM_BP_ALL	GO:0009893~positive regulation of metabolic process	3
GOTERM_MF_ALL	GO:0015450~P-P-bond-hydrolysis-driven protein transme	2
GOTERM_MF_ALL	GO:0022884~macromolecule transmembrane transporter	2
GOTERM_BP_ALL	GO:0048316~seed development	18
GOTERM_BP_ALL	GO:0051641~cellular localization	21
GOTERM_BP_ALL	GO:0006139~nucleobase, nucleoside, nucleotide and nucl	121
GOTERM_BP_ALL	GO:0016043~cellular component organization	58
GOTERM_BP_ALL	GO:0006351~transcription, DNA-dependent	3
GOTERM_MF_ALL	GO:0016866~intramolecular transferase activity	2
GOTERM_CC_ALL	GO:0031461~cullin-RING ubiquitin ligase complex	6
GOTERM_BP_ALL	GO:0048229~gametophyte development	7
GOTERM_BP_ALL	GO:0016137~glycoside metabolic process	3
GOTERM_BP_ALL	GO:0009790~embryonic development	17
GOTERM_BP_ALL	GO:0051704~multi-organism process	44
GOTERM_MF_ALL	GO:0019842~vitamin binding	6
GOTERM_BP_ALL	GO:0009581~detection of external stimulus	2
GOTERM_BP_ALL	GO:0006352~transcription initiation	2
GOTERM_MF_ALL	GO:0015035~protein disulfide oxidoreductase activity	2
GOTERM_MF_ALL	GO:0008170~N-methyltransferase activity	2

GOTERM_BP_ALL	GO:0009913~epidermal cell differentiation	4
GOTERM_BP_ALL	GO:0032774~RNA biosynthetic process	3
GOTERM_MF_ALL	GO:0004519~endonuclease activity	3
GOTERM_MF_ALL	GO:0016651~oxidoreductase activity, acting on NADH o	3
GOTERM_BP_ALL	GO:0030036~actin cytoskeleton organization	2
GOTERM_MF_ALL	GO:0016830~carbon-carbon lyase activity	4
GOTERM_BP_ALL	GO:0007398~ectoderm development	4
GOTERM_BP_ALL	GO:0008544~epidermis development	4
GOTERM_BP_ALL	GO:0045941~positive regulation of transcription	2
GOTERM_BP_ALL	GO:0046148~pigment biosynthetic process	3
GOTERM_BP_ALL	GO:0010628~positive regulation of gene expression	2
GOTERM_MF_ALL	GO:0003755~peptidyl-prolyl cis-trans isomerase activity	2
GOTERM_BP_ALL	GO:0045454~cell redox homeostasis	5
GOTERM_BP_ALL	GO:0016192~vesicle-mediated transport	11
GOTERM_BP_ALL	GO:0009165~nucleotide biosynthetic process	6
GOTERM_BP_ALL	GO:0006066~alcohol metabolic process	11
GOTERM_BP_ALL	GO:0044242~cellular lipid catabolic process	2
GOTERM_BP_ALL	GO:0009812~flavonoid metabolic process	2
GOTERM_MF_ALL	GO:0016859~cis-trans isomerase activity	2
GOTERM_BP_ALL	GO:0010608~posttranscriptional regulation of gene expre	5
GOTERM_CC_ALL	GO:0042170~plastid membrane	3
GOTERM_CC_ALL	GO:0005874~microtubule	4
GOTERM_BP_ALL	GO:0012501~programmed cell death	8
GOTERM_BP_ALL	GO:0009072~aromatic amino acid family metabolic proce	2
GOTERM_MF_ALL	GO:0030955~potassium ion binding	2
GOTERM_BP_ALL	GO:0007017~microtubule-based process	4
GOTERM_MF_ALL	GO:0031406~carboxylic acid binding	3
GOTERM_BP_ALL	GO:0009064~glutamine family amino acid metabolic proc	2
GOTERM_MF_ALL	GO:0008320~protein transmembrane transporter activity	2
GOTERM_MF_ALL	GO:0016462~pyrophosphatase activity	35
GOTERM_CC_ALL	GO:0080008~CUL4 RING ubiquitin ligase complex	4
GOTERM_MF_ALL	GO:0015036~disulfide oxidoreductase activity	2
GOTERM_BP_ALL	GO:0006778~porphyrin metabolic process	2
GOTERM_BP_ALL	GO:0043623~cellular protein complex assembly	4
GOTERM_BP_ALL	GO:0016138~glycoside biosynthetic process	2
GOTERM_BP_ALL	GO:0033013~tetrapyrrole metabolic process	2
GOTERM_MF_ALL	GO:0032561~guanyl ribonucleotide binding	10
GOTERM_MF_ALL	GO:0017111~nucleoside-triphosphatase activity	33
GOTERM_BP_ALL	GO:0006511~ubiquitin-dependent protein catabolic proce	10
GOTERM_BP_ALL	GO:0006839~mitochondrial transport	2
GOTERM_BP_ALL	GO:0045935~positive regulation of nucleobase, nucleosid	2
GOTERM_BP_ALL	GO:0051173~positive regulation of nitrogen compound m	2
GOTERM_BP_ALL	GO:0010557~positive regulation of macromolecule biosy	2
GOTERM_BP_ALL	GO:0006725~cellular aromatic compound metabolic proc	13
GOTERM_CC_ALL	GO:0000786~nucleosome	2
GOTERM_BP_ALL	GO:0006575~cellular amino acid derivative metabolic pro	10
GOTERM_BP_ALL	GO:0009117~nucleotide metabolic process	8

GOTERM_BP_ALL	GO:0006753~nucleoside phosphate metabolic process	8
GOTERM_MF_ALL	GO:0016818~hydrolase activity, acting on acid anhydride	35
GOTERM_MF_ALL	GO:0019001~guanyl nucleotide binding	10
GOTERM_CC_ALL	GO:0005654~nucleoplasm	5
GOTERM_MF_ALL	GO:0008234~cysteine-type peptidase activity	5
GOTERM_BP_ALL	GO:0030243~cellulose metabolic process	2
GOTERM_MF_ALL	GO:0000287~magnesium ion binding	12
GOTERM_BP_ALL	GO:0009266~response to temperature stimulus	13
GOTERM_MF_ALL	GO:0016817~hydrolase activity, acting on acid anhydride	35
GOTERM_MF_ALL	GO:0004518~nuclease activity	6
GOTERM_BP_ALL	GO:0070271~protein complex biogenesis	6
GOTERM_BP_ALL	GO:0006461~protein complex assembly	6
GOTERM_MF_ALL	GO:0015077~monovalent inorganic cation transmembran	5
GOTERM_BP_ALL	GO:0010035~response to inorganic substance	21
GOTERM_CC_ALL	GO:0030135~coated vesicle	2
GOTERM_BP_ALL	GO:0034641~cellular nitrogen compound metabolic proce	140
GOTERM_MF_ALL	GO:0008565~protein transporter activity	4
GOTERM_CC_ALL	GO:0031982~vesicle	3
GOTERM_BP_ALL	GO:0006334~nucleosome assembly	2
GOTERM_BP_ALL	GO:0010604~positive regulation of macromolecule metab	2
GOTERM_BP_ALL	GO:0034728~nucleosome organization	2
GOTERM_MF_ALL	GO:0016903~oxidoreductase activity, acting on the aldeh	2
GOTERM_BP_ALL	GO:0048522~positive regulation of cellular process	4
GOTERM_CC_ALL	GO:0032993~protein-DNA complex	2
GOTERM_BP_ALL	GO:0031497~chromatin assembly	2
GOTERM_BP_ALL	GO:0044106~cellular amine metabolic process	15
GOTERM_MF_ALL	GO:0016627~oxidoreductase activity, acting on the CH-C	2
GOTERM_BP_ALL	GO:0065004~protein-DNA complex assembly	2
GOTERM_MF_ALL	GO:0051082~unfolded protein binding	3
GOTERM_BP_ALL	GO:0009642~response to light intensity	2
GOTERM_MF_ALL	GO:0008168~methyltransferase activity	8
GOTERM_BP_ALL	GO:0000096~sulfur amino acid metabolic process	2
GOTERM_CC_ALL	GO:0044451~nucleoplasm part	4
GOTERM_MF_ALL	GO:0016741~transferase activity, transferring one-carbon	8
GOTERM_BP_ALL	GO:0006259~DNA metabolic process	12
GOTERM_BP_ALL	GO:0008380~RNA splicing	3
GOTERM_BP_ALL	GO:0034621~cellular macromolecular complex subunit o	7
GOTERM_MF_ALL	GO:0016874~ligase activity	18
GOTERM_BP_ALL	GO:0009553~embryo sac development	2
GOTERM_BP_ALL	GO:0006323~DNA packaging	2
GOTERM_MF_ALL	GO:0015078~hydrogen ion transmembrane transporter ac	4
GOTERM_CC_ALL	GO:0031966~mitochondrial membrane	7
GOTERM_CC_ALL	GO:0016023~cytoplasmic membrane-bounded vesicle	2
GOTERM_CC_ALL	GO:0009543~chloroplast thylakoid lumen	2
GOTERM_CC_ALL	GO:0031978~plastid thylakoid lumen	2
GOTERM_CC_ALL	GO:0005768~endosome	2
GOTERM_MF_ALL	GO:0030599~pectinesterase activity	4

GOTERM_BP_ALL	GO:0006818~hydrogen transport	2
GOTERM_BP_ALL	GO:0015992~proton transport	2
GOTERM_MF_ALL	GO:0005525~GTP binding	8
GOTERM_BP_ALL	GO:0034613~cellular protein localization	9
GOTERM_BP_ALL	GO:0016053~organic acid biosynthetic process	14
GOTERM_BP_ALL	GO:0046394~carboxylic acid biosynthetic process	14
GOTERM_BP_ALL	GO:0051649~establishment of localization in cell	16
GOTERM_BP_ALL	GO:0034622~cellular macromolecular complex assembly	6
GOTERM_BP_ALL	GO:0043933~macromolecular complex subunit organizati	9
GOTERM_BP_ALL	GO:0046395~carboxylic acid catabolic process	2
GOTERM_BP_ALL	GO:0016054~organic acid catabolic process	2
GOTERM_BP_ALL	GO:0022607~cellular component assembly	10
GOTERM_CC_ALL	GO:0019866~organelle inner membrane	7
GOTERM_MF_ALL	GO:0005543~phospholipid binding	2
GOTERM_BP_ALL	GO:0016071~mRNA metabolic process	4
GOTERM_BP_ALL	GO:0005996~monosaccharide metabolic process	5
GOTERM_BP_ALL	GO:0006631~fatty acid metabolic process	6
GOTERM_MF_ALL	GO:0008237~metallopeptidase activity	2
GOTERM_CC_ALL	GO:0005740~mitochondrial envelope	7
GOTERM_BP_ALL	GO:0065003~macromolecular complex assembly	8
GOTERM_MF_ALL	GO:0031072~heat shock protein binding	3
GOTERM_BP_ALL	GO:0016567~protein ubiquitination	3
GOTERM_CC_ALL	GO:0005743~mitochondrial inner membrane	5
GOTERM_CC_ALL	GO:0005856~cytoskeleton	7
GOTERM_BP_ALL	GO:0019318~hexose metabolic process	4
GOTERM_BP_ALL	GO:0051188~cofactor biosynthetic process	4
GOTERM_BP_ALL	GO:0009108~coenzyme biosynthetic process	2
GOTERM_CC_ALL	GO:0015630~microtubule cytoskeleton	4
GOTERM_CC_ALL	GO:0031977~thylakoid lumen	2
GOTERM_CC_ALL	GO:0044430~cytoskeletal part	5
GOTERM_BP_ALL	GO:0070727~cellular macromolecule localization	9
GOTERM_CC_ALL	GO:0000785~chromatin	2
GOTERM_BP_ALL	GO:0006333~chromatin assembly or disassembly	2
GOTERM_BP_ALL	GO:0008104~protein localization	17
GOTERM_MF_ALL	GO:0003774~motor activity	2
GOTERM_BP_ALL	GO:0032446~protein modification by small protein conju	3
GOTERM_BP_ALL	GO:0022402~cell cycle process	3
GOTERM_BP_ALL	GO:0006397~mRNA processing	3
GOTERM_BP_ALL	GO:0043436~oxoacid metabolic process	28
GOTERM_BP_ALL	GO:0019752~carboxylic acid metabolic process	28
GOTERM_BP_ALL	GO:0006082~organic acid metabolic process	28
GOTERM_BP_ALL	GO:0016568~chromatin modification	3
GOTERM_CC_ALL	GO:0070469~respiratory chain	2
GOTERM_BP_ALL	GO:0016052~carbohydrate catabolic process	5
GOTERM_BP_ALL	GO:0022900~electron transport chain	4
GOTERM_CC_ALL	GO:0031410~cytoplasmic vesicle	2
GOTERM_MF_ALL	GO:0016779~nucleotidyltransferase activity	4

GOTERM_BP_ALL	GO:0042180~cellular ketone metabolic process	28
GOTERM_BP_ALL	GO:0050832~defense response to fungus	12
GOTERM_MF_ALL	GO:0030234~enzyme regulator activity	10
GOTERM_MF_ALL	GO:0004857~enzyme inhibitor activity	4
GOTERM_BP_ALL	GO:0044271~nitrogen compound biosynthetic process	16
GOTERM_BP_ALL	GO:0019438~aromatic compound biosynthetic process	5
GOTERM_BP_ALL	GO:0070647~protein modification by small protein conju	3
GOTERM_BP_ALL	GO:0007010~cytoskeleton organization	2
GOTERM_BP_ALL	GO:0006730~one-carbon metabolic process	2
GOTERM_BP_ALL	GO:0009620~response to fungus	13
GOTERM_MF_ALL	GO:0008026~ATP-dependent helicase activity	2
GOTERM_MF_ALL	GO:0070035~purine NTP-dependent helicase activity	2
GOTERM_BP_ALL	GO:0006325~chromatin organization	5
GOTERM_BP_ALL	GO:0051726~regulation of cell cycle	2
GOTERM_BP_ALL	GO:0006006~glucose metabolic process	2
GOTERM_CC_ALL	GO:0044427~chromosomal part	3
GOTERM_BP_ALL	GO:0042398~cellular amino acid derivative biosynthetic	4
GOTERM_BP_ALL	GO:0009698~phenylpropanoid metabolic process	3
GOTERM_BP_ALL	GO:0045184~establishment of protein localization	14
GOTERM_BP_ALL	GO:0015031~protein transport	14
GOTERM_MF_ALL	GO:0016614~oxidoreductase activity, acting on CH-OH g	3
GOTERM_BP_ALL	GO:0006520~cellular amino acid metabolic process	10
GOTERM_BP_ALL	GO:0009309~amine biosynthetic process	4
GOTERM_BP_ALL	GO:0009408~response to heat	2
GOTERM_BP_ALL	GO:0051301~cell division	3
GOTERM_BP_ALL	GO:0009699~phenylpropanoid biosynthetic process	2
GOTERM_BP_ALL	GO:0006886~intracellular protein transport	6
GOTERM_BP_ALL	GO:0006399~tRNA metabolic process	2
GOTERM_BP_ALL	GO:0006605~protein targeting	2
GOTERM_BP_ALL	GO:0009451~RNA modification	2
GOTERM_BP_ALL	GO:0046907~intracellular transport	10
GOTERM_CC_ALL	GO:0044429~mitochondrial part	7
GOTERM_BP_ALL	GO:0043170~macromolecule metabolic process	292
GOTERM_BP_ALL	GO:0006519~cellular amino acid and derivative metaboli	18
GOTERM_CC_ALL	GO:0031090~organelle membrane	28
GOTERM_BP_ALL	GO:0008652~cellular amino acid biosynthetic process	3
GOTERM_MF_ALL	GO:0016616~oxidoreductase activity, acting on the CH-O	2
GOTERM_BP_ALL	GO:0051276~chromosome organization	5
GOTERM_BP_ALL	GO:0008152~metabolic process	492
GOTERM_BP_ALL	GO:0001906~cell killing	6
GOTERM_BP_ALL	GO:0031640~killing of cells of another organism	6
GOTERM_CC_ALL	GO:0005634~nucleus	145
GOTERM_BP_ALL	GO:0006732~coenzyme metabolic process	3
GOTERM_BP_ALL	GO:0007049~cell cycle	4
GOTERM_BP_ALL	GO:0044238~primary metabolic process	397
GOTERM_BP_ALL	GO:0051186~cofactor metabolic process	5
GOTERM_BP_ALL	GO:0006457~protein folding	4

GOTERM_CC_ALL	GO:0005694~chromosome	3
GOTERM_BP_ALL	GO:0006915~apoptosis	2
GOTERM_MF_ALL	GO:0004386~helicase activity	2
GOTERM_BP_ALL	GO:0044260~cellular macromolecule metabolic process	254
GOTERM_BP_ALL	GO:0019538~protein metabolic process	169
GOTERM_CC_ALL	GO:0005739~mitochondrion	45
GOTERM_BP_ALL	GO:0010038~response to metal ion	7
GOTERM_CC_ALL	GO:0031984~organelle subcompartment	6
GOTERM_BP_ALL	GO:0044085~cellular component biogenesis	15
GOTERM_CC_ALL	GO:0044436~thylakoid part	6
GOTERM_CC_ALL	GO:0042651~thylakoid membrane	4
GOTERM_BP_ALL	GO:0006996~organelle organization	13
GOTERM_CC_ALL	GO:0043234~protein complex	44
GOTERM_CC_ALL	GO:0031976~plastid thylakoid	5
GOTERM_CC_ALL	GO:0009534~chloroplast thylakoid	5
GOTERM_BP_ALL	GO:0034645~cellular macromolecule biosynthetic proces	108
GOTERM_MF_ALL	GO:0005198~structural molecule activity	10
GOTERM_BP_ALL	GO:0009059~macromolecule biosynthetic process	108
GOTERM_BP_ALL	GO:0006396~RNA processing	7
GOTERM_BP_ALL	GO:0016070~RNA metabolic process	14
GOTERM_BP_ALL	GO:0046686~response to cadmium ion	4
GOTERM_BP_ALL	GO:0006091~generation of precursor metabolites and ene	6
GOTERM_CC_ALL	GO:0034357~photosynthetic membrane	4
GOTERM_CC_ALL	GO:0009535~chloroplast thylakoid membrane	3
GOTERM_CC_ALL	GO:0055035~plastid thylakoid membrane	3
GOTERM_CC_ALL	GO:0009579~thylakoid	8
GOTERM_CC_ALL	GO:0009526~plastid envelope	7
GOTERM_BP_ALL	GO:0034660~ncRNA metabolic process	2
GOTERM_BP_ALL	GO:0044267~cellular protein metabolic process	135
GOTERM_CC_ALL	GO:0031981~nuclear lumen	7
GOTERM_CC_ALL	GO:0009941~chloroplast envelope	6
GOTERM_CC_ALL	GO:0005829~cytosol	13
GOTERM_MF_ALL	GO:0003676~nucleic acid binding	161
GOTERM_CC_ALL	GO:0031975~envelope	14
GOTERM_CC_ALL	GO:0043233~organelle lumen	11
GOTERM_CC_ALL	GO:0070013~intracellular organelle lumen	11
GOTERM_CC_ALL	GO:0044428~nuclear part	10
GOTERM_CC_ALL	GO:0031974~membrane-enclosed lumen	11
GOTERM_BP_ALL	GO:0009058~biosynthetic process	163
GOTERM_CC_ALL	GO:0009570~chloroplast stroma	4
GOTERM_BP_ALL	GO:0044237~cellular metabolic process	347
GOTERM_CC_ALL	GO:0031967~organelle envelope	13
GOTERM_CC_ALL	GO:0005730~nucleolus	2
GOTERM_CC_ALL	GO:0009532~plastid stroma	4
GOTERM_BP_ALL	GO:0044249~cellular biosynthetic process	151
GOTERM_BP_ALL	GO:0010467~gene expression	94
GOTERM_BP_ALL	GO:0009987~cellular process	452

GOTERM_CC_ALL	GO:0044435~plastid part	16
GOTERM_CC_ALL	GO:0044434~chloroplast part	15
GOTERM_CC_ALL	GO:0043227~membrane-bounded organelle	372
GOTERM_CC_ALL	GO:0044444~cytoplasmic part	258
GOTERM_CC_ALL	GO:0043231~intracellular membrane-bounded organelle	371
GOTERM_CC_ALL	GO:0005840~ribosome	2
GOTERM_CC_ALL	GO:0030529~ribonucleoprotein complex	5
GOTERM_CC_ALL	GO:0005737~cytoplasm	281
GOTERM_CC_ALL	GO:0009507~chloroplast	95
GOTERM_CC_ALL	GO:0032991~macromolecular complex	50
GOTERM_MF_ALL	GO:0003723~RNA binding	22
GOTERM_CC_ALL	GO:0009536~plastid	97
GOTERM_CC_ALL	GO:0043226~organelle	377
GOTERM_CC_ALL	GO:0043229~intracellular organelle	376
GOTERM_CC_ALL	GO:0043232~intracellular non-membrane-bounded organ	14
GOTERM_CC_ALL	GO:0043228~non-membrane-bounded organelle	14
GOTERM_MF_ALL	GO:0033897~ribonuclease T2 activity	1
GOTERM_MF_ALL	GO:0008169~C-methyltransferase activity	1
GOTERM_MF_ALL	GO:0005201~extracellular matrix structural constituent	1
GOTERM_MF_ALL	GO:0004012~phospholipid-translocating ATPase activity	1
GOTERM_MF_ALL	GO:0042947~glucoside transmembrane transporter activit	1
GOTERM_MF_ALL	GO:0015928~fucosidase activity	1
GOTERM_MF_ALL	GO:0080044~quercetin 7-O-glucosyltransferase activity	1
GOTERM_MF_ALL	GO:0000062~acyl-CoA binding	1
GOTERM_MF_ALL	GO:0032451~demethylase activity	1
GOTERM_MF_ALL	GO:0005363~maltose transmembrane transporter activity	1
GOTERM_MF_ALL	GO:0015247~aminophospholipid transporter activity	1
GOTERM_MF_ALL	GO:0035004~phosphoinositide 3-kinase activity	1
GOTERM_MF_ALL	GO:0004424~imidazoleglycerol-phosphate dehydratase ac	1
GOTERM_MF_ALL	GO:0047720~indoleacetaldoxime dehydratase activity	1
GOTERM_MF_ALL	GO:0004057~arginyltransferase activity	1
GOTERM_MF_ALL	GO:0004527~exonuclease activity	1
GOTERM_MF_ALL	GO:0008080~N-acetyltransferase activity	1
GOTERM_MF_ALL	GO:0050505~hydroquinone glucosyltransferase activity	1
GOTERM_MF_ALL	GO:0005372~water transporter activity	1
GOTERM_MF_ALL	GO:0008235~metalloexopeptidase activity	1
GOTERM_MF_ALL	GO:0016624~oxidoreductase activity, acting on the aldeh	1
GOTERM_MF_ALL	GO:0030414~peptidase inhibitor activity	1
GOTERM_MF_ALL	GO:0051184~cofactor transporter activity	1
GOTERM_MF_ALL	GO:0004133~glycogen debranching enzyme activity	1
GOTERM_MF_ALL	GO:0016229~steroid dehydrogenase activity	1
GOTERM_MF_ALL	GO:0004345~glucose-6-phosphate dehydrogenase activity	1
GOTERM_MF_ALL	GO:0008805~carbon-monoxide oxygenase activity	1
GOTERM_MF_ALL	GO:0016307~phosphatidylinositol phosphate kinase activ	1
GOTERM_MF_ALL	GO:0016634~oxidoreductase activity, acting on the CH-C	1
GOTERM_MF_ALL	GO:0032791~lead ion binding	1
GOTERM_MF_ALL	GO:0022820~potassium ion symporter activity	1

GOTERM_MF_ALL	GO:0031403~lithium ion binding	1
GOTERM_MF_ALL	GO:0015151~alpha-glucoside transmembrane transporter	1
GOTERM_MF_ALL	GO:0015326~cationic amino acid transmembrane transpo	1
GOTERM_MF_ALL	GO:0047364~desulfoglucosinolate sulfotransferase activit	1
GOTERM_MF_ALL	GO:0010436~carotenoid dioxygenase activity	1
GOTERM_MF_ALL	GO:0008891~glycolate oxidase activity	1
GOTERM_MF_ALL	GO:0019144~ADP-sugar diphosphatase activity	1
GOTERM_MF_ALL	GO:0008716~D-alanine-D-alanine ligase activity	1
GOTERM_MF_ALL	GO:0046524~sucrose-phosphate synthase activity	1
GOTERM_MF_ALL	GO:0003865~3-oxo-5-alpha-steroid 4-dehydrogenase acti	1
GOTERM_MF_ALL	GO:0080043~quercetin 3-O-glucosyltransferase activity	1
GOTERM_MF_ALL	GO:0043138~3'-5' DNA helicase activity	1
GOTERM_MF_ALL	GO:0047889~ferredoxin-nitrate reductase activity	1
GOTERM_MF_ALL	GO:0019210~kinase inhibitor activity	1
GOTERM_MF_ALL	GO:0016538~cyclin-dependent protein kinase regulator a	1
GOTERM_MF_ALL	GO:0080105~6-methylthiopropyl glucosinolate S-oxygen	1
GOTERM_MF_ALL	GO:0015142~tricarboxylic acid transmembrane transporte	1
GOTERM_MF_ALL	GO:0016774~phosphotransferase activity, carboxyl group	1
GOTERM_MF_ALL	GO:0015250~water channel activity	1
GOTERM_MF_ALL	GO:0031559~oxidosqualene cyclase activity	1
GOTERM_MF_ALL	GO:0030410~nicotianamine synthase activity	1
GOTERM_MF_ALL	GO:0080104~5-methylthiopropyl glucosinolate S-oxygen	1
GOTERM_MF_ALL	GO:0010297~heteroglycan binding	1
GOTERM_MF_ALL	GO:0008759~UDP-3-O-[3-hydroxymyristoyl] N-acetylglu	1
GOTERM_MF_ALL	GO:0005242~inward rectifier potassium channel activity	1
GOTERM_MF_ALL	GO:0004866~endopeptidase inhibitor activity	1
GOTERM_MF_ALL	GO:0004003~ATP-dependent DNA helicase activity	1
GOTERM_MF_ALL	GO:0004298~threonine-type endopeptidase activity	1
GOTERM_MF_ALL	GO:0016753~O-sinapoyltransferase activity	1
GOTERM_MF_ALL	GO:0004725~protein tyrosine phosphatase activity	1
GOTERM_MF_ALL	GO:0004747~ribokinase activity	1
GOTERM_MF_ALL	GO:0008417~fucosyltransferase activity	1
GOTERM_MF_ALL	GO:0004707~MAP kinase activity	1
GOTERM_MF_ALL	GO:0008094~DNA-dependent ATPase activity	1
GOTERM_MF_ALL	GO:0016875~ligase activity, forming carbon-oxygen bond	1
GOTERM_MF_ALL	GO:0000210~NAD+ diphosphatase activity	1
GOTERM_MF_ALL	GO:0010309~acireductone dioxygenase [iron(II)-requirin	1
GOTERM_MF_ALL	GO:0004812~aminoacyl-tRNA ligase activity	1
GOTERM_MF_ALL	GO:0017016~Ras GTPase binding	1
GOTERM_MF_ALL	GO:0015369~calcium:hydrogen antiporter activity	1
GOTERM_MF_ALL	GO:0016876~ligase activity, forming aminoacyl-tRNA an	1
GOTERM_MF_ALL	GO:0016917~GABA receptor activity	1
GOTERM_MF_ALL	GO:0003830~beta-1,4-mannosylglycoprotein 4-beta-N-ac	1
GOTERM_MF_ALL	GO:0005247~voltage-gated chloride channel activity	1
GOTERM_MF_ALL	GO:0004860~protein kinase inhibitor activity	1
GOTERM_MF_ALL	GO:0043140~ATP-dependent 3'-5' DNA helicase activity	1
GOTERM_MF_ALL	GO:0009982~pseudouridine synthase activity	1

GOTERM_MF_ALL	GO:0045551~cinnamyl-alcohol dehydrogenase activity	1
GOTERM_MF_ALL	GO:0009975~cyclase activity	1
GOTERM_MF_ALL	GO:0003678~DNA helicase activity	1
GOTERM_MF_ALL	GO:0031219~levanase activity	1
GOTERM_MF_ALL	GO:0015926~glucosidase activity	1
GOTERM_MF_ALL	GO:0016291~acyl-CoA thioesterase activity	1
GOTERM_MF_ALL	GO:0008937~ferredoxin reductase activity	1
GOTERM_MF_ALL	GO:0005200~structural constituent of cytoskeleton	1
GOTERM_MF_ALL	GO:0008381~mechanically-gated ion channel activity	1
GOTERM_MF_ALL	GO:0015137~citrate transmembrane transporter activity	1
GOTERM_MF_ALL	GO:0008508~bile acid:sodium symporter activity	1
GOTERM_MF_ALL	GO:0004437~inositol or phosphatidylinositol phosphatase	1
GOTERM_MF_ALL	GO:0008474~palmitoyl-(protein) hydrolase activity	1
GOTERM_MF_ALL	GO:0047196~long-chain-alcohol O-fatty-acyltransferase a	1
GOTERM_MF_ALL	GO:0019139~cytokinin dehydrogenase activity	1
GOTERM_MF_ALL	GO:0016407~acetyltransferase activity	1
GOTERM_MF_ALL	GO:0080103~4-methylthiopropyl glucosinolate S-oxygen	1
GOTERM_MF_ALL	GO:0003743~translation initiation factor activity	1
GOTERM_MF_ALL	GO:0034387~4-aminobutyrate:pyruvate transaminase acti	1
GOTERM_MF_ALL	GO:0016289~CoA hydrolase activity	1
GOTERM_MF_ALL	GO:0016661~oxidoreductase activity, acting on other nitr	1
GOTERM_MF_ALL	GO:0008395~steroid hydroxylase activity	1
GOTERM_MF_ALL	GO:0003840~gamma-glutamyltransferase activity	1
GOTERM_MF_ALL	GO:0009674~potassium:sodium symporter activity	1
GOTERM_MF_ALL	GO:0005254~chloride channel activity	1
GOTERM_MF_ALL	GO:0009917~sterol 5-alpha reductase activity	1
GOTERM_MF_ALL	GO:0005343~organic acid:sodium symporter activity	1
GOTERM_MF_ALL	GO:0004126~cytidine deaminase activity	1
GOTERM_MF_ALL	GO:0046592~polyamine oxidase activity	1
GOTERM_MF_ALL	GO:0042389~omega-3 fatty acid desaturase activity	1
GOTERM_MF_ALL	GO:0051747~DNA demethylase activity	1
GOTERM_MF_ALL	GO:0004559~alpha-mannosidase activity	1
GOTERM_MF_ALL	GO:0022833~mechanically gated channel activity	1
GOTERM_MF_ALL	GO:0016308~1-phosphatidylinositol-4-phosphate 5-kinas	1
GOTERM_MF_ALL	GO:0019207~kinase regulator activity	1
GOTERM_MF_ALL	GO:0008430~selenium binding	1
GOTERM_MF_ALL	GO:0050513~glycoprotein 2-beta-D-xylosyltransferase ac	1
GOTERM_MF_ALL	GO:0031267~small GTPase binding	1
GOTERM_MF_ALL	GO:0046870~cadmium ion binding	1
GOTERM_MF_ALL	GO:0004560~alpha-L-fucosidase activity	1
GOTERM_MF_ALL	GO:0005504~fatty acid binding	1
GOTERM_MF_ALL	GO:0016531~copper chaperone activity	1
GOTERM_MF_ALL	GO:0045543~gibberellin 2-beta-dioxygenase activity	1
GOTERM_MF_ALL	GO:0008878~glucose-1-phosphate adenylyltransferase ac	1
GOTERM_MF_ALL	GO:0004338~glucan 1,3-beta-glucosidase activity	1
GOTERM_MF_ALL	GO:0046423~allene-oxide cyclase activity	1
GOTERM_MF_ALL	GO:0010348~lithium:hydrogen antiporter activity	1

GOTERM_MF_ALL	GO:0080118~brassinosteroid sulfotransferase activity	1
GOTERM_MF_ALL	GO:0033765~steroid dehydrogenase activity, acting on th	1
GOTERM_MF_ALL	GO:0000224~peptide-N4-(N-acetyl-beta-glucosaminyl)as	1
GOTERM_MF_ALL	GO:0019829~cation-transporting ATPase activity	1
GOTERM_MF_ALL	GO:0009884~cytokinin receptor activity	1
GOTERM_MF_ALL	GO:0046933~hydrogen ion transporting ATP synthase act	1
GOTERM_MF_ALL	GO:0047513~1,2-alpha-L-fucosidase activity	1
GOTERM_MF_ALL	GO:0080096~phosphatidate-sterol O-acyltransferase activ	1
GOTERM_MF_ALL	GO:0070003~threonine-type peptidase activity	1
GOTERM_MF_ALL	GO:0008199~ferric iron binding	1
GOTERM_MF_ALL	GO:0003810~protein-glutamine gamma-glutamyltransfera	1
GOTERM_MF_ALL	GO:0070566~adenylyltransferase activity	1
GOTERM_MF_ALL	GO:0016532~superoxide dismutase copper chaperone act	1
GOTERM_MF_ALL	GO:0005527~macrolide binding	1
GOTERM_MF_ALL	GO:0015101~organic cation transmembrane transporter a	1
GOTERM_MF_ALL	GO:0004069~L-aspartate:2-oxoglutarate aminotransferase	1
GOTERM_MF_ALL	GO:0010329~auxin efflux transmembrane transporter acti	1
GOTERM_MF_ALL	GO:0010298~dihydrocamalexic acid decarboxylase activi	1
GOTERM_MF_ALL	GO:0005086~ARF guanyl-nucleotide exchange factor act	1
GOTERM_MF_ALL	GO:0016622~oxidoreductase activity, acting on the aldeh	1
GOTERM_MF_ALL	GO:0019899~enzyme binding	1
GOTERM_MF_ALL	GO:0042277~peptide binding	1
GOTERM_MF_ALL	GO:0008144~drug binding	1
GOTERM_MF_ALL	GO:0004441~inositol-1,4-bisphosphate 1-phosphatase act	1
GOTERM_MF_ALL	GO:0008696~4-amino-4-deoxychorismate lyase activity	1
GOTERM_MF_ALL	GO:0004693~cyclin-dependent protein kinase activity	1
GOTERM_MF_ALL	GO:0035091~phosphoinositide binding	1
GOTERM_MF_ALL	GO:0050421~nitrite reductase (NO-forming) activity	1
GOTERM_MF_ALL	GO:0016760~cellulose synthase (UDP-forming) activity	1
GOTERM_MF_ALL	GO:0015086~cadmium ion transmembrane transporter act	1
GOTERM_MF_ALL	GO:0080107~8-methylthiopropyl glucosinolate S-oxygen	1
GOTERM_MF_ALL	GO:0019156~isoamylase activity	1
GOTERM_MF_ALL	GO:0051183~vitamin transporter activity	1
GOTERM_MF_ALL	GO:0016899~oxidoreductase activity, acting on the CH-O	1
GOTERM_MF_ALL	GO:0008553~hydrogen-exporting ATPase activity, phosp	1
GOTERM_MF_ALL	GO:0008142~oxysterol binding	1
GOTERM_MF_ALL	GO:0004784~superoxide dismutase activity	1
GOTERM_MF_ALL	GO:0004811~tRNA isopentenyltransferase activity	1
GOTERM_MF_ALL	GO:0004594~pantothenate kinase activity	1
GOTERM_MF_ALL	GO:0015925~galactosidase activity	1
GOTERM_MF_ALL	GO:0008551~cadmium-exporting ATPase activity	1
GOTERM_MF_ALL	GO:0015079~potassium ion transmembrane transporter ac	1
GOTERM_MF_ALL	GO:0008135~translation factor activity, nucleic acid bind	1
GOTERM_MF_ALL	GO:0042162~telomeric DNA binding	1
GOTERM_MF_ALL	GO:0080102~3-methylthiopropyl glucosinolate S-oxygen	1
GOTERM_MF_ALL	GO:0016647~oxidoreductase activity, acting on the CH-N	1
GOTERM_MF_ALL	GO:0080106~7-methylthiopropyl glucosinolate S-oxygen	1

GOTERM_MF_ALL	GO:0042887~amide transporter activity	1
GOTERM_MF_ALL	GO:0003973~(S)-2-hydroxy-acid oxidase activity	1
GOTERM_MF_ALL	GO:0015125~bile acid transmembrane transporter activity	1
GOTERM_MF_ALL	GO:0019202~amino acid kinase activity	1
GOTERM_MF_ALL	GO:0004566~beta-glucuronidase activity	1
GOTERM_MF_ALL	GO:0030291~protein serine/threonine kinase inhibitor act	1
GOTERM_MF_ALL	GO:0033971~hydroxyisourate hydrolase activity	1
GOTERM_MF_ALL	GO:0003906~DNA-(apurinic or apyrimidinic site) lyase a	1
GOTERM_MF_ALL	GO:0008441~3'(2'),5'-bisphosphate nucleotidase activity	1
GOTERM_MF_ALL	GO:0016664~oxidoreductase activity, acting on other nitr	1
GOTERM_MF_ALL	GO:0051020~GTPase binding	1
GOTERM_MF_ALL	GO:0048307~ferredoxin-nitrite reductase activity	1
GOTERM_MF_ALL	GO:0016860~intramolecular oxidoreductase activity	1
GOTERM_MF_ALL	GO:0016312~inositol bisphosphate phosphatase activity	1
GOTERM_MF_ALL	GO:0000254~C-4 methylsterol oxidase activity	1
GOTERM_MF_ALL	GO:0005471~ATP:ADP antiporter activity	1
GOTERM_MF_ALL	GO:0004371~glycerone kinase activity	1
GOTERM_MF_ALL	GO:0005338~nucleotide-sugar transmembrane transporter	1
GOTERM_MF_ALL	GO:0080002~UDP-glucose:4-aminobenzoate acylglucosy	1
GOTERM_MF_ALL	GO:0015154~disaccharide transmembrane transporter act	1
GOTERM_MF_ALL	GO:0015651~quaternary ammonium group transmembran	1
GOTERM_MF_ALL	GO:0080065~4-alpha-methyl-delta7-sterol-4alpha-methyl	1
GOTERM_MF_ALL	GO:0005528~FK506 binding	1
GOTERM_MF_ALL	GO:0004676~3-phosphoinositide-dependent protein kinas	1
GOTERM_MF_ALL	GO:0015020~glucuronosyltransferase activity	1
GOTERM_MF_ALL	GO:0015434~cadmium-transporting ATPase activity	1
GOTERM_MF_ALL	GO:0033293~monocarboxylic acid binding	1
GOTERM_MF_ALL	GO:0008773~[protein-PII] uridylyltransferase activity	1
GOTERM_MF_ALL	GO:0003715~transcription termination factor activity	1
GOTERM_MF_ALL	GO:0004351~glutamate decarboxylase activity	1
GOTERM_MF_ALL	GO:0004366~glycerol-3-phosphate O-acyltransferase acti	1
GOTERM_MF_ALL	GO:0019200~carbohydrate kinase activity	1
GOTERM_MF_ALL	GO:0008373~sialyltransferase activity	1
GOTERM_MF_ALL	GO:0016752~sinapoyltransferase activity	1
GOTERM_MF_ALL	GO:0008028~monocarboxylic acid transmembrane transp	1
GOTERM_MF_ALL	GO:0003735~structural constituent of ribosome	1
GOTERM_MF_ALL	GO:0046961~proton-transporting ATPase activity, rotatio	1
GOTERM_MF_ALL	GO:0003867~4-aminobutyrate transaminase activity	1
GOTERM_MF_ALL	GO:0004222~metalloendopeptidase activity	1
GOTERM_MF_ALL	GO:0005085~guanyl-nucleotide exchange factor activity	1
GOTERM_MF_ALL	GO:0009881~photoreceptor activity	1
GOTERM_MF_ALL	GO:0045549~9-cis-epoxycarotenoid dioxygenase activity	1
GOTERM_MF_ALL	GO:0016303~1-phosphatidylinositol-3-kinase activity	1
GOTERM_MF_ALL	GO:0004708~MAP kinase kinase activity	1
GOTERM_MF_ALL	GO:0001653~peptide receptor activity	1
GOTERM_MF_ALL	GO:0004869~cysteine-type endopeptidase inhibitor activi	1
GOTERM_MF_ALL	GO:0004540~ribonuclease activity	1

GOTERM_MF_ALL	GO:0004324~ferredoxin-NADP+ reductase activity	1
GOTERM_MF_ALL	GO:0051015~actin filament binding	1
GOTERM_MF_ALL	GO:0016721~oxidoreductase activity, acting on superoxid	1
GOTERM_MF_ALL	GO:0008536~Ran GTPase binding	1
GOTERM_MF_ALL	GO:0009882~blue light photoreceptor activity	1
GOTERM_MF_ALL	GO:0047202~sinapoylglucose-choline O-sinapoyltransfer	1
GOTERM_MF_ALL	GO:0080087~callose binding	1
GOTERM_MF_ALL	GO:0015204~urea transmembrane transporter activity	1
GOTERM_MF_ALL	GO:0051669~fructan beta-fructosidase activity	1
GOTERM_MF_ALL	GO:0003777~microtubule motor activity	1
GOTERM_MF_ALL	GO:0015174~basic amino acid transmembrane transporte	1
GOTERM_MF_ALL	GO:0019887~protein kinase regulator activity	1
GOTERM_MF_ALL	GO:0008252~nucleotidase activity	1
GOTERM_MF_ALL	GO:0080046~quercetin 4'-O-glucosyltransferase activity	1
GOTERM_MF_ALL	GO:0004181~metallocarboxypeptidase activity	1
GOTERM_MF_ALL	GO:0004521~endoribonuclease activity	1
GOTERM_MF_ALL	GO:0016720~delta12-fatty acid dehydrogenase activity	1
GOTERM_MF_ALL	GO:0016707~gibberellin 3-beta-dioxygenase activity	1
GOTERM_MF_ALL	GO:0004861~cyclin-dependent protein kinase inhibitor ac	1
GOTERM_MF_ALL	GO:0009824~adenylate dimethylallyltransferase activity	1
GOTERM_MF_ALL	GO:0047631~ADP-ribose diphosphatase activity	1
GOTERM_MF_ALL	GO:0010279~indole-3-acetic acid amido synthetase activi	1
GOTERM_MF_ALL	GO:0070569~uridylyltransferase activity	1
GOTERM_MF_ALL	GO:0016730~oxidoreductase activity, acting on iron-sulfu	1
GOTERM_MF_ALL	GO:0008138~protein tyrosine/serine/threonine phosphata	1
GOTERM_MF_ALL	GO:0016530~metallochaperone activity	1
GOTERM_MF_ALL	GO:0080095~phosphatidylethanolamine-sterol O-acyltran	1
GOTERM_MF_ALL	GO:0016894~endonuclease activity, active with either rib	1
GOTERM_MF_ALL	GO:0008408~3'-5' exonuclease activity	1
GOTERM_MF_ALL	GO:0042299~lupeol synthase activity	1
GOTERM_MF_ALL	GO:0043168~anion binding	1
GOTERM_MF_ALL	GO:0008301~DNA bending activity	1
GOTERM_MF_ALL	GO:0051287~NAD or NADH binding	1
GOTERM_MF_ALL	GO:0016463~zinc-exporting ATPase activity	1
GOTERM_MF_ALL	GO:0032934~sterol binding	1
GOTERM_MF_ALL	GO:0016833~oxo-acid-lyase activity	1
GOTERM_MF_ALL	GO:0004965~GABA-B receptor activity	1
GOTERM_MF_ALL	GO:0003863~3-methyl-2-oxobutanoate dehydrogenase (2	1
GOTERM_MF_ALL	GO:0008429~phosphatidylethanolamine binding	1
GOTERM_MF_ALL	GO:0015085~calcium ion transmembrane transporter acti	1
GOTERM_MF_ALL	GO:0015226~carnitine transporter activity	1
GOTERM_MF_ALL	GO:0004851~uroporphyrin-III C-methyltransferase activit	1
GOTERM_MF_ALL	GO:0016812~hydrolase activity, acting on carbon-nitroge	1
GOTERM_MF_ALL	GO:0051139~metal ion:hydrogen antiporter activity	1
GOTERM_MF_ALL	GO:0016731~oxidoreductase activity, acting on iron-sulfu	1
GOTERM_MF_ALL	GO:0045485~omega-6 fatty acid desaturase activity	1
GOTERM_MF_ALL	GO:0016814~hydrolase activity, acting on carbon-nitroge	1

GOTERM_MF_ALL	GO:0016662~oxidoreductase activity, acting on other nitr	1
GOTERM_MF_ALL	GO:0004109~coproporphyrinogen oxidase activity	1
GOTERM_MF_ALL	GO:0004072~aspartate kinase activity	1
GOTERM_MF_ALL	GO:0033558~protein deacetylase activity	1
GOTERM_MF_ALL	GO:0016410~N-acyltransferase activity	1
GOTERM_MF_ALL	GO:0031404~chloride ion binding	1
GOTERM_MF_ALL	GO:0008327~methyl-CpG binding	1
GOTERM_MF_ALL	GO:0008375~acetylglucosaminyltransferase activity	1
GOTERM_MF_ALL	GO:0004565~beta-galactosidase activity	1
GOTERM_MF_ALL	GO:0016892~endoribonuclease activity, producing 3'-pho	1
GOTERM_MF_ALL	GO:0004407~histone deacetylase activity	1
GOTERM_MF_ALL	GO:0042300~beta-amyrin synthase activity	1
GOTERM_MF_ALL	GO:0008422~beta-glucosidase activity	1
GOTERM_CC_ALL	GO:0005622~intracellular	442
GOTERM_CC_ALL	GO:0016591~DNA-directed RNA polymerase II, holoenz	1
GOTERM_CC_ALL	GO:0022626~cytosolic ribosome	1
GOTERM_CC_ALL	GO:0000502~proteasome complex	1
GOTERM_CC_ALL	GO:0031969~chloroplast membrane	1
GOTERM_CC_ALL	GO:0033180~proton-transporting V-type ATPase, V1 do	1
GOTERM_CC_ALL	GO:0031301~integral to organelle membrane	1
GOTERM_CC_ALL	GO:0044445~cytosolic part	1
GOTERM_CC_ALL	GO:0030126~COPI vesicle coat	1
GOTERM_CC_ALL	GO:0005578~proteinaceous extracellular matrix	1
GOTERM_CC_ALL	GO:0016328~lateral plasma membrane	1
GOTERM_CC_ALL	GO:0005839~proteasome core complex	1
GOTERM_CC_ALL	GO:0009986~cell surface	1
GOTERM_CC_ALL	GO:0005672~transcription factor TFIIA complex	1
GOTERM_CC_ALL	GO:0000323~lytic vacuole	1
GOTERM_CC_ALL	GO:0000932~cytoplasmic mRNA processing body	1
GOTERM_CC_ALL	GO:0016607~nuclear speck	1
GOTERM_CC_ALL	GO:0048475~coated membrane	1
GOTERM_CC_ALL	GO:0015629~actin cytoskeleton	1
GOTERM_CC_ALL	GO:0016469~proton-transporting two-sector ATPase com	1
GOTERM_CC_ALL	GO:0030120~vesicle coat	1
GOTERM_CC_ALL	GO:0030133~transport vesicle	1
GOTERM_CC_ALL	GO:0005802~trans-Golgi network	1
GOTERM_CC_ALL	GO:0031209~SCAR complex	1
GOTERM_CC_ALL	GO:0019897~extrinsic to plasma membrane	1
GOTERM_CC_ALL	GO:0030136~clathrin-coated vesicle	1
GOTERM_CC_ALL	GO:0009524~phragmoplast	1
GOTERM_CC_ALL	GO:0031228~intrinsic to Golgi membrane	1
GOTERM_CC_ALL	GO:0009341~beta-galactosidase complex	1
GOTERM_CC_ALL	GO:0008076~voltage-gated potassium channel complex	1
GOTERM_CC_ALL	GO:0031304~intrinsic to mitochondrial inner membrane	1
GOTERM_CC_ALL	GO:0005742~mitochondrial outer membrane translocase	1
GOTERM_CC_ALL	GO:0030662~coated vesicle membrane	1
GOTERM_CC_ALL	GO:0030288~outer membrane-bounded periplasmic space	1

GOTERM_CC_ALL	GO:0033176~proton-transporting V-type ATPase comple	1
GOTERM_CC_ALL	GO:0031234~extrinsic to internal side of plasma membra	1
GOTERM_CC_ALL	GO:0042597~periplasmic space	1
GOTERM_CC_ALL	GO:0030137~COPI-coated vesicle	1
GOTERM_CC_ALL	GO:0034703~cation channel complex	1
GOTERM_CC_ALL	GO:0034705~potassium channel complex	1
GOTERM_CC_ALL	GO:0030313~cell envelope	1
GOTERM_CC_ALL	GO:0030173~integral to Golgi membrane	1
GOTERM_CC_ALL	GO:0009527~plastid outer membrane	1
GOTERM_CC_ALL	GO:0033178~proton-transporting two-sector ATPase com	1
GOTERM_CC_ALL	GO:0048471~perinuclear region of cytoplasm	1
GOTERM_CC_ALL	GO:0010170~glucose-1-phosphate adenylyltransferase co	1
GOTERM_CC_ALL	GO:0016604~nuclear body	1
GOTERM_CC_ALL	GO:0009514~glyoxysome	1
GOTERM_CC_ALL	GO:0044422~organelle part	57
GOTERM_CC_ALL	GO:0005635~nuclear envelope	1
GOTERM_CC_ALL	GO:0044462~external encapsulating structure part	1
GOTERM_CC_ALL	GO:0030663~COPI coated vesicle membrane	1
GOTERM_CC_ALL	GO:0009706~chloroplast inner membrane	1
GOTERM_CC_ALL	GO:0030659~cytoplasmic vesicle membrane	1
GOTERM_CC_ALL	GO:0044433~cytoplasmic vesicle part	1
GOTERM_CC_ALL	GO:0030140~trans-Golgi network transport vesicle	1
GOTERM_CC_ALL	GO:0030660~Golgi-associated vesicle membrane	1
GOTERM_CC_ALL	GO:0030117~membrane coat	1
GOTERM_CC_ALL	GO:0016459~myosin complex	1
GOTERM_CC_ALL	GO:0031985~Golgi cisterna	1
GOTERM_CC_ALL	GO:0034707~chloride channel complex	1
GOTERM_CC_ALL	GO:0005764~lysosome	1
GOTERM_CC_ALL	GO:0010369~chromocenter	1
GOTERM_CC_ALL	GO:0005942~phosphoinositide 3-kinase complex	1
GOTERM_CC_ALL	GO:0044424~intracellular part	414
GOTERM_CC_ALL	GO:0044446~intracellular organelle part	56
GOTERM_CC_ALL	GO:0009530~primary cell wall	1
GOTERM_CC_ALL	GO:0045298~tubulin complex	1
GOTERM_CC_ALL	GO:0043224~nuclear SCF ubiquitin ligase complex	1
GOTERM_CC_ALL	GO:0005797~Golgi medial cisterna	1
GOTERM_BP_ALL	GO:0046128~purine ribonucleoside metabolic process	1
GOTERM_BP_ALL	GO:0042752~regulation of circadian rhythm	1
GOTERM_BP_ALL	GO:0032413~negative regulation of ion transmembrane tr	1
GOTERM_BP_ALL	GO:0031936~negative regulation of chromatin silencing	1
GOTERM_BP_ALL	GO:0010233~phloem transport	1
GOTERM_BP_ALL	GO:0022613~ribonucleoprotein complex biogenesis	1
GOTERM_BP_ALL	GO:0009161~ribonucleoside monophosphate metabolic p	1
GOTERM_BP_ALL	GO:0008655~pyrimidine salvage	1
GOTERM_BP_ALL	GO:0009644~response to high light intensity	1
GOTERM_BP_ALL	GO:0006890~retrograde vesicle-mediated transport, Golg	1
GOTERM_BP_ALL	GO:0006862~nucleotide transport	1

GOTERM_BP_ALL	GO:0006821~chloride transport	1
GOTERM_BP_ALL	GO:0009127~purine nucleoside monophosphate biosynth	1
GOTERM_BP_ALL	GO:0000741~karyogamy	1
GOTERM_BP_ALL	GO:0032409~regulation of transporter activity	1
GOTERM_BP_ALL	GO:0044247~cellular polysaccharide catabolic process	1
GOTERM_BP_ALL	GO:0009566~fertilization	1
GOTERM_BP_ALL	GO:0000023~maltose metabolic process	1
GOTERM_BP_ALL	GO:0055046~microgametogenesis	1
GOTERM_BP_ALL	GO:0048193~Golgi vesicle transport	1
GOTERM_BP_ALL	GO:0006884~cell volume homeostasis	1
GOTERM_BP_ALL	GO:0046654~tetrahydrofolate biosynthetic process	1
GOTERM_BP_ALL	GO:0015937~coenzyme A biosynthetic process	1
GOTERM_BP_ALL	GO:0006024~glycosaminoglycan biosynthetic process	1
GOTERM_BP_ALL	GO:0006817~phosphate transport	1
GOTERM_BP_ALL	GO:0045736~negative regulation of cyclin-dependent pro	1
GOTERM_BP_ALL	GO:0009853~photorespiration	1
GOTERM_BP_ALL	GO:0051098~regulation of binding	1
GOTERM_BP_ALL	GO:0009124~nucleoside monophosphate biosynthetic pro	1
GOTERM_BP_ALL	GO:0033674~positive regulation of kinase activity	1
GOTERM_BP_ALL	GO:0009559~embryo sac central cell differentiation	1
GOTERM_BP_ALL	GO:0009594~detection of nutrient	1
GOTERM_BP_ALL	GO:0042254~ribosome biogenesis	1
GOTERM_BP_ALL	GO:0010077~maintenance of inflorescence meristem iden	1
GOTERM_BP_ALL	GO:0009920~cell plate formation involved in plant-type c	1
GOTERM_BP_ALL	GO:0051246~regulation of protein metabolic process	1
GOTERM_BP_ALL	GO:0016110~tetraterpenoid catabolic process	1
GOTERM_BP_ALL	GO:0031032~actomyosin structure organization	1
GOTERM_BP_ALL	GO:0010014~meristem initiation	1
GOTERM_BP_ALL	GO:0006305~DNA alkylation	1
GOTERM_BP_ALL	GO:0016337~cell-cell adhesion	1
GOTERM_BP_ALL	GO:0042814~monopolar cell growth	1
GOTERM_BP_ALL	GO:0006566~threonine metabolic process	1
GOTERM_BP_ALL	GO:0007005~mitochondrion organization	1
GOTERM_BP_ALL	GO:0048508~embryonic meristem development	1
GOTERM_BP_ALL	GO:0010351~lithium ion transport	1
GOTERM_BP_ALL	GO:0006342~chromatin silencing	1
GOTERM_BP_ALL	GO:0031407~oxylipin metabolic process	1
GOTERM_BP_ALL	GO:0043481~anthocyanin accumulation in tissues in resp	1
GOTERM_BP_ALL	GO:0009595~detection of biotic stimulus	1
GOTERM_BP_ALL	GO:0080111~DNA demethylation	1
GOTERM_BP_ALL	GO:0010439~regulation of glucosinolate biosynthetic pro	1
GOTERM_BP_ALL	GO:0010103~stomatal complex morphogenesis	1
GOTERM_BP_ALL	GO:0006023~aminoglycan biosynthetic process	1
GOTERM_BP_ALL	GO:0006261~DNA-dependent DNA replication	1
GOTERM_BP_ALL	GO:0009720~detection of hormone stimulus	1
GOTERM_BP_ALL	GO:0010540~basipetal auxin transport	1
GOTERM_BP_ALL	GO:0000270~peptidoglycan metabolic process	1

GOTERM_BP_ALL	GO:0015979~photosynthesis	1
GOTERM_BP_ALL	GO:0009086~methionine biosynthetic process	1
GOTERM_BP_ALL	GO:0009582~detection of abiotic stimulus	1
GOTERM_BP_ALL	GO:0009065~glutamine family amino acid catabolic proc	1
GOTERM_BP_ALL	GO:0001560~regulation of cell growth by extracellular sti	1
GOTERM_BP_ALL	GO:0046246~terpene biosynthetic process	1
GOTERM_BP_ALL	GO:0048653~anther development	1
GOTERM_BP_ALL	GO:0019509~methionine salvage	1
GOTERM_BP_ALL	GO:0009942~longitudinal axis specification	1
GOTERM_BP_ALL	GO:0042178~xenobiotic catabolic process	1
GOTERM_BP_ALL	GO:0006540~glutamate decarboxylation to succinate	1
GOTERM_BP_ALL	GO:0051762~sesquiterpene biosynthetic process	1
GOTERM_BP_ALL	GO:0046283~anthocyanin metabolic process	1
GOTERM_BP_ALL	GO:0010492~maintenance of shoot apical meristem ident	1
GOTERM_BP_ALL	GO:0010048~vernalization response	1
GOTERM_BP_ALL	GO:0009992~cellular water homeostasis	1
GOTERM_BP_ALL	GO:0006071~glycerol metabolic process	1
GOTERM_BP_ALL	GO:0042401~biogenic amine biosynthetic process	1
GOTERM_BP_ALL	GO:0009126~purine nucleoside monophosphate metaboli	1
GOTERM_BP_ALL	GO:0046473~phosphatidic acid metabolic process	1
GOTERM_BP_ALL	GO:0009904~chloroplast accumulation movement	1
GOTERM_BP_ALL	GO:0033043~regulation of organelle organization	1
GOTERM_BP_ALL	GO:0010360~negative regulation of anion channel activit	1
GOTERM_BP_ALL	GO:0010941~regulation of cell death	1
GOTERM_BP_ALL	GO:0010076~maintenance of floral meristem identity	1
GOTERM_BP_ALL	GO:0009963~positive regulation of flavonoid biosyntheti	1
GOTERM_BP_ALL	GO:0045053~protein retention in Golgi apparatus	1
GOTERM_BP_ALL	GO:0007638~mechanosensory behavior	1
GOTERM_BP_ALL	GO:0006878~cellular copper ion homeostasis	1
GOTERM_BP_ALL	GO:0015802~basic amino acid transport	1
GOTERM_BP_ALL	GO:0000912~formation of actomyosin apparatus involved	1
GOTERM_BP_ALL	GO:0009959~negative gravitropism	1
GOTERM_BP_ALL	GO:0007172~signal complex assembly	1
GOTERM_BP_ALL	GO:0009901~anther dehiscence	1
GOTERM_BP_ALL	GO:0010071~root meristem specification	1
GOTERM_BP_ALL	GO:0016553~base conversion or substitution editing	1
GOTERM_BP_ALL	GO:0046488~phosphatidylinositol metabolic process	1
GOTERM_BP_ALL	GO:0008033~tRNA processing	1
GOTERM_BP_ALL	GO:0016122~xanthophyll metabolic process	1
GOTERM_BP_ALL	GO:0051090~regulation of transcription factor activity	1
GOTERM_BP_ALL	GO:0045990~regulation of transcription by carbon catabo	1
GOTERM_BP_ALL	GO:0031087~deadenylation-independent decapping of nu	1
GOTERM_BP_ALL	GO:0010638~positive regulation of organelle organizatio	1
GOTERM_BP_ALL	GO:0080117~secondary growth	1
GOTERM_BP_ALL	GO:0051169~nuclear transport	1
GOTERM_BP_ALL	GO:0048768~root hair cell tip growth	1
GOTERM_BP_ALL	GO:0031935~regulation of chromatin silencing	1

GOTERM_BP_ALL	GO:0007243~protein kinase cascade	1
GOTERM_BP_ALL	GO:0010082~regulation of root meristem growth	1
GOTERM_BP_ALL	GO:0010249~auxin conjugate metabolic process	1
GOTERM_BP_ALL	GO:0019722~calcium-mediated signaling	1
GOTERM_BP_ALL	GO:0000097~sulfur amino acid biosynthetic process	1
GOTERM_BP_ALL	GO:0010155~regulation of proton transport	1
GOTERM_BP_ALL	GO:0006014~D-ribose metabolic process	1
GOTERM_BP_ALL	GO:0018298~protein-chromophore linkage	1
GOTERM_BP_ALL	GO:0006547~histidine metabolic process	1
GOTERM_BP_ALL	GO:0009902~chloroplast relocation	1
GOTERM_BP_ALL	GO:0043414~biopolymer methylation	1
GOTERM_BP_ALL	GO:0006418~tRNA aminoacylation for protein translatio	1
GOTERM_BP_ALL	GO:0006310~DNA recombination	1
GOTERM_BP_ALL	GO:0010188~response to microbial phytotoxin	1
GOTERM_BP_ALL	GO:0044275~cellular carbohydrate catabolic process	1
GOTERM_BP_ALL	GO:0043066~negative regulation of apoptosis	1
GOTERM_BP_ALL	GO:0009693~ethylene biosynthetic process	1
GOTERM_BP_ALL	GO:0016106~sesquiterpenoid biosynthetic process	1
GOTERM_BP_ALL	GO:0051101~regulation of DNA binding	1
GOTERM_BP_ALL	GO:0034765~regulation of ion transmembrane transport	1
GOTERM_BP_ALL	GO:0032259~methylation	1
GOTERM_BP_ALL	GO:0009063~cellular amino acid catabolic process	1
GOTERM_BP_ALL	GO:0051128~regulation of cellular component organizati	1
GOTERM_BP_ALL	GO:0006412~translation	2
GOTERM_BP_ALL	GO:0043067~regulation of programmed cell death	1
GOTERM_BP_ALL	GO:0007267~cell-cell signaling	1
GOTERM_BP_ALL	GO:0016117~carotenoid biosynthetic process	1
GOTERM_BP_ALL	GO:0030026~cellular manganese ion homeostasis	1
GOTERM_BP_ALL	GO:0006935~chemotaxis	1
GOTERM_BP_ALL	GO:0006367~transcription initiation from RNA polymera	1
GOTERM_BP_ALL	GO:0006085~acetyl-CoA biosynthetic process	1
GOTERM_BP_ALL	GO:0009871~jasmonic acid and ethylene-dependent syste	1
GOTERM_BP_ALL	GO:0046345~abscisic acid catabolic process	1
GOTERM_BP_ALL	GO:0005985~sucrose metabolic process	1
GOTERM_BP_ALL	GO:0006767~water-soluble vitamin metabolic process	1
GOTERM_BP_ALL	GO:0010043~response to zinc ion	1
GOTERM_BP_ALL	GO:0048766~root hair initiation	1
GOTERM_BP_ALL	GO:0048826~cotyledon morphogenesis	1
GOTERM_BP_ALL	GO:0043289~apocarotenoid biosynthetic process	1
GOTERM_BP_ALL	GO:0009641~shade avoidance	1
GOTERM_BP_ALL	GO:0009692~ethylene metabolic process	1
GOTERM_BP_ALL	GO:0015980~energy derivation by oxidation of organic c	1
GOTERM_BP_ALL	GO:0015936~coenzyme A metabolic process	1
GOTERM_BP_ALL	GO:0008653~lipopolysaccharide metabolic process	1
GOTERM_BP_ALL	GO:0007626~locomotory behavior	1
GOTERM_BP_ALL	GO:0010050~vegetative phase change	1
GOTERM_BP_ALL	GO:0048284~organelle fusion	1

GOTERM_BP_ALL	GO:0016118~carotenoid catabolic process	1
GOTERM_BP_ALL	GO:0007008~outer mitochondrial membrane organization	1
GOTERM_BP_ALL	GO:0042278~purine nucleoside metabolic process	1
GOTERM_BP_ALL	GO:0015711~organic anion transport	1
GOTERM_BP_ALL	GO:0032412~regulation of ion transmembrane transporter	1
GOTERM_BP_ALL	GO:0045814~negative regulation of gene expression, epig	1
GOTERM_BP_ALL	GO:0048573~photoperiodism, flowering	1
GOTERM_BP_ALL	GO:0034220~ion transmembrane transport	1
GOTERM_BP_ALL	GO:0000914~phragmoplast formation	1
GOTERM_BP_ALL	GO:0031670~cellular response to nutrient	1
GOTERM_BP_ALL	GO:0035196~gene silencing by miRNA, production of mi	1
GOTERM_BP_ALL	GO:0009785~blue light signaling pathway	1
GOTERM_BP_ALL	GO:0070585~protein localization in mitochondrion	1
GOTERM_BP_ALL	GO:0010565~regulation of cellular ketone metabolic proc	1
GOTERM_BP_ALL	GO:0043449~cellular alkene metabolic process	1
GOTERM_BP_ALL	GO:0031204~posttranslational protein targeting to membr	1
GOTERM_BP_ALL	GO:0048831~regulation of shoot development	1
GOTERM_BP_ALL	GO:0048479~style development	1
GOTERM_BP_ALL	GO:0032012~regulation of ARF protein signal transductio	1
GOTERM_BP_ALL	GO:0016554~cytidine to uridine editing	1
GOTERM_BP_ALL	GO:0010371~regulation of gibberellin biosynthetic proce	1
GOTERM_BP_ALL	GO:0006916~anti-apoptosis	1
GOTERM_BP_ALL	GO:0006766~vitamin metabolic process	1
GOTERM_BP_ALL	GO:0046890~regulation of lipid biosynthetic process	1
GOTERM_BP_ALL	GO:0042330~taxis	1
GOTERM_BP_ALL	GO:0042023~DNA endoreduplication	1
GOTERM_BP_ALL	GO:0000280~nuclear division	1
GOTERM_BP_ALL	GO:0046655~folic acid metabolic process	1
GOTERM_BP_ALL	GO:0007067~mitosis	1
GOTERM_BP_ALL	GO:0051238~sequestering of metal ion	1
GOTERM_BP_ALL	GO:0010497~plasmodesmata-mediated intercellular trans	1
GOTERM_BP_ALL	GO:0000375~RNA splicing, via transesterification reactio	1
GOTERM_BP_ALL	GO:0000105~histidine biosynthetic process	1
GOTERM_BP_ALL	GO:0006880~intracellular sequestering of iron ion	1
GOTERM_BP_ALL	GO:0000278~mitotic cell cycle	1
GOTERM_BP_ALL	GO:0032956~regulation of actin cytoskeleton organizatio	1
GOTERM_BP_ALL	GO:0031408~oxylipin biosynthetic process	1
GOTERM_BP_ALL	GO:0019745~pentacyclic triterpenoid biosynthetic proces	1
GOTERM_BP_ALL	GO:0006914~autophagy	1
GOTERM_BP_ALL	GO:0032583~regulation of gene-specific transcription	1
GOTERM_BP_ALL	GO:0048572~short-day photoperiodism	1
GOTERM_BP_ALL	GO:0009958~positive gravitropism	1
GOTERM_BP_ALL	GO:0019484~beta-alanine catabolic process	1
GOTERM_BP_ALL	GO:0043290~apocarotenoid catabolic process	1
GOTERM_BP_ALL	GO:0009116~nucleoside metabolic process	1
GOTERM_BP_ALL	GO:0051553~flavone biosynthetic process	1
GOTERM_BP_ALL	GO:0005977~glycogen metabolic process	1

GOTERM_BP_ALL	GO:0009657~plastid organization	1
GOTERM_BP_ALL	GO:0051254~positive regulation of RNA metabolic proce	1
GOTERM_BP_ALL	GO:0010305~leaf vascular tissue pattern formation	1
GOTERM_BP_ALL	GO:0006665~sphingolipid metabolic process	1
GOTERM_BP_ALL	GO:0009252~peptidoglycan biosynthetic process	1
GOTERM_BP_ALL	GO:0010496~intercellular transport	1
GOTERM_BP_ALL	GO:0009691~cytokinin biosynthetic process	1
GOTERM_BP_ALL	GO:0006112~energy reserve metabolic process	1
GOTERM_BP_ALL	GO:0051656~establishment of organelle localization	1
GOTERM_BP_ALL	GO:0010324~membrane invagination	1
GOTERM_BP_ALL	GO:0010286~heat acclimation	1
GOTERM_BP_ALL	GO:0030203~glycosaminoglycan metabolic process	1
GOTERM_BP_ALL	GO:0045014~negative regulation of transcription by gluc	1
GOTERM_BP_ALL	GO:0005978~glycogen biosynthetic process	1
GOTERM_BP_ALL	GO:0043269~regulation of ion transport	1
GOTERM_BP_ALL	GO:0009396~folic acid and derivative biosynthetic proces	1
GOTERM_BP_ALL	GO:0010431~seed maturation	1
GOTERM_BP_ALL	GO:0016046~detection of fungus	1
GOTERM_BP_ALL	GO:0051130~positive regulation of cellular component or	1
GOTERM_BP_ALL	GO:0009658~chloroplast organization	1
GOTERM_BP_ALL	GO:0030705~cytoskeleton-dependent intracellular transp	1
GOTERM_BP_ALL	GO:0007264~small GTPase mediated signal transduction	1
GOTERM_BP_ALL	GO:0022611~dormancy process	1
GOTERM_BP_ALL	GO:0034434~sterol esterification	1
GOTERM_BP_ALL	GO:0006825~copper ion transport	1
GOTERM_BP_ALL	GO:0051348~negative regulation of transferase activity	1
GOTERM_BP_ALL	GO:0042168~heme metabolic process	1
GOTERM_BP_ALL	GO:0001558~regulation of cell growth	1
GOTERM_BP_ALL	GO:0009695~jasmonic acid biosynthetic process	1
GOTERM_BP_ALL	GO:0009648~photoperiodism	1
GOTERM_BP_ALL	GO:0010215~cellulose microfibril organization	1
GOTERM_BP_ALL	GO:0055062~phosphate ion homeostasis	1
GOTERM_BP_ALL	GO:0060548~negative regulation of cell death	1
GOTERM_BP_ALL	GO:0055061~di-, tri-valent inorganic anion homeostasis	1
GOTERM_BP_ALL	GO:0051125~regulation of actin nucleation	1
GOTERM_BP_ALL	GO:0009269~response to desiccation	1
GOTERM_BP_ALL	GO:0009650~UV protection	1
GOTERM_BP_ALL	GO:0006469~negative regulation of protein kinase activit	1
GOTERM_BP_ALL	GO:0043562~cellular response to nitrogen levels	1
GOTERM_BP_ALL	GO:0006366~transcription from RNA polymerase II prom	1
GOTERM_BP_ALL	GO:0009738~abscisic acid mediated signaling	1
GOTERM_BP_ALL	GO:0019747~regulation of isoprenoid metabolic process	1
GOTERM_BP_ALL	GO:0051321~meiotic cell cycle	1
GOTERM_BP_ALL	GO:0042554~superoxide anion generation	1
GOTERM_BP_ALL	GO:0042447~hormone catabolic process	1
GOTERM_BP_ALL	GO:0006882~cellular zinc ion homeostasis	1
GOTERM_BP_ALL	GO:0016126~sterol biosynthetic process	1

GOTERM_BP_ALL	GO:0016121~carotene catabolic process	1
GOTERM_BP_ALL	GO:0080022~primary root development	1
GOTERM_BP_ALL	GO:0019400~alditol metabolic process	1
GOTERM_BP_ALL	GO:0015986~ATP synthesis coupled proton transport	1
GOTERM_BP_ALL	GO:0009865~pollen tube adhesion	1
GOTERM_BP_ALL	GO:0009123~nucleoside monophosphate metabolic proce	1
GOTERM_BP_ALL	GO:0034067~protein localization in Golgi apparatus	1
GOTERM_BP_ALL	GO:0045962~positive regulation of development, heteroc	1
GOTERM_BP_ALL	GO:0051763~sesquiterpene catabolic process	1
GOTERM_BP_ALL	GO:0009694~jasmonic acid metabolic process	1
GOTERM_BP_ALL	GO:0034470~ncRNA processing	1
GOTERM_BP_ALL	GO:0040011~locomotion	1
GOTERM_BP_ALL	GO:0015780~nucleotide-sugar transport	1
GOTERM_BP_ALL	GO:0046622~positive regulation of organ growth	1
GOTERM_BP_ALL	GO:0010047~fruit dehiscence	1
GOTERM_BP_ALL	GO:0006997~nucleus organization	1
GOTERM_BP_ALL	GO:0044093~positive regulation of molecular function	1
GOTERM_BP_ALL	GO:0009168~purine ribonucleoside monophosphate biosy	1
GOTERM_BP_ALL	GO:0033673~negative regulation of kinase activity	1
GOTERM_BP_ALL	GO:0009688~abscisic acid biosynthetic process	1
GOTERM_BP_ALL	GO:0048236~plant-type spore development	1
GOTERM_BP_ALL	GO:0022898~regulation of transmembrane transporter act	1
GOTERM_BP_ALL	GO:0051193~regulation of cofactor metabolic process	1
GOTERM_BP_ALL	GO:0051644~plastid localization	1
GOTERM_BP_ALL	GO:0015746~citrate transport	1
GOTERM_BP_ALL	GO:0046493~lipid A metabolic process	1
GOTERM_BP_ALL	GO:0016119~carotene metabolic process	1
GOTERM_BP_ALL	GO:0006722~triterpenoid metabolic process	1
GOTERM_BP_ALL	GO:0010158~abaxial cell fate specification	1
GOTERM_BP_ALL	GO:0015692~lead ion transport	1
GOTERM_BP_ALL	GO:0006598~polyamine catabolic process	1
GOTERM_BP_ALL	GO:0030048~actin filament-based movement	1
GOTERM_BP_ALL	GO:0045815~positive regulation of gene expression, epig	1
GOTERM_BP_ALL	GO:0006105~succinate metabolic process	1
GOTERM_BP_ALL	GO:0042537~benzene and derivative metabolic process	1
GOTERM_BP_ALL	GO:0046482~para-aminobenzoic acid metabolic process	1
GOTERM_BP_ALL	GO:0019742~pentacyclic triterpenoid metabolic process	1
GOTERM_BP_ALL	GO:0010183~pollen tube guidance	1
GOTERM_BP_ALL	GO:0050982~detection of mechanical stimulus	1
GOTERM_BP_ALL	GO:0002831~regulation of response to biotic stimulus	1
GOTERM_BP_ALL	GO:0046156~siroheme metabolic process	1
GOTERM_BP_ALL	GO:0042762~regulation of sulfur metabolic process	1
GOTERM_BP_ALL	GO:0006995~cellular response to nitrogen starvation	1
GOTERM_BP_ALL	GO:0046467~membrane lipid biosynthetic process	1
GOTERM_BP_ALL	GO:0051555~flavonol biosynthetic process	1
GOTERM_BP_ALL	GO:0046620~regulation of organ growth	1
GOTERM_BP_ALL	GO:0009247~glycolipid biosynthetic process	1

GOTERM_BP_ALL	GO:0000165~MAPKKK cascade	1
GOTERM_BP_ALL	GO:0009823~cytokinin catabolic process	1
GOTERM_BP_ALL	GO:0051049~regulation of transport	1
GOTERM_BP_ALL	GO:0033865~nucleoside bisphosphate metabolic process	1
GOTERM_BP_ALL	GO:0042981~regulation of apoptosis	1
GOTERM_BP_ALL	GO:0009729~detection of brassinosteroid stimulus	1
GOTERM_BP_ALL	GO:0006664~glycolipid metabolic process	1
GOTERM_BP_ALL	GO:0045893~positive regulation of transcription, DNA-d	1
GOTERM_BP_ALL	GO:0006783~heme biosynthetic process	1
GOTERM_BP_ALL	GO:0043255~regulation of carbohydrate biosynthetic pro	1
GOTERM_BP_ALL	GO:0043085~positive regulation of catalytic activity	1
GOTERM_BP_ALL	GO:0051667~establishment of plastid localization	1
GOTERM_BP_ALL	GO:0009273~peptidoglycan-based cell wall biogenesis	1
GOTERM_BP_ALL	GO:0015985~energy coupled proton transport, down elec	1
GOTERM_BP_ALL	GO:0010162~seed dormancy	1
GOTERM_BP_ALL	GO:0046113~nucleobase catabolic process	1
GOTERM_BP_ALL	GO:0009294~DNA mediated transformation	1
GOTERM_BP_ALL	GO:0010306~rhamnogalacturonan II biosynthetic process	1
GOTERM_BP_ALL	GO:0030104~water homeostasis	1
GOTERM_BP_ALL	GO:0018874~benzoate metabolic process	1
GOTERM_BP_ALL	GO:0009808~lignin metabolic process	1
GOTERM_BP_ALL	GO:0043900~regulation of multi-organism process	1
GOTERM_BP_ALL	GO:0051552~flavone metabolic process	1
GOTERM_BP_ALL	GO:0010398~xylogalacturonan metabolic process	1
GOTERM_BP_ALL	GO:0043102~amino acid salvage	1
GOTERM_BP_ALL	GO:0048838~release of seed from dormancy	1
GOTERM_BP_ALL	GO:0016036~cellular response to phosphate starvation	1
GOTERM_BP_ALL	GO:0051554~flavonol metabolic process	1
GOTERM_BP_ALL	GO:0048645~organ formation	1
GOTERM_BP_ALL	GO:0045860~positive regulation of protein kinase activity	1
GOTERM_BP_ALL	GO:0001522~pseudouridine synthesis	1
GOTERM_BP_ALL	GO:0010374~stomatal complex development	1
GOTERM_BP_ALL	GO:0016598~protein arginylation	1
GOTERM_BP_ALL	GO:0006304~DNA modification	1
GOTERM_BP_ALL	GO:0045927~positive regulation of growth	1
GOTERM_BP_ALL	GO:0019482~beta-alanine metabolic process	1
GOTERM_BP_ALL	GO:0032582~negative regulation of gene-specific transcri	1
GOTERM_BP_ALL	GO:0009746~response to hexose stimulus	1
GOTERM_BP_ALL	GO:0006084~acetyl-CoA metabolic process	1
GOTERM_BP_ALL	GO:0046685~response to arsenic	1
GOTERM_BP_ALL	GO:0010359~regulation of anion channel activity	1
GOTERM_BP_ALL	GO:0009450~gamma-aminobutyric acid catabolic process	1
GOTERM_BP_ALL	GO:0010493~Lewis a epitope biosynthetic process	1
GOTERM_BP_ALL	GO:0048575~short-day photoperiodism, flowering	1
GOTERM_BP_ALL	GO:0043479~pigment accumulation in tissues in response	1
GOTERM_BP_ALL	GO:0000079~regulation of cyclin-dependent protein kinas	1
GOTERM_BP_ALL	GO:0006109~regulation of carbohydrate metabolic proces	1

GOTERM_BP_ALL	GO:0009167~purine ribonucleoside monophosphate meta	1
GOTERM_BP_ALL	GO:0009103~lipopolysaccharide biosynthetic process	1
GOTERM_BP_ALL	GO:0006306~DNA methylation	1
GOTERM_BP_ALL	GO:0009561~megagametogenesis	1
GOTERM_BP_ALL	GO:0016107~sesquiterpenoid catabolic process	1
GOTERM_BP_ALL	GO:0034623~cellular macromolecular complex disassem	1
GOTERM_BP_ALL	GO:0051495~positive regulation of cytoskeleton organiza	1
GOTERM_BP_ALL	GO:0034433~steroid esterification	1
GOTERM_BP_ALL	GO:0009835~ripening	1
GOTERM_BP_ALL	GO:0009615~response to virus	1
GOTERM_BP_ALL	GO:0032879~regulation of localization	1
GOTERM_BP_ALL	GO:0055067~monovalent inorganic cation homeostasis	1
GOTERM_BP_ALL	GO:0048481~ovule development	1
GOTERM_BP_ALL	GO:0034655~nucleobase, nucleoside, nucleotide and nucl	1
GOTERM_BP_ALL	GO:0050918~positive chemotaxis	1
GOTERM_BP_ALL	GO:0010197~polar nucleus fusion	1
GOTERM_BP_ALL	GO:0043480~pigment accumulation in tissues	1
GOTERM_BP_ALL	GO:0009749~response to glucose stimulus	1
GOTERM_BP_ALL	GO:0006842~tricarboxylic acid transport	1
GOTERM_BP_ALL	GO:0010224~response to UV-B	1
GOTERM_BP_ALL	GO:0009245~lipid A biosynthetic process	1
GOTERM_BP_ALL	GO:0010362~negative regulation of anion channel activit	1
GOTERM_BP_ALL	GO:0006401~RNA catabolic process	1
GOTERM_BP_ALL	GO:0000398~nuclear mRNA splicing, via spliceosome	1
GOTERM_BP_ALL	GO:0016124~xanthophyll catabolic process	1
GOTERM_BP_ALL	GO:0010358~leaf shaping	1
GOTERM_BP_ALL	GO:0055081~anion homeostasis	1
GOTERM_BP_ALL	GO:0009292~genetic transfer	1
GOTERM_BP_ALL	GO:0044070~regulation of anion transport	1
GOTERM_BP_ALL	GO:0052546~cell wall pectin metabolic process	1
GOTERM_BP_ALL	GO:0055071~manganese ion homeostasis	1
GOTERM_BP_ALL	GO:0009910~negative regulation of flower development	1
GOTERM_BP_ALL	GO:0009961~response to 1-aminocyclopropane-1-carbox	1
GOTERM_BP_ALL	GO:0043069~negative regulation of programmed cell dea	1
GOTERM_BP_ALL	GO:0015865~purine nucleotide transport	1
GOTERM_BP_ALL	GO:0010393~galacturonan metabolic process	1
GOTERM_BP_ALL	GO:0050994~regulation of lipid catabolic process	1
GOTERM_BP_ALL	GO:0009750~response to fructose stimulus	1
GOTERM_BP_ALL	GO:0006353~transcription termination	1
GOTERM_BP_ALL	GO:0006626~protein targeting to mitochondrion	1
GOTERM_BP_ALL	GO:0000919~cell plate formation	1
GOTERM_BP_ALL	GO:0051640~organelle localization	1
GOTERM_BP_ALL	GO:0010232~vascular transport	1
GOTERM_BP_ALL	GO:0010271~regulation of chlorophyll catabolic process	1
GOTERM_BP_ALL	GO:0043624~cellular protein complex disassembly	1
GOTERM_BP_ALL	GO:0010361~regulation of anion channel activity by blue	1
GOTERM_BP_ALL	GO:0044038~cell wall macromolecule biosynthetic proce	1

GOTERM_BP_ALL	GO:0031086~nuclear-transcribed mRNA catabolic proces	1
GOTERM_BP_ALL	GO:0009817~defense response to fungus, incompatible in	1
GOTERM_BP_ALL	GO:0017038~protein import	1
GOTERM_BP_ALL	GO:0009119~ribonucleoside metabolic process	1
GOTERM_BP_ALL	GO:0010078~maintenance of root meristem identity	1
GOTERM_BP_ALL	GO:0006897~endocytosis	1
GOTERM_BP_ALL	GO:0032984~macromolecular complex disassembly	1
GOTERM_BP_ALL	GO:0006402~mRNA catabolic process	1
GOTERM_BP_ALL	GO:0009088~threonine biosynthetic process	1
GOTERM_BP_ALL	GO:0080006~internode patterning	1
GOTERM_BP_ALL	GO:0048015~phosphoinositide-mediated signaling	1
GOTERM_BP_ALL	GO:0010112~regulation of systemic acquired resistance	1
GOTERM_BP_ALL	GO:0031329~regulation of cellular catabolic process	1
GOTERM_BP_ALL	GO:0030244~cellulose biosynthetic process	1
GOTERM_BP_ALL	GO:0005983~starch catabolic process	1
GOTERM_BP_ALL	GO:0008285~negative regulation of cell proliferation	1
GOTERM_BP_ALL	GO:0010438~cellular response to sulfur starvation	1
GOTERM_BP_ALL	GO:0034284~response to monosaccharide stimulus	1
GOTERM_BP_ALL	GO:0000087~M phase of mitotic cell cycle	1
GOTERM_BP_ALL	GO:0046015~regulation of transcription by glucose	1
GOTERM_BP_ALL	GO:0010383~cell wall polysaccharide metabolic process	1
GOTERM_BP_ALL	GO:0035315~hair cell differentiation	1
GOTERM_BP_ALL	GO:0009962~regulation of flavonoid biosynthetic process	1
GOTERM_BP_ALL	GO:0043478~pigment accumulation in response to UV lig	1
GOTERM_BP_ALL	GO:0015804~neutral amino acid transport	1
GOTERM_BP_ALL	GO:0007186~G-protein coupled receptor protein signalin	1
GOTERM_BP_ALL	GO:0010675~regulation of cellular carbohydrate metaboli	1
GOTERM_BP_ALL	GO:0051347~positive regulation of transferase activity	1
GOTERM_BP_ALL	GO:0032410~negative regulation of transporter activity	1
GOTERM_BP_ALL	GO:0008356~asymmetric cell division	1
GOTERM_BP_ALL	GO:0022411~cellular component disassembly	1
GOTERM_BP_ALL	GO:0022403~cell cycle phase	1
GOTERM_BP_ALL	GO:0034762~regulation of transmembrane transport	1
GOTERM_BP_ALL	GO:0006885~regulation of pH	1
GOTERM_BP_ALL	GO:0010352~lithium ion export	1
GOTERM_BP_ALL	GO:0010026~trichome differentiation	1
GOTERM_BP_ALL	GO:0010090~trichome morphogenesis	1
GOTERM_BP_ALL	GO:0030418~nicotianamine biosynthetic process	1
GOTERM_BP_ALL	GO:0006656~phosphatidylcholine biosynthetic process	1
GOTERM_BP_ALL	GO:0034656~nucleobase, nucleoside and nucleotide catab	1
GOTERM_BP_ALL	GO:0009864~induced systemic resistance, jasmonic acid	1
GOTERM_BP_ALL	GO:0009845~seed germination	1
GOTERM_BP_ALL	GO:0043648~dicarboxylic acid metabolic process	1
GOTERM_BP_ALL	GO:0009567~double fertilization forming a zygote and en	1
GOTERM_BP_ALL	GO:0009880~embryonic pattern specification	1
GOTERM_BP_ALL	GO:0051302~regulation of cell division	1
GOTERM_BP_ALL	GO:0010288~response to lead ion	1

GOTERM_BP_ALL	GO:0043039~tRNA aminoacylation	1
GOTERM_BP_ALL	GO:0009075~histidine family amino acid metabolic proce	1
GOTERM_BP_ALL	GO:0009726~detection of endogenous stimulus	1
GOTERM_BP_ALL	GO:0048480~stigma development	1
GOTERM_BP_ALL	GO:0043473~pigmentation	1
GOTERM_BP_ALL	GO:0006913~nucleocytoplasmic transport	1
GOTERM_BP_ALL	GO:0042402~biogenic amine catabolic process	1
GOTERM_BP_ALL	GO:0010119~regulation of stomatal movement	1
GOTERM_BP_ALL	GO:0030417~nicotianamine metabolic process	1
GOTERM_BP_ALL	GO:0046854~phosphoinositide phosphorylation	1
GOTERM_BP_ALL	GO:0043038~amino acid activation	1
GOTERM_BP_ALL	GO:0010017~red or far red light signaling pathway	1
GOTERM_BP_ALL	GO:0033559~unsaturated fatty acid metabolic process	1
GOTERM_BP_ALL	GO:0045013~negative regulation of transcription by carb	1
GOTERM_BP_ALL	GO:0009076~histidine family amino acid biosynthetic pro	1
GOTERM_BP_ALL	GO:0009448~gamma-aminobutyric acid metabolic proces	1
GOTERM_BP_ALL	GO:0009813~flavonoid biosynthetic process	1
GOTERM_BP_ALL	GO:0010021~amylopectin biosynthetic process	1
GOTERM_BP_ALL	GO:0045040~protein import into mitochondrial outer me	1
GOTERM_BP_ALL	GO:0009809~lignin biosynthetic process	1
GOTERM_BP_ALL	GO:0009156~ribonucleoside monophosphate biosynthetic	1
GOTERM_BP_ALL	GO:0043241~protein complex disassembly	1
GOTERM_BP_ALL	GO:0046110~xanthine metabolic process	1
GOTERM_BP_ALL	GO:0046653~tetrahydrofolate metabolic process	1
GOTERM_BP_ALL	GO:0006595~polyamine metabolic process	1
GOTERM_BP_ALL	GO:0019354~siroheme biosynthetic process	1
GOTERM_BP_ALL	GO:0010091~trichome branching	1
GOTERM_BP_ALL	GO:0006538~glutamate catabolic process	1
GOTERM_BP_ALL	GO:0000578~embryonic axis specification	1
GOTERM_BP_ALL	GO:0045047~protein targeting to ER	1
GOTERM_BP_ALL	GO:0042559~pteridine and derivative biosynthetic proces	1
GOTERM_BP_ALL	GO:0006620~posttranslational protein targeting to membr	1
GOTERM_BP_ALL	GO:0019751~polyol metabolic process	1
GOTERM_BP_ALL	GO:0070726~cell wall assembly	1
GOTERM_BP_ALL	GO:0010315~auxin efflux	1
GOTERM_BP_ALL	GO:0055069~zinc ion homeostasis	1
GOTERM_BP_ALL	GO:0009799~determination of symmetry	1
GOTERM_BP_ALL	GO:0051051~negative regulation of transport	1
GOTERM_BP_ALL	GO:0019321~pentose metabolic process	1
GOTERM_BP_ALL	GO:0016104~triterpenoid biosynthetic process	1
GOTERM_BP_ALL	GO:0009645~response to low light intensity stimulus	1
GOTERM_BP_ALL	GO:0009846~pollen germination	1
GOTERM_BP_ALL	GO:0016109~tetraterpenoid biosynthetic process	1
GOTERM_BP_ALL	GO:0007006~mitochondrial membrane organization	1
GOTERM_BP_ALL	GO:0030245~cellulose catabolic process	1
GOTERM_BP_ALL	GO:0065002~intracellular protein transmembrane transpo	1
GOTERM_BP_ALL	GO:0051493~regulation of cytoskeleton organization	1

GOTERM_BP_ALL	GO:0046834~lipid phosphorylation	1
GOTERM_BP_ALL	GO:0006636~unsaturated fatty acid biosynthetic process	1
GOTERM_BP_ALL	GO:0009866~induced systemic resistance, ethylene media	1
GOTERM_BP_ALL	GO:0015914~phospholipid transport	1
GOTERM_BP_ALL	GO:0010599~RNA interference, production of lsiRNA	1
GOTERM_BP_ALL	GO:0048285~organelle fission	1
GOTERM_BP_ALL	GO:0006119~oxidative phosphorylation	1
GOTERM_BP_ALL	GO:0010589~leaf proximal/distal pattern formation	1
GOTERM_BP_ALL	GO:0044270~nitrogen compound catabolic process	1
GOTERM_BP_ALL	GO:0009556~microsporogenesis	1
GOTERM_BP_ALL	GO:0000377~RNA splicing, via transesterification reactio	1
GOTERM_BP_ALL	GO:0051127~positive regulation of actin nucleation	1
GOTERM_BP_ALL	GO:0032970~regulation of actin filament-based process	1
GOTERM_BP_ALL	GO:0001708~cell fate specification	1
GOTERM_BP_ALL	GO:0045786~negative regulation of cell cycle	1
GOTERM_BP_ALL	GO:0009635~response to herbicide	1
GOTERM_BP_ALL	GO:0043476~pigment accumulation	1
GOTERM_BP_ALL	GO:0009903~chloroplast avoidance movement	1
GOTERM_BP_ALL	GO:0043450~alkene biosynthetic process	1
GOTERM_BP_ALL	GO:0046700~heterocycle catabolic process	1
GOTERM_BP_ALL	GO:0007050~cell cycle arrest	1
GOTERM_BP_ALL	GO:0015800~acidic amino acid transport	1
GOTERM_BP_ALL	GO:0006145~purine base catabolic process	1
GOTERM_BP_ALL	GO:0048598~embryonic morphogenesis	1
GOTERM_BP_ALL	GO:0000279~M phase	1
GOTERM_BP_ALL	GO:0070589~cellular component macromolecule biosynt	1
GOTERM_BP_ALL	GO:0055070~copper ion homeostasis	1
GOTERM_BP_ALL	GO:0007346~regulation of mitotic cell cycle	1
GOTERM_BP_ALL	GO:0000956~nuclear-transcribed mRNA catabolic proces	1

Pathways up-regulated in *aba* 1.6 (KEGG and DAVID analysis)

Category	Term	Gene Count
KEGG_PATHWAY	ath00360:Phenylalanine metabolism	10
KEGG_PATHWAY	ath00680:Methane metabolism	9
KEGG_PATHWAY	ath00940:Phenylpropanoid biosynthesis	10
KEGG_PATHWAY	ath00905:Brassinosteroid biosynthesis	3
KEGG_PATHWAY	ath00960:Tropane, piperidine and pyridine alkaloid biosy	4
PANTHER_PATHWAY	P02723:Adenine and hypoxanthine salvage pathway	3
KEGG_PATHWAY	ath03410:Base excision repair	5
PANTHER_PATHWAY	P00037:Ionotropic glutamate receptor pathway	3
PANTHER_PATHWAY	P00034:Integrin signalling pathway	6
KEGG_PATHWAY	ath00904:Diterpenoid biosynthesis	3
KEGG_PATHWAY	ath00500:Starch and sucrose metabolism	7
PANTHER_PATHWAY	P04393:Ras Pathway	4
PANTHER_PATHWAY	P00033:Insulin/IGF pathway-protein kinase B signaling c	3
PANTHER_PATHWAY	P00021:FGF signaling pathway	7

KEGG_PATHWAY	ath00380:Tryptophan metabolism	4
PANTHER_PATHWAY	P00047:PDGF signaling pathway	6
KEGG_PATHWAY	ath00906:Carotenoid biosynthesis	3
PANTHER_PATHWAY	P00036:Interleukin signaling pathway	4
KEGG_PATHWAY	ath00100:Steroid biosynthesis	3
KEGG_PATHWAY	ath04130:SNARE interactions in vesicular transport	4
KEGG_PATHWAY	ath01040:Biosynthesis of unsaturated fatty acids	3
KEGG_PATHWAY	ath00510:N-Glycan biosynthesis	3
PANTHER_PATHWAY	P02766:Phenylethylamine degradation	2
PANTHER_PATHWAY	P00025:Hedgehog signaling pathway	3
PANTHER_PATHWAY	P00018:EGF receptor signaling pathway	5
KEGG_PATHWAY	ath00945:Stilbenoid, diarylheptanoid and gingerol biosynt	4
KEGG_PATHWAY	ath00910:Nitrogen metabolism	3
PANTHER_PATHWAY	P00039:Metabotropic glutamate receptor group III pathwa	2
PANTHER_PATHWAY	P04398:p53 pathway feedback loops 2	2
KEGG_PATHWAY	ath00903:Limonene and pinene degradation	4
PANTHER_PATHWAY	P00016:Cytoskeletal regulation by Rho GTPase	3
KEGG_PATHWAY	ath00908:Zeatin biosynthesis	2
PANTHER_PATHWAY	P02746:Heme biosynthesis	2
PANTHER_PATHWAY	P00055:Transcription regulation by bZIP transcription fac	4
KEGG_PATHWAY	ath01061:Biosynthesis of phenylpropanoids	10
PANTHER_PATHWAY	P00026:Heterotrimeric G-protein signaling pathway-Gi al	2
PANTHER_PATHWAY	P00005:Angiogenesis	2
PANTHER_PATHWAY	P00019:Endothelin signaling pathway	2
PANTHER_PATHWAY	P00031:Inflammation mediated by chemokine and cytokin	3
KEGG_PATHWAY	ath00270:Cysteine and methionine metabolism	3
KEGG_PATHWAY	ath00040:Pentose and glucuronate interconversions	2
KEGG_PATHWAY	ath00052:Galactose metabolism	2
PANTHER_PATHWAY	P00048:PI3 kinase pathway	2
PANTHER_PATHWAY	P00052:TGF-beta signaling pathway	2
KEGG_PATHWAY	ath01070:Biosynthesis of plant hormones	11
KEGG_PATHWAY	ath00280:Valine, leucine and isoleucine degradation	2
PANTHER_PATHWAY	P00059:p53 pathway	3
KEGG_PATHWAY	ath01062:Biosynthesis of terpenoids and steroids	7
PANTHER_PATHWAY	P00006:Apoptosis signaling pathway	3
PANTHER_PATHWAY	P00057:Wnt signaling pathway	4
KEGG_PATHWAY	ath04070:Phosphatidylinositol signaling system	2
KEGG_PATHWAY	ath00562:Inositol phosphate metabolism	2
KEGG_PATHWAY	ath01064:Biosynthesis of alkaloids derived from ornithine	6
KEGG_PATHWAY	ath00030:Pentose phosphate pathway	2
PANTHER_PATHWAY	P00023:General transcription regulation	2
PANTHER_PATHWAY	P00029:Huntington disease	3
KEGG_PATHWAY	ath00190:Oxidative phosphorylation	4
KEGG_PATHWAY	ath04144:Endocytosis	2
KEGG_PATHWAY	ath00520:Amino sugar and nucleotide sugar metabolism	2
KEGG_PATHWAY	ath00230:Purine metabolism	2
KEGG_PATHWAY	ath01065:Biosynthesis of alkaloids derived from histidine	2

KEGG_PATHWAY	ath00400:Phenylalanine, tyrosine and tryptophan biosynth	1
KEGG_PATHWAY	ath00966:Glucosinolate biosynthesis	1
KEGG_PATHWAY	ath01063:Biosynthesis of alkaloids derived from shikimat	1
KEGG_PATHWAY	ath00480:Glutathione metabolism	1
KEGG_PATHWAY	ath00260:Glycine, serine and threonine metabolism	1
KEGG_PATHWAY	ath00592:alpha-Linolenic acid metabolism	1
KEGG_PATHWAY	ath00941:Flavonoid biosynthesis	1
KEGG_PATHWAY	ath00860:Porphyrin and chlorophyll metabolism	1
KEGG_PATHWAY	ath04140:Regulation of autophagy	1
KEGG_PATHWAY	ath00563:Glycosylphosphatidylinositol(GPI)-anchor biosy	1
KEGG_PATHWAY	ath00564:Glycerophospholipid metabolism	1
KEGG_PATHWAY	ath00061:Fatty acid biosynthesis	1
KEGG_PATHWAY	ath00565:Ether lipid metabolism	1
KEGG_PATHWAY	ath00300:Lysine biosynthesis	1
KEGG_PATHWAY	ath00250:Alanine, aspartate and glutamate metabolism	1
KEGG_PATHWAY	ath03050:Proteasome	1
KEGG_PATHWAY	ath00944:Flavone and flavonol biosynthesis	1
KEGG_PATHWAY	ath03040:Spliceosome	1
KEGG_PATHWAY	ath03018:RNA degradation	1
KEGG_PATHWAY	ath00511:Other glycan degradation	1
KEGG_PATHWAY	ath04120:Ubiquitin mediated proteolysis	1
KEGG_PATHWAY	ath00710:Carbon fixation in photosynthetic organisms	1
KEGG_PATHWAY	ath00600:Sphingolipid metabolism	1
KEGG_PATHWAY	ath00010:Glycolysis / Gluconeogenesis	1
KEGG_PATHWAY	ath00195:Photosynthesis	1
KEGG_PATHWAY	ath00770:Pantothenate and CoA biosynthesis	1
KEGG_PATHWAY	ath00350:Tyrosine metabolism	1
KEGG_PATHWAY	ath00330:Arginine and proline metabolism	1
KEGG_PATHWAY	ath00232:Caffeine metabolism	1
KEGG_PATHWAY	ath00950:Isoquinoline alkaloid biosynthesis	1
KEGG_PATHWAY	ath03022:Basal transcription factors	1
KEGG_PATHWAY	ath01066:Biosynthesis of alkaloids derived from terpenoi	1
KEGG_PATHWAY	ath00460:Cyanoamino acid metabolism	1
KEGG_PATHWAY	ath00740:Riboflavin metabolism	1
KEGG_PATHWAY	ath00290:Valine, leucine and isoleucine biosynthesis	1
PANTHER_PATHWAY	P00003:Alzheimer disease-amyloid secretase pathway	1
PANTHER_PATHWAY	P02762:Pentose phosphate pathway	1
PANTHER_PATHWAY	P04397:p53 pathway by glucose deprivation	1
PANTHER_PATHWAY	P05734:Synaptic_vesicle_trafficking	1
PANTHER_PATHWAY	P02750:Lipoate_biosynthesis	1
PANTHER_PATHWAY	P00053:T cell activation	1
PANTHER_PATHWAY	P00056:VEGF signaling pathway	1
PANTHER_PATHWAY	P00044:Nicotinic acetylcholine receptor signaling pathwa	1
PANTHER_PATHWAY	P02731:Biotin biosynthesis	1
PANTHER_PATHWAY	P02763:Peptidoglycan biosynthesis	1
PANTHER_PATHWAY	P00032:Insulin/IGF pathway-mitogen activated protein ki	1
PANTHER_PATHWAY	P02730:Asparagine and aspartate biosynthesis	1

PANTHER_PATHWAY	P02738:De novo purine biosynthesis	1
PANTHER_PATHWAY	P00049:Parkinson disease	1
PANTHER_PATHWAY	P00046:Oxidative stress response	1
PANTHER_PATHWAY	P02765:Phenylalanine biosynthesis	1
PANTHER_PATHWAY	P02748:Isoleucine biosynthesis	1
PANTHER_PATHWAY	P00012:Cadherin signaling pathway	1
PANTHER_PATHWAY	P02784:Tyrosine biosynthesis	1
PANTHER_PATHWAY	P02749:Leucine biosynthesis	1
PANTHER_PATHWAY	P02736:Coenzyme A biosynthesis	1
PANTHER_PATHWAY	P00010:B cell activation	1
PANTHER_PATHWAY	P02724:Alanine biosynthesis	1
PANTHER_PATHWAY	P02747:Histidine biosynthesis	1
PANTHER_PATHWAY	P02785:Valine biosynthesis	1
PANTHER_PATHWAY	P04395:Vasopressin synthesis	1
PANTHER_PATHWAY	P00017:DNA replication	1
PANTHER_PATHWAY	P05914:Nicotine degradation	1
PANTHER_PATHWAY	P00030:Hypoxia response via HIF activation	1
PANTHER_PATHWAY	P00004:Alzheimer disease-presenilin pathway	1
PANTHER_PATHWAY	P00035:Interferon-gamma signaling pathway	1

Supplementary Table S7. Differentially expressed genes grouped in different functional and pathways categories. Down-regulated genes of *aba* 1.6 grouped in different cellular functions categories (DAVID analysis)

Category	Term	Gene Count
GOTERM_BP_ALL	GO:0009628~response to abiotic stimulus	149
GOTERM_BP_ALL	GO:0009408~response to heat	42
GOTERM_BP_ALL	GO:0006950~response to stress	231
GOTERM_BP_ALL	GO:0009266~response to temperature stimulus	68
GOTERM_BP_ALL	GO:0050896~response to stimulus	327
GOTERM_BP_ALL	GO:0042221~response to chemical stimulus	182
GOTERM_BP_ALL	GO:0009416~response to light stimulus	68
GOTERM_BP_ALL	GO:0009314~response to radiation	68
GOTERM_BP_ALL	GO:0006979~response to oxidative stress	49
GOTERM_BP_ALL	GO:0009644~response to high light intensity	19
GOTERM_BP_ALL	GO:0010035~response to inorganic substance	71
GOTERM_CC_ALL	GO:0044434~chloroplast part	98
GOTERM_CC_ALL	GO:0044435~plastid part	99
GOTERM_BP_ALL	GO:0009642~response to light intensity	21
GOTERM_BP_ALL	GO:0015979~photosynthesis	33
GOTERM_BP_ALL	GO:0042542~response to hydrogen peroxide	26
GOTERM_CC_ALL	GO:0009507~chloroplast	237
GOTERM_CC_ALL	GO:0009526~plastid envelope	56
GOTERM_CC_ALL	GO:0009536~plastid	240
GOTERM_CC_ALL	GO:0009579~thylakoid	58
GOTERM_BP_ALL	GO:0055114~oxidation reduction	111
GOTERM_CC_ALL	GO:0009534~chloroplast thylakoid	46
GOTERM_CC_ALL	GO:0031976~plastid thylakoid	46
GOTERM_CC_ALL	GO:0055035~plastid thylakoid membrane	41
GOTERM_CC_ALL	GO:0009535~chloroplast thylakoid membrane	41
GOTERM_CC_ALL	GO:0031984~organelle subcompartment	46
GOTERM_MF_ALL	GO:0030528~transcription regulator activity	157
GOTERM_BP_ALL	GO:0000302~response to reactive oxygen species	27
GOTERM_CC_ALL	GO:0009941~chloroplast envelope	53
GOTERM_CC_ALL	GO:0010287~plastoglobule	17
GOTERM_CC_ALL	GO:0042651~thylakoid membrane	41
GOTERM_CC_ALL	GO:0044436~thylakoid part	45
GOTERM_BP_ALL	GO:0006091~generation of precursor metabolites and energ	50
GOTERM_CC_ALL	GO:0034357~photosynthetic membrane	42
GOTERM_BP_ALL	GO:0019748~secondary metabolic process	50
GOTERM_BP_ALL	GO:0019684~photosynthesis, light reaction	19
GOTERM_MF_ALL	GO:0003700~transcription factor activity	136
GOTERM_MF_ALL	GO:0016168~chlorophyll binding	11
GOTERM_CC_ALL	GO:0009570~chloroplast stroma	47
GOTERM_BP_ALL	GO:0031323~regulation of cellular metabolic process	168
GOTERM_BP_ALL	GO:0022900~electron transport chain	26
GOTERM_CC_ALL	GO:0009532~plastid stroma	47
GOTERM_BP_ALL	GO:0031326~regulation of cellular biosynthetic process	160
GOTERM_BP_ALL	GO:0009889~regulation of biosynthetic process	160

GOTERM_CC_ALL	GO:0005622~intracellular	602
GOTERM_BP_ALL	GO:0009698~phenylpropanoid metabolic process	25
GOTERM_BP_ALL	GO:0009753~response to jasmonic acid stimulus	24
GOTERM_CC_ALL	GO:0031967~organelle envelope	68
GOTERM_BP_ALL	GO:0045449~regulation of transcription	154
GOTERM_CC_ALL	GO:0031975~envelope	68
GOTERM_BP_ALL	GO:0010556~regulation of macromolecule biosynthetic pro	156
GOTERM_BP_ALL	GO:0051171~regulation of nitrogen compound metabolic p	155
GOTERM_BP_ALL	GO:0019219~regulation of nucleobase, nucleoside, nucleoti	154
GOTERM_MF_ALL	GO:0016491~oxidoreductase activity	117
GOTERM_BP_ALL	GO:0009414~response to water deprivation	25
GOTERM_BP_ALL	GO:0010038~response to metal ion	42
GOTERM_BP_ALL	GO:0009765~photosynthesis, light harvesting	10
GOTERM_BP_ALL	GO:0080090~regulation of primary metabolic process	161
GOTERM_BP_ALL	GO:0009611~response to wounding	22
GOTERM_BP_ALL	GO:0006575~cellular amino acid derivative metabolic proc	33
GOTERM_BP_ALL	GO:0019222~regulation of metabolic process	172
GOTERM_BP_ALL	GO:0010033~response to organic substance	96
GOTERM_BP_ALL	GO:0009415~response to water	25
GOTERM_BP_ALL	GO:0010468~regulation of gene expression	160
GOTERM_BP_ALL	GO:0045454~cell redox homeostasis	21
GOTERM_BP_ALL	GO:0046686~response to cadmium ion	36
GOTERM_BP_ALL	GO:0006970~response to osmotic stress	41
GOTERM_BP_ALL	GO:0010114~response to red light	12
GOTERM_BP_ALL	GO:0009409~response to cold	28
GOTERM_BP_ALL	GO:0009699~phenylpropanoid biosynthetic process	19
GOTERM_CC_ALL	GO:0005737~cytoplasm	426
GOTERM_BP_ALL	GO:0060255~regulation of macromolecule metabolic proce	162
GOTERM_CC_ALL	GO:0044424~intracellular part	572
GOTERM_BP_ALL	GO:0042398~cellular amino acid derivative biosynthetic pr	24
GOTERM_MF_ALL	GO:0016702~oxidoreductase activity, acting on single dono	12
GOTERM_MF_ALL	GO:0046906~tetrapyrrole binding	39
GOTERM_BP_ALL	GO:0009411~response to UV	13
GOTERM_CC_ALL	GO:0030076~light-harvesting complex	8
GOTERM_BP_ALL	GO:0010218~response to far red light	10
GOTERM_MF_ALL	GO:0016209~antioxidant activity	19
GOTERM_BP_ALL	GO:0009743~response to carbohydrate stimulus	24
GOTERM_MF_ALL	GO:0051213~dioxygenase activity	12
GOTERM_MF_ALL	GO:0009055~electron carrier activity	56
GOTERM_BP_ALL	GO:0009719~response to endogenous stimulus	78
GOTERM_MF_ALL	GO:0004601~peroxidase activity	17
GOTERM_MF_ALL	GO:0016684~oxidoreductase activity, acting on peroxide as	17
GOTERM_CC_ALL	GO:0043231~intracellular membrane-bounded organelle	499
GOTERM_CC_ALL	GO:0043227~membrane-bounded organelle	499
GOTERM_MF_ALL	GO:0046527~glucosyltransferase activity	18
GOTERM_BP_ALL	GO:0009639~response to red or far red light	21
GOTERM_BP_ALL	GO:0007623~circadian rhythm	9

GOTERM_BP_ALL	GO:0006457~protein folding	27
GOTERM_MF_ALL	GO:0016701~oxidoreductase activity, acting on single dono	12
GOTERM_MF_ALL	GO:0016667~oxidoreductase activity, acting on sulfur grou	15
GOTERM_BP_ALL	GO:0010200~response to chitin	17
GOTERM_BP_ALL	GO:0006350~transcription	97
GOTERM_BP_ALL	GO:0009651~response to salt stress	35
GOTERM_BP_ALL	GO:0070301~cellular response to hydrogen peroxide	13
GOTERM_BP_ALL	GO:0042744~hydrogen peroxide catabolic process	13
GOTERM_BP_ALL	GO:0006355~regulation of transcription, DNA-dependent	82
GOTERM_BP_ALL	GO:0042743~hydrogen peroxide metabolic process	13
GOTERM_BP_ALL	GO:0051252~regulation of RNA metabolic process	82
GOTERM_BP_ALL	GO:0009605~response to external stimulus	33
GOTERM_BP_ALL	GO:0010224~response to UV-B	9
GOTERM_BP_ALL	GO:0015977~carbon utilization by fixation of carbon dioxi	6
GOTERM_CC_ALL	GO:0044444~cytoplasmic part	378
GOTERM_CC_ALL	GO:0048046~apoplast	34
GOTERM_BP_ALL	GO:0009767~photosynthetic electron transport chain	7
GOTERM_CC_ALL	GO:0009521~photosystem	11
GOTERM_MF_ALL	GO:0035251~UDP-glucosyltransferase activity	15
GOTERM_BP_ALL	GO:0048511~rhythmic process	9
GOTERM_BP_ALL	GO:0006833~water transport	4
GOTERM_BP_ALL	GO:0042044~fluid transport	4
GOTERM_BP_ALL	GO:0034614~cellular response to reactive oxygen species	13
GOTERM_BP_ALL	GO:0034599~cellular response to oxidative stress	13
GOTERM_CC_ALL	GO:0043229~intracellular organelle	510
GOTERM_CC_ALL	GO:0043226~organelle	510
GOTERM_MF_ALL	GO:0008943~glyceraldehyde-3-phosphate dehydrogenase a	4
GOTERM_CC_ALL	GO:0010319~stromule	7
GOTERM_BP_ALL	GO:0050794~regulation of cellular process	219
GOTERM_BP_ALL	GO:0006519~cellular amino acid and derivative metabolic	49
GOTERM_BP_ALL	GO:0019438~aromatic compound biosynthetic process	22
GOTERM_BP_ALL	GO:0009813~flavonoid biosynthetic process	9
GOTERM_CC_ALL	GO:0031977~thylakoid lumen	12
GOTERM_MF_ALL	GO:0016564~transcription repressor activity	9
GOTERM_MF_ALL	GO:0005506~iron ion binding	55
GOTERM_BP_ALL	GO:0010286~heat acclimation	5
GOTERM_MF_ALL	GO:0016563~transcription activator activity	16
GOTERM_BP_ALL	GO:0006725~cellular aromatic compound metabolic proces	31
GOTERM_MF_ALL	GO:0003677~DNA binding	156
GOTERM_MF_ALL	GO:0043565~sequence-specific DNA binding	39
GOTERM_BP_ALL	GO:0050789~regulation of biological process	233
GOTERM_BP_ALL	GO:0009812~flavonoid metabolic process	9
GOTERM_MF_ALL	GO:0015250~water channel activity	7
GOTERM_MF_ALL	GO:0005372~water transporter activity	7
GOTERM_CC_ALL	GO:0031090~organelle membrane	61
GOTERM_BP_ALL	GO:0009737~response to abscisic acid stimulus	26
GOTERM_BP_ALL	GO:0018298~protein-chromophore linkage	6

GOTERM_MF_ALL	GO:0051082~unfolded protein binding	13
GOTERM_BP_ALL	GO:0019725~cellular homeostasis	22
GOTERM_MF_ALL	GO:0050660~FAD binding	17
GOTERM_CC_ALL	GO:0005618~cell wall	46
GOTERM_MF_ALL	GO:0050662~coenzyme binding	31
GOTERM_BP_ALL	GO:0006800~oxygen and reactive oxygen species metaboli	13
GOTERM_BP_ALL	GO:0016053~organic acid biosynthetic process	34
GOTERM_BP_ALL	GO:0046394~carboxylic acid biosynthetic process	34
GOTERM_CC_ALL	GO:0009522~photosystem I	6
GOTERM_MF_ALL	GO:0015036~disulfide oxidoreductase activity	9
GOTERM_BP_ALL	GO:0006808~regulation of nitrogen utilization	5
GOTERM_MF_ALL	GO:0016765~transferase activity, transferring alkyl or aryl	14
GOTERM_BP_ALL	GO:0042592~homeostatic process	25
GOTERM_CC_ALL	GO:0009523~photosystem II	8
GOTERM_CC_ALL	GO:0030312~external encapsulating structure	46
GOTERM_MF_ALL	GO:0016830~carbon-carbon lyase activity	13
GOTERM_BP_ALL	GO:0042180~cellular ketone metabolic process	57
GOTERM_BP_ALL	GO:0009725~response to hormone stimulus	65
GOTERM_MF_ALL	GO:0000156~two-component response regulator activity	7
GOTERM_MF_ALL	GO:0016705~oxidoreductase activity, acting on paired don	19
GOTERM_MF_ALL	GO:0048037~cofactor binding	40
GOTERM_MF_ALL	GO:0015035~protein disulfide oxidoreductase activity	8
GOTERM_MF_ALL	GO:0046914~transition metal ion binding	170
GOTERM_BP_ALL	GO:0005984~disaccharide metabolic process	7
GOTERM_MF_ALL	GO:0016207~4-coumarate-CoA ligase activity	4
GOTERM_CC_ALL	GO:0005576~extracellular region	81
GOTERM_BP_ALL	GO:0009637~response to blue light	8
GOTERM_BP_ALL	GO:0009735~response to cytokinin stimulus	10
GOTERM_CC_ALL	GO:0044422~organelle part	162
GOTERM_CC_ALL	GO:0044446~intracellular organelle part	162
GOTERM_BP_ALL	GO:0009063~cellular amino acid catabolic process	7
GOTERM_BP_ALL	GO:0006733~oxidoreduction coenzyme metabolic process	8
GOTERM_MF_ALL	GO:0016903~oxidoreductase activity, acting on the aldehyd	9
GOTERM_BP_ALL	GO:0042364~water-soluble vitamin biosynthetic process	8
GOTERM_BP_ALL	GO:0009751~response to salicylic acid stimulus	15
GOTERM_BP_ALL	GO:0046351~disaccharide biosynthetic process	6
GOTERM_MF_ALL	GO:0016840~carbon-nitrogen lyase activity	5
GOTERM_MF_ALL	GO:0004737~pyruvate decarboxylase activity	3
GOTERM_MF_ALL	GO:0004506~squalene monooxygenase activity	3
GOTERM_BP_ALL	GO:0009773~photosynthetic electron transport in photosyst	4
GOTERM_BP_ALL	GO:0042548~regulation of photosynthesis, light reaction	4
GOTERM_BP_ALL	GO:0019253~reductive pentose-phosphate cycle	4
GOTERM_BP_ALL	GO:0016051~carbohydrate biosynthetic process	22
GOTERM_BP_ALL	GO:0009310~amine catabolic process	7
GOTERM_BP_ALL	GO:0006721~terpenoid metabolic process	11
GOTERM_BP_ALL	GO:0009723~response to ethylene stimulus	22
GOTERM_BP_ALL	GO:0006767~water-soluble vitamin metabolic process	8

GOTERM_BP_ALL	GO:0009744~response to sucrose stimulus	6
GOTERM_BP_ALL	GO:0019685~photosynthesis, dark reaction	4
GOTERM_BP_ALL	GO:0043467~regulation of generation of precursor metabol	4
GOTERM_BP_ALL	GO:0019752~carboxylic acid metabolic process	54
GOTERM_BP_ALL	GO:0043436~oxoacid metabolic process	54
GOTERM_BP_ALL	GO:0006082~organic acid metabolic process	54
GOTERM_BP_ALL	GO:0034285~response to disaccharide stimulus	6
GOTERM_MF_ALL	GO:0004365~glyceraldehyde-3-phosphate dehydrogenase (	3
GOTERM_MF_ALL	GO:0045543~gibberellin 2-beta-dioxygenase activity	3
GOTERM_BP_ALL	GO:0042819~vitamin B6 biosynthetic process	3
GOTERM_BP_ALL	GO:0010205~photoinhibition	3
GOTERM_BP_ALL	GO:0043155~negative regulation of photosynthesis, light re	3
GOTERM_BP_ALL	GO:0008615~pyridoxine biosynthetic process	3
GOTERM_BP_ALL	GO:0008614~pyridoxine metabolic process	3
GOTERM_BP_ALL	GO:0042816~vitamin B6 metabolic process	3
GOTERM_BP_ALL	GO:0009607~response to biotic stimulus	60
GOTERM_BP_ALL	GO:0006006~glucose metabolic process	12
GOTERM_BP_ALL	GO:0010109~regulation of photosynthesis	4
GOTERM_BP_ALL	GO:0006633~fatty acid biosynthetic process	15
GOTERM_BP_ALL	GO:0006739~NADP metabolic process	6
GOTERM_BP_ALL	GO:0009312~oligosaccharide biosynthetic process	6
GOTERM_MF_ALL	GO:0004402~histone acetyltransferase activity	4
GOTERM_MF_ALL	GO:0004468~lysine N-acetyltransferase activity	4
GOTERM_BP_ALL	GO:0009311~oligosaccharide metabolic process	7
GOTERM_BP_ALL	GO:0032787~monocarboxylic acid metabolic process	28
GOTERM_MF_ALL	GO:0016831~carboxy-lyase activity	8
GOTERM_BP_ALL	GO:0005985~sucrose metabolic process	4
GOTERM_BP_ALL	GO:0022610~biological adhesion	4
GOTERM_BP_ALL	GO:0007155~cell adhesion	4
GOTERM_BP_ALL	GO:0016114~terpenoid biosynthetic process	9
GOTERM_MF_ALL	GO:0016723~oxidoreductase activity, oxidizing metal ions,	3
GOTERM_MF_ALL	GO:0000293~ferric-chelate reductase activity	3
GOTERM_MF_ALL	GO:0020037~heme binding	28
GOTERM_BP_ALL	GO:0008610~lipid biosynthetic process	31
GOTERM_MF_ALL	GO:0016597~amino acid binding	6
GOTERM_MF_ALL	GO:0030414~peptidase inhibitor activity	6
GOTERM_MF_ALL	GO:0030976~thiamin pyrophosphate binding	3
GOTERM_MF_ALL	GO:0016668~oxidoreductase activity, acting on sulfur grou	3
GOTERM_MF_ALL	GO:0004869~cysteine-type endopeptidase inhibitor activity	3
GOTERM_BP_ALL	GO:0006066~alcohol metabolic process	23
GOTERM_MF_ALL	GO:0016877~ligase activity, forming carbon-sulfur bonds	5
GOTERM_MF_ALL	GO:0043176~amine binding	6
GOTERM_BP_ALL	GO:0046395~carboxylic acid catabolic process	9
GOTERM_BP_ALL	GO:0016054~organic acid catabolic process	9
GOTERM_MF_ALL	GO:0008134~transcription factor binding	6
GOTERM_BP_ALL	GO:0006769~nicotinamide metabolic process	6
GOTERM_BP_ALL	GO:0046496~nicotinamide nucleotide metabolic process	6

GOTERM_BP_ALL	GO:0000160~two-component signal transduction system (p	18
GOTERM_BP_ALL	GO:0009110~vitamin biosynthetic process	8
GOTERM_CC_ALL	GO:0005773~vacuole	43
GOTERM_MF_ALL	GO:0008172~S-methyltransferase activity	3
GOTERM_BP_ALL	GO:0034637~cellular carbohydrate biosynthetic process	16
GOTERM_BP_ALL	GO:0019318~hexose metabolic process	14
GOTERM_BP_ALL	GO:0015994~chlorophyll metabolic process	6
GOTERM_BP_ALL	GO:0009820~alkaloid metabolic process	7
GOTERM_MF_ALL	GO:0080048~GDP-D-glucose phosphorylase activity	2
GOTERM_MF_ALL	GO:0001671~ATPase activator activity	2
GOTERM_MF_ALL	GO:0010475~galactose-1-phosphate guanylyltransferase (G	2
GOTERM_MF_ALL	GO:0004657~proline dehydrogenase activity	2
GOTERM_MF_ALL	GO:0008886~glyceraldehyde-3-phosphate dehydrogenase (	2
GOTERM_MF_ALL	GO:0047100~glyceraldehyde-3-phosphate dehydrogenase (	2
GOTERM_MF_ALL	GO:0004018~N6-(1,2-dicarboxyethyl)AMP AMP-lyase (fu	2
GOTERM_BP_ALL	GO:0046148~pigment biosynthetic process	9
GOTERM_MF_ALL	GO:0016620~oxidoreductase activity, acting on the aldehyd	6
GOTERM_BP_ALL	GO:0009407~toxin catabolic process	6
GOTERM_BP_ALL	GO:0009404~toxin metabolic process	6
GOTERM_BP_ALL	GO:0065007~biological regulation	243
GOTERM_MF_ALL	GO:0016758~transferase activity, transferring hexosyl grou	28
GOTERM_MF_ALL	GO:0016405~CoA-ligase activity	4
GOTERM_BP_ALL	GO:0006766~vitamin metabolic process	8
GOTERM_BP_ALL	GO:0044262~cellular carbohydrate metabolic process	36
GOTERM_MF_ALL	GO:0019842~vitamin binding	14
GOTERM_CC_ALL	GO:0009543~chloroplast thylakoid lumen	8
GOTERM_CC_ALL	GO:0031978~plastid thylakoid lumen	8
GOTERM_BP_ALL	GO:0015995~chlorophyll biosynthetic process	5
GOTERM_MF_ALL	GO:0003825~alpha,alpha-trehalose-phosphate synthase (U	3
GOTERM_MF_ALL	GO:0003712~transcription cofactor activity	5
GOTERM_CC_ALL	GO:0005829~cytosol	46
GOTERM_MF_ALL	GO:0008194~UDP-glycosyltransferase activity	18
GOTERM_BP_ALL	GO:0009739~response to gibberellin stimulus	11
GOTERM_BP_ALL	GO:0006720~isoprenoid metabolic process	12
GOTERM_MF_ALL	GO:0004364~glutathione transferase activity	6
GOTERM_MF_ALL	GO:0043167~ion binding	207
GOTERM_BP_ALL	GO:0019362~pyridine nucleotide metabolic process	6
GOTERM_MF_ALL	GO:0016878~acid-thiol ligase activity	4
GOTERM_BP_ALL	GO:0006007~glucose catabolic process	10
GOTERM_BP_ALL	GO:0006098~pentose-phosphate shunt	5
GOTERM_BP_ALL	GO:0016138~glycoside biosynthetic process	7
GOTERM_CC_ALL	GO:0043233~organelle lumen	43
GOTERM_CC_ALL	GO:0070013~intracellular organelle lumen	43
GOTERM_BP_ALL	GO:0046365~monosaccharide catabolic process	10
GOTERM_BP_ALL	GO:0019320~hexose catabolic process	10
GOTERM_MF_ALL	GO:0051119~sugar transmembrane transporter activity	10
GOTERM_BP_ALL	GO:0043603~cellular amide metabolic process	6

GOTERM_BP_ALL	GO:0006631~fatty acid metabolic process	17
GOTERM_MF_ALL	GO:0031406~carboxylic acid binding	9
GOTERM_MF_ALL	GO:0046983~protein dimerization activity	19
GOTERM_MF_ALL	GO:0043169~cation binding	206
GOTERM_MF_ALL	GO:0016722~oxidoreductase activity, oxidizing metal ions	3
GOTERM_BP_ALL	GO:0051186~cofactor metabolic process	21
GOTERM_CC_ALL	GO:0031974~membrane-enclosed lumen	43
GOTERM_BP_ALL	GO:0005986~sucrose biosynthetic process	3
GOTERM_BP_ALL	GO:0005996~monosaccharide metabolic process	15
GOTERM_MF_ALL	GO:0016829~lyase activity	26
GOTERM_MF_ALL	GO:0046872~metal ion binding	196
GOTERM_MF_ALL	GO:0016706~oxidoreductase activity, acting on paired don	8
GOTERM_BP_ALL	GO:0009808~lignin metabolic process	7
GOTERM_MF_ALL	GO:0070001~aspartic-type peptidase activity	8
GOTERM_MF_ALL	GO:0004190~aspartic-type endopeptidase activity	8
GOTERM_MF_ALL	GO:0016624~oxidoreductase activity, acting on the aldehyd	3
GOTERM_MF_ALL	GO:0045548~phenylalanine ammonia-lyase activity	2
GOTERM_MF_ALL	GO:0015193~L-proline transmembrane transporter activity	2
GOTERM_MF_ALL	GO:0016842~amidine-lyase activity	2
GOTERM_MF_ALL	GO:0003701~RNA polymerase I transcription factor activit	2
GOTERM_MF_ALL	GO:0004866~endopeptidase inhibitor activity	5
GOTERM_BP_ALL	GO:0016098~monoterpenoid metabolic process	2
GOTERM_BP_ALL	GO:0046621~negative regulation of organ growth	2
GOTERM_BP_ALL	GO:0016099~monoterpenoid biosynthetic process	2
GOTERM_BP_ALL	GO:0015824~proline transport	2
GOTERM_BP_ALL	GO:0006562~proline catabolic process	2
GOTERM_BP_ALL	GO:0009854~oxidative photosynthetic carbon pathway	2
GOTERM_BP_ALL	GO:0019853~L-ascorbic acid biosynthetic process	3
GOTERM_BP_ALL	GO:0019852~L-ascorbic acid metabolic process	3
GOTERM_CC_ALL	GO:0019898~extrinsic to membrane	17
GOTERM_MF_ALL	GO:0005351~sugar:hydrogen symporter activity	9
GOTERM_MF_ALL	GO:0015295~solute:hydrogen symporter activity	9
GOTERM_MF_ALL	GO:0005402~cation:sugar symporter activity	9
GOTERM_BP_ALL	GO:0042594~response to starvation	7
GOTERM_CC_ALL	GO:0005634~nucleus	191
GOTERM_BP_ALL	GO:0009615~response to virus	5
GOTERM_BP_ALL	GO:0008299~isoprenoid biosynthetic process	10
GOTERM_MF_ALL	GO:0004857~enzyme inhibitor activity	14
GOTERM_CC_ALL	GO:0031969~chloroplast membrane	7
GOTERM_MF_ALL	GO:0015144~carbohydrate transmembrane transporter activ	10
GOTERM_BP_ALL	GO:0016137~glycoside metabolic process	8
GOTERM_BP_ALL	GO:0006732~coenzyme metabolic process	15
GOTERM_BP_ALL	GO:0046164~alcohol catabolic process	10
GOTERM_MF_ALL	GO:0004564~beta-fructofuranosidase activity	3
GOTERM_BP_ALL	GO:0042440~pigment metabolic process	9
GOTERM_MF_ALL	GO:0005515~protein binding	209
GOTERM_BP_ALL	GO:0019439~aromatic compound catabolic process	4

GOTERM_MF_ALL	GO:0050551~myrcene synthase activity	2
GOTERM_MF_ALL	GO:0004645~phosphorylase activity	2
GOTERM_MF_ALL	GO:0080118~brassinosteroid sulfotransferase activity	2
GOTERM_MF_ALL	GO:0034768~(E)-beta-ocimene synthase activity	2
GOTERM_BP_ALL	GO:0010167~response to nitrate	3
GOTERM_BP_ALL	GO:0042981~regulation of apoptosis	3
GOTERM_BP_ALL	GO:0031324~negative regulation of cellular metabolic proc	8
GOTERM_BP_ALL	GO:0010196~nonphotochemical quenching	2
GOTERM_BP_ALL	GO:0009067~aspartate family amino acid biosynthetic proc	5
GOTERM_MF_ALL	GO:0051536~iron-sulfur cluster binding	8
GOTERM_MF_ALL	GO:0051540~metal cluster binding	8
GOTERM_MF_ALL	GO:0016671~oxidoreductase activity, acting on sulfur grou	4
GOTERM_BP_ALL	GO:0009058~biosynthetic process	242
GOTERM_BP_ALL	GO:0009806~lignan metabolic process	3
GOTERM_BP_ALL	GO:0009807~lignan biosynthetic process	3
GOTERM_BP_ALL	GO:0051707~response to other organism	51
GOTERM_BP_ALL	GO:0031408~oxylipin biosynthetic process	4
GOTERM_BP_ALL	GO:0009733~response to auxin stimulus	23
GOTERM_BP_ALL	GO:0009809~lignin biosynthetic process	5
GOTERM_BP_ALL	GO:0009553~embryo sac development	7
GOTERM_BP_ALL	GO:0019252~starch biosynthetic process	3
GOTERM_BP_ALL	GO:0006952~defense response	63
GOTERM_MF_ALL	GO:0031072~heat shock protein binding	10
GOTERM_MF_ALL	GO:0016841~ammonia-lyase activity	2
GOTERM_MF_ALL	GO:0047635~alanine-oxo-acid transaminase activity	2
GOTERM_MF_ALL	GO:0004021~L-alanine:2-oxoglutarate aminotransferase ac	2
GOTERM_MF_ALL	GO:0030410~nicotianamine synthase activity	2
GOTERM_MF_ALL	GO:0017057~6-phosphogluconolactonase activity	2
GOTERM_BP_ALL	GO:0006778~porphyrin metabolic process	6
GOTERM_MF_ALL	GO:0051537~2 iron, 2 sulfur cluster binding	4
GOTERM_BP_ALL	GO:0030417~nicotianamine metabolic process	2
GOTERM_BP_ALL	GO:0048571~long-day photoperiodism	2
GOTERM_BP_ALL	GO:0016103~diterpenoid catabolic process	2
GOTERM_BP_ALL	GO:0006537~glutamate biosynthetic process	2
GOTERM_BP_ALL	GO:0048574~long-day photoperiodism, flowering	2
GOTERM_BP_ALL	GO:0045487~gibberellin catabolic process	2
GOTERM_BP_ALL	GO:0030418~nicotianamine biosynthetic process	2
GOTERM_CC_ALL	GO:0030095~chloroplast photosystem II	3
GOTERM_BP_ALL	GO:0046942~carboxylic acid transport	7
GOTERM_BP_ALL	GO:0015849~organic acid transport	7
GOTERM_BP_ALL	GO:0033013~tetrapyrrole metabolic process	6
GOTERM_BP_ALL	GO:0016126~sterol biosynthetic process	4
GOTERM_BP_ALL	GO:0006779~porphyrin biosynthetic process	5
GOTERM_BP_ALL	GO:0032268~regulation of cellular protein metabolic proce	5
GOTERM_MF_ALL	GO:0005509~calcium ion binding	26
GOTERM_BP_ALL	GO:0019319~hexose biosynthetic process	3
GOTERM_BP_ALL	GO:0009269~response to desiccation	3

GOTERM_MF_ALL	GO:0004497~monooxygenase activity	21
GOTERM_BP_ALL	GO:0006096~glycolysis	7
GOTERM_BP_ALL	GO:0009909~regulation of flower development	8
GOTERM_CC_ALL	GO:0009505~plant-type cell wall	18
GOTERM_MF_ALL	GO:0019203~carbohydrate phosphatase activity	3
GOTERM_BP_ALL	GO:0010017~red or far red light signaling pathway	6
GOTERM_MF_ALL	GO:0016757~transferase activity, transferring glycosyl grou	31
GOTERM_BP_ALL	GO:0046364~monosaccharide biosynthetic process	3
GOTERM_BP_ALL	GO:0006544~glycine metabolic process	3
GOTERM_MF_ALL	GO:0009011~starch synthase activity	2
GOTERM_MF_ALL	GO:0016157~sucrose synthase activity	2
GOTERM_MF_ALL	GO:0004739~pyruvate dehydrogenase (acetyl-transferring)	2
GOTERM_MF_ALL	GO:0045430~chalcone isomerase activity	2
GOTERM_MF_ALL	GO:0004738~pyruvate dehydrogenase activity	2
GOTERM_MF_ALL	GO:0070568~guanylyltransferase activity	2
GOTERM_BP_ALL	GO:0006972~hyperosmotic response	5
GOTERM_BP_ALL	GO:0009736~cytokinin mediated signaling	5
GOTERM_MF_ALL	GO:0008168~methyltransferase activity	17
GOTERM_BP_ALL	GO:0031667~response to nutrient levels	7
GOTERM_BP_ALL	GO:0031407~oxylipin metabolic process	4
GOTERM_BP_ALL	GO:0046620~regulation of organ growth	2
GOTERM_BP_ALL	GO:0015804~neutral amino acid transport	2
GOTERM_BP_ALL	GO:0006559~L-phenylalanine catabolic process	2
GOTERM_MF_ALL	GO:0016741~transferase activity, transferring one-carbon g	17
GOTERM_BP_ALL	GO:0033014~tetrapyrrole biosynthetic process	5
GOTERM_BP_ALL	GO:0006865~amino acid transport	6
GOTERM_BP_ALL	GO:0018130~heterocycle biosynthetic process	10
GOTERM_BP_ALL	GO:0009631~cold acclimation	3
GOTERM_BP_ALL	GO:0016125~sterol metabolic process	4
GOTERM_BP_ALL	GO:0044249~cellular biosynthetic process	228
GOTERM_MF_ALL	GO:0008080~N-acetyltransferase activity	5
GOTERM_BP_ALL	GO:0008643~carbohydrate transport	7
GOTERM_BP_ALL	GO:0015837~amine transport	6
GOTERM_BP_ALL	GO:0008202~steroid metabolic process	6
GOTERM_CC_ALL	GO:0009706~chloroplast inner membrane	4
GOTERM_MF_ALL	GO:0005507~copper ion binding	12
GOTERM_MF_ALL	GO:0016248~channel inhibitor activity	2
GOTERM_MF_ALL	GO:0045549~9-cis-epoxycarotenoid dioxygenase activity	2
GOTERM_MF_ALL	GO:0008200~ion channel inhibitor activity	2
GOTERM_MF_ALL	GO:0003913~DNA photolyase activity	2
GOTERM_MF_ALL	GO:0004611~phosphoenolpyruvate carboxykinase activity	2
GOTERM_MF_ALL	GO:0010436~carotenoid dioxygenase activity	2
GOTERM_MF_ALL	GO:0015037~peptide disulfide oxidoreductase activity	2
GOTERM_MF_ALL	GO:0015038~glutathione disulfide oxidoreductase activity	2
GOTERM_MF_ALL	GO:0060590~ATPase regulator activity	2
GOTERM_MF_ALL	GO:0016984~ribulose-bisphosphate carboxylase activity	2
GOTERM_BP_ALL	GO:0009685~gibberellin metabolic process	3

GOTERM_BP_ALL	GO:0005992~trehalose biosynthetic process	3
GOTERM_BP_ALL	GO:0033554~cellular response to stress	27
GOTERM_BP_ALL	GO:0044237~cellular metabolic process	435
GOTERM_BP_ALL	GO:0051644~plastid localization	2
GOTERM_BP_ALL	GO:0006094~gluconeogenesis	2
GOTERM_BP_ALL	GO:0006636~unsaturated fatty acid biosynthetic process	2
GOTERM_BP_ALL	GO:0009902~chloroplast relocation	2
GOTERM_BP_ALL	GO:0051667~establishment of plastid localization	2
GOTERM_BP_ALL	GO:0009309~amine biosynthetic process	14
GOTERM_MF_ALL	GO:0016717~oxidoreductase activity, acting on paired don	3
GOTERM_BP_ALL	GO:0048582~positive regulation of post-embryonic develop	4
GOTERM_BP_ALL	GO:0046165~alcohol biosynthetic process	4
GOTERM_MF_ALL	GO:0046982~protein heterodimerization activity	4
GOTERM_CC_ALL	GO:0042170~plastid membrane	7
GOTERM_BP_ALL	GO:0051704~multi-organism process	58
GOTERM_BP_ALL	GO:0016101~diterpenoid metabolic process	3
GOTERM_BP_ALL	GO:0051716~cellular response to stimulus	58
GOTERM_MF_ALL	GO:0080043~quercetin 3-O-glucosyltransferase activity	3
GOTERM_MF_ALL	GO:0022838~substrate specific channel activity	9
GOTERM_MF_ALL	GO:0080046~quercetin 4'-O-glucosyltransferase activity	2
GOTERM_MF_ALL	GO:0004337~geranyltranstransferase activity	2
GOTERM_MF_ALL	GO:0016247~channel regulator activity	2
GOTERM_BP_ALL	GO:0045941~positive regulation of transcription	5
GOTERM_BP_ALL	GO:0048523~negative regulation of cellular process	13
GOTERM_MF_ALL	GO:0015294~solute:cation symporter activity	9
GOTERM_MF_ALL	GO:0015267~channel activity	9
GOTERM_MF_ALL	GO:0022803~passive transmembrane transporter activity	9
GOTERM_BP_ALL	GO:0019751~polyol metabolic process	4
GOTERM_BP_ALL	GO:0005991~trehalose metabolic process	3
GOTERM_BP_ALL	GO:0017004~cytochrome complex assembly	3
GOTERM_BP_ALL	GO:0006744~ubiquinone biosynthetic process	2
GOTERM_BP_ALL	GO:0009772~photosynthetic electron transport in photosyst	2
GOTERM_BP_ALL	GO:0045926~negative regulation of growth	2
GOTERM_BP_ALL	GO:0000184~nuclear-transcribed mRNA catabolic process,	2
GOTERM_BP_ALL	GO:0009074~aromatic amino acid family catabolic process	2
GOTERM_BP_ALL	GO:0006743~ubiquinone metabolic process	2
GOTERM_BP_ALL	GO:0051248~negative regulation of protein metabolic proc	2
GOTERM_BP_ALL	GO:0031399~regulation of protein modification process	2
GOTERM_BP_ALL	GO:0006995~cellular response to nitrogen starvation	2
GOTERM_BP_ALL	GO:0032269~negative regulation of cellular protein metabo	2
GOTERM_MF_ALL	GO:0016410~N-acyltransferase activity	5
GOTERM_BP_ALL	GO:0010628~positive regulation of gene expression	5
GOTERM_CC_ALL	GO:0044421~extracellular region part	4
GOTERM_BP_ALL	GO:0007568~aging	6
GOTERM_BP_ALL	GO:0051188~cofactor biosynthetic process	11
GOTERM_MF_ALL	GO:0005102~receptor binding	4
GOTERM_BP_ALL	GO:0042401~biogenic amine biosynthetic process	4

GOTERM_BP_ALL	GO:0005975~carbohydrate metabolic process	56
GOTERM_MF_ALL	GO:0015293~symporter activity	10
GOTERM_BP_ALL	GO:0006413~translational initiation	6
GOTERM_MF_ALL	GO:0016645~oxidoreductase activity, acting on the CH-NH	3
GOTERM_MF_ALL	GO:0015179~L-amino acid transmembrane transporter acti	2
GOTERM_MF_ALL	GO:0004332~fructose-bisphosphate aldolase activity	2
GOTERM_MF_ALL	GO:0031177~phosphopantetheine binding	2
GOTERM_MF_ALL	GO:0015175~neutral amino acid transmembrane transporte	2
GOTERM_MF_ALL	GO:0004602~glutathione peroxidase activity	2
GOTERM_MF_ALL	GO:0051087~chaperone binding	2
GOTERM_BP_ALL	GO:0008152~metabolic process	547
GOTERM_BP_ALL	GO:0044255~cellular lipid metabolic process	29
GOTERM_BP_ALL	GO:0006753~nucleoside phosphate metabolic process	15
GOTERM_BP_ALL	GO:0009117~nucleotide metabolic process	15
GOTERM_BP_ALL	GO:0033559~unsaturated fatty acid metabolic process	2
GOTERM_BP_ALL	GO:0006560~proline metabolic process	2
GOTERM_BP_ALL	GO:0055086~nucleobase, nucleoside and nucleotide metab	17
GOTERM_BP_ALL	GO:0044275~cellular carbohydrate catabolic process	10
GOTERM_BP_ALL	GO:0009086~methionine biosynthetic process	3
GOTERM_BP_ALL	GO:0016117~carotenoid biosynthetic process	3
GOTERM_BP_ALL	GO:0016109~tetraterpenoid biosynthetic process	3
GOTERM_BP_ALL	GO:0009991~response to extracellular stimulus	7
GOTERM_BP_ALL	GO:0009624~response to nematode	5
GOTERM_BP_ALL	GO:0006576~biogenic amine metabolic process	5
GOTERM_BP_ALL	GO:0042538~hyperosmotic salinity response	4
GOTERM_BP_ALL	GO:0009853~photorespiration	4
GOTERM_BP_ALL	GO:0009561~megagametogenesis	4
GOTERM_BP_ALL	GO:0044106~cellular amine metabolic process	25
GOTERM_BP_ALL	GO:0009064~glutamine family amino acid metabolic proce	5
GOTERM_BP_ALL	GO:0051094~positive regulation of developmental process	4
GOTERM_BP_ALL	GO:0048443~stamen development	4
GOTERM_BP_ALL	GO:0048466~androecium development	4
GOTERM_MF_ALL	GO:0008083~growth factor activity	2
GOTERM_BP_ALL	GO:0006629~lipid metabolic process	45
GOTERM_BP_ALL	GO:0042742~defense response to bacterium	12
GOTERM_BP_ALL	GO:0006188~IMP biosynthetic process	2
GOTERM_BP_ALL	GO:0008300~isoprenoid catabolic process	2
GOTERM_BP_ALL	GO:0016115~terpenoid catabolic process	2
GOTERM_BP_ALL	GO:0051656~establishment of organelle localization	2
GOTERM_BP_ALL	GO:0046040~IMP metabolic process	2
GOTERM_BP_ALL	GO:0009065~glutamine family amino acid catabolic proces	2
GOTERM_BP_ALL	GO:0009267~cellular response to starvation	5
GOTERM_MF_ALL	GO:0042802~identical protein binding	11
GOTERM_BP_ALL	GO:0048519~negative regulation of biological process	22
GOTERM_MF_ALL	GO:0003993~acid phosphatase activity	4
GOTERM_BP_ALL	GO:0006694~steroid biosynthetic process	4
GOTERM_BP_ALL	GO:0043067~regulation of programmed cell death	3

GOTERM_CC_ALL	GO:0005886~plasma membrane	121
GOTERM_BP_ALL	GO:0044042~glucan metabolic process	10
GOTERM_BP_ALL	GO:0031325~positive regulation of cellular metabolic proc	6
GOTERM_MF_ALL	GO:0046910~pectinesterase inhibitor activity	5
GOTERM_MF_ALL	GO:0016774~phosphotransferase activity, carboxyl group a	2
GOTERM_MF_ALL	GO:0016880~acid-ammonia (or amide) ligase activity	2
GOTERM_MF_ALL	GO:0016872~intramolecular lyase activity	2
GOTERM_MF_ALL	GO:0016211~ammonia ligase activity	2
GOTERM_MF_ALL	GO:0016744~transferase activity, transferring aldehyde or k	2
GOTERM_BP_ALL	GO:0045935~positive regulation of nucleobase, nucleoside,	5
GOTERM_BP_ALL	GO:0051173~positive regulation of nitrogen compound met	5
GOTERM_BP_ALL	GO:0010557~positive regulation of macromolecule biosynt	5
GOTERM_CC_ALL	GO:0016604~nuclear body	4
GOTERM_BP_ALL	GO:0009893~positive regulation of metabolic process	6
GOTERM_MF_ALL	GO:0015171~amino acid transmembrane transporter activit	5
GOTERM_BP_ALL	GO:0043193~positive regulation of gene-specific transcript	2
GOTERM_MF_ALL	GO:0016638~oxidoreductase activity, acting on the CH-NH	4
GOTERM_BP_ALL	GO:0009640~photomorphogenesis	4
GOTERM_MF_ALL	GO:0003743~translation initiation factor activity	7
GOTERM_CC_ALL	GO:0009528~plastid inner membrane	4
GOTERM_BP_ALL	GO:0009617~response to bacterium	15
GOTERM_MF_ALL	GO:0016838~carbon-oxygen lyase activity, acting on phosp	2
GOTERM_MF_ALL	GO:0009815~1-aminocyclopropane-1-carboxylate oxidase	2
GOTERM_MF_ALL	GO:0045735~nutrient reservoir activity	5
GOTERM_BP_ALL	GO:0043094~cellular metabolic compound salvage	5
GOTERM_BP_ALL	GO:0009911~positive regulation of flower development	3
GOTERM_BP_ALL	GO:0010941~regulation of cell death	3
GOTERM_CC_ALL	GO:0005730~nucleolus	19
GOTERM_MF_ALL	GO:0046961~proton-transporting ATPase activity, rotation	3
GOTERM_MF_ALL	GO:0016782~transferase activity, transferring sulfur-contai	3
GOTERM_MF_ALL	GO:0016651~oxidoreductase activity, acting on NADH or	6
GOTERM_MF_ALL	GO:0008270~zinc ion binding	90
GOTERM_BP_ALL	GO:0009892~negative regulation of metabolic process	12
GOTERM_MF_ALL	GO:0016407~acetyltransferase activity	5
GOTERM_MF_ALL	GO:0008889~glycerophosphodiester phosphodiesterase acti	2
GOTERM_CC_ALL	GO:0016607~nuclear speck	3
GOTERM_BP_ALL	GO:0044271~nitrogen compound biosynthetic process	29
GOTERM_BP_ALL	GO:0043085~positive regulation of catalytic activity	3
GOTERM_BP_ALL	GO:0010149~senescence	3
GOTERM_BP_ALL	GO:0009891~positive regulation of biosynthetic process	5
GOTERM_BP_ALL	GO:0031328~positive regulation of cellular biosynthetic pr	5
GOTERM_BP_ALL	GO:0043289~apocarotenoid biosynthetic process	2
GOTERM_BP_ALL	GO:0009688~abscisic acid biosynthetic process	2
GOTERM_BP_ALL	GO:0000956~nuclear-transcribed mRNA catabolic process	2
GOTERM_BP_ALL	GO:0043562~cellular response to nitrogen levels	2
GOTERM_BP_ALL	GO:0015931~nucleobase, nucleoside, nucleotide and nuclei	4
GOTERM_BP_ALL	GO:0000097~sulfur amino acid biosynthetic process	4

GOTERM_CC_ALL	GO:0005840~ribosome	26
GOTERM_BP_ALL	GO:0006073~cellular glucan metabolic process	8
GOTERM_BP_ALL	GO:0031669~cellular response to nutrient levels	5
GOTERM_BP_ALL	GO:0010604~positive regulation of macromolecule metabo	5
GOTERM_MF_ALL	GO:0030234~enzyme regulator activity	20
GOTERM_MF_ALL	GO:0005215~transporter activity	69
GOTERM_BP_ALL	GO:0016116~carotenoid metabolic process	3
GOTERM_BP_ALL	GO:0016108~tetraterpenoid metabolic process	3
GOTERM_BP_ALL	GO:0044093~positive regulation of molecular function	3
GOTERM_MF_ALL	GO:0008171~O-methyltransferase activity	3
GOTERM_MF_ALL	GO:0016762~xyloglucan:xyloglucosyl transferase activity	3
GOTERM_MF_ALL	GO:0000287~magnesium ion binding	19
GOTERM_BP_ALL	GO:0006520~cellular amino acid metabolic process	22
GOTERM_MF_ALL	GO:0030614~oxidoreductase activity, acting on phosphorus	2
GOTERM_MF_ALL	GO:0008794~arsenate reductase (glutaredoxin) activity	2
GOTERM_MF_ALL	GO:0030613~oxidoreductase activity, acting on phosphorus	2
GOTERM_MF_ALL	GO:0004372~glycine hydroxymethyltransferase activity	2
GOTERM_MF_ALL	GO:0016899~oxidoreductase activity, acting on the CH-OH	2
GOTERM_BP_ALL	GO:0048580~regulation of post-embryonic development	9
GOTERM_BP_ALL	GO:0000271~polysaccharide biosynthetic process	7
GOTERM_MF_ALL	GO:0005275~amine transmembrane transporter activity	5
GOTERM_BP_ALL	GO:0034641~cellular nitrogen compound metabolic proces	162
GOTERM_BP_ALL	GO:0005976~polysaccharide metabolic process	13
GOTERM_BP_ALL	GO:0019722~calcium-mediated signaling	2
GOTERM_BP_ALL	GO:0006558~L-phenylalanine metabolic process	2
GOTERM_BP_ALL	GO:0009646~response to absence of light	2
GOTERM_BP_ALL	GO:0051028~mRNA transport	2
GOTERM_MF_ALL	GO:0008135~translation factor activity, nucleic acid bindin	9
GOTERM_BP_ALL	GO:0015698~inorganic anion transport	4
GOTERM_BP_ALL	GO:0009066~aspartate family amino acid metabolic proces	5
GOTERM_BP_ALL	GO:0006119~oxidative phosphorylation	6
GOTERM_BP_ALL	GO:0009108~coenzyme biosynthetic process	6
GOTERM_BP_ALL	GO:0065008~regulation of biological quality	36
GOTERM_BP_ALL	GO:0008652~cellular amino acid biosynthetic process	11
GOTERM_MF_ALL	GO:0009881~photoreceptor activity	2
GOTERM_MF_ALL	GO:0030611~arsenate reductase activity	2
GOTERM_MF_ALL	GO:0045551~cinnamyl-alcohol dehydrogenase activity	2
GOTERM_BP_ALL	GO:0005982~starch metabolic process	3
GOTERM_MF_ALL	GO:0051539~4 iron, 4 sulfur cluster binding	3
GOTERM_BP_ALL	GO:0016052~carbohydrate catabolic process	12
GOTERM_BP_ALL	GO:0006402~mRNA catabolic process	2
GOTERM_BP_ALL	GO:0051640~organelle localization	2
GOTERM_BP_ALL	GO:0000041~transition metal ion transport	5
GOTERM_BP_ALL	GO:0009867~jasmonic acid mediated signaling pathway	3
GOTERM_CC_ALL	GO:0005615~extracellular space	2
GOTERM_BP_ALL	GO:0009308~amine metabolic process	27
GOTERM_MF_ALL	GO:0008559~xenobiotic-transporting ATPase activity	2

GOTERM_MF_ALL	GO:0004805~trehalose-phosphatase activity	2
GOTERM_MF_ALL	GO:0005315~inorganic phosphate transmembrane transport	2
GOTERM_MF_ALL	GO:0042910~xenobiotic transporter activity	2
GOTERM_BP_ALL	GO:0006084~acetyl-CoA metabolic process	4
GOTERM_CC_ALL	GO:0005643~nuclear pore	3
GOTERM_MF_ALL	GO:0019825~oxygen binding	13
GOTERM_BP_ALL	GO:0009873~ethylene mediated signaling pathway	10
GOTERM_BP_ALL	GO:0044248~cellular catabolic process	51
GOTERM_BP_ALL	GO:0051236~establishment of RNA localization	2
GOTERM_BP_ALL	GO:0001666~response to hypoxia	2
GOTERM_BP_ALL	GO:0010025~wax biosynthetic process	2
GOTERM_BP_ALL	GO:0009127~purine nucleoside monophosphate biosyntheti	2
GOTERM_BP_ALL	GO:0009126~purine nucleoside monophosphate metabolic	2
GOTERM_BP_ALL	GO:0006817~phosphate transport	2
GOTERM_BP_ALL	GO:0050658~RNA transport	2
GOTERM_BP_ALL	GO:0006342~chromatin silencing	2
GOTERM_BP_ALL	GO:0032583~regulation of gene-specific transcription	2
GOTERM_BP_ALL	GO:0009167~purine ribonucleoside monophosphate metabo	2
GOTERM_BP_ALL	GO:0006403~RNA localization	2
GOTERM_BP_ALL	GO:0070482~response to oxygen levels	2
GOTERM_BP_ALL	GO:0043624~cellular protein complex disassembly	2
GOTERM_BP_ALL	GO:0050657~nucleic acid transport	2
GOTERM_BP_ALL	GO:0009168~purine ribonucleoside monophosphate biosyn	2
GOTERM_BP_ALL	GO:0034623~cellular macromolecular complex disassembl	2
GOTERM_MF_ALL	GO:0016740~transferase activity	155
GOTERM_BP_ALL	GO:0009910~negative regulation of flower development	3
GOTERM_BP_ALL	GO:0035295~tube development	5
GOTERM_BP_ALL	GO:0009250~glucan biosynthetic process	5
GOTERM_BP_ALL	GO:0048868~pollen tube development	5
GOTERM_BP_ALL	GO:0046483~heterocycle metabolic process	24
GOTERM_CC_ALL	GO:0005665~DNA-directed RNA polymerase II, core comp	2
GOTERM_MF_ALL	GO:0030246~carbohydrate binding	12
GOTERM_CC_ALL	GO:0019866~organelle inner membrane	13
GOTERM_MF_ALL	GO:0004551~nucleotide diphosphatase activity	2
GOTERM_MF_ALL	GO:0080044~quercetin 7-O-glucosyltransferase activity	2
GOTERM_MF_ALL	GO:0008471~laccase activity	2
GOTERM_MF_ALL	GO:0033293~monocarboxylic acid binding	2
GOTERM_BP_ALL	GO:0042214~terpene metabolic process	3
GOTERM_BP_ALL	GO:0048518~positive regulation of biological process	12
GOTERM_BP_ALL	GO:0006807~nitrogen compound metabolic process	165
GOTERM_CC_ALL	GO:0031981~nuclear lumen	25
GOTERM_BP_ALL	GO:0044264~cellular polysaccharide metabolic process	9
GOTERM_BP_ALL	GO:0006536~glutamate metabolic process	2
GOTERM_BP_ALL	GO:0055072~iron ion homeostasis	2
GOTERM_BP_ALL	GO:0032984~macromolecular complex disassembly	2
GOTERM_BP_ALL	GO:0016106~sesquiterpenoid biosynthetic process	2
GOTERM_BP_ALL	GO:0010166~wax metabolic process	2

GOTERM_BP_ALL	GO:0006020~inositol metabolic process	2
GOTERM_BP_ALL	GO:0043241~protein complex disassembly	2
GOTERM_BP_ALL	GO:0046271~phenylpropanoid catabolic process	2
GOTERM_BP_ALL	GO:0051347~positive regulation of transferase activity	2
GOTERM_BP_ALL	GO:0051762~sesquiterpene biosynthetic process	2
GOTERM_BP_ALL	GO:0046274~lignin catabolic process	2
GOTERM_CC_ALL	GO:0022626~cytosolic ribosome	17
GOTERM_MF_ALL	GO:0016779~nucleotidyltransferase activity	10
GOTERM_MF_ALL	GO:0070279~vitamin B6 binding	7
GOTERM_MF_ALL	GO:0030170~pyridoxal phosphate binding	7
GOTERM_CC_ALL	GO:0005774~vacuolar membrane	5
GOTERM_CC_ALL	GO:0045259~proton-transporting ATP synthase complex	3
GOTERM_MF_ALL	GO:0030145~manganese ion binding	14
GOTERM_CC_ALL	GO:0009654~oxygen evolving complex	2
GOTERM_BP_ALL	GO:0009908~flower development	13
GOTERM_MF_ALL	GO:0015239~multidrug transporter activity	2
GOTERM_BP_ALL	GO:0055085~transmembrane transport	12
GOTERM_CC_ALL	GO:0009705~plant-type vacuole membrane	3
GOTERM_MF_ALL	GO:0022804~active transmembrane transporter activity	32
GOTERM_BP_ALL	GO:0009072~aromatic amino acid family metabolic proces	4
GOTERM_BP_ALL	GO:0015985~energy coupled proton transport, down electr	4
GOTERM_BP_ALL	GO:0015986~ATP synthesis coupled proton transport	4
GOTERM_BP_ALL	GO:0042375~quinone cofactor metabolic process	2
GOTERM_BP_ALL	GO:0006595~polyamine metabolic process	2
GOTERM_BP_ALL	GO:0045426~quinone cofactor biosynthetic process	2
GOTERM_BP_ALL	GO:0046688~response to copper ion	2
GOTERM_BP_ALL	GO:0045893~positive regulation of transcription, DNA-dep	2
GOTERM_BP_ALL	GO:0010197~polar nucleus fusion	2
GOTERM_MF_ALL	GO:0016679~oxidoreductase activity, acting on diphenols a	3
GOTERM_MF_ALL	GO:0015103~inorganic anion transmembrane transporter ac	4
GOTERM_MF_ALL	GO:0008081~phosphoric diester hydrolase activity	4
GOTERM_CC_ALL	GO:0046930~pore complex	3
GOTERM_CC_ALL	GO:0044437~vacuolar part	5
GOTERM_BP_ALL	GO:0016441~posttranscriptional gene silencing	6
GOTERM_MF_ALL	GO:0005496~steroid binding	2
GOTERM_MF_ALL	GO:0015114~phosphate transmembrane transporter activity	2
GOTERM_MF_ALL	GO:0004180~carboxypeptidase activity	4
GOTERM_MF_ALL	GO:0019200~carbohydrate kinase activity	3
GOTERM_MF_ALL	GO:0005342~organic acid transmembrane transporter activ	5
GOTERM_MF_ALL	GO:0046943~carboxylic acid transmembrane transporter ac	5
GOTERM_MF_ALL	GO:0015238~drug transporter activity	5
GOTERM_BP_ALL	GO:0031668~cellular response to extracellular stimulus	5
GOTERM_BP_ALL	GO:0009687~abscisic acid metabolic process	2
GOTERM_BP_ALL	GO:0051607~defense response to virus	2
GOTERM_BP_ALL	GO:0009559~embryo sac central cell differentiation	2
GOTERM_BP_ALL	GO:0006401~RNA catabolic process	2
GOTERM_BP_ALL	GO:0048284~organelle fusion	2

GOTERM_BP_ALL	GO:0043288~apocarotenoid metabolic process	2
GOTERM_BP_ALL	GO:0006090~pyruvate metabolic process	2
GOTERM_BP_ALL	GO:0000741~karyogamy	2
GOTERM_MF_ALL	GO:0003899~DNA-directed RNA polymerase activity	4
GOTERM_MF_ALL	GO:0008757~S-adenosylmethionine-dependent methyltrans	7
GOTERM_BP_ALL	GO:0006606~protein import into nucleus	3
GOTERM_CC_ALL	GO:0016469~proton-transporting two-sector ATPase compl	4
GOTERM_MF_ALL	GO:0008146~sulfotransferase activity	2
GOTERM_MF_ALL	GO:0015291~secondary active transmembrane transporter a	16
GOTERM_BP_ALL	GO:0033692~cellular polysaccharide biosynthetic process	6
GOTERM_BP_ALL	GO:0006351~transcription, DNA-dependent	5
GOTERM_BP_ALL	GO:0032879~regulation of localization	2
GOTERM_BP_ALL	GO:0051170~nuclear import	3
GOTERM_BP_ALL	GO:0048437~floral organ development	7
GOTERM_BP_ALL	GO:0043623~cellular protein complex assembly	7
GOTERM_MF_ALL	GO:0004707~MAP kinase activity	2
GOTERM_CC_ALL	GO:0005834~heterotrimeric G-protein complex	4
GOTERM_CC_ALL	GO:0005635~nuclear envelope	4
GOTERM_CC_ALL	GO:0046658~anchored to plasma membrane	4
GOTERM_BP_ALL	GO:0010608~posttranscriptional regulation of gene express	8
GOTERM_MF_ALL	GO:0016846~carbon-sulfur lyase activity	3
GOTERM_CC_ALL	GO:0005739~mitochondrion	69
GOTERM_BP_ALL	GO:0032774~RNA biosynthetic process	5
GOTERM_BP_ALL	GO:0006071~glycerol metabolic process	2
GOTERM_BP_ALL	GO:0010565~regulation of cellular ketone metabolic proces	2
GOTERM_BP_ALL	GO:0045814~negative regulation of gene expression, epige	2
GOTERM_BP_ALL	GO:0051254~positive regulation of RNA metabolic process	2
GOTERM_BP_ALL	GO:0009636~response to toxin	2
GOTERM_BP_ALL	GO:0022411~cellular component disassembly	2
GOTERM_MF_ALL	GO:0030599~pectinesterase activity	8
GOTERM_BP_ALL	GO:0048438~floral whorl development	6
GOTERM_BP_ALL	GO:0006461~protein complex assembly	10
GOTERM_BP_ALL	GO:0070271~protein complex biogenesis	10
GOTERM_BP_ALL	GO:0009620~response to fungus	23
GOTERM_MF_ALL	GO:0008237~metallopeptidase activity	5
GOTERM_MF_ALL	GO:0005528~FK506 binding	2
GOTERM_MF_ALL	GO:0016832~aldehyde-lyase activity	2
GOTERM_MF_ALL	GO:0005527~macrolide binding	2
GOTERM_BP_ALL	GO:0034621~cellular macromolecular complex subunit org	12
GOTERM_BP_ALL	GO:0006099~tricarboxylic acid cycle	3
GOTERM_BP_ALL	GO:0046356~acetyl-CoA catabolic process	3
GOTERM_BP_ALL	GO:0034504~protein localization in nucleus	3
GOTERM_BP_ALL	GO:0044272~sulfur compound biosynthetic process	6
GOTERM_CC_ALL	GO:0019897~extrinsic to plasma membrane	4
GOTERM_BP_ALL	GO:0034220~ion transmembrane transport	4
GOTERM_BP_ALL	GO:0048229~gametophyte development	10
GOTERM_BP_ALL	GO:0019400~alditol metabolic process	2

GOTERM_BP_ALL	GO:0019932~second-messenger-mediated signaling	2
GOTERM_MF_ALL	GO:0004428~inositol or phosphatidylinositol kinase activit	3
GOTERM_CC_ALL	GO:0005788~endoplasmic reticulum lumen	2
GOTERM_BP_ALL	GO:0007602~phototransduction	3
GOTERM_BP_ALL	GO:0009583~detection of light stimulus	3
GOTERM_BP_ALL	GO:0009585~red, far-red light phototransduction	3
GOTERM_BP_ALL	GO:0010015~root morphogenesis	5
GOTERM_MF_ALL	GO:0034062~RNA polymerase activity	4
GOTERM_BP_ALL	GO:0043933~macromolecular complex subunit organizatio	15
GOTERM_MF_ALL	GO:0046915~transition metal ion transmembrane transporte	3
GOTERM_BP_ALL	GO:0051604~protein maturation	2
GOTERM_BP_ALL	GO:0009567~double fertilization forming a zygote and end	2
GOTERM_BP_ALL	GO:0042775~mitochondrial ATP synthesis coupled electro	2
GOTERM_BP_ALL	GO:0045892~negative regulation of transcription, DNA-de	2
GOTERM_BP_ALL	GO:0006714~sesquiterpenoid metabolic process	2
GOTERM_BP_ALL	GO:0009695~jasmonic acid biosynthetic process	2
GOTERM_BP_ALL	GO:0002252~immune effector process	2
GOTERM_BP_ALL	GO:0009156~ribonucleoside monophosphate biosynthetic p	2
GOTERM_BP_ALL	GO:0051253~negative regulation of RNA metabolic proces	2
GOTERM_BP_ALL	GO:0051761~sesquiterpene metabolic process	2
GOTERM_BP_ALL	GO:0006863~purine transport	2
GOTERM_CC_ALL	GO:0042579~microbody	9
GOTERM_CC_ALL	GO:0005777~peroxisome	9
GOTERM_BP_ALL	GO:0006790~sulfur metabolic process	9
GOTERM_BP_ALL	GO:0006730~one-carbon metabolic process	6
GOTERM_BP_ALL	GO:0051606~detection of stimulus	4
GOTERM_BP_ALL	GO:0006913~nucleocytoplasmic transport	4
GOTERM_BP_ALL	GO:0051169~nuclear transport	4
GOTERM_MF_ALL	GO:0008144~drug binding	2
GOTERM_MF_ALL	GO:0004867~serine-type endopeptidase inhibitor activity	2
GOTERM_CC_ALL	GO:0000325~plant-type vacuole	4
GOTERM_BP_ALL	GO:0010558~negative regulation of macromolecule biosynt	4
GOTERM_BP_ALL	GO:0030154~cell differentiation	16
GOTERM_BP_ALL	GO:0009566~fertilization	2
GOTERM_BP_ALL	GO:0006825~copper ion transport	2
GOTERM_BP_ALL	GO:0015851~nucleobase transport	2
GOTERM_BP_ALL	GO:0009161~ribonucleoside monophosphate metabolic pro	2
GOTERM_CC_ALL	GO:0031012~extracellular matrix	2
GOTERM_MF_ALL	GO:0004521~endoribonuclease activity	3
GOTERM_MF_ALL	GO:0019829~cation-transporting ATPase activity	3
GOTERM_CC_ALL	GO:0044464~cell part	875
GOTERM_CC_ALL	GO:0005623~cell	875
GOTERM_BP_ALL	GO:0035195~gene silencing by miRNA	4
GOTERM_BP_ALL	GO:0015833~peptide transport	4
GOTERM_BP_ALL	GO:0006857~oligopeptide transport	4
GOTERM_MF_ALL	GO:0005345~purine transmembrane transporter activity	2
GOTERM_MF_ALL	GO:0016847~1-aminocyclopropane-1-carboxylate synthase	2

GOTERM_MF_ALL	GO:0005085~guanyl-nucleotide exchange factor activity	2
GOTERM_MF_ALL	GO:0004673~protein histidine kinase activity	2
GOTERM_MF_ALL	GO:0016775~phosphotransferase activity, nitrogenous grou	2
GOTERM_MF_ALL	GO:0000155~two-component sensor activity	2
GOTERM_BP_ALL	GO:0009109~coenzyme catabolic process	3
GOTERM_BP_ALL	GO:0040008~regulation of growth	3
GOTERM_BP_ALL	GO:0021700~developmental maturation	3
GOTERM_BP_ALL	GO:0009755~hormone-mediated signaling	22
GOTERM_BP_ALL	GO:0032870~cellular response to hormone stimulus	22
GOTERM_BP_ALL	GO:0035194~posttranscriptional gene silencing by RNA	5
GOTERM_MF_ALL	GO:0004222~metalloendopeptidase activity	3
GOTERM_BP_ALL	GO:0048864~stem cell development	2
GOTERM_BP_ALL	GO:0006826~iron ion transport	2
GOTERM_BP_ALL	GO:0006997~nucleus organization	2
GOTERM_BP_ALL	GO:0042278~purine nucleoside metabolic process	2
GOTERM_BP_ALL	GO:0046128~purine ribonucleoside metabolic process	2
GOTERM_BP_ALL	GO:0048653~anther development	2
GOTERM_MF_ALL	GO:0016627~oxidoreductase activity, acting on the CH-CH	4
GOTERM_BP_ALL	GO:0048522~positive regulation of cellular process	7
GOTERM_BP_ALL	GO:0042545~cell wall modification	7
GOTERM_CC_ALL	GO:0044451~nucleoplasm part	7
GOTERM_BP_ALL	GO:0009582~detection of abiotic stimulus	3
GOTERM_BP_ALL	GO:0009060~aerobic respiration	3
GOTERM_BP_ALL	GO:0006555~methionine metabolic process	3
GOTERM_BP_ALL	GO:0050793~regulation of developmental process	13
GOTERM_BP_ALL	GO:0015980~energy derivation by oxidation of organic com	5
GOTERM_BP_ALL	GO:0046246~terpene biosynthetic process	2
GOTERM_BP_ALL	GO:0048863~stem cell differentiation	2
GOTERM_BP_ALL	GO:0009084~glutamine family amino acid biosynthetic pro	2
GOTERM_BP_ALL	GO:0009694~jasmonic acid metabolic process	2
GOTERM_CC_ALL	GO:0000428~DNA-directed RNA polymerase complex	2
GOTERM_CC_ALL	GO:0030880~RNA polymerase complex	2
GOTERM_CC_ALL	GO:0055029~nuclear DNA-directed RNA polymerase com	2
GOTERM_BP_ALL	GO:0031327~negative regulation of cellular biosynthetic pr	4
GOTERM_BP_ALL	GO:0009890~negative regulation of biosynthetic process	4
GOTERM_BP_ALL	GO:0010228~vegetative to reproductive phase transition	4
GOTERM_MF_ALL	GO:0046933~hydrogen ion transporting ATP synthase activ	2
GOTERM_MF_ALL	GO:0042578~phosphoric ester hydrolase activity	18
GOTERM_BP_ALL	GO:0009056~catabolic process	67
GOTERM_BP_ALL	GO:0009069~serine family amino acid metabolic process	3
GOTERM_BP_ALL	GO:0000096~sulfur amino acid metabolic process	4
GOTERM_MF_ALL	GO:0043492~ATPase activity, coupled to movement of sub	9
GOTERM_MF_ALL	GO:0042626~ATPase activity, coupled to transmembrane m	9
GOTERM_MF_ALL	GO:0022857~transmembrane transporter activity	49
GOTERM_CC_ALL	GO:0005743~mitochondrial inner membrane	9
GOTERM_BP_ALL	GO:0016246~RNA interference	2
GOTERM_BP_ALL	GO:0040034~regulation of development, heterochronic	2

GOTERM_CC_ALL	GO:0033178~proton-transporting two-sector ATPase compl	2
GOTERM_CC_ALL	GO:0009707~chloroplast outer membrane	2
GOTERM_MF_ALL	GO:0004197~cysteine-type endopeptidase activity	3
GOTERM_MF_ALL	GO:0004175~endopeptidase activity	15
GOTERM_BP_ALL	GO:0051239~regulation of multicellular organismal proces	10
GOTERM_MF_ALL	GO:0016682~oxidoreductase activity, acting on diphenols a	2
GOTERM_MF_ALL	GO:0008047~enzyme activator activity	4
GOTERM_BP_ALL	GO:0010605~negative regulation of macromolecule metabo	9
GOTERM_BP_ALL	GO:0006139~nucleobase, nucleoside, nucleotide and nuclei	130
GOTERM_BP_ALL	GO:0006541~glutamine metabolic process	2
GOTERM_BP_ALL	GO:0006563~L-serine metabolic process	2
GOTERM_BP_ALL	GO:0009581~detection of external stimulus	3
GOTERM_MF_ALL	GO:0015205~nucleobase transmembrane transporter activit	2
GOTERM_BP_ALL	GO:0009259~ribonucleotide metabolic process	7
GOTERM_MF_ALL	GO:0005529~sugar binding	6
GOTERM_MF_ALL	GO:0004185~serine-type carboxypeptidase activity	3
GOTERM_MF_ALL	GO:0070008~serine-type exopeptidase activity	3
GOTERM_CC_ALL	GO:0031226~intrinsic to plasma membrane	5
GOTERM_MF_ALL	GO:0016747~transferase activity, transferring acyl groups o	13
GOTERM_BP_ALL	GO:0009124~nucleoside monophosphate biosynthetic proc	2
GOTERM_BP_ALL	GO:0042219~cellular amino acid derivative catabolic proce	2
GOTERM_BP_ALL	GO:0043455~regulation of secondary metabolic process	2
GOTERM_BP_ALL	GO:0031047~gene silencing by RNA	5
GOTERM_BP_ALL	GO:0016458~gene silencing	6
GOTERM_CC_ALL	GO:0044428~nuclear part	30
GOTERM_MF_ALL	GO:0008017~microtubule binding	2
GOTERM_MF_ALL	GO:0016820~hydrolase activity, acting on acid anhydrides,	9
GOTERM_BP_ALL	GO:0048581~negative regulation of post-embryonic develo	3
GOTERM_BP_ALL	GO:0034622~cellular macromolecular complex assembly	10
GOTERM_BP_ALL	GO:0065003~macromolecular complex assembly	13
GOTERM_CC_ALL	GO:0019005~SCF ubiquitin ligase complex	2
GOTERM_BP_ALL	GO:0006820~anion transport	4
GOTERM_BP_ALL	GO:0009123~nucleoside monophosphate metabolic process	2
GOTERM_BP_ALL	GO:0051258~protein polymerization	2
GOTERM_BP_ALL	GO:0046700~heterocycle catabolic process	2
GOTERM_MF_ALL	GO:0016746~transferase activity, transferring acyl groups	14
GOTERM_MF_ALL	GO:0005319~lipid transporter activity	2
GOTERM_MF_ALL	GO:0008509~anion transmembrane transporter activity	5
GOTERM_MF_ALL	GO:0015405~P-P-bond-hydrolysis-driven transmembrane tr	11
GOTERM_BP_ALL	GO:0009887~organ morphogenesis	8
GOTERM_BP_ALL	GO:0006818~hydrogen transport	4
GOTERM_BP_ALL	GO:0015992~proton transport	4
GOTERM_MF_ALL	GO:0022891~substrate-specific transmembrane transporter	38
GOTERM_MF_ALL	GO:0015399~primary active transmembrane transporter act	11
GOTERM_BP_ALL	GO:0042773~ATP synthesis coupled electron transport	2
GOTERM_BP_ALL	GO:0051338~regulation of transferase activity	2
GOTERM_BP_ALL	GO:0009658~chloroplast organization	3

GOTERM_BP_ALL	GO:0055066~di-, tri-valent inorganic cation homeostasis	3
GOTERM_MF_ALL	GO:0010181~FMN binding	2
GOTERM_BP_ALL	GO:0051093~negative regulation of developmental process	4
GOTERM_MF_ALL	GO:0004553~hydrolase activity, hydrolyzing O-glycosyl co	20
GOTERM_BP_ALL	GO:0032502~developmental process	90
GOTERM_CC_ALL	GO:0016020~membrane	279
GOTERM_MF_ALL	GO:0004540~ribonuclease activity	4
GOTERM_CC_ALL	GO:0033177~proton-transporting two-sector ATPase compl	2
GOTERM_BP_ALL	GO:0051246~regulation of protein metabolic process	5
GOTERM_BP_ALL	GO:0051187~cofactor catabolic process	3
GOTERM_BP_ALL	GO:0016481~negative regulation of transcription	3
GOTERM_BP_ALL	GO:0009740~gibberellic acid mediated signaling	2
GOTERM_BP_ALL	GO:0019321~pentose metabolic process	2
GOTERM_BP_ALL	GO:0010476~gibberellin-mediated signaling	2
GOTERM_CC_ALL	GO:0005654~nucleoplasm	7
GOTERM_BP_ALL	GO:0050832~defense response to fungus	19
GOTERM_BP_ALL	GO:0010016~shoot morphogenesis	7
GOTERM_MF_ALL	GO:0042803~protein homodimerization activity	3
GOTERM_MF_ALL	GO:0022892~substrate-specific transporter activity	44
GOTERM_BP_ALL	GO:0010026~trichome differentiation	3
GOTERM_BP_ALL	GO:0035315~hair cell differentiation	3
GOTERM_BP_ALL	GO:0009555~pollen development	6
GOTERM_CC_ALL	GO:0009527~plastid outer membrane	2
GOTERM_BP_ALL	GO:0048869~cellular developmental process	21
GOTERM_BP_ALL	GO:0048481~ovule development	2
GOTERM_MF_ALL	GO:0004659~prenyltransferase activity	2
GOTERM_MF_ALL	GO:0016791~phosphatase activity	14
GOTERM_BP_ALL	GO:0048364~root development	9
GOTERM_BP_ALL	GO:0022622~root system development	9
GOTERM_MF_ALL	GO:0003824~catalytic activity	443
GOTERM_BP_ALL	GO:0009150~purine ribonucleotide metabolic process	6
GOTERM_BP_ALL	GO:0009152~purine ribonucleotide biosynthetic process	6
GOTERM_BP_ALL	GO:0008283~cell proliferation	2
GOTERM_BP_ALL	GO:0009116~nucleoside metabolic process	3
GOTERM_BP_ALL	GO:0009965~leaf morphogenesis	5
GOTERM_MF_ALL	GO:0008092~cytoskeletal protein binding	5
GOTERM_BP_ALL	GO:0009856~pollination	6
GOTERM_MF_ALL	GO:0015082~di-, tri-valent inorganic cation transmembrane	3
GOTERM_MF_ALL	GO:0051287~NAD or NADH binding	3
GOTERM_MF_ALL	GO:0016298~lipase activity	6
GOTERM_BP_ALL	GO:0003006~reproductive developmental process	39
GOTERM_CC_ALL	GO:0022625~cytosolic large ribosomal subunit	6
GOTERM_MF_ALL	GO:0016891~endoribonuclease activity, producing 5'-phosp	2
GOTERM_MF_ALL	GO:0016641~oxidoreductase activity, acting on the CH-NH	2
GOTERM_MF_ALL	GO:0003954~NADH dehydrogenase activity	2
GOTERM_BP_ALL	GO:0048569~post-embryonic organ development	8
GOTERM_CC_ALL	GO:0031461~cullin-RING ubiquitin ligase complex	7

GOTERM_BP_ALL	GO:0006839~mitochondrial transport	3
GOTERM_BP_ALL	GO:0045333~cellular respiration	4
GOTERM_BP_ALL	GO:0051174~regulation of phosphorus metabolic process	2
GOTERM_BP_ALL	GO:0019220~regulation of phosphate metabolic process	2
GOTERM_CC_ALL	GO:0031225~anchored to membrane	14
GOTERM_MF_ALL	GO:0016853~isomerase activity	10
GOTERM_CC_ALL	GO:0016591~DNA-directed RNA polymerase II, holoenzy	2
GOTERM_MF_ALL	GO:0045330~aspartyl esterase activity	3
GOTERM_BP_ALL	GO:0016036~cellular response to phosphate starvation	2
GOTERM_BP_ALL	GO:0048765~root hair cell differentiation	2
GOTERM_BP_ALL	GO:0048764~trichoblast maturation	2
GOTERM_BP_ALL	GO:0048573~photoperiodism, flowering	2
GOTERM_BP_ALL	GO:0048469~cell maturation	2
GOTERM_MF_ALL	GO:0008238~exopeptidase activity	4
GOTERM_MF_ALL	GO:0015631~tubulin binding	2
GOTERM_MF_ALL	GO:0008378~galactosyltransferase activity	2
GOTERM_MF_ALL	GO:0016887~ATPase activity	21
GOTERM_BP_ALL	GO:0006164~purine nucleotide biosynthetic process	6
GOTERM_MF_ALL	GO:0005488~binding	640
GOTERM_BP_ALL	GO:0030243~cellulose metabolic process	3
GOTERM_BP_ALL	GO:0048468~cell development	7
GOTERM_BP_ALL	GO:0009790~embryonic development	19
GOTERM_BP_ALL	GO:0034654~nucleobase, nucleoside, nucleotide and nuclei	8
GOTERM_BP_ALL	GO:0034404~nucleobase, nucleoside and nucleotide biosyn	8
GOTERM_BP_ALL	GO:0000226~microtubule cytoskeleton organization	2
GOTERM_BP_ALL	GO:0006417~regulation of translation	2
GOTERM_MF_ALL	GO:0016798~hydrolase activity, acting on glycosyl bonds	20
GOTERM_MF_ALL	GO:0060589~nucleoside-triphosphatase regulator activity	4
GOTERM_BP_ALL	GO:0045229~external encapsulating structure organization	13
GOTERM_BP_ALL	GO:0009657~plastid organization	4
GOTERM_BP_ALL	GO:0009260~ribonucleotide biosynthetic process	6
GOTERM_BP_ALL	GO:0006163~purine nucleotide metabolic process	6
GOTERM_BP_ALL	GO:0051234~establishment of localization	104
GOTERM_BP_ALL	GO:0051172~negative regulation of nitrogen compound me	3
GOTERM_BP_ALL	GO:0045934~negative regulation of nucleobase, nucleoside	3
GOTERM_MF_ALL	GO:0016893~endonuclease activity, active with either ribo-	2
GOTERM_BP_ALL	GO:0022607~cellular component assembly	14
GOTERM_BP_ALL	GO:0007275~multicellular organismal development	80
GOTERM_CC_ALL	GO:0080008~CUL4 RING ubiquitin ligase complex	5
GOTERM_BP_ALL	GO:0009932~cell tip growth	3
GOTERM_BP_ALL	GO:0010054~trichoblast differentiation	2
GOTERM_BP_ALL	GO:0009648~photoperiodism	2
GOTERM_BP_ALL	GO:0010629~negative regulation of gene expression	7
GOTERM_CC_ALL	GO:0005740~mitochondrial envelope	10
GOTERM_MF_ALL	GO:0016614~oxidoreductase activity, acting on CH-OH gro	7
GOTERM_MF_ALL	GO:0015078~hydrogen ion transmembrane transporter activ	6
GOTERM_BP_ALL	GO:0007047~cell wall organization	12

GOTERM_BP_ALL	GO:0048608~reproductive structure development	34
GOTERM_BP_ALL	GO:0010648~negative regulation of cell communication	2
GOTERM_BP_ALL	GO:0009968~negative regulation of signal transduction	2
GOTERM_MF_ALL	GO:0003735~structural constituent of ribosome	17
GOTERM_CC_ALL	GO:0015934~large ribosomal subunit	7
GOTERM_MF_ALL	GO:0003779~actin binding	3
GOTERM_CC_ALL	GO:0044429~mitochondrial part	13
GOTERM_CC_ALL	GO:0031980~mitochondrial lumen	3
GOTERM_CC_ALL	GO:0005759~mitochondrial matrix	3
GOTERM_MF_ALL	GO:0050661~NADP or NADPH binding	2
GOTERM_BP_ALL	GO:0009119~ribonucleoside metabolic process	2
GOTERM_MF_ALL	GO:0015297~antiporter activity	6
GOTERM_BP_ALL	GO:0051179~localization	106
GOTERM_BP_ALL	GO:0009165~nucleotide biosynthetic process	7
GOTERM_MF_ALL	GO:0004620~phospholipase activity	2
GOTERM_BP_ALL	GO:0048367~shoot development	12
GOTERM_BP_ALL	GO:0042445~hormone metabolic process	3
GOTERM_BP_ALL	GO:0010154~fruit development	20
GOTERM_BP_ALL	GO:0022904~respiratory electron transport chain	2
GOTERM_BP_ALL	GO:0048316~seed development	19
GOTERM_BP_ALL	GO:0043648~dicarboxylic acid metabolic process	3
GOTERM_BP_ALL	GO:0042546~cell wall biogenesis	3
GOTERM_BP_ALL	GO:0007154~cell communication	7
GOTERM_BP_ALL	GO:0006810~transport	102
GOTERM_CC_ALL	GO:0044459~plasma membrane part	11
GOTERM_BP_ALL	GO:0046417~chorismate metabolic process	2
GOTERM_BP_ALL	GO:0009073~aromatic amino acid family biosynthetic proc	2
GOTERM_BP_ALL	GO:0000904~cell morphogenesis involved in differentiatio	4
GOTERM_BP_ALL	GO:0022621~shoot system development	12
GOTERM_CC_ALL	GO:0031966~mitochondrial membrane	9
GOTERM_BP_ALL	GO:0009886~post-embryonic morphogenesis	3
GOTERM_BP_ALL	GO:0009738~abscisic acid mediated signaling	3
GOTERM_BP_ALL	GO:0009793~embryonic development ending in seed dorma	16
GOTERM_CC_ALL	GO:0044455~mitochondrial membrane part	3
GOTERM_MF_ALL	GO:0015932~nucleobase, nucleoside, nucleotide and nuclei	2
GOTERM_MF_ALL	GO:0008324~cation transmembrane transporter activity	19
GOTERM_BP_ALL	GO:0009626~plant-type hypersensitive response	2
GOTERM_MF_ALL	GO:0008415~acyltransferase activity	9
GOTERM_BP_ALL	GO:0006855~multidrug transport	3
GOTERM_BP_ALL	GO:0022414~reproductive process	41
GOTERM_BP_ALL	GO:0000398~nuclear mRNA splicing, via spliceosome	2
GOTERM_BP_ALL	GO:0034050~host programmed cell death induced by symb	2
GOTERM_BP_ALL	GO:0010053~root epidermal cell differentiation	2
GOTERM_BP_ALL	GO:0033365~protein localization in organelle	3
GOTERM_CC_ALL	GO:0030529~ribonucleoprotein complex	29
GOTERM_CC_ALL	GO:0015935~small ribosomal subunit	5
GOTERM_MF_ALL	GO:0004565~beta-galactosidase activity	2

GOTERM_MF_ALL	GO:0001882~nucleoside binding	122
GOTERM_BP_ALL	GO:0040029~regulation of gene expression, epigenetic	6
GOTERM_MF_ALL	GO:0022890~inorganic cation transmembrane transporter a	9
GOTERM_BP_ALL	GO:0048513~organ development	29
GOTERM_BP_ALL	GO:0006261~DNA-dependent DNA replication	2
GOTERM_BP_ALL	GO:0006605~protein targeting	5
GOTERM_BP_ALL	GO:0048731~system development	29
GOTERM_MF_ALL	GO:0008565~protein transporter activity	5
GOTERM_MF_ALL	GO:0042625~ATPase activity, coupled to transmembrane m	3
GOTERM_CC_ALL	GO:0016021~integral to membrane	103
GOTERM_BP_ALL	GO:0032501~multicellular organismal process	82
GOTERM_BP_ALL	GO:0048440~carpel development	2
GOTERM_MF_ALL	GO:0030554~adenyl nucleotide binding	121
GOTERM_MF_ALL	GO:0001883~purine nucleoside binding	121
GOTERM_BP_ALL	GO:0015893~drug transport	3
GOTERM_BP_ALL	GO:0017038~protein import	3
GOTERM_MF_ALL	GO:0015077~monovalent inorganic cation transmembrane t	6
GOTERM_BP_ALL	GO:0009653~anatomical structure morphogenesis	20
GOTERM_CC_ALL	GO:0043232~intracellular non-membrane-bounded organel	51
GOTERM_CC_ALL	GO:0043228~non-membrane-bounded organelle	51
GOTERM_MF_ALL	GO:0019899~enzyme binding	2
GOTERM_MF_ALL	GO:0015450~P-P-bond-hydrolysis-driven protein transmem	2
GOTERM_MF_ALL	GO:0022884~macromolecule transmembrane transporter ac	2
GOTERM_BP_ALL	GO:0044036~cell wall macromolecule metabolic process	2
GOTERM_MF_ALL	GO:0004518~nuclease activity	7
GOTERM_BP_ALL	GO:0055080~cation homeostasis	3
GOTERM_BP_ALL	GO:0042493~response to drug	3
GOTERM_BP_ALL	GO:0046034~ATP metabolic process	4
GOTERM_BP_ALL	GO:0007010~cytoskeleton organization	4
GOTERM_BP_ALL	GO:0006754~ATP biosynthetic process	4
GOTERM_MF_ALL	GO:0015925~galactosidase activity	2
GOTERM_CC_ALL	GO:0005783~endoplasmic reticulum	18
GOTERM_BP_ALL	GO:0032940~secretion by cell	2
GOTERM_BP_ALL	GO:0046903~secretion	2
GOTERM_BP_ALL	GO:0009860~pollen tube growth	2
GOTERM_MF_ALL	GO:0016866~intramolecular transferase activity	2
GOTERM_BP_ALL	GO:0000375~RNA splicing, via transesterification reaction	2
GOTERM_BP_ALL	GO:0000377~RNA splicing, via transesterification reaction	2
GOTERM_BP_ALL	GO:0009791~post-embryonic development	38
GOTERM_CC_ALL	GO:0005875~microtubule associated complex	2
GOTERM_BP_ALL	GO:0000003~reproduction	41
GOTERM_BP_ALL	GO:0007242~intracellular signaling cascade	35
GOTERM_MF_ALL	GO:0005543~phospholipid binding	3
GOTERM_MF_ALL	GO:0008483~transaminase activity	2
GOTERM_BP_ALL	GO:0006352~transcription initiation	2
GOTERM_MF_ALL	GO:0004519~endonuclease activity	3
GOTERM_BP_ALL	GO:0009913~epidermal cell differentiation	4

GOTERM_MF_ALL	GO:0016616~oxidoreductase activity, acting on the CH-OH	5
GOTERM_MF_ALL	GO:0008289~lipid binding	9
GOTERM_BP_ALL	GO:0048856~anatomical structure development	61
GOTERM_BP_ALL	GO:0010073~meristem maintenance	2
GOTERM_CC_ALL	GO:0033279~ribosomal subunit	11
GOTERM_BP_ALL	GO:0009144~purine nucleoside triphosphate metabolic pro	4
GOTERM_BP_ALL	GO:0009199~ribonucleoside triphosphate metabolic proces	4
GOTERM_BP_ALL	GO:0009205~purine ribonucleoside triphosphate metabolic	4
GOTERM_BP_ALL	GO:0009206~purine ribonucleoside triphosphate biosynthet	4
GOTERM_BP_ALL	GO:0009201~ribonucleoside triphosphate biosynthetic proc	4
GOTERM_BP_ALL	GO:0009145~purine nucleoside triphosphate biosynthetic p	4
GOTERM_BP_ALL	GO:0048467~gynoecium development	2
GOTERM_BP_ALL	GO:0048584~positive regulation of response to stimulus	2
GOTERM_BP_ALL	GO:0048509~regulation of meristem development	2
GOTERM_BP_ALL	GO:0007398~ectoderm development	4
GOTERM_BP_ALL	GO:0009142~nucleoside triphosphate biosynthetic process	4
GOTERM_BP_ALL	GO:0008544~epidermis development	4
GOTERM_MF_ALL	GO:0003755~peptidyl-prolyl cis-trans isomerase activity	2
GOTERM_BP_ALL	GO:0009141~nucleoside triphosphate metabolic process	4
GOTERM_BP_ALL	GO:0015674~di-, tri-valent inorganic cation transport	2
GOTERM_MF_ALL	GO:0016859~cis-trans isomerase activity	2
GOTERM_CC_ALL	GO:0044432~endoplasmic reticulum part	3
GOTERM_BP_ALL	GO:0044242~cellular lipid catabolic process	2
GOTERM_BP_ALL	GO:0048878~chemical homeostasis	4
GOTERM_BP_ALL	GO:0010817~regulation of hormone levels	4
GOTERM_BP_ALL	GO:0006915~apoptosis	6
GOTERM_MF_ALL	GO:0035091~phosphoinositide binding	2
GOTERM_MF_ALL	GO:0030955~potassium ion binding	2
GOTERM_MF_ALL	GO:0042623~ATPase activity, coupled	13
GOTERM_BP_ALL	GO:0009101~glycoprotein biosynthetic process	2
GOTERM_BP_ALL	GO:0043413~biopolymer glycosylation	2
GOTERM_BP_ALL	GO:0006486~protein amino acid glycosylation	2
GOTERM_BP_ALL	GO:0070085~glycosylation	2
GOTERM_BP_ALL	GO:0016569~covalent chromatin modification	2
GOTERM_BP_ALL	GO:0012501~programmed cell death	8
GOTERM_BP_ALL	GO:0048507~meristem development	3
GOTERM_MF_ALL	GO:0008320~protein transmembrane transporter activity	2
GOTERM_BP_ALL	GO:0007017~microtubule-based process	4
GOTERM_MF_ALL	GO:0004722~protein serine/threonine phosphatase activity	5
GOTERM_CC_ALL	GO:0000786~nucleosome	2
GOTERM_BP_ALL	GO:0006886~intracellular protein transport	10
GOTERM_BP_ALL	GO:0050801~ion homeostasis	3
GOTERM_BP_ALL	GO:0048646~anatomical structure formation involved in m	3
GOTERM_MF_ALL	GO:0016772~transferase activity, transferring phosphorus-c	71
GOTERM_CC_ALL	GO:0031968~organelle outer membrane	2
GOTERM_MF_ALL	GO:0005198~structural molecule activity	21
GOTERM_CC_ALL	GO:0070469~respiratory chain	3

GOTERM_BP_ALL	GO:0009100~glycoprotein metabolic process	2
GOTERM_BP_ALL	GO:0008219~cell death	9
GOTERM_BP_ALL	GO:0016265~death	9
GOTERM_BP_ALL	GO:0070727~cellular macromolecule localization	11
GOTERM_BP_ALL	GO:0032989~cellular component morphogenesis	8
GOTERM_BP_ALL	GO:0000902~cell morphogenesis	7
GOTERM_BP_ALL	GO:0051128~regulation of cellular component organization	2
GOTERM_BP_ALL	GO:0019953~sexual reproduction	2
GOTERM_MF_ALL	GO:0003777~microtubule motor activity	2
GOTERM_BP_ALL	GO:0048366~leaf development	6
GOTERM_MF_ALL	GO:0008233~peptidase activity	23
GOTERM_CC_ALL	GO:0019867~outer membrane	2
GOTERM_MF_ALL	GO:0070011~peptidase activity, acting on L-amino acid pe	21
GOTERM_BP_ALL	GO:0034613~cellular protein localization	10
GOTERM_BP_ALL	GO:0048585~negative regulation of response to stimulus	2
GOTERM_BP_ALL	GO:0015672~monovalent inorganic cation transport	5
GOTERM_BP_ALL	GO:0065009~regulation of molecular function	5
GOTERM_MF_ALL	GO:0016769~transferase activity, transferring nitrogenous g	2
GOTERM_CC_ALL	GO:0032993~protein-DNA complex	2
GOTERM_MF_ALL	GO:0015075~ion transmembrane transporter activity	22
GOTERM_BP_ALL	GO:0006334~nucleosome assembly	2
GOTERM_BP_ALL	GO:0034728~nucleosome organization	2
GOTERM_MF_ALL	GO:0016301~kinase activity	60
GOTERM_BP_ALL	GO:0044092~negative regulation of molecular function	2
GOTERM_MF_ALL	GO:0004650~polygalacturonase activity	2
GOTERM_MF_ALL	GO:0004527~exonuclease activity	2
GOTERM_BP_ALL	GO:0042254~ribosome biogenesis	8
GOTERM_MF_ALL	GO:0016810~hydrolase activity, acting on carbon-nitrogen	3
GOTERM_BP_ALL	GO:0031497~chromatin assembly	2
GOTERM_BP_ALL	GO:0006470~protein amino acid dephosphorylation	2
GOTERM_BP_ALL	GO:0010876~lipid localization	5
GOTERM_MF_ALL	GO:0004091~carboxylesterase activity	12
GOTERM_MF_ALL	GO:0017076~purine nucleotide binding	128
GOTERM_BP_ALL	GO:0065004~protein-DNA complex assembly	2
GOTERM_MF_ALL	GO:0031420~alkali metal ion binding	2
GOTERM_BP_ALL	GO:0009888~tissue development	8
GOTERM_BP_ALL	GO:0080134~regulation of response to stress	2
GOTERM_BP_ALL	GO:0048588~developmental cell growth	2
GOTERM_BP_ALL	GO:0000272~polysaccharide catabolic process	2
GOTERM_MF_ALL	GO:0019843~rRNA binding	2
GOTERM_MF_ALL	GO:0016773~phosphotransferase activity, alcohol group as	53
GOTERM_CC_ALL	GO:0022627~cytosolic small ribosomal subunit	3
GOTERM_BP_ALL	GO:0022613~ribonucleoprotein complex biogenesis	8
GOTERM_MF_ALL	GO:0070035~purine NTP-dependent helicase activity	3
GOTERM_MF_ALL	GO:0008026~ATP-dependent helicase activity	3
GOTERM_BP_ALL	GO:0044085~cellular component biogenesis	24
GOTERM_MF_ALL	GO:0016874~ligase activity	18

GOTERM_MF_ALL	GO:0060089~molecular transducer activity	30
GOTERM_MF_ALL	GO:0004871~signal transducer activity	30
GOTERM_MF_ALL	GO:0005516~calmodulin binding	5
GOTERM_MF_ALL	GO:0005524~ATP binding	105
GOTERM_BP_ALL	GO:0008380~RNA splicing	3
GOTERM_BP_ALL	GO:0034645~cellular macromolecule biosynthetic process	133
GOTERM_BP_ALL	GO:0030001~metal ion transport	8
GOTERM_MF_ALL	GO:0046873~metal ion transmembrane transporter activity	4
GOTERM_BP_ALL	GO:0006323~DNA packaging	2
GOTERM_MF_ALL	GO:0004721~phosphoprotein phosphatase activity	6
GOTERM_BP_ALL	GO:0009059~macromolecule biosynthetic process	133
GOTERM_BP_ALL	GO:0006811~ion transport	18
GOTERM_BP_ALL	GO:0009664~plant-type cell wall organization	2
GOTERM_BP_ALL	GO:0032259~methylation	2
GOTERM_CC_ALL	GO:0031224~intrinsic to membrane	119
GOTERM_BP_ALL	GO:0007165~signal transduction	51
GOTERM_BP_ALL	GO:0006869~lipid transport	4
GOTERM_BP_ALL	GO:0048827~phyllome development	6
GOTERM_MF_ALL	GO:0005083~small GTPase regulator activity	2
GOTERM_CC_ALL	GO:0000151~ubiquitin ligase complex	7
GOTERM_BP_ALL	GO:0016042~lipid catabolic process	6
GOTERM_BP_ALL	GO:0048610~reproductive cellular process	3
GOTERM_BP_ALL	GO:0006812~cation transport	13
GOTERM_BP_ALL	GO:0050790~regulation of catalytic activity	4
GOTERM_BP_ALL	GO:0051649~establishment of localization in cell	16
GOTERM_MF_ALL	GO:0032559~adenyl ribonucleotide binding	105
GOTERM_CC_ALL	GO:0044445~cytosolic part	8
GOTERM_BP_ALL	GO:0016311~dephosphorylation	2
GOTERM_MF_ALL	GO:0016879~ligase activity, forming carbon-nitrogen bond	11
GOTERM_BP_ALL	GO:0009966~regulation of signal transduction	3
GOTERM_BP_ALL	GO:0048583~regulation of response to stimulus	4
GOTERM_MF_ALL	GO:0030695~GTPase regulator activity	2
GOTERM_BP_ALL	GO:0009987~cellular process	514
GOTERM_CC_ALL	GO:0015630~microtubule cytoskeleton	4
GOTERM_MF_ALL	GO:0005216~ion channel activity	2
GOTERM_MF_ALL	GO:0016835~carbon-oxygen lyase activity	3
GOTERM_MF_ALL	GO:0008234~cysteine-type peptidase activity	4
GOTERM_BP_ALL	GO:0016071~mRNA metabolic process	4
GOTERM_BP_ALL	GO:0006364~rRNA processing	3
GOTERM_BP_ALL	GO:0006260~DNA replication	3
GOTERM_BP_ALL	GO:0016072~rRNA metabolic process	3
GOTERM_BP_ALL	GO:0060560~developmental growth involved in morphogen	4
GOTERM_BP_ALL	GO:0009826~unidimensional cell growth	4
GOTERM_BP_ALL	GO:0010646~regulation of cell communication	3
GOTERM_CC_ALL	GO:0000785~chromatin	2
GOTERM_BP_ALL	GO:0001906~cell killing	9
GOTERM_BP_ALL	GO:0031640~killing of cells of another organism	9

GOTERM_BP_ALL	GO:0046907~intracellular transport	13
GOTERM_MF_ALL	GO:0003676~nucleic acid binding	195
GOTERM_CC_ALL	GO:0044425~membrane part	143
GOTERM_MF_ALL	GO:0016788~hydrolase activity, acting on ester bonds	40
GOTERM_BP_ALL	GO:0016310~phosphorylation	48
GOTERM_BP_ALL	GO:0003002~regionalization	2
GOTERM_BP_ALL	GO:0051641~cellular localization	17
GOTERM_BP_ALL	GO:0007264~small GTPase mediated signal transduction	2
GOTERM_BP_ALL	GO:0007169~transmembrane receptor protein tyrosine kina	3
GOTERM_BP_ALL	GO:0007167~enzyme linked receptor protein signaling path	3
GOTERM_MF_ALL	GO:0004674~protein serine/threonine kinase activity	37
GOTERM_BP_ALL	GO:0006333~chromatin assembly or disassembly	2
GOTERM_BP_ALL	GO:0007166~cell surface receptor linked signal transductio	4
GOTERM_MF_ALL	GO:0003774~motor activity	2
GOTERM_BP_ALL	GO:0008361~regulation of cell size	6
GOTERM_BP_ALL	GO:0009814~defense response, incompatible interaction	2
GOTERM_MF_ALL	GO:0005525~GTP binding	7
GOTERM_BP_ALL	GO:0016568~chromatin modification	3
GOTERM_MF_ALL	GO:0004386~helicase activity	4
GOTERM_MF_ALL	GO:0008236~serine-type peptidase activity	4
GOTERM_MF_ALL	GO:0017171~serine hydrolase activity	4
GOTERM_BP_ALL	GO:0006796~phosphate metabolic process	51
GOTERM_MF_ALL	GO:0004672~protein kinase activity	42
GOTERM_BP_ALL	GO:0006793~phosphorus metabolic process	51
GOTERM_CC_ALL	GO:0043234~protein complex	54
GOTERM_BP_ALL	GO:0032535~regulation of cellular component size	6
GOTERM_BP_ALL	GO:0006468~protein amino acid phosphorylation	41
GOTERM_MF_ALL	GO:0019787~small conjugating protein ligase activity	7
GOTERM_MF_ALL	GO:0004872~receptor activity	19
GOTERM_BP_ALL	GO:0010467~gene expression	134
GOTERM_BP_ALL	GO:0048589~developmental growth	4
GOTERM_BP_ALL	GO:0006281~DNA repair	4
GOTERM_BP_ALL	GO:0016049~cell growth	5
GOTERM_MF_ALL	GO:0016462~pyrophosphatase activity	29
GOTERM_BP_ALL	GO:0040007~growth	6
GOTERM_CC_ALL	GO:0044430~cytoskeletal part	4
GOTERM_BP_ALL	GO:0045087~innate immune response	7
GOTERM_MF_ALL	GO:0032561~guanyl ribonucleotide binding	7
GOTERM_CC_ALL	GO:0044427~chromosomal part	3
GOTERM_MF_ALL	GO:0000166~nucleotide binding	147
GOTERM_MF_ALL	GO:0032555~purine ribonucleotide binding	112
GOTERM_MF_ALL	GO:0032553~ribonucleotide binding	112
GOTERM_BP_ALL	GO:0007389~pattern specification process	2
GOTERM_BP_ALL	GO:0034470~ncRNA processing	4
GOTERM_BP_ALL	GO:0006325~chromatin organization	5
GOTERM_MF_ALL	GO:0019001~guanyl nucleotide binding	7
GOTERM_BP_ALL	GO:0051726~regulation of cell cycle	2

GOTERM_BP_ALL	GO:0006974~response to DNA damage stimulus	4
GOTERM_MF_ALL	GO:0016818~hydrolase activity, acting on acid anhydrides,	29
GOTERM_CC_ALL	GO:0005874~microtubule	2
GOTERM_BP_ALL	GO:0009734~auxin mediated signaling pathway	2
GOTERM_MF_ALL	GO:0016817~hydrolase activity, acting on acid anhydrides	29
GOTERM_MF_ALL	GO:0016881~acid-amino acid ligase activity	7
GOTERM_MF_ALL	GO:0004842~ubiquitin-protein ligase activity	6
GOTERM_MF_ALL	GO:0004888~transmembrane receptor activity	4
GOTERM_BP_ALL	GO:0006955~immune response	7
GOTERM_CC_ALL	GO:0032991~macromolecular complex	84
GOTERM_CC_ALL	GO:0005856~cytoskeleton	5
GOTERM_BP_ALL	GO:0016567~protein ubiquitination	2
GOTERM_CC_ALL	GO:0005694~chromosome	4
GOTERM_MF_ALL	GO:0017111~nucleoside-triphosphatase activity	26
GOTERM_BP_ALL	GO:0051301~cell division	3
GOTERM_BP_ALL	GO:0002376~immune system process	7
GOTERM_BP_ALL	GO:0032446~protein modification by small protein conjuga	2
GOTERM_BP_ALL	GO:0006511~ubiquitin-dependent protein catabolic process	6
GOTERM_BP_ALL	GO:0006397~mRNA processing	2
GOTERM_BP_ALL	GO:0044265~cellular macromolecule catabolic process	17
GOTERM_BP_ALL	GO:0051276~chromosome organization	5
GOTERM_BP_ALL	GO:0016043~cellular component organization	45
GOTERM_BP_ALL	GO:0070647~protein modification by small protein conjuga	2
GOTERM_BP_ALL	GO:0006508~proteolysis	35
GOTERM_BP_ALL	GO:0006996~organelle organization	17
GOTERM_BP_ALL	GO:0044238~primary metabolic process	401
GOTERM_MF_ALL	GO:0004713~protein tyrosine kinase activity	6
GOTERM_BP_ALL	GO:0044257~cellular protein catabolic process	15
GOTERM_BP_ALL	GO:0043687~post-translational protein modification	51
GOTERM_BP_ALL	GO:0045184~establishment of protein localization	12
GOTERM_BP_ALL	GO:0015031~protein transport	12
GOTERM_BP_ALL	GO:0006396~RNA processing	10
GOTERM_BP_ALL	GO:0030163~protein catabolic process	15
GOTERM_BP_ALL	GO:0043632~modification-dependent macromolecule catab	14
GOTERM_BP_ALL	GO:0019941~modification-dependent protein catabolic pro	14
GOTERM_BP_ALL	GO:0006259~DNA metabolic process	7
GOTERM_BP_ALL	GO:0009057~macromolecule catabolic process	19
GOTERM_MF_ALL	GO:0016787~hydrolase activity	127
GOTERM_BP_ALL	GO:0051603~proteolysis involved in cellular protein catabo	14
GOTERM_BP_ALL	GO:0008104~protein localization	12
GOTERM_CC_ALL	GO:0012505~endomembrane system	172
GOTERM_BP_ALL	GO:0034660~ncRNA metabolic process	4
GOTERM_CC_ALL	GO:0005794~Golgi apparatus	6
GOTERM_BP_ALL	GO:0033036~macromolecule localization	18
GOTERM_BP_ALL	GO:0016070~RNA metabolic process	16
GOTERM_BP_ALL	GO:0006464~protein modification process	54
GOTERM_BP_ALL	GO:0007049~cell cycle	2

GOTERM_BP_ALL	GO:0043412~biopolymer modification	55
GOTERM_BP_ALL	GO:0044260~cellular macromolecule metabolic process	240
GOTERM_BP_ALL	GO:0043170~macromolecule metabolic process	264
GOTERM_BP_ALL	GO:0006412~translation	25
GOTERM_BP_ALL	GO:0044267~cellular protein metabolic process	118
GOTERM_BP_ALL	GO:0019538~protein metabolic process	140
GOTERM_MF_ALL	GO:0003723~RNA binding	22
GOTERM_BP_ALL	GO:0006414~translational elongation	2
GOTERM_BP_ALL	GO:0052545~callose localization	1
GOTERM_BP_ALL	GO:0009690~cytokinin metabolic process	1
GOTERM_BP_ALL	GO:0010039~response to iron ion	1
GOTERM_BP_ALL	GO:0015969~guanosine tetraphosphate metabolic process	1
GOTERM_BP_ALL	GO:0033194~response to hydroperoxide	1
GOTERM_BP_ALL	GO:0031050~dsRNA fragmentation	1
GOTERM_BP_ALL	GO:0006012~galactose metabolic process	1
GOTERM_BP_ALL	GO:0010335~response to non-ionic osmotic stress	1
GOTERM_BP_ALL	GO:0048444~floral organ morphogenesis	1
GOTERM_BP_ALL	GO:0016104~triterpenoid biosynthetic process	1
GOTERM_BP_ALL	GO:0032272~negative regulation of protein polymerization	1
GOTERM_BP_ALL	GO:0043414~biopolymer methylation	1
GOTERM_BP_ALL	GO:0009768~photosynthesis, light harvesting in photosyste	1
GOTERM_BP_ALL	GO:0070589~cellular component macromolecule biosynthe	1
GOTERM_BP_ALL	GO:0032886~regulation of microtubule-based process	1
GOTERM_BP_ALL	GO:0009816~defense response to bacterium, incompatible i	1
GOTERM_BP_ALL	GO:0006873~cellular ion homeostasis	1
GOTERM_BP_ALL	GO:0043255~regulation of carbohydrate biosynthetic proce	1
GOTERM_BP_ALL	GO:0006528~asparagine metabolic process	1
GOTERM_BP_ALL	GO:0010337~regulation of salicylic acid metabolic process	1
GOTERM_BP_ALL	GO:0009252~peptidoglycan biosynthetic process	1
GOTERM_BP_ALL	GO:0009606~tropism	1
GOTERM_BP_ALL	GO:0080022~primary root development	1
GOTERM_BP_ALL	GO:0009082~branched chain family amino acid biosyntheti	1
GOTERM_BP_ALL	GO:0048609~reproductive process in a multicellular organi	1
GOTERM_BP_ALL	GO:0009629~response to gravity	1
GOTERM_BP_ALL	GO:0006144~purine base metabolic process	1
GOTERM_BP_ALL	GO:0048544~recognition of pollen	1
GOTERM_BP_ALL	GO:0016128~phytosteroid metabolic process	1
GOTERM_BP_ALL	GO:0042435~indole derivative biosynthetic process	1
GOTERM_BP_ALL	GO:0006268~DNA unwinding during replication	1
GOTERM_BP_ALL	GO:0006109~regulation of carbohydrate metabolic process	1
GOTERM_BP_ALL	GO:0010360~negative regulation of anion channel activity	1
GOTERM_BP_ALL	GO:0010152~pollen maturation	1
GOTERM_BP_ALL	GO:0007205~activation of protein kinase C activity by G-p	1
GOTERM_BP_ALL	GO:0050776~regulation of immune response	1
GOTERM_BP_ALL	GO:0019359~nicotinamide nucleotide biosynthetic process	1
GOTERM_BP_ALL	GO:0046836~glycolipid transport	1
GOTERM_BP_ALL	GO:0006024~glycosaminoglycan biosynthetic process	1

GOTERM_BP_ALL	GO:0006306~DNA methylation	1
GOTERM_BP_ALL	GO:0010043~response to zinc ion	1
GOTERM_BP_ALL	GO:0010075~regulation of meristem growth	1
GOTERM_BP_ALL	GO:0042547~cell wall modification during multidimension	1
GOTERM_BP_ALL	GO:0010942~positive regulation of cell death	1
GOTERM_BP_ALL	GO:0009715~chalcone biosynthetic process	1
GOTERM_BP_ALL	GO:0051410~detoxification of nitrogen compound	1
GOTERM_BP_ALL	GO:0051321~meiotic cell cycle	1
GOTERM_BP_ALL	GO:0000103~sulfate assimilation	1
GOTERM_BP_ALL	GO:0006522~alanine metabolic process	1
GOTERM_BP_ALL	GO:0031537~regulation of anthocyanin metabolic process	1
GOTERM_BP_ALL	GO:0042168~heme metabolic process	1
GOTERM_BP_ALL	GO:0046685~response to arsenic	1
GOTERM_BP_ALL	GO:0044087~regulation of cellular component biogenesis	1
GOTERM_BP_ALL	GO:0031329~regulation of cellular catabolic process	1
GOTERM_BP_ALL	GO:0030245~cellulose catabolic process	1
GOTERM_BP_ALL	GO:0022898~regulation of transmembrane transporter activ	1
GOTERM_BP_ALL	GO:0012502~induction of programmed cell death	1
GOTERM_BP_ALL	GO:0019637~organophosphate metabolic process	1
GOTERM_BP_ALL	GO:0031998~regulation of fatty acid beta-oxidation	1
GOTERM_BP_ALL	GO:0042762~regulation of sulfur metabolic process	1
GOTERM_BP_ALL	GO:0032413~negative regulation of ion transmembrane tra	1
GOTERM_BP_ALL	GO:0010289~homogalacturonan biosynthetic process	1
GOTERM_BP_ALL	GO:0015692~lead ion transport	1
GOTERM_BP_ALL	GO:0010639~negative regulation of organelle organization	1
GOTERM_BP_ALL	GO:0007015~actin filament organization	1
GOTERM_BP_ALL	GO:0018920~glyphosate metabolic process	1
GOTERM_BP_ALL	GO:0009894~regulation of catabolic process	1
GOTERM_BP_ALL	GO:0010374~stomatal complex development	1
GOTERM_BP_ALL	GO:0009696~salicylic acid metabolic process	1
GOTERM_BP_ALL	GO:0032504~multicellular organism reproduction	1
GOTERM_BP_ALL	GO:0045859~regulation of protein kinase activity	1
GOTERM_BP_ALL	GO:0009962~regulation of flavonoid biosynthetic process	1
GOTERM_BP_ALL	GO:0006305~DNA alkylation	1
GOTERM_BP_ALL	GO:0006346~methylation-dependent chromatin silencing	1
GOTERM_BP_ALL	GO:0030003~cellular cation homeostasis	1
GOTERM_BP_ALL	GO:0002213~defense response to insect	1
GOTERM_BP_ALL	GO:0022604~regulation of cell morphogenesis	1
GOTERM_BP_ALL	GO:0019375~galactolipid biosynthetic process	1
GOTERM_BP_ALL	GO:0048586~regulation of long-day photoperiodism, flowe	1
GOTERM_BP_ALL	GO:0043066~negative regulation of apoptosis	1
GOTERM_BP_ALL	GO:0050879~multicellular organismal movement	1
GOTERM_BP_ALL	GO:0046320~regulation of fatty acid oxidation	1
GOTERM_BP_ALL	GO:0006783~heme biosynthetic process	1
GOTERM_BP_ALL	GO:0009825~multidimensional cell growth	1
GOTERM_BP_ALL	GO:0042752~regulation of circadian rhythm	1
GOTERM_BP_ALL	GO:0048445~carpel morphogenesis	1

GOTERM_BP_ALL	GO:0042447~hormone catabolic process	1
GOTERM_BP_ALL	GO:0009933~meristem structural organization	1
GOTERM_BP_ALL	GO:0009625~response to insect	1
GOTERM_BP_ALL	GO:0008272~sulfate transport	1
GOTERM_BP_ALL	GO:0010304~PSII associated light-harvesting complex II ca	1
GOTERM_BP_ALL	GO:0009078~pyruvate family amino acid metabolic process	1
GOTERM_BP_ALL	GO:0015688~iron chelate transport	1
GOTERM_BP_ALL	GO:0010193~response to ozone	1
GOTERM_BP_ALL	GO:0042434~indole derivative metabolic process	1
GOTERM_BP_ALL	GO:0042256~mature ribosome assembly	1
GOTERM_BP_ALL	GO:0032271~regulation of protein polymerization	1
GOTERM_BP_ALL	GO:0009112~nucleobase metabolic process	1
GOTERM_BP_ALL	GO:0010082~regulation of root meristem growth	1
GOTERM_BP_ALL	GO:0046112~nucleobase biosynthetic process	1
GOTERM_BP_ALL	GO:0008360~regulation of cell shape	1
GOTERM_BP_ALL	GO:0034765~regulation of ion transmembrane transport	1
GOTERM_BP_ALL	GO:0006643~membrane lipid metabolic process	1
GOTERM_BP_ALL	GO:0052018~modulation by symbiont of RNA levels in hos	1
GOTERM_BP_ALL	GO:0009850~auxin metabolic process	1
GOTERM_BP_ALL	GO:0033233~regulation of protein sumoylation	1
GOTERM_BP_ALL	GO:0045489~pectin biosynthetic process	1
GOTERM_BP_ALL	GO:0019217~regulation of fatty acid metabolic process	1
GOTERM_BP_ALL	GO:0009970~cellular response to sulfate starvation	1
GOTERM_BP_ALL	GO:0045088~regulation of innate immune response	1
GOTERM_BP_ALL	GO:0016123~xanthophyll biosynthetic process	1
GOTERM_BP_ALL	GO:0000273~lipoic acid metabolic process	1
GOTERM_BP_ALL	GO:0006586~indolalkylamine metabolic process	1
GOTERM_BP_ALL	GO:0006406~mRNA export from nucleus	1
GOTERM_BP_ALL	GO:0006399~tRNA metabolic process	1
GOTERM_BP_ALL	GO:0009832~plant-type cell wall biogenesis	1
GOTERM_BP_ALL	GO:0009787~regulation of abscisic acid mediated signaling	1
GOTERM_BP_ALL	GO:0030005~cellular di-, tri-valent inorganic cation homeo	1
GOTERM_BP_ALL	GO:0006085~acetyl-CoA biosynthetic process	1
GOTERM_BP_ALL	GO:0043068~positive regulation of programmed cell death	1
GOTERM_BP_ALL	GO:0010439~regulation of glucosinolate biosynthetic proce	1
GOTERM_BP_ALL	GO:0001708~cell fate specification	1
GOTERM_BP_ALL	GO:0080010~regulation of oxygen and reactive oxygen spe	1
GOTERM_BP_ALL	GO:0015854~guanine transport	1
GOTERM_BP_ALL	GO:0006664~glycolipid metabolic process	1
GOTERM_BP_ALL	GO:0042853~L-alanine catabolic process	1
GOTERM_BP_ALL	GO:0019430~removal of superoxide radicals	1
GOTERM_BP_ALL	GO:0046219~indolalkylamine biosynthetic process	1
GOTERM_BP_ALL	GO:0009105~lipoic acid biosynthetic process	1
GOTERM_BP_ALL	GO:0045596~negative regulation of cell differentiation	1
GOTERM_BP_ALL	GO:0045116~protein neddylation	1
GOTERM_BP_ALL	GO:0006875~cellular metal ion homeostasis	1
GOTERM_BP_ALL	GO:0043331~response to dsRNA	1

GOTERM_BP_ALL	GO:0030029~actin filament-based process	1
GOTERM_BP_ALL	GO:0006108~malate metabolic process	1
GOTERM_BP_ALL	GO:0032527~protein exit from endoplasmic reticulum	1
GOTERM_BP_ALL	GO:0006982~response to lipid hydroperoxide	1
GOTERM_BP_ALL	GO:0009749~response to glucose stimulus	1
GOTERM_BP_ALL	GO:0000305~response to oxygen radical	1
GOTERM_BP_ALL	GO:0048638~regulation of developmental growth	1
GOTERM_BP_ALL	GO:0006525~arginine metabolic process	1
GOTERM_BP_ALL	GO:0009081~branched chain family amino acid metabolic	1
GOTERM_BP_ALL	GO:0010208~pollen wall assembly	1
GOTERM_BP_ALL	GO:0051494~negative regulation of cytoskeleton organizati	1
GOTERM_BP_ALL	GO:0042430~indole and derivative metabolic process	1
GOTERM_BP_ALL	GO:0019742~pentacyclic triterpenoid metabolic process	1
GOTERM_BP_ALL	GO:0010143~cutin biosynthetic process	1
GOTERM_BP_ALL	GO:0033234~negative regulation of protein sumoylation	1
GOTERM_BP_ALL	GO:0032412~regulation of ion transmembrane transporter a	1
GOTERM_BP_ALL	GO:0031111~negative regulation of microtubule polymeriz	1
GOTERM_BP_ALL	GO:0019296~coenzyme M metabolic process	1
GOTERM_BP_ALL	GO:0009926~auxin polar transport	1
GOTERM_BP_ALL	GO:0006983~ER overload response	1
GOTERM_BP_ALL	GO:0006023~aminoglycan biosynthetic process	1
GOTERM_BP_ALL	GO:0044403~symbiosis, encompassing mutualism through	1
GOTERM_BP_ALL	GO:0010188~response to microbial phytotoxin	1
GOTERM_BP_ALL	GO:0046777~protein amino acid autophosphorylation	1
GOTERM_BP_ALL	GO:0080021~response to benzoic acid stimulus	1
GOTERM_BP_ALL	GO:0007186~G-protein coupled receptor protein signaling	1
GOTERM_BP_ALL	GO:0010380~regulation of chlorophyll biosynthetic process	1
GOTERM_BP_ALL	GO:0046084~adenine biosynthetic process	1
GOTERM_BP_ALL	GO:0052386~cell wall thickening	1
GOTERM_BP_ALL	GO:0010206~photosystem II repair	1
GOTERM_BP_ALL	GO:0006828~manganese ion transport	1
GOTERM_BP_ALL	GO:0009616~virus induced gene silencing	1
GOTERM_BP_ALL	GO:0034976~response to endoplasmic reticulum stress	1
GOTERM_BP_ALL	GO:0048629~trichome patterning	1
GOTERM_BP_ALL	GO:0031122~cytoplasmic microtubule organization	1
GOTERM_BP_ALL	GO:0044038~cell wall macromolecule biosynthetic process	1
GOTERM_BP_ALL	GO:0009714~chalcone metabolic process	1
GOTERM_BP_ALL	GO:0042550~photosystem I stabilization	1
GOTERM_BP_ALL	GO:0055065~metal ion homeostasis	1
GOTERM_BP_ALL	GO:0044419~interspecies interaction between organisms	1
GOTERM_BP_ALL	GO:0006304~DNA modification	1
GOTERM_BP_ALL	GO:0017148~negative regulation of translation	1
GOTERM_BP_ALL	GO:0009088~threonine biosynthetic process	1
GOTERM_BP_ALL	GO:0042727~riboflavin and derivative biosynthetic process	1
GOTERM_BP_ALL	GO:0045089~positive regulation of innate immune respons	1
GOTERM_BP_ALL	GO:0002218~activation of innate immune response	1
GOTERM_BP_ALL	GO:0009268~response to pH	1

GOTERM_BP_ALL	GO:0019758~glycosinolate biosynthetic process	1
GOTERM_BP_ALL	GO:0009823~cytokinin catabolic process	1
GOTERM_BP_ALL	GO:0000060~protein import into nucleus, translocation	1
GOTERM_BP_ALL	GO:0006596~polyamine biosynthetic process	1
GOTERM_BP_ALL	GO:0007267~cell-cell signaling	1
GOTERM_BP_ALL	GO:0015908~fatty acid transport	1
GOTERM_BP_ALL	GO:0009704~de-etiolation	1
GOTERM_BP_ALL	GO:0060548~negative regulation of cell death	1
GOTERM_BP_ALL	GO:0006546~glycine catabolic process	1
GOTERM_BP_ALL	GO:0046173~polyol biosynthetic process	1
GOTERM_BP_ALL	GO:0031400~negative regulation of protein modification pr	1
GOTERM_BP_ALL	GO:0006813~potassium ion transport	1
GOTERM_BP_ALL	GO:0051605~protein maturation by peptide bond cleavage	1
GOTERM_BP_ALL	GO:0050778~positive regulation of immune response	1
GOTERM_BP_ALL	GO:0009071~serine family amino acid catabolic process	1
GOTERM_BP_ALL	GO:0010383~cell wall polysaccharide metabolic process	1
GOTERM_BP_ALL	GO:0006916~anti-apoptosis	1
GOTERM_BP_ALL	GO:0010104~regulation of ethylene mediated signaling pat	1
GOTERM_BP_ALL	GO:0043647~inositol phosphate metabolic process	1
GOTERM_BP_ALL	GO:0019757~glycosinolate metabolic process	1
GOTERM_BP_ALL	GO:0006022~aminoglycan metabolic process	1
GOTERM_BP_ALL	GO:0008216~spermidine metabolic process	1
GOTERM_BP_ALL	GO:0009627~systemic acquired resistance	1
GOTERM_BP_ALL	GO:0007034~vacuolar transport	1
GOTERM_BP_ALL	GO:0048510~regulation of timing of transition from vegeta	1
GOTERM_BP_ALL	GO:0052249~modulation of RNA levels in other organism	1
GOTERM_BP_ALL	GO:0006566~threonine metabolic process	1
GOTERM_BP_ALL	GO:0007018~microtubule-based movement	1
GOTERM_BP_ALL	GO:0015675~nickel ion transport	1
GOTERM_BP_ALL	GO:0045787~positive regulation of cell cycle	1
GOTERM_BP_ALL	GO:0016143~S-glycoside metabolic process	1
GOTERM_BP_ALL	GO:0000303~response to superoxide	1
GOTERM_BP_ALL	GO:0016570~histone modification	1
GOTERM_BP_ALL	GO:0009132~nucleoside diphosphate metabolic process	1
GOTERM_BP_ALL	GO:0010442~guard cell morphogenesis	1
GOTERM_BP_ALL	GO:0048317~seed morphogenesis	1
GOTERM_BP_ALL	GO:0045292~nuclear mRNA cis splicing, via spliceosome	1
GOTERM_BP_ALL	GO:0070297~regulation of two-component signal transduct	1
GOTERM_BP_ALL	GO:0010361~regulation of anion channel activity by blue li	1
GOTERM_BP_ALL	GO:0042325~regulation of phosphorylation	1
GOTERM_BP_ALL	GO:0010101~post-embryonic root morphogenesis	1
GOTERM_BP_ALL	GO:0009834~secondary cell wall biogenesis	1
GOTERM_BP_ALL	GO:0048578~positive regulation of long-day photoperiodis	1
GOTERM_BP_ALL	GO:0010031~circumnutation	1
GOTERM_BP_ALL	GO:0051409~response to nitrosative stress	1
GOTERM_BP_ALL	GO:0006121~mitochondrial electron transport, succinate to	1
GOTERM_BP_ALL	GO:0008535~respiratory chain complex IV assembly	1

GOTERM_BP_ALL	GO:0010305~leaf vascular tissue pattern formation	1
GOTERM_BP_ALL	GO:0006821~chloride transport	1
GOTERM_BP_ALL	GO:0010087~phloem or xylem histogenesis	1
GOTERM_BP_ALL	GO:0043101~purine salvage	1
GOTERM_BP_ALL	GO:0010223~secondary shoot formation	1
GOTERM_BP_ALL	GO:0009682~induced systemic resistance	1
GOTERM_BP_ALL	GO:0035304~regulation of protein amino acid dephosphory	1
GOTERM_BP_ALL	GO:0046369~galactose biosynthetic process	1
GOTERM_BP_ALL	GO:0008033~tRNA processing	1
GOTERM_BP_ALL	GO:0016119~carotene metabolic process	1
GOTERM_BP_ALL	GO:0030041~actin filament polymerization	1
GOTERM_BP_ALL	GO:0009106~lipoate metabolic process	1
GOTERM_BP_ALL	GO:0019499~cyanide metabolic process	1
GOTERM_BP_ALL	GO:0030203~glycosaminoglycan metabolic process	1
GOTERM_BP_ALL	GO:0043254~regulation of protein complex assembly	1
GOTERM_BP_ALL	GO:0052541~plant-type cell wall cellulose metabolic proce	1
GOTERM_BP_ALL	GO:0015691~cadmium ion transport	1
GOTERM_BP_ALL	GO:0016973~poly(A)+ mRNA export from nucleus	1
GOTERM_BP_ALL	GO:0006081~cellular aldehyde metabolic process	1
GOTERM_BP_ALL	GO:0009875~pollen-pistil interaction	1
GOTERM_BP_ALL	GO:0010080~regulation of floral meristem growth	1
GOTERM_BP_ALL	GO:0043096~purine base salvage	1
GOTERM_BP_ALL	GO:0006270~DNA replication initiation	1
GOTERM_BP_ALL	GO:0032508~DNA duplex unwinding	1
GOTERM_BP_ALL	GO:0006168~adenine salvage	1
GOTERM_BP_ALL	GO:0006021~inositol biosynthetic process	1
GOTERM_BP_ALL	GO:0052542~callose deposition during defense response	1
GOTERM_BP_ALL	GO:0048528~post-embryonic root development	1
GOTERM_BP_ALL	GO:0016102~diterpenoid biosynthetic process	1
GOTERM_BP_ALL	GO:0010102~lateral root morphogenesis	1
GOTERM_BP_ALL	GO:0080120~CAAX-box protein processing	1
GOTERM_BP_ALL	GO:0006415~translational termination	1
GOTERM_BP_ALL	GO:0016131~brassinosteroid metabolic process	1
GOTERM_BP_ALL	GO:0022603~regulation of anatomical structure morphogen	1
GOTERM_BP_ALL	GO:0016122~xanthophyll metabolic process	1
GOTERM_BP_ALL	GO:0015886~heme transport	1
GOTERM_BP_ALL	GO:0051493~regulation of cytoskeleton organization	1
GOTERM_BP_ALL	GO:0051789~response to protein stimulus	1
GOTERM_BP_ALL	GO:0010362~negative regulation of anion channel activity	1
GOTERM_BP_ALL	GO:0010438~cellular response to sulfur starvation	1
GOTERM_BP_ALL	GO:0031347~regulation of defense response	1
GOTERM_BP_ALL	GO:0051168~nuclear export	1
GOTERM_BP_ALL	GO:0009231~riboflavin biosynthetic process	1
GOTERM_BP_ALL	GO:0009273~peptidoglycan-based cell wall biogenesis	1
GOTERM_BP_ALL	GO:0045036~protein targeting to chloroplast	1
GOTERM_BP_ALL	GO:0051051~negative regulation of transport	1
GOTERM_BP_ALL	GO:0019915~lipid storage	1

GOTERM_BP_ALL	GO:0010359~regulation of anion channel activity	1
GOTERM_BP_ALL	GO:0030244~cellulose biosynthetic process	1
GOTERM_BP_ALL	GO:0042128~nitrate assimilation	1
GOTERM_BP_ALL	GO:0043069~negative regulation of programmed cell death	1
GOTERM_BP_ALL	GO:0009410~response to xenobiotic stimulus	1
GOTERM_BP_ALL	GO:0010118~stomatal movement	1
GOTERM_BP_ALL	GO:0009864~induced systemic resistance, jasmonic acid m	1
GOTERM_BP_ALL	GO:0030865~cortical cytoskeleton organization	1
GOTERM_BP_ALL	GO:0005513~detection of calcium ion	1
GOTERM_BP_ALL	GO:0009556~microsporogenesis	1
GOTERM_BP_ALL	GO:0048563~post-embryonic organ morphogenesis	1
GOTERM_BP_ALL	GO:0009788~negative regulation of abscisic acid mediated	1
GOTERM_BP_ALL	GO:0046500~S-adenosylmethionine metabolic process	1
GOTERM_BP_ALL	GO:0034754~cellular hormone metabolic process	1
GOTERM_BP_ALL	GO:0033865~nucleoside bisphosphate metabolic process	1
GOTERM_BP_ALL	GO:0008295~spermidine biosynthetic process	1
GOTERM_BP_ALL	GO:0052544~callose deposition in cell wall during defense	1
GOTERM_BP_ALL	GO:0043650~dicarboxylic acid biosynthetic process	1
GOTERM_BP_ALL	GO:0051817~modification of morphology or physiology of	1
GOTERM_BP_ALL	GO:0042851~L-alanine metabolic process	1
GOTERM_BP_ALL	GO:0009107~lipoate biosynthetic process	1
GOTERM_BP_ALL	GO:0051865~protein autoubiquitination	1
GOTERM_BP_ALL	GO:0006353~transcription termination	1
GOTERM_BP_ALL	GO:0009262~deoxyribonucleotide metabolic process	1
GOTERM_BP_ALL	GO:0019363~pyridine nucleotide biosynthetic process	1
GOTERM_BP_ALL	GO:0005977~glycogen metabolic process	1
GOTERM_BP_ALL	GO:0048532~anatomical structure arrangement	1
GOTERM_BP_ALL	GO:0010584~pollen exine formation	1
GOTERM_BP_ALL	GO:0006792~regulation of sulfur utilization	1
GOTERM_BP_ALL	GO:0006597~spermine biosynthetic process	1
GOTERM_BP_ALL	GO:0015717~triose phosphate transport	1
GOTERM_BP_ALL	GO:0044070~regulation of anion transport	1
GOTERM_BP_ALL	GO:0034762~regulation of transmembrane transport	1
GOTERM_BP_ALL	GO:0006529~asparagine biosynthetic process	1
GOTERM_BP_ALL	GO:0000162~tryptophan biosynthetic process	1
GOTERM_BP_ALL	GO:0032392~DNA geometric change	1
GOTERM_BP_ALL	GO:0010103~stomatal complex morphogenesis	1
GOTERM_BP_ALL	GO:0006722~triterpenoid metabolic process	1
GOTERM_BP_ALL	GO:0000270~peptidoglycan metabolic process	1
GOTERM_BP_ALL	GO:0034284~response to monosaccharide stimulus	1
GOTERM_BP_ALL	GO:0008617~guanosine metabolic process	1
GOTERM_BP_ALL	GO:0050826~response to freezing	1
GOTERM_BP_ALL	GO:0051049~regulation of transport	1
GOTERM_BP_ALL	GO:0031110~regulation of microtubule polymerization or d	1
GOTERM_BP_ALL	GO:0043335~protein unfolding	1
GOTERM_BP_ALL	GO:0005978~glycogen biosynthetic process	1
GOTERM_BP_ALL	GO:0006623~protein targeting to vacuole	1

GOTERM_BP_ALL	GO:0009306~protein secretion	1
GOTERM_BP_ALL	GO:0030036~actin cytoskeleton organization	1
GOTERM_BP_ALL	GO:0009405~pathogenesis	1
GOTERM_BP_ALL	GO:0043086~negative regulation of catalytic activity	1
GOTERM_BP_ALL	GO:0055082~cellular chemical homeostasis	1
GOTERM_BP_ALL	GO:0033037~polysaccharide localization	1
GOTERM_BP_ALL	GO:0032147~activation of protein kinase activity	1
GOTERM_BP_ALL	GO:0042732~D-xylose metabolic process	1
GOTERM_BP_ALL	GO:0000059~protein import into nucleus, docking	1
GOTERM_BP_ALL	GO:0009638~phototropism	1
GOTERM_BP_ALL	GO:0051972~regulation of telomerase activity	1
GOTERM_BP_ALL	GO:0009746~response to hexose stimulus	1
GOTERM_BP_ALL	GO:0009831~plant-type cell wall modification during multi	1
GOTERM_BP_ALL	GO:0006097~glyoxylate cycle	1
GOTERM_BP_ALL	GO:0006000~fructose metabolic process	1
GOTERM_BP_ALL	GO:0009827~plant-type cell wall modification	1
GOTERM_BP_ALL	GO:0046578~regulation of Ras protein signal transduction	1
GOTERM_BP_ALL	GO:0006518~peptide metabolic process	1
GOTERM_BP_ALL	GO:0051259~protein oligomerization	1
GOTERM_BP_ALL	GO:0000038~very-long-chain fatty acid metabolic process	1
GOTERM_BP_ALL	GO:0030422~RNA interference, production of siRNA	1
GOTERM_BP_ALL	GO:0048482~ovule morphogenesis	1
GOTERM_BP_ALL	GO:0019745~pentacyclic triterpenoid biosynthetic process	1
GOTERM_BP_ALL	GO:0051056~regulation of small GTPase mediated signal tr	1
GOTERM_BP_ALL	GO:0010345~suberin biosynthetic process	1
GOTERM_BP_ALL	GO:0009804~coumarin metabolic process	1
GOTERM_BP_ALL	GO:0050994~regulation of lipid catabolic process	1
GOTERM_BP_ALL	GO:0048236~plant-type spore development	1
GOTERM_BP_ALL	GO:0052482~cell wall thickening during defense response	1
GOTERM_BP_ALL	GO:0008037~cell recognition	1
GOTERM_BP_ALL	GO:0010090~trichome morphogenesis	1
GOTERM_BP_ALL	GO:0009113~purine base biosynthetic process	1
GOTERM_BP_ALL	GO:0019760~glucosinolate metabolic process	1
GOTERM_BP_ALL	GO:0042726~riboflavin and derivative metabolic process	1
GOTERM_BP_ALL	GO:0055070~copper ion homeostasis	1
GOTERM_BP_ALL	GO:0042126~nitrate metabolic process	1
GOTERM_BP_ALL	GO:0006984~ER-nuclear signaling pathway	1
GOTERM_BP_ALL	GO:0009769~photosynthesis, light harvesting in photosyste	1
GOTERM_BP_ALL	GO:0008154~actin polymerization or depolymerization	1
GOTERM_BP_ALL	GO:0006914~autophagy	1
GOTERM_BP_ALL	GO:0006805~xenobiotic metabolic process	1
GOTERM_BP_ALL	GO:0032410~negative regulation of transporter activity	1
GOTERM_BP_ALL	GO:0046467~membrane lipid biosynthetic process	1
GOTERM_BP_ALL	GO:0007005~mitochondrion organization	1
GOTERM_BP_ALL	GO:0019761~glucosinolate biosynthetic process	1
GOTERM_BP_ALL	GO:0010675~regulation of cellular carbohydrate metabolic	1
GOTERM_BP_ALL	GO:0030091~protein repair	1

GOTERM_BP_ALL	GO:0035303~regulation of dephosphorylation	1
GOTERM_BP_ALL	GO:0042255~ribosome assembly	1
GOTERM_BP_ALL	GO:0016485~protein processing	1
GOTERM_BP_ALL	GO:0006112~energy reserve metabolic process	1
GOTERM_BP_ALL	GO:0006265~DNA topological change	1
GOTERM_BP_ALL	GO:0045165~cell fate commitment	1
GOTERM_BP_ALL	GO:0009914~hormone transport	1
GOTERM_BP_ALL	GO:0010072~primary shoot apical meristem specification	1
GOTERM_BP_ALL	GO:0006014~D-ribose metabolic process	1
GOTERM_BP_ALL	GO:0009186~deoxyribonucleoside diphosphate metabolic p	1
GOTERM_BP_ALL	GO:0051701~interaction with host	1
GOTERM_BP_ALL	GO:0015749~monosaccharide transport	1
GOTERM_BP_ALL	GO:0045168~cell-cell signaling involved in cell fate specifi	1
GOTERM_BP_ALL	GO:0002253~activation of immune response	1
GOTERM_BP_ALL	GO:0006405~RNA export from nucleus	1
GOTERM_BP_ALL	GO:0019295~coenzyme M biosynthetic process	1
GOTERM_BP_ALL	GO:0046083~adenine metabolic process	1
GOTERM_BP_ALL	GO:0010157~response to chlorate	1
GOTERM_BP_ALL	GO:0019481~L-alanine catabolic process, by transaminatio	1
GOTERM_BP_ALL	GO:0010346~shoot formation	1
GOTERM_BP_ALL	GO:0043549~regulation of kinase activity	1
GOTERM_BP_ALL	GO:0010081~regulation of inflorescence meristem growth	1
GOTERM_BP_ALL	GO:0046487~glyoxylate metabolic process	1
GOTERM_BP_ALL	GO:0031540~regulation of anthocyanin biosynthetic proces	1
GOTERM_BP_ALL	GO:0002684~positive regulation of immune system process	1
GOTERM_BP_ALL	GO:0010089~xylem histogenesis	1
GOTERM_BP_ALL	GO:0009901~anther dehiscence	1
GOTERM_BP_ALL	GO:0032012~regulation of ARF protein signal transduction	1
GOTERM_BP_ALL	GO:0009247~glycolipid biosynthetic process	1
GOTERM_BP_ALL	GO:0015822~ornithine transport	1
GOTERM_BP_ALL	GO:0045488~pectin metabolic process	1
GOTERM_BP_ALL	GO:0002682~regulation of immune system process	1
GOTERM_BP_ALL	GO:0031113~regulation of microtubule polymerization	1
GOTERM_BP_ALL	GO:0009080~pyruvate family amino acid catabolic process	1
GOTERM_BP_ALL	GO:0031333~negative regulation of protein complex assem	1
GOTERM_BP_ALL	GO:0080093~regulation of photorespiration	1
GOTERM_BP_ALL	GO:0045962~positive regulation of development, heterochr	1
GOTERM_BP_ALL	GO:0016144~S-glycoside biosynthetic process	1
GOTERM_BP_ALL	GO:0006749~glutathione metabolic process	1
GOTERM_BP_ALL	GO:0048508~embryonic meristem development	1
GOTERM_BP_ALL	GO:0033043~regulation of organelle organization	1
GOTERM_BP_ALL	GO:0015718~monocarboxylic acid transport	1
GOTERM_BP_ALL	GO:0051973~positive regulation of telomerase activity	1
GOTERM_BP_ALL	GO:0043622~cortical microtubule organization	1
GOTERM_BP_ALL	GO:0010052~guard cell differentiation	1
GOTERM_BP_ALL	GO:0006568~tryptophan metabolic process	1
GOTERM_BP_ALL	GO:0009647~skotomorphogenesis	1

GOTERM_BP_ALL	GO:0048830~adventitious root development	1
GOTERM_BP_ALL	GO:0010670~positive regulation of oxygen and reactive ox	1
GOTERM_BP_ALL	GO:0032957~inositol trisphosphate metabolic process	1
GOTERM_BP_ALL	GO:0010311~lateral root formation	1
GOTERM_BP_ALL	GO:0070298~negative regulation of two-component signal	1
GOTERM_BP_ALL	GO:0006524~alanine catabolic process	1
GOTERM_BP_ALL	GO:0010048~vernalization response	1
GOTERM_BP_ALL	GO:0009593~detection of chemical stimulus	1
GOTERM_BP_ALL	GO:0006003~fructose 2,6-bisphosphate metabolic process	1
GOTERM_BP_ALL	GO:0006801~superoxide metabolic process	1
GOTERM_BP_ALL	GO:0044003~modification by symbiont of host morphology	1
GOTERM_BP_ALL	GO:0051129~negative regulation of cellular component org	1
GOTERM_BP_ALL	GO:0009903~chloroplast avoidance movement	1
GOTERM_BP_ALL	GO:0033674~positive regulation of kinase activity	1
GOTERM_BP_ALL	GO:0052543~callose deposition in cell wall	1
GOTERM_BP_ALL	GO:0009697~salicylic acid biosynthetic process	1
GOTERM_BP_ALL	GO:0042181~ketone biosynthetic process	1
GOTERM_BP_ALL	GO:0022618~ribonucleoprotein complex assembly	1
GOTERM_BP_ALL	GO:0015853~adenine transport	1
GOTERM_BP_ALL	GO:0010207~photosystem II assembly	1
GOTERM_BP_ALL	GO:0009686~gibberellin biosynthetic process	1
GOTERM_BP_ALL	GO:0006771~riboflavin metabolic process	1
GOTERM_BP_ALL	GO:0032880~regulation of protein localization	1
GOTERM_BP_ALL	GO:0010014~meristem initiation	1
GOTERM_BP_ALL	GO:0010051~xylem and phloem pattern formation	1
GOTERM_BP_ALL	GO:0006986~response to unfolded protein	1
GOTERM_BP_ALL	GO:0051592~response to calcium ion	1
GOTERM_BP_ALL	GO:0009423~chorismate biosynthetic process	1
GOTERM_BP_ALL	GO:0009845~seed germination	1
GOTERM_BP_ALL	GO:0043269~regulation of ion transport	1
GOTERM_BP_ALL	GO:0006556~S-adenosylmethionine biosynthetic process	1
GOTERM_BP_ALL	GO:0019374~galactolipid metabolic process	1
GOTERM_BP_ALL	GO:0019216~regulation of lipid metabolic process	1
GOTERM_BP_ALL	GO:0045860~positive regulation of protein kinase activity	1
GOTERM_BP_ALL	GO:0010091~trichome branching	1
GOTERM_BP_ALL	GO:0010105~negative regulation of ethylene mediated sign	1
GOTERM_BP_ALL	GO:0008215~spermine metabolic process	1
GOTERM_BP_ALL	GO:0010044~response to aluminum ion	1
GOTERM_BP_ALL	GO:0034620~cellular response to unfolded protein	1
GOTERM_BP_ALL	GO:0006879~cellular iron ion homeostasis	1
GOTERM_BP_ALL	GO:0045595~regulation of cell differentiation	1
GOTERM_BP_ALL	GO:0051193~regulation of cofactor metabolic process	1
GOTERM_BP_ALL	GO:0010927~cellular component assembly involved in mor	1
GOTERM_BP_ALL	GO:0009750~response to fructose stimulus	1
GOTERM_BP_ALL	GO:0051181~cofactor transport	1
GOTERM_BP_ALL	GO:0031115~negative regulation of microtubule polymeriz	1
GOTERM_BP_ALL	GO:0006741~NADP biosynthetic process	1

GOTERM_BP_ALL	GO:0009900~dehiscence	1
GOTERM_BP_ALL	GO:0031349~positive regulation of defense response	1
GOTERM_BP_ALL	GO:0019827~stem cell maintenance	1
GOTERM_BP_ALL	GO:0006366~transcription from RNA polymerase II promo	1
GOTERM_BP_ALL	GO:0010382~cellular cell wall macromolecule metabolic pr	1
GOTERM_BP_ALL	GO:0009828~plant-type cell wall loosening	1
GOTERM_BP_ALL	GO:0070507~regulation of microtubule cytoskeleton organi	1
GOTERM_BP_ALL	GO:0048506~regulation of timing of meristematic phase tra	1
GOTERM_BP_ALL	GO:0048527~lateral root development	1
GOTERM_BP_ALL	GO:0010441~guard cell development	1
GOTERM_BP_ALL	GO:0030104~water homeostasis	1
GOTERM_BP_ALL	GO:0006122~mitochondrial electron transport, ubiquinol to	1
GOTERM_BP_ALL	GO:0009251~glucan catabolic process	1
GOTERM_BP_ALL	GO:0009805~coumarin biosynthetic process	1
GOTERM_BP_ALL	GO:0009904~chloroplast accumulation movement	1
GOTERM_BP_ALL	GO:0032409~regulation of transporter activity	1
GOTERM_BP_ALL	GO:0034605~cellular response to heat	1
GOTERM_BP_ALL	GO:0010243~response to organic nitrogen	1
GOTERM_BP_ALL	GO:0009821~alkaloid biosynthetic process	1
GOTERM_BP_ALL	GO:0043617~cellular response to sucrose starvation	1
GOTERM_BP_ALL	GO:0042549~photosystem II stabilization	1
GOTERM_CC_ALL	GO:0045254~pyruvate dehydrogenase complex	1
GOTERM_CC_ALL	GO:0005789~endoplasmic reticulum membrane	1
GOTERM_CC_ALL	GO:0009783~photosystem II antenna complex	1
GOTERM_CC_ALL	GO:0009842~cyanelle	1
GOTERM_CC_ALL	GO:0031982~vesicle	1
GOTERM_CC_ALL	GO:0005750~mitochondrial respiratory chain complex III	1
GOTERM_CC_ALL	GO:0012511~monolayer-surrounded lipid storage body	1
GOTERM_CC_ALL	GO:0005971~ribonucleoside-diphosphate reductase comple	1
GOTERM_CC_ALL	GO:0009986~cell surface	1
GOTERM_CC_ALL	GO:0000267~cell fraction	1
GOTERM_CC_ALL	GO:0000922~spindle pole	1
GOTERM_CC_ALL	GO:0009503~thylakoid light-harvesting complex	1
GOTERM_CC_ALL	GO:0042175~nuclear envelope-endoplasmic reticulum netw	1
GOTERM_CC_ALL	GO:0009538~photosystem I reaction center	1
GOTERM_CC_ALL	GO:0009897~external side of plasma membrane	1
GOTERM_CC_ALL	GO:0044438~microbody part	1
GOTERM_CC_ALL	GO:0031300~intrinsic to organelle membrane	1
GOTERM_CC_ALL	GO:0005911~cell-cell junction	1
GOTERM_CC_ALL	GO:0044454~nuclear chromosome part	1
GOTERM_CC_ALL	GO:0045273~respiratory chain complex II	1
GOTERM_CC_ALL	GO:0005782~peroxisomal matrix	1
GOTERM_CC_ALL	GO:0044423~virion part	1
GOTERM_CC_ALL	GO:0005852~eukaryotic translation initiation factor 3 comp	1
GOTERM_CC_ALL	GO:0005667~transcription factor complex	1
GOTERM_CC_ALL	GO:0030093~chloroplast photosystem I	1
GOTERM_CC_ALL	GO:0009524~phragmoplast	1

GOTERM_CC_ALL	GO:0009346~citrate lyase complex	1
GOTERM_CC_ALL	GO:0005626~insoluble fraction	1
GOTERM_CC_ALL	GO:0009501~amyloplast	1
GOTERM_CC_ALL	GO:0009517~PSII associated light-harvesting complex II	1
GOTERM_CC_ALL	GO:0045263~proton-transporting ATP synthase complex, c	1
GOTERM_CC_ALL	GO:0048492~ribulose bisphosphate carboxylase complex	1
GOTERM_CC_ALL	GO:0032040~small-subunit processome	1
GOTERM_CC_ALL	GO:0042598~vesicular fraction	1
GOTERM_CC_ALL	GO:0009504~cell plate	1
GOTERM_CC_ALL	GO:0045261~proton-transporting ATP synthase complex, c	1
GOTERM_CC_ALL	GO:0005960~glycine cleavage complex	1
GOTERM_CC_ALL	GO:0080137~DNA-directed RNA polymerase V complex	1
GOTERM_CC_ALL	GO:0009317~acetyl-CoA carboxylase complex	1
GOTERM_CC_ALL	GO:0010007~magnesium chelatase complex	1
GOTERM_CC_ALL	GO:0009512~cytochrome b6f complex	1
GOTERM_CC_ALL	GO:0005819~spindle	1
GOTERM_CC_ALL	GO:0031907~microbody lumen	1
GOTERM_CC_ALL	GO:0005753~mitochondrial proton-transporting ATP synth	1
GOTERM_CC_ALL	GO:0005792~microsome	1
GOTERM_CC_ALL	GO:0005624~membrane fraction	1
GOTERM_CC_ALL	GO:0005815~microtubule organizing center	1
GOTERM_CC_ALL	GO:0030532~small nuclear ribonucleoprotein complex	1
GOTERM_CC_ALL	GO:0009341~beta-galactosidase complex	1
GOTERM_CC_ALL	GO:0000228~nuclear chromosome	1
GOTERM_CC_ALL	GO:0009898~internal side of plasma membrane	1
GOTERM_CC_ALL	GO:0045281~succinate dehydrogenase complex	1
GOTERM_CC_ALL	GO:0000418~DNA-directed RNA polymerase IV complex	1
GOTERM_CC_ALL	GO:0033179~proton-transporting V-type ATPase, V0 doma	1
GOTERM_CC_ALL	GO:0000811~GINS complex	1
GOTERM_CC_ALL	GO:0033176~proton-transporting V-type ATPase complex	1
GOTERM_CC_ALL	GO:0005768~endosome	1
GOTERM_CC_ALL	GO:0005686~snRNP U2	1
GOTERM_CC_ALL	GO:0030684~preribosome	1
GOTERM_CC_ALL	GO:0030964~NADH dehydrogenase complex	1
GOTERM_CC_ALL	GO:0030054~cell junction	1
GOTERM_CC_ALL	GO:0005746~mitochondrial respiratory chain	1
GOTERM_CC_ALL	GO:0045271~respiratory chain complex I	1
GOTERM_CC_ALL	GO:0010169~thioglucosidase complex	1
GOTERM_CC_ALL	GO:0045275~respiratory chain complex III	1
GOTERM_CC_ALL	GO:0031304~intrinsic to mitochondrial inner membrane	1
GOTERM_CC_ALL	GO:0009533~chloroplast stromal thylakoid	1
GOTERM_CC_ALL	GO:0031312~extrinsic to organelle membrane	1
GOTERM_CC_ALL	GO:0019028~viral capsid	1
GOTERM_CC_ALL	GO:0005811~lipid particle	1
GOTERM_CC_ALL	GO:0016602~CCAAT-binding factor complex	1
GOTERM_CC_ALL	GO:0034707~chloride channel complex	1
GOTERM_CC_ALL	GO:0044439~peroxisomal part	1

GOTERM_CC_ALL	GO:0009514~glyoxysome	1
GOTERM_CC_ALL	GO:0019012~virion	1
GOTERM_CC_ALL	GO:0042406~extrinsic to endoplasmic reticulum membrane	1
GOTERM_CC_ALL	GO:0008287~protein serine/threonine phosphatase complex	1
GOTERM_CC_ALL	GO:0005853~eukaryotic translation elongation factor 1 com	1
GOTERM_CC_ALL	GO:0009295~nucleoid	1
GOTERM_CC_ALL	GO:0009573~chloroplast ribulose bisphosphate carboxylase	1
GOTERM_CC_ALL	GO:0034702~ion channel complex	1
GOTERM_CC_ALL	GO:0010240~plastid pyruvate dehydrogenase complex	1
GOTERM_MF_ALL	GO:0019904~protein domain specific binding	1
GOTERM_MF_ALL	GO:0010472~GDP-galactose:glucose-1-phosphate guanyltr	1
GOTERM_MF_ALL	GO:0016725~oxidoreductase activity, acting on CH or CH2	1
GOTERM_MF_ALL	GO:0051184~cofactor transporter activity	1
GOTERM_MF_ALL	GO:0004747~ribokinase activity	1
GOTERM_MF_ALL	GO:0047558~3-cyanoalanine hydratase activity	1
GOTERM_MF_ALL	GO:0015116~sulfate transmembrane transporter activity	1
GOTERM_MF_ALL	GO:0033840~NDP-glucose-starch glucosyltransferase activ	1
GOTERM_MF_ALL	GO:0016615~malate dehydrogenase activity	1
GOTERM_MF_ALL	GO:0019840~isoprenoid binding	1
GOTERM_MF_ALL	GO:0009979~16:0 monogalactosyldiacylglycerol desaturas	1
GOTERM_MF_ALL	GO:0005247~voltage-gated chloride channel activity	1
GOTERM_MF_ALL	GO:0050278~sedoheptulose-bisphosphatase activity	1
GOTERM_MF_ALL	GO:0035197~siRNA binding	1
GOTERM_MF_ALL	GO:0031202~RNA splicing factor activity, transesterificati	1
GOTERM_MF_ALL	GO:0008970~phospholipase A1 activity	1
GOTERM_MF_ALL	GO:0016149~translation release factor activity, codon speci	1
GOTERM_MF_ALL	GO:0005088~Ras guanyl-nucleotide exchange factor activit	1
GOTERM_MF_ALL	GO:0004802~transketolase activity	1
GOTERM_MF_ALL	GO:0003680~AT DNA binding	1
GOTERM_MF_ALL	GO:0016857~racemase and epimerase activity, acting on ca	1
GOTERM_MF_ALL	GO:0047325~inositol tetrakisphosphate 1-kinase activity	1
GOTERM_MF_ALL	GO:0016861~intramolecular oxidoreductase activity, interc	1
GOTERM_MF_ALL	GO:0004105~choline-phosphate cytidylyltransferase activit	1
GOTERM_MF_ALL	GO:0042277~peptide binding	1
GOTERM_MF_ALL	GO:0010291~carotene beta-ring hydroxylase activity	1
GOTERM_MF_ALL	GO:0004723~calcium-dependent protein serine/threonine p	1
GOTERM_MF_ALL	GO:0016854~racemase and epimerase activity	1
GOTERM_MF_ALL	GO:0042973~glucan endo-1,3-beta-D-glucosidase activity	1
GOTERM_MF_ALL	GO:0004353~glutamate dehydrogenase [NAD(P)+] activity	1
GOTERM_MF_ALL	GO:0004396~hexokinase activity	1
GOTERM_MF_ALL	GO:0048529~magnesium-protoporphyrin IX monomethyl e	1
GOTERM_MF_ALL	GO:0004096~catalase activity	1
GOTERM_MF_ALL	GO:0010294~abscisic acid glucosyltransferase activity	1
GOTERM_MF_ALL	GO:0005086~ARF guanyl-nucleotide exchange factor activ	1
GOTERM_MF_ALL	GO:0003682~chromatin binding	1
GOTERM_MF_ALL	GO:0046905~phytoene synthase activity	1
GOTERM_MF_ALL	GO:0022836~gated channel activity	1

GOTERM_MF_ALL	GO:0008113~peptide-methionine-(S)-S-oxide reductase act	1
GOTERM_MF_ALL	GO:0004020~adenylylsulfate kinase activity	1
GOTERM_MF_ALL	GO:0008417~fucosyltransferase activity	1
GOTERM_MF_ALL	GO:0051748~UTP-monosaccharide-1-phosphate uridylyltra	1
GOTERM_MF_ALL	GO:0004028~3-chloroallyl aldehyde dehydrogenase activit	1
GOTERM_MF_ALL	GO:0008825~cyclopropane-fatty-acyl-phospholipid synthas	1
GOTERM_MF_ALL	GO:0016805~dipeptidase activity	1
GOTERM_MF_ALL	GO:0008878~glucose-1-phosphate adenylyltransferase activ	1
GOTERM_MF_ALL	GO:0003690~double-stranded DNA binding	1
GOTERM_MF_ALL	GO:0051538~3 iron, 4 sulfur cluster binding	1
GOTERM_MF_ALL	GO:0005484~SNAP receptor activity	1
GOTERM_MF_ALL	GO:0051003~ligase activity, forming nitrogen-metal bonds,	1
GOTERM_MF_ALL	GO:0008308~voltage-gated anion channel activity	1
GOTERM_MF_ALL	GO:0009882~blue light photoreceptor activity	1
GOTERM_MF_ALL	GO:0042736~NADH kinase activity	1
GOTERM_MF_ALL	GO:0016760~cellulose synthase (UDP-forming) activity	1
GOTERM_MF_ALL	GO:0046524~sucrose-phosphate synthase activity	1
GOTERM_MF_ALL	GO:0016843~amine-lyase activity	1
GOTERM_MF_ALL	GO:0017089~glycolipid transporter activity	1
GOTERM_MF_ALL	GO:0080061~indole-3-acetonitrile nitrilase activity	1
GOTERM_MF_ALL	GO:0008883~glutamyl-tRNA reductase activity	1
GOTERM_MF_ALL	GO:0004526~ribonuclease P activity	1
GOTERM_MF_ALL	GO:0035198~miRNA binding	1
GOTERM_MF_ALL	GO:0015099~nickel ion transmembrane transporter activity	1
GOTERM_MF_ALL	GO:0047427~cyanoalanine nitrilase activity	1
GOTERM_MF_ALL	GO:0043531~ADP binding	1
GOTERM_MF_ALL	GO:0000257~nitrilase activity	1
GOTERM_MF_ALL	GO:0016752~sinapoyltransferase activity	1
GOTERM_MF_ALL	GO:0003978~UDP-glucose 4-epimerase activity	1
GOTERM_MF_ALL	GO:0032549~ribonucleoside binding	1
GOTERM_MF_ALL	GO:0008705~methionine synthase activity	1
GOTERM_MF_ALL	GO:0016681~oxidoreductase activity, acting on diphenols a	1
GOTERM_MF_ALL	GO:0043021~ribonucleoprotein binding	1
GOTERM_MF_ALL	GO:0003746~translation elongation factor activity	1
GOTERM_MF_ALL	GO:0008536~Ran GTPase binding	1
GOTERM_MF_ALL	GO:0008898~homocysteine S-methyltransferase activity	1
GOTERM_MF_ALL	GO:0016160~amylase activity	1
GOTERM_MF_ALL	GO:0004748~ribonucleoside-diphosphate reductase activity	1
GOTERM_MF_ALL	GO:0050403~trans-zeatin O-beta-D-glucosyltransferase acti	1
GOTERM_MF_ALL	GO:0046480~galactolipid galactosyltransferase activity	1
GOTERM_MF_ALL	GO:0003914~DNA (6-4) photolyase activity	1
GOTERM_MF_ALL	GO:0033613~transcription activator binding	1
GOTERM_MF_ALL	GO:0080062~cytokinin 9-beta-glucosyltransferase activity	1
GOTERM_MF_ALL	GO:0015386~potassium:hydrogen antiporter activity	1
GOTERM_MF_ALL	GO:0043168~anion binding	1
GOTERM_MF_ALL	GO:0016780~phosphotransferase activity, for other substitu	1
GOTERM_MF_ALL	GO:0016844~strictosidine synthase activity	1

GOTERM_MF_ALL	GO:0070456~galactose-1-phosphate phosphatase activity	1
GOTERM_MF_ALL	GO:0016639~oxidoreductase activity, acting on the CH-NH	1
GOTERM_MF_ALL	GO:0008107~galactoside 2-alpha-L-fucosyltransferase activ	1
GOTERM_MF_ALL	GO:0008686~3,4-dihydroxy-2-butanone-4-phosphate synth	1
GOTERM_MF_ALL	GO:0035300~inositol-1,3,4-trisphosphate 5/6-kinase activit	1
GOTERM_MF_ALL	GO:0016759~cellulose synthase activity	1
GOTERM_MF_ALL	GO:0051766~inositol trisphosphate kinase activity	1
GOTERM_MF_ALL	GO:0004612~phosphoenolpyruvate carboxykinase (ATP) a	1
GOTERM_MF_ALL	GO:0008429~phosphatidylethanolamine binding	1
GOTERM_MF_ALL	GO:0070566~adenylyltransferase activity	1
GOTERM_MF_ALL	GO:0003713~transcription coactivator activity	1
GOTERM_MF_ALL	GO:0000036~acyl carrier activity	1
GOTERM_MF_ALL	GO:0005253~anion channel activity	1
GOTERM_MF_ALL	GO:0009045~xylose isomerase activity	1
GOTERM_MF_ALL	GO:0070283~radical SAM enzyme activity	1
GOTERM_MF_ALL	GO:0010242~oxygen evolving activity	1
GOTERM_MF_ALL	GO:0008905~mannose-phosphate guanylyltransferase activ	1
GOTERM_MF_ALL	GO:0004103~choline kinase activity	1
GOTERM_MF_ALL	GO:0008276~protein methyltransferase activity	1
GOTERM_MF_ALL	GO:0046409~p-coumarate 3-hydroxylase activity	1
GOTERM_MF_ALL	GO:0003873~6-phosphofructo-2-kinase activity	1
GOTERM_MF_ALL	GO:0004033~aldo-keto reductase activity	1
GOTERM_MF_ALL	GO:0070569~uridylyltransferase activity	1
GOTERM_MF_ALL	GO:0003885~D-arabinono-1,4-lactone oxidase activity	1
GOTERM_MF_ALL	GO:0031559~oxidosqualene cyclase activity	1
GOTERM_MF_ALL	GO:0046912~transferase activity, transferring acyl groups,	1
GOTERM_MF_ALL	GO:0008138~protein tyrosine/serine/threonine phosphatase	1
GOTERM_MF_ALL	GO:0016813~hydrolase activity, acting on carbon-nitrogen	1
GOTERM_MF_ALL	GO:0051861~glycolipid binding	1
GOTERM_MF_ALL	GO:0080045~quercetin 3'-O-glucosyltransferase activity	1
GOTERM_MF_ALL	GO:0048038~quinone binding	1
GOTERM_MF_ALL	GO:0008934~inositol-1(or 4)-monophosphatase activity	1
GOTERM_MF_ALL	GO:0015298~solute:cation antiporter activity	1
GOTERM_MF_ALL	GO:0004053~arginase activity	1
GOTERM_MF_ALL	GO:0000250~lanosterol synthase activity	1
GOTERM_MF_ALL	GO:0051765~inositol tetrakisphosphate kinase activity	1
GOTERM_MF_ALL	GO:0004176~ATP-dependent peptidase activity	1
GOTERM_MF_ALL	GO:0016814~hydrolase activity, acting on carbon-nitrogen	1
GOTERM_MF_ALL	GO:0016778~diphosphotransferase activity	1
GOTERM_MF_ALL	GO:0008441~3'(2'),5'-bisphosphate nucleotidase activity	1
GOTERM_MF_ALL	GO:0016992~lipoate synthase activity	1
GOTERM_MF_ALL	GO:0016801~hydrolase activity, acting on ether bonds	1
GOTERM_MF_ALL	GO:0004335~galactokinase activity	1
GOTERM_MF_ALL	GO:0010180~thioglucosidase binding	1
GOTERM_MF_ALL	GO:0016767~geranylgeranyl-diphosphate geranylgeranyltra	1
GOTERM_MF_ALL	GO:0016851~magnesium chelatase activity	1
GOTERM_MF_ALL	GO:0004437~inositol or phosphatidylinositol phosphatase a	1

GOTERM_MF_ALL	GO:0016860~intramolecular oxidoreductase activity	1
GOTERM_MF_ALL	GO:0050897~cobalt ion binding	1
GOTERM_MF_ALL	GO:0045550~geranylgeranyl reductase activity	1
GOTERM_MF_ALL	GO:0003747~translation release factor activity	1
GOTERM_MF_ALL	GO:0046863~ribulose-1,5-bisphosphate carboxylase/oxyge	1
GOTERM_MF_ALL	GO:0005254~chloride channel activity	1
GOTERM_MF_ALL	GO:0033765~steroid dehydrogenase activity, acting on the	1
GOTERM_MF_ALL	GO:0003917~DNA topoisomerase type I activity	1
GOTERM_MF_ALL	GO:0008526~phosphatidylinositol transporter activity	1
GOTERM_MF_ALL	GO:0043022~ribosome binding	1
GOTERM_MF_ALL	GO:0008865~fructokinase activity	1
GOTERM_MF_ALL	GO:0042162~telomeric DNA binding	1
GOTERM_MF_ALL	GO:0031267~small GTPase binding	1
GOTERM_MF_ALL	GO:0016979~lipoate-protein ligase activity	1
GOTERM_MF_ALL	GO:0016279~protein-lysine N-methyltransferase activity	1
GOTERM_MF_ALL	GO:0004106~chorismate mutase activity	1
GOTERM_MF_ALL	GO:0015645~fatty-acid ligase activity	1
GOTERM_MF_ALL	GO:0005375~copper ion transmembrane transporter activit	1
GOTERM_MF_ALL	GO:0003865~3-oxo-5-alpha-steroid 4-dehydrogenase activi	1
GOTERM_MF_ALL	GO:0016278~lysine N-methyltransferase activity	1
GOTERM_MF_ALL	GO:0016763~transferase activity, transferring pentosyl grou	1
GOTERM_MF_ALL	GO:0005089~Rho guanyl-nucleotide exchange factor activi	1
GOTERM_MF_ALL	GO:0008728~GTP diphosphokinase activity	1
GOTERM_MF_ALL	GO:0042085~5-methyltetrahydropteroyltri-L-glutamate-dep	1
GOTERM_MF_ALL	GO:0031418~L-ascorbic acid binding	1
GOTERM_MF_ALL	GO:0004462~lactoylglutathione lyase activity	1
GOTERM_MF_ALL	GO:0050827~toxin receptor binding	1
GOTERM_MF_ALL	GO:0004427~inorganic diphosphatase activity	1
GOTERM_MF_ALL	GO:0004252~serine-type endopeptidase activity	1
GOTERM_MF_ALL	GO:0016642~oxidoreductase activity, acting on the CH-NH	1
GOTERM_MF_ALL	GO:0003983~UTP:glucose-1-phosphate uridylyltransferase	1
GOTERM_MF_ALL	GO:0000104~succinate dehydrogenase activity	1
GOTERM_MF_ALL	GO:0004013~adenosylhomocysteinase activity	1
GOTERM_MF_ALL	GO:0000210~NAD+ diphosphatase activity	1
GOTERM_MF_ALL	GO:0008252~nucleotidase activity	1
GOTERM_MF_ALL	GO:0008187~poly-pyrimidine tract binding	1
GOTERM_MF_ALL	GO:0010471~GDP-galactose:mannose-1-phosphate guanylt	1
GOTERM_MF_ALL	GO:0004478~methionine adenosyltransferase activity	1
GOTERM_MF_ALL	GO:0003691~double-stranded telomeric DNA binding	1
GOTERM_MF_ALL	GO:0003973~(S)-2-hydroxy-acid oxidase activity	1
GOTERM_MF_ALL	GO:0015145~monosaccharide transmembrane transporter a	1
GOTERM_MF_ALL	GO:0004321~fatty-acyl-CoA synthase activity	1
GOTERM_MF_ALL	GO:0047958~glycine:2-oxoglutarate aminotransferase activ	1
GOTERM_MF_ALL	GO:0004549~tRNA-specific ribonuclease activity	1
GOTERM_MF_ALL	GO:0009374~biotin binding	1
GOTERM_MF_ALL	GO:0004352~glutamate dehydrogenase activity	1
GOTERM_MF_ALL	GO:0008453~alanine-glyoxylate transaminase activity	1

GOTERM_MF_ALL	GO:0031957~very-long-chain-fatty-acid-CoA ligase activit	1
GOTERM_MF_ALL	GO:0008266~poly(U) RNA binding	1
GOTERM_MF_ALL	GO:0003715~transcription termination factor activity	1
GOTERM_MF_ALL	GO:0045486~naringenin 3-dioxygenase activity	1
GOTERM_MF_ALL	GO:0003924~GTPase activity	1
GOTERM_MF_ALL	GO:0004022~alcohol dehydrogenase (NAD) activity	1
GOTERM_MF_ALL	GO:0050502~cis-zeatin O-beta-D-glucosyltransferase activi	1
GOTERM_MF_ALL	GO:0008271~secondary active sulfate transmembrane trans	1
GOTERM_MF_ALL	GO:0016210~naringenin-chalcone synthase activity	1
GOTERM_MF_ALL	GO:0015232~heme transporter activity	1
GOTERM_MF_ALL	GO:0004806~triacylglycerol lipase activity	1
GOTERM_MF_ALL	GO:0051738~xanthophyll binding	1
GOTERM_MF_ALL	GO:0022832~voltage-gated channel activity	1
GOTERM_MF_ALL	GO:0016713~oxidoreductase activity, acting on paired don	1
GOTERM_MF_ALL	GO:0004331~fructose-2,6-bisphosphate 2-phosphatase acti	1
GOTERM_MF_ALL	GO:0042284~sphingolipid delta-4 desaturase activity	1
GOTERM_MF_ALL	GO:0050136~NADH dehydrogenase (quinone) activity	1
GOTERM_MF_ALL	GO:0080039~xyloglucan endotransglucosylase activity	1
GOTERM_MF_ALL	GO:0051020~GTPase binding	1
GOTERM_MF_ALL	GO:0051920~peroxiredoxin activity	1
GOTERM_MF_ALL	GO:0035250~UDP-galactosyltransferase activity	1
GOTERM_MF_ALL	GO:0017140~lipoic acid synthase activity	1
GOTERM_MF_ALL	GO:0016655~oxidoreductase activity, acting on NADH or	1
GOTERM_MF_ALL	GO:0004791~thioredoxin-disulfide reductase activity	1
GOTERM_MF_ALL	GO:0043566~structure-specific DNA binding	1
GOTERM_MF_ALL	GO:0050347~trans-octaprenyltranstransferase activity	1
GOTERM_MF_ALL	GO:0008443~phosphofructokinase activity	1
GOTERM_MF_ALL	GO:0008891~glycolate oxidase activity	1
GOTERM_MF_ALL	GO:0008887~glycerate kinase activity	1
GOTERM_MF_ALL	GO:0003999~adenine phosphoribosyltransferase activity	1
GOTERM_MF_ALL	GO:0004467~long-chain-fatty-acid-CoA ligase activity	1
GOTERM_MF_ALL	GO:0031404~chloride ion binding	1
GOTERM_MF_ALL	GO:0003989~acetyl-CoA carboxylase activity	1
GOTERM_MF_ALL	GO:0008374~O-acyltransferase activity	1
GOTERM_MF_ALL	GO:0016628~oxidoreductase activity, acting on the CH-CH	1
GOTERM_MF_ALL	GO:0018822~nitrile hydratase activity	1
GOTERM_MF_ALL	GO:0010474~glucose-1-phosphate guanylyltransferase (GD	1
GOTERM_MF_ALL	GO:0016688~L-ascorbate peroxidase activity	1
GOTERM_MF_ALL	GO:0047714~galactolipase activity	1
GOTERM_MF_ALL	GO:0000062~acyl-CoA binding	1
GOTERM_MF_ALL	GO:0004618~phosphoglycerate kinase activity	1
GOTERM_MF_ALL	GO:0004311~farnesyltranstransferase activity	1
GOTERM_MF_ALL	GO:0017016~Ras GTPase binding	1
GOTERM_MF_ALL	GO:0003935~GTP cyclohydrolase II activity	1
GOTERM_MF_ALL	GO:0005244~voltage-gated ion channel activity	1
GOTERM_MF_ALL	GO:0004435~phosphoinositide phospholipase C activity	1
GOTERM_MF_ALL	GO:0003916~DNA topoisomerase activity	1

GOTERM_MF_ALL	GO:0010347~L-galactose-1-phosphate phosphatase activity	1
GOTERM_MF_ALL	GO:0008928~mannose-1-phosphate guanylyltransferase (G	1
GOTERM_MF_ALL	GO:0045158~electron transporter, transferring electrons wi	1
GOTERM_MF_ALL	GO:0003871~5-methyltetrahydropteroyltriglutamate-homoc	1
GOTERM_MF_ALL	GO:0042084~5-methyltetrahydrofolate-dependent methyltr	1
GOTERM_MF_ALL	GO:0016709~oxidoreductase activity, acting on paired don	1
GOTERM_MF_ALL	GO:0031409~pigment binding	1
GOTERM_MF_ALL	GO:0080133~midchain alkane hydroxylase activity	1
GOTERM_MF_ALL	GO:0003984~acetolactate synthase activity	1
GOTERM_MF_ALL	GO:0017050~D-erythro-sphingosine kinase activity	1
GOTERM_MF_ALL	GO:0030060~L-malate dehydrogenase activity	1
GOTERM_MF_ALL	GO:0032934~sterol binding	1
GOTERM_MF_ALL	GO:0008235~metalloexopeptidase activity	1
GOTERM_MF_ALL	GO:0015079~potassium ion transmembrane transporter acti	1
GOTERM_MF_ALL	GO:0070567~cytidylyltransferase activity	1
GOTERM_MF_ALL	GO:0016885~ligase activity, forming carbon-carbon bonds	1
GOTERM_MF_ALL	GO:0051002~ligase activity, forming nitrogen-metal bonds	1
GOTERM_MF_ALL	GO:0004181~metallocarboxypeptidase activity	1
GOTERM_MF_ALL	GO:0004712~protein serine/threonine/tyrosine kinase activi	1
GOTERM_MF_ALL	GO:0022821~potassium ion antiporter activity	1
GOTERM_MF_ALL	GO:0004683~calmodulin-dependent protein kinase activity	1
GOTERM_MF_ALL	GO:0032182~small conjugating protein binding	1
GOTERM_MF_ALL	GO:0016229~steroid dehydrogenase activity	1
GOTERM_MF_ALL	GO:0008137~NADH dehydrogenase (ubiquinone) activity	1
GOTERM_MF_ALL	GO:0047800~cysteamine dioxygenase activity	1
GOTERM_MF_ALL	GO:0008119~thiopurine S-methyltransferase activity	1
GOTERM_MF_ALL	GO:0015300~solute:solute antiporter activity	1
GOTERM_MF_ALL	GO:0016728~oxidoreductase activity, acting on CH or CH2	1
GOTERM_MF_ALL	GO:0008121~ubiquinol-cytochrome-c reductase activity	1
GOTERM_MF_ALL	GO:0018024~histone-lysine N-methyltransferase activity	1
GOTERM_MF_ALL	GO:0004072~aspartate kinase activity	1
GOTERM_MF_ALL	GO:0016802~trialkylsulfonium hydrolase activity	1
GOTERM_MF_ALL	GO:0008177~succinate dehydrogenase (ubiquinone) activit	1
GOTERM_MF_ALL	GO:0042389~omega-3 fatty acid desaturase activity	1
GOTERM_MF_ALL	GO:0016987~sigma factor activity	1
GOTERM_MF_ALL	GO:0016836~hydro-lyase activity	1
GOTERM_MF_ALL	GO:0005384~manganese ion transmembrane transporter act	1
GOTERM_MF_ALL	GO:0008810~cellulase activity	1
GOTERM_MF_ALL	GO:0017077~oxidative phosphorylation uncoupler activity	1
GOTERM_MF_ALL	GO:0016161~beta-amylase activity	1
GOTERM_MF_ALL	GO:0016884~carbon-nitrogen ligase activity, with glutamin	1
GOTERM_MF_ALL	GO:0008963~phospho-N-acetylmuramoyl-pentapeptide-tra	1
GOTERM_MF_ALL	GO:0010473~GDP-galactose:myoinositol-1-phosphate guan	1
GOTERM_MF_ALL	GO:0043130~ubiquitin binding	1
GOTERM_MF_ALL	GO:0042054~histone methyltransferase activity	1
GOTERM_MF_ALL	GO:0008964~phosphoenolpyruvate carboxylase activity	1
GOTERM_MF_ALL	GO:0019139~cytokinin dehydrogenase activity	1

GOTERM_MF_ALL	GO:0004148~dihydrolipoyl dehydrogenase activity	1
GOTERM_MF_ALL	GO:0050664~oxidoreductase activity, acting on NADH or	1
GOTERM_MF_ALL	GO:0004161~dimethylallyltranstransferase activity	1
GOTERM_MF_ALL	GO:0015174~basic amino acid transmembrane transporter a	1
GOTERM_MF_ALL	GO:0030794~(S)-coclaurine-N-methyltransferase activity	1
GOTERM_MF_ALL	GO:0005504~fatty acid binding	1
GOTERM_MF_ALL	GO:0003933~GTP cyclohydrolase activity	1
GOTERM_MF_ALL	GO:0005548~phospholipid transporter activity	1
GOTERM_MF_ALL	GO:0047213~anthocyanidin 3-O-glucosyltransferase activit	1
GOTERM_MF_ALL	GO:0008142~oxysterol binding	1
GOTERM_MF_ALL	GO:0050284~sinapate 1-glucosyltransferase activity	1
GOTERM_MF_ALL	GO:0016635~oxidoreductase activity, acting on the CH-CH	1
GOTERM_MF_ALL	GO:0043295~glutathione binding	1
GOTERM_MF_ALL	GO:0016754~sinapoylglucose-malate O-sinapoyltransferase	1
GOTERM_MF_ALL	GO:0009670~triose-phosphate transmembrane transporter a	1
GOTERM_MF_ALL	GO:0015491~cation:cation antiporter activity	1
GOTERM_MF_ALL	GO:0000339~RNA cap binding	1
GOTERM_MF_ALL	GO:0047066~phospholipid-hydroperoxide glutathione pero	1
GOTERM_MF_ALL	GO:0016753~O-sinapoyltransferase activity	1
GOTERM_MF_ALL	GO:0008170~N-methyltransferase activity	1
GOTERM_MF_ALL	GO:0003962~cystathionine gamma-synthase activity	1
GOTERM_MF_ALL	GO:0004629~phospholipase C activity	1
GOTERM_MF_ALL	GO:0003866~3-phosphoshikimate 1-carboxyvinyltransferas	1
GOTERM_MF_ALL	GO:0003727~single-stranded RNA binding	1
GOTERM_MF_ALL	GO:0004014~adenosylmethionine decarboxylase activity	1
GOTERM_MF_ALL	GO:0016408~C-acyltransferase activity	1
GOTERM_MF_ALL	GO:0015299~solute:hydrogen antiporter activity	1
GOTERM_MF_ALL	GO:0008079~translation termination factor activity	1
GOTERM_MF_ALL	GO:0046481~digalactosyldiacylglycerol synthase activity	1
GOTERM_MF_ALL	GO:0004143~diacylglycerol kinase activity	1
GOTERM_MF_ALL	GO:0016783~sulfurtransferase activity	1
GOTERM_MF_ALL	GO:0015089~high affinity copper ion transmembrane trans	1
GOTERM_MF_ALL	GO:0047807~cytokinin 7-beta-glucosyltransferase activity	1
GOTERM_MF_ALL	GO:0019202~amino acid kinase activity	1
GOTERM_MF_ALL	GO:0016630~protochlorophyllide reductase activity	1
GOTERM_MF_ALL	GO:0003863~3-methyl-2-oxobutanoate dehydrogenase (2-m	1
GOTERM_MF_ALL	GO:0031127~alpha(1,2)-fucosyltransferase activity	1
GOTERM_MF_ALL	GO:0015326~cationic amino acid transmembrane transport	1
GOTERM_MF_ALL	GO:0008783~agmatinase activity	1
GOTERM_MF_ALL	GO:0016815~hydrolase activity, acting on carbon-nitrogen	1
GOTERM_MF_ALL	GO:0005451~monovalent cation:hydrogen antiporter activit	1
GOTERM_MF_ALL	GO:0003951~NAD+ kinase activity	1
GOTERM_MF_ALL	GO:0003878~ATP citrate synthase activity	1
GOTERM_MF_ALL	GO:0004375~glycine dehydrogenase (decarboxylating) acti	1
GOTERM_MF_ALL	GO:0004066~asparagine synthase (glutamine-hydrolyzing)	1
GOTERM_MF_ALL	GO:0004764~shikimate 5-dehydrogenase activity	1
GOTERM_MF_ALL	GO:0004675~transmembrane receptor protein serine/threon	1

GOTERM_MF_ALL	GO:0004362~glutathione-disulfide reductase activity	1
GOTERM_MF_ALL	GO:0004523~ribonuclease H activity	1

Category	Term	Gene Count
KEGG_PATHWAY	ath01061:Biosynthesis of phenylpropanoids	33
KEGG_PATHWAY	ath00940:Phenylpropanoid biosynthesis	18
KEGG_PATHWAY	ath00196:Photosynthesis	7
PANTHER_PATHWAY	P00006:Apoptosis signaling pathway	9
KEGG_PATHWAY	ath00680:Methane metabolism	13
PANTHER_PATHWAY	P04392:P53 pathway feedback loops 1	4
KEGG_PATHWAY	ath00710:Carbon fixation in photosynthetic organisms	12
KEGG_PATHWAY	ath01064:Biosynthesis of alkaloids derived from ornithine,	20
KEGG_PATHWAY	ath00360:Phenylalanine metabolism	12
KEGG_PATHWAY	ath00195:Photosynthesis	11
KEGG_PATHWAY	ath04712:Circadian rhythm	6
KEGG_PATHWAY	ath01063:Biosynthesis of alkaloids derived from shikimate	19
PANTHER_PATHWAY	P00022:General transcription by RNA polymerase I	3
PANTHER_PATHWAY	P02730:Asparagine and aspartate biosynthesis	3
KEGG_PATHWAY	ath00960:Tropane, piperidine and pyridine alkaloid biosynt	4
KEGG_PATHWAY	ath00010:Glycolysis / Gluconeogenesis	10
KEGG_PATHWAY	ath00941:Flavonoid biosynthesis	4
KEGG_PATHWAY	ath00480:Glutathione metabolism	7
KEGG_PATHWAY	ath01065:Biosynthesis of alkaloids derived from histidine a	16
KEGG_PATHWAY	ath00250:Alanine, aspartate and glutamate metabolism	6
KEGG_PATHWAY	ath01062:Biosynthesis of terpenoids and steroids	18
KEGG_PATHWAY	ath01066:Biosynthesis of alkaloids derived from terpenoid	15
PANTHER_PATHWAY	P00049:Parkinson disease	8
KEGG_PATHWAY	ath00906:Carotenoid biosynthesis	4
PANTHER_PATHWAY	P02753:Methionine biosynthesis	2
KEGG_PATHWAY	ath00460:Cyanoamino acid metabolism	4
KEGG_PATHWAY	ath00020:Citrate cycle (TCA cycle)	7
KEGG_PATHWAY	ath01070:Biosynthesis of plant hormones	25
PANTHER_PATHWAY	P00046:Oxidative stress response	3
KEGG_PATHWAY	ath00620:Pyruvate metabolism	7
PANTHER_PATHWAY	P00030:Hypoxia response via HIF activation	3
KEGG_PATHWAY	ath00260:Glycine, serine and threonine metabolism	5
PANTHER_PATHWAY	P00045:Notch signaling pathway	2
KEGG_PATHWAY	ath00980:Metabolism of xenobiotics by cytochrome P450	3
KEGG_PATHWAY	ath00750:Vitamin B6 metabolism	2
PANTHER_PATHWAY	P02776:Serine glycine biosynthesis	2
KEGG_PATHWAY	ath00945:Stilbenoid, diarylheptanoid and gingerol biosynth	6
KEGG_PATHWAY	ath00590:Arachidonic acid metabolism	2
KEGG_PATHWAY	ath00860:Porphyrin and chlorophyll metabolism	3
KEGG_PATHWAY	ath00910:Nitrogen metabolism	4
KEGG_PATHWAY	ath00500:Starch and sucrose metabolism	7

KEGG_PATHWAY	ath00051:Fructose and mannose metabolism	4
KEGG_PATHWAY	ath00904:Diterpenoid biosynthesis	2
KEGG_PATHWAY	ath00053:Ascorbate and aldarate metabolism	3
KEGG_PATHWAY	ath04144:Endocytosis	5
PANTHER_PATHWAY	P00003:Alzheimer disease-amyloid secretase pathway	2
PANTHER_PATHWAY	P00019:Endothelin signaling pathway	2
PANTHER_PATHWAY	P00035:Interferon-gamma signaling pathway	2
KEGG_PATHWAY	ath00052:Galactose metabolism	3
KEGG_PATHWAY	ath04650:Natural killer cell mediated cytotoxicity	2
KEGG_PATHWAY	ath00030:Pentose phosphate pathway	4
KEGG_PATHWAY	ath00670:One carbon pool by folate	2
KEGG_PATHWAY	ath00903:Limonene and pinene degradation	5
PANTHER_PATHWAY	P00031:Inflammation mediated by chemokine and cytokine	3
KEGG_PATHWAY	ath00640:Propanoate metabolism	2
PANTHER_PATHWAY	P00052:TGF-beta signaling pathway	2
KEGG_PATHWAY	ath00230:Purine metabolism	7
KEGG_PATHWAY	ath00920:Sulfur metabolism	2
KEGG_PATHWAY	ath00908:Zeatin biosynthesis	2
PANTHER_PATHWAY	P00029:Huntington disease	4
KEGG_PATHWAY	ath00900:Terpenoid backbone biosynthesis	3
KEGG_PATHWAY	ath00650:Butanoate metabolism	2
PANTHER_PATHWAY	P00024:Glycolysis	2
PANTHER_PATHWAY	P00032:Insulin/IGF pathway-mitogen activated protein kina	2
KEGG_PATHWAY	ath00270:Cysteine and methionine metabolism	4
KEGG_PATHWAY	ath00130:Ubiquinone and other terpenoid-quinone biosynth	2
PANTHER_PATHWAY	P00036:Interleukin signaling pathway	2
PANTHER_PATHWAY	P02738:De novo purine biosynthesis	2
KEGG_PATHWAY	ath00100:Steroid biosynthesis	2
PANTHER_PATHWAY	P00047:PDGF signaling pathway	3
KEGG_PATHWAY	ath00450:Selenoamino acid metabolism	2
KEGG_PATHWAY	ath03040:Spliceosome	6
KEGG_PATHWAY	ath03020:RNA polymerase	2
PANTHER_PATHWAY	P00018:EGF receptor signaling pathway	3
KEGG_PATHWAY	ath00290:Valine, leucine and isoleucine biosynthesis	2
KEGG_PATHWAY	ath00040:Pentose and glucuronate interconversions	2
KEGG_PATHWAY	ath00630:Glyoxylate and dicarboxylate metabolism	2
PANTHER_PATHWAY	P00021:FGF signaling pathway	3
KEGG_PATHWAY	ath00280:Valine, leucine and isoleucine degradation	2
PANTHER_PATHWAY	P00034:Integrin signalling pathway	2
KEGG_PATHWAY	ath00380:Tryptophan metabolism	2
KEGG_PATHWAY	ath00190:Oxidative phosphorylation	7
KEGG_PATHWAY	ath00400:Phenylalanine, tyrosine and tryptophan biosynthes	2
KEGG_PATHWAY	ath00240:Pyrimidine metabolism	3
KEGG_PATHWAY	ath00520:Amino sugar and nucleotide sugar metabolism	3
KEGG_PATHWAY	ath00330:Arginine and proline metabolism	2
KEGG_PATHWAY	ath04120:Ubiquitin mediated proteolysis	3
KEGG_PATHWAY	ath03010:Ribosome	9

KEGG_PATHWAY	ath00300:Lysine biosynthesis	1
KEGG_PATHWAY	ath00944:Flavone and flavonol biosynthesis	1
KEGG_PATHWAY	ath00740:Riboflavin metabolism	1
KEGG_PATHWAY	ath00071:Fatty acid metabolism	1
KEGG_PATHWAY	ath00561:Glycerolipid metabolism	1
KEGG_PATHWAY	ath00785:Lipoic acid metabolism	1
KEGG_PATHWAY	ath03018:RNA degradation	1
KEGG_PATHWAY	ath03060:Protein export	1
KEGG_PATHWAY	ath00901:Indole alkaloid biosynthesis	1
KEGG_PATHWAY	ath00562:Inositol phosphate metabolism	1
KEGG_PATHWAY	ath04130:SNARE interactions in vesicular transport	1
KEGG_PATHWAY	ath00902:Monoterpenoid biosynthesis	1
KEGG_PATHWAY	ath00061:Fatty acid biosynthesis	1
KEGG_PATHWAY	ath04070:Phosphatidylinositol signaling system	1
PANTHER_PATHWAY	P02778:Sulfate assimilation	1
PANTHER_PATHWAY	P00059:p53 pathway	1
PANTHER_PATHWAY	P00057:Wnt signaling pathway	1
PANTHER_PATHWAY	P00016:Cytoskeletal regulation by Rho GTPase	1
PANTHER_PATHWAY	P02743:Formyltetrahydroformate biosynthesis	1
PANTHER_PATHWAY	P02771:Pyrimidine Metabolism	1
PANTHER_PATHWAY	P00002:Alpha adrenergic receptor signaling pathway	1
PANTHER_PATHWAY	P02734:Chorismate biosynthesis	1
PANTHER_PATHWAY	P02741:Flavin biosynthesis	1
PANTHER_PATHWAY	P05918:p38 MAPK pathway	1
PANTHER_PATHWAY	P02773:S adenosyl methionine biosynthesis	1
PANTHER_PATHWAY	P02750:Lipoate_biosynthesis	1
PANTHER_PATHWAY	P02744:Fructose galactose metabolism	1
PANTHER_PATHWAY	P02727:Androgen/estrogene/progesterone biosynthesis	1
PANTHER_PATHWAY	P02772:Pyruvate metabolism	1
PANTHER_PATHWAY	P02745:Glutamine glutamate conversion	1
PANTHER_PATHWAY	P04397:p53 pathway by glucose deprivation	1
PANTHER_PATHWAY	P00015:Circadian clock system	1
PANTHER_PATHWAY	P02784:Tyrosine biosynthesis	1
PANTHER_PATHWAY	P02765:Phenylalanine biosynthesis	1
PANTHER_PATHWAY	P02763:Peptidoglycan biosynthesis	1

Supplementary Table S8. MALDI TOF-TOF MSMS based identification of differentially accumulated proteins in combined stress treated *ein*2.

SSP no.[a]	Th. Mr/pI Exp. Mr/pI[b]	Avg Fold change[c]	Protein (Taxonomy)	Accession No.[d]	Mascot Score[e]
2403	41.735/5.31 44.70/5.40	0.085	Heat shock protein 70 (*Arabidopsis thaliana*)	gi|6746592	185
4401	77.105/5.13 46.60/5.70	0.65	ACT7 (actin 7) (*A. thaliana*)	gi|15242516	180
3505	255.2/6.0 50.70/5.50	0.725	Acetyl-CoA carboxylase (*Aspergillus clavatus*)	gi|121698358	60
9002	7.5/6.54 13.20/7.0	0.55	Cold shock transcriptional regulator CspA (*Vibrioparahaemolyticus*)	gi|28901144	49

1102	24.70/9.12<br>20.60/5.1	0.67	Peroxiredoxin type 2, putative (*A. thaliana*)	gi|15231718	290
7501	53.15/6.23<br>58.80/6.3	0.165	RUBISCO Large subunit (*Tillandsia multicaulis*)	gi|53831942	152
8101	18.72/6.15<br>25.20/6.6	0.65	Hypothetical	gi|164688345	46
4603	52.95/5.87<br>67.20/5.60	0.59	RUBISCO Large subunit (*A. thaliana*)	gi|7525041	584
8309	10.35/9.40<br>41.30/7.00	0.76	Cystatin A2 (*Dictyostelium discoideum*)	gi|66813992	67
1802	76.50/5.07<br>93.80/5.30	0.24	CPHSC70-1 (chloroplast heat shock protein 70-1) (*A. thaliana*)	gi|15233779	901

1901	64.00/4.82<br>150.00/5.10	0.27	PATL1 (PATELLIN 1); (*A. thaliana*)	gi|15218382	130
7001	14.70/5.69<br>13.3/6.30	0.054	F1O19.10/F1O19.10 (*A. thaliana*)	gi|13926229	383
1104	28.20/8.83<br>20.50/5.20	0.70	Peptidyl-prolyl cis-trans isomerase ROC4 (*A. thaliana*)	gi|15228674	154
3003	20.35/7.58<br>13.90/5.50	0.75	RuBisCO small subunit 2B (RBCS-2B) (*A. thaliana*)	gi|15240903	163
4803	79.96/5.94<br>95.9/5.60	0.43	Transketolase, putative (*A. thaliana*)	gi|18411711	397
4804	51.50/5.56<br>90.80/5.60	0.46	Thioglucoside glucohydrolase	gi|30690089	254

			1(TGG1) (*A. thaliana*)		
2803	68.81/5.11<br>89.50/5.40	0.32	ATP synthase<br>(*A. thaliana*)	gi|15219234	146
3604	63.809/6.21<br>76.50/5.50	0.68	Chaperonin-60 beta<br>(*A. thaliana*)	gi|15222729	131
2701	54.107/4.98<br>68.40/5.30	0.6	Vacuolar ATP synthase<br>(*A. thaliana*)	gi|15222929	131
7804	64.41/6.01<br>84.60/6.40	0.66	NADP-Malic enzyme<br>(*A. thaliana*)	gi|15239146	194
4101	23.758/7.71<br>20.10/5.60	0.72	PSBP-1<br>(Oxygen evolving enhancer protein2)	gi|186478207	126

			(*A. thaliana*)		
2301	42.418/6.17<br>38.70/5.30	0.40	SBPASE (Sedoheptulose bisphosphatase)	gi|15228194	57
3101	24.80/6.18<br>23.3/5.50	0.50	NADH-ubiquinone oxidoreductase chain I (*Helicobacter pylori P12*)	gi|210135427	51
1106	21.63/5.20<br>16.70/5.30	0.68	hypothetical protein RTM1035_17367 (*Roseovarius sp.*)	gi|149201417	45
5101	9.38/9.60<br>17.50/5.80	2.23	Predicted protein (*Populus trichocarpa*)	gi|224124102	27
8308	36.91/6.67<br>41.70/6.80	1.68	GAPC-2; glyceraldehyde-	gi|15222848	191

			3-phosphate dehydrogenase (*A. thaliana*)		
5303	14.27/5.09 37.40/5.80	3.88	Autonomous glycyl radical cofactor GrcA (*A. thaliana*)	gi|110642741	54
5302	20.35/7.58 40.40/5.80	1.90	Ribulose bisphosphate carboxylase small chain 2B (*A. thaliana*)	gi|15240903	78
5305	57.19/5.97 39.60/5.90	2.1	Predicted protein (*Physcomitrella patens subsp. Patens*)	gi|168034232	39
5301	11.50/9.35 37.40/5.70	3.18	DNA binding protein HU (*Hydrogenivirga sp.*)	gi|163782099	69

7504	34.50/5.56<br>58.80/6.40	1.26	Pyridoxine/pyridoxal 5-phosphate biosynthesis enzyme (*Cenarchaeum symbiosum A*)	gi|118575273	76
6604	53.34/6.04<br>65.10/6.20	30.58	Large subunit of riblose-1,5-bisphosphatecarboxylase/oxygenase (*Aethionema cordifolium*)	gi|139390356	358

Identified differentially accumulated proteins in combined stress treated Col-0 and ein2 plants. [a]Assigned sample protein . [b]Th Mr/pI, theoretical mass of protein in kDa and pI; Exp Mr/pI, experimental mass of protein in kDa and pI. [c]Average spot intensity fold change. [d]NCBI accession number of identified protein spots. [e]Statistical probability of true positive identification of predicted proteins calculated by MASCOT (http://www.matrixscience.com).

Supplementary Table S9. MALDI TOF-TOF MSMS based identification of differentially accumulated proteins in combined stress treated *aba*1.6.

SSP no.[a]	Th. Mr/pI<br>Exp. Mr/pI[b]	Avg Fold change[c]	Protein (Taxonomy)	Accession No.[d]	Mascot Score[e]
6209	35.00/5.91<br>26.00/6.0	0.76	PSBO-2/PSBO2 (Photosynthetic MU-subunit)<br>(*A. thaliana*)	gi|15230324	81
1001	35.13/5.66<br>17.40/4.8	0.71	oxygen-evolving protein<br>(*A. thaliana*)	gi|22571	83
8104	13.00/9.13<br>21.00/6.5	0.26	Peptidyl-prolyl cis-trans isomerase Pin1<br>(*A. thaliana*)	gi|15227956	64
5201	28.20/8.83<br>30.3/5.79	0.29	Peptidylprolyl isomerase ROC4<br>(*A. thaliana*)	gi|21555831	124

5001	25.65/5.78<br><br>17.2/5.70	0.51	ATGSTU19 (Glutathione transferase U19)<br><br>(*A. thaliana*)	gi|18411929	70
8501	101.96/5.43<br><br>55.5/6.40	0.15	Lipoxygenase LOX2<br><br>(*A. thaliana*)	gi|17065070	132
202	48.50/7.55<br><br>29.00/4.50	0.65	RCA (Rubisco activase)<br><br>(*A. thaliana*)	gi|30687999	158
6001	19.93/4.83<br><br>13.60/5.90	0.49	Glycine-rich RNA-binding protein, putative (*Ricinus communis*)	gi|223549240	82
4504	47.41/6.43<br><br>51.0/5.50	0.33	GS2 (Glutamine synthetase 2)<br><br>(*A. thaliana*)	gi|15238559	113

1202	17.51/5.91<br>29.6/4.70	0.77	Major latex protein-related / MLP-related proteins (*A. thaliana*)	gi|15236566	76
8701	56.19/8.49<br>74.1/6.40	0.58	Cytochrome P450, family 81, subfamily D, polypeptide 2 (*A. thaliana*)	gi|15235535	72
3405	48.36/5.84<br>41.1/5.30	0.72	RUBISCO large subunit (*Falcatifolium papuanum*)	gi|13548844	89
4504	53.93/5.38<br>51.0/5.5	0.33	ATP synthase CF1 beta subunit (*A. thaliana*)	gi|7525040	215

6101	53.93/5.38<br><br>51.0/5.5	0.73	Phosphoinositide 4-kinase gamma 7<br><br>(*A. thaliana*)	gi|18395629	77
5402	59.67/6.18<br><br>37.9/5.6	0.33	ATP synthase subunit beta-1, mitochondria<br><br>(*A. thaliana*)	gi|26391487	246
9601	72.99/5.63<br><br>66.1/6.9	0.28	HSO70 2<br><br>(*A. thaliana*)	gi|15242459	74
3302	47.039/5.89<br><br>34.0/5.20	0.54	CORI3 (Coronatine induced1INDUCED1 transaminase<br><br>(*A. thaliana*)	gi|15236533	196

8201	26.49/7.80<br>25.8/6.40	0.46	30S ribosomal protein S2 (*Francisella tularensis subsp. mediasiatica FSC147*)	gi|187932155	72
5301	59.57/8.23<br>32.9/5.60	0.69	Ankyrin repeat-containing protein (*A. thaliana*)	gi|15238604	76
3502	50.11/5.91<br>54.1/5.30	0.71	Phosphoglycerate kinase (*A. thaliana*)	gi|15230595	90
6501	44.10/6.79<br>50.80/5.80	0.55	HCF136 (High chlorophyll fluorescence 136) (*A. thaliana*)	gi|15237225	307

2204	49.49/5.16<br>29.1/5.10	0.58	DNA replication protein DnaC (*Selenomonas flueggei ATCC 43531*)	gi|238885609	73
8002	41.17/8.51<br>18.8/6.50	0.24	ATLFNR2 (LEAF FNR 2)<br>(*A. thaliana*)	gi|15223753	77
3003	20.21/7.59<br>18.00/5.30	0.10	RBCS1A; ribulose-bisphosphate carboxylase<br>(*A. thaliana*)	gi|15219826	98
9102	32.16/5.46<br>21.20/6.80	0.109	PBP1 (PYK10-BINDING PROTEIN 1)<br>(*A. thaliana*)	gi|15228198	115

1601	105.00/5.79<br>64.9/4.5	2.428	Mismatch repair protein<br>(*A. thaliana*)	gi|4139230	66
3602	55.28/5.18<br>57.0/5.2	1.57	ATPase alpha subunit<br>(*Capsella bursa-pastoris*)	gi|139387237	107
2602	55.32/5.19<br>60.6/5.1	2.48	ATP synthase CF1 alpha subunit<br>(*A. thaliana*)	gi|7525018	490
2201	24.99/5.12<br>24.20/4.90	2.80	Chlorophyll a/b binding protein (LHCP AB 180)<br>(*A. thaliana*)	gi|16374	80
101	30.29/6.14<br>24.0/4.30	3.23	Aspartate carbamoyltransferase	gi|159487733	70

			(Chlamydomonas reinhardtii)		
1604	54.74/4.98<br>61.00/4.70	4.37	Nucleotide-binding subunit of vacuolar ATPase<br>(*A. thaliana*)	gi|166627	71
1201	24.19/9.45<br>24.4/4.6	1.82	Rop3 small GTP binding protein<br>(*Zea mays*)	gi|162464413	57
5402	52.95/5.87<br>37.9/5.60	1.28	RUBISCO large subunit<br>(*A. thaliana*)	gi|7525041	188
1201	26.49/7.80<br>24.5/4.60	1.42	30S ribosomal protein S2<br>(*Francisella tularensis subsp.*	gi|187932155	72

			mediasiatica FSC147)		
3601	49.49/5.16<br>73.40/5.20	4.7	DNA replication protein DnaC (*Selenomonas flueggei ATCC 43531*)	gi|238885609	73

Identified differentially accumulated proteins in combined stress treated Col-0 and *aba*1.6plants. [a]Assigned sample protein . [b]Th Mr/pI, theoretical mass of protein in kDa and pI; Exp Mr/pI, experimental mass of protein in kDa and pI. [c]Average spot intensity fold change. [d]NCBI accession number of identified protein spots. [e]Statistical probability of true positive identification of predicted proteins calculated by MASCOT (http://www.matrixscience.com).

Supplementary Table S10. List of primers used in quantitative and semi-quantitative RT-PCR.

Genes	Forward Primer (5’- 3’)	Reverse Primer (5’-3’)	Amplicon size in bp (Ref. No.)
*CAD*4	ATGGGAAGTGTAGAAGCAGGA	TACCGCAGCATCCAACGACC	307 (NM_112832.3)

*TPX*2	ATGGCTCCAATTACT GTCGGC	AAACTTCACATGCTT GTTCTCTG	300 (NM_105269.4)
*HSP*70	ATGTTGGGAATGAG AACTGTGT	TCTCACCCATATACCG AAGCCG	306 (NM_101038.4)
*APX*2	ATGGTGAAGAAGAGTT ACCCG	CTAGCTGGTAGAAGTC AGCAT	302 NM_111798.3
ACTIN3	ATGGCTGATGGTGAAG ATATTCA	CATTGTAGAAAGTATG ATGCCAG	283 NM_115235.3
*CCA*1	ATGGAGACAAATTCGT CTGGA	ATTGTTTGGTTTACGCT TAGGC	306 NM_180129.3
*GSTU*23	ATGGAGGAAGAGATTA TCCTATT	GTAATCTGCCCAAAATC TAGCT	303 NM_106481.3
Monophosphatase	ATGGCGGACAATGATC AGTTTT	ACCCGTGAACGAAATT GGTTG	298 XM_007026216.1
LHY	ATGGATACTAATACAT CTGGAG	AGGCGGAATTTCTATGT CCAA	279 NM_001035847.1
*AP*2	ATGGCGGATCTCTTCG GTG	TGTTTTGGTAATGGAA CTGAGA	304 AT1G36060
SHN2	ATGGTACATTCGAAG AAGTTCC	ACGGTGTCTGGTCTTTA CAGT	307 AT5G25390
CCA1	ATGGAGACAAATTCGT	ATTGTTTGGTTTACGCT	

	CTGGA	TAGGC	306 NM_180129.3
*GSTF*8	ATGGGAGCAATTCAAG CTCGT	AATTTGACCGAAGGGG TTGAG	309 NM_180148.4
*GSTU*14	ATGGCTCAGAACGATA CAGTG	AGGCATTAGAAGGAAG GATGGAT	283 NM_102476.3
CIPK	ATGGATAAAAACGGCA TAGTTTT	AGCTCACCGCCTTTAA CGTA	290 NM_123950.3
*NCED*9	ATGACGATAATAACCA TTATTTCT	TACTAGTTTCTTGTTTC TAAGTG	285 NM_106486.2
*HSP*90.1	ATGGCGGATGTTCAG ATGGCT	TTCCCAAGTTGTTCAC CAAAT	301 NM_124642.3
*HSP*17.4	ATGTCTCTAGTTCCG AGCTTT	TCACTCTTCTCTTCAT TCTCA	300 NM_114492.4
*HSP*70B	ATGGCGACGAAATC AGAGAAA	GGGGCCGGAAACAAC CTTGA	300 NM_101471.2
*MYB*108	ATGGATGAAAAAGG AAGAAGCTTG	CTATTCCCCATTTGAC CCGAATA	725 (NM_111525.3)
*HSP*17.6	ATGTCGCTGATTCCA AGCT	ACGATGCCACGTGTC CTGCT	300 NM_104679.3
